# Supplementary material for: Stereodefined polymetalloid alkenes synthesis via stereoselective boron-masking of polyborylated alkenes
Source: Nat Commun. 2023 Apr 11;14:2022. doi: 10.1038/s41467-023-37733-0 (PMC10090189; doi:10.1038/s41467-023-37733-0)
Supplement: Supplementary file 1 — Supplementary Information [file 41467_2023_37733_MOESM1_ESM.pdf]

Supplementary Information for

Stereodefined polyMetalloid Alkenes Synthesis via Stereoselective Boron-Masking of polyBorylated Alkenes

Nadim Eghbarieh[1], Nicole Hanania[1], and Ahmad Masarwa[1]*

[1]Institute of Chemistry, The Center for Nanoscience and Nanotechnology, Casali Center for Applied Chemistry, The Hebrew University of Jerusalem, Jerusalem 9190401 (Israel).

*Corresponding author: E-mail: Ahmad.Masarwa1@mail.huji.ac.il.

Table of Contents

1. Supplementary Notes……………………………………………………..….……………......3

1.1. Materials and General Remarks…………………………………………………………....3

2. Supplementary Methods………………………………………………………….........4

2.1 Procedures (A-F) and Characterization for Starting Materials [**V-(1-4)**]………………......4

2.2 General Procedure G and Characterization for Triflourination Products (**1**)…………….…...20

2.3 General Procedure H and Characterization for *gem*-(Bpin,Bdan)-alkene (**2**)…..................36

2.4 Procedure I and Characterization for *gem*-(Bdan,Bdan)-alkene (**2a-dan**)............….….….49

2.5 General Procedure J and Characterization for *gem*-(Bpin,BMIDA)-alkene (**3, 4**)............51

2.6 General Procedure K and Characterization for Products (**5a, 5b and 5c**) …...……...…...77

2.7 General Procedure L and Characterization for Products (**6a, 6b and 6c**) …………..………80

2.8 General Procedure M and Characterization for Products (**5b, 5d and 5e**) ……….....……83

2.9 General Procedure N and Characterization for Products (**7**) ………….…………………...86

2.10 General Procedure O and Characterization for Products (**8**) ………………….……........87

2.11 General Procedure P and Characterization for Products (**9**)………..…….…….…….…...88

2.12 Procedure Q and Characterization for *gem*-(Bpin,Bdan) Product (**10**) ….…….………….89

2.13 Procedure R and Characterization for *gem*-(Bpin,Bdan) Product (**11**) ..………...…........90

3. Supplementary Discussion ………………………………………………………..92

3.1 X-Ray Crystallography Data……………………………………………..……92

3.2 NMR Spectra………….…………………………..…….……..…………………99

3.3 2D-NMR NOESY Analysis…………………………………...…..…………242

4 Supplementary References………………………………………..…..….……260

1. Supplementary Notes

1.1. Materials and General Remarks

Unless otherwise stated, reactions were performed in oven-dried glassware fitted with rubber septa under inert atmosphere (N_2) and were stirred with teflon-coated magnetic stirring bars. Materials and chemicals were purchased from Sigma-Aldrich Inc., Combi-Blocks Inc., Alfa Aesar., and other commercial suppliers. Liquid reagents and solvents were transferred via syringe using standard Schlenk techniques. Solvents DMSO, toluene, methanol and acetonitrile were used as commercial grade and used as received with no further drying. Tetrahydrofuran (THF), diethyl ether (Et_2O), and dichloromethane (CH_2Cl_2) were used from a solvent purification system. All other reagents were used as received unless otherwise noted. Thin layer chromatography (TLC) was performed using silica gel 60 F-254 precoated plates (0.25 mm) and visualized by UV irradiation ƛ=232 nm, CAM stain, $KMnO_4$ stain, and other stains. Silica gel of particle size 230-400 mesh was used for flash chromatography, Flash chromatography (FC) was performed using CombiFlash, with SiO_2 columns. ^{1}H and ^{13}C NMR spectra were recorded on 400, 500 MHz spectrometers with ^{13}C operating frequencies of 101, 126 MHz, ^{11}B operating frequencies of 128, 160 MHz, ^{19}F operating frequencies of 376, 471 MHz respectively. ^{1}H and ^{13}C NMR spectra were referenced to TMS as an internal standard with a deuterated solvent unless otherwise stated. X-Ray structures were visualized with PLATON [1], most of the hydrogen atoms are removed for clarity. Chemical shifts (δ) are reported in ppm relative to the residual solvents ($CDCl_3$) signal (δ = 7.26 for ^{1}H NMR and δ = 77.0 for ^{13}C NMR) and (DMSO-d_6) signal (δ = 2.50 (ppm) for ^{1}H NMR and δ = 39.5 (septet) for ^{13}C NMR). Data for ^{1}H NMR spectra are reported as follows: chemical shift (multiplicity, coupling constants, and number of hydrogen). Abbreviations are as follows: s (singlet), d (doublet), t (triplet), q (quartet), p (pentate), m (multiplet), brs (broad singlet). High-Resolution Mass Spectrometry (HRMS) using acetonitrile or methanol as solvent.

2. Supplementary Methods

2.1. Procedures (A-F) and Characterizations for Starting Materials [V-1(4)]

Procedure A: The Synthesis of *gem*-diborylalkene (V-1a and V-3w):

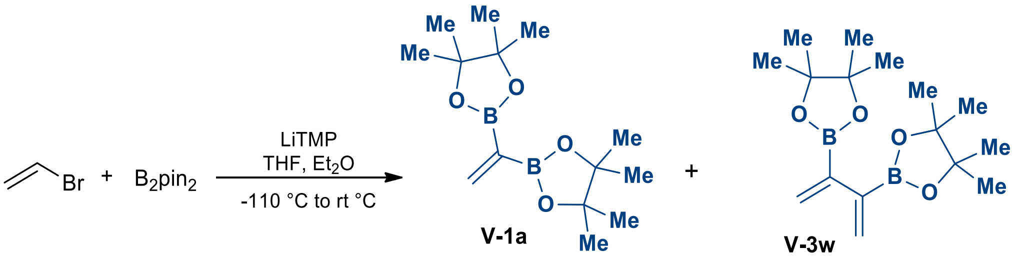

Products **V-1a** and **V-3w** were prepared according to a previous reported procedure.[2] Under inert conditions (N_2), bis(pinacolato)diboron-(B_2pin_2) (2.5 g, 9.00 mmol) was dissolved in a mixture of dry ether 30 ml and pentane 7.5 ml followed by addition of vinyl bromide (1.0 M in THF) (27 mL, 27 mmol), the reaction mixture was cooled to -110 °C and stirred for 10 min. Next, a freshly prepared lithium tetramethylpiperidide (LiTMP) (27 mmol) solution was added dropwise, with slow addition. The reaction mixture was kept at -110 °C for further 30 minutes, then the residue was heated gradually to rt and stirred for another 1 h. The reaction mixture was quenched with a solution of 10 % $NaHSO_4$ (15 mL) and H_2O (15 mL) and stirred for 5 more minutes. The mixture was extracted with Et_2O (3 × 30 mL). The organic layers were combined and washed with brine solution, dried over $MgSO_4$, filtered and concentrated under reduced pressure (by evaporator). The crude material was purified by silica gel column chromatography using hexane/EtOAc as an eluent. Product **V-1a** was isolated in 55% yield as light yellowish solid and product **V-3w** was isolated in 35% yield as a white solid.

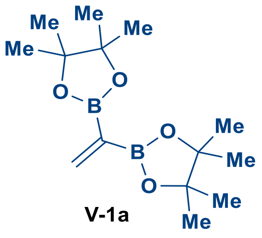

V-1a

2,2'-(ethene-1,1-diyl)bis(4,4,5,5-tetramethyl-1,3,2-dioxaborolane) **(V-1a):**

Prepared according to general procedure A, product **V-1a** was isolated in (1.386 gr, 55% yield) as a yellowish solid.

R_f = 0.34 (10% EtOAc in hexane).

^{1}H NMR (400 MHz, $CDCl_3$) δ: 6.58 (s, 2H), 1.26 (s, 24H).

^{11}B NMR (128 MHz, $CDCl_3$) δ: 30.6.

The spectral data are consistent with those reported in the literature.[2]

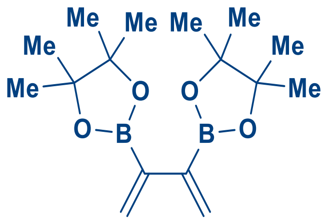

V-3w

***2,2'-(buta-1,3-diene-2,3-diyl)bis(4,4,5,5-tetramethyl-1,3,2-dioxaborolane)* (V-3w):**

Prepared according to general procedure A, product **V-3w** was isolated in (0.963 gr, 35% yield) as a white solid.

R_f = 0.47 (10% EtOAc in hexane).

^{1}H NMR (400 MHz, $CDCl_3$) δ: 5.93 (d, J = 3.40 Hz, 2H), 5.85 (d, J = 3.68 Hz, 2H), 1.27 (s, 24H).

^{13}C NMR (101 MHz, $CDCl_3$) δ: 130.73, 83.62, 24.93. (C-B) Carbon signal not observed due to quadrupolar relaxation.[3]

^{11}B NMR (128 MHz, $CDCl_3$) δ: 30.1.

The spectral data are consistent with those reported in the literature.[2]

General Procedure B: Synthesis of Substituted *gem*-diborylalkene [V-1(b-m)] and V-3j:

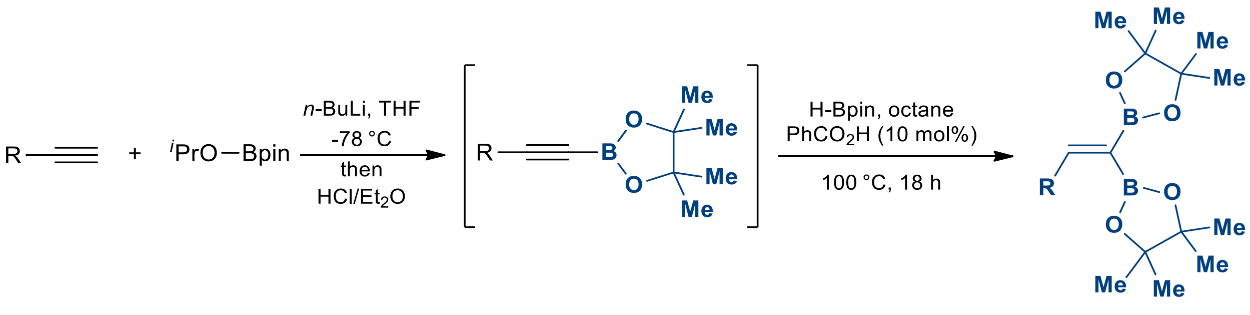

[V-1(b-m)], V-3j
R = Aryl, Alkyl

Products **[V-1(b-m)]** and **V-3j** were prepared according to a literature reported procedure with slight modification.[4, 5] To a cold (-78 °C) solution of alkyne (1.2 mmol) in THF (3.0 mL) inside a

50 mL Schlenk tube under N_2, *n*-BuLi (2.5M in hexane) (0.48 mL, 1.2 mmol) was added in a dropwise addition. The reaction mixture was stirred for 1 hour at -78 °C. Then a THF solution (2.5 mL) of 4,4,5,5-tetramethyl-2-(1-methylethoxy)-1,3,2-dioxaborolane (312.0 mg, 1.0 mmol) was added to the reaction mixture at -78 °C. After being stirred for 2 hours at -78 °C, the reaction mixture was quenched with 1.0 M HCl/Et_2O solution (1.25 mL, 1.25 mmol), and the mixture was warmed to room temperature with additional 1 hour stirring. Filtration and evaporation afforded the crude material, that was used for the next step without any further purifications.

The crude material was taken in octane (1.0 mL) with benzoic acid (11.0 mg, 10 mol%), and a dropwise addition of pinacolborane (H-Bpin) (0.75 mL, 5.0 mmol) under nitrogen atmosphere, and the reaction was stirred at 100 °C for 18 hours. After cooling to room temperature, the reaction mixture was concentrated under vacuum (by evaporator), and the crude material was purified by column chromatography to afford the desired product.[4,5]

Of Note: Compound (**V-2u** and **V-2t**) were purchased as commercially available reagents.

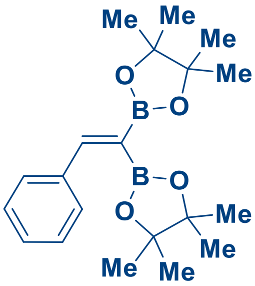

V-1b

2,2'-(2-phenylethene-1,1-diyl)bis(4,4,5,5-tetramethyl-1,3,2-dioxaborolane) (V-1b):

Prepared according to general procedure B, product **V-1b** was isolated in (0.384 gr, 90% yield) as a colorless oil.

R_f = 0.53 (10% EtOAc in hexane).

^{1}H NMR (400 MHz, $CDCl_3$) δ: 7.64 (s, 1H), 7.41 (d, *J* = 6.8 Hz, 2H), 7.24 - 7.18 (m, 3H), 1.24 (s, 12), 1.21 (s, 12).

The spectral data are consistent with those reported in the literature.[4]

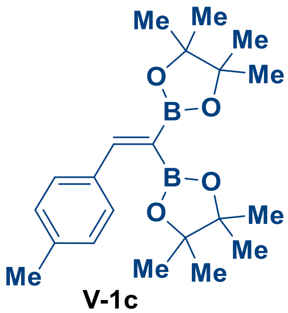

V-1c

2,2'-(2-(p-tolyl)ethene-1,1-diyl)bis(4,4,5,5-tetramethyl-1,3,2-dioxaborolane) (V-1c):

Prepared according to general procedure B, product **V-1c** was isolated in (0.168 gr, 83% yield) as a colorless oil.

R_f = 0.55 (10% EtOAc in hexane).

^{1}H NMR (400 MHz, $CDCl_3$) δ: 7.68 (s, 1H), 7.39 (d, *J* = 8.0 Hz, 2H), 7.10 (d, *J* = 8.0 Hz, 2H), 2.25 (s, 3H), 1.25 (s, 12H), 1.20 (s, 12H).

The spectral data are consistent with those reported in the literature.[5]

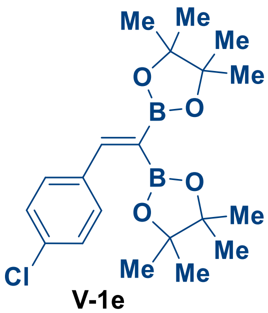

V-1e

2,2'-(2-(4-chlorophenyl)ethene-1,1-diyl)bis(4,4,5,5-tetramethyl-1,3,2-dioxaborolane) (V-1e):

Prepared according to general procedure B, product **V-1e** was isolated in (0.346 gr, 74% yield) as a white solid.

R_f = 0.54 (10% EtOAc in hexane).

^{1}H NMR (400 MHz, $CDCl_3$) δ: 7.57 (s, 1H), 7.35 (d, *J* = 7.6 Hz, 2H), 7.19 (d, *J* = 7.2 Hz, 2H), 1.24 (s, 12H), 1.20 (s, 12H).

The spectral data are consistent with those reported in the literature.[6]

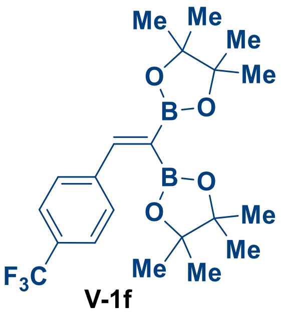

V-1f

2,2'-(2-(4-(trifluoromethyl)phenyl)ethene-1,1-diyl)bis(4,4,5,5-tetramethyl-1,3,2-dioxaborolane) (V-1f):

Prepared according to general procedure B, product **V-1f** was isolated in (0.412 gr, 81% yield) as a yellow oil.

R_f = 0.53 (10% EtOAc in hexane).

^{1}H NMR (400 MHz, $CDCl_3$) δ: 7.70 (s, 1H), 7.59 - 7.53 (m, 4H), 1.31 (s, 12H), 1.29 (s, 12H). The spectral data are consistent with those reported in the literature.[5]

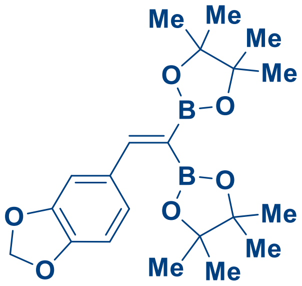

V-1g

2,2'-(2-(Benzo[d][1,3]dioxol-5-yl)ethene-1,1-diyl)bis(4,4,5,5-tetramethyl-1,3,2- dioxaborolane) (V-1g):

Prepared according to previous reported procedure [6], product **V-1g** was isolated in (0.321 gr, 67% yield) as a white solid.

R_f = 0.43 (10% EtOAc in hexane).

^{1}H NMR (400 MHz, $CDCl_3$) δ: 7.59 (s, 1H), 7.06 (d, *J* = 1.6 Hz, 1H), 6.95 (dd, *J* = 8.0, 1.6 Hz, 1H), 6.73 (d, *J* = 8.0 Hz, 1H), 5.59 (s, 2H), 1.33 (s, 12H), 1.27 (s, 12H). The spectral data are consistent with those reported in the literature. [6]

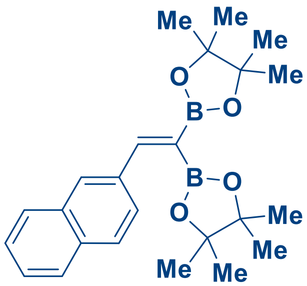

V-1h

2,2'-(2-(Naphthalen-2-yl)ethene-1,1-diyl)bis(4,4,5,5-tetramethyl-1,3,2- dioxaborolane) (V-1h):

Prepared according to general procedure B, product **V-1h** was isolated in (0.326 gr, 67% yield) as a white solid.

R_f = 0.57 (10% EtOAc in hexane).

^{1}H NMR (400 MHz, $CDCl_3$) δ: 7.96 (s, 1H), 7.87 (s, 1H), 7.81 - 7.75 (m, 3H), 7.64 (d, J = 8.4 Hz, 1H), 7.45 (d, J = 8.4 Hz, 2H), 1.34 (s, 12H), 1.30 (s, 12H).

The spectral data are consistent with those reported in the literature.[5]

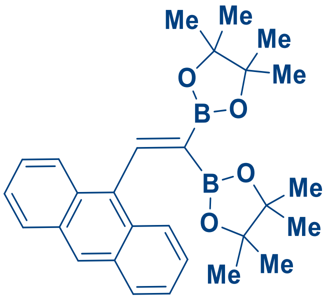

V-1i

2,2'-(2-(anthracen-9-yl)ethene-1,1-diyl)bis(4,4,5,5-tetramethyl-1,3,2-dioxaborolane) (V-1i):

Prepared according to general procedure B, product **V-1i** was isolated in (0.290 gr, 53% yield) as a yellow solid.

R_f = 0.60 (10% EtOAc in hexane).

^{1}H NMR (400 MHz, $CDCl_3$) δ: 8.52 (s, 1H), 8.35 (s, 1H), 8.25 - 8.21 (m, 2H), 7.97 - 7.94 (m, 2H), 7.47-7.41 (m, 4H), 1.38 (s, 12H), 0.61 (s, 12H).

^{13}C NMR (101 MHz, $CDCl_3$) δ 154.25, 135.77, 131.35, 129.08, 128.31, 127.06, 126.22, 125.20, 83.57, 83.05, 25.22, 24.00. (C-B) Carbon signal was not observed due to quadrupolar relaxation.[3]

^{11}B NMR (128 MHz, $CDCl_3$) δ: 31.9.

HRMS (ESI) Calcd for $[C_{28}H_{34}B_2O_4 + H]^+$ $[M+H]^+$: m/z 457.2721, found 457.2719.

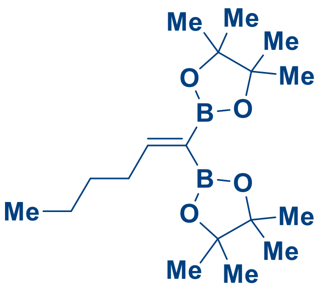

V-1j

2,2'-(hex-1-ene-1,1-diyl)bis(4,4,5,5-tetramethyl-1,3,2-dioxaborolane) (V-1j):

Prepared according to the general procedure B, product **V-1j** was isolated in (0.288 gr, 70% yield) as a colorless oil.

R_f = 0.53 (10% EtOAc in hexane).

^{1}**H NMR** (400 MHz, $CDCl_3$) δ: 6.89 (t, J = 7.2 Hz, 1H), 2.22 (q, J = 7.3 Hz, 2H), 1.25 (s, 12H), 1.19 (m, 16H), 0.84 (t, J = 7.2 Hz, 3H).

The spectral data are consistent with those reported in the literature.[5]

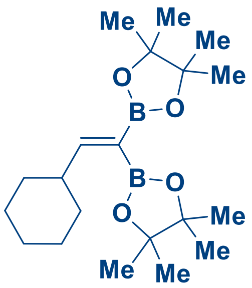

V-1k

2,2'-(2-cyclohexylethene-1,1-diyl)bis(4,4,5,5-tetramethyl-1,3,2-dioxaborolane) (V-1k):

Prepared according to general procedure B, product **V-1k** was isolated in (0.373 gr, 86% yield) as a colorless oil.

R_f = 0.53 (10% EtOAc in hexane).

^{1}**H NMR** (400 MHz, $CDCl_3$) δ: 6.74 (d, J = 8.8 Hz, 1H), 2.26 - 2.22 (m, 1H), 1.70 - 1.61 (m, 4H), 1.29 (s, 12H), 1.28 - 1.22 (m, 16H).

The spectral data are consistent with those reported in the literature.[5]

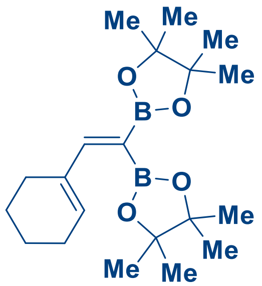

V-1l

2,2'-(2-(cyclohex-1-en-1-yl)ethene-1,1-diyl)bis(4,4,5,5-tetramethyl-1,3,2-dioxaborolane) (V-1l): Prepared according to general procedure B, product **V-1l** was isolated in (0.263 gr, 61% yield) as a colorless oil.

R_f = 0.54 (10% EtOAc in hexane).

^{1}H NMR (400 MHz, $CDCl_3$) δ: 7.18 (s, 1H), 6.01-5.99 (m, 1H), 2.21 - 2.12 (m, 4H), 1.63-1.55 (m, 4H), 1.30 (s, 12H), 1.23 (s, 12H).

The spectral data are consistent with those reported in the literature.[5]

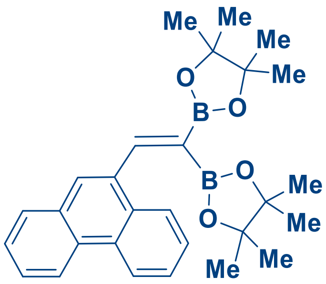

V-3j

2,2'-(2-(phenanthren-9-yl)ethene-1,1-diyl)bis(4,4,5,5-tetramethyl-1,3,2-dioxaborolane) (V-3j): Prepared according to general procedure B, product **V-3j** was isolated in (0.399 gr, 73% yield) as a white solid.

R_f = 0.59 (10% EtOAc in hexane).

^{1}H NMR (400 MHz, $CDCl_3$) δ: 8.68 (dd, J = 16.62, 8.10 Hz, 2H), 8.40 (s, 1H), 8.17 (dd, J = 8.03, 1.37 Hz, 1H), 7.90 (s, 1H), 7.83 (dd, J = 8.07, 1.42 Hz, 1H), 7.68 - 7.54 (m, 4H), 1.34 (s, 12H), 1.15 (s, 12H).

^{13}C NMR (101 MHz, $CDCl_3$) δ: 153.85, 136.91, 131.51, 130.64, 130.60, 130.14, 128.75, 126.66, 126.62, 126.60, 126.53, 126.29, 125.80, 122.76, 122.57, 83.52, 83.32, 24.99, 24.58. (C-B) Carbon signal was not observed due to quadrupolar relaxation.[3]

^{11}B NMR (128 MHz, $CDCl_3$) δ: 33.8.

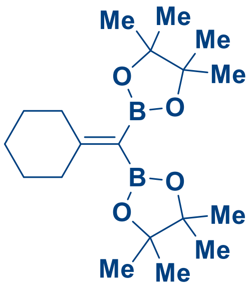

V-1m

2,2'-(cyclohexylidenemethylene)bis(4,4,5,5-tetramethyl-1,3,2-dioxaborolane) (V-1m):

Prepared according to previous reported procedure,[7] product **V-1m** was isolated in (0.167 gr, 40% yield) as a colorless oil.

R_f = 0.58 (10% EtOAc in hexane).

^{1}H NMR (400 MHz, $CDCl_3$) δ: 2.39 - 2.36 (m, 4H), 1.63 - 1.51 (m, 6H), 1.24 (s, 24H).

The spectral data are consistent with those reported in the literature.[7]

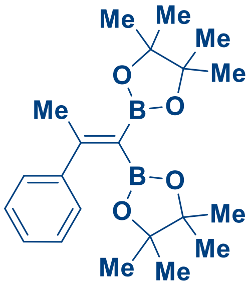

V-1n

2,2'-(2-phenylprop-1-ene-1,1-diyl)bis(4,4,5,5-tetramethyl-1,3,2-dioxaborolane) V-1n):

Prepared according to previous reported procedure, [7] product **V-1n** was isolated in (0.133 gr, 30% yield) as a colorless oil.

R_f = 0.43 (10% EtOAc in hexane).

^{1}H NMR (400 MHz, $CDCl_3$) δ: 7.32 - 7.21 (m, 5H), 2.34 (s, 3H), 1.29 (s, 12H), 1.06 (s, 12H).

The spectral data are consistent with those reported in the literature.[8]

General Procedure C: The synthesis of *gem*-diborylalkene (V-1o):

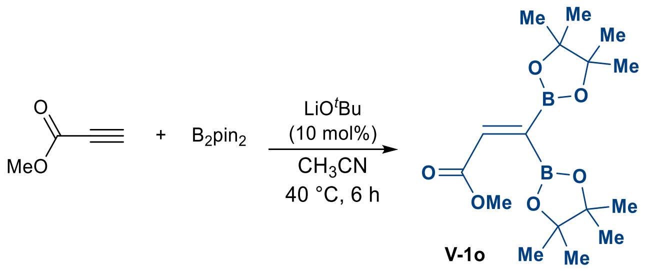

methyl 3,3-bis(4,4,5,5-tetramethyl-1,3,2-dioxaborolan-2-yl)acrylate (V-1o):

Product **V-1o** was prepared according to a literature reported procedure.[7] Inside the glovebox, bis(pinacolato)diboron-(B_2pin_2) (3.81 g, 15 mmol) was placed in a pressure tube containing a magnetic stirring bar. Acetonitrile (30 mL), ethyl propiolate (1.47 g, 15 mmol) and LiO^{t}Bu (120 mg, 1.5 mmol) were sequentially added to the flask. The reaction was stirred for 6 h at 40 °C, and the mixture was filtered through a short plug of silica gel with continues washing with Et_2O. The solvent was removed under reduced pressure (by evaporator). The crude material was then purified by column chromatography to give the pure product (**V-1o**) as a white solid in (4.30 gr, 85% yield). R_f = 0.29 (10% EtOAc in hexane).

^{1}H NMR (400 MHz, $CDCl_3$) δ: 6.77 (s, 1H), 3.75 (s, 3H), 1.36 (s, 12H), 1.25 (s, 12H).

The spectral data are consistent with those reported in the literature.[9]

General procedure D: The Synthesis of 1,2-diborylalkene [V-2(p-r)] and V-2v:

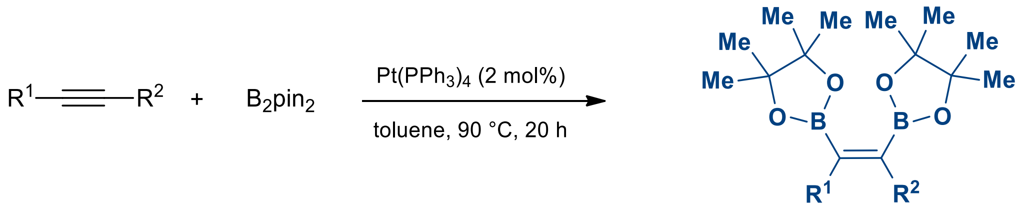

[V-2(p-r)] and V-2v

R^1 = Ar, $Si(R)_3$

R^2 = H, Me

Products **[V-2(p-r)]** and **V-2v** were prepared according to a literature reported procedure with slight modifications.[4] A dry nitrogen flushed 50 mL flask equipped with a magnetic stirring bar was charged with $Pt(PPh_3)_4$ (25.0 mg, 0.03 mmol) and bis(pinacolato)diboron-(B_2pin_2) (257.0 mg, 1 mmol), 5 mL of toluene was then added to the mixture followed by the alkyne (1.0 mmol). The

mixture was stirred overnight at 90 °C. After that the toluene was removed under vacuum (by evaporator) and the crude material was then purified by flash chromatography on silica gel.

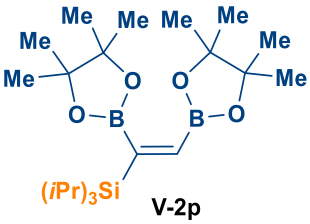

V-2p

(E)-(1,2-bis(4,4,5,5-tetramethyl-1,3,2-dioxaborolan-2-yl)vinyl)triisopropylsilane (V-2p):

Prepared according to general procedure D, product **V-2p** was isolated in (0.348 gr, 80% yield) as a colorless oil.

R_f = 0.51 (5% EtOAc in hexane).

^{1}H NMR (400 MHz, $CDCl_3$) δ: 6.51 (s, 1H), 1.30 (s, 12H), 1.27 (s, 12H), 1.20 - 1.16 (m, 3H), 1.04 (d, J = 7.37, 18H).

^{13}C NMR (101 MHz, $CDCl_3$) δ: 83.59, 83.48, 25.48, 25.06, 18.84, 11.48. (C-B) Carbon signal not observed due to quadrupolar relaxation.[3]

^{11}B NMR (128 MHz, $CDCl_3$) δ: 31.3, 28.1.

HRMS (ESI) Calcd for $[C_{23}H_{46}B_2O_4Si+H]^+$ $[M+H]^+$: m/z 437.3433, found 437.3432.

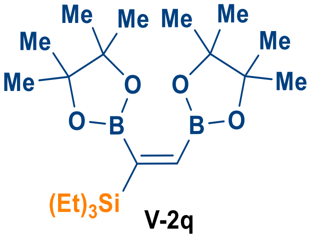

V-2q

(E)-(1,2-bis(4,4,5,5-tetramethyl-1,3,2-dioxaborolan-2-yl)vinyl)triethylsilane (V-2q):

Prepared according to general procedure D, product **V-2q** was isolated in (0.327 gr, 83% yield) as a colorless oil.

R_f = 0.54 (5% EtOAc in hexane).

^{1}H NMR (400 MHz, $CDCl_3$) δ: 6.56 (s, 1H), 1.31 (s, 12H), 1.25 (s, 12H), 0.91 (t, J = 7.88 Hz), 0.61 (q, J = 7.93 Hz).

^{13}C NMR (101 MHz, $CDCl_3$) δ 83.62, 83.51, 25.35, 25.03, 7.52, 3.58, 3.32, 3.06. (C-B) Carbon signal not observed due to quadrupolar relaxation.[3]

^{11}B NMR (128 MHz, $CDCl_3$) δ: 31.5, 28.3.

HRMS (ESI) Calcd for $[C_{20}H_{40}B_2O_4Si+H]^+$ $[M+H]^+$: m/z 395.2963, found 395.2950.

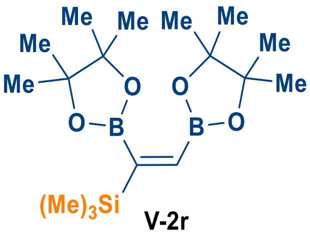

V-2r

(E)-(1,2-bis(4,4,5,5-tetramethyl-1,3,2-dioxaborolan-2-yl)vinyl)trimethylsilane (V-2r):

Prepared according to general procedure D, product **V-2r** isolated in (0.306 gr, 87% yield) as a yellow oil.

R_f = 0.56 (5% EtOAc in hexane).

^{1}H NMR (400 MHz, $CDCl_3$) δ: 6.59 (s, 1H), 1.33 (s, 12H), 1.26 (s, 12H), 0.10 (s, 9H).

^{13}C NMR (126 MHz, $CDCl_3$) δ: 83.71, 83.67, 25.33, 25.04, -1.30. (C-B) Carbon signal was not observed due to quadrupolar relaxation.[3]

^{11}B NMR (161 MHz, $CDCl_3$) δ: 31.7, 28.6.

The spectral data are consistent with those reported in the literature.[4]

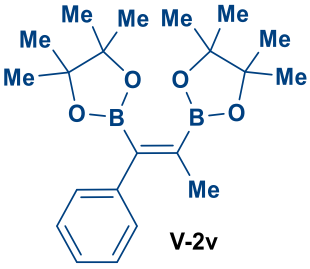

V-2v

(Z)-2,2'-(1-phenylprop-1-ene-1,2-diyl)bis(4,4,5,5-tetramethyl-1,3,2-dioxaborolane) (V-2v):

Prepared according to general procedure D, product **V-2v** was isolated in (0.288 gr, 78% yield) as a colorless liquid. R_f = 0.52 (10% EtOAc in hexane).

^{1}H NMR (400 MHz, CDCl3) δ: 7.32 - 7.26 (m, 2H), 7.20 - 7.18 (m, 1H), 7.16 - 7.12 (m, 2H), 1.72 (s, 3H), 1.33 (s, 12H), 1.26 (s, 12H).

The spectral data are consistent with those reported in the literature.[10]

General procedure E: The Synthesis of tri-substituted *gem*-diborylalkene [V-3(y - ab)]:

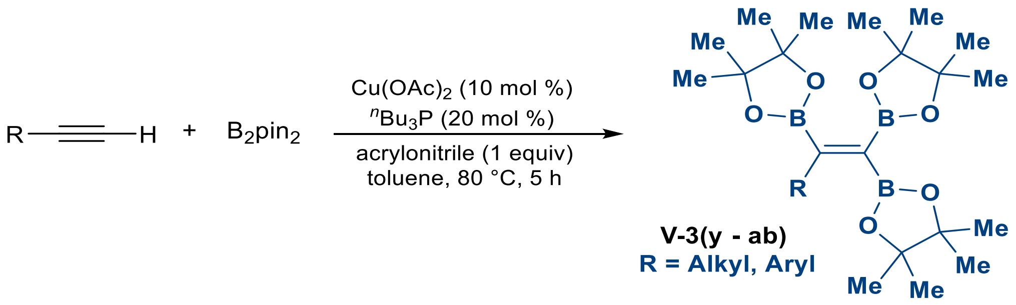

Products **[V-3(y - ab)]** were prepared according to a literature reported procedure.[3] Inside the glove box, to a 20 mL thick-walled reaction tube equipped with a magnetic stirring bar, $Cu(OAc)_2$ (10 mol %, 14.4 mg, 0.08 mmol), B_2pin_2 (3 equiv, 609.6 mg, 2.4 mmol) and toluene (4 mL) were added. Then, phenyl acetylene (81.6 mg, 88 µL, 0.8 mmol), acrylonitrile (42.4 mg, 52 µL, 0.8 mmol) and $^nBu_3P$ (32.4 mg, 39.6 µL, 0.16 mmol) were added in that order and the tube was sealed and taken out of the glove box. The reaction was heated at 80 °C for 5 h. The reaction mixture was then diluted with Et_2O (16 mL) and filtered through a plug of celite. The solvent was removed under reduced pressure (by evaporator), and the residue was purified by column chromatography on silica gel to give the desired product.

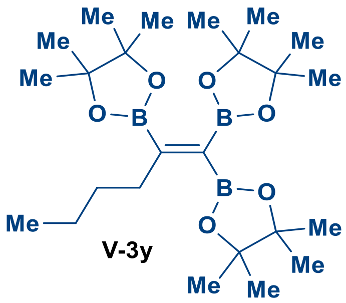

V-3y

2,2',2''-(hex-1-ene-1,1,2-triyl)tris(4,4,5,5-tetramethyl-1,3,2-dioxaborolane) (V-3y):

Prepared according to general procedure E, product **V-3y** was isolated in (0.232 gr, 55% yield) as a white solid.

R_f = 0.46 (10% EtOAc in hexane).

^{1}H NMR (400 MHz, $CDCl_3$) δ: 2.39 (t, *J* = 7 Hz, 2H), 1.40 - 1.33 (m, 4H), 1.30 (s, 12H), 1.27 (s, 12H), 1.26 (s, 12H), 0.88 (t, *J* = 7 Hz, 3H).

The spectral data are consistent with those reported in the literature.[3]

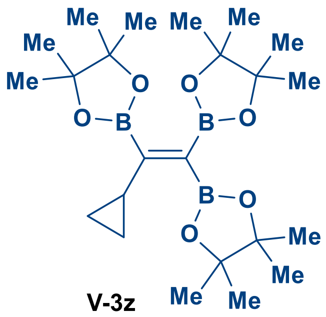

V-3z

2,2',2''-(2-cyclopropylethene-1,1,2-triyl)tris(4,4,5,5-tetramethyl-1,3,2-dioxaborolane) (V-3z):

Prepared according to general procedure E, product **V-3z** was isolated in (0.192 gr, 54% yield) as a white solid.

R_f = 0.33 (10% EtOAc in hexane).

^{1}H NMR (400 MHz, $CDCl_3$) δ: 1.97 - 1.90 (m, 1H), 1.24 (s, 36H), 0.78 - 0.66 (m, 4H).

The spectral data are consistent with those reported in the literature.[3]

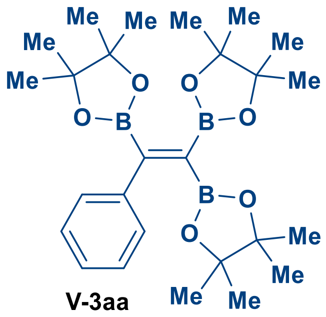

V-3aa

2,2',2''-(2-phenylethene-1,1,2-triyl)tris(4,4,5,5-tetramethyl-1,3,2-dioxaborolane) (V-3aa):

Prepared according to general procedure E, product **V-3aa** was isolated in (0.26 gr, 68% yield) as a white solid.

R_f = 0.30 (10% EtOAc in hexane).

^{1}H NMR (400 MHz, $CDCl_3$) δ: 7.22 - 7.08 (m, 5H), 1.23 (s, 12H), 1.20 (s, 12H), 1.01 (s, 12H).

The spectral data are consistent with those reported in the literature.[3]

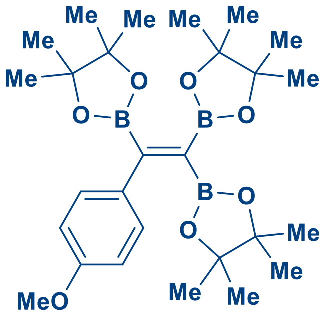

V-3ab

2,2',2''-(2-(4-methoxyphenyl)ethene-1,1,2-triyl)tris(4,4,5,5-tetramethyl-1,3,2-dioxaborolane) (V-3ab):

Prepared according to general procedure E, product **V-3ab** was isolated in (0.292 gr, 72% yield) as a white solid.

R_f = 0.35 (15% EtOAc in hexane).

^{1}H NMR (400 MHz, $CDCl_3$) δ: 7.24 (d, J = 9 Hz, 2H), 6.77 (d, J = 9 Hz, 2H), 3.77 (s, 3H), 1.30 (s, 12H), 1.27 (s, 12H), 1.11 (s, 12H).

The spectral data are consistent with those reported in the literature.[3]

General Procedure F: The Synthesis of (V-2x) and (V-4):

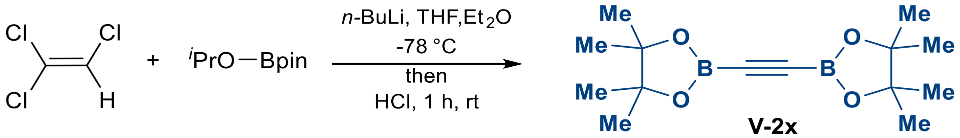

***1,2-bis(4,4,5,5-tetramethyl-1,3,2-dioxaborolan-2-yl)ethyne (V-2x)*:**

Products **(V-2x)** and **(V-4)** were prepared according to a literature reported procedure.[11] n-BuLi (2.5 M in hexane, 50 mmol) was added to a 250 ml Schlenk tube filled with a 70 mL 1:1 mixture of THF and Et_2O at -78 °C. Then 1.5 mL (16.6 mmol) of trichloroethylene in 20 mL of Et_2O was added dropwise. After that, the cooling bath was removed and the resulting pale yellow mixture was stirred overnight. Then, that the yellowish mixture was added to a precooled (-78 °C) solution of 2-isopropoxy-4,4,5,5-tetramethyl-1,3,2-dioxaborolane (6.6 mL, 32.6 mmol) in Et_2O (70 mL),

and stirred at -78 °C for 4 h, followed by another 2 h at room temperature. The reaction mixture was then cooled to 0 °C with the dropwise addition of 14 mL HCl (4.0 M in Et_2O, 53.3 mmol), and the reaction mixture was stirred for 30 min at room temperature. Then the mixture was filtered through a pad of celite, the solvent was then removed under reduced pressure (by evaporator) and the crude mixture was washed repeatedly with hexane to afford the title product as a brown solid.

^{1}H NMR (400 MHz, $CDCl_3$) δ: 1.25 (s, 24H).

^{11}B NMR (128 MHz, $CDCl_3$) δ: 23.3.

The spectral data are consistent with those reported in the literature.[11]

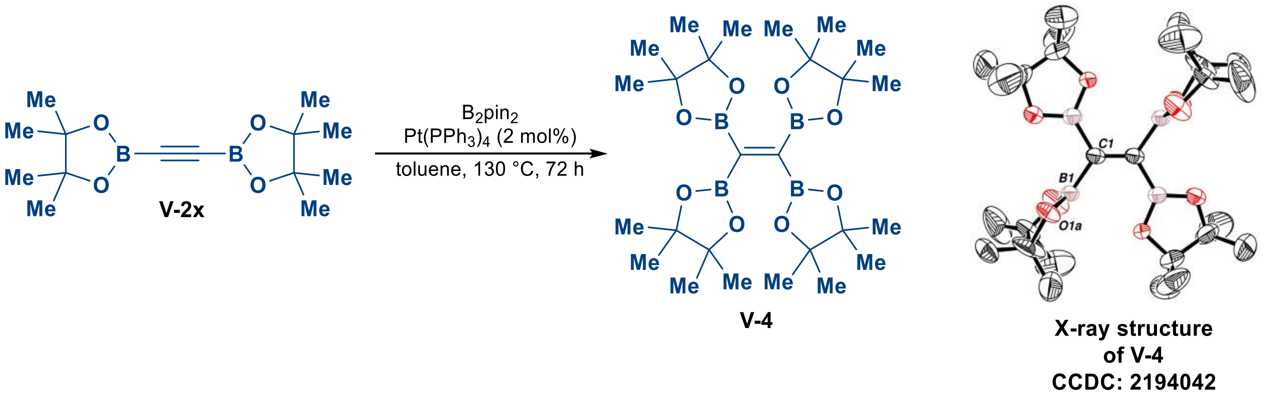

X-ray structure of V-4
CCDC: 2194042

***1,1,2,2-tetrakis(4,4,5,5-tetramethyl-1,3,2-dioxaborolan-2-yl) ethane (V-4)*:**

Product **(V-4)** was prepared according to a literature reported procedure with slight modification.[11] A dry nitrogen flushed 50 mL flask equipped with a magnetic stirring bar was charged with alkyne (1.0 mmol), $Pt(PPh_3)_4$ (25.0 mg, 0.03 mmol), bis(pinacolato)diboron (257.0 mg, 1 mmol) and 5 mL of toluene, and the mixture was stirred for 72 h at 130 °C. The reaction was cooled in ice bath for 5 min, and hexane was added dropwise to the crude reaction until white solid starts to precipitate. The solid was collected via filtration and washed thoroughly with hexane to finally afford the product in (0.389 gr, 70% yield) as a creamy colored solid.

^{1}H NMR (400 MHz, $CDCl_3$) δ: 1.24 (s, 48H).

The spectral data are consistent with those reported in the literature.[11]

Notes: (1) The structure of **V-4** was confirmed by X-ray crystallographic analysis, CCDC 2194042 (see Supplementary Table 10, page 97). (2) Compound **V-4** was recrystallized by using a solvent mixture of hexane:EtOAc (10:1).

2.2. General Procedure G and Characterization for Triflourination Products (1)

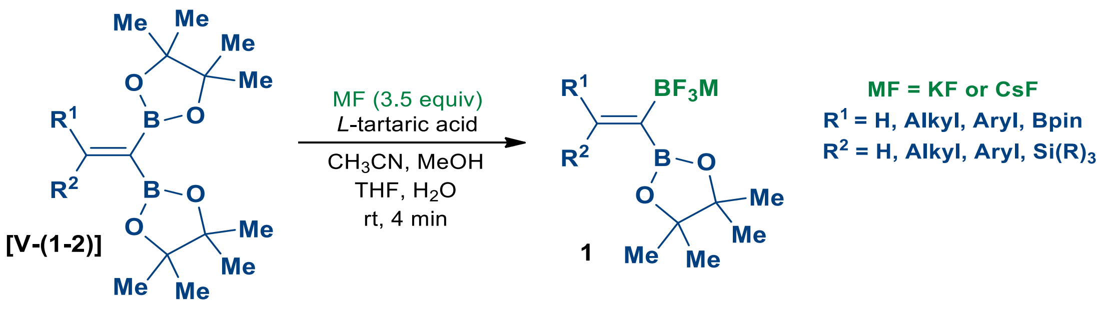

To initiates the reaction, a diboroalkene substrate [**V-(1-2)**] (0.10 mmol) was added to an air-open flask followed by the addition of a mixture of acetonitrile (0.5 mL) and methanol (0.5 mL). Following that, to this mixture, a solution of fluoride salt i.e. KF or CsF (0.35 mmol) in H_2O (0.2 mL) was added, and stirring was continued for 2 minutes at room temperature. Next, *L*-tartaric acid (0.20 mmol) in THF (0.7 mL) was gradually added to the mixture, which was being stirred vigorously. The stirring was continued for an additional 4 minutes, during which a white precipitate formed. The mixture was filtered to remove the white precipitate and thoroughly washed with excess acetonitrile (5 mL). The filtrate was then concentrated using a reduced-pressure evaporator, resulting in a residue of crude solid. The solid residue was washed with diethyl ether and hexane, giving rise to the corresponding organotrifluoroborate salt alkene (**1**) as an amorphous white solid. The solid was then dried further under high vacuum overnight. [12]

Supplementary Table 1. Optimization table for *gem*-organotrifluoroborate salts (**1**) preparation:

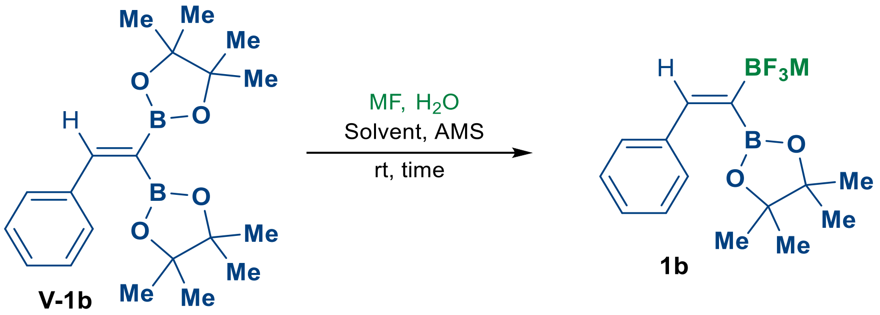

Entry	MF	Solvent 1	Solvent 2	Solvent 3	AMS	Time	Yield of (1b)[a,b]
1	KF	CH_3CN	MeOH	THF	*L*-tartaric acid	4 min	81 %
2	KF	CH_3CN	MeOH	THF	Citric acid	4 min	72 %
3	KF	CH_3CN	MeOH	THF	18-crown-6	4 min	78 %
4	KHF_2 (2 equiv)	-	MeOH	-	-	5 h	20 %
5	CsF	CH_3CN	MeOH	THF	*L*-tartaric acid	4 min	93 %
6	CsF	CH_3CN	-	THF	*L*-tartaric acid	4 min	10 %
7	CsF	Acetone	MeOH	THF	*L*-tartaric acid	4 min	20 %
8	LiF	CH_3CN	MeOH	THF	*L*-tartaric acid	4 min	5 %
9	TBAF	CH_3CN	MeOH	THF	-	4 min	FC

[a] Reactions were carried out with 0.20 mmol of **V-1b** and 0.7 mmol of MF along with 1 ml of Solvent-1, 1 ml of Solvent-2, 0.7 ml of Solvent-3 and 0.41 mmol of AMS (Alkali Metal Sponge) in an open flask at rt for the indicated amount of time. [b] Isolated yield. [c] full conversion (FC) by ^{1}H-NMR.

Supplementary Table 2. Scope of the *gem*-organotrifluoroborate salts (**1**):

Entry	Substrate	MF	Yield (%)[a]	Isomeric ratio[b]	Product
**1**	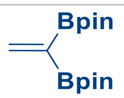 V-1a	CsF	93	-	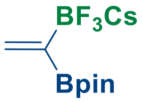 1a
**2**	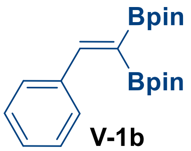 V-1b	CsF	91	*E*:*Z* = 99:01	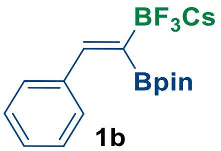 1b
**3**	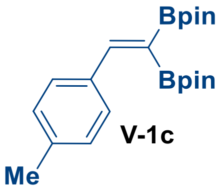 V-1c	CsF	88	*E*:*Z* = 99:01	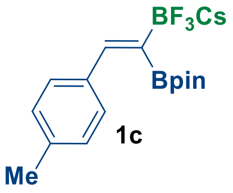 1c
**4**	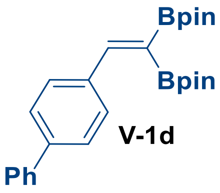 V-1d	CsF	83	*E*:*Z* = 99:01	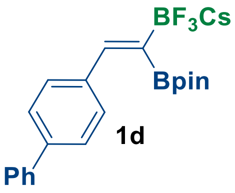 1d
**5**	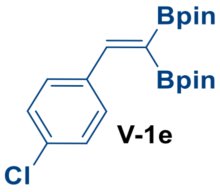 V-1e	CsF	46	*E*:*Z* = 99:01	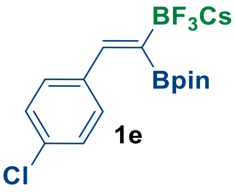 1e
**6**	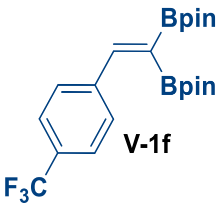 V-1f	CsF	89	*E*:*Z* = 99:01	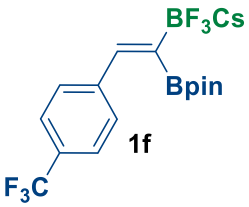 1f
**7**	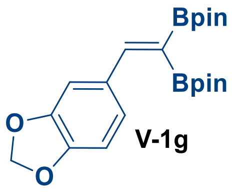 V-1g	CsF	84	*E*:*Z* = 99:01	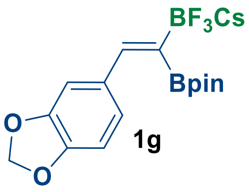 1g

**8**	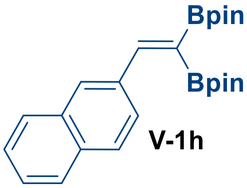<br>**V-1h**	CsF	80	*E*:*Z* = 99:01	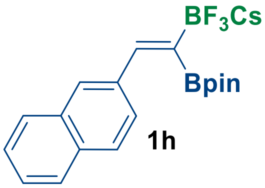<br>**1h**
**9**	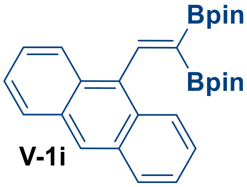<br>**V-1i**	CsF	71	*E*:*Z* = 99:01	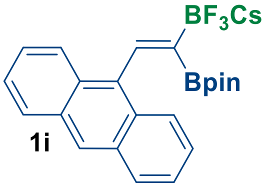<br>**1i**
**10**	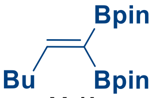<br>**V-1j**	KF	91	*E*:*Z* = 97: 03	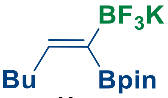<br>**1j**
**11**	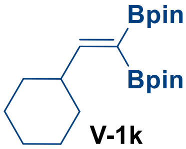<br>**V-1k**	KF	89	*E*:*Z* = 97: 03	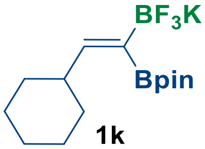<br>**1k**
**12**	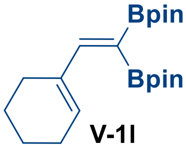<br>**V-1l**	KF	79	*E*:*Z* = 99:01	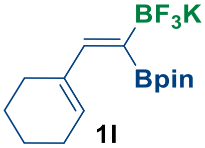<br>**1l**
**13**	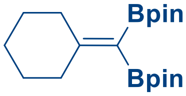<br>**V-1m**	CsF	88	-	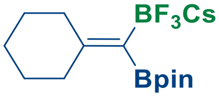<br>**1m**
**14**	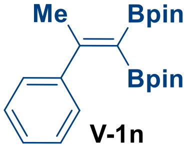<br>**V-1n**	CsF	40	*E*:*Z* = 86:14	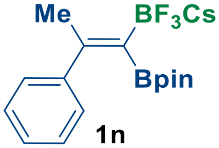<br>**1n**
**15**	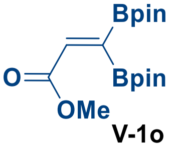<br>**V-1o**	CsF	86	*E*:*Z* = 99:01	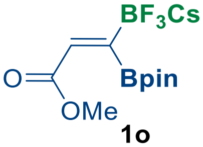<br>**1o**
**16**	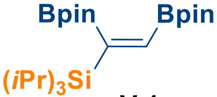<br>**V-1p**	CsF	89	*E*:*Z* = 99:01<br>*s.s.* = 99:01	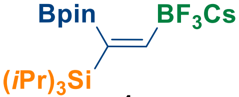<br>**1p**

17	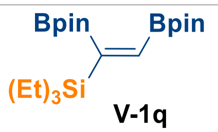 V-1q	CsF	92	*E*:*Z* = 99:01 *s.s.* = 99:01	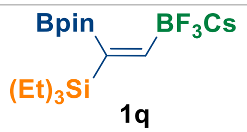 1q
18	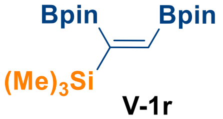 V-1r	CsF	95	*E*:*Z* = 99:01 *s.s.* = 99:01	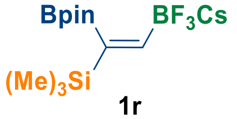 1r
19	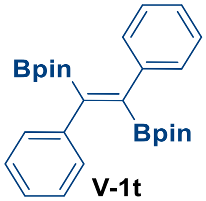 V-1t	CsF	33	*E*:*Z* = 99:01	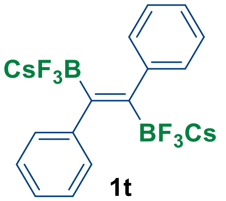 1t

[a] Isolated yield. [b] *E:Z.* = *isomeric ratio*. *s.s.* = site selectivity ratio.

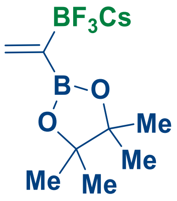
1a

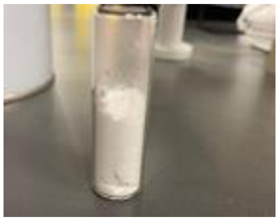

Picture of product **1a** from a gram scale reaction

2-(ethene-1,1-diyl)bis(4,4,5,5-tetramethyl-1,3,2-dioxaborolane) cesium salt **(1a):**

Prepared according to general procedure G, product **1a** was isolated in (42 mg, 93% yield) as a white solid. A 2-gram scale reaction gave a (0.9 gr, 63% yield).

^{1}H NMR (400 MHZ, DMSO-*d*$_6$) δ: 5.73 (s, 2H), 1.14 (s, 12H).

^{13}C NMR (101 MHz, DMSO-*d*$_6$) δ: 132.51, 132.47, 81.24, 24.71. (C-B) Carbon signal was not observed due to quadrupolar relaxation.[3]

^{11}B NMR (128 MHz, DMSO-*d*$_6$) δ: 32.0, 1.4.

^{19}F NMR (376 MHz, DMSO-*d*$_6$) δ: -136.0 (s).

HRMS (ESI) Calcd for $[C_8H_{14}B_2O_2F_3]^-$ $[M]^-$: m/z 221.11, found 221.11.

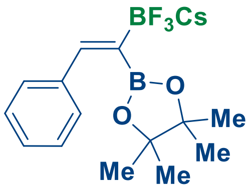

1b

(E)-4,4,5,5-tetramethyl-2-(2-phenyl-1-(trifluoro-l4-boraneyl)vinyl)-1,3,2-dioxaborolane cesium salt (1b):

Prepared according to general procedure G, product **1b** was isolated in (52 mg, 91% yield), *E:Z = 99:01*, as a white solid.

^{1}**H NMR** (500 MHZ, DMSO-d_6) δ: 7.25 (d, J = 7.58 Hz, 2H), 7.21 (t, J = 7.49 Hz, 2H), 7.10 (d, J = 7.15 Hz, 1H), 6.79 (s, 1H), 1.17 (s, 12H).

^{13}**C NMR** (101 MHz, DMSO-d_6) δ: 142.32, 138.41, 138.36, 127.74, 126.75, 125.64, 81.63, 24.69. (C-B) Carbon signal was not observed due to quadrupolar relaxation.[3]

^{11}**B NMR** (128 MHz, DMSO-d_6) δ: 35.2, 1.2.

^{19}**F NMR** (376 MHz, DMSO-d_6) δ: -136.1 (s).

HRMS (ESI) Calcd for $[C_{14}H_{18}B_2O_2F_3]^-$ $[M]^-$: m/z 297.1456, found 297.1455.

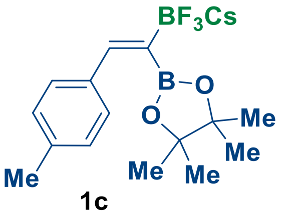

1c

(E)-4,4,5,5-tetramethyl-2-(2-(p-tolyl)-1-(trifluoro-l4-boraneyl)vinyl)-1,3,2-dioxaborolane, cesium salt (1c):

Prepared according to general procedure G, product **1c** was isolated in (54 mg, 88% yield), *E:Z = 99:01*, as a white solid.

^{1}**H NMR** (400 MHZ, DMSO-d_6) δ: 7.17 (d, J = 7.71 Hz, 2H), 7.02 (d, J = 7.56 Hz, 2H), 6.73 (s, 1H), 2.25 (s, 3H), 1.18 (s, 12H).

^{13}**C NMR** (101 MHz, DMSO-d_6) δ: 139.50, 138.52, 138.48, 134.68, 128.84, 128.36, 128.04, 127.87, 126.75, 81.66, 24.71, 20.69. (C-B) Carbon signal was not observed due to quadrupolar relaxation.[3]

^{11}B NMR (128 MHz, DMSO-*d_6*) δ: 35.0, 2.4.

^{19}F NMR (376 MHz, DMSO-*d_6*) δ: -136.2 (s).

HRMS (ESI) Calcd for $[C_{15}H_{20}B_2O_2F_3]^-$ $[M]^-$: m/z 311.1312, found 311.1613.

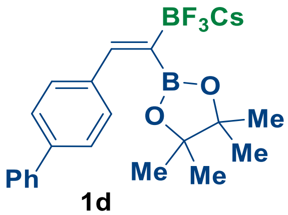

1d

(E)-2-(2-([1,1'-biphenyl]-4-yl)-1-(trifluoro-l4-boraneyl)vinyl)-4,4,5,5-tetramethyl-1,3,2-dioxaborolane cesium salt (1d):

Prepared according to general procedure G, product **1d** was isolated in (82 mg, 83% yield), *E:Z = 99:01*, as a white solid.

^{1}H NMR (400 MHZ, DMSO-*d_6*) δ: 7.65 (d, *J* = 8.45 Hz, 2H), 7.53 (d, *J* = 8.25 Hz, 2H), 7.44 (t, *J* = 7.88 Hz, 2H), 7.38-7.30 (m, 3H), 6.82 (s, 1H), 1.20 (s, 12H).

^{13}C NMR (101 MHz, DMSO-*d_6*) δ: 141.53, 140.08, 137.76, 137.72, 137.31, 128.85, 127.33, 127.03, 126.34, 126.05, 81.68, 24.73. (C-B) Carbon signal was not observed due to quadrupolar relaxation.[3]

^{11}B NMR (128 MHz, DMSO-*d_6*) δ: 33.8, 1.6.

^{19}F NMR (376 MHz, DMSO-*d_6*) δ: -136.0.

Note: HRMS was not accurate due to decomposition and solubility issues.

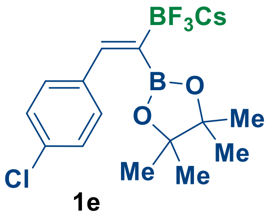

1e

(E)-2-(2-(4-chlorophenyl)-1-(trifluoro-l4-boraneyl)vinyl)-4,4,5,5-tetramethyl-1,3,2-dioxaborolane cesium salt (1e):

Prepared according to general procedure G, product **1e** was isolated in (30 mg, 46% yield), *E:Z = 99:01*, as a white solid.

^{1}H NMR (400 MHZ, DMSO-d_6) δ: 7.51 - 7.49 (m, 2H), 7.10 - 7.07 (m, 2H), 6.75 (s, 1H), 1.17 (s, 12H).

^{13}C NMR (101 MHz, DMSO-d_6) δ: 141.26, 136.89, 130.34, 129.98, 128.39, 127.73, 126.42, 81.75, 24.96, 24.69. (C-B) Carbon signal was not observed due to quadrupolar relaxation.[3]

^{11}B NMR (128 MHz, DMSO-d_6) δ: 33.9, 3.0.

^{19}F NMR (376 MHz, DMSO-d_6) δ: -136.0 (s).

HRMS (ESI) Calcd for $[C_{14}H_{17}B_2O_2F_3Cl]^+$ $[M]^+$: m/z 331.1061, found 331.1030.

Note: Some unexplained impurity is shown in the NMR reprints.

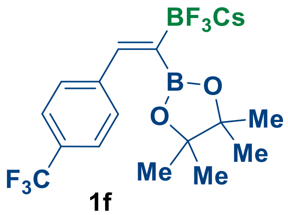

1f

(E)-4,4,5,5-tetramethyl-2-(1-(trifluoro-l4-boraneyl)-2-(4-(trifluoromethyl)phenyl)vinyl)-1,3,2-dioxaborolane, cesium salt (1f):

Prepared according to general procedure G, product **1f** was isolated in (64 mg, 89% yield), *E:Z = 99:01*, as a white solid.

^{1}H NMR (400 MHZ, DMSO-d_6) δ: 7.57 (d, J = 8.40 Hz, 2H), 7.45 (d, J = 8.22 Hz, 2H), 6.85 (s, 1H), 1.18 (s, 12H).

^{13}C NMR (101 MHz, DMSO-d_6) δ: 146.29, 136.81, 136.76, 127.16, 124.74, 124.70, 81.88, 24.66. (C-B) Carbon signal was not observed due to quadrupolar relaxation.[3]

^{11}B NMR (128 MHz, DMSO-d_6) δ: 33.7, 1.5.

^{19}F NMR (376 MHz, DMSO-d_6) δ: -60.5 (s, 3F), -136.5 (s, 3F).

HRMS (ESI) Calcd for $[C_{15}H_{17}B_2O_2F_6]^+$ $[M]^+$: m/z 365.1324, found 365.1323.

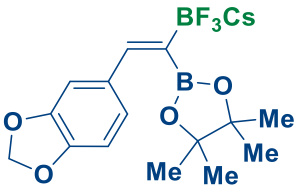

1g

(E)-2-(2-(benzo[1,3]dioxol-5-yl)-1-(trifluoro-l4-boraneyl)vinyl)-4,4,5,5-tetramethyl-1,3,2-dioxaborolane, cesium salt (1g):

Prepared according to general procedure G, product **1g** was isolated in (80 mg, 84% yield), *E:Z = 99:01*, as a white solid.

^{1}H NMR (400 MHZ, DMSO-d_6) δ: 6.90 (d, *J* = 1.63 Hz, 1H), 6.76 (d, *J* = 7.62 Hz, 1H), 6.70 (dd, *J* = 8.03, 1.64 Hz, 2H), 5.94 (s, 2H), 1.17 (s, 12H).

^{13}C NMR (101 MHz, DMSO-d_6) δ: 147.09, 145.26, 137.94, 137.91, 137.15, 120.59, 107.73, 106.42, 100.58, 81.61, 24.71. (C-B) Carbon signal was not observed due to quadrupolar relaxation.[3]

^{11}B NMR (128 MHz, DMSO-d_6) δ: 33.7, 1.5.

^{19}F NMR (376 MHz, DMSO-d_6) δ: -135.6 (s).

Notes: (1) The relative configuration of **1g** was determined by 2D-NMR NOESY (see Supplementary Figures 289-290, page 242). (2) HRMS was not accurate due to decomposition and solubility issues.

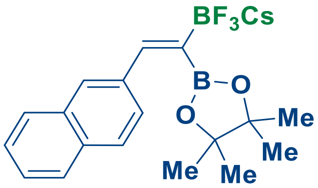

1h

(E)-4,4,5,5-tetramethyl-2-(2-(naphthalen-2-yl)-1-(trifluoro-l4-boraneyl)vinyl)-1,3,2-dioxaborolane, cesium salt (1h):

Prepared according to general procedure G, product **1h** was isolated in (55.4 mg, 80% yield), *E:Z = 99:01*, as a white solid.

^{1}H NMR (400 MHZ, DMSO-d_6) δ: 7.84 - 7.73 (m, 4H), 7.52 - 7.38 (m, 3H), 6.96 (s, 1H), 1.21 (s, 12H).

^{13}**C NMR** (101 MHz, DMSO-d_6) δ: 140.10, 138.23, 138.18, 133.21, 131.68, 127.42, 127.35, 127.00, 126.10, 125.91, 124.93, 124.46, 81.72, 24.75. (C-B) Carbon signal was not observed due to quadrupolar relaxation.[3]

^{11}**B NMR** (128 MHz, DMSO-d_6) δ: 32.7, 1.8.

^{19}**F NMR** (376 MHz, DMSO-d_6) δ: -136.0 (s).

HRMS (ESI) Calcd for $[C_{18}H_{20}B_2O_2F_3]^+$ $[M]^+$: m/z 347.1608, found 347.1611.

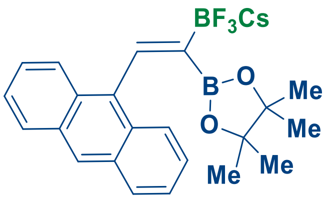

1i

(E)-2-(2-(anthracen-9-yl)-1-(trifluoro-l4-boraneyl)vinyl)-4,4,5,5-tetramethyl-1,3,2-dioxaborolane, cesium salt (1i):

Prepared according to general procedure G, product **1i** was isolated in (56.8 mg, 71% yield), *E:Z = 99:01*, as a white solid.

^{1}**H NMR** (400 MHZ, DMSO-d_6) δ: 8.34 (s, 1H), 8.24 (dd, J = 8.73, 1.29 Hz, 2H), 7.98 (dd, J = 8.52, 1.12 Hz, 2H), 7.53 (brs, 1H), 7.46 - 7.37 (m, 4H), 0.49 (s, 12H).

^{13}**C NMR** (101 MHz, DMSO-d_6) δ: 139.22, 135.47, 130.98, 128.75, 127.93, 127.44, 124.87, 123.96, 123.74, 80.83, 23.99. (C-B) Carbon signal was not observed due to quadrupolar relaxation.[3]

^{11}**B NMR** (128 MHz, DMSO-d_6) δ: 34.7, 2.5.

^{19}**F NMR** (376 MHz, DMSO-d_6) δ: -136.2 (s).

HRMS (ESI) Calcd for $[C_{22}H_{26}B_2O_2F_3]^-$ $[M]^-$: m/z 400.2006, found 400.1988

Note: The relative configuration of **1i** was determined by 2D-NMR NOESY (see Supplementary Figures 291-292, page 243).

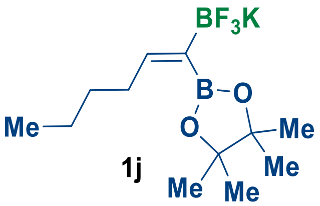

1j

(E)-4,4,5,5-tetramethyl-2-(1-(trifluoro-l4-boraneyl)hex-1-en-1-yl)-1,3,2-dioxaborolane, potassium salt (1j):

Prepared according to general procedure G, product **1j** was isolated in (50.4 mg, 91% yield), *E:Z = 97:03*, as a white solid.

^{1}H NMR (400 MHZ, DMSO-d_6) δ: 5.90 (t, J = 7.78 Hz, 1H), 1.97 (d, J = 6.35, 2H), 1.25 - 1.22 (m, 4H), 1.13 (s, 12H), 0.84 (t, J = 7.06, 3H).

^{13}C NMR (101 MHz, DMSO-d_6) δ: 142.94, 142.89, 81.03, 33.80, 31.92, 24.69, 21.84, 13.88 (C-B) Carbon signal was not observed due to quadrupolar relaxation.[3]

^{11}B NMR (128 MHz, DMSO-d_6) δ: 33.9, 3.0.

^{19}F NMR (376 MHz, DMSO-d_6) δ: -136.0 (s).

HRMS (ESI) Calcd for $[C_{12}H_{22}B_2O_2F_3]^+$ $[M]^+$: m/z 277.1762, found 277.1762.

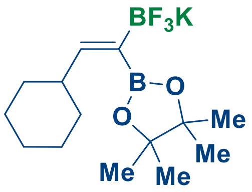

1k

(E)-2-(2-cyclohexyl-1-(trifluoro-l4-boraneyl)vinyl)-4,4,5,5-tetramethyl-1,3,2-dioxaborolane, potassium salt (1k):

Prepared according to general procedure G, product **1k** was isolated in (53.8 mg, 89 % yield), *E:Z = 97:03*, as a white solid.

^{1}H NMR (400 MHZ, DMSO-d_6) δ: 5.70 (d, J = 8.35 Hz, 1H), 2.03 - 2.00 (m, 1H), 1.64 (d, J = 9.56 Hz, 2H), 1.55 (d, J = 12.96 Hz, 3H), 1.25 (s, 1H), 1.15 (brs, 14H), 0.94 (q, J = 12.15 Hz, 2H).

^{13}C NMR (101 MHz, DMSO-d_6) δ: 148.11, 148.07, 81.00, 42.65, 33.43, 25.84, 25.74, 24.66. (C-B) Carbon signal was not observed due to quadrupolar relaxation.[3]

^{11}B NMR (128 MHz, DMSO-d_6) δ: 33.9, 3.1.

^{19}F NMR (376 MHz, DMSO-d_6) δ: -136.0 (s).

HRMS (ESI) Calcd for $[C_{14}H_{24}B_2O_2F_3]^+$ $[M]^+$: m/z 303.1920, found 303.1915.

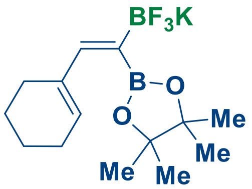

1l

(E)-2-(2-(cyclohex-1-en-1-yl)-1-(trifluoro-l4-boraneyl)vinyl)-4,4,5,5-tetramethyl-1,3,2-dioxaborolane, potassium salt (1l):

Prepared according to general procedure G, product **1l** was isolated in (53.6 mg, 79% yield), *E:Z = 99:01*, as a white solid.

^{1}H NMR (400 MHZ, DMSO-d_6) δ: 6.28 (s, 1H), 5.47 (s, 1H), 2.05 (brs, 4H), 1.53 - 1.47 (m, 4H), 1.14 (s, 12H).

^{13}C NMR (101 MHz, DMSO-d_6) δ: 141.77, 139.82, 124.51, 81.33, 26.09, 25.26, 24.71, 22.28, 22.25. (C-B) Carbon signal was not observed due to quadrupolar relaxation.[3]

^{11}B NMR (128 MHz, DMSO-d_6) δ: 32.4, -0.4.

^{19}F NMR (376 MHz, DMSO-d_6) δ: -135.7 (s).

Notes: (1) The relative configuration of **1l** was determined by 2D-NMR NOESY (see Supplementary Figure 293, page 244).

(2) HRMS was not accurate due to decomposition and solubility issues.

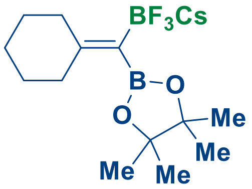

1m

2-(cyclohexylidene(trifluoro-l4-boraneyl)methyl)-4,4,5,5-tetramethyl-1,3,2-dioxaborolane, cesium salt (1m):

Prepared according to general procedure G, product **1m** was isolated in (50.8 mg, 88% yield), as a white solid.

^{1}H NMR (400 MHZ, DMSO-d_6) δ: 2.15 (brs, 2H), 1.94 (brs, 2H), 1.40 (brs, 6H), 1.12 (s, 12H).

^{13}C NMR (101 MHz, DMSO-d_6) δ: 149.37, 80.91, 34.22, 28.59, 28.18, 26.74, 24.73. (C-B) Carbon signal was not observed due to quadrupolar relaxation.[3]

^{11}B NMR (128 MHz, DMSO-d_6) δ: 34.6, 2.9.

^{19}F NMR (376 MHz, DMSO-d_6) δ: -136.0 (s).

HRMS (ESI) Calcd for $[C_{13}H_{22}B_2O_2F_3]^+$ $[M]^+$: m/z 289.1757, found 289.1754.

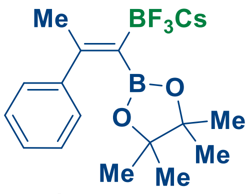

1n

***(E)-4,4,5,5-tetramethyl-2-(2-phenyl-1-(trifluoro-l4-boraneyl)prop-1-en-1-yl)-1,3,2-dioxaborolane, cesium salt (1n)*:**

Prepared according to general procedure G, product **1n** was isolated in (35.6 mg, 40% yield), *E:Z = 86:14*, as a white solid.

^{1}H NMR (400 MHZ, DMSO-d_6) δ: 7.45 - 7.07 (m, 5H + 2 H for **both diastereomers**), 2.01 (s, 3H for **major diastereomer**), 1.98 (s, 3H for **minor diastereomer**), 1.20 (s, 12H for **minor diastereomer**), 0.97 (s, 12 H for **major diastereomer**).

^{13}C NMR (101 MHz, DMSO-d_6, for **both diastereomer**) δ: 128.25, 128.23, 127.74, 127.20, 127.03, 125.70, 125.54, 81.60, 25.08, 23.48 (C-B) Carbon signal was not observed due to quadrupolar relaxation.[3]

^{11}B NMR (128 MHz, DMSO-d_6, for **both diastereomer**) δ: 34.6, 2.5.

^{19}F NMR (376 MHz, DMSO-d_6, for **both diastereomer**) δ: -137.3 (s).

Notes: (1) The relative configuration of **1n** was determined by 2D-NMR NOESY (see Supplementary Figure 294, page 244).

(2) HRMS was not accurate due to decomposition and solubility issues.

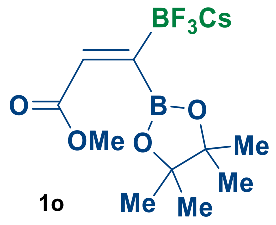

1o

Methyl-(E)-3-(4,4,5,5-tetramethyl-1,3,2-dioxaborolan-2-yl)-3-(trifluoro-l4-boraneyl)acrylate, cesium salt (1o):

Prepared according to general procedure G, product **1o** was isolated in (51.8 mg, 86% yield), *E:Z = 99:01*, as a white solid.

^{1}H NMR (400 MHZ, DMSO-d_6) δ: 6.02 (s, 1H), 3.57 (s, 3H), 1.19 (s, 12H).

^{13}C NMR (101 MHz, DMSO-d_6) δ: 167.67, 127.14, 81.86, 50.61, 24.66. (C-B) Carbon signal was not observed due to quadrupolar relaxation.[3]

^{11}B NMR (128 MHz, DMSO-d_6) δ: 34.5, 2.3.

^{19}F NMR (376 MHz, DMSO-d_6) δ: -137.8 (s).

HRMS (ESI) Calcd for $[C_{10}H_{16}B_2O_4F_3]^+$ $[M]^+$: m/z 302.1083, found 302.1086.

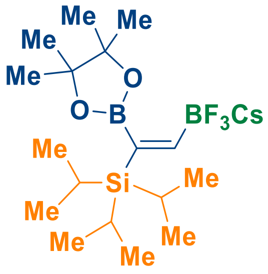

1p

(E)-triisopropyl(1-(4,4,5,5-tetramethyl-1,3,2-dioxaborolan-2-yl)-2-(trifluoro-l4-boraneyl)vinyl)silane, cesium salt (1p):

Prepared according to general procedure G, product **1p** was isolated in (67.2 mg, 89% yield), *E:Z = 99:01*, as a white solid.

^{1}H NMR (400 MHZ, DMSO-d_6) δ: 6.57 (q, *J* = 4.30 Hz, 1H), 1.16 (s, 12H), 1.08 - 0.96 (m, 21H).

^{13}C NMR (101 MHz, DMSO-d_6) δ: 81.45, 25.17, 18.74, 11.13. (C-B) Carbon signal was not observed due to quadrupolar relaxation.[3]

^{11}B NMR (128 MHz, DMSO-d_6) δ: 35.8, 2.5.

^{19}F NMR (376 MHz, DMSO-d_6) δ: -136.1 (s).

HRMS (ESI) Calcd for $[C_{17}H_{34}B_2O_2F_3Si]^-$ $[M]^-$: m/z 377.2479, found 377.2481.

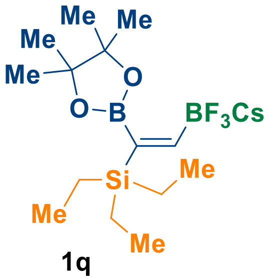

1q

(E)-triethyl(1-(4,4,5,5-tetramethyl-1,3,2-dioxaborolan-2-yl)-2-(trifluoro-l4-boraneyl)vinyl)silane, cesium salt (1q):

Prepared according to general procedure G, product **1q** was isolated in (61.6 mg, 92% yield), *E:Z = 99:01*, as a white solid.

^{1}H NMR (400 MHZ, DMSO-d_6) δ: 6.55 (q, J = 4.12 Hz, 1H), 1.17 (s, 12H), 0.87 (t, J = 7.87 Hz, 9H), 0.46 (q, J= 7.90 Hz, 6H).

^{13}C NMR (101 MHz, DMSO-d_6) δ: 81.46, 25.01, 7.51, 3.40. (C-B) Carbon signal was not observed due to quadrupolar relaxation.[3]

^{11}B NMR (128 MHz, DMSO-d_6) δ: 34.4, 2.2.

^{19}F NMR (376 MHz, DMSO-d_6) δ: -137.1 (s).

HRMS (ESI) Calcd for $[C_{14}H_{28}B_2O_2F_3Si]^+$ $[M]^-$: m/z 335.2008, found 335.2004.

1r

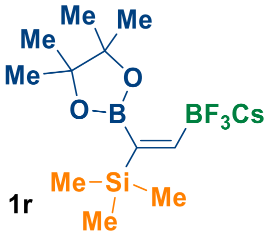

(E)-trimethyl(1-(4,4,5,5-tetramethyl-1,3,2-dioxaborolan-2-yl)-2-(trifluoro-l4-boraneyl)vinyl)silane, cesium salt (1r):

Prepared according to general procedure G, product **1r** was isolated in (55.6 mg, 95% yield), *E:Z = 99:01*, as a white solid.

^{1}H NMR (400 MHZ, DMSO-d_6) δ: 6.52 (q, J = 4.13 Hz, 1H), 1.16 (s, 12H), -0.04 (s, 9H).

^{13}C NMR (101 MHz, DMSO-d_6) δ: 81.47, 24.95, -0.49. (C-B) Carbon signal was not observed due to quadrupolar relaxation.[3]

^{11}B NMR (128 MHz, DMSO-d_6) δ: 34.7, 2.5.

^{19}F NMR (376 MHz, DMSO-d_6) δ: -136.1 (s).

HRMS (ESI) Calcd for $[C_{11}H_{22}B_2O_2F_3Si]^+$ $[M]^+$: m/z 293.1538, found 293.1539.

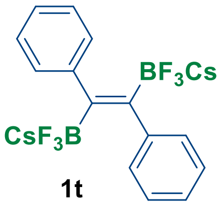

1t

(E)-1,2-diphenyl-1,2-bis(trifluoro-l4-boraneyl)ethene, dicesium salt (1t):

Prepared according to general procedure G, product **1t** was isolated in (38.2 mg, 33% yield), *E:Z = 99:01*, as a white solid.

^{1}H NMR (400 MHZ, DMSO-d_6) δ: 7.09 - 6.92 (m, 10H).

^{13}C NMR (101 MHz, DMSO-d_6) δ: 128.42, 128.35, 128.15, 127.85, 127.81, 127.30, 127.15, 127.08, 126.95, 123.77. (C-B) Carbon signal was not observed due to quadrupolar relaxation.[3]

^{11}B NMR (128 MHz, DMSO-d_6) δ: 6.25, 4.70.

^{19}F NMR (376 MHz, DMSO-d_6) δ: -143.3 (s).

2.3. General Procedure H and Characterization for *gem*-(Bpin,Bdan)-alkene (2)

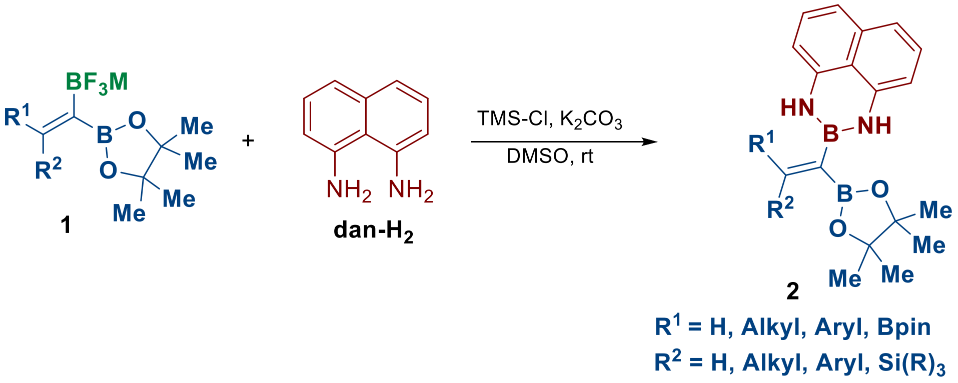

An oven-dried 10 ml Schlenk tube was cooled under N_2 before the addition of potassium carbonate (3 equiv, 0.3 mmol) and trifluoroborate salt (**1**) (0.1 mmol). DMSO (1 ml) was then added to the reaction mixture along with 1,8-diaminonaphthalene (1.5 equiv, 0.15 mmol), and following the dropwise addition of trimethylsilyl chloride TMS-Cl (3 equiv, 0.3 mmol), then the mixture was stirred for 2 hours at room temperature. After completion of the reaction, the mixture was diluted with EtOAc (3 ml) and H_2O (3 ml), and the residue was extracted with EtOAc (3 × 5 mL). The organic layers were then washed with brine, dried over $MgSO_4$, and evaporated by an evaporator under reduced pressure to obtain a crude material. The crude material was further purified by a short column on silica gel, resulting in the formation of a yellow solid, which was identified as product (**2**).

Supplementary Table 3. Scope of the *gem*-(Bpin,Bdan)-alkene (**2**) products:

Entry	Substrate	Yield (%)[a]	Isomeric ratio[b]	Product
**1**	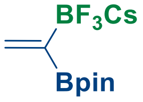 **1a**	73	-	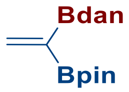 **2a**
**2**	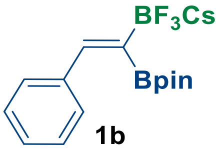 **1b**	70	*E*:*Z* = 01:99	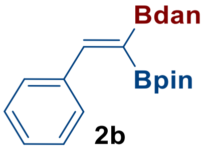 **2b**

**3**	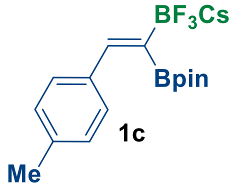 **1c**	74	E:Z = 01:99	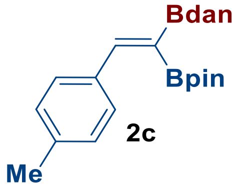 **2c**
**4**	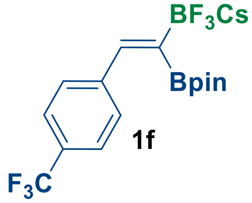 **1f**	75	E:Z = 01:99	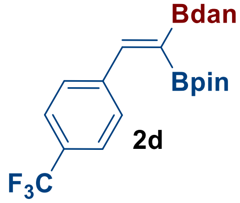 **2d**
**5**	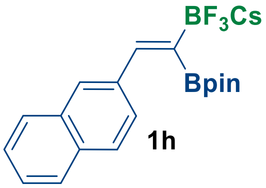 **1h**	58	E:Z = 01:99	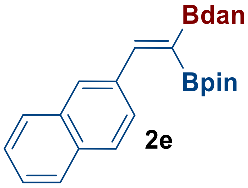 **2e**
**6**	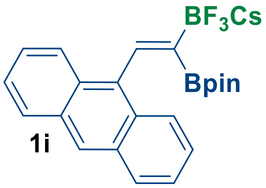 **1i**	65	E:Z = 01:99	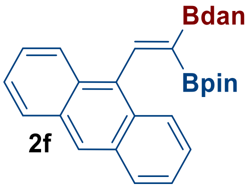 **2f**
**7**	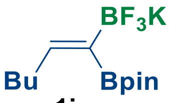 **1j**	68	E:Z = 03:97	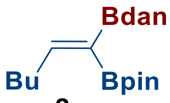 **2g**
**8**	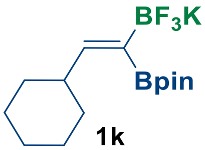 **1k**	63	E:Z = 03:97	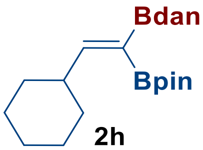 **2h**
**9**	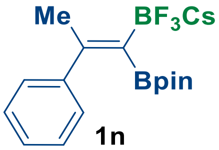 **1n**	62	E:Z = 10:90	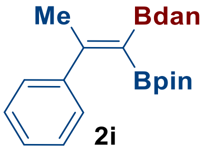 **2i**
**10**	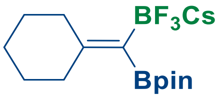 **1m**	54	-	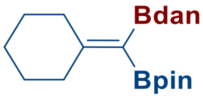 **2j**
**11**	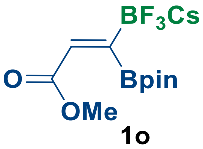 **1o**	77	E:Z = 01:99	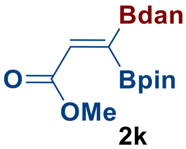 **2k**

**12**	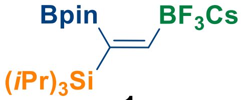 **1p**	80	*E*:*Z* = 99:01	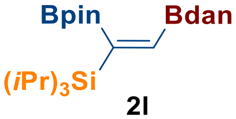 **2l**
**13**	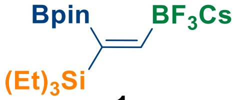 **1q**	76	*E*:*Z* = 99:01	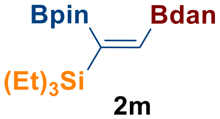 **2m**
**14**	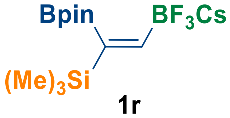 **1r**	73	*E*:*Z* = 99:01	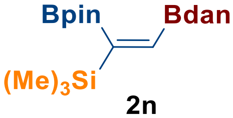 **2n**

[a] Isolated yield. [b] *E*:*Z* = *isomeric ratio*.

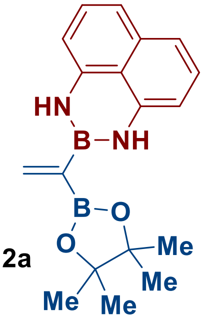

2a

2-(1-(4,4,5,5-tetramethyl-1,3,2-dioxaborolan-2-yl)vinyl)-2,3-dihydro-1H-naphtho[1,8-de][1,3,2]diazaborinine (2a):

Prepared according to general procedure H, product **2a** was isolated in (23.4 mg, 73% yield), as a yellow solid.

R_f = 0.33 (10% EtOAc in hexane).

^{1}H NMR (400 MHZ, $CDCl_3$) δ: 7.09 (t, *J* = 7.30 Hz, 2H), 6.99 (dd, *J* = 8.36, 0.91 Hz, 2H), 6.65 (d, *J* = 4.59 Hz, 1H), 6.49 (d, *J* = 4.44 Hz, 1H), 6.34 (dd, *J* = 7.35, 0.98 Hz, 2H), 6.30 (brs, 2H), 1.33 (s, 12H).

^{13}C NMR (101 MHz, $CDCl_3$) δ: 144.00, 141.63, 136.55, 127.68, 120.10, 117.38, 105.85, 83.66, 25.06. (C-B) Carbon signal was not observed due to quadrupolar relaxation.[3]

^{11}B NMR (128 MHz, $CDCl_3$) δ: 30.8, 28.2.

HRMS (ESI) Calcd for $[C_{19}H_{23}B_2O_2N_2+H]^+$ $[M+H]^+$: m/z 321.1947, found 321.1948.

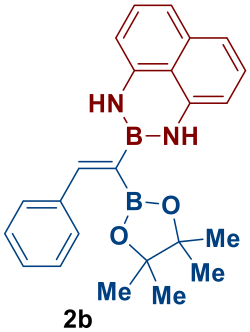

2b

(Z)-2-(2-phenyl-1-(4,4,5,5-tetramethyl-1,3,2-dioxaborolan-2-yl)vinyl)-2,3-dihydro-1H-naphtho[1,8-de][1,3,2]diazaborinine (2b):

Prepared according to general procedure H, product **2b** was isolated in (27.8 mg, 70% yield), *E:Z = 01:99*, as a yellow solid.

R_f = 0.50 (10% EtOAc in hexane).

^{1}H NMR (400 MHZ, $CDCl_3$) δ: 7.60 (s, 1H), 7.52 (dd, *J* = 7.77, 1.66 Hz, 2H), 7.40 - 7.33 (m, 3H), 7.14 (t, *J* = 7.38 Hz, 2H), 7.07 (d, *J* = 8.10 Hz, 2H), 6.38 (d, *J* = 7.27 Hz, 1H), 6.22 (brs, 2H), 1.33 (s, 12H).

^{13}C NMR (101 MHz, $CDCl_3$) δ: 151.32, 141.51, 139.46, 136.48, 128.52, 128.44, 128.29, 128.26, 128.21, 127.67, 119.91, 117.50, 105.88, 83.95, 24.97. (C-B) Carbon signal was not observed due to quadrupolar relaxation.[3]

^{11}B NMR (128 MHz, $CDCl_3$) δ: 31.9, 28.8.

HRMS (ESI) Calcd for $[C_{24}H_{26}B_2O_2N_2+H]^+$ $[M+H]^+$: m/z 397.2262, found 397.2271.

Note: The relative configuration of **2b** was determined by 2D-NMR NOESY (see Supplementary Figures 295-297, pages 245-246).

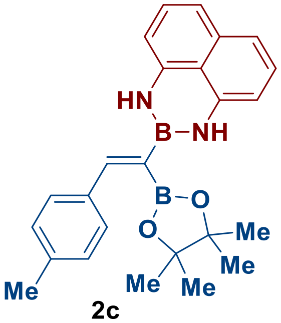

2c

(Z)-2-(1-(4,4,5,5-tetramethyl-1,3,2-dioxaborolan-2-yl)-2-(p-tolyl)vinyl)-2,3-dihydro-1H-naphtho[1,8-de][1,3,2]diazaborinine (2c):

Prepared according to general procedure H, product **2c** was isolated in (30.4 mg, 74% yield), *E:Z = 01:99*, as a yellow solid.

R_f = 0.51 (10% EtOAc in hexane).

^{1}H NMR (400 MHZ, $CDCl_3$) δ: 7.53 (s, 1H), 7.49 (d, *J* = 7.27 Hz, 2H), 7.16 - 7.10 (m, 4H), 7.01 (d, *J* = 7.99 Hz, 2H), 6.35 (d, *J* = 6.94 Hz, 2H), 6.19 (brs, 2H), 2.37 (s, 3H), 1.36 (s, 12H).

^{13}C NMR (101 MHz, $CDCl_3$) δ: 151.40, 141.63, 138.65, 136.65, 136.53, 128.99, 128.61, 128.53, 127.71, 119.92, 117.46, 105.85, 83.94, 25.05, 21.47.[3]

(C-B) Carbon signal was not observed due to quadrupolar relaxation.

^{11}B NMR (128 MHz, $CDCl_3$) δ: 32.8, 29.7.

HRMS (ESI) Calcd for $[C_{25}H_{28}B_2O_2N_2+H]^+$ $[M+H]^+$: m/z 411.2418, found 411.2421.

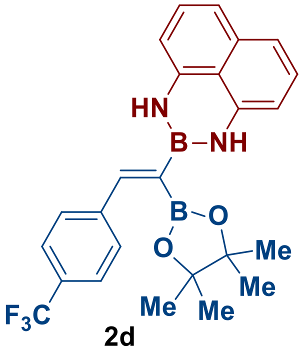

2d

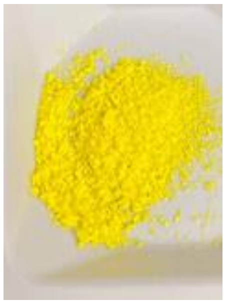

Picture of product **2d** from a gram scale reaction

(Z)-2-(1-(4,4,5,5-tetramethyl-1,3,2-dioxaborolan-2-yl)-2-(4-(trifluoromethyl)phenyl)vinyl)-2,3-dihydro-1H-naphtho[1,8-de][1,3,2]diazaborinine(2d):

Prepared according to general procedure H, product **2d** was isolated in (34.9 mg, 75% yield), *E:Z = 01:99*, as a yellow solid. A 1-gram scale reaction gave a (0.6 gr, 53% yield).

R_f = 0.45 (10% EtOAc in hexane).

^{1}**H NMR** (400 MHZ, $CDCl_3$) δ: 7.61 - 7.56 (m, 5H), 7.13 (t, *J* = 7.28 Hz, 2H), 7.03 (dd, *J* = 8.40, 0.89 Hz, 2H), 6.37 (dd, *J* = 7.29, 0.97 Hz, 2H), 6.23 (s, 2H), 1.33 (s, 12H).

^{13}**C NMR** (101 MHz, $CDCl_3$) δ: 149.47, 142.96, 141.35, 136.52, 128.63, 127.74, 125.24, 125.21, 120.02, 117.77, 106.04, 84.25, 25.01. (C-B) Carbon signal was not observed due to quadrupolar relaxation.[3]

^{11}**B NMR** (128 MHz, $CDCl_3$) δ: 32.6, 29.4.

^{19}**F NMR** (376 MHz, $CDCl_3$) δ: -62.5 (s).

HRMS (ESI) Calcd for $[C_{25}H_{25}B_2O_2N_2F_3+H]^+$ $[M+H]^+$: m/z 465.2136, found 465.2132.

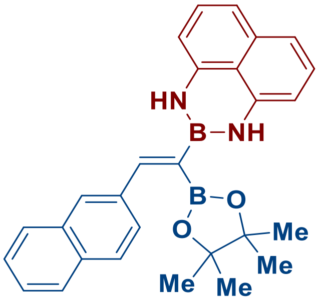

2e

(Z)-2-(2-(naphthalen-2-yl)-1-(4,4,5,5-tetramethyl-1,3,2-dioxaborolan-2-yl)vinyl)-2,3-dihydro-1H-naphtho[1,8-de][1,3,2]diazaborinine(2e):

Prepared according to general procedure H, product **2e** was isolated in (25.9 mg, 58% yield), *E:Z = 01:99*, as a yellow solid.

R_f = 0.48 (10% EtOAc in hexane).

^{1}**H NMR** (400 MHZ, $CDCl_3$) δ: 7.95 (s, 1H), 7.84 - 7.79 (m, 3H), 7.73 (s, 1H), 7.63 (dd, *J* = 8.43, 1.71 Hz, 1H), 7.50 - 7.46 (m, 2H), 7.13 (t, *J* = 7.30 Hz, 2H), 7.02 (dd, *J* = 8.37, 0.84 Hz, 2H), 6.37 (dd, *J* = 7.29, 0.93 Hz, 2H), 6.23 (s, 2H), 1.36 (s, 12H).

^{13}**C NMR** (101 MHz, $CDCl_3$) δ: 151.37, 141.59, 137.12, 136.54, 133.49, 133.36, 128.36, 127.99, 127.85, 127.74, 126.44, 126.40, 126.27, 119.98, 117.57, 105.94, 84.09, 25.11. (C-B) Carbon signal was not observed due to quadrupolar relaxation.[3]

^{11}**B NMR** (128 MHz, $CDCl_3$) δ: 28.6.

HRMS (ESI) Calcd for $[C_{28}H_{28}B_2O_2N_2+H]^+$ $[M+H]^+$: m/z 447.2419, found 447.2420.

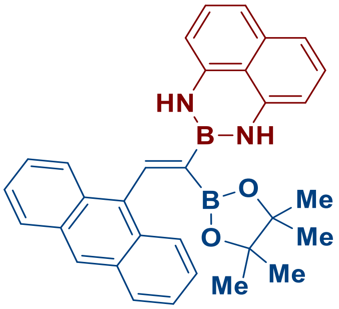

2f

(Z)-2-(2-(anthracen-9-yl)-1-(4,4,5,5-tetramethyl-1,3,2-dioxaborolan-2-yl)vinyl)-2,3-dihydro-1H-naphtho[1,8-de][1,3,2]diazaborinine (2f):

Prepared according to general procedure H, product **2f** was isolated in (32.3 mg, 65% yield), *E:Z = 01:99*, as a yellow solid.

R_f = 0.36 (5% EtOAc in hexane).

^{1}H NMR (400 MHZ, $CDCl_3$) δ: 8.39 (d, *J* = 8.60 Hz, 2H), 8.20 - 8.17 (m, 2H), 8.02 - 7.99 (m, 2H), 7.50 - 7.45 (m, 4H), 7.16 (dd, *J* = 8.15, 7.30 Hz, 2H), 7.06 (dd, *J* = 8.28, 0.59 Hz, 2H), 6.48 (s, 2H), 6.44 (dd, *J* = 7.25, 0.73 Hz, 2H), 0.70 (s, 12H).

^{13}C NMR (101 MHz, $CDCl_3$) δ: 150.61, 141.63, 136.61, 135.70, 131.39, 129.31, 128.64, 127.76, 126.48, 126.29, 125.39, 125.19, 120.16, 117.61, 106.05, 83.14, 24.37. (C-B) Carbon signal was not observed due to quadrupolar relaxation.[3]

^{11}B NMR (128 MHz, $CDCl_3$) δ: 30.1.

HRMS (ESI) Calcd for $[C_{32}H_{30}B_2O_2N_2+H]^+$ $[M+H]^+$: m/z 497.2577, found 497.2575.

Note: The relative configuration of **2f** was determined by 2D-NMR NOESY (see Supplementary Figures 298-300, pages 246-247).

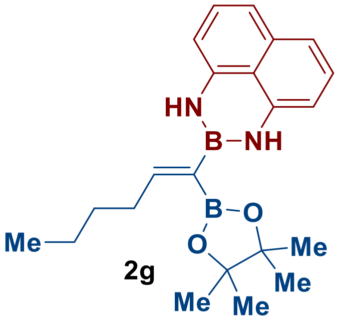

(Z)-2-(1-(4,4,5,5-tetramethyl-1,3,2-dioxaborolan-2-yl)hex-1-en-1-yl)-2,3-dihydro-1H-naphtho[1,8-de][1,3,2]diazaborinine (2g):

Prepared according to general procedure H, product **2g** was isolated in (25.6 mg, 68% yield), *E:Z = 03:97*, as a yellow solid.

R_f = 0.51 (10% EtOAc in hexane).

^{1}H NMR (400 MHZ, $CDCl_3$) δ: 7.10 (dd, *J* = 8.25, 7.33 Hz, 2H), 6.97 (d, *J* = 7.84 Hz, 2H), 6.87 (t, *J* = 7.20 Hz, 1H), 6.30 (dd, *J* = 7.32, 0.64 Hz, 2H), 6.24 (s, 2H), 2.51 (q, *J* = 7.32 Hz, 2H), 1.49 - 1.35 (m, 4H), 1.34 (s, 12H), 0.93 (t, *J* = 7.22 Hz, 3H).

^{13}C NMR (101 MHz, $CDCl_3$) δ: 161.07, 141.90, 136.53, 127.67, 119.83, 117.08, 105.63, 83.25, 34.13, 31.72, 25.10, 22.50, 14.09. (C-B) Carbon signal was not observed due to quadrupolar relaxation.[3]

^{11}B NMR (128 MHz, $CDCl_3$) δ: 32.8, 29.7.

HRMS (ESI) Calcd for $[C_{22}H_{30}B_2O_2N_2+H]^+$ $[M+H]^+$: m/z 377.2574, found 377.2580.

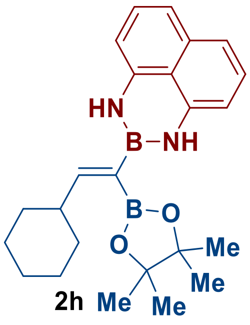

(Z)-2-(2-cyclohexyl-1-(4,4,5,5-tetramethyl-1,3,2-dioxaborolan-2-yl)vinyl)-2,3-dihydro-1H-naphtho[1,8-de][1,3,2]diazaborinine (2h):

Prepared according to general procedure H, product **2h** was isolated in (25.4 mg, 63% yield), *E:Z = 03:97*, as a yellow solid.

R_f = 0.45 (10% EtOAc in hexane).

^{1}H NMR (400 MHZ, $CDCl_3$) δ: 7.09 (dd, *J* = 8.27, 7.31 Hz, 2H), 6.97 (dd, *J* = 8.30, 0.78 Hz, 2H), 6.67 (d, *J* = 8.83 Hz, 1H), 6.32 (dd, *J* = 7.32, 0.90 Hz, 2H), 6.21 (s, 2H), 2.79 - 2.69 (m, 1H), 1.78 - 1.67 (m, 5H), 1.34 (s, 12H), 1.30 - 1.10 (m, 5H).

^{13}C NMR (101 MHz, $CDCl_3$) δ: 165.88, 141.89, 136.52, 127.66, 119.83, 117.08, 105.62, 83.24, 42.72, 33.28, 26.10, 25.93, 25.06. (C-B) Carbon signal was not observed due to quadrupolar relaxation.[3]

^{11}B NMR (128 MHz, $CDCl_3$) δ: 31.1, 28.5.

HRMS (ESI) Calcd for $[C_{24}H_{32}B_2O_2N_2+H]^+$ $[M+H]^+$: m/z 403.2731, found 403.2733.

Note: The relative configuration of **2h** was determined by 2D-NMR NOESY (see Supplementary Figures 301-303, pages 248-249).

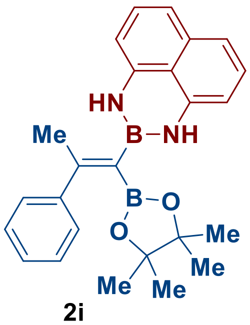

2i

(Z)-2-(2-phenyl-1-(4,4,5,5-tetramethyl-1,3,2-dioxaborolan-2-yl)prop-1-en-1-yl)-2,3-dihydro-1H-naphtho[1,8-de][1,3,2]diazaborinine (2i):

Prepared according to general procedure H, product **2i** was isolated in (25.5 mg, 62% yield), *E:Z = 10:90*, as a yellow solid.

R_f = 0.39 (10% EtOAc in hexane).

^{1}H NMR (400 MHZ, $CDCl_3$) δ: 7.27 - 7.18 (m, 5H), 7.03 (dd, *J* = 8.40, 7.33 Hz, 2H), 6.94 (dd, *J* = 8.40, 0.93 Hz, 2H), 6.25 (dd, *J* = 7.34, 0.99 Hz, 2H), 5.82 (brs, 2H), 2.23 (s, 3H), 1.01(s, 12H).

^{13}C NMR (101 MHz, $CDCl_3$) δ: 158.69, 146.69, 141.55, 136.48, 128.08, 127.73, 127.41, 127.06, 119.77, 117.55, 105.81, 83.52, 25.76, 24.74. (C-B) Carbon signal was not observed due to quadrupolar relaxation.[3]

^{11}B NMR (128 MHz, $CDCl_3$) δ: 32.0, 27.3.

HRMS (ESI) Calcd for $[C_{25}H_{28}B_2O_2N_2+H]^+$ $[M+H]^+$: m/z 411.2415, found 411.2422.

Note: The relative configuration of **2i** was determined by 2D-NMR NOESY (see Supplementary Figure 304, page 249).

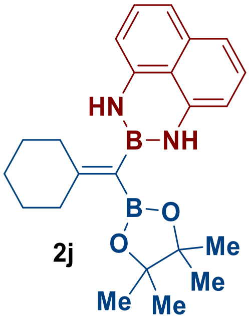

***2-(cyclohexylidene(4,4,5,5-tetramethyl-1,3,2-dioxaborolan-2-yl)methyl)-2,3-dihydro-1H-naphtho[1,8-de][1,3,2]diazaborinine (2j)*:**

Prepared according to general procedure H, product **2j** was isolated in (21 mg, 54% yield) as a yellow solid.

R_f = 0.42 (10% EtOAc in hexane).

^{1}H NMR (400 MHZ, $CDCl_3$) δ: 7.10 (dd, *J* = 8.34, 7.30 Hz, 2H), 6.99 (dd, *J* = 8.45, 0.91 Hz, 2H), 6.27 (dd, *J* = 7.34, 0.94 Hz, 2H), 5.75 (s, 2H), 2.44 - 2.35 (m, 4H), 1.66 - 1.58 (m, 6H), 1.28 (s, 12H).

^{13}C NMR (101 MHz, $CDCl_3$) δ: 166.95, 141.80, 136.48, 127.68, 119.64, 117.23, 105.57, 83.32, 37.56, 37.52, 29.24, 29.06, 26.60, 24.96, 24.90. (C-B) Carbon signal was not observed due to quadrupolar relaxation.[3]

^{11}B NMR (128 MHz, $CDCl_3$) δ: 31.3, 28.2.

HRMS (ESI) Calcd for $[C_{23}H_{30}B_2O_2N_2+H]^+$ $[M+H]^+$: m/z 389.2575, found 389.2587.

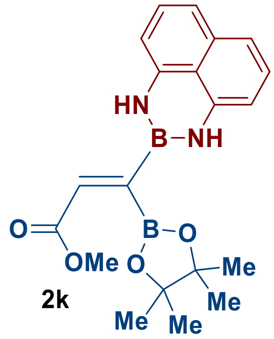

2k

Methyl (Z)-3-(1H-naphtho[1,8-de][1,3,2]diazaborinin-2(3H)-yl)-3-(4,4,5,5-tetramethyl-1,3,2-dioxaborolan-2-yl)acrylate (2k):

Prepared according to general procedure H, product **2k** was isolated in (29.2 mg, 77% yield), *E:Z = 01:99*, as a yellow solid.

R_f = 0.3 (15% EtOAc in hexane).

^{1}H NMR (400 MHZ, $CDCl_3$) δ: 7.10 (dd, *J* = 8.15, 7.40 Hz, 2H), 7.03 (d, *J* = 8.46 Hz, 2H), 6.59 (s, 1H), 6.32 (dd, *J* = 7.30, 0.88 Hz, 2H), 5.91 (brs, 2H), 3.79 (s, 3H), 1.41 (s, 12H).

^{13}C NMR (101 MHz, $CDCl_3$) δ: 167.35, 140.62, 136.33, 135.77, 127.57, 120.12, 118.10, 106.19, 84.35, 52.10, 24.93.(C-B) Carbon signal was not observed due to quadrupolar relaxation.[3]

^{11}B NMR (128 MHz, $CDCl_3$) δ: 31.6, 28.7.

HRMS (ESI) Calcd for $[C_{20}H_{24}B_2O_4N_2+H]^+$ $[M+H]^+$: m/z 379.2002, found 379.2027.

Note: The relative configuration of **2k** was determined by 2D-NMR NOESY (see Supplementary Figures 305-306, page 250).

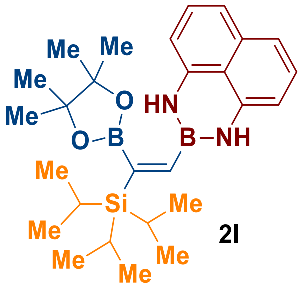

2l

***(E)-2-(2-(4,4,5,5-tetramethyl-1,3,2-dioxaborolan-2-yl)-2-(triisopropylsilyl)vinyl)-2,3-dihydro-1H-naphtho[1,8-de][1,3,2]diazaborinine (2l)*:**

Prepared according to general procedure H, product **2l** was isolated in (38.1 mg, 80% yield), *E:Z = 99:01*, as a yellow solid.

R_f = 0.59 (5% EtOAc in hexane).

^{1}**H NMR** (400 MHZ, $CDCl_3$) δ: 7.08 (dd, *J* = 8.40, 7.33 Hz, 2H), 6.98 (dd, *J* = 8.40, 0.93 Hz, 2H), 6.82 (brs, 1H), 6.38 (s, 2H), 6.29 (dd, *J* = 7.34, 0.99 Hz, 2H), 1.32 (s, 12H), 1.10 - 1.05 (m, 21H).

^{13}**C NMR** (101 MHz, $CDCl_3$) δ: 141.62, 136.58, 127.72, 120.15, 117.40, 105.70, 83.87, 25.32, 18.93, 11.45. (C-B) Carbon signal was not observed due to quadrupolar relaxation.[3]

^{11}**B NMR** (128 MHz, $CDCl_3$) δ: 32.0, 27.3.

HRMS (ESI) Calcd for $[C_{27}H_{42}B_2O_2N_2Si+H]^+$ $[M+H]^+$: m/z 477.3280, found 477.3289.

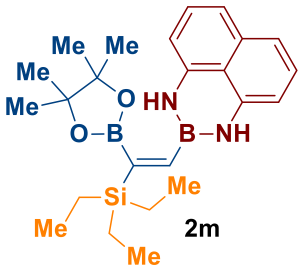

2m

(E)-2-(2-(4,4,5,5-tetramethyl-1,3,2-dioxaborolan-2-yl)-2-(triethylsilyl)vinyl)-2,3-dihydro-1H-naphtho[1,8-de][1,3,2]diazaborinine (2m)**:**

Prepared according to general procedure H, product **2m** was isolated in (33 mg, 76% yield), *E:Z = 99:01*, as a yellow solid.

R_f = 0.60 (5% EtOAc in hexane).

^{1}**H NMR** (400 MHZ, $CDCl_3$) δ: 7.07 (t, *J* = 7.62 Hz, 2H), 6.97 (d, *J* = 8.19 Hz, 2H), 6.82 (s, 1H), 6.48 (s, 2H), 6.27 (d, *J* = 7.29 Hz, 2H), 1.34 (s, 12H), 0.95 (t, *J* = 7.89 Hz, 9H), 0.68 (q, *J* = 7.89 Hz, 6H).

^{13}**C NMR** (101 MHz, $CDCl_3$) δ: 141.73, 136.59, 127.73, 120.20, 117.36, 105.67, 83.90, 25.24, 7.63, 3.44. (C-B) Carbon signal was not observed due to quadrupolar relaxation.[3]

^{11}**B NMR** (128 MHz, $CDCl_3$) δ: 31.9, 27.2.

HRMS (ESI) Calcd for $[C_{24}H_{36}B_2O_2N_2Si+H]^+$ $[M+H]^+$: m/z 435.2814, found 435.2806.

Note: The relative configuration of **2m** was determined by 2D-NMR NOESY (see Supplementary Figures 307-309, pages 251-252).

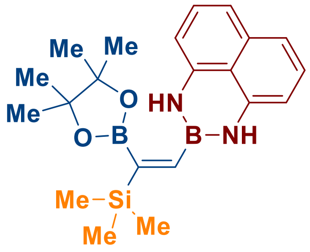

2n

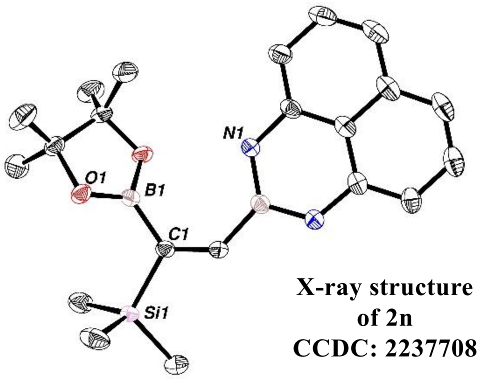

X-ray structure of 2n CCDC: 2237708

***(E)-2-(2-(4,4,5,5-tetramethyl-1,3,2-dioxaborolan-2-yl)-2-(trimethylsilyl)vinyl)-2,3-dihydro-1H-naphtho[1,8-de][1,3,2]diazaborinine (2n)*:**

Prepared according to general procedure H, product **2n** was isolated in (28.7 mg, 73% yield), *E:Z = 99:01*, as a yellow solid.

R_f = 0.61 (5% EtOAc in hexane).

^{1}H NMR (400 MHZ, $CDCl_3$) δ: 7.09 (dd, *J* = 8.34, 7.32 Hz, 2H), 6.98 (dd, *J* = 8.39, 0.93 Hz, 2H), 6.85 (s, 1H), 6.50 (brs, 2H), 6.28 (dd, *J* = 7.33, 0.98 Hz, 2H), 1.35 (s, 12H), 0.16 (s, 9H).

^{13}C NMR (101 MHz, $CDCl_3$) δ: 141.72, 136.58, 127.73, 120.21, 117.38, 105.67, 84.00, 25.23, -1.05. (C-B) Carbon signal was not observed due to quadrupolar relaxation.[3]

^{11}B NMR (128 MHz, $CDCl_3$) δ: 31.7, 27.1.

HRMS (ESI) Calcd for $[C_{21}H_{30}B_2O_2N_2Si+H]^+$ $[M+H]^+$: m/z 393.2344, found 393.2348.

Notes: (1) The structure of **2n** was confirmed by X-ray crystallographic analysis, CCDC 2237708 (see Supplementary Table 5, page 92). (2) Compound **2n** was recrystallized by using EtOAc as a solvent. (3) The relative configuration of **2n** was also determined by 2D-NMR NOESY (see Supplementary Figures 310-312, pages 252-253).

2.4. Procedure I and Characterizations for *gem*-(Bdan,Bdan)-alkene (2a-dan)

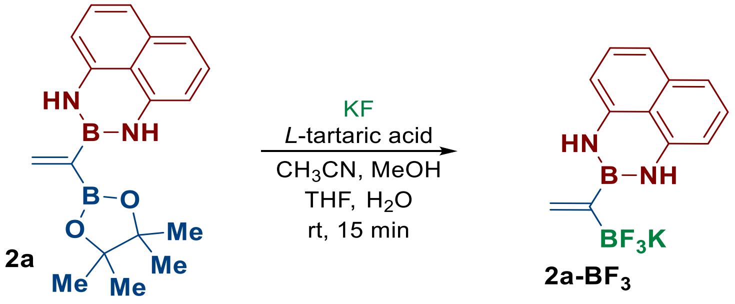

To initiates the reaction, a diboroalkene substrate **2a** (0.10 mmol) was added to an air-open flask followed by the addition of a mixture of acetonitrile (0.5 mL) and methanol (0.5 mL). Following that, to this mixture, a solution of fluoride salt i.e. KF or CsF (0.35 mmol) in H_2O (0.2 mL) was added, and stirring was continued for 2 minutes at room temperature. Next, *L*-tartaric acid (0.20 mmol) in THF (0.7 mL) was gradually added to the mixture, which was being stirred vigorously. The stirring was continued for an additional 4 minutes, during which a white precipitate formed. The mixture was filtered to remove the white precipitate and thoroughly washed with excess acetonitrile (5 mL). The filtrate was then concentrated using a reduced-pressure evaporator, resulting in a residue of crude solid. The solid residue was washed with diethyl ether and hexane, giving rise to the corresponding organotrifluoroborate salt alkene **2a-BF$_3$** as an amorphous white solid. The solid was then dried further under high vacuum overnight.

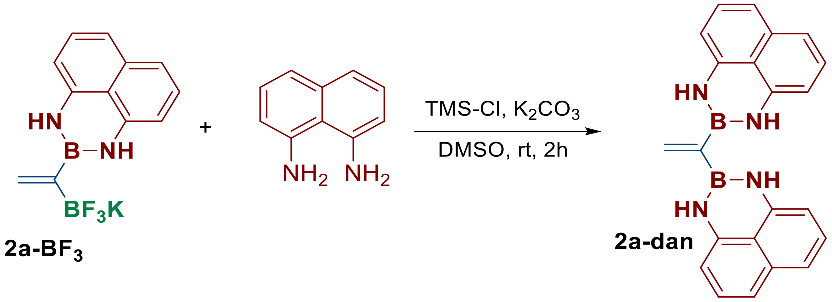

An oven-dried 10 ml Schlenk tube was cooled under N_2 before the addition of potassium carbonate (3 equiv, 0.3 mmol) and trifluoroborate salt (**2a-BF$_3$**) (0.1 mmol). DMSO (1 ml) was then added to the reaction mixture along with 1,8-diaminonaphthalene (1.5 equiv, 0.15 mmol), and following the dropwise addition of trimethylsilyl chloride TMS-Cl (3 equiv, 0.3 mmol), then the mixture was stirred for 2 hours at room temperature. After completion of the reaction, the mixture was diluted

with EtOAc (3 ml) and H_2O (3 ml), and the residue was extracted with EtOAc (3 × 5 mL). The organic layers were then washed with brine, dried over $MgSO_4$, and evaporated by an evaporator under reduced pressure to obtain a crude material. The crude material was further purified by a short column on silica gel, resulting in the formation of a yellow solid, which was identified as product (**2a-dan**).

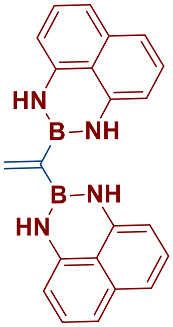

2a-dan

2,2'-(ethene-1,1-diyl)bis(2,3-dihydro-1H-naphtho[1,8-de][1,3,2]diazaborinine) (2a-dan)**:**
Prepared according to procedure I, product **2a-dan** was isolated in (57.8 mg, 80% yield) as a yellow solid.

R_f = 0.38 (15% EtOAc in hexane).

^{1}H NMR (400 MHZ, $CDCl_3$) δ: 7.13 (dd, *J* = 8.30, 7.30 Hz, 4H), 7.06 (dd, *J* = 8.45, 0.92 Hz, 4H), 6.36 (dd, *J* = 7.24, 0.99 Hz, 4H), 6.27 (s, 2H), 5.79 (s, 4H).

^{13}C NMR (101 MHz, $CDCl_3$) δ: 141.03, 136.74, 136.45, 127.75, 119.96, 118.08, 106.19. (C-B) Carbon signal was not observed due to quadrupolar relaxation.[3]

^{11}B NMR (128 MHz, $CDCl_3$) δ: 27.9.

HRMS (ESI) Calcd for $[C_{22}H_{18}B_2N_4+H]^+$ $[M+H]^+$: m/z 361.1798, found 361.1798.

2.5. General Procedure J and Characterization for *gem*-(Bpin,BMIDA)-alkene (3, 4)

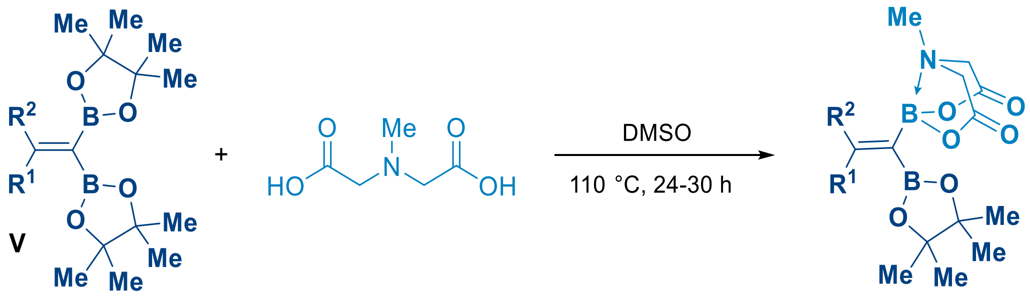

R[1] = H, Alkly, Aryl, Bpin, Si(R)$_3$
R[2] = H, Alkyl, Aryl, Bpin

Inside the glove box, to a 10 mL thick-walled reaction tube equipped with a magnetic stirring bar, *gem*-diborylalkene (**V**) (0.1 mmol, 1 equiv) and N-methyliminodiacetic acid (MIDA) (0.4 mmol, 4 equiv) were added, followed by 1 ml of DMSO. The tube was sealed with a crimped septum cap, taken out of the glove box and heated at 110 °C for the indicated amount of time (15-30 h). The reaction mixture was then diluted with a dropwise addition of water (1 mL) and extracted with EtOAc (2 x 5 ml). The combined organic phase was washed with brine 10 ml, and dried over magnesium sulfate. The solvents were removed under reduced pressure (by evaporator), forming a crude material as a solid,[(1)] that was then washed several times with diethyl ether and hexane, yielding **3**, **4** as a solid. [(2)]

Notes: (1) If the crude material was not solid, it can be left on high vacuum for a few hours until it solidifies. (2) For some products a column chromatography was necessary to fully purify the compounds.

Supplementary Table 4. Scope of *gem*-(Bpin,BMIDA)-alkene (**3**, **4**) products:

Entry	Substrate	Time (h)	Yield (%)[a]	Isomeric ratio	Product
**1**	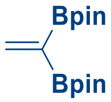 **V-1a**	12	63	-	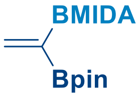 **3a**
**2**	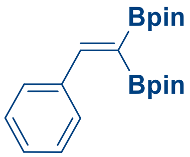 **V-1b**	24	75	*E:Z = 99:01*	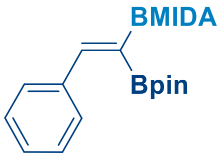 **3b**
**3**	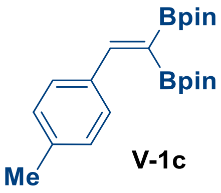 **V-1c**	24	69	*E:Z = 99:01*	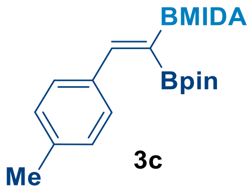 **3c**
**4**	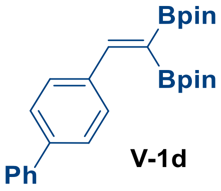 **V-1d**	30	71	*E:Z = 99:01*	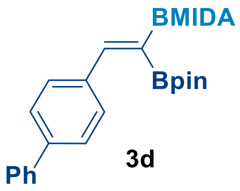 **3d**
**5**	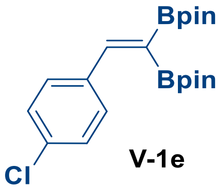 **V-1e**	20	42	*E:Z = 99:01*	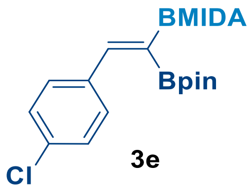 **3e**
**6**	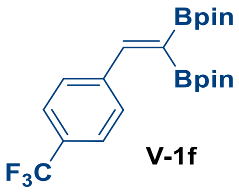 **V-1f**	30	73	*E:Z = 99:01*	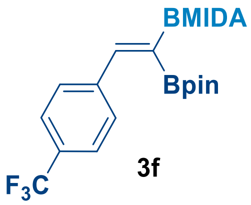 **3f**
**7**	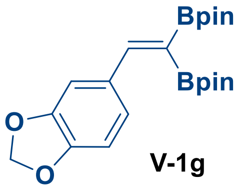 **V-1g**	30	63	*E:Z = 99:01*	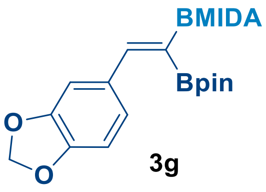 **3g**

**8**	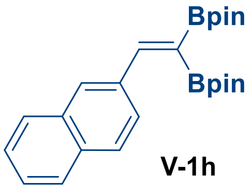 **V-1h**	24	64	*E:Z = 99:01*	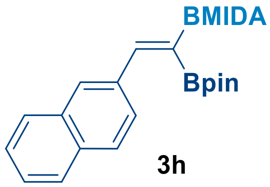 **3h**
**9**	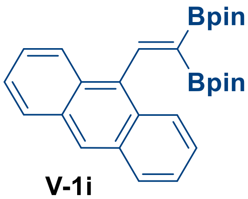 **V-1i**	30	75	*E:Z = 99:01*	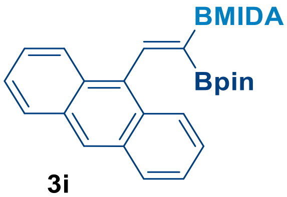 **3i**
**10**	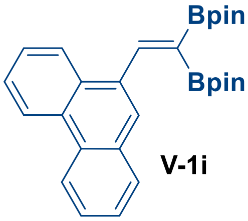 **V-1i**	30	52	*E:Z = 99:01*	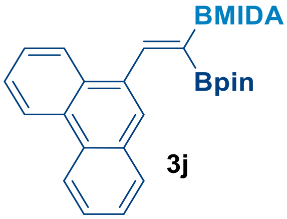 **3j**
**11**	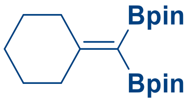 **V-1m**	30	62	-	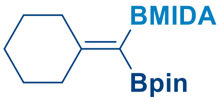 **3k**
**12**	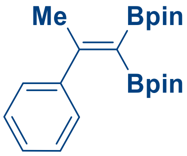 **V-1n**	30	64	*E:Z = 99:01*	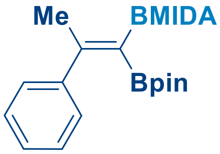 **3l**
**13**	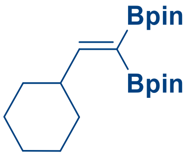 **V-1k**	30	73	*E:Z = 90:10*	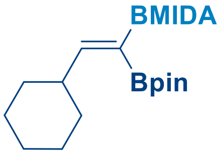 **3m**
**14**	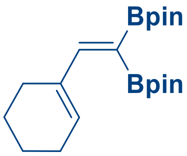 **V-1l**	30	58	*E:Z = 99:01*	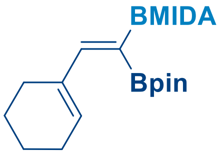 **3n**

**15**	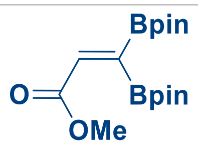<br>**V-1o**	24	73	*E:Z = 99:01*	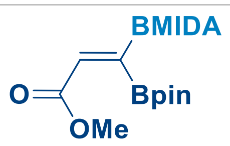<br>**3o**
**16**	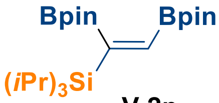<br>**V-2p**	24	76	*E:Z = 99:01*<br>*s.s. = 99:01*	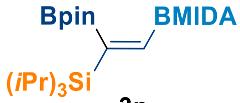<br>**3p**
**17**	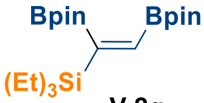<br>**V-2q**	24	79	*E:Z = 99:01*<br>*s.s. = 99:01*	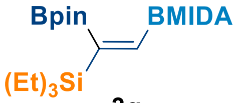<br>**3q**
**18**	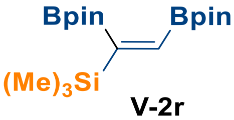<br>**V-2r**	24	81	*E:Z = 99:01*<br>*s.s. = 99:01*	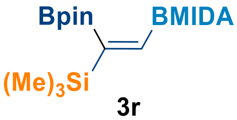<br>**3r**
**19**	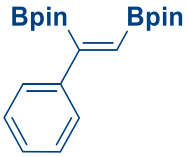<br>**V-2s**	30	81	*E:Z = 99:01*<br>*s.s. = 99:01*	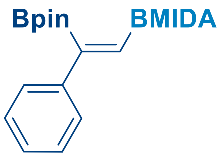<br>**3s**
**20**	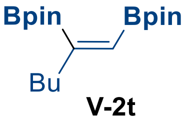<br>**V-2t**	30<br>T = 90 °C	46	*E:Z = 95:05*<br>*s.s. = 99:01*	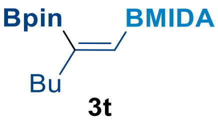<br>**3t**
**21**	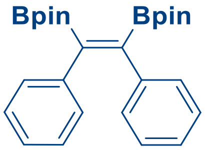<br>**V-2u**	30	75	*E:Z = 01:99*	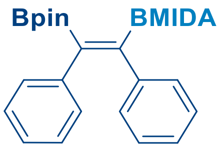<br>**3u**
**22**	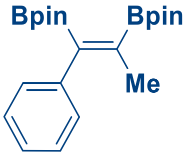<br>**V-2v**	24	64	*E:Z = 01:99*<br>*s.s. = 99:01*	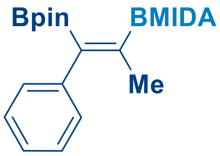<br>**3v**
**23**	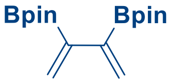<br>**V-2w**	35	46	-	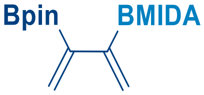<br>**3w**
**24**	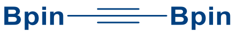<br>**V-2x**	24	76	-	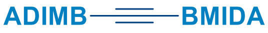<br>**3x**

**25**	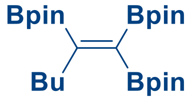 **V-3y**	30	49	*E:Z = 99:01*<br>*s.s.* = *99:01*	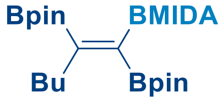 **3y**
**26**	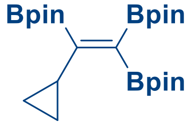 **V-3z**	30	48	*E:Z = 99:01*<br>*s.s.* = *99:01*	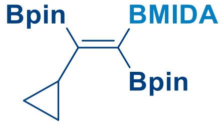 **3z**
**27**	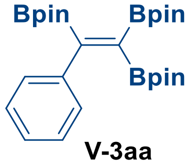 **V-3aa**	30	70	*E:Z = 99:01*<br>*s.s.* = *99:01*	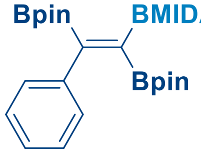 **3aa**
**28**	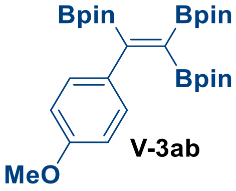 **V-3ab**	30	72	*E:Z = 99:01*<br>*s.s.* = *99:01*	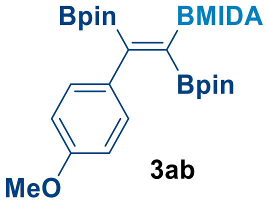 **3ab**
**29**	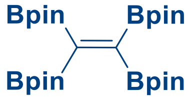 **V-4**	30 (48)	66 (69)	-	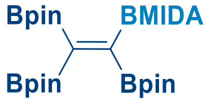 **4**

[a] Isolated yield. [b] *E:Z = isomeric ratio.* [c] *s.s.* = site selectivity ratio.

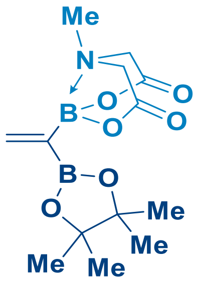

3a

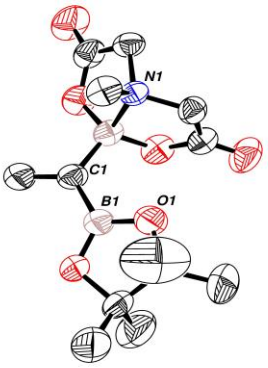

X-ray structure of 3a
CCDC : 2194038

***6-methyl-2-(1-(4,4,5,5-tetramethyl-1,3,2-dioxaborolan-2-yl)vinyl)-1,3,6,2-dioxazaborocane-4,8-dione (3a)*:**

Prepared according to general procedure J, product **3a** was isolated in (19.5 mg, 63% yield) as a white solid.

^{1}H NMR (400 MHZ, $CDCl_3$) δ: 6.57 (d, *J* = 11.78 Hz, 2H), 3.98 (d, *J* = 16.03 Hz, 2H), 3.81 (d, *J* = 16.03 Hz, 2H), 2.78 (s, 3H), 1.25 (s, 12H).

^{13}C NMR (101 MHz, CD_3CN) δ: 169.47, 143.56, 118.22, 84.33, 63.53, 47.97, 24.93. (C-B) Carbon signal was not observed due to quadrupolar relaxation.[3]

^{11}B NMR (128 MHz, $CDCl_3$) δ: 31.6, 11.9.

HRMS (ESI) Calcd for $[C_{13}H_{21}B_2NO_6+H]^+$ $[M+H]^+$: m/z 310.1633, found 310.1636.

Notes: (1) The structure of **3a** was confirmed by X-ray crystallographic analysis, CCDC 2194038 (see Supplementary Table 6, page 93). (2) Compound **3a** was recrystallized by using a solvent mixture of hexane:EtOAc (10:1).

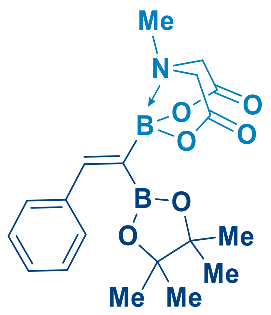

3b

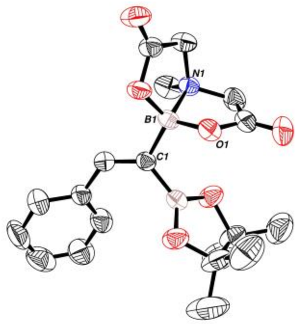

X-ray structure of 3b CCDC : 2194037

***(E)-6-methyl-2-(2-phenyl-1-(4,4,5,5-tetramethyl-1,3,2-dioxaborolan-2-yl)vinyl)-1,3,6,2-dioxazaborocane-4,8-dione (3b)*:**

Prepared according to general procedure J, product **3b** was isolated in (28.9 mg, 75% yield), *E:Z = 99:01*, as a white solid. A 1-gram scale reaction gave a (47%) yield.

^{1}H NMR (400 MHZ, $CDCl_3$) δ: 7.57 (s, 1H), 7.42 (dd, *J* = 7.90, 1.93 Hz, 2H), 7.31 - 7.27 (m, 3H), 3.96 (d, *J* = 16.22 Hz, 2H), 3.82 (d, *J* = 16.25 Hz, 2H), 2.90 (s, 3H), 1.26 (s, 12H).

^{13}C NMR (101 MHz, $CDCl_3$) δ: 167.64, 152.69, 139.41, 128.56, 128.22, 84.05, 62.95, 47.86, 25.01. (C-B) Carbon signal was not observed due to quadrupolar relaxation.[3]

^{11}B NMR (128 MHz, $CDCl_3$) δ: 32.5, 11.5.

HRMS (ESI) Calcd for $[C_{19}H_{25}B_2NO_6+H]^+$ $[M+H]^+$: m/z 386.1948, found 386.1954.

Notes: (1) The structure of **3b** was confirmed by X-ray crystallographic analysis, CCDC 2194037 (see Supplementary Table 7, page 94). (2) Compound **3b** was recrystallized by using chloroform as a solvent.

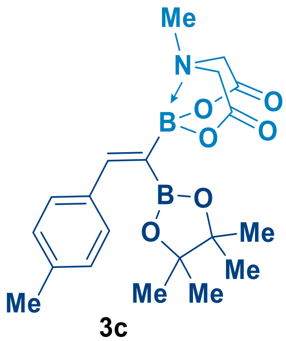

3c

***(E)-6-methyl-2-(1-(4,4,5,5-tetramethyl-1,3,2-dioxaborolan-2-yl)-2-(p-tolyl)vinyl)-1,3,6,2-dioxazaborocane-4,8-dione (3c)*:**

Prepared according to general procedure J, product **3c** was isolated in (27.6 mg, 69% yield), *E:Z = 99:01*, as a white solid.

^{1}H NMR (400 MHZ, $CDCl_3$) δ: 7.48 (s, 1H), 7.31 (d, *J* = 8.07 Hz, 2H), 7.09 (d, *J* = 7.90 Hz, 2H), 3.95 (dd, *J* = 23.30, 4.72 Hz, 4H), 2.86 (s, 3H), 2.33 (s, 3H), 1.25 (s, 12H).

^{13}C NMR (101 MHz, $CDCl_3$) δ: 168.43, 152.09, 138.41, 136.57, 128.91, 128.51, 83.92, 62.85, 47.98, 24.98, 21.38. (C-B) Carbon signal was not observed due to quadrupolar relaxation.[3]

^{11}B NMR (128 MHz, $CDCl_3$) δ: 32.4, 11.5.

HRMS (ESI) Calcd for $[C_{20}H_{27}B_2NO_6+H]^+$ $[M+H]^+$: m/z 400.2104, found 400.2105.

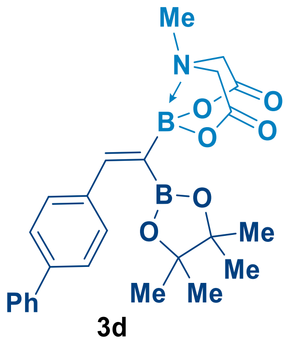

3d

***(E)-2-(2-([1,1'-biphenyl]-4-yl)-1-(4,4,5,5-tetramethyl-1,3,2-dioxaborolan-2-yl)vinyl)-6-methyl-1,3,6,2-dioxazaborocane-4,8-dione (3d)*:**

Prepared according to general procedure J, product **3d** was isolated in (32.3 mg, 71% yield), *E:Z = 99:01*, as a white solid.

^{1}H NMR (400 MHZ, $CDCl_3$) δ: 7.60 - 7.58 (m, 3H), 7.55 (q, *J* = 8.50 Hz, 4H), 7.45 (t, *J* = 7.51 Hz, 2H), 7.34 (tt, *J* = 1.21 Hz, 1H), 3.95 (d, *J* = 16.33 Hz, 2H), 3.91 (d, *J* = 16.33 Hz, 2H), 2.91 (s, 3H), 1.28 (s, 12H).

^{13}C NMR (101 MHz, $CDCl_3$) δ: 168.15, 151.91, 141.22, 140.76, 138.34, 129.08, 128.92, 127.56, 127.14, 126.91, 84.08, 62.95, 47.99, 25.02. (C-B) Carbon signal was not observed due to quadrupolar relaxation.[3]

^{11}B NMR (128 MHz, $CDCl_3$) δ: 33.0, 12.0.

HRMS (ESI) Calcd for $[C_{25}H_{29}B_2NO_6+H]^+$ $[M+H]^+$: m/z 462.2263, found 462.2262.

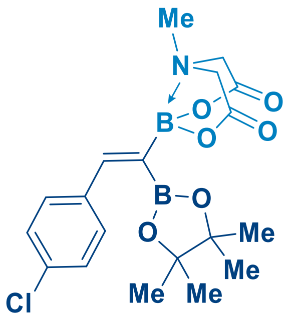

3e

***(E)-2-(2-(4-chlorophenyl)-1-(4,4,5,5-tetramethyl-1,3,2-dioxaborolan-2-yl)vinyl)-6-methyl-1,3,6,2-dioxazaborocane-4,8-dione (3e)*:**

Prepared according to general procedure J, product **3e** was isolated in (18.5 mg, 42% yield), *E:Z = 99:01*, as a white solid.

^{1}H NMR (400 MHZ, $CDCl_3$) δ: 7.47 (s, 1H), 7.35 - 7.33 (m, 2H), 7.25 - 7.23 (m, 2H), 4.02 (d, *J* = 16.34 Hz, 2H), 3.92 (d, *J* = 16.34 Hz, 2H), 2.88 (s, 3H), 1.23 (s, 12H).

^{13}C NMR (101 MHz, $CDCl_3$) δ: 168.50, 150.87, 137.91, 134.12, 129.80, 128.32, 84.08, 62.93, 48.03, 24.95. (C-B) Carbon signal was not observed due to quadrupolar relaxation.[3]

^{11}B NMR (128 MHz, $CDCl_3$) δ: 32.1, 11.2.

HRMS (ESI) Calcd for $[C_{19}H_{24}B_2NO_6Cl+Na]^+$ $[M+Na]^+$: m/z 442.1376, found 442.1382.

Note: The relative configuration of **3e** was determined by 2D-NMR NOESY (see Supplementary Figures 313-314, page 254).

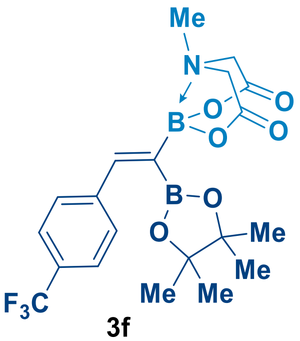

3f

***(E)-6-methyl-2-(1-(4,4,5,5-tetramethyl-1,3,2-dioxaborolan-2-yl)-2-(4-(trifluoromethyl)phenyl)vinyl)-1,3,6,2-dioxazaborocane-4,8-dione (3f)*:**

Prepared according to general procedure J, product **3f** was isolated in (34.7 mg, 73% yield), *E:Z = 99:01*, as a white solid.

^{1}H NMR (400 MHZ, $CDCl_3$) δ: 7.59 - 7.49 (m, 5H), 3.98 (d, *J* = 16.13 Hz, 2H), 3.90 (d, *J* = 16.13 Hz, 2H), 2.91 (s, 3H), 1.25 (s, 12H).

^{13}C NMR (101 MHz, $CDCl_3$) δ: 167.65, 151.02, 142.82, 128.71, 125.17, 125.14, 84.30, 63.05, 47.89, 24.97. (C-B) Carbon signal was not observed due to quadrupolar relaxation.[3]

^{11}B NMR (128 MHz, $CDCl_3$) δ: 31.10.

^{19}F NMR (376 MHz, $CDCl_3$) δ: -62.5 (s).

HRMS (ESI) Calcd for $[C_{13}H_{21}B_2NO_6+Na]^+$ $[M+H]^+$: m/z 476.1640, found 476.1646.

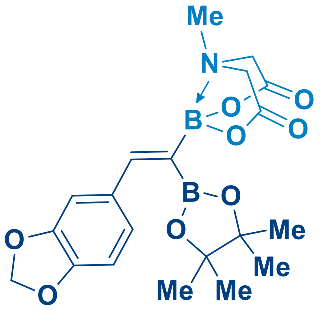

3g

***(E)-2-(2-(benzo[d][1,3]dioxol-5-yl)-1-(4,4,5,5-tetramethyl-1,3,2-dioxaborolan-2-yl)vinyl)-6-methyl-1,3,6,2-dioxazaborocane-4,8-dione (3g)*:**

Prepared according to general procedure J, product **3g** was isolated in (27.1 mg, 63% yield), *E:Z = 99:01*, as a white solid.

^{1}**H NMR** (400 MHZ, $CDCl_3$) δ: 7.43 (s, 1H), 7.00 (d, *J* = 1.73 Hz, 1H), 6.88 (d, *J* = 7.91, 0.62 Hz, 1H), 6.74 (d, *J* = 8.01 Hz, 1H), 5.95 (s, 2H), 3.92 (d, *J* = 16.33 Hz, 2H), 3.88 (d, *J* = 16.33 Hz, 2H), 2.87 (s, 3H), 1.27 (s, 12H).

^{13}**C NMR** (101 MHz, $CDCl_3$) δ: 168.16, 151.89, 147.97, 147.72, 133.91, 123.44, 108.23, 108.09, 101.30, 84.00, 62.92, 47.97, 25.01. (C-B) Carbon signal was not observed due to quadrupolar relaxation.[3]

^{11}**B NMR** (128 MHz, $CDCl_3$) δ: 32.8, 11.9.

HRMS (ESI) Calcd for $[C_{20}H_{25}B_2NO_8+H]^+$ $[M+H]^+$: m/z 430.1846, found 430.1858.

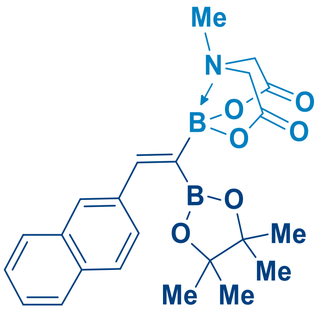

3h

***(E)-6-methyl-2-(2-(naphthalen-2-yl)-1-(4,4,5,5-tetramethyl-1,3,2-dioxaborolan-2-yl)vinyl)-1,3,6,2-dioxazaborocane-4,8-dione (3h)*:**

Prepared according to general procedure J, product **3h** was isolated in (29.3 mg, 64% yield), *E:Z = 99:01*, as a white solid.

^{1}**H NMR** (400 MHZ, $CDCl_3$) δ: 7.86 (s, 1H), 7.80 - 7.73 (m, 3H), 7.69 (s, 1H), 7.56 (d, *J* = 8.10 Hz, 1H), 7.45 - 7.43 (m, 2H), 4.02 (d, *J* = 16.70 Hz, 2H), 395 (d, *J* = 16.70 Hz, 2H), 2.91 (s, 3H), 1.25 (s, 12H).

^{13}**C NMR** (101 MHz, $CDCl_3$) δ: 168.30, 152.31, 137.04, 133.35, 133.28, 128.25, 127.86, 127.77, 127.74, 126.39, 126.33, 84.06, 62.92, 48.03, 25.04. (C-B) Carbon signal was not observed due to quadrupolar relaxation.[3]

^{11}**B NMR** (128 MHz, $CDCl_3$) δ: 32.8, 11.9.

HRMS (ESI) Calcd for $[C_{13}H_{21}B_2NO_6+H]^+$ $[M+H]^+$: m/z 458.1922, found 458.1929.

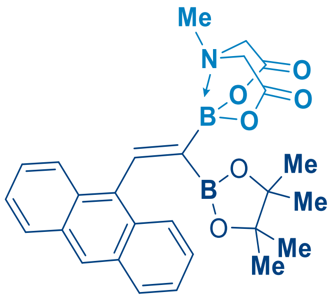

3i

***(E)-2-(2-(anthracen-9-yl)-1-(4,4,5,5-tetramethyl-1,3,2-dioxaborolan-2-yl)vinyl)-6-methyl-1,3,6,2-dioxazaborocane-4,8-dione (3i)*:**

Prepared according to general procedure J, product **3i** was isolated in (36.4 mg, 75% yield), *E:Z = 99:01*, as a white solid.

^{1}H NMR (400 MHZ, $CDCl_3$) δ: 8.38 (s, 1H), 8.33 (s, 1H), 8.09 (d, *J* = 9.01 Hz, 2H), 7.96 (d, *J* = 8.13 Hz, 2H), 7.43 - 7.36 (m, 4H), 4.08 (d, *J* = 16.37 Hz, 2H), 4.02 (d, *J* = 16.37 Hz, 2H), 3.19 (s, 3H), 0.59 (s, 12H).

^{13}C NMR (101 MHz, $CDCl_3$) δ: 167.81, 151.96, 131.37, 129.21, 129.02, 128.66, 126.32, 126.25, 126.09, 125.18, 125.05, 84.09, 83.20, 63.18, 62.71, 47.81, 25.04, 24.31. (C-B) Carbon signal was not observed due to quadrupolar relaxation.[3]

^{11}B NMR (128 MHz, $CDCl_3$) δ: 34.2, 13.2.

HRMS (ESI) Calcd for $[C_{27}H_{29}B_2NO_6+H]^+$ $[M+H]^+$: m/z 486.2263, found 486.2266.

Note: The relative configuration of **3i** was determined by 2D-NMR NOESY (see Supplementary Figure 315, page 255).

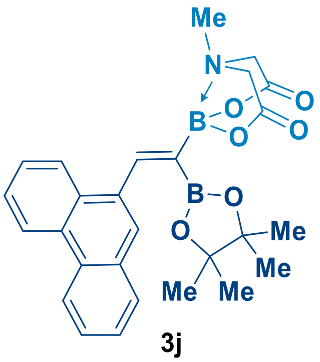

3j

(E)-6-methyl-2-(2-(phenanthren-9-yl)-1-(4,4,5,5-tetramethyl-1,3,2-dioxaborolan-2-yl)vinyl)-1,3,6,2-dioxazaborocane-4,8-dione (3j)**:**

Prepared according to general procedure J, product **3j** was isolated in (25.2 mg, 52% yield), *E:Z = 99:01*, as a white solid.

^{1}H NMR (400 MHZ, $CDCl_3$) δ: 8.68 (dd, *J* = 13.09, 8.11 Hz, 2H), 8.24 (s, 1H), 8.14 (dd, *J* = 7.88, 1.51 Hz, 1H), 7.78 (dd, *J* = 7.82, 1.33 Hz, 1H), 7.69 - 7.54 (m, 5H), 4.04 (d, *J* = 16.22 Hz, 2H), 3.88 (d, *J* = 16.22 Hz, 2H), 3.04 (s, 3H), 1.10 (s, 12H).

^{13}C NMR (101 MHz, $CDCl_3$) δ: 167.77, 151.52, 136.81, 131.40, 130.82, 130.78, 130.30, 128.47, 126.99, 126.85, 126.82, 126.76, 126.22, 125.96, 122.96, 122.85, 83.87, 62.99, 47.90, 24.86. (C-B) Carbon signal was not observed due to quadrupolar relaxation.[3]

^{11}B NMR (128 MHz, $CDCl_3$) δ: 36.8, 13.7.

Note: HRMS was not accurate due to decomposition and solubility issues.

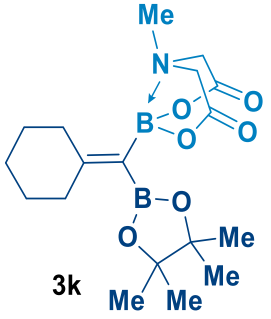

3k

2-(cyclohexylidene(4,4,5,5-tetramethyl-1,3,2-dioxaborolan-2-yl)methyl)-6-methyl-1,3,6,2-dioxazaborocane-4,8-dione (3k)**:**

Prepared according to general procedure J, product **3k** was isolated in (23.4 mg, 62% yield) as a white solid.

^{1}H NMR (400 MHZ, $CDCl_3$) δ: 3.88 (d, *J* = 16.47 Hz, 2H), 3.81 (d, *J* = 16.47 Hz, 2H), 2.88 (s, 3H), 2.35 - 2.30 (m, 4H), 1.64 - 1.55 (m, 6H), 1.24 (s, 12H).

^{13}C NMR (101 MHz, $CDCl_3$) δ: 168.17, 167.74, 83.55, 63.04, 47.54, 39.36, 35.01, 29.22, 28.87, 26.62, 25.07. (C-B) Carbon signal was not observed due to quadrupolar relaxation.[3]

^{11}B NMR (128 MHz, $CDCl_3$) δ: 31.0, 10.1.

HRMS (ESI) Calcd for $[C_{18}H_{29}B_2NO_6+H]^+$ $[M+H]^+$: m/z 378.2260, found 378.2267.

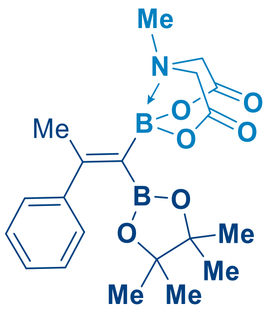

3l

***(E)-6-methyl-2-(2-phenyl-1-(4,4,5,5-tetramethyl-1,3,2-dioxaborolan-2-yl)prop-1-en-1-yl)-1,3,6,2-dioxazaborocane-4,8-dione (3l)*:**

Prepared according to general procedure J, product **3l** was isolated in (27 mg, 64% yield), *E:Z = 99:01*, as a white solid.

^{1}H NMR (400 MHZ, $CDCl_3$) δ: 7.57 (s, 1H), 7.26 - 7.20 (m, 4H), 3.96 (d, *J* = 16.22 Hz, 2H), 3.81 (d, *J* = 16.25 Hz, 2H), 3.03 (s, 3H), 2.24 (s, 3H), 1.26 (s, 12H).

^{13}C NMR (101 MHz, $CDCl_3$) δ: 167.63, 162.54, 148.46, 128.09, 127.32, 127.02, 83.56, 63.12, 47.26, 24.94. (C-B) Carbon signal was not observed due to quadrupolar relaxation.[3]

^{11}B NMR (128 MHz, $CDCl_3$) δ: 32.4, 12.1.

HRMS (ESI) Calcd for $[C_{20}H_{27}B_2NO_6+Na]^+$ $[M+Na]^+$: m/z 422.1922, found 422.1929.

Note: The relative configuration of **3l** was determined by 2D-NMR NOESY (see Supplementary Figure 316, page 255).

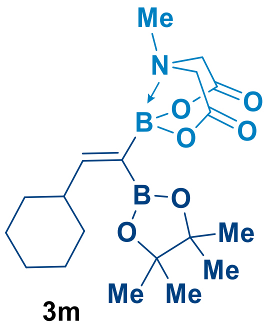

3m

***(E)-2-(2-cyclohexyl-1-(4,4,5,5-tetramethyl-1,3,2-dioxaborolan-2-yl)vinyl)-6-methyl-1,3,6,2-dioxazaborocane-4,8-dione (3m)*:**

Prepared according to general procedure J, product **3m** was isolated in (30.2 mg, 73% yield), *E:Z = 90:10*, as a white solid.

^{1}H NMR (400 MHZ, $CDCl_3$) δ: 6.65 (d, *J* = 9.08 Hz, 1H), 3.93 (d, *J* = 16.07 Hz, 2H), 3.75 (d, *J* = 16.07 Hz, 2H), 2.77 (s, 3H), 2.66 - 2.56 (m, 1H), 1.73 - 1.61 (m, 6H), 1.32 - 1.27 (m, 1H), 1.25 (s, 12H), 1.19 - 1.04 (m, 3H).

^{13}C NMR (101 MHz, $CDCl_3$) δ: 167.83, 166.14, 83.37, 63.11, 47.52, 42.62, 33.36, 26.07, 25.93, 24.90, 24.80. (C-B) Carbon signal was not observed due to quadrupolar relaxation.[3]

^{11}B NMR (128 MHz, $CDCl_3$) δ: 31.8, 12.0.

HRMS (ESI) Calcd for $[C_{19}H_{31}B_2NO_6+Na]^+$ $[M+Na]^+$: m/z 414.2235, found 414.2243.

Note: The relative configuration of **3m** was determined by 2D-NMR NOESY (see Supplementary Figures 317-318, page 256).

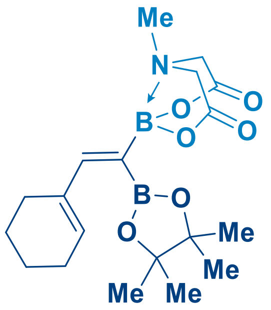

3n

***(E)-2-(2-(cyclohex-1-en-1-yl)-1-(4,4,5,5-tetramethyl-1,3,2-dioxaborolan-2-yl)vinyl)-6-methyl-1,3,6,2-dioxazaborocane-4,8-dione (3n)*:**

Prepared according to general procedure J, product **3n** was isolated in (22.7 mg, 58% yield), *E:Z = 99:01*, as a white a solid.

^{1}H NMR (400 MHZ, $CDCl_3$) δ: 6.93 (s, 1H), 5.90 (s, 1H), 3.90 (d, J = 16.20 Hz, 2H), 3.77 (d, J = 16.20 Hz, 2H), 2.82 (s, 3H), 2.23 (brs, 1H), 2.14 (brs, 1H), 1.64 - 1.58 (m, 6H), 1.26 (s, 12H).

^{13}C NMR (101 MHz, $CDCl_3$) δ: 167.81, 155.10, 138.92, 133.56, 83.88, 62.80, 47.87, 26.86, 26.21, 25.16, 25.01, 24.73, 22.64, 22.20. (C-B) Carbon signal was not observed due to quadrupolar relaxation.[3]

^{11}B NMR (128 MHz, $CDCl_3$) δ: 32.9, 12.0.

HRMS (ESI) Calcd for $[C_{19}H_{29}B_2NO_6+H]^+$ $[M+H]^+$: m/z 392.2315, found 392.2319.

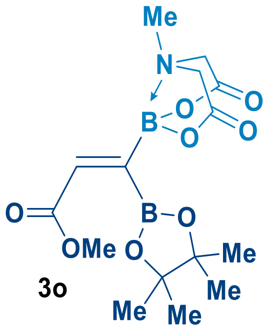

3o

***methyl (E)-3-(6-methyl-4,8-dioxo-1,3,6,2-dioxazaborocan-2-yl)-3-(4,4,5,5-tetramethyl-1,3,2-dioxaborolan-2-yl)acrylate (3o)*:**

Prepared according to general procedure J, product **3o** was isolated in (26.8 mg, 73% yield), *E:Z = 99:01*, as a white solid.

^{1}H NMR (400 MHZ, $CDCl_3$) δ: 6.56 (s, 1H), 4.05 (d, J = 16.78 Hz, 2H), 3.81 (d, J = 16.78 Hz, 2H), 3.71 (s, 3H), 2.82 (s, 3H), 1.28 (s, 12H).

^{13}C NMR (101 MHz, $CDCl_3$) δ: 168.32, 168.30, 167.26, 137.81, 84.35, 62.48, 51.94, 47.90, 25.08. (C-B) Carbon signal was not observed due to quadrupolar relaxation.[3]

^{11}B NMR (128 MHz, $CDCl_3$) δ: 35.0, 10.2.

HRMS (ESI) Calcd for $[C_{15}H_{23}B_2NO_8+H]^+$ $[M+H]^+$: m/z 368.1688, found 368.1683.

Note: The relative configuration of **3o** was determined by 2D-NMR NOESY (see Supplementary Figure 319, page 257).

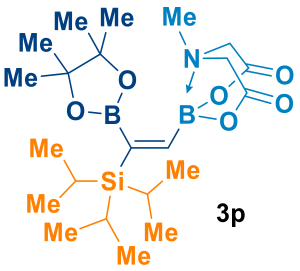

***(E)-6-methyl-2-(2-(4,4,5,5-tetramethyl-1,3,2-dioxaborolan-2-yl)-2-(triisopropylsilyl)vinyl)-1,3,6,2-dioxazaborocane-4,8-dione (3p)*:**

Prepared according to general procedure J, product **3p** was isolated in (35.4 mg, 76% yield), *r.r. = 99:01*, as a white solid.

^{1}H NMR (400 MHZ, $CDCl_3$) δ: 6.59 (s, 1H), 3.80 (s, 4H), 2.97 (s, 3H), 1.25 (s, 12H), 1.24 - 1.20 (m, 3H), 1.05 (d, *J* = 7.28 Hz, 18H).

^{13}C NMR (101 MHz, $CDCl_3$) δ: 167.55, 83.70, 63.04, 48.43, 25.36, 19.01, 11.58. (C-B) Carbon signal was not observed due to quadrupolar relaxation.[3]

^{11}B NMR (128 MHz, $CDCl_3$) δ: 31.9, 9.5.

HRMS (ESI) Calcd for $[C_{22}H_{41}B_2NO_6Si+H]^+$ $[M+H]^+$: m/z 466.2971, found 466.2971.

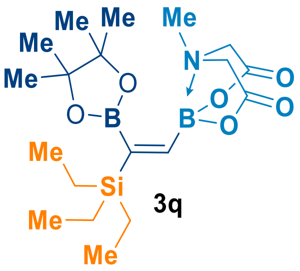

***(E)-6-methyl-2-(2-(4,4,5,5-tetramethyl-1,3,2-dioxaborolan-2-yl)-2-(triethylsilyl)vinyl)-1,3,6,2-dioxazaborocane-4,8-dione (3q)*:**

Prepared according to general procedure J, product **3q** was isolated in (33.5 mg, 79% yield), *r.r. = 99:01*, as a white solid.

^{1}H NMR (400 MHZ, $CDCl_3$) δ: 6.55 (s, 1H), 3.83 (d, *J* = 17.34 Hz, 2H), 3.78 (d, *J* = 17.34 Hz, 2H), 2.91 (s, 3H), 1.27 (s, 12H), 0.91 (t, *J* = 7.87 Hz, 9H), 0.63 (q, *J* = 7.86 Hz, 6H),

^{13}C NMR (101 MHz, $CDCl_3$) δ: 167.72, 83.70, 62.92, 48.25, 25.22, 7.62, 3.40. (C-B) Carbon signal was not observed due to quadrupolar relaxation.[3]

^{11}B NMR (128 MHz, $CDCl_3$) δ: 29.8, 9.9.

HRMS (ESI) Calcd for $[C_{19}H_{35}B_2NO_6Si+H]^+$ $[M+H]^+$: m/z 424.2500, found 424.2504.

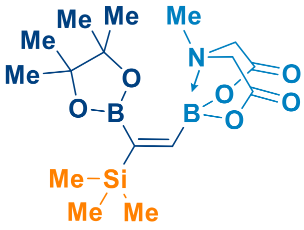

3r

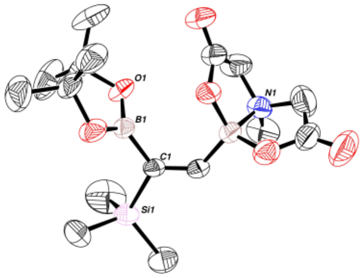

X-ray structure of 3r CCDC: 2194039

***(E)-6-methyl-2-(2-(4,4,5,5-tetramethyl-1,3,2-dioxaborolan-2-yl)-2-(trimethylsilyl)vinyl)-1,3,6,2-dioxazaborocane-4,8-dione (3r)*:**

Prepared according to general procedure J, product **3r** was isolated in (30.9 mg, 81% yield), *r.r. = 99:01*, as a white solid.

^{1}H NMR (400 MHZ, $CDCl_3$) δ: 6.58 (s, 1H), 3.85 (d, J = 16.37 Hz, 2H), 3.75 (d, J = 16.37 Hz, 2H), 2.89 (s, 3H), 1.27 (s, 12H), 0.10 (s, 9H).

^{13}C NMR (101 MHz, $CDCl_3$) δ: 167.81, 83.78, 62.81, 48.11, 25.25, -0.94. (C-B) Carbon signal was not observed due to quadrupolar relaxation.[3]

^{11}B NMR (128 MHz, $CDCl_3$) δ: 33.2, 10.9.

HRMS (ESI) Calcd for $[C_{16}H_{29}B_2NO_6Si+H]^+$ $[M+H]^+$: m/z 382.2030, found 382.2030.

Notes: (1) The structure of **3r** was confirmed by X-ray crystallographic analysis, CCDC 2194039 (see Supplementary Table 8, page 95). (2) Compound **3r** was recrystallized by using chloroform as a solvent.

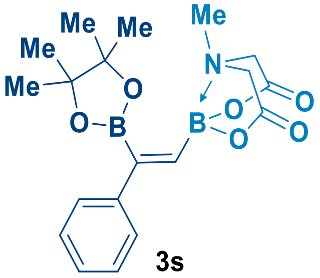

3s

***(E)-6-methyl-2-(2-phenyl-2-(4,4,5,5-tetramethyl-1,3,2-dioxaborolan-2-yl)vinyl)-1,3,6,2-dioxazaborocane-4,8-dione (3s)*:**

Prepared according to general procedure J, product **3s** was isolated in (31.2 mg, 81% yield), *E:Z = 99:01* and *s.s. = 99:01*, as a white solid.

^{1}H NMR (400 MHZ, $CDCl_3$) δ: 7.40 - 7.37 (m, 2H), 7.33 - 7.27 (m, 3H), 6.25 (s, 1H), 3.81 (s, 4H), 2.98 (s, 3H), 1.35 (s, 12H).

^{13}C NMR (101 MHz, $CDCl_3$) δ: 168.05, 144.00, 128.51, 127.49, 126.79, 84.51, 62.80, 48.28, 25.12. (C-B) Carbon signal was not observed due to quadrupolar relaxation.[3]

^{11}B NMR (128 MHz, $CDCl_3$) δ: 33.2, 12.3.

HRMS (ESI) Calcd for $[C_{19}H_{25}B_2NO_6+H]^+$ $[M+H]^+$: m/z 386.1948, found 386.1945.

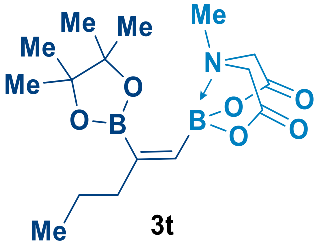

3t

***(E)-6-methyl-2-(2-(4,4,5,5-tetramethyl-1,3,2-dioxaborolan-2-yl)pent-1-en-1-yl)-1,3,6,2-dioxazaborocane-4,8-dione (3t)*:**

Prepared according to general procedure J, product **3t** was isolated in (16.2 mg, 46% yield), *E:Z = 99:01* and *s.s. = 95:05*, as a white solid.

^{1}H NMR (400 MHZ, $CDCl_3$) δ: 5.84 (s, 1H), 3.82 (d, J = 16.90 Hz, 2H), 3.79 (d, J = 16.90 Hz, 2H), 2.94 (s, 3H), 2.25 (t, J = 7.46 Hz, 2H), 1.43 (quin, J = 7.44 Hz, 2H), 1.27 (s, 12H),

0.88 (t, J = 7.36 Hz, 3H).

^{13}C NMR (101 MHz, $CDCl_3$) δ: 167.66, 83.99, 63.12, 48.38, 43.07, 24.88, 22.58, 14.08. (C-B) Carbon signal was not observed due to quadrupolar relaxation.[3]

^{11}B NMR (128 MHz, $CDCl_3$) δ: 32.3, 12.5.

HRMS (ESI) Calcd for $[C_{16}H_{27}B_2NO_6+H]^+$ $[M+H]^+$: m/z 352.2103, found 352.2110.

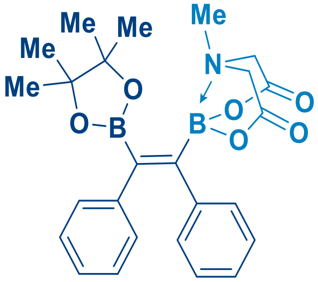

3u

***(Z)-2-(1,2-diphenyl-2-(4,4,5,5-tetramethyl-1,3,2-dioxaborolan-2-yl)vinyl)-6-methyl-1,3,6,2-dioxazaborocane-4,8-dione (3u)*:**

Prepared according to general procedure J, product **3u** was isolated in (34.6 mg, 75% yield), *E:Z = 01:99*, as a white solid.

^{1}H NMR (400 MHZ, $CDCl_3$) δ: 7.09 - 6.86 (m, 10H), 3.66 (d, J = 16.21 Hz, 2H), 3.25 (d, J = 16.18 Hz, 2H), 3.04 (s, 3H), 1.33 (s, 12H).

^{13}C NMR (101 MHz, $CDCl_3$) δ: 167.23, 142.28, 141.87, 130.28, 128.73, 128.15, 127.79, 126.11, 125.90, 84.25, 62.59, 46.61, 25.33. (C-B) Carbon signal was not observed due to quadrupolar relaxation.[3]

^{11}B NMR (128 MHz, $CDCl_3$) δ: 31.2, 11.4.

HRMS (ESI) Calcd for $[C_{25}H_{29}B_2NO_6+H]^+$ $[M+H]^+$: m/z 462.2263, found 462.2274.

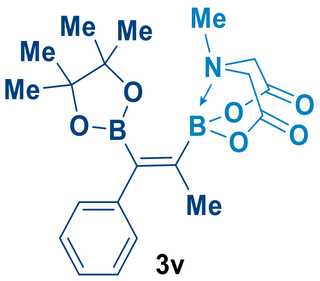

3v

***(Z)-6-methyl-2-(1-phenyl-1-(4,4,5,5-tetramethyl-1,3,2-dioxaborolan-2-yl)prop-1-en-2-yl)-1,3,6,2-dioxazaborocane-4,8-dione (3v)*:**

Prepared according to general procedure J, product **3v** was isolated in (25.6 mg, 64% yield), *E:Z = 01:99* and *s.s. = 99:01*, as a white solid.

^{1}H NMR (400 MHZ, CD_3CN) δ: 7.37 - 7.30 (m, 2H), 7.21 (tt, *J* = 7.50, 1.32 Hz, 2H), 7.06-7.03 (m, 2H), 3.97 (d, *J* = 17.22 Hz, 2H), 3.90 (d, *J* = 17.22 Hz, 2H), 2.98 (s, 3H), 1.51 (s, 3H), 1.18 (s, 12H).

^{13}C NMR (101 MHz, CD_3CN) δ: 169.70, 144.45, 129.11, 129.10, 128.93, 126.84, 84.44, 64.13, 48.73, 25.17, 18.99. (C-B) Carbon signal was not observed due to quadrupolar relaxation.[3]

^{11}B NMR (128 MHz, $CDCl_3$) δ: 31.6, 12.0.

HRMS (ESI) Calcd for $[C_{20}H_{27}B_2NO_6+H]^+$ $[M+H]^+$: m/z 400.2104, found 400.2104.

Note: The relative configuration of **3v** was determined by 2D-NMR NOESY (see Supplementary Figure 320, page 257).

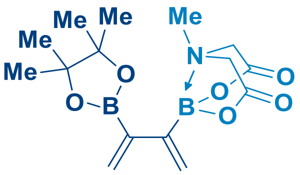

3w

***6-methyl-2-(3-(4,4,5,5-tetramethyl-1,3,2-dioxaborolan-2-yl)buta-1,3-dien-2-yl)-1,3,6,2-dioxazaborocane-4,8-dione (3w)*:**

Prepared according to general procedure J, product **3w** was isolated in (15.4 mg, 46% yield) as a white solid.

^{1}H NMR (400 MHZ, $CDCl_3$) δ: 5.76 (q, *J* = 3.60 Hz, 2H), 5.62 (d, *J* = 3.01 Hz, 1H), 5.45 (d, *J* = 3.05 Hz, 1H), 3.91 (d, *J* = 16.54 Hz, 2H), 3.77 (d, *J* = 16.54 Hz, 2H), 2.92 (s, 3H), 1.26 (s, 12H).

^{13}**C NMR** (101 MHz, $CDCl_3$) δ: 167.93, 130.44, 126.27, 84.03, 62.89, 47.91, 24.88. (C-B) Carbon signal was not observed due to quadrupolar relaxation.[3]

^{11}**B NMR** (128 MHz, $CDCl_3$) δ: 31.2, 11.3.

HRMS (ESI) Calcd for $[C_{15}H_{23}B_2NO_6+H]^+$ $[M+H]^+$: m/z 336.1790, found 336.1792.

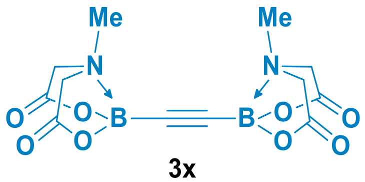

3x

***2,2'-(ethyne-1,2-diyl)bis(6-methyl-1,3,6,2-dioxazaborocane-4,8-dione) (3x)*:**

Prepared according to general procedure J, product **3x** was isolated in (27.3 mg, 76% yield) as a white solid.

^{1}**H NMR** (400 MHZ, CD_3CN) δ: 4.01 (dd, *J* = 17.18, 2.35 Hz, 4H), 3.89 (dd, *J* = 17.18, 2.35 Hz, 4H), 3.03 (s, 6H).

^{13}**C NMR** (101 MHz, CD_3CN) δ: 168.76, 62.43, 48.89, 48.78. (C-B) Carbon signal was not observed due to quadrupolar relaxation.[3]

^{11}**B NMR** (128 MHz, $CDCl_3$) δ: 5.7.

HRMS (ESI) Calcd for $[C_{12}H_{14}B_2N_2O_8+Na]^+$ $[M+Na]^+$: m/z 359.0840, found 359.0834.

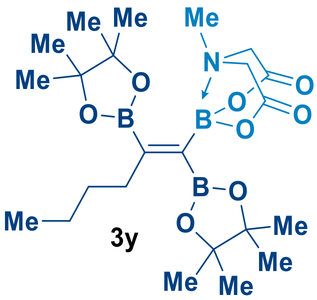

3y

***(E)-2-(1,2-bis(4,4,5,5-tetramethyl-1,3,2-dioxaborolan-2-yl)hex-1-en-1-yl)-6-methyl-1,3,6,2-dioxazaborocane-4,8-dione (3y)*:**

Prepared according to general procedure J, product **3y** was isolated in (24.1 mg, 49% yield), *E:Z = 99:01* and *r.r.* = *99:01*, as a white solid.

^{1}H NMR (400 MHZ, $CDCl_3$) δ: 3.90 (d, *J* = 16.15 Hz, 2H), 3.74 (d, *J* = 16.15 Hz, 2H), 3.02 (s, 3H), 2.47 (t, *J* = 15.09 Hz, 2H), 1.40 - 1.30 (m, 4H), 1.27 (s, 12H), 1.24 (s, 12H), 0.87 (t, *J* = 7.19 Hz, 3H).

^{13}C NMR (101 MHz, $CDCl_3$) δ: 168.31, 83.74, 83.52, 64.09, 48.95, 38.81, 32.63, 25.03, 22.93, 14.16. (C-B) Carbon signal was not observed due to quadrupolar relaxation.[3]

^{11}B NMR (128 MHz, $CDCl_3$) δ: 32.9, 12.0.

HRMS (ESI) Calcd for $[C_{23}H_{40}B_3NO_8+H]^+$ $[M+H]^+$: m/z 492.3118, found 492.3131.

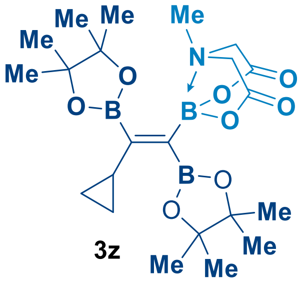

3z

***(E)-2-(2-cyclopropyl-1,2-bis(4,4,5,5-tetramethyl-1,3,2-dioxaborolan-2-yl)vinyl)-6-methyl-1,3,6,2-dioxazaborocane-4,8-dione (3z)*:**

Prepared according to general procedure J, product **3z** was isolated in (22.8 mg, 48% yield), *E:Z = 99:01* and *r.r.* = *99:01*, as a white solid.

^{1}H NMR (400 MHZ, $CDCl_3$) δ: 3.92 (d, *J* = 16.39 Hz, 2H), 3.68 (d, *J* = 16.34 Hz, 2H), 3.01 (s, 3H), 2.01 (quin, *J* = 7.07 Hz, 1H), 1.27 (s, 12H), 1.25 (s, 12H), 0.72 (d, *J* = 7.82 Hz, 4H),

^{13}C NMR (101 MHz, $CDCl_3$) δ: 168.21, 83.92, 83.54, 63.98, 48.67, 25.59, 25.07, 7.40. (C-B) Carbon signal was not observed due to quadrupolar relaxation.[3]

^{11}B NMR (128 MHz, $CDCl_3$) δ: 31.8, 11.8.

HRMS (ESI) Calcd for $[C_{22}H_{36}B_3NO_8+H]^+$ $[M+H]^+$: m/z 476.2805, found 476.2803.

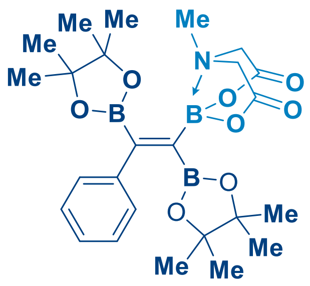

3aa

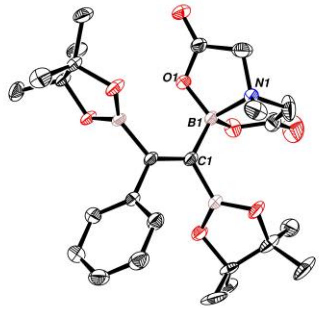

X-ray structure of 3aa
CCDC : 2194040

***(E)-6-methyl-2-(2-phenyl-1,2-bis(4,4,5,5-tetramethyl-1,3,2-dioxaborolan-2-yl)vinyl)-1,3,6,2-dioxazaborocane-4,8-dione (3aa)*:**

Prepared according to general procedure J, product **3aa** was isolated in (35.8 mg, 70% yield), *E:Z = 99:01* and *r.r. = 99:01*, as a white solid.

^{1}H NMR (400 MHZ, CD_3CN) δ: 7.30 - 7.21 (m, 5H), 3.98 (d, J = 17.10 Hz, 2H), 3.95 (d, J = 17.10 Hz, 2H), 3.02 (s, 3H), 1.22 (s, 12H), 1.03 (s, 12H).

^{13}C NMR (101 MHz, CD_3CN) δ: 169.78, 128.79, 128.25, 127.77, 84.73, 84.31, 64.18, 49.32, 25.06. (C-B) Carbon signal was not observed due to quadrupolar relaxation.[3]

^{11}B NMR (128 MHz, CD_3CN) δ: 31.5, 11.3.

HRMS (ESI) Calcd for $[C_{25}H_{36}B_3NO_8+H]^+$ $[M+H]^+$: m/z 512.2806, found 512.2814.

NoteS: (1) The structure of **3aa** was confirmed by X-ray crystallographic analysis, CCDC 2194040 (see Supplementary Table 9, page 96). (2) Compound **3aa** was recrystallized by using EtOAc as a solvent.

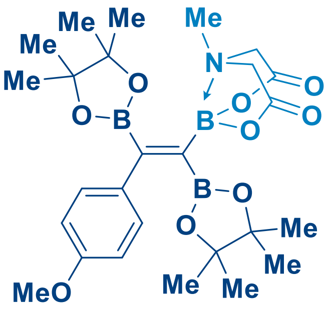

3ab

***(E)-2-(2-(4-methoxyphenyl)-1,2-bis(4,4,5,5-tetramethyl-1,3,2-dioxaborolan-2-yl)vinyl)-6-methyl-1,3,6,2-dioxazaborocane-4,8-dione (3ab)*:**

Prepared according to general procedure J, product **3ab** was isolated in (39 mg, 72% yield), *E:Z = 99:01* and *r.r.* = *99:01*, as a white solid.

^{1}H NMR (400 MHZ, $CDCl_3$) δ: 7.27 (dt, *J* = 8.79, 2.08 Hz, 2H), 6.78 (dt, *J* = 8.79, 2.92 Hz, 2H), 3.95 (d, *J* = 16.77 Hz, 2H), 3.80 (d, *J* = 16.77 Hz, 2H), 3.10 (s, 3H), 1.24 (s, 12H), 1.08 (s, 12H)

^{13}C NMR (101 MHz, $CDCl_3$) δ: 168.20, 158.94, 137.98, 128.80, 113.27, 83.99, 83.64, 63.66, 55.39, 48.71, 24.97. (C-B) Carbon signal was not observed due to quadrupolar relaxation.[3]

^{11}B NMR (128 MHz, $CDCl_3$) δ: 31.9, 11.7.

HRMS (ESI) Calcd for $[C_{26}H_{38}B_3NO_9+H]^+$ $[M+H]^+$: m/z 542.2912, found 542.2906.

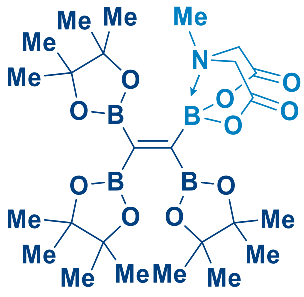

4

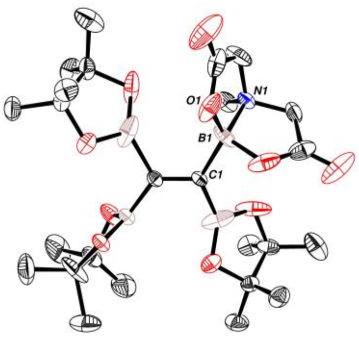

X-ray structure of 4 CCDC: 2194041

***6-methyl-2-(1,2,2-tris(4,4,5,5-tetramethyl-1,3,2-dioxaborolan-2-yl)vinyl)-1,3,6,2-dioxazaborocane-4,8-dione (4)*:**

Prepared according to general procedure J, product **4** was isolated in (40.3 mg, 69% yield) as a white solid.

^{1}H NMR (400 MHZ, $CDCl_3$) δ: 3.90 (d, *J* = 16.21 Hz, 2H), 3.72 (d, *J* = 16.28 Hz, 2H), 2.96 (s, 3H), 1.29 (s, 12H), 1.27 (s, 12H), 1.24 (s, 12H).

^{13}C NMR (101 MHz, $CDCl_3$) δ: 168.11, 83.96, 83.77, 83.75, 63.95, 48.72, 24.99. (C-B) Carbon signal was not observed due to quadrupolar relaxation.[3]

^{11}B NMR (128 MHz, $CDCl_3$) δ: 31.6, 10.9.

HRMS (ESI) Calcd for $[C_{25}H_{43}B_2NO_{10}+Na]^+$ $[M+Na]^+$: m/z 584.3108, found 584.3169.

Notes: (1) The structure of **4** was confirmed by X-ray crystallographic analysis, CCDC 2194041 (see Supplementary Table 11, page 98). (2) Compound **4** was recrystallized by using EtOAc as a solvent.

2.6. General Procedure K and Characterization for Products (5a, 5b and 5c)

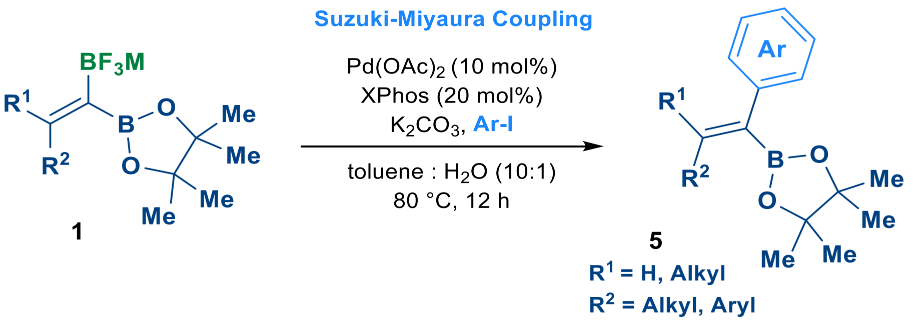

Inside the glove box, into a 10 ml vial equipped with a magnetic stirring bar, $Pd(OAc)_2$ (0.01 mmol, 10 mol%), XPhos (0.02 mmol, 20 mol%) and trifluoroborate salt (**1**) (0.12 mmol, 1.2 equiv) were dissolve with toluene and stirred for 10 min. After that, aryl iodide (0.1 mmol, 1 equiv) and base (0.3 mmol, 3 equiv) were added and the vial was sealed and taken out of the glovebox. 0.1 ml of H_2O was added under nitrogen (N_2) and the reaction was heated on an oil bath for 12 h. After completion, the reaction was diluted with H_2O (5 ml), and extracted with EtOAc (2 x 5 ml). The organic phase was dried over magnesium sulfate and concentrated under reduced pressure (by evaporator) to give the crude material, which was purified by column chromatography on silica gel to give products (**5**).

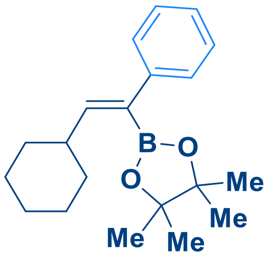

5a

***(E)-2-(2-cyclohexyl-1-phenylvinyl)-4,4,5,5-tetramethyl-1,3,2-dioxaborolane (5a)*:**

Prepared according to general procedure K, product **5a** was isolated in (32.2 mg, 86% yield), *E:Z = 99:01*, as a colorless oil.

R_f = 0.53 (10% EtOAc in hexane).

¹H NMR (400 MHZ, $CDCl_3$) δ: 7.33 - 7.26 (m, 3H), 7.25 - 7.24 (m, 1H), 7.19 - 7.15 (m, 1H), 6.22 (d, *J* = 9.4 Hz, 1H), 2.64 - 2.55 (m, 1H), 1.77 - 1.65 (m, 5H), 1.34 (s, 12H), 1.33 - 1.26 (m, 2H), 1.23 - 1.10 (3H).

¹³C NMR (101 MHz, $CDCl_3$) δ: 152.24, 143.09, 128.25, 127.16, 126.23, 83.58, 40.84, 33.67, 26.14, 26.05, 24.96. (C-B) Carbon signal was not observed due to quadrupolar relaxation.[3]

¹¹B NMR (128 MHz, $CDCl_3$) δ: 31.3.

Note: The relative configuration of **5a** was determined by 2D-NMR NOESY (see Supplementary Figure 321, page 258).

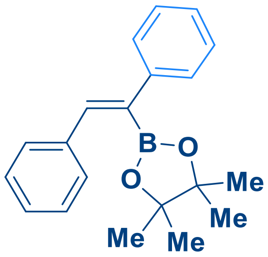

5b

***(E)-2-(1,2-diphenylvinyl)-4,4,5,5-tetramethyl-1,3,2-dioxaborolane (5b)*:**

Prepared according to general procedure K, product **5b** was isolated in (33.4 mg, 91% yield), *E:Z = 99:01*, as a colorless oil.

R_f = 0.57 (10% EtOAc in hexane).

¹H NMR (400 MHZ, $CDCl_3$) δ: 7.49 - 7.44 (m, 4H), 7.37 - 7.31 (m, 4H), 7.29 - 7.24 (m, 3H), 1.33 (s, 12H).

¹³C NMR (101 MHz, $CDCl_3$) δ: 142.76, 140.93, 138.94, 128.59, 128.40, 128.24, 127.71, 127.10, 127.03, 84.21, 25.03. (C-B) Carbon signal was not observed due to quadrupolar relaxation.[3]

¹¹B NMR (128 MHz, $CDCl_3$) δ: 31.8.

The spectral data are consistent with those reported in the literature.[13]

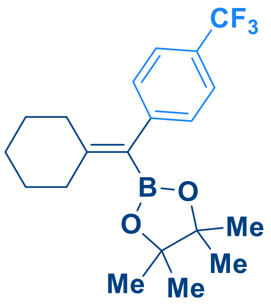

5c

***2-(cyclohexylidene(4-(trifluoromethyl)phenyl)methyl)-4,4,5,5-tetramethyl-1,3,2-dioxaborolane (5c)*:**

Prepared according to general procedure K, product **5c** was isolated in (32 mg, 73% yield) as a colorless oil.

R_f = 0.55 (10% EtOAc in hexane).

^{1}H NMR (400 MHZ, $CDCl_3$) δ: 7.25 (d, *J* = 8.1 Hz, 2H), 7.18 (d, *J* = 8.1 Hz, 2H), 2.56 - 2.53 (m, 2H), 2.07 - 2.04 (m, 2H), 1.71 - 1.66 (m, 2H), 1.61 - 1.57 (m, 2H), 1.52 - 1.48 (m, 2H), 1.24 (s, 12H).

^{13}C NMR (101 MHz, $CDCl_3$) δ: 157.30, 146.55, 129.51, 127.44, 124.93, 124.90, 124.87, 124.84, 123.65, 83.51, 35.39, 32.42, 28.99, 28.63, 26.41, 24.80. (C-B) Carbon signal was not observed due to quadrupolar relaxation.[3]

^{11}B NMR (128 MHz, $CDCl_3$) δ: 30.8.

^{19}F NMR (376 MHz, CDCl3) δ: -62.15 (s).

2.7. General Procedure L and Characterization for Products (6a, 6b and 6c)

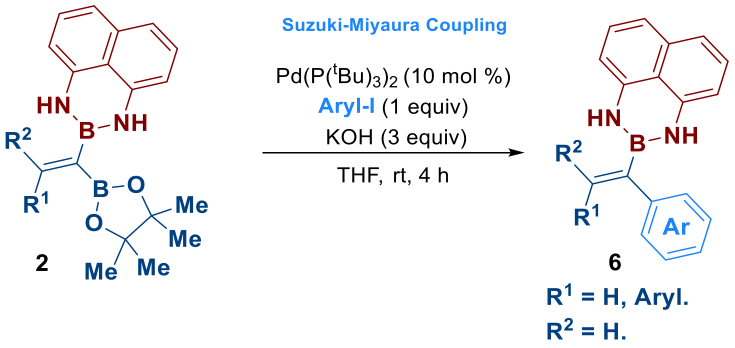

Inside the glove box, into a 10 ml vial equipped with a magnetic stirring bar, Pd(P(tBu)$_3$)$_2$, **2** (0.1 mmol, 1 equiv), aryl iodide (0.1 mmol, 1 equiv) and powdered KOH (0.3 mmol, 3 equiv) were added and dissolved in THF. The vial was sealed and stirred for 4 h. After completion, the reaction was diluted with H_2O (5 ml), and extracted with EtOAc (2 x 5 ml). The organic phase was dried over magnesium sulfate and concentrated under reduced pressure (by evaporator) to give the crude material, which was purified by column chromatography on silica gel to give product (**6**).

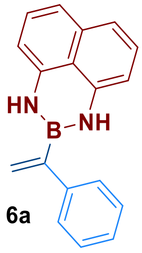

6a

***2-(1-phenylvinyl)-2,3-dihydro-1H-naphtho[1,8-de][1,3,2]diazaborinine (6a)*:**

Prepared according to general procedure L, product **6a** was isolated in (24.5 mg, 91% yield), *E:Z = 99:01*, as a colorless oil.

R_f = 0.51 (10% EtOAc in hexane).

^{1}H NMR (400 MHZ, $CDCl_3$) δ: 7.30 - 7.19 (m, 5H), 7.01 - 6.97 (m, 2H), 6.93 - 6.91 (m, 2H), 6.17 (dd, *J* = 7.3, 1.0 Hz, 2H), 5.7 (d, *J* = 2.1 Hz, 1H), 5.65 - 5.64 (m, 3H).

^{13}C NMR (101 MHz, $CDCl_3$) δ: 142.26, 141.09, 136.45, 128.81, 127.70, 127.63, 127.48, 125.06, 120.02, 117.91, 106.07. (C-B) Carbon signal was not observed due to quadrupolar relaxation.[3]

^{11}B NMR (128 MHz, $CDCl_3$) δ: 29.0.

The spectral data are consistent with those reported in the literature.[14]

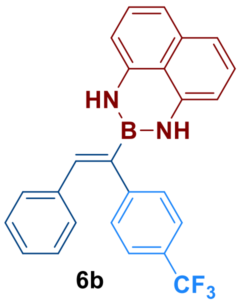

6b

***(Z)-2-(2-phenyl-1-(4-(trifluoromethyl)phenyl)vinyl)-2,3-dihydro-1H-naphtho[1,8-de][1,3,2]diazaborinine (xx)*:**

Prepared according to general procedure L, product **6b** was isolated in (38.6 mg, 93% yield), *E:Z = 99:01*, as a colorless oil.

R_f = 0.38 (10% EtOAc in hexane).

^{1}H NMR (400 MHZ, $CDCl_3$) δ: 7.64 (d, *J* = 8.2 Hz, 2H), 7.29 (d, *J* = 7.4 Hz, 2H), 7.19 - 7.16 (m, 4H), 7.12 - 7.09 (m, 2H), 7.05 - 6.99 (m, 4H), 6.32 (dd, *J* = 7.2, 1.0 Hz, 2H), 5.62 (s, 2H).

^{13}C NMR (101 MHz, $CDCl_3$) δ: 145.11, 140.93, 138.70, 136.45, 136.35, 129.91, 129.33, 128.39, 128.17, 127.73, 126.17, 126.13, 119.91, 118.10, 106.24. (C-B) Carbon signal was not observed due to quadrupolar relaxation.[3]

^{11}B NMR (128 MHz, $CDCl_3$) δ: 28.82.

^{19}F NMR (376 MHz, CDCl3) δ: -62.32 (s).

HRMS (ESI) Calcd for $[C_{25}H_{18}BN_2F_3+H]^+$ $[M+H]^+$: m/z 415.1587, found 415.1587.

Procedure L and Characterization for Product (6c):

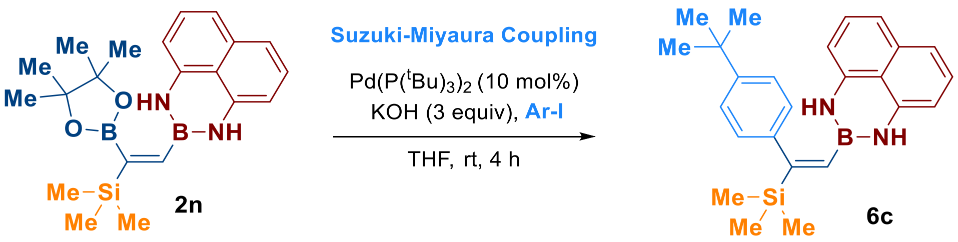

Inside the glove box, into a 10 ml vial equipped with a magnetic stirring bar, Pd(P(^{t}Bu)$_3$)$_2$, **2n** (0.1 mmol, 1 equiv), aryl iodide (0.1 mmol, 1 equiv) and powdered KOH (0.3 mmol, 3 equiv) were added and dissolved in THF. The vial was sealed and stirred for 4 h. After completion, the reaction was diluted with H_2O (5 ml), and extracted with EtOAc (2 x 5 ml). The organic phase was dried over magnesium sulfate and concentrated under reduced pressure (by evaporator) to give the crude material, which was purified by column chromatography on silica gel to give product **6c** in (35.1 mg, 88% yield) as a yellowish solid.

(E)-2-(2-(4-(tert-butyl)phenyl)-2-(trimethylsilyl)vinyl)-2,3-dihydro-1H-naphtho[1,8-de][1,3,2]diazaborinin (6c):

R_f = 0.59 (10% EtOAc in hexane).

^{1}H NMR (400 MHZ, $CDCl_3$) δ: 7.29 - 7.25 (m, 2H), 6.87 - 6.83 (m, 4H), 6.78 - 6.75 (m, 2H), 5.99 (s, 1H), 5.75 (dd, *J* = 7.4, 1.01 Hz, 2H), 4.97 (s, 2H), 1.27 (s, 9H), 0.00 (s, 9H).

^{13}C NMR (101 MHz, $CDCl_3$) δ: 168.20, 149.50, 141.83, 141.37, 136.36, 127.59, 126.42, 125.25, 119.79, 117.20, 105.55, 34.72, 31.67, -1.79. (C-B) Carbon signal was not observed due to quadrupolar relaxation.[3]

^{11}B NMR (128 MHz, $CDCl_3$) δ: 27.2.

HRMS (ESI) Calcd for $[C_{25}H_{31}BN_2Si+H]^+$ $[M+H]^+$: m/z 399.2420, found 399.2422.

2.8. General Procedure M and Characterization for Products (5b, 5d and 5e)

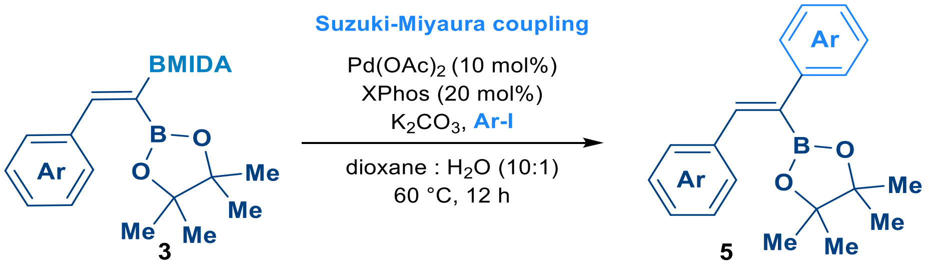

Inside the glove box, into a 10 ml vial equipped with a magnetic stirring bar, $Pd(OAc)_2$ (0.01 mmol, 10 mol%), XPhos (0.02 mmol, 20 mol%) and (**3**) (0.12 mmol, 1.2 equiv) were dissolved with toluene and stirred for 10 min. After that, aryl iodide (0.1 mmol, 1 equiv) and base (0.3 mmol, 3 equiv) were added and the vial was sealed and taken out of the glovebox. 0.1 ml of H_2O was added under nitrogen (N_2) and the reaction was heated on an oil bath for 12 h. After completion, the reaction was diluted with H_2O (5 ml), and extracted with EtOAc (2 x 5 ml). The organic phase was dried over magnesium sulfate and concentrated under reduced pressure (by evaporator) to give the crude material, which was purified by column chromatography on silica gel to give the product (**5**).

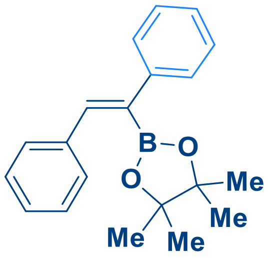

5b

***(E)-2-(1,2-diphenylvinyl)-4,4,5,5-tetramethyl-1,3,2-dioxaborolane (5b)*:**

Prepared according to general procedure M, product **5b** was isolated in (30.1 mg, 82% yield), *E:Z = 99:01*, as a colorless oil.

R_f = 0.37 (10% EtOAc in hexane).

^{1}H NMR (400 MHZ, $CDCl_3$) δ: 7.49 - 4.44 (m, 4H), 7.37 - 7.31 (m, 4H), 7.29 - 7.24 (m, 3H), 1.33 (s, 12H).

^{13}C NMR (101 MHz, $CDCl_3$) δ: 142.76, 140.93, 138.94, 128.59, 128.40, 128.24, 127.71, 127.10, 127.03, 84.21, 25.03. (C-B) Carbon signal was not observed due to quadrupolar relaxation.[3]

^{11}B NMR (128 MHz, $CDCl_3$) δ: 31.8.

The spectral data are consistent with those reported in the literature.[13]

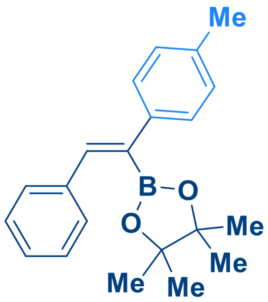

5d

***(E)-4,4,5,5-tetramethyl-2-(2-phenyl-1-(p-tolyl)vinyl)-1,3,2-dioxaborolane (5d)*:**

Prepared according to general procedure M, product **5d** was isolated in (26.6 mg, 69% yield), *E:Z = 99:01*, as a colorless oil.

R_f = 0.56 (10% EtOAc in hexane).

^{1}H NMR (400 MHZ, $CDCl_3$) δ: 7.47 - 7.44 (m, 2H), 7.36 - 7.29 (m, 4H), 7.27 - 7.23 (m, 3H), 7.17 - 7.12 (m, 2H), 2.35 (s, 3H), 1.30 (s, 12H).

^{13}C NMR (101 MHz, $CDCl_3$) δ: 140.13, 139.91, 139.06, 136.74, 129.32, 128.21, 127.57, 126.97, 84.16, 25.04, 21.29. (C-B) Carbon signal was not observed due to quadrupolar relaxation.[3]

^{11}B NMR (128 MHz, $CDCl_3$) δ: 31.3.

HRMS (ESI) Calcd for $[C_{21}H_{25}BO_2+H]^+$ $[M+H]^+$: m/z 321.2021, found 321.2021.

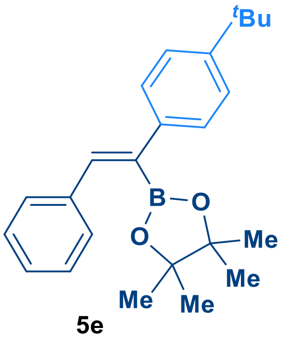

5e

***(E)-2-(1-(4-(tert-butyl)phenyl)-2-phenylvinyl)-4,4,5,5-tetramethyl-1,3,2-dioxaborolane (5e)*:**

Prepared according to general procedure M, product **5e** was isolated in (39.5 mg, 91% yield), *E:Z = 99:01*, as a colorless oil.

R_f = 0.56 (10% EtOAc in hexane).

^{1}H NMR (400 MHZ, $CDCl_3$) δ: 7.47 - 7.44 (m, 2H), 7.41 - 7.36 (m, 3H), 7.35 - 7.33 (m, 1H), 7.32 - 7.29 (m, 2H), 7.27 - 7.26 (m, 1H), 7.26 - 7.23 (m, 1H).

^{13}C NMR (101 MHz, $CDCl_3$) δ: 149.95, 140.22, 139.77, 139.10, 128.40, 128.20, 127.57, 126.71, 125.56, 84.15, 34.64, 31.50, 25.07. (C-B) Carbon signal was not observed due to quadrupolar relaxation.[3]

^{11}B NMR (128 MHz, $CDCl_3$) δ: 31.3.

The spectral data are consistent with those reported in the literature.[13]

2.9. Procedure N and Characterization for Product (7)

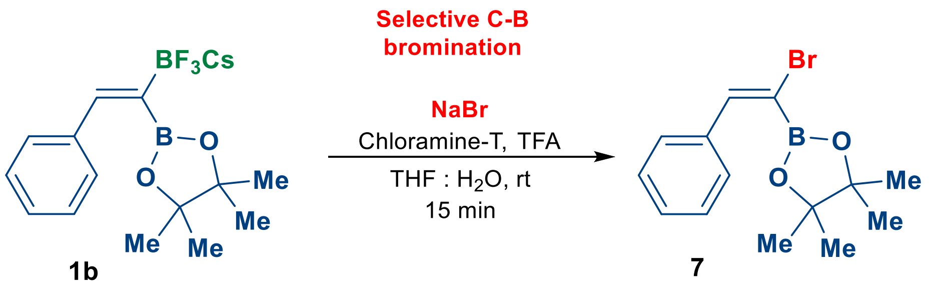

(E)-2-(1-bromo-2-phenylvinyl)-4,4,5,5-tetramethyl-1,3,2-dioxaborolane (7):

To a 25 mL round-bottom flask, trifluoroacetic acid (TFA) (0.033 mmol, 0.33 equiv) was added to a solution of organotrifluoroborate salt (**1b**) (0.1 mmol), sodium bromide and sodium chloro(4-methylbenzene-1-sulfonyl)azanide (Chloramine-T) in THF : H_2O (1:1, 1 ml). The reaction was stirred in open air at rt for 15 min. The reaction was quenched with water (2 ml), the aqueous layer was extracted with Et_2O (5 mL x 2). The organic phase was dried over (Na_2SO_4), filtered, and concentrated under reduced pressure (by evaporator) and the resulting crude material was purified using column chromatography to afford the desired pure product (**7**) in (27.7 mg, 90% yield).

R_f = 0.54 (10% EtOAc in hexane).

^{1}H NMR (400 MHZ, $CDCl_3$) δ: 7.64 (s, 1H), 7.36 - 7.34 (m, 2H), 7.34 - 7.26 (m, 3H), 1.30 (s, 12H).

^{13}C NMR (101 MHz, $CDCl_3$) δ: 145.76, 143.95, 137.01, 129.89, 129.20, 128.48, 128.43, 128.28, 127.93, 84.93, 24.60. (C-B) Carbon signal was not observed due to quadrupolar relaxation.[3]

^{11}B NMR (128 MHz, $CDCl_3$) δ: 28.7.

The spectral data are consistent with those reported in the literature.[15]

2.10. Procedure O and Characterization for Product (8)

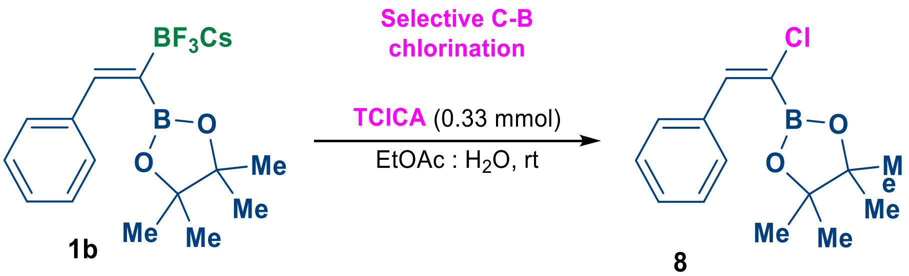

(E)-2-(1-chloro-2-phenylvinyl)-4,4,5,5-tetramethyl-1,3,2-dioxaborolane (8):

To a 25 mL round-bottom flask, trichloroisocyanuric acid (TCICA) (0.033 mmol, 0.33 equiv) was added in one portion to a solution of organotrifluoroborate salt (**1b**) (0.1 mmol) in EtOAc:H_2O (1:1, 1 ml). The reaction was stirred in open air at rt for 30 min. The reaction was quenched with water (2 ml), and the aqueous layer was extracted with Et_2O (5 ml x 2). The organic phase was dried over (Na_2SO_4), filtered, and concentrated under reduced pressure (by evaporator). The resulting crude material was purified using column chromatography to afford the desired pure product (**8**) in (10.5 mg, 40% yield).

R_f = 0.51 (10% EtOAc in hexane).

^{1}H NMR (400 MHZ, $CDCl_3$) δ: 7.40 (s, 1H), 7.38 - 7.35 (m, 2H), 7.32 - 7.28 (m, 3H), 1.30 (s, 12H).

^{13}C NMR (101 MHz, $CDCl_3$) δ: 143.43, 135.66, 128.41, 128.35, 128.28, 84.88, 24.65. (C-B) Carbon signal was not observed due to quadrupolar relaxation.[3]

^{11}B NMR (128 MHz, $CDCl_3$) δ: 28.0.

The spectral data are consistent with those reported in the literature.[16]

2.11. Procedure P and Characterization for Product (9)

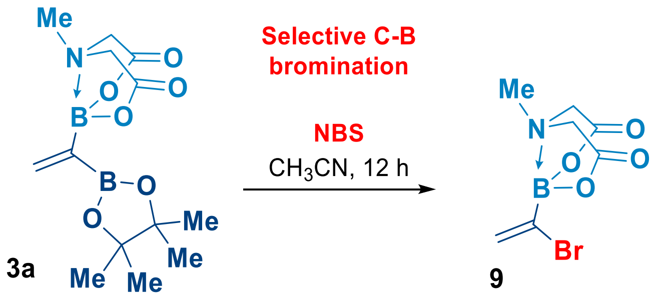

2-(1-bromovinyl)-6-methyl-1,3,6,2-dioxazaborocane-4,8-dione (9):

Into a 10 ml vial equipped with a magnetic stirring bar, **3a** (0.1 mmol, 1 equiv) and N-bromosuccinimide (NBS) were dissolved in 1 ml of CH_3CN under inert atmosphere (N_2). The vial was sealed and stirred for 12 h. After completion, the solvent was evaporated fully to give a crude solid, that was washed with diethyl ether and hexane to give product (**9**) as a white solid in (28.1 mg, 78% yield).

^{1}H NMR (400 MHZ, $CDCl_3$) δ: 6.39 - 6.38 (m, 1H), 6.22 (s, 1H), 4.08 - 3.91 (m, 4H), 2.94 (s, 3H).

^{11}B NMR (128 MHz, $CDCl_3$) δ: 8.6.

The spectral data are consistent with those reported in the literature.[17]

2.12. Procedure Q and Characterization for *gem*-(Bpin,Bdan) Product (10)

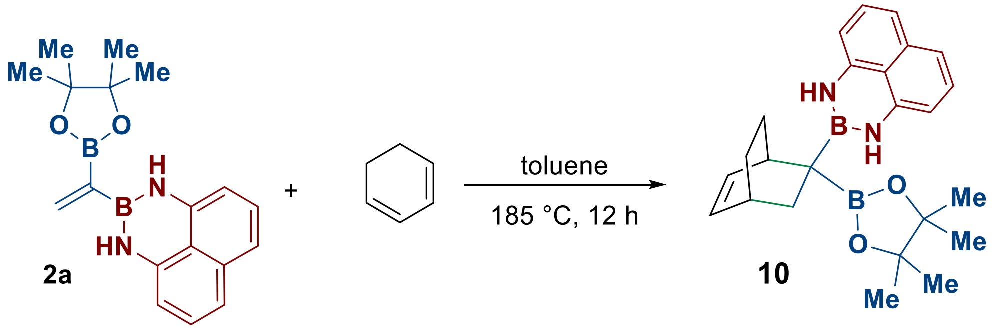

***2-(4,4,5,5-tetramethyl-1,3,2-dioxaborolan-2-yl)bicyclo[2.2.2]oct-5-en-2-yl)-2,3-dihydro-1H-naphtho[1,8-de][1,3,2]diazaborinine (10)*:**

Following a previous reported procedure.[2] *Gem*-diborylalkene (**2a**) (1 eq, 0.3 mmol) was inserted into a 10 ml vial and dissolved with toluene (3 ml), under inert conditions (N_2), followed by addition of 1,3-cyclohexadiene (3 eq, 0.9 mmol). The tube was sealed and heated in an oil bath for 12 h, after completion of the reaction the residue was concentrated under reduced pressure (by evaporator). The crude material was purified by short silica gel chromatography with EtOAc/hexane affording the cycloaddition product (**10**) in (98.7 mg, 82% yield), *d.r. = 1:1*, as a white solid.

R_f = 0.41 (10% EtOAc in hexane).

^{1}H NMR (400 MHZ, $CDCl_3$) δ: 7.12 - 6.96 (m, 8H), 6.48 - 6.36 (m, 2H), 6.33 - 6.24 (m, 4H), 6.25 - 6.22 (m, 2H), 5.94 (s, 2H), 5.76 (s, 2H), 2.82 (m, 2H), 2.63 - 2.60 (m, 2H), 2.11 - 2.04 (m, 4H), 1.86 - 1.70 (m, 4H), 1.63 - 1.41 (m, 4H), 1.27 - 1.18 (m, 24H).

^{13}C NMR (101 MHz, $CDCl_3$) δ: 141.63, 141.56, 136.64, 136.42, 136.40, 135.57, 133.99, 133.74, 127.72, 127.67, 119.61, 119.45, 117.35, 117.16, 105.60, 105.49, 83.59, 83.41, 34.58, 34.32, 32.11, 31.40, 30.52, 30.51, 26.96, 26.58, 25.04, 25.03, 24.82, 24.79, 24.73, 24.66. (C-B) Carbon signal was not observed due to quadrupolar relaxation.[3]

^{11}B NMR (128 MHz, $CDCl_3$) δ: 34.7, 31.92.

HRMS (ESI) Calcd for $[C_{24}H_{30}B_2N_2O_2+H]^+$ $[M+H]^+$: m/z 401.2575, found 401.2564

2.13. Procedure R and Characterization for *gem*-(Bpin,Bdan)- Product (11)

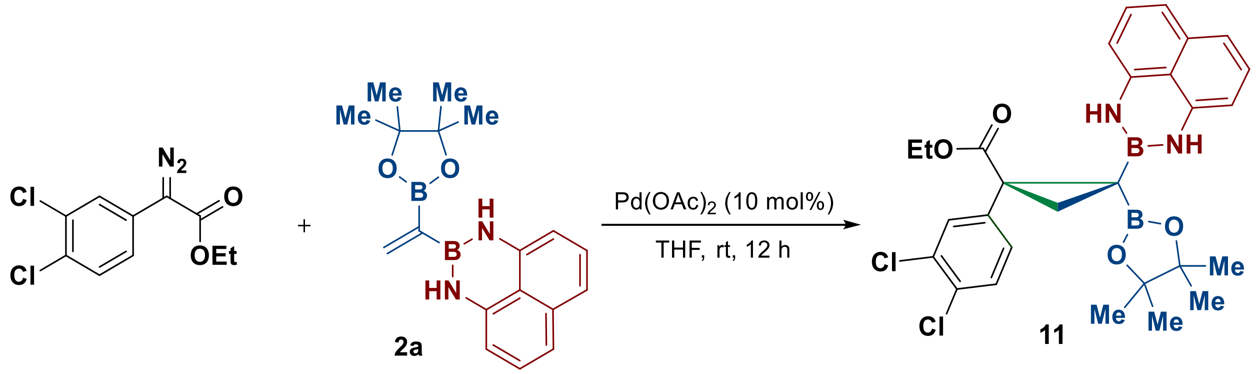

***Ethyl (1R,2R)-1-(3,4-dichlorophenyl)-2-(1H-naphtho[1,8-de][1,3,2]diazaborinin-2(3H)-yl)-2-(4,4,5,5-tetramethyl-1,3,2-dioxaborolan-2-yl)cyclopropane-1-carboxylate (11)*:**

Following a previous reported procedure.[18] *Gem*-diborylalkene (**2a**) (1 eq, 0.2 mmol) and $Pd(OAc)_2$ (10%) were dissolved in THF (2 ml) under inert conditions (N_2). The reaction was stirred for 10 min, and then α-diazoaryl solution (2 eq, 0.4 mmol in 6 ml THF) was added dropwise over a period of 12 h via syringe pump. The reaction was monitored by TLC, and after completion of the reaction, the residue was concentrated under reduced pressure (by evaporator) to give the crude material. That was purified by flash chromatography with EtOAc/hexane affording the *gem*-(Bpin-Bdan)-cyclopropane (**11**) in (78.2 mg, 71 % yield), *d.r. = 99:01*, as a white solid.

Note: Avoid bright lights near the setup of the reaction.

R_f = 0.42 (15% EtOAc in hexane).

^{1}H NMR (400 MHZ, $CDCl_3$) δ: 7.63 (d, *J* = 2.0 Hz, 1H), 7.39 - 7.32 (m, 2H), 7.12 - 7.08 (m, 2H), 7.01 - 6.98 (m, 2H), 6.34 (dd, *J* = 7.4, 1.01 Hz, 2H), 6.06 (s, 2H), 4.07 - 3.90 (m, 2H), 1.91 - 1.85 (m, 2H), 1.06 (s, 6H), 1.01 (t, *J* = 7.0 Hz, 3H), 0.82 (s, 6H).

^{13}C NMR (101 MHz, $CDCl_3$) δ: 171.93, 141.44, 138.50, 136.45, 133.80, 131.77, 131.67, 130.78, 129.68, 127.69, 119.73, 117.41, 105.68, 83.97, 61.84, 38.42, 25.12, 24.36, 19.56, 14.10. (C-B) Carbon signal was not observed due to quadrupolar relaxation.[3]

^{11}B NMR (128 MHz, $CDCl_3$) δ: 32.1.

HRMS (ESI) Calcd for $[C_{28}H_{30}B_2N_2O_4Cl_2+H]^+$ $[M+H]^+$: m/z 551.1841, found 551.1848.

Note: The relative configuration of **11** was determined by 2D-NMR NOESY (see Supplementary Figures 322-323, pages 258-259).

<u>Comparison of ^{11}B-NMR and ^{11}F-NMR Between (**V-1f**, **1f**, **2d** and **3f**)</u>

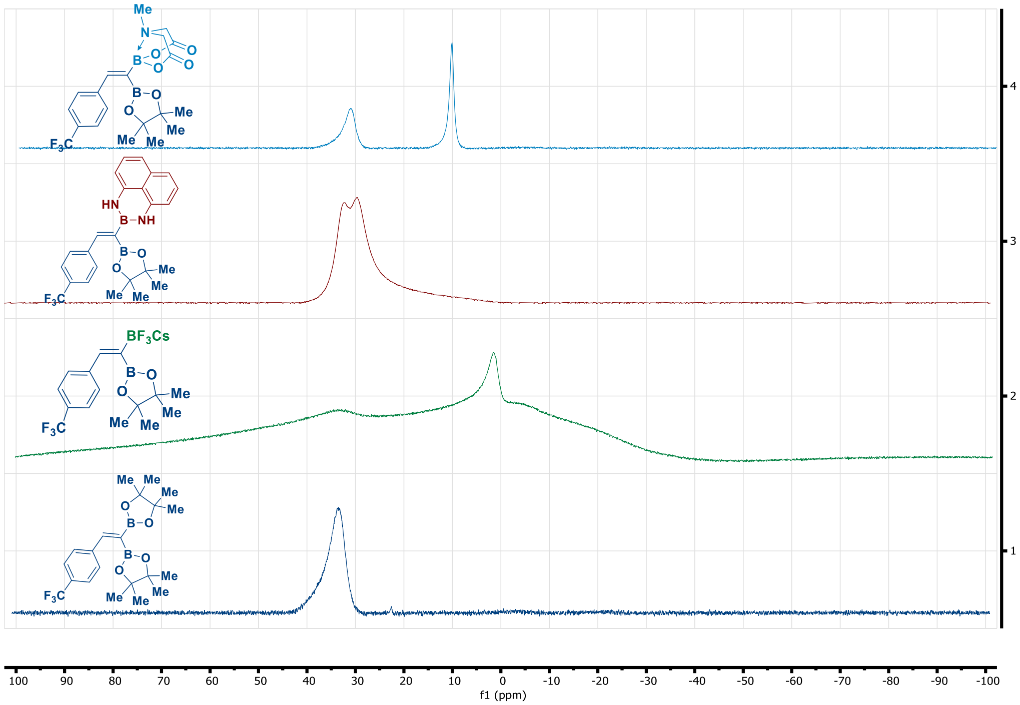

Supplementary Figure 1. Comparison of ^{11}B-NMR (128 MHz, DMSO-d_6) at room temperature

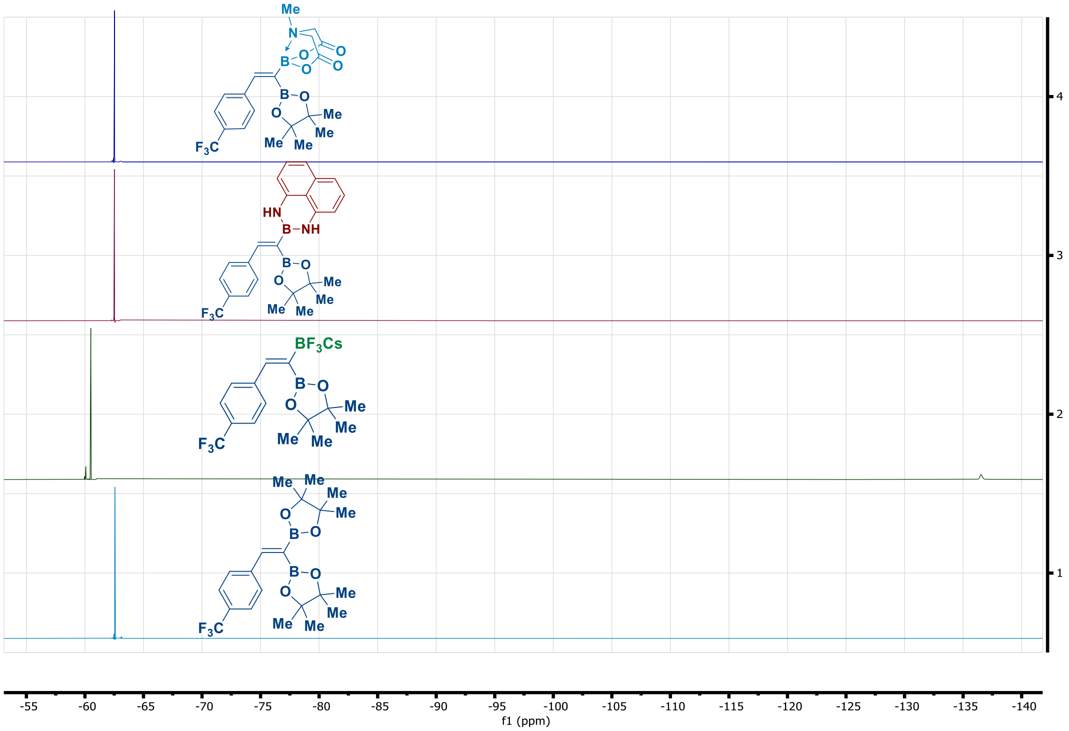

Supplementary Figure 2. Comparison of ^{19}F-NMR (376 MHz, DMSO-d_6) at room temperature

3. Supplementary Discussion

3.1. X-Ray Crystallography Data

Supplementary Table 5. Crystal data and structure refinement for **(2n)**

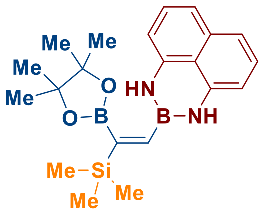

2n

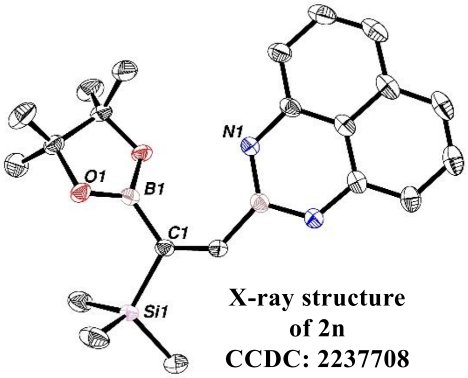

X-ray structure of 2n
CCDC: 2237708

Identification code	AhmadM40
Empirical formula	$C_{21}H_{30}B_2N_2O_2Si$
Formula weight	392.18
Temperature/K	149.99(10)
Crystal system	orthorhombic
Space group	Pbca
a/Å	9.7781(3)
b/Å	13.1745(4)
c/Å	34.287(1)
α/°	90
β/°	90
γ/°	90
Volume/Å^3	4416.9(2)
Z	8
ρ_{calc}g/cm^3	1.180
μ/mm^{-1}	0.125
F(000)	1680.0
Crystal size/mm^3	0.59 × 0.45 × 0.17
Radiation	Mo Kα (λ = 0.71073)
2Θ range for data collection/°	4.752 to 65.174
Index ranges	$-12 \leq h \leq 13$, $-16 \leq k \leq 18$, $-48 \leq l \leq 45$
Reflections collected	33281
Independent reflections	6585 [R_{int} = 0.0407, R_{sigma} = 0.0348]
Data/restraints/parameters	6585/0/268
Goodness-of-fit on F^2	1.033
Final R indexes [I>=2σ (I)]	R_1 = 0.0471, wR_2 = 0.1102
Final R indexes [all data]	R_1 = 0.0726, wR_2 = 0.1282
Largest diff. peak/hole / e Å^{-3}	0.38/-0.29

Supplementary Table 6. Crystal data and structure refinement for (**3a**)

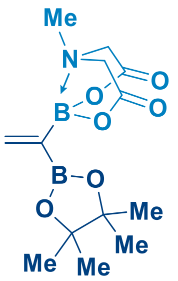

3a

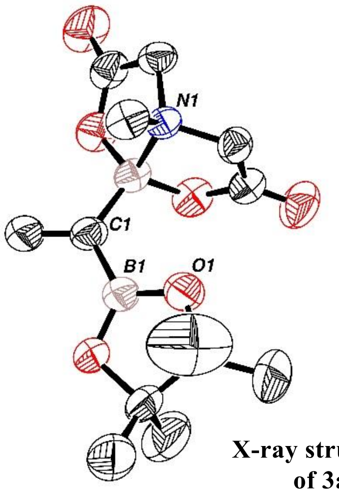

X-ray structure of 3a
CCDC: 2194038

Identification code	AhmadM6
Empirical formula	$C_{13}H_{21}B_2NO_6$
Formula weight	308.93
Temperature/K	295.4(2)
Crystal system	monoclinic
Space group	$P2_1/n$
a/Å	10.6704(5)
b/Å	14.1614(5)
c/Å	10.9924(5)
α/°	90
β/°	95.533(4)
γ/°	90
Volume/Å^3	1653.30(12)
Z	4
ρ_{calc}g/cm^3	1.241
μ/mm^{-1}	0.094
F(000)	656.0
Crystal size/mm^3	0.234 × 0.201 × 0.178
Radiation	Mo Kα (λ = 0.71073)
2Θ range for data collection/°	4.704 to 61.536
Index ranges	$-13 \le h \le 15$, $-19 \le k \le 20$, $-15 \le l \le 15$
Reflections collected	23608
Independent reflections	4405 [R_{int} = 0.0783, R_{sigma} = 0.0397]
Data/restraints/parameters	4405/0/204
Goodness-of-fit on F^2	1.062
Final R indexes [I>=2σ (I)]	R_1 = 0.0559, wR_2 = 0.1742
Final R indexes [all data]	R_1 = 0.0812, wR_2 = 0.1935
Largest diff. peak/hole / e Å^{-3}	0.23/-0.20

Supplementary Table 7. Crystal data and structure refinement for (**3b**)

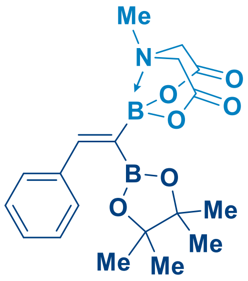

3b

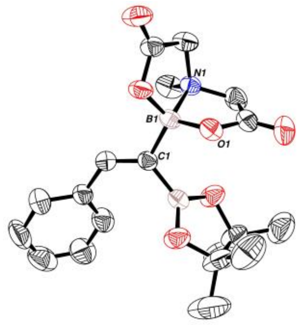

X-ray structure of 3b
CCDC: 2194037

Identification code	AhmadM8
Empirical formula	$C_{19}H_{25}B_2NO_6$
Formula weight	385.02
Temperature/K	293(2)
Crystal system	monoclinic
Space group	I2/a
a/Å	32.8250(15)
b/Å	6.6484(2)
c/Å	39.9201(18)
α/°	90
β/°	104.162(5)
γ/°	90
Volume/Å^3	8447.1(6)
Z	16
ρ_{calc}g/cm^3	1.211
μ/mm^{-1}	0.087
F(000)	3264.0
Crystal size/mm^3	0.212 × 0.055 × 0.046
Radiation	Mo Kα (λ = 0.71073)
2Θ range for data collection/°	3.69 to 59.418
Index ranges	$-39 \leq h \leq 44$, $-8 \leq k \leq 8$, $-53 \leq l \leq 49$
Reflections collected	47198
Independent reflections	9765 [R_{int} = 0.1248, R_{sigma} = 0.0998]
Data/restraints/parameters	9765/0/636
Goodness-of-fit on F^2	1.074
Final R indexes [I>=2σ (I)]	R_1 = 0.0902, wR_2 = 0.1842
Final R indexes [all data]	R_1 = 0.1773, wR_2 = 0.2200
Largest diff. peak/hole / e Å^{-3}	0.32/-0.29

Supplementary Table 8. Crystal data and structure refinement for (**3r**)

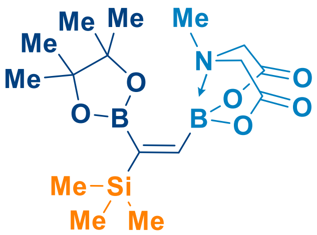

3r

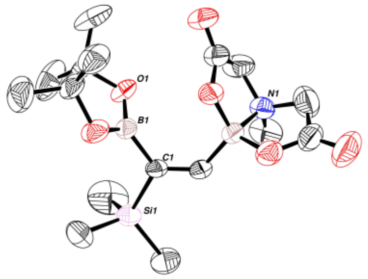

X-ray structure of 3r
CCDC: 2194039

Identification code	AhmadM15
Empirical formula	$C_{16}H_{29}B_2NO_6Si$
Formula weight	381.11
Temperature/K	297.9(9)
Crystal system	monoclinic
Space group	$P2_1/c$
a/Å	16.3694(7)
b/Å	10.9912(4)
c/Å	11.9185(5)
α/°	90
β/°	97.879(4)
γ/°	90
Volume/Å^3	2124.13(15)
Z	4
ρ_{calc}g/cm^3	1.192
μ/mm^{-1}	0.139
F(000)	816.0
Crystal size/mm^3	0.355 × 0.072 × 0.023
Radiation	Mo Kα (λ = 0.71073)
2Θ range for data collection/°	4.478 to 57.998
Index ranges	-22 ≤ h ≤ 21, -12 ≤ k ≤ 14, -15 ≤ l ≤ 16
Reflections collected	21009
Independent reflections	5575 [R_{int} = 0.0789, R_{sigma} = 0.0652]
Data/restraints/parameters	5575/0/273
Goodness-of-fit on F^2	1.022
Final R indexes [I>=2σ (I)]	R_1 = 0.0871, wR_2 = 0.2117
Final R indexes [all data]	R_1 = 0.1528, wR_2 = 0.2392
Largest diff. peak/hole / e Å^{-3}	0.45/-0.22

Supplementary Table 9. Crystal data and structure refinement for (**3aa**)

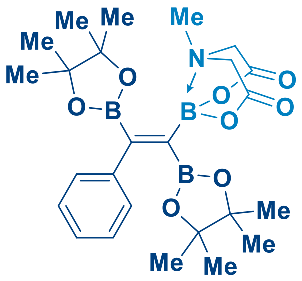

3aa

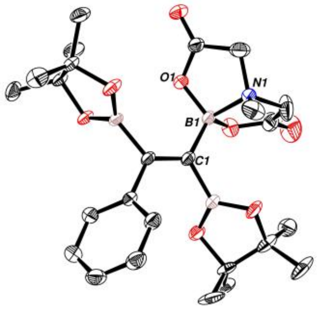

X-ray structure of 3aa
CCDC: 2194040

Identification code	AhmadM16
Empirical formula	$C_{25}H_{36}B_3NO_8$
Formula weight	510.98
Temperature/K	297.1(5)
Crystal system	monoclinic
Space group	$P2_1/c$
a/Å	13.3694(4)
b/Å	18.6603(6)
c/Å	12.0577(4)
α/°	90
β/°	116.212(4)
γ/°	90
Volume/Å^3	2698.78(17)
Z	4
ρ_{calc}g/cm^3	1.258
μ/mm^{-1}	0.090
F(000)	1088.0
Crystal size/mm^3	0.193 × 0.062 × 0.052
Radiation	Mo Kα (λ = 0.71073)
2Θ range for data collection/°	4.036 to 64.226
Index ranges	$-19 \leq h \leq 19$, $-26 \leq k \leq 23$, $-14 \leq l \leq 17$
Reflections collected	21877
Independent reflections	7518 [R_{int} = 0.0218, R_{sigma} = 0.0286]
Data/restraints/parameters	7518/0/401
Goodness-of-fit on F^2	1.039
Final R indexes [I>=2σ (I)]	R_1 = 0.0625, wR_2 = 0.1641
Final R indexes [all data]	R_1 = 0.0961, wR_2 = 0.1825
Largest diff. peak/hole / e Å^{-3}	0.31/-0.26

Supplementary Table 10. Crystal data and structure refinement for (**V-4**)

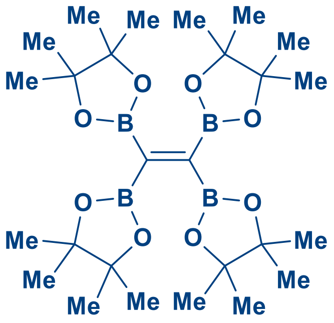

V-4

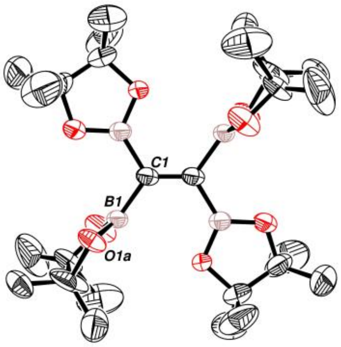

X-ray structure of V-4
CCDC: 2194042

Identification code	AhmadM24c_autored
Empirical formula	$C_{26}H_{48}B_4O_8$
Formula weight	531.88
Temperature/K	229.98(11)
Crystal system	orthorhombic
Space group	Pbca
a/Å	12.6263(5)
b/Å	11.5215(5)
c/Å	22.3824(8)
α/°	90
β/°	90
γ/°	90
Volume/Å^3	3256.1(2)
Z	4
ρ_{calc}g/cm^3	1.085
μ/mm^{-1}	0.076
F(000)	1152.0
Crystal size/mm^3	0.391 × 0.275 × 0.105
Radiation	Mo Kα (λ = 0.71073)
2Θ range for data collection/°	4.864 to 64.516
Index ranges	$-18 \le h \le 14$, $-17 \le k \le 15$, $-32 \le l \le 31$
Reflections collected	22776
Independent reflections	4901 [R_{int} = 0.0362, R_{sigma} = 0.0365]
Data/restraints/parameters	4901/6/323
Goodness-of-fit on F^2	1.029
Final R indexes [I>=2σ (I)]	R_1 = 0.0814, wR_2 = 0.2138
Final R indexes [all data]	R_1 = 0.1271, wR_2 = 0.2421
Largest diff. peak/hole / e Å^{-3}	0.52/-0.22

Supplementary Table 11. Crystal data and structure refinement for (**4**)

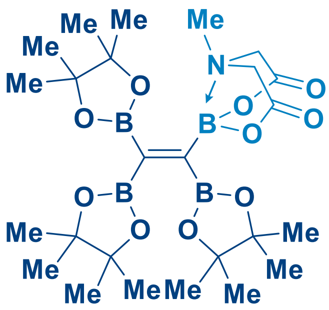

4

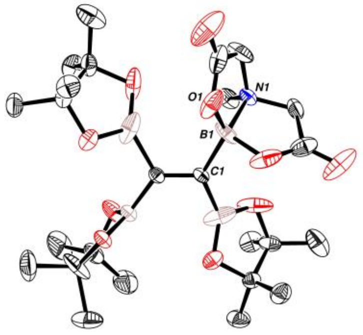

X-ray structure of 4
CCDC: 2194041

Identification code	AhmadM22a
Empirical formula	$C_{25}H_{43}B_4NO_{10}$
Formula weight	560.84
Temperature/K	149.98(10)
Crystal system	orthorhombic
Space group	$Cmc2_1$
a/Å	21.5968(6)
b/Å	11.3917(3)
c/Å	12.6511(3)
α/°	90
β/°	90
γ/°	90
Volume/Å^3	3112.48(14)
Z	4
ρ_{calc}g/cm^3	1.197
μ/mm^{-1}	0.088
F(000)	1200.0
Crystal size/mm^3	0.29 × 0.15 × 0.05
Radiation	Mo Kα (λ = 0.71073)
2Θ range for data collection/°	5.168 to 55.992
Index ranges	-27 ≤ h ≤ 28, -15 ≤ k ≤ 14, -16 ≤ l ≤ 15
Reflections collected	14015
Independent reflections	3680 [R_{int} = 0.0201, R_{sigma} = 0.0186]
Data/restraints/parameters	3680/1/233
Goodness-of-fit on F^2	1.094
Final R indexes [I>=2σ (I)]	R_1 = 0.0491, wR_2 = 0.1125
Final R indexes [all data]	R_1 = 0.0535, wR_2 = 0.1145
Largest diff. peak/hole / e Å^{-3}	0.21/-0.25
Flack parameter	-0.1(3)

3.2 NMR Spectra

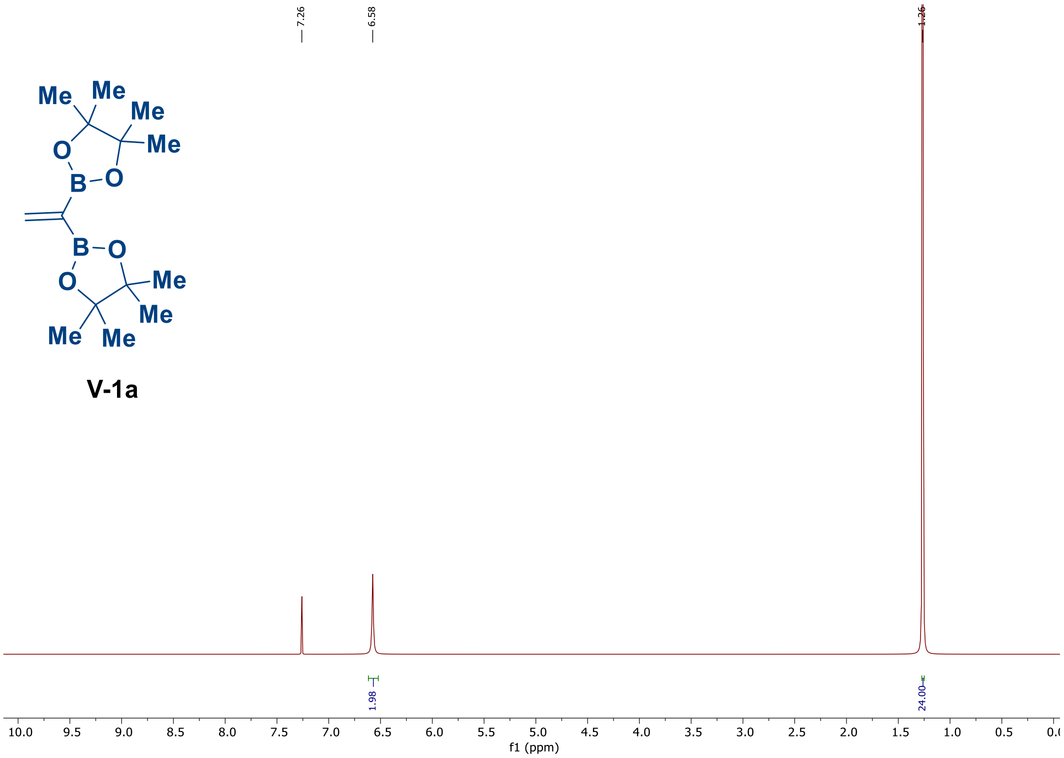

Supplementary Figure 3. ^{1}H NMR (400 MHz, $CDCl_3$) of compound (**V-1a**).

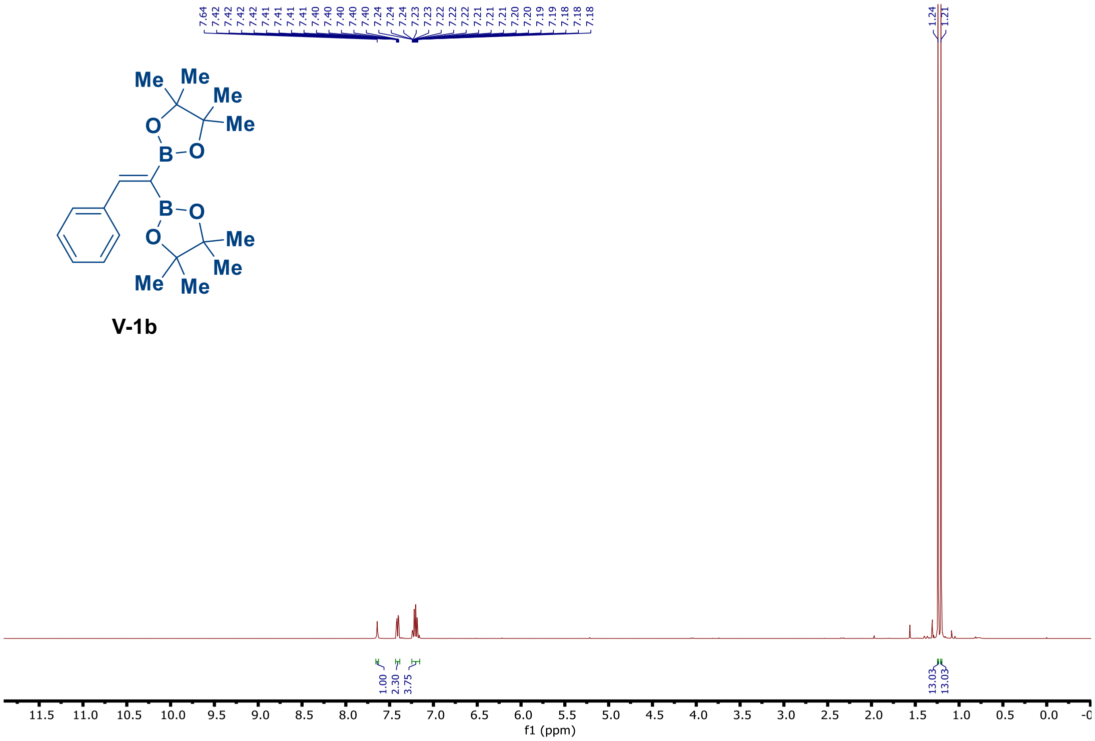

Supplementary Figure 4. ^{1}H NMR (400 MHz, $CDCl_3$) of compound (**V-1b**).

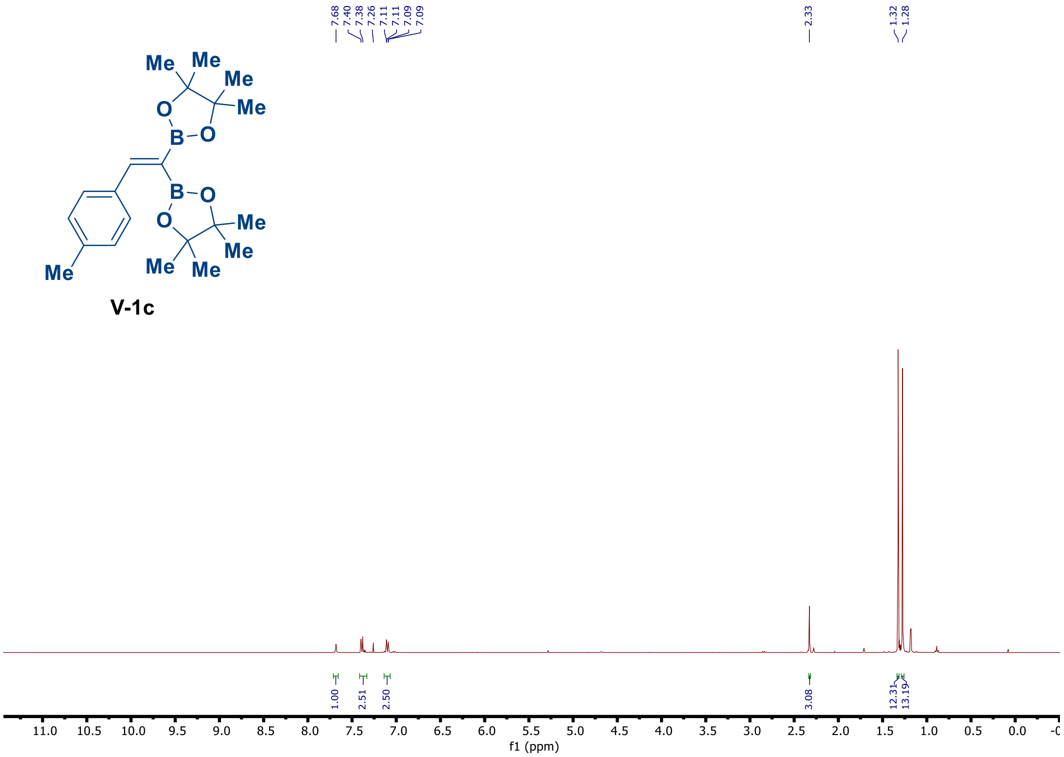

Supplementary Figure 5. ^{1}H NMR (400 MHz, $CDCl_3$) of compound (**V-1c**).

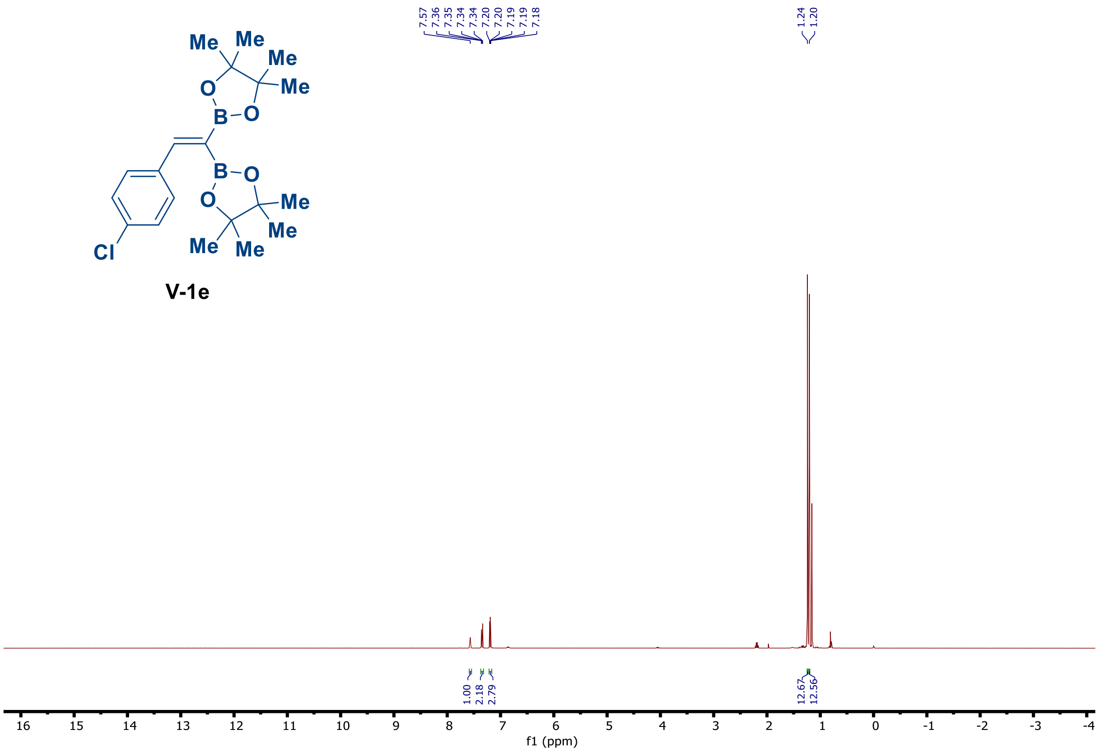

Supplementary Figure 6. ^{1}H NMR (400 MHz, $CDCl_3$) of compound (**V-1e**).

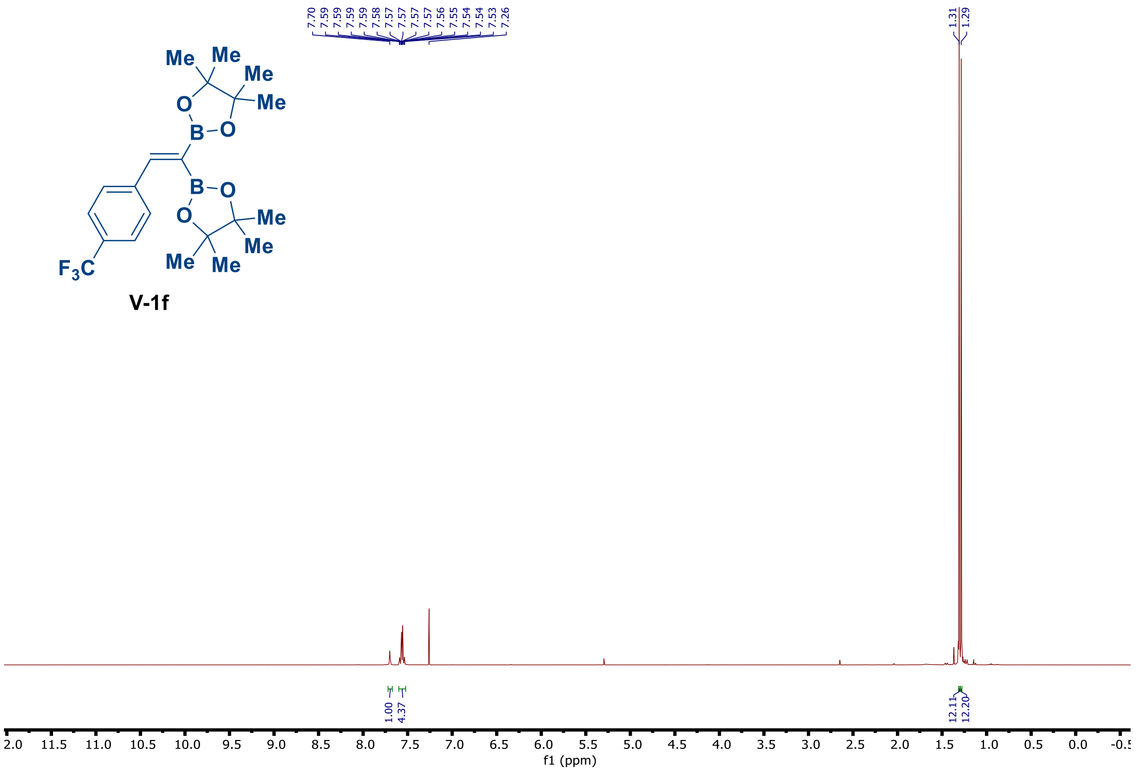

Supplementary Figure 7. ^{1}H NMR (400 MHz, $CDCl_3$) of compound (**V-1f**).

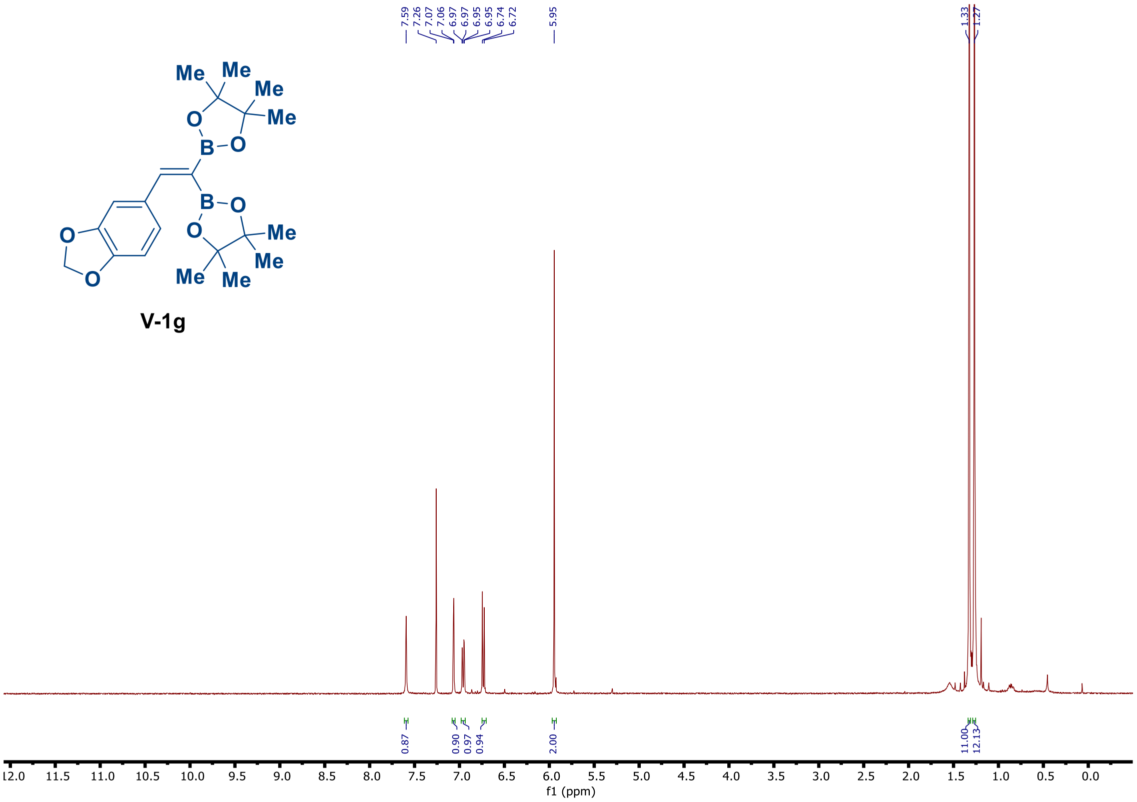

Supplementary Figure 8. ^{1}H NMR (400 MHz, $CDCl_3$) of compound (**V-1g**).

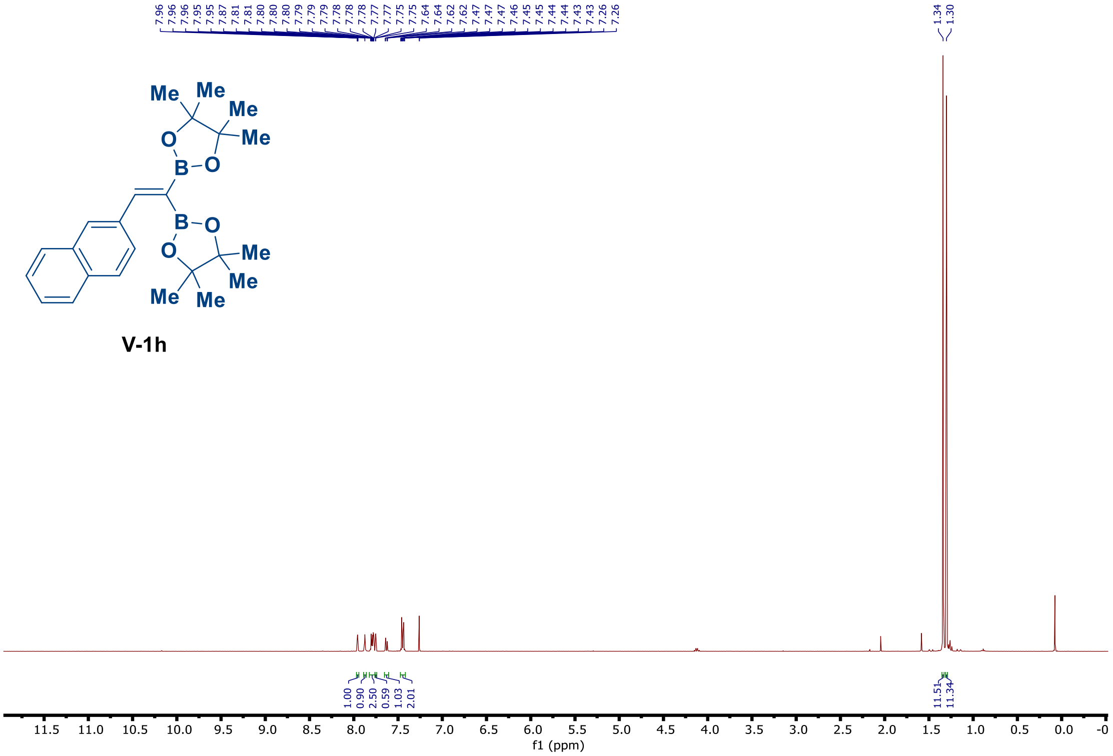

Supplementary Figure 9. ^{1}H NMR (400 MHz, $CDCl_3$) of compound (**V-1h**).

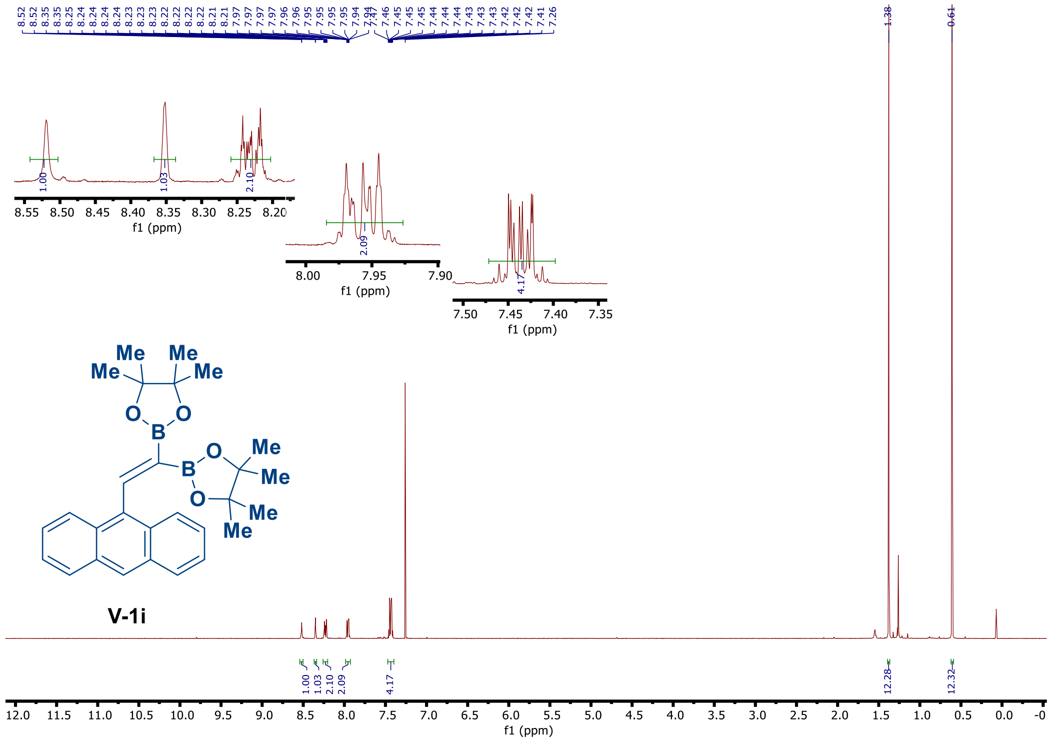

Supplementary Figure 10. ^{1}H NMR (400 MHz, $CDCl_3$) of compound (**V-1i**).

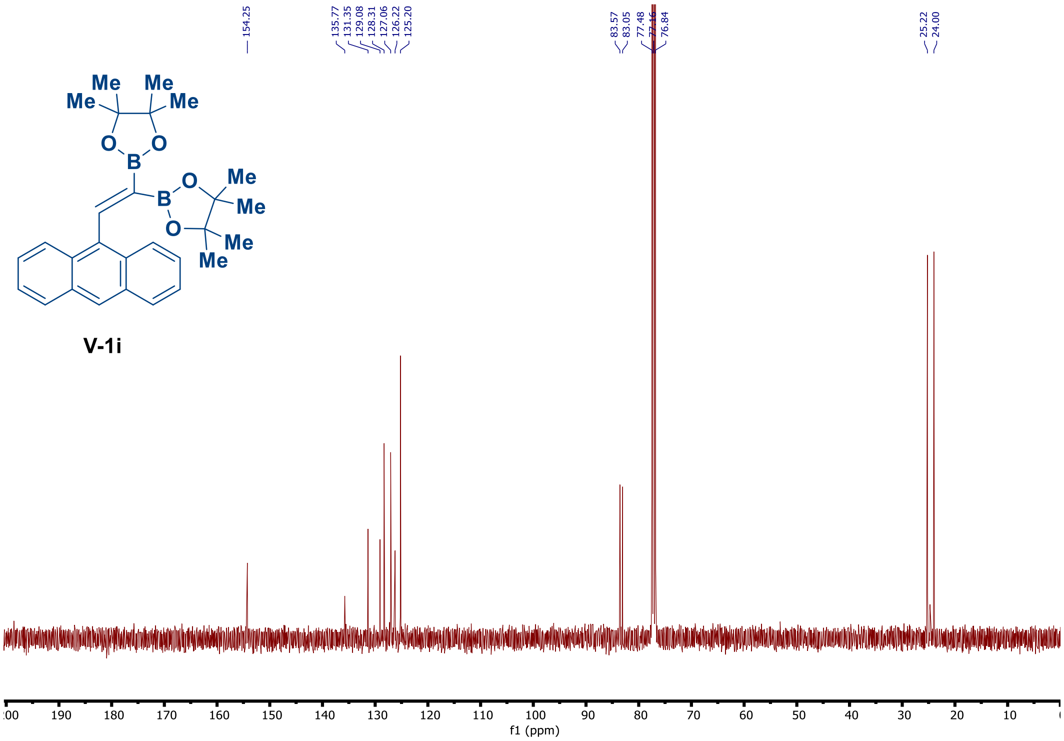

Supplementary Figure 11. ^{13}C NMR (400 MHz, $CDCl_3$) of compound (**V-1i**).

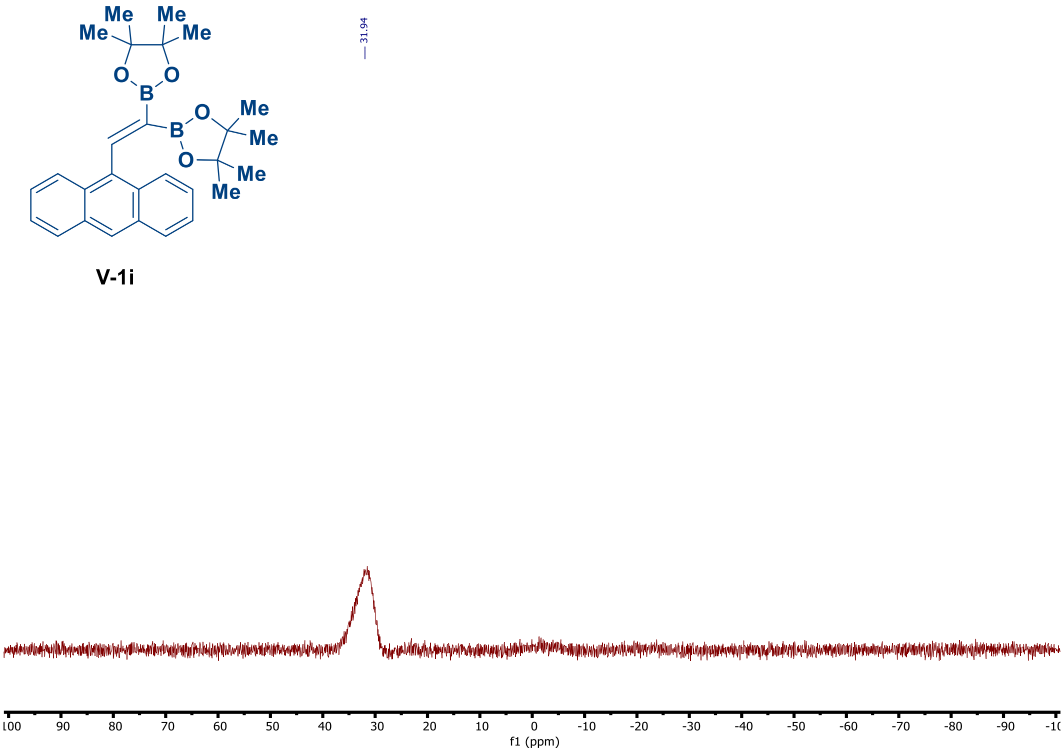

Supplementary Figure 12. ^{11}B NMR (400 MHz, $CDCl_3$) of compound (**V-1i**).

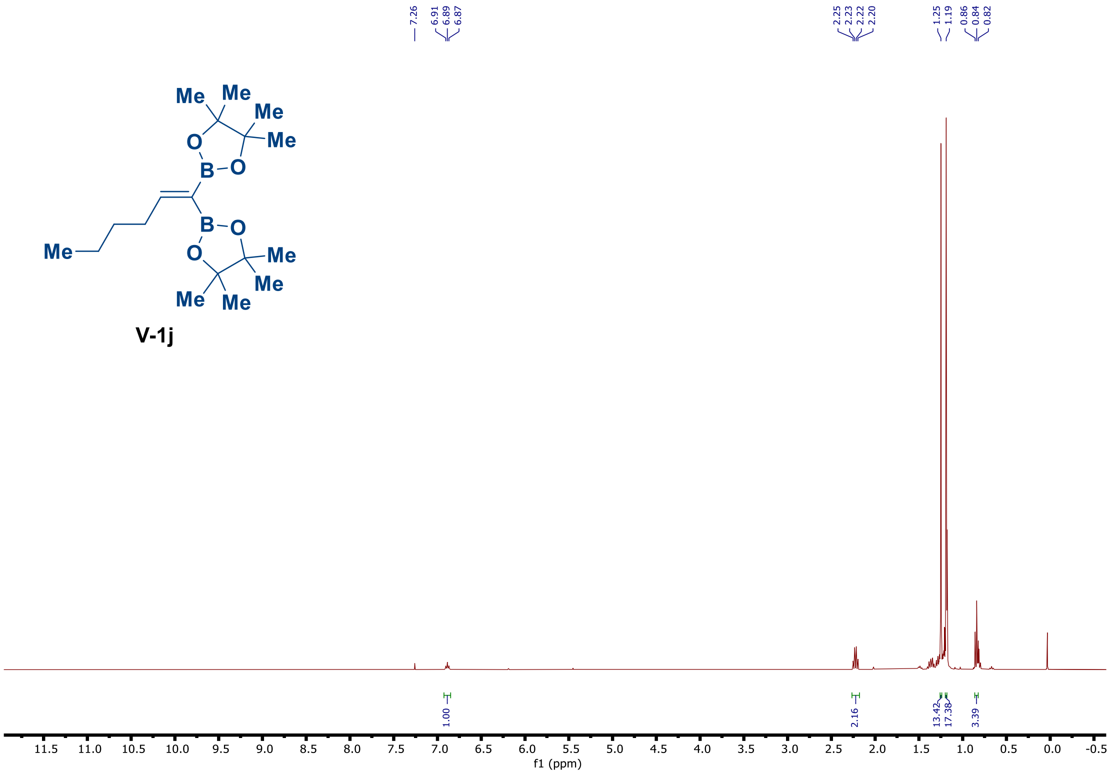

Supplementary Figure 13. ^{1}H NMR (400 MHz, $CDCl_3$) of compound (**V-1j**).

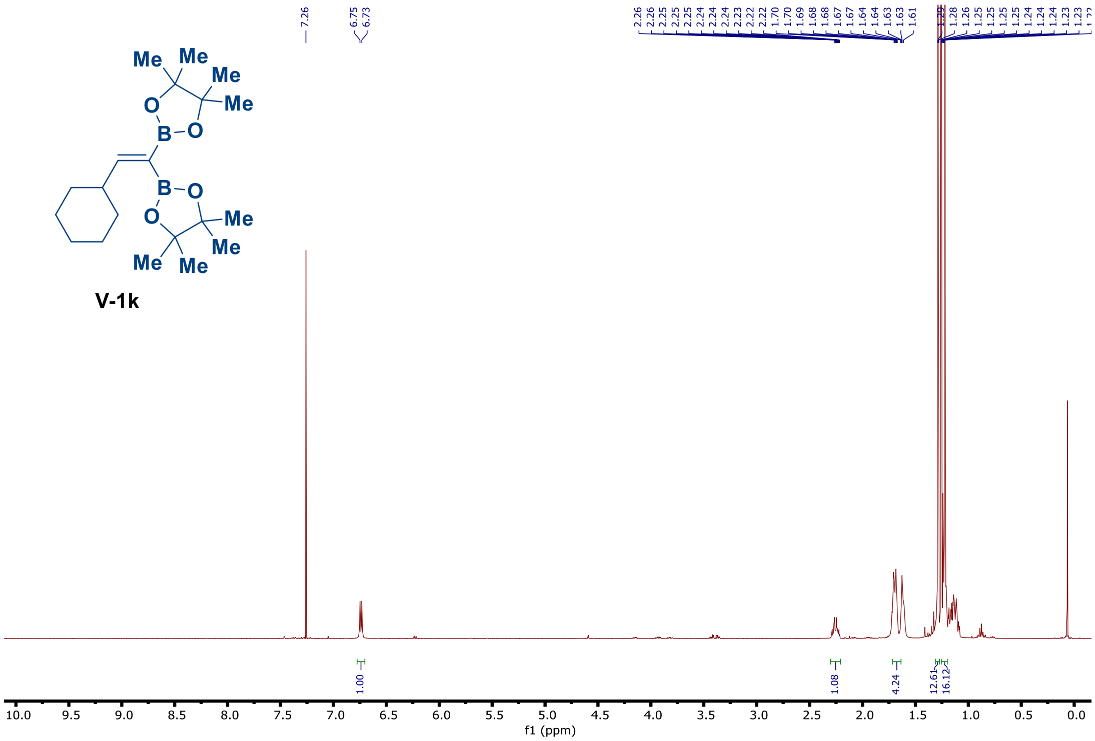

Supplementary Figure 14. ^{1}H NMR (400 MHz, $CDCl_3$) of compound (**V-1k**).

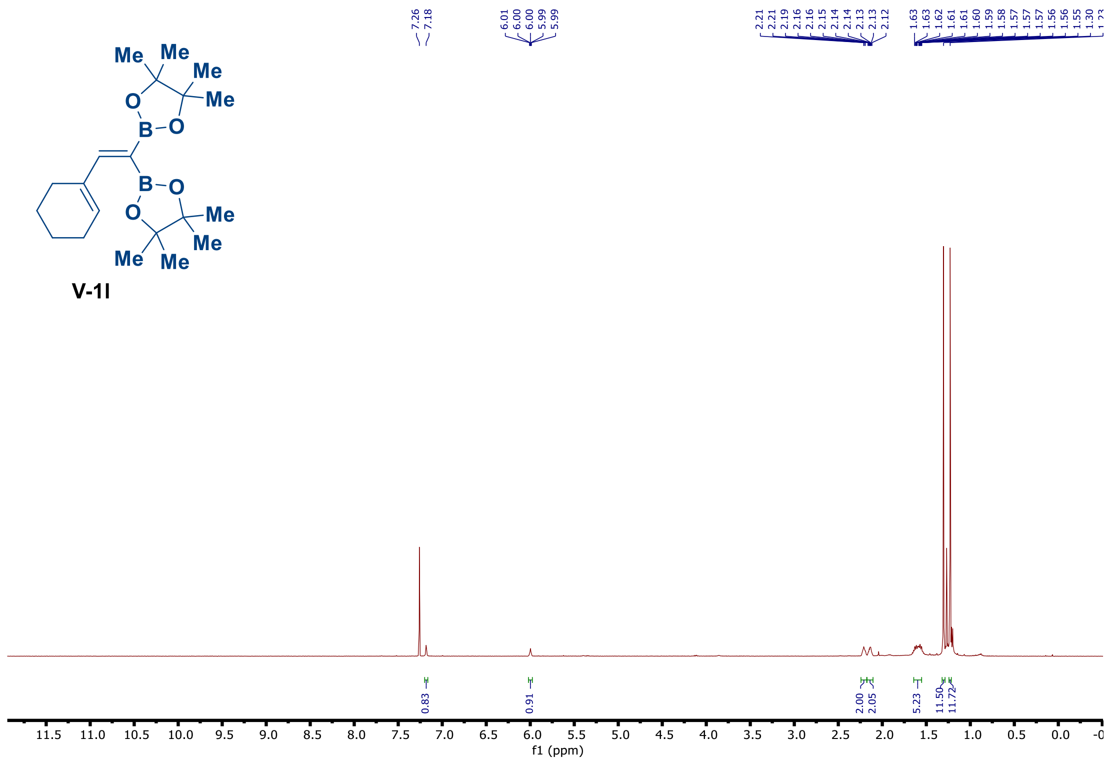

Supplementary Figure 15. ^{1}H NMR (400 MHz, $CDCl_3$) of compound (**V-1l**).

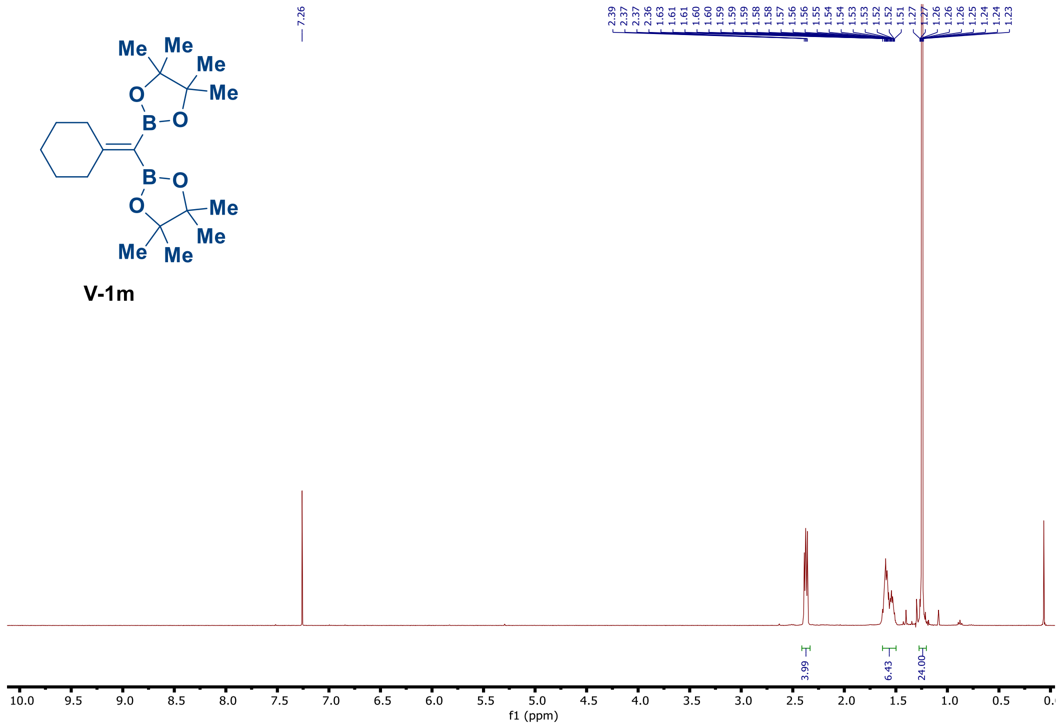

Supplementary Figure 16. ^{1}H NMR (400 MHz, $CDCl_3$) of compound (**V-1m**).

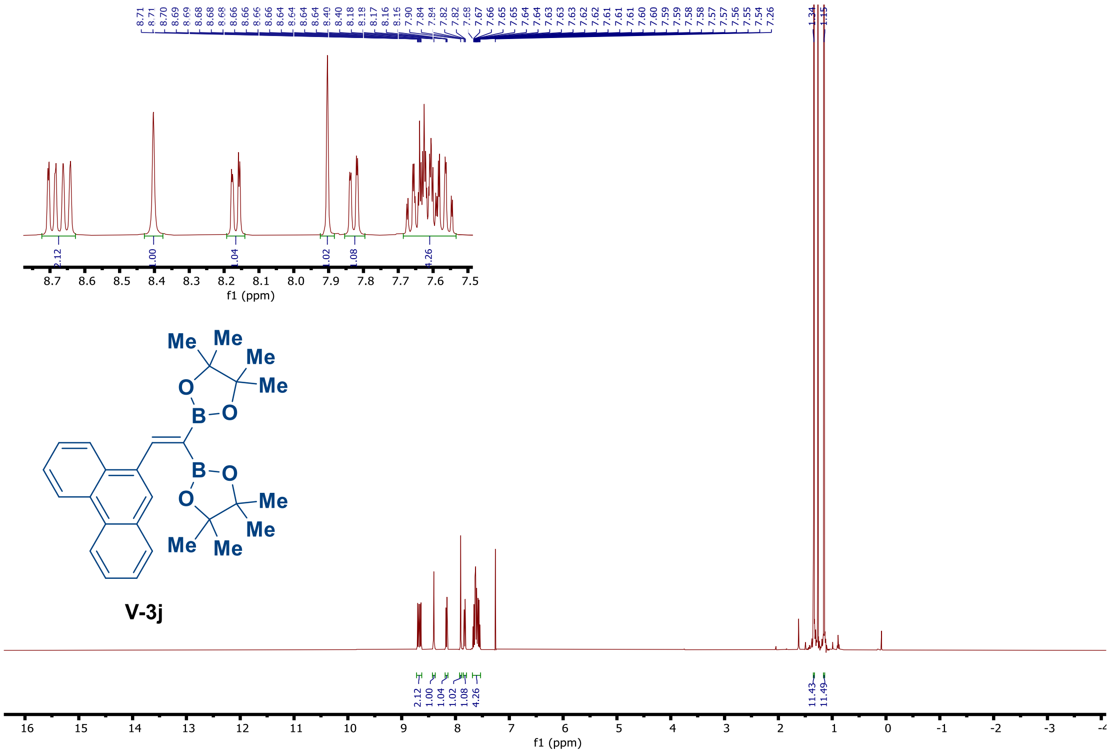

Supplementary Figure 17. ^{1}H NMR (400 MHz, $CDCl_3$) of compound (**V-3j**).

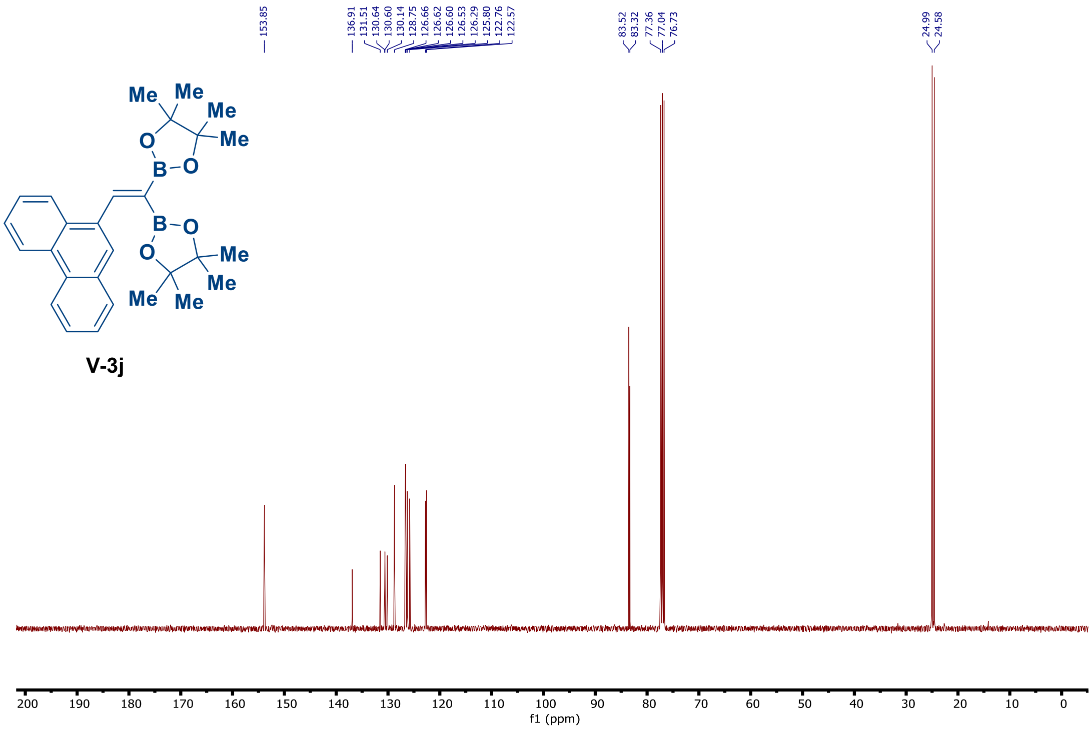

Supplementary Figure 18. ^{13}C NMR (400 MHz, $CDCl_3$) of compound (**V-3j**).

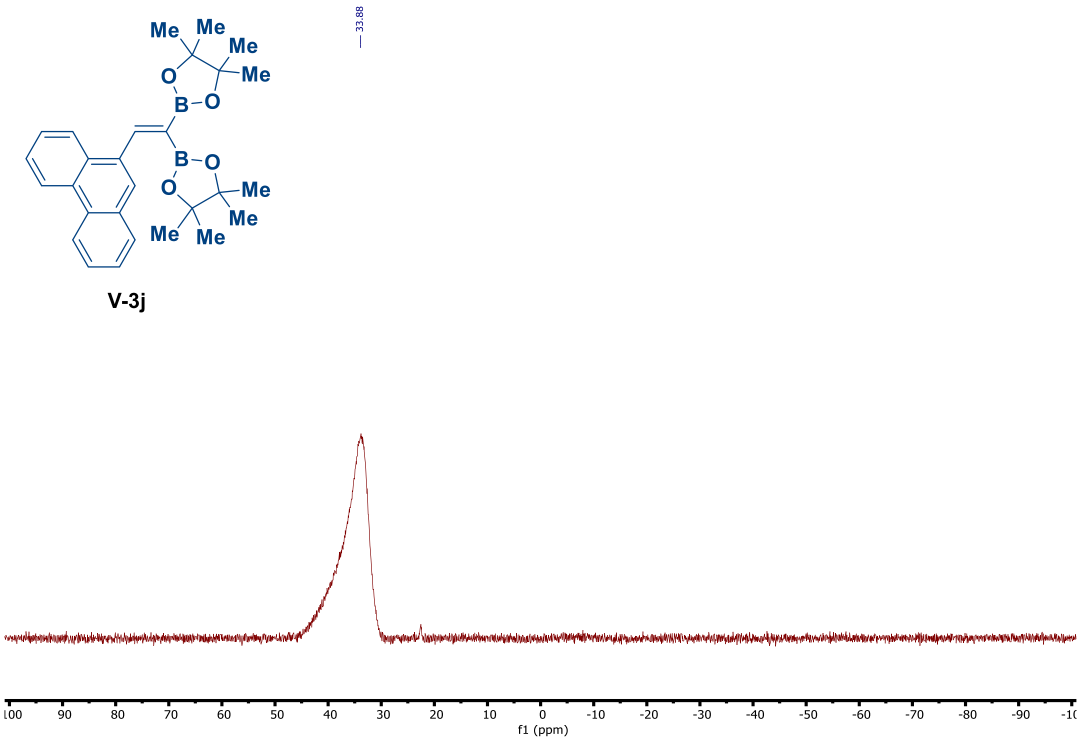

Supplementary Figure 19. ^{11}B NMR (400 MHz, $CDCl_3$) of compound (**V-3j**).

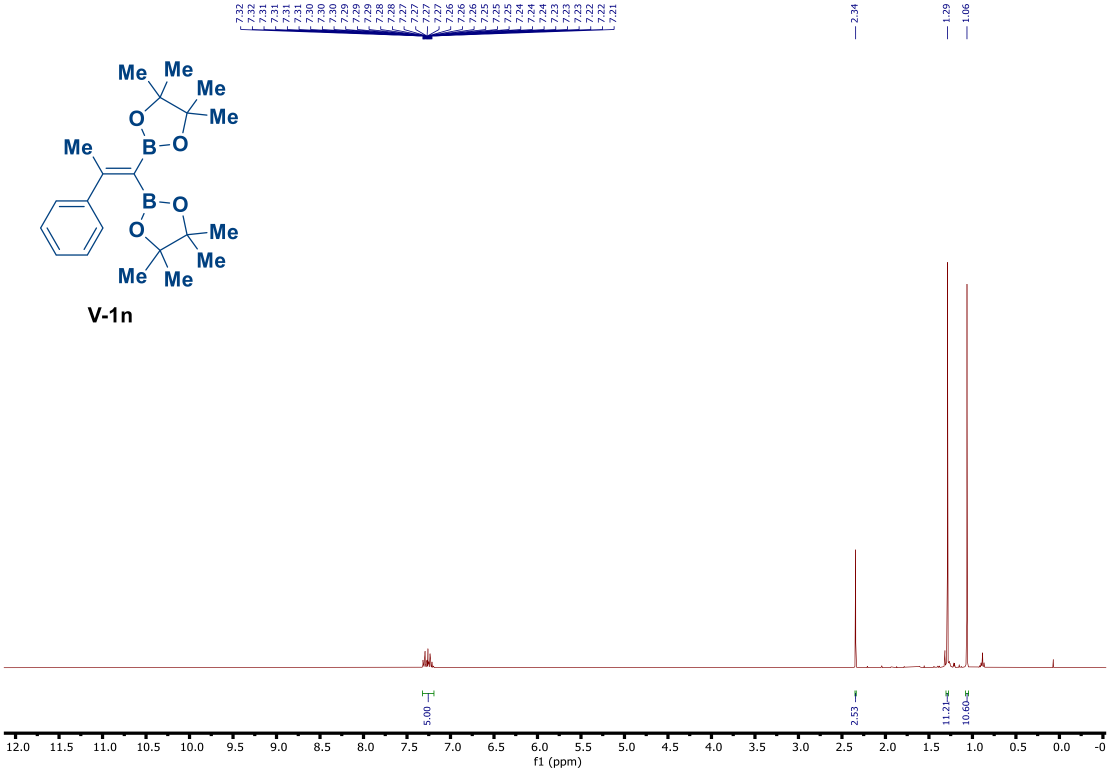

Supplementary Figure 20. ^{1}H NMR (400 MHz, $CDCl_3$) of compound (**V-1n**).

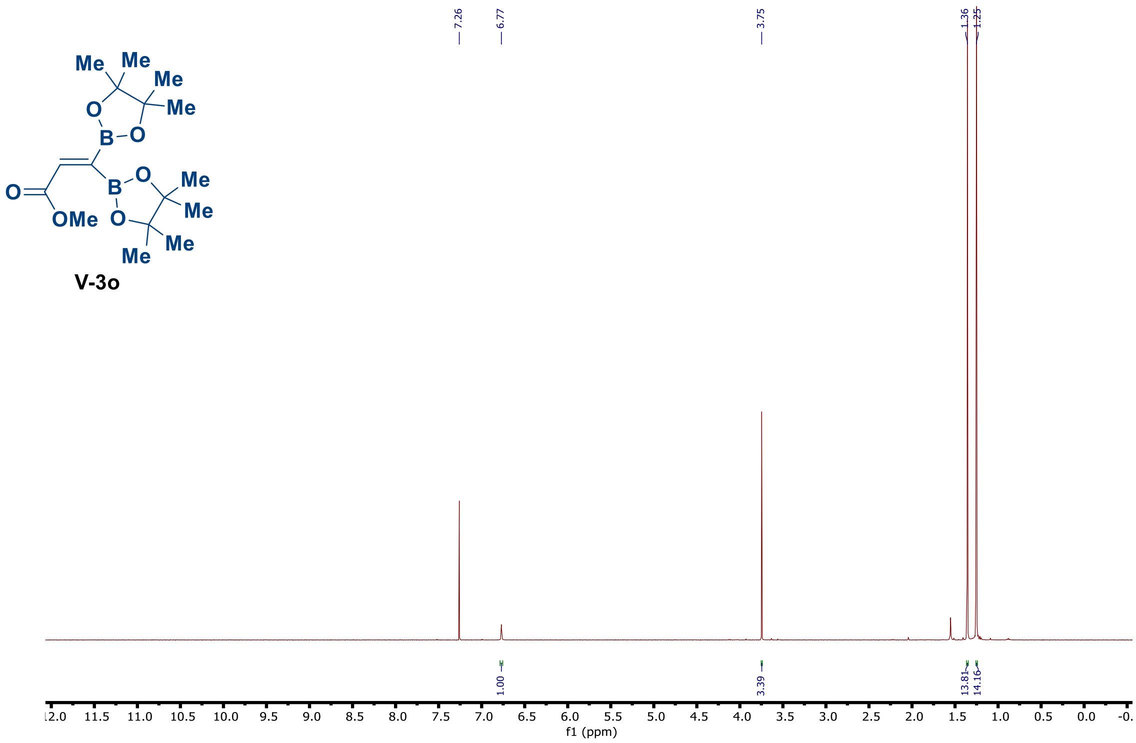

Supplementary Figure 21. ^{1}H NMR (400 MHz, $CDCl_3$) of compound (**V-1o**).

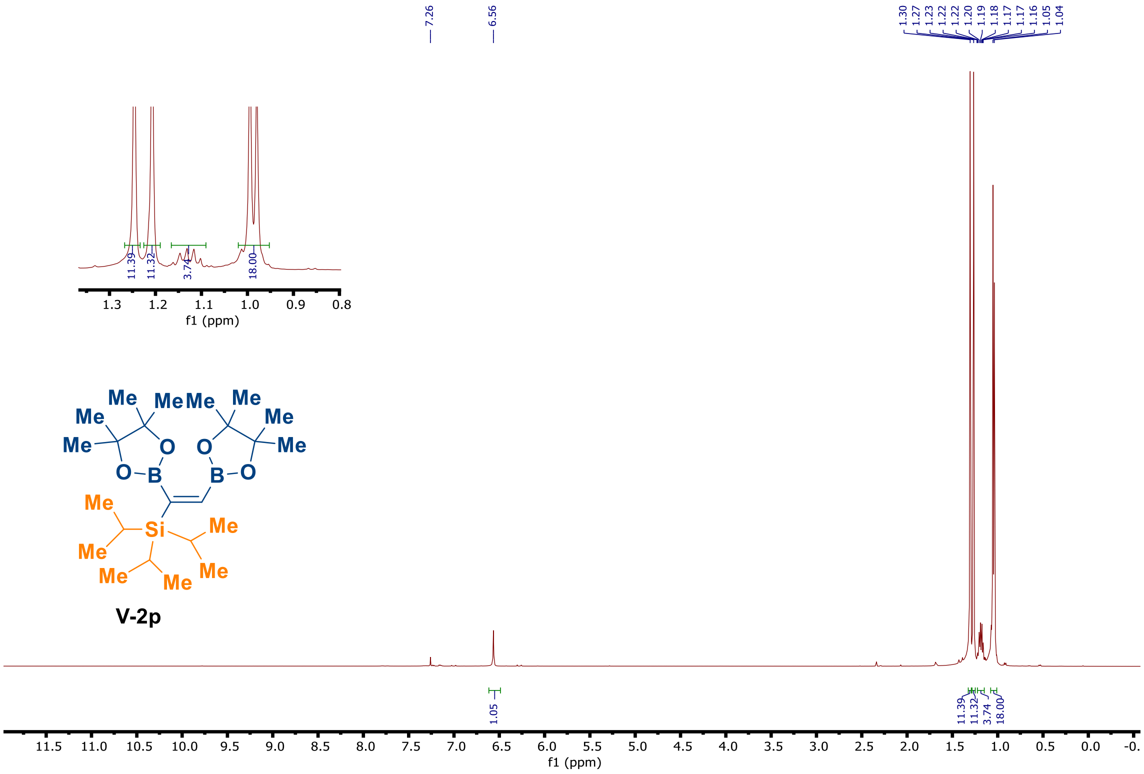

Supplementary Figure 22. ^{1}H NMR (400 MHz, $CDCl_3$) of compound (**V-2p**).

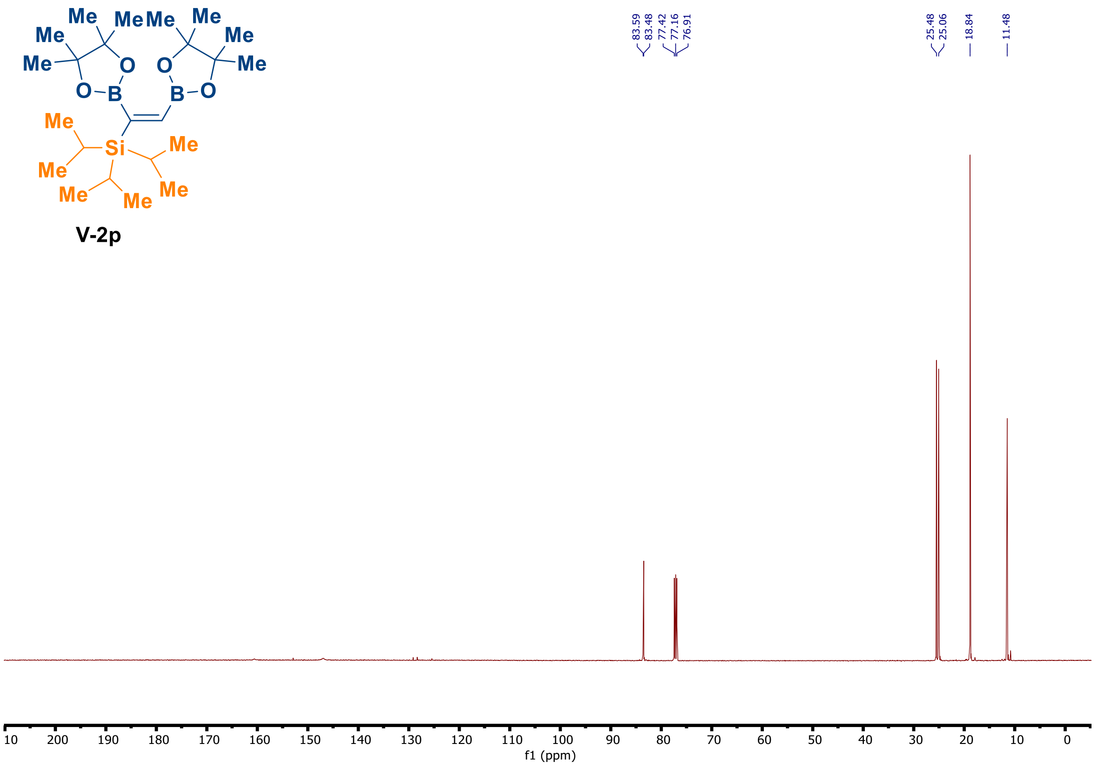

Supplementary Figure 23. ^{13}C NMR (400 MHz, $CDCl_3$) of compound (**V-2p**).

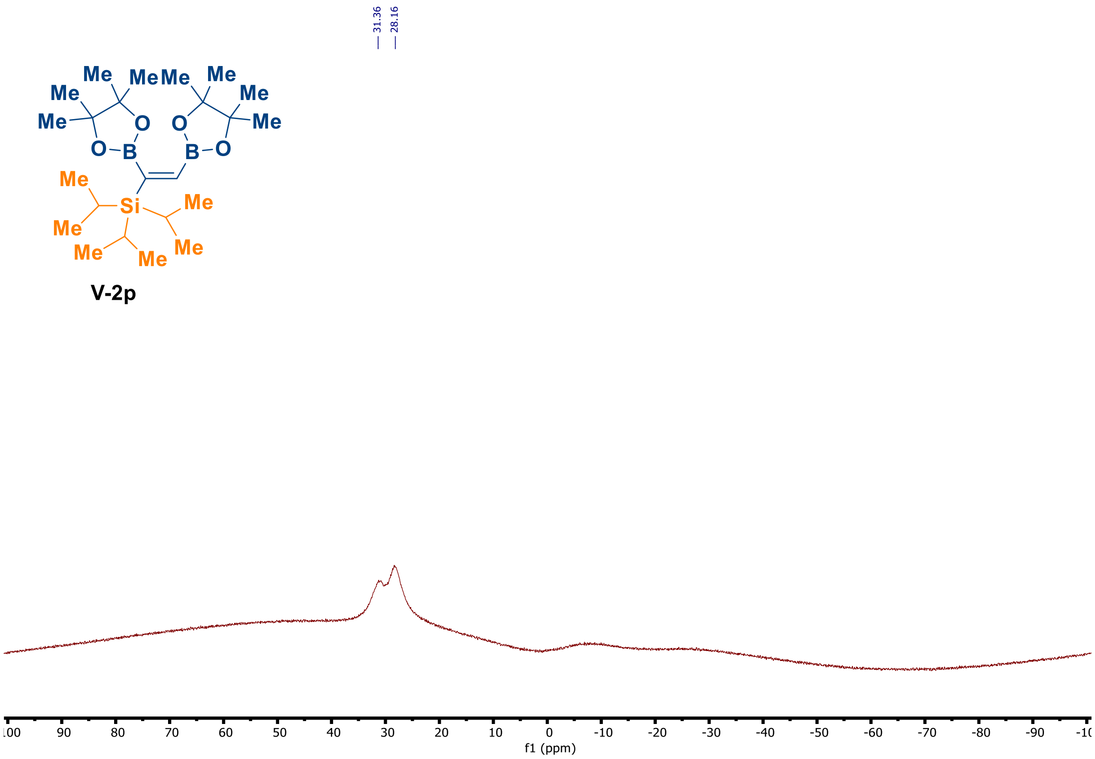

Supplementary Figure 24. ^{11}B NMR (128 MHz, $CDCl_3$) of compound (**V-2p**).

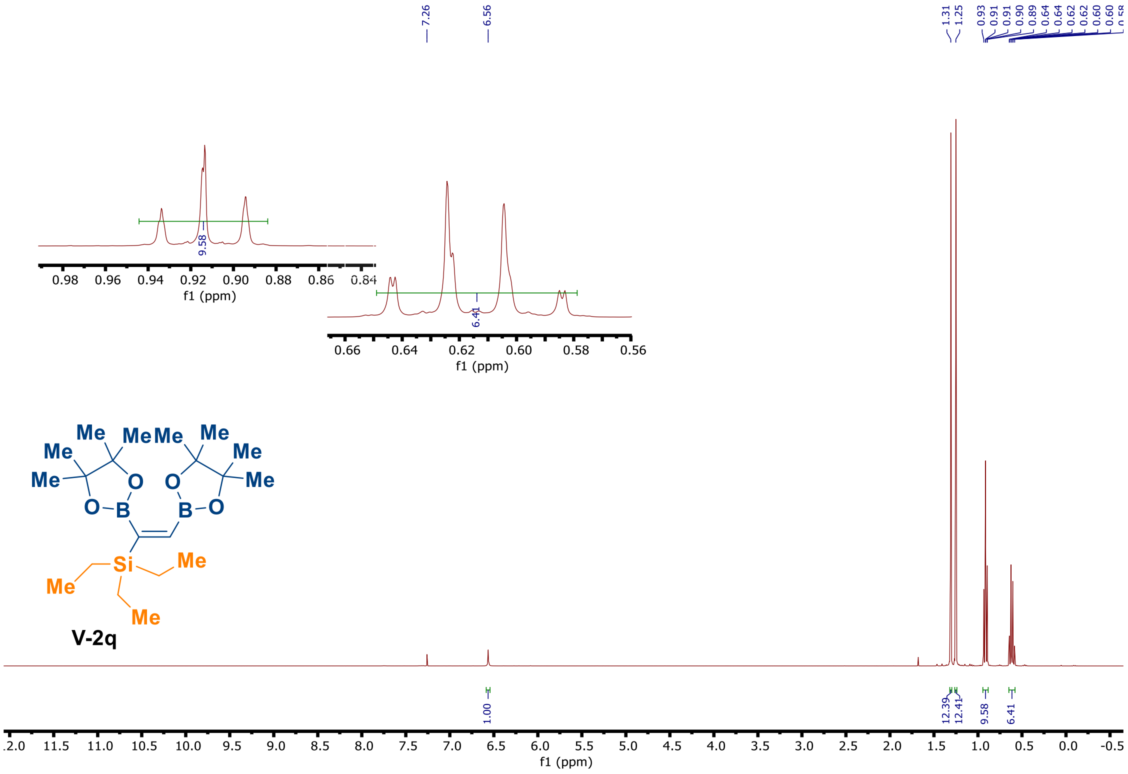

Supplementary Figure 25. ^{1}H NMR (400 MHz, $CDCl_3$) of compound (**V-2q**).

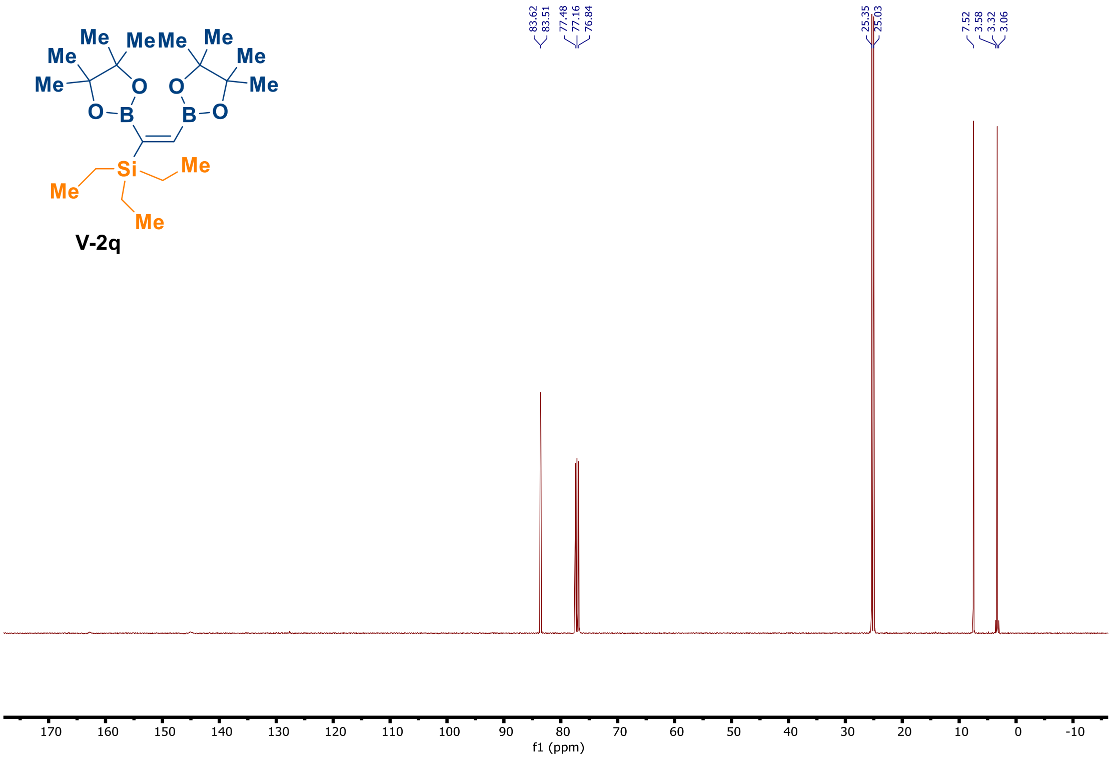

Supplementary Figure 26. ^{13}C NMR (400 MHz, $CDCl_3$) of compound (**V-2q**).

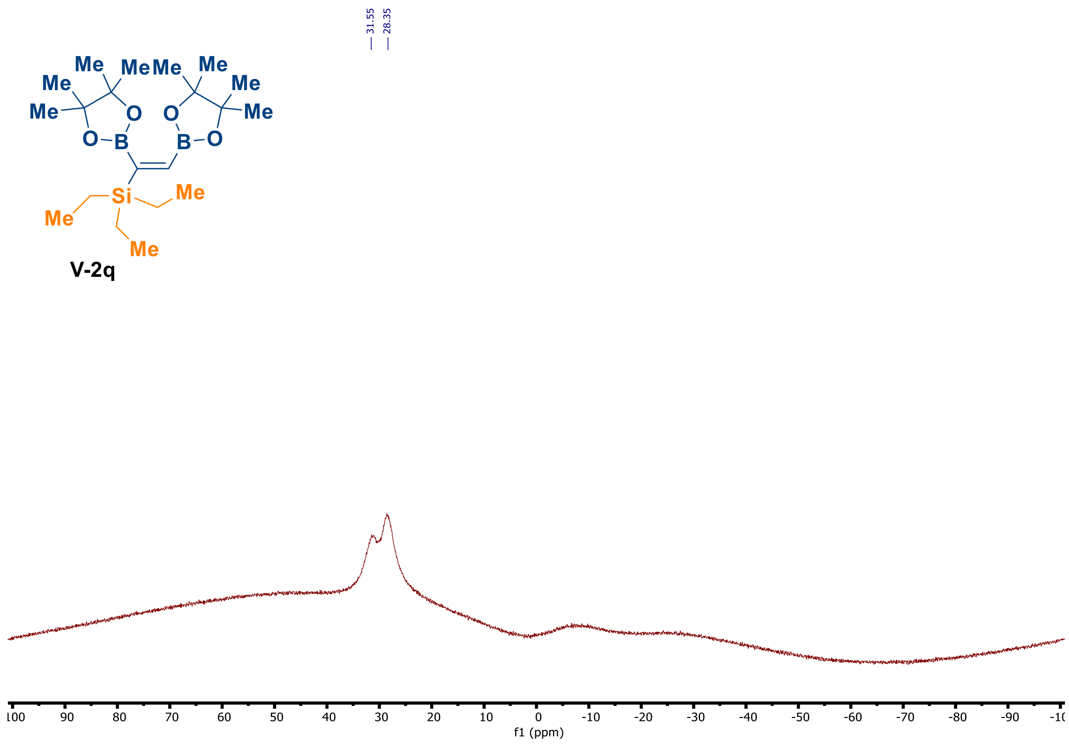

Supplementary Figure 27. ^{11}B NMR (128 MHz, $CDCl_3$) of compound (**V-2q**).

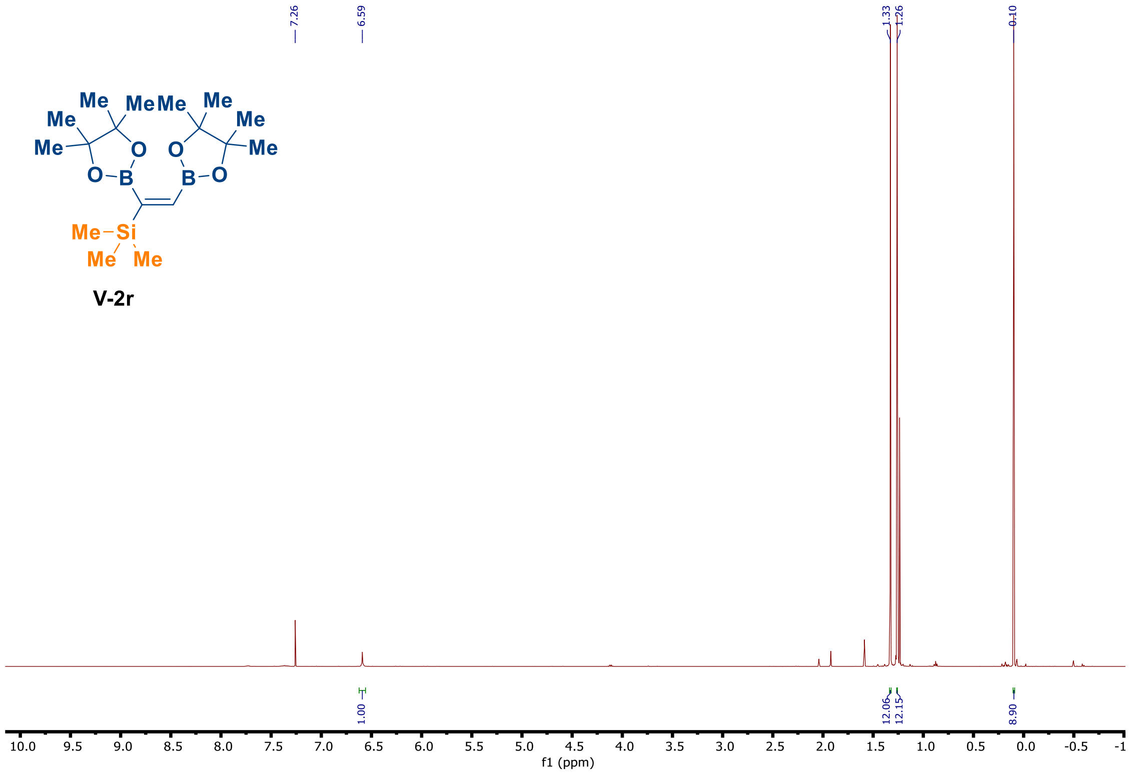

Supplementary Figure 28. ^{1}H NMR (500 MHz, $CDCl_3$) of compound (**V-2r**).

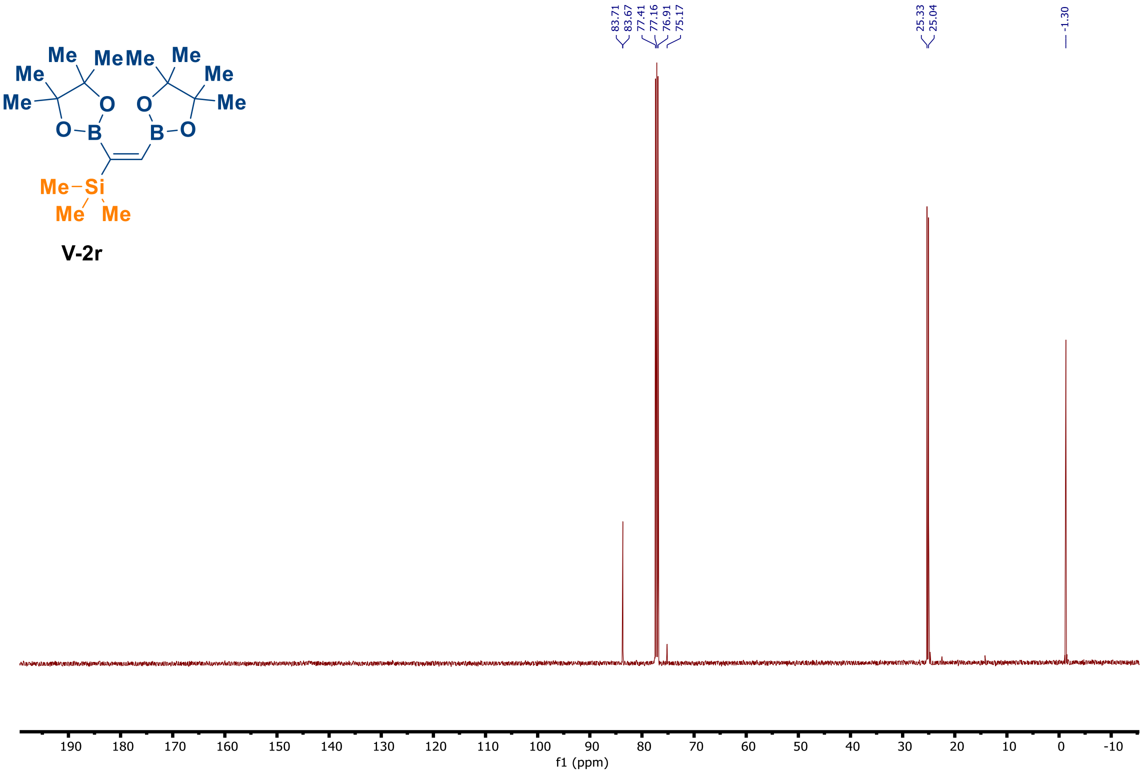

Supplementary Figure 29. ^{13}C NMR (126 MHz, $CDCl_3$) of compound (**V-2r**).

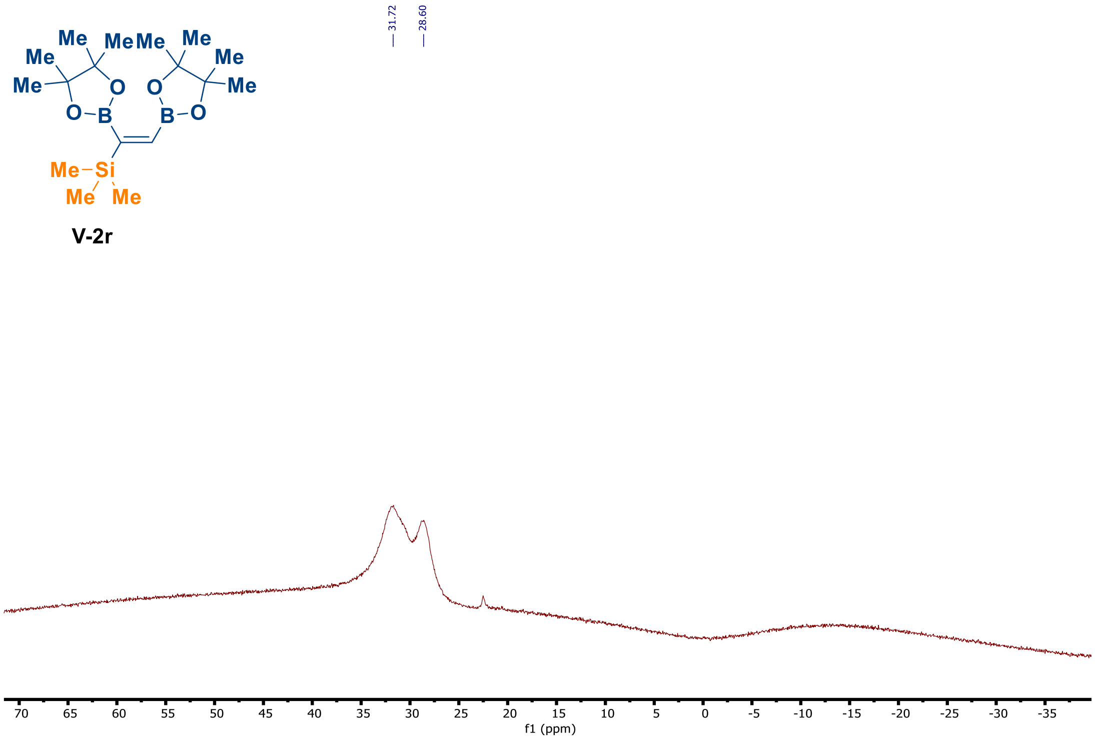

Supplementary Figure 30. ^{11}B NMR (161 MHz, $CDCl_3$) of compound (**V-2r**).

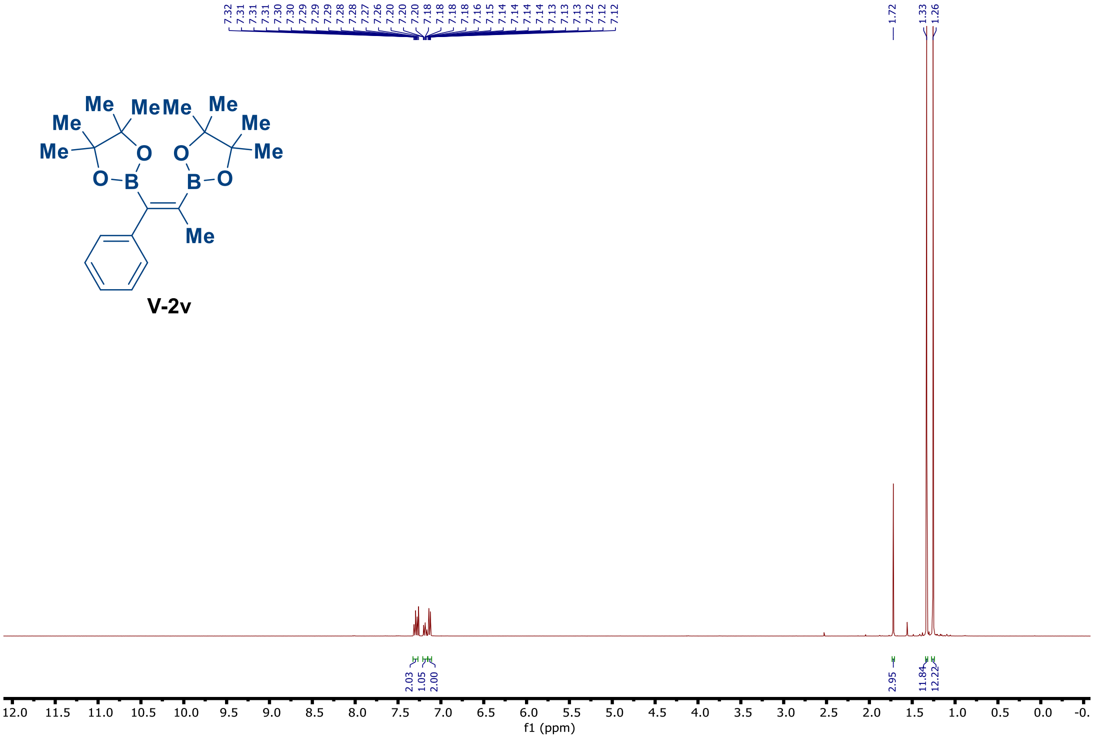

Supplementary Figure 31. ^{1}H NMR (400 MHz, $CDCl_3$) of compound (**V-2v**).

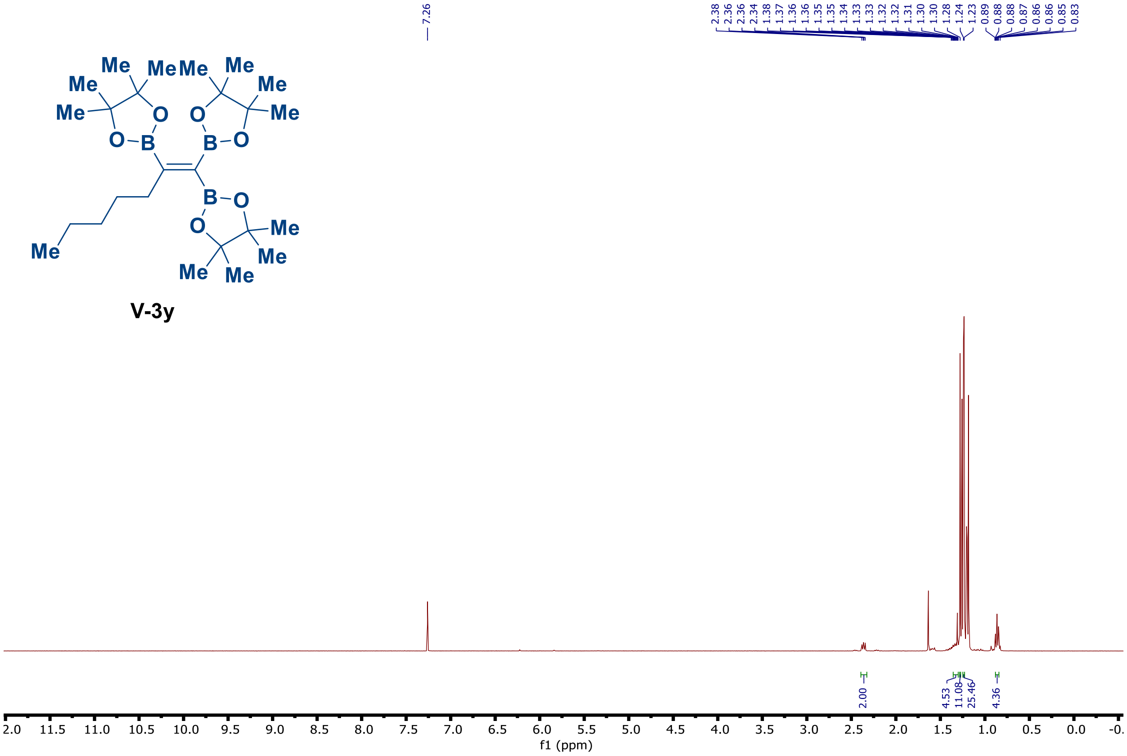

Supplementary Figure 32. ^{1}H NMR (400 MHz, $CDCl_3$) of compound (**V-3y**).

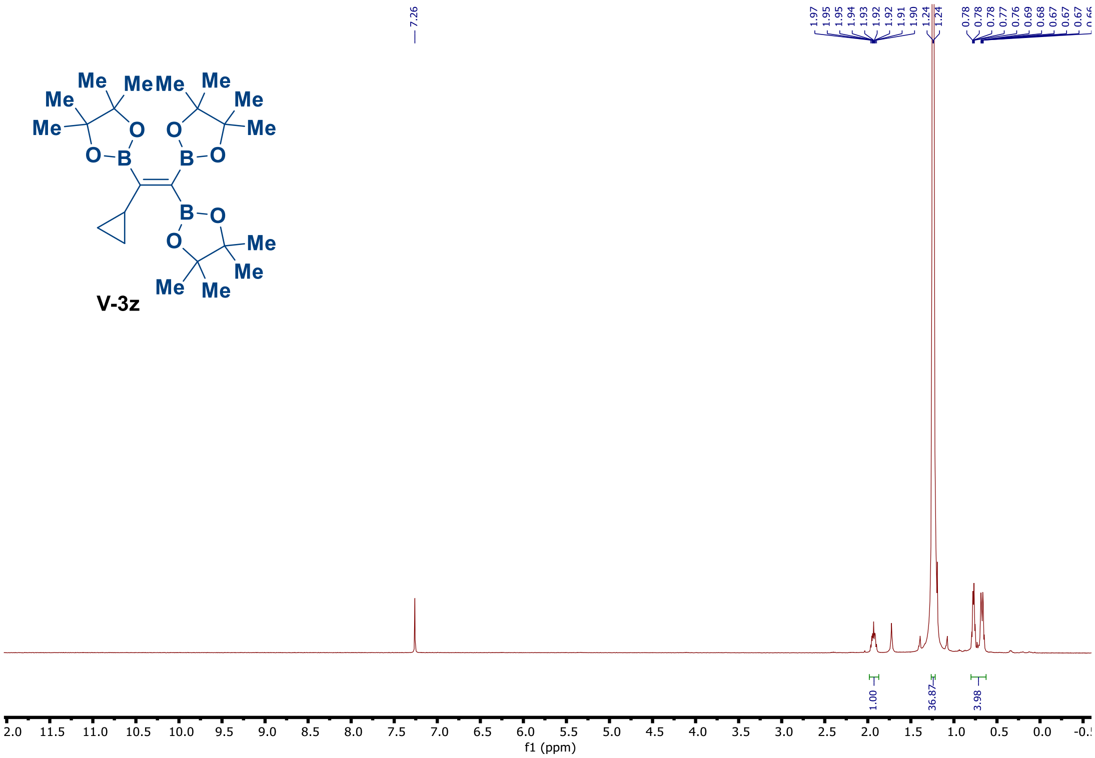

Supplementary Figure 33. ^{1}H NMR (400 MHz, $CDCl_3$) of compound (**V-3z**).

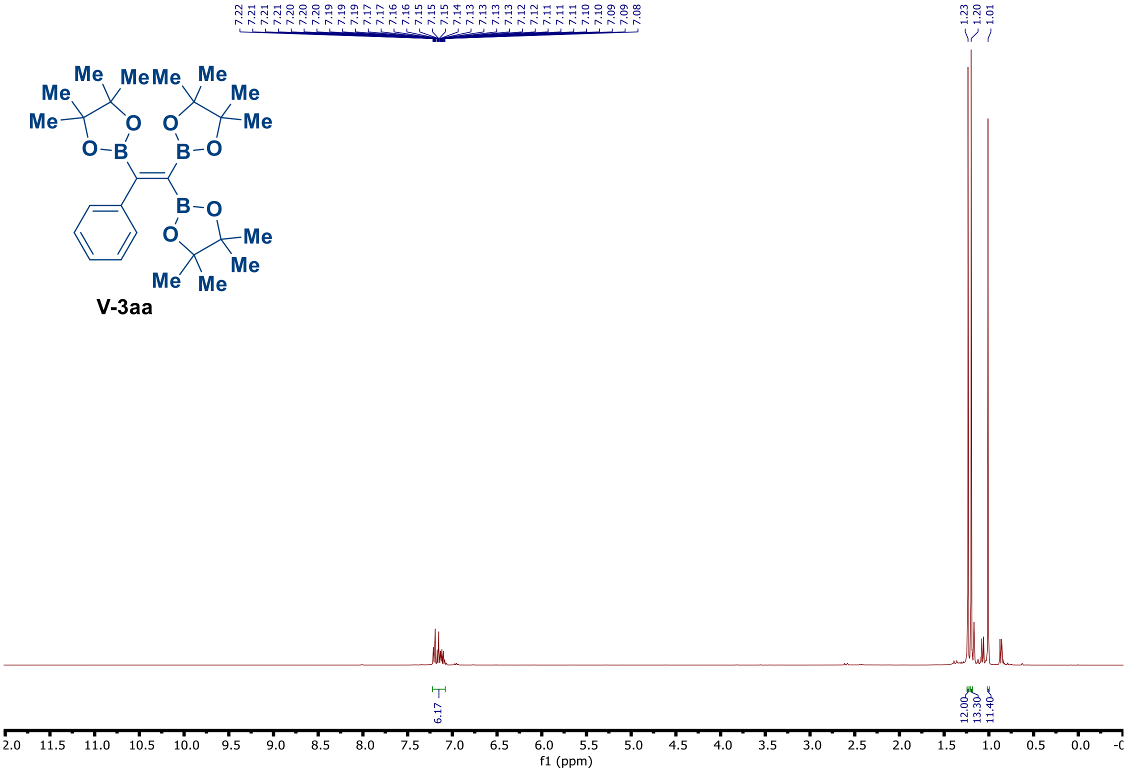

Supplementary Figure 34. ^{1}H NMR (400 MHz, $CDCl_3$) of compound (**V-3aa**).

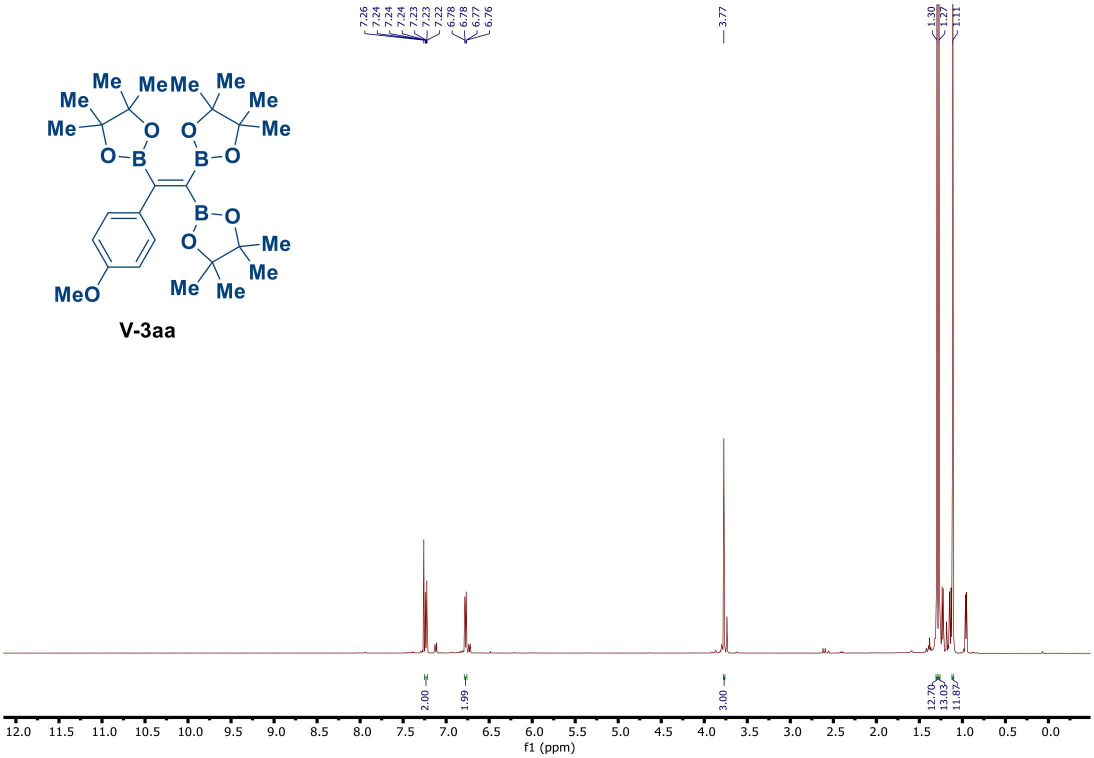

Supplementary Figure 35. ^{1}H NMR (400 MHz, $CDCl_3$) of compound (**V-3ab**).

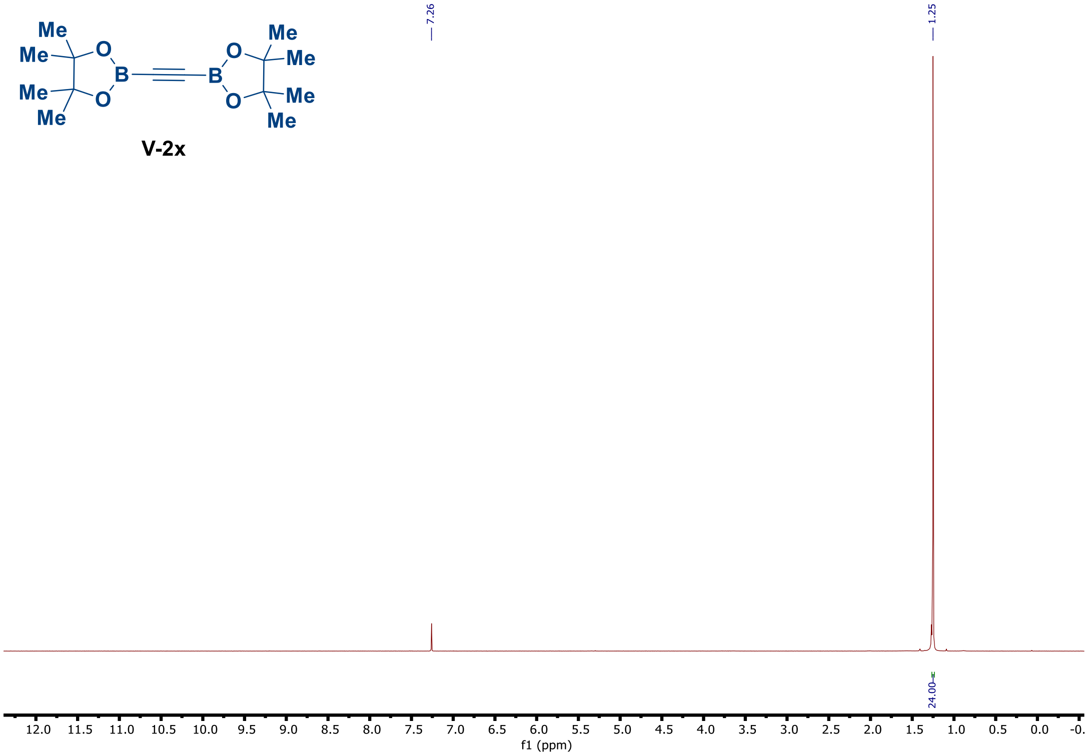

Supplementary Figure 36. ^{1}H NMR (400 MHz, $CDCl_3$) of compound (**V-2x**).

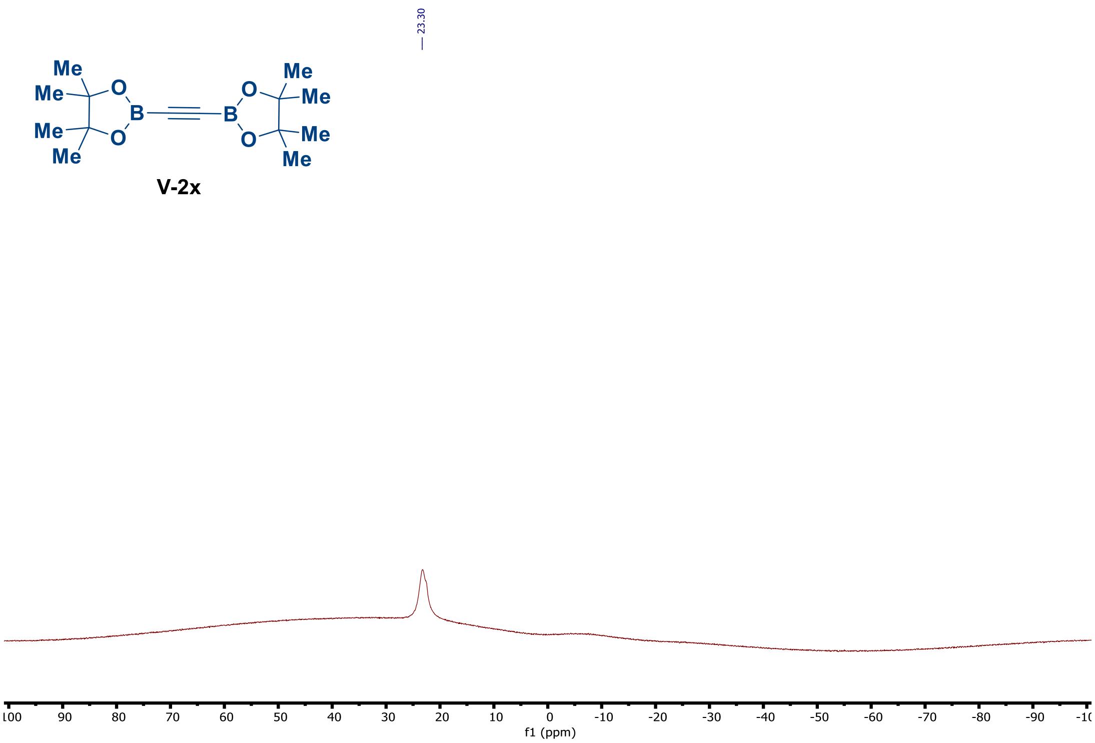

Supplementary Figure 37. ^{11}B NMR (128 MHz, $CDCl_3$) of compound (**V-2x**).

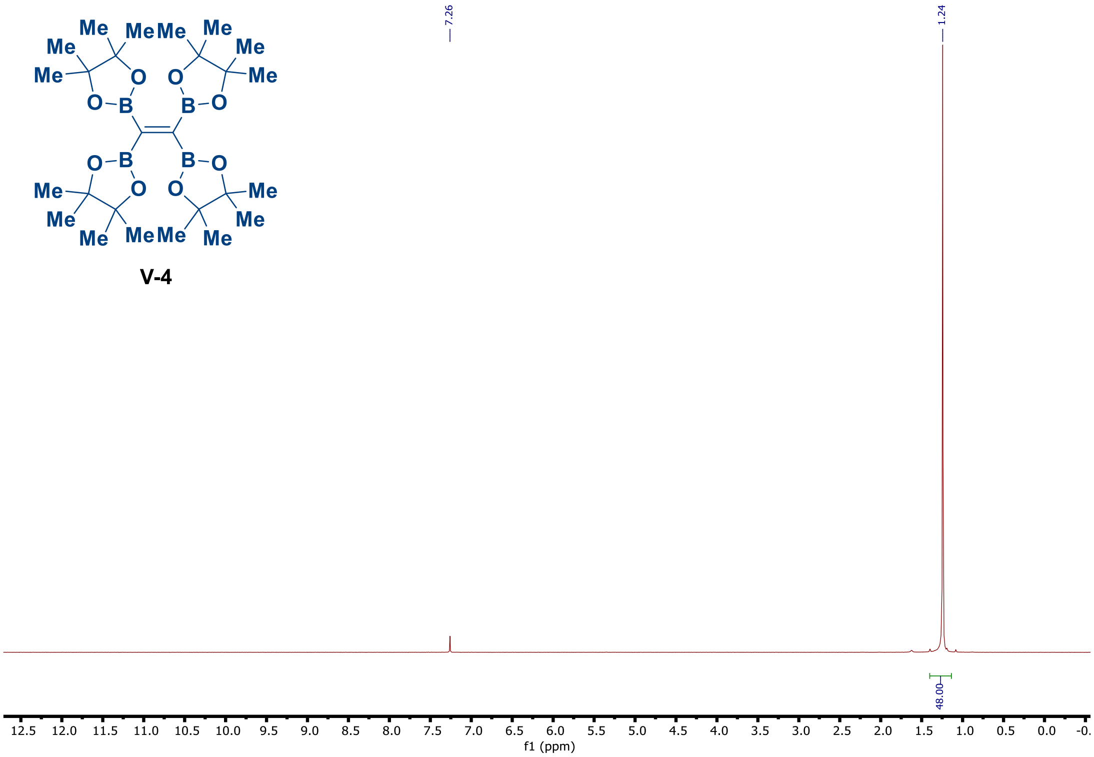

Supplementary Figure 38. ^{1}H NMR (400 MHz, $CDCl_3$) of compound (**V-4**).

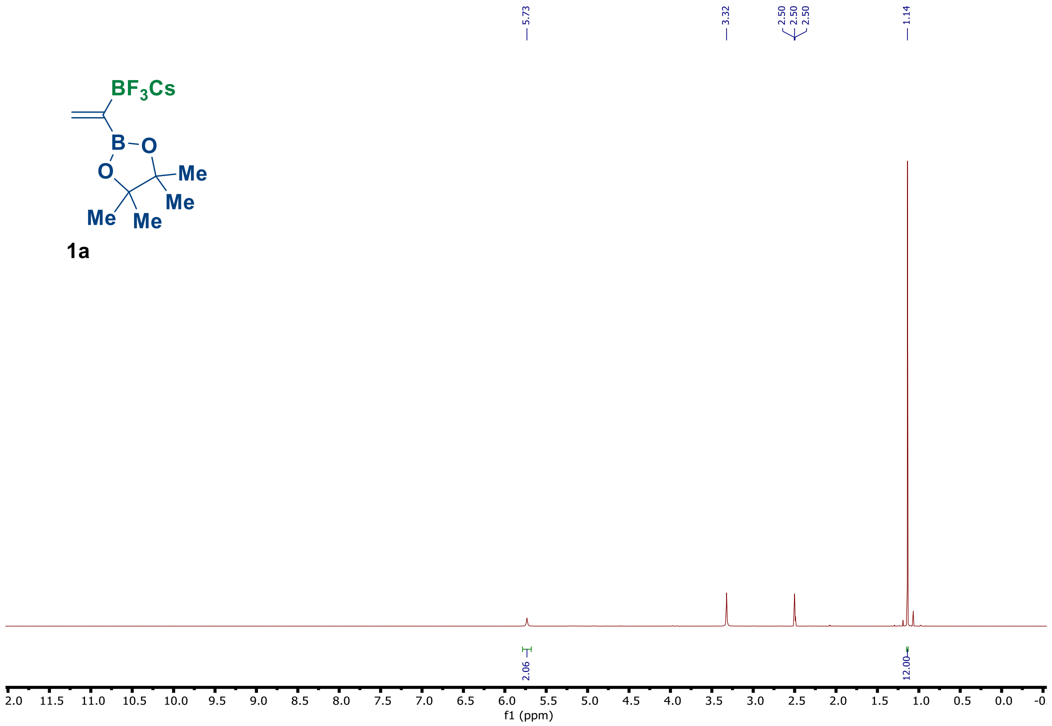

Supplementary Figure 39. ^{1}H NMR (400 MHz, DMSO-d_6) of compound (**1a**).

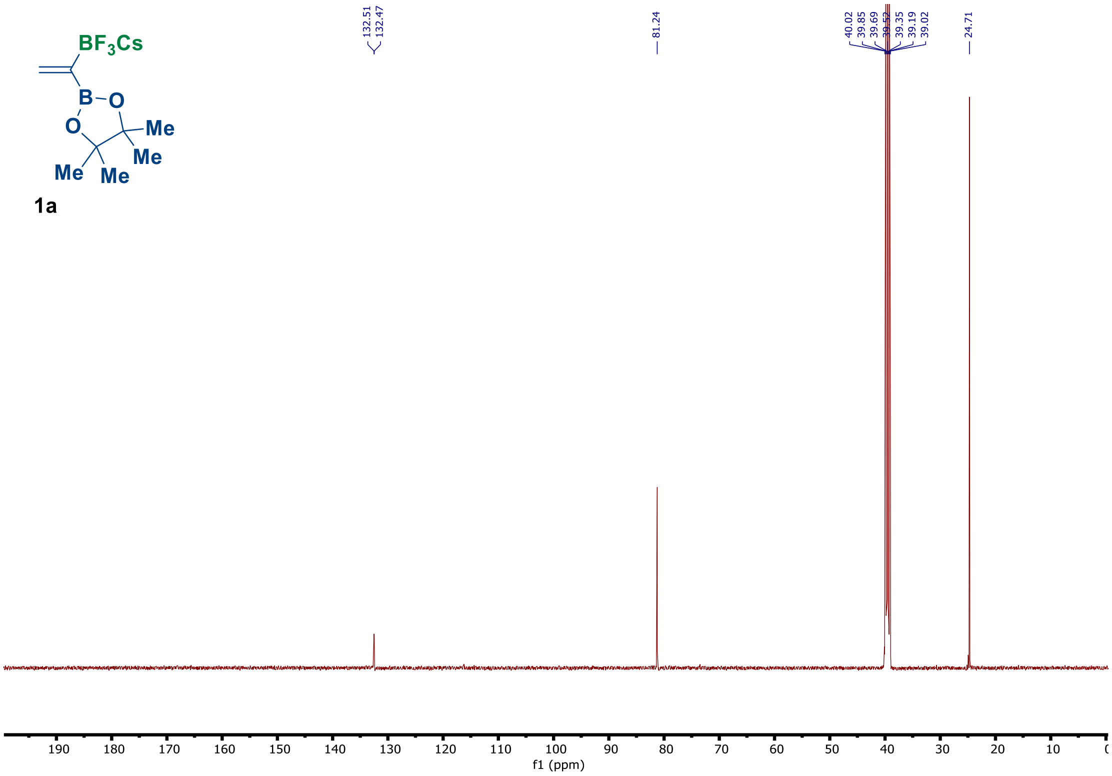

Supplementary Figure 40. ^{13}C NMR (101 MHz, DMSO-d_6) of compound (**1a**).

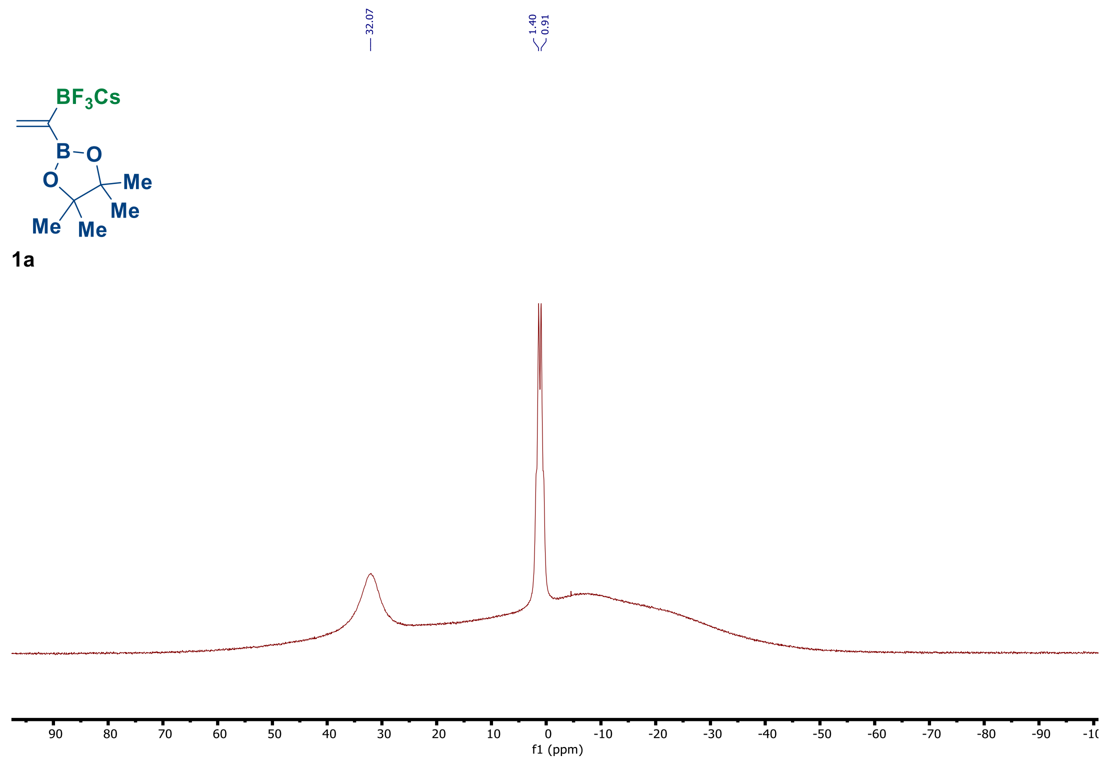

Supplementary Figure 41. ^{11}B NMR (128 MHz, DMSO-d_6) of compound (**1a**).

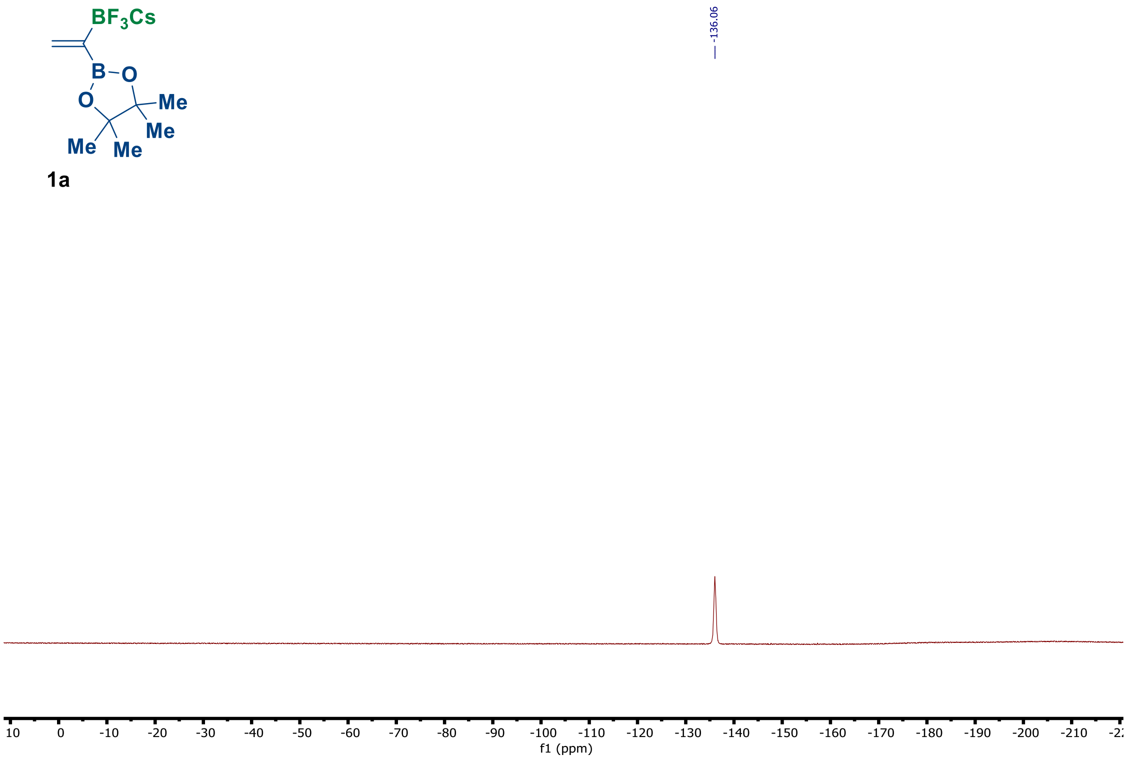

Supplementary Figure 42. ^{19}F NMR (376 MHz, DMSO-d_6) of compound (**1a**).

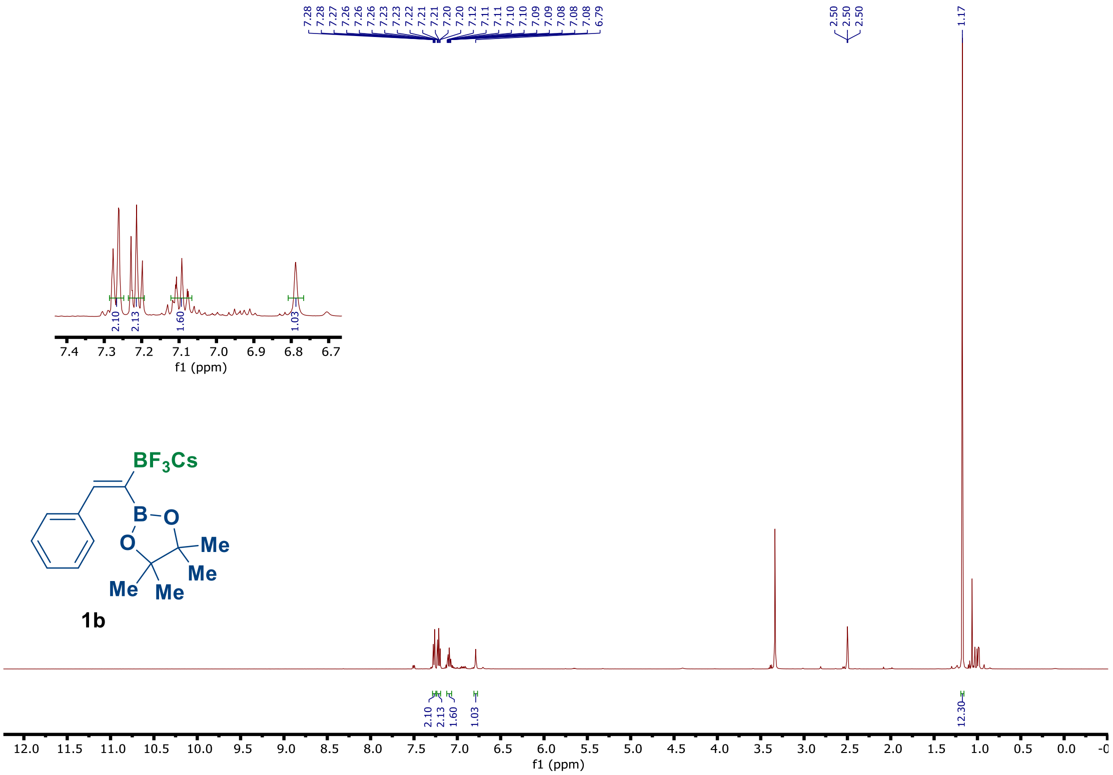

Supplementary Figure 43. ^{1}H NMR (400 MHz, DMSO-d_6) of compound (**1b**).

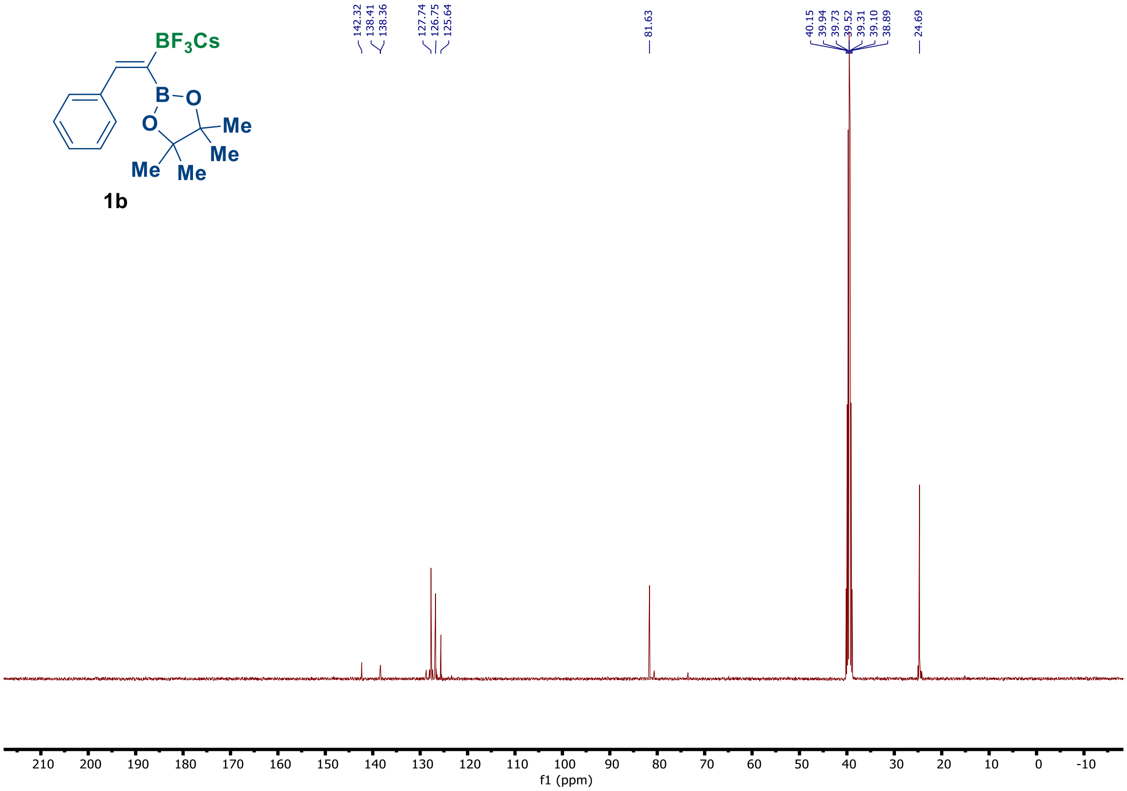

Supplementary Figure 44. ^{13}C NMR (101 MHz, DMSO-d_6) of compound (**1b**).

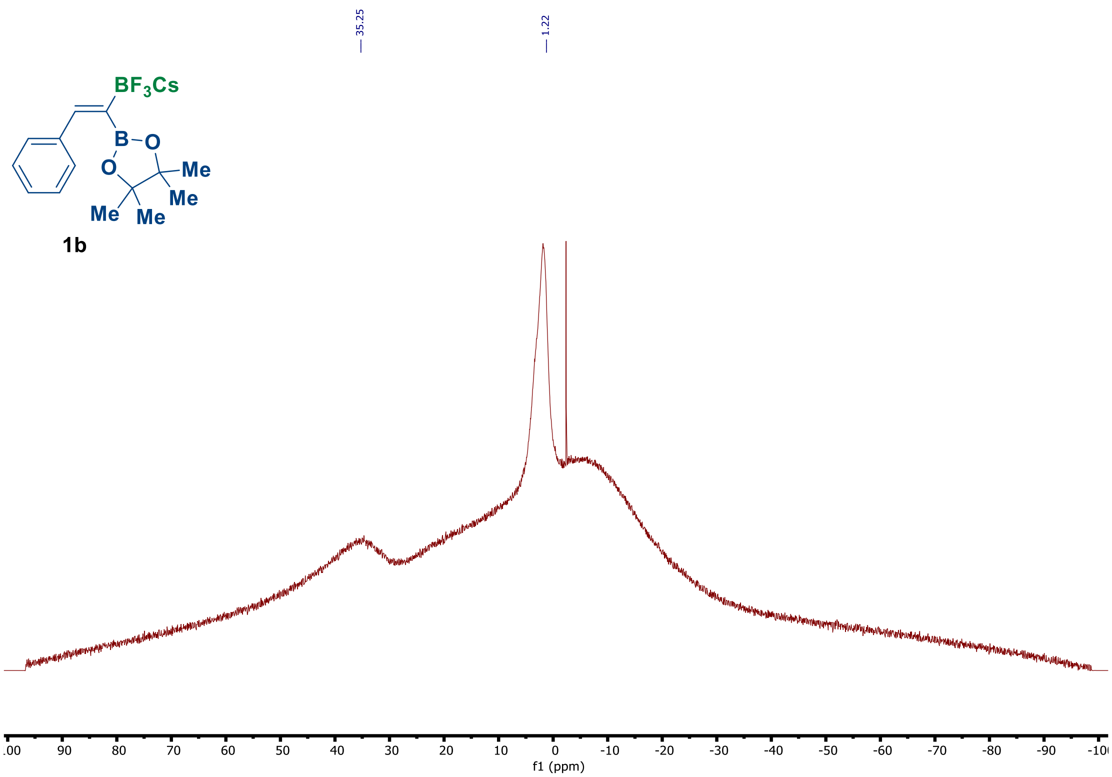

Supplementary Figure 45. ^{11}B NMR (128 MHz, DMSO-d_6) of compound (**1b**).

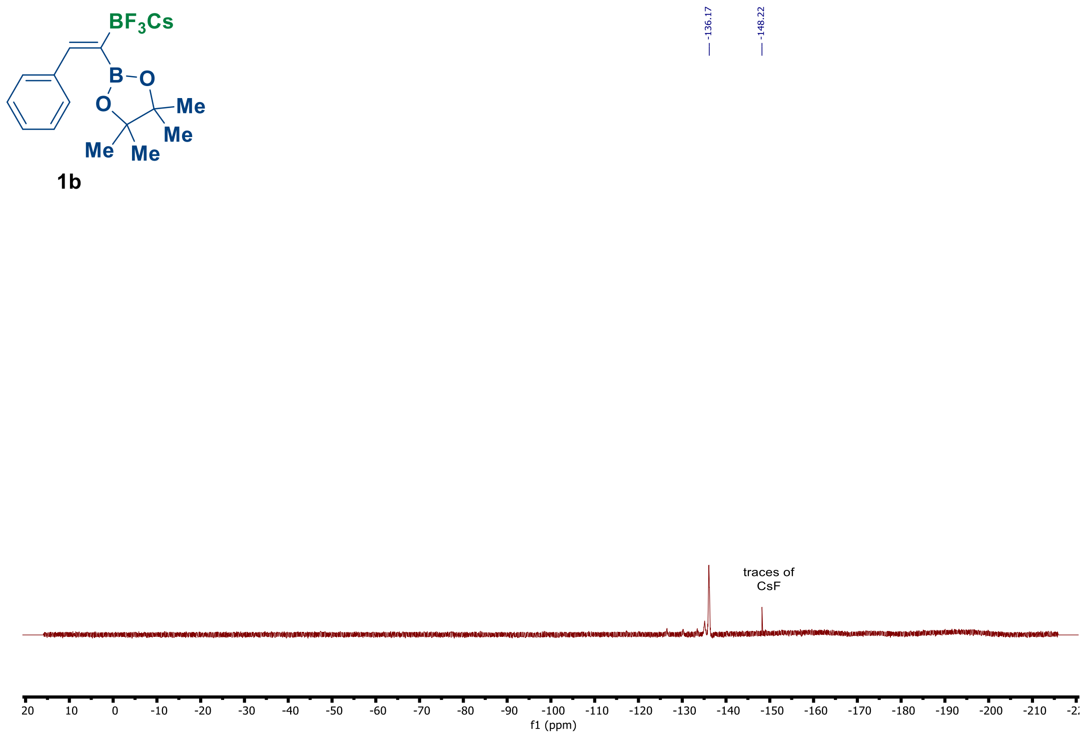

Supplementary Figure 46. ^{19}F NMR (376 MHz, DMSO-d_6) of compound (**1b**).

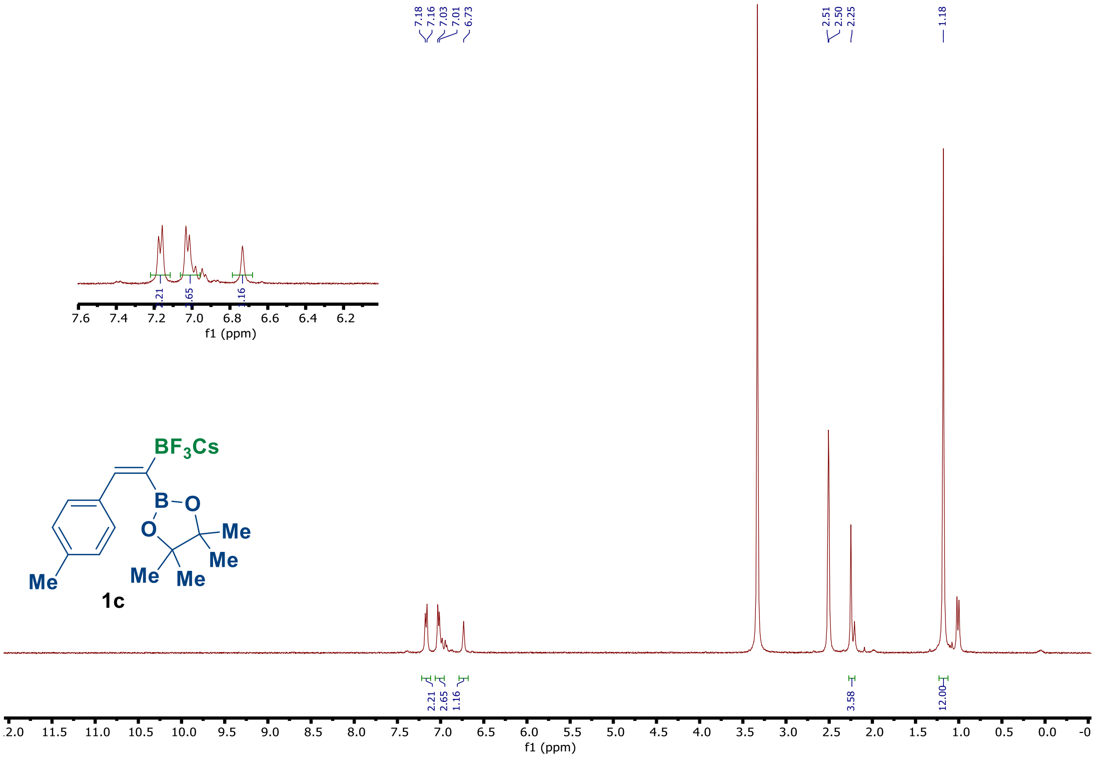

Supplementary Figure 47. ^{1}H NMR (400 MHz, DMSO-d_6) of compound (**1c**).

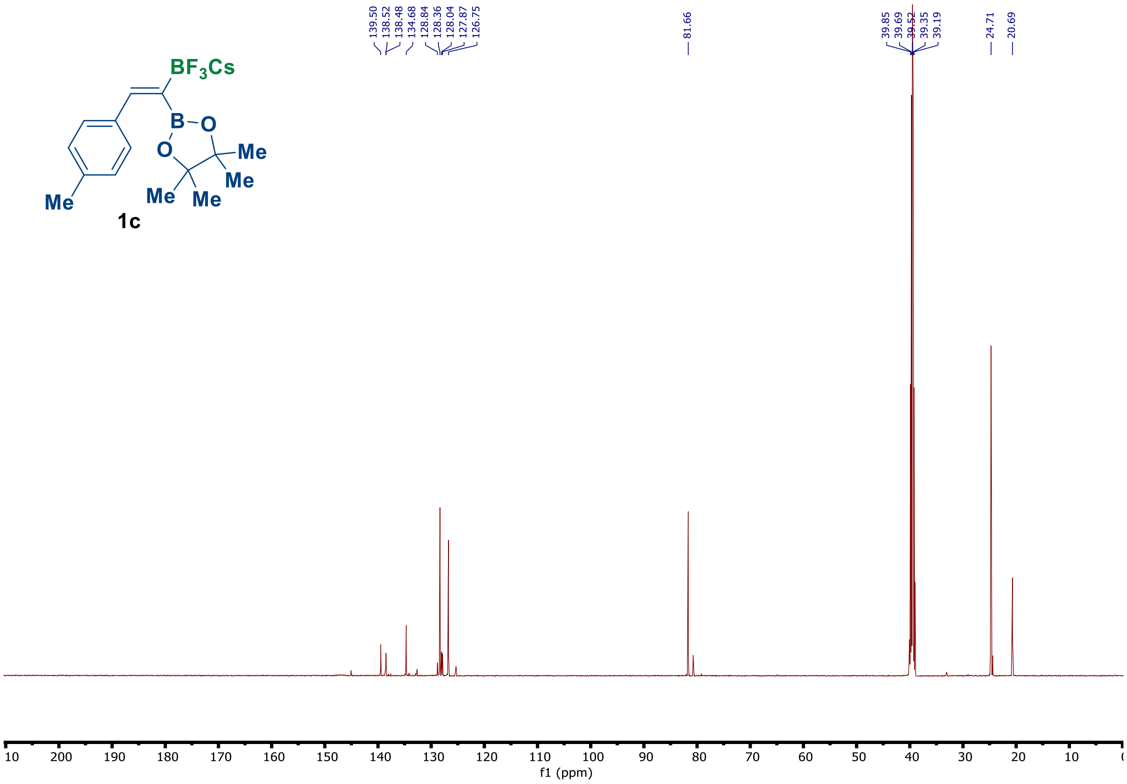

Supplementary Figure 48. ^{13}C NMR (101 MHz, DMSO-d_6) of compound (**1c**).

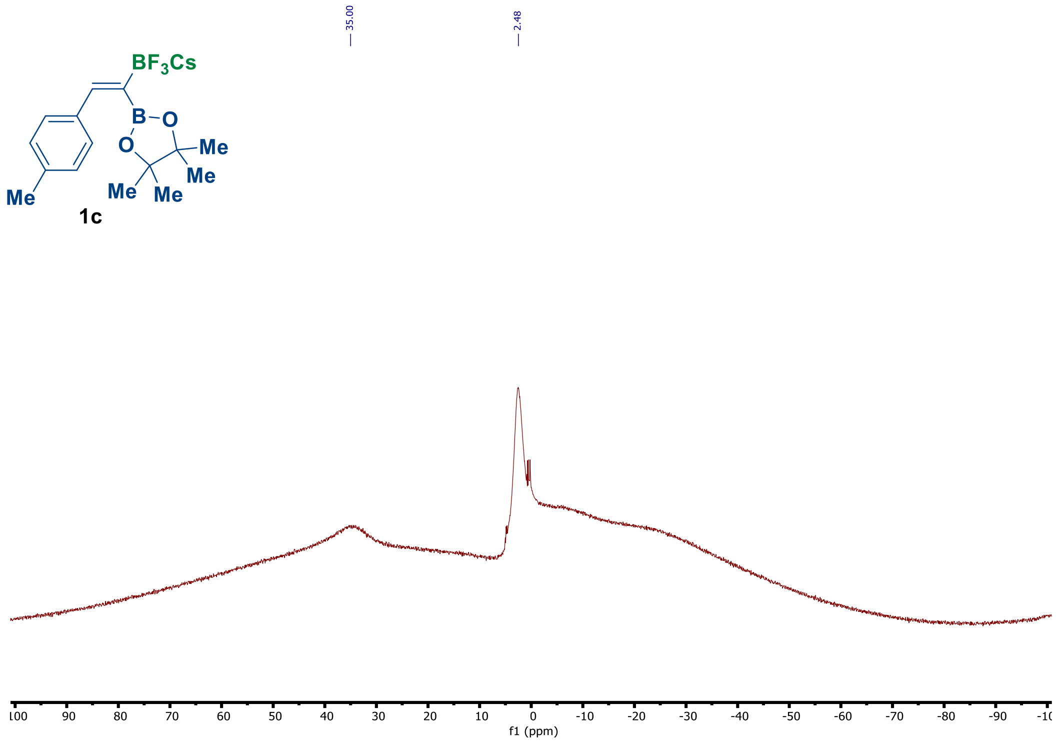

Supplementary Figure 49. ^{11}B NMR (128 MHz, DMSO-d_6) of compound (**1c**).

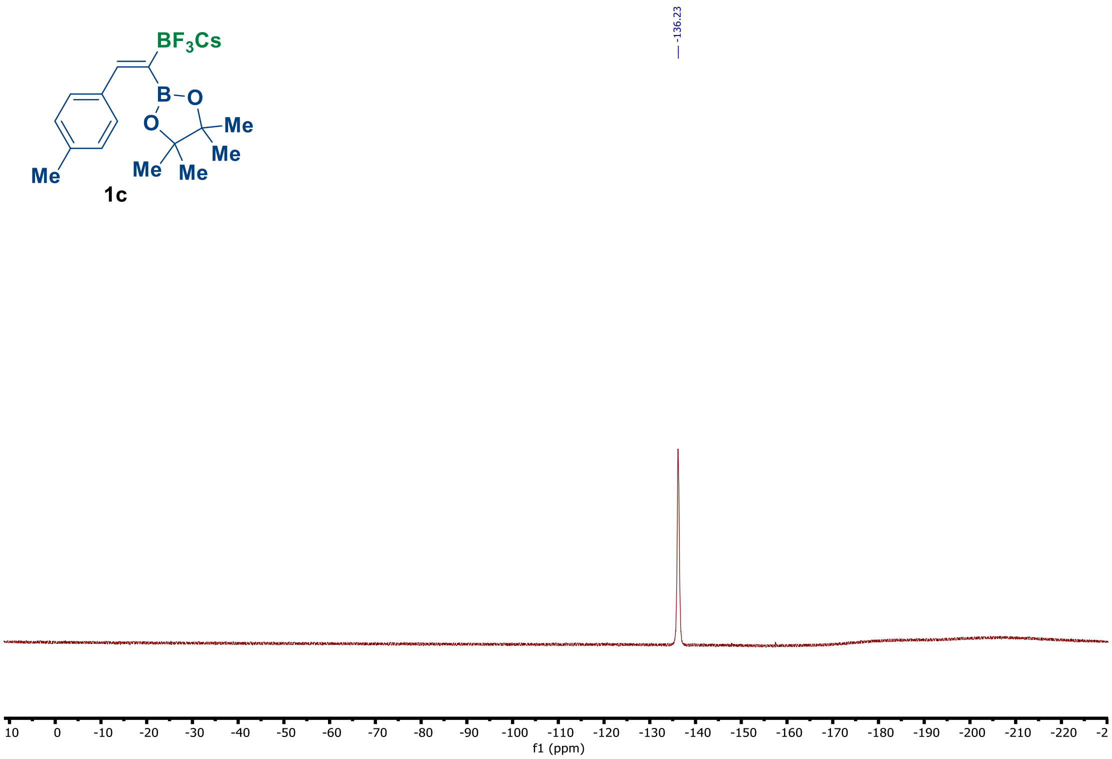

Supplementary Figure 50. ^{19}F NMR (376 MHz, DMSO-d_6) of compound (**1c**).

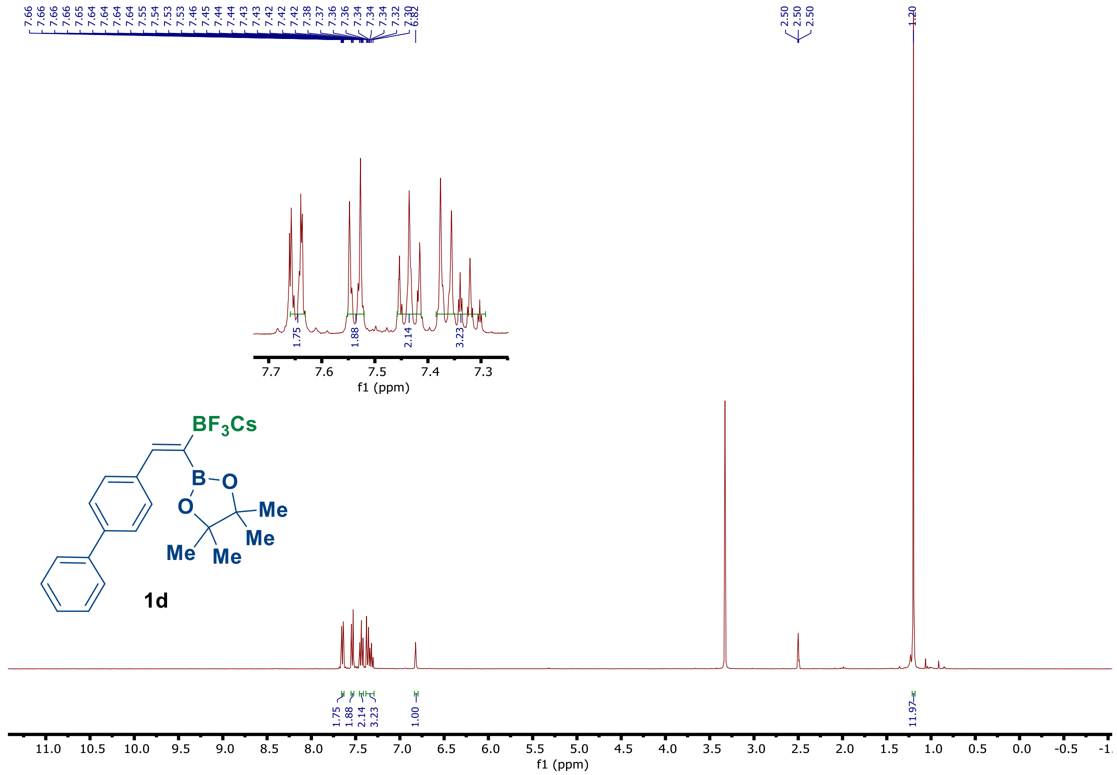

Supplementary Figure 51. ^{1}H NMR (400 MHz, DMSO-d_6) of compound (**1d**).

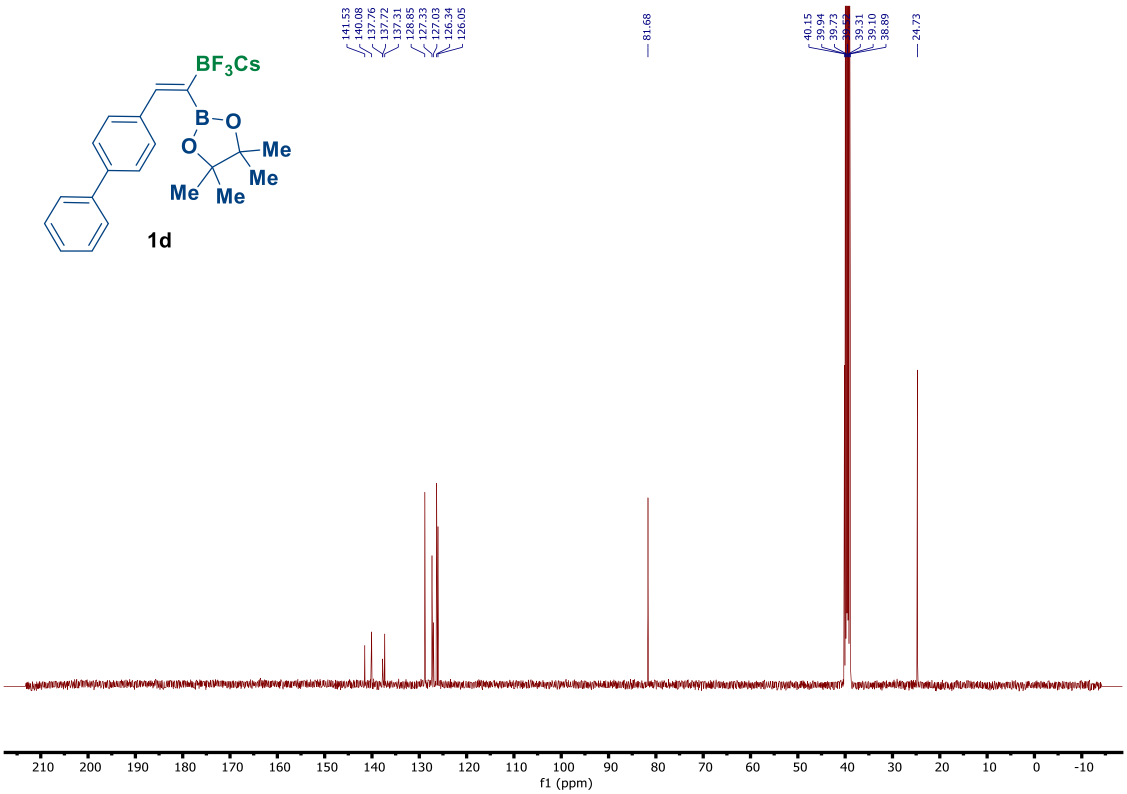

Supplementary Figure 52. ^{13}C NMR (101 MHz, DMSO-d_6) of compound (**1d**).

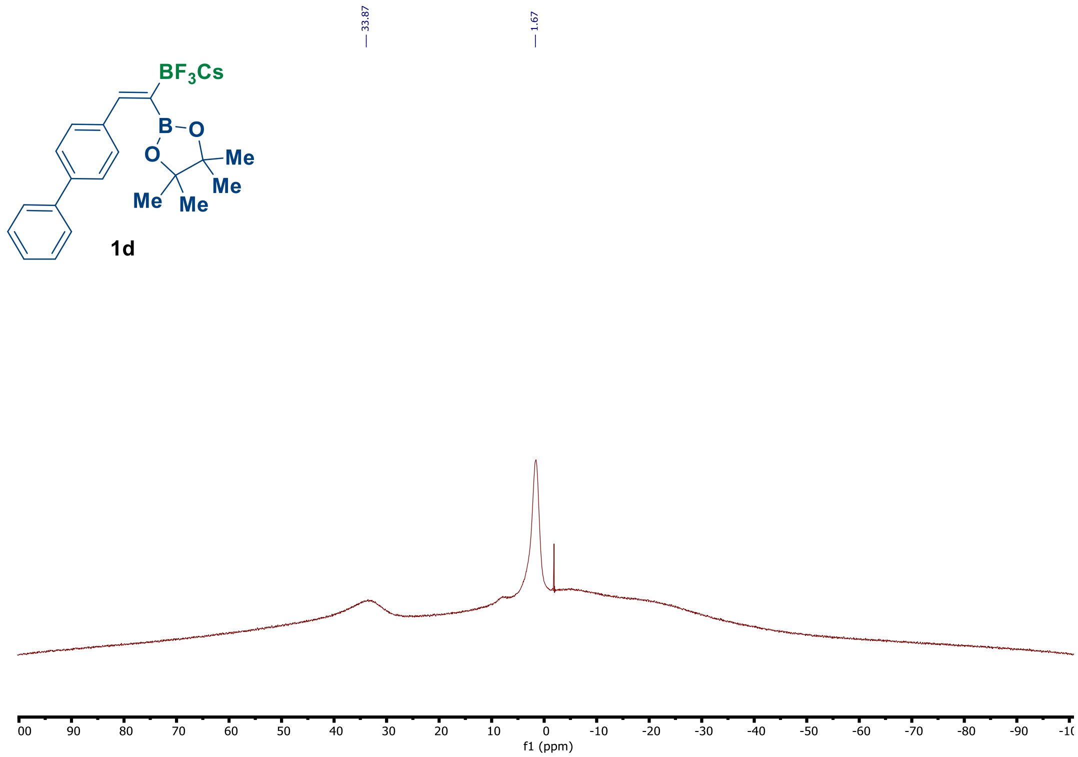

Supplementary Figure 53. ^{11}B NMR (128 MHz, DMSO-d_6) of compound (**1d**).

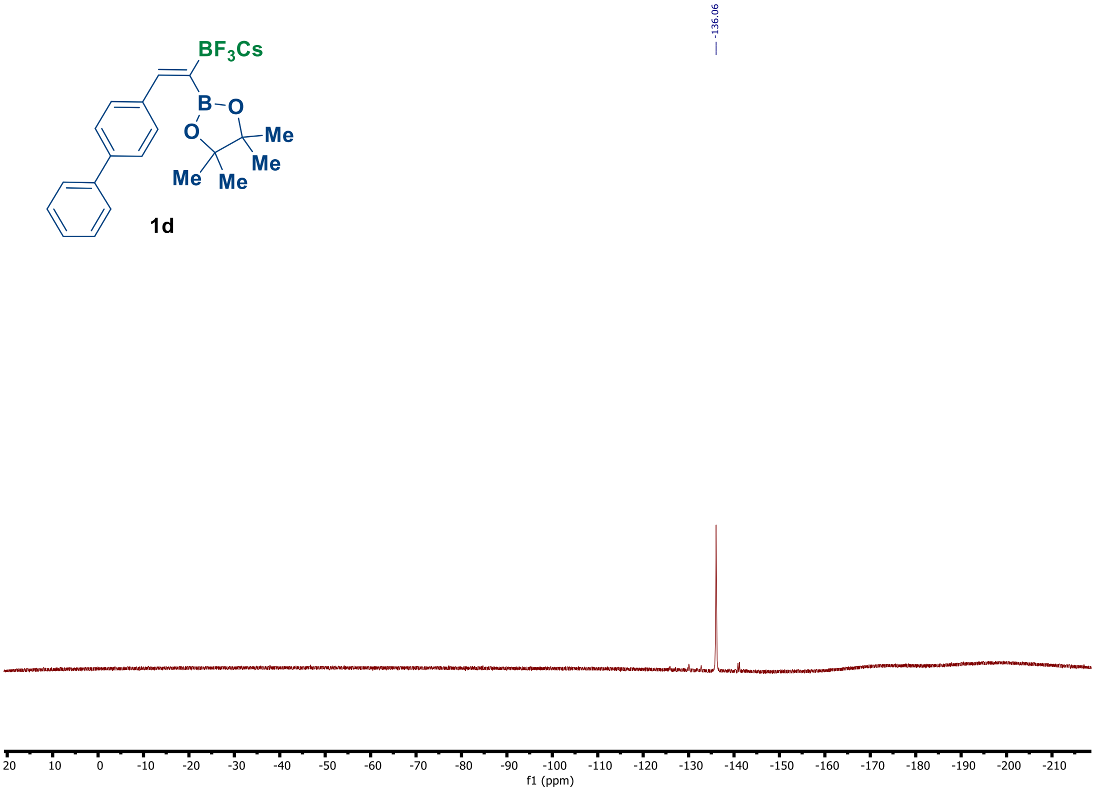

Supplementary Figure 54. ^{19}F NMR (376 MHz, DMSO-d_6) of compound (**1d**).

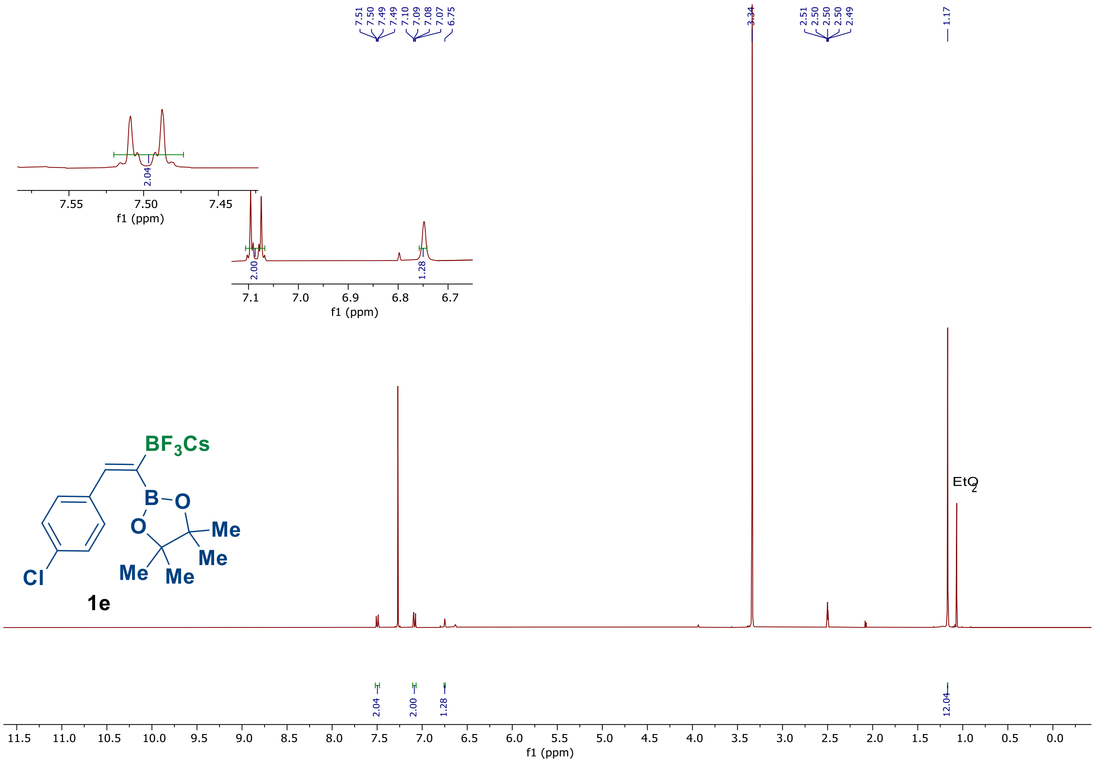

Supplementary Figure 55. ^{1}H NMR (400 MHz, DMSO-d_6) of compound (**1e**).

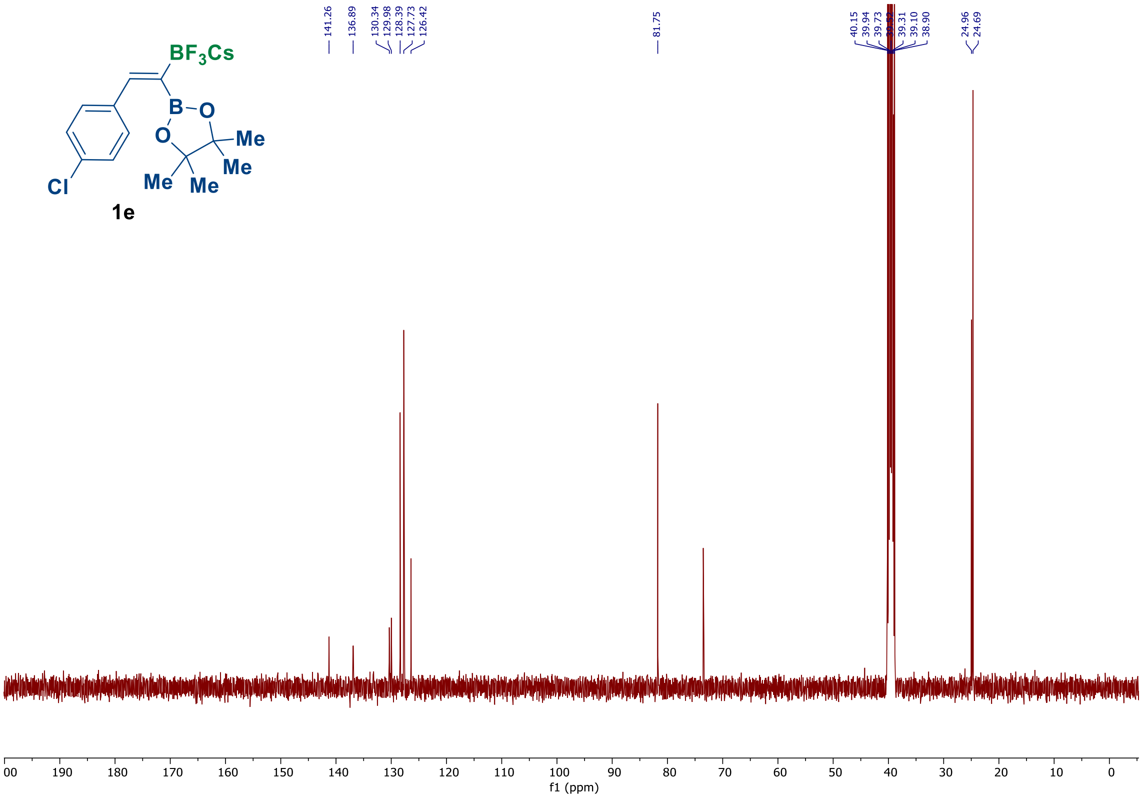

Supplementary Figure 56. ^{13}C NMR (101 MHz, DMSO-d_6) of compound (**1e**) .

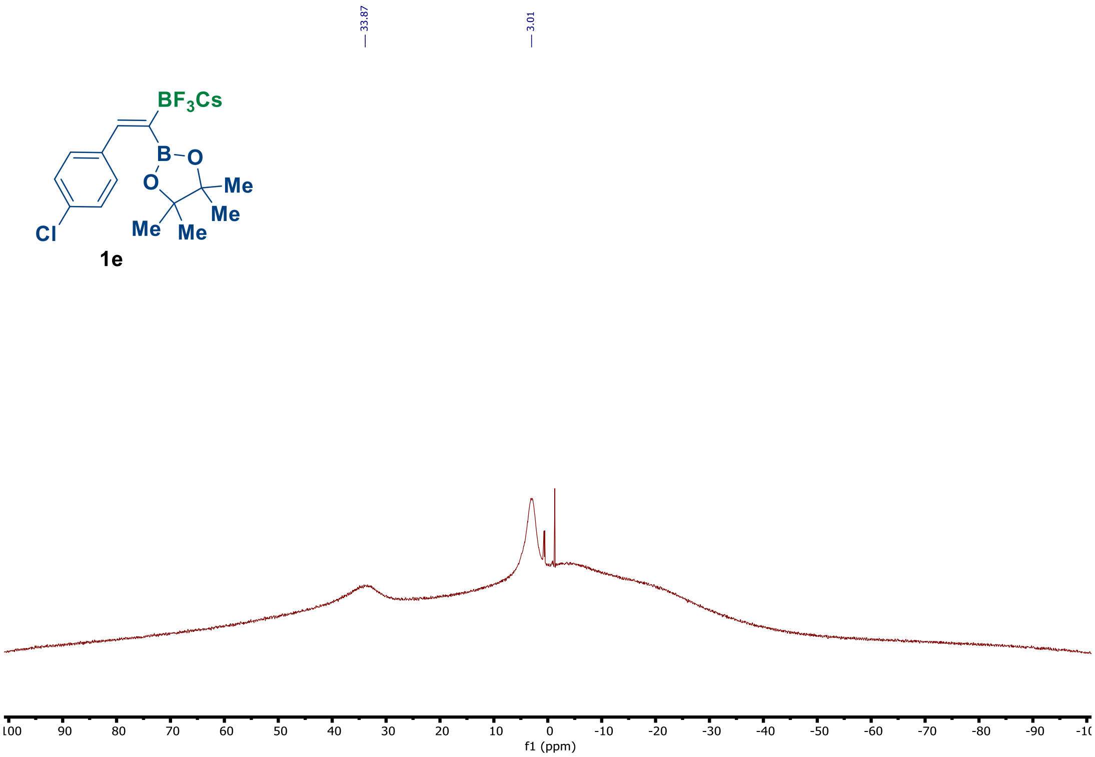

Supplementary Figure 57. ^{11}B NMR (128 MHz, DMSO-d_6) of compound (**1e**).

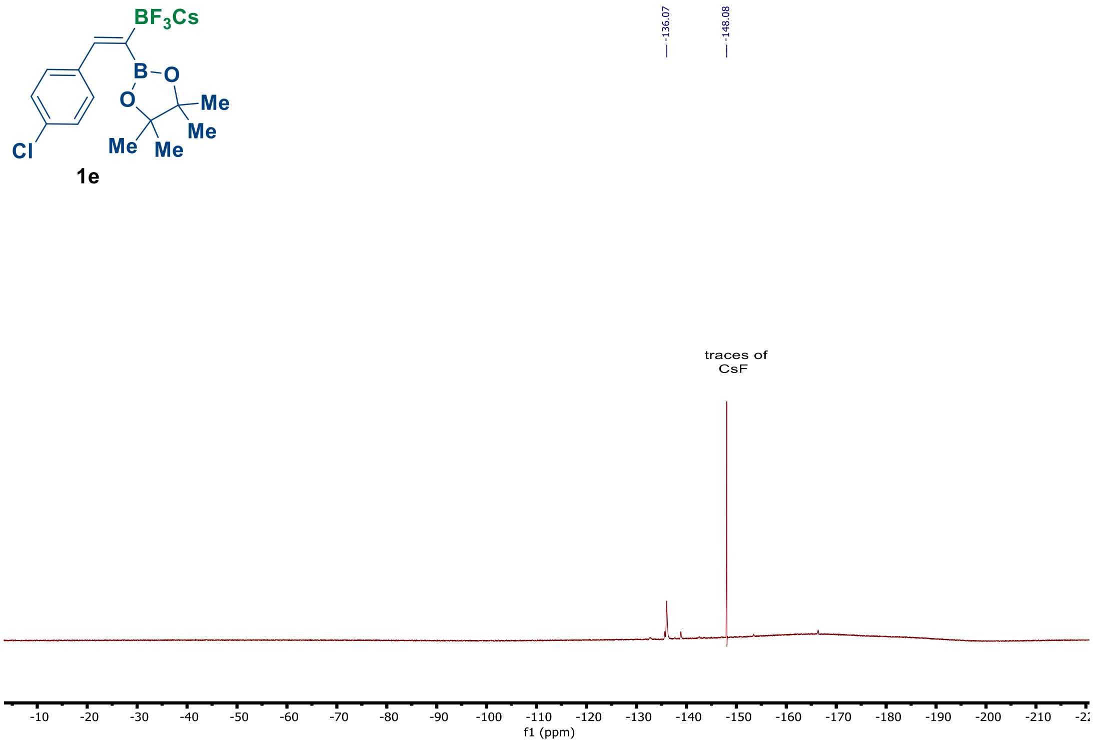

Supplementary Figure 58. ^{19}F NMR (376 MHz, DMSO-d_6) of compound (**1e**).

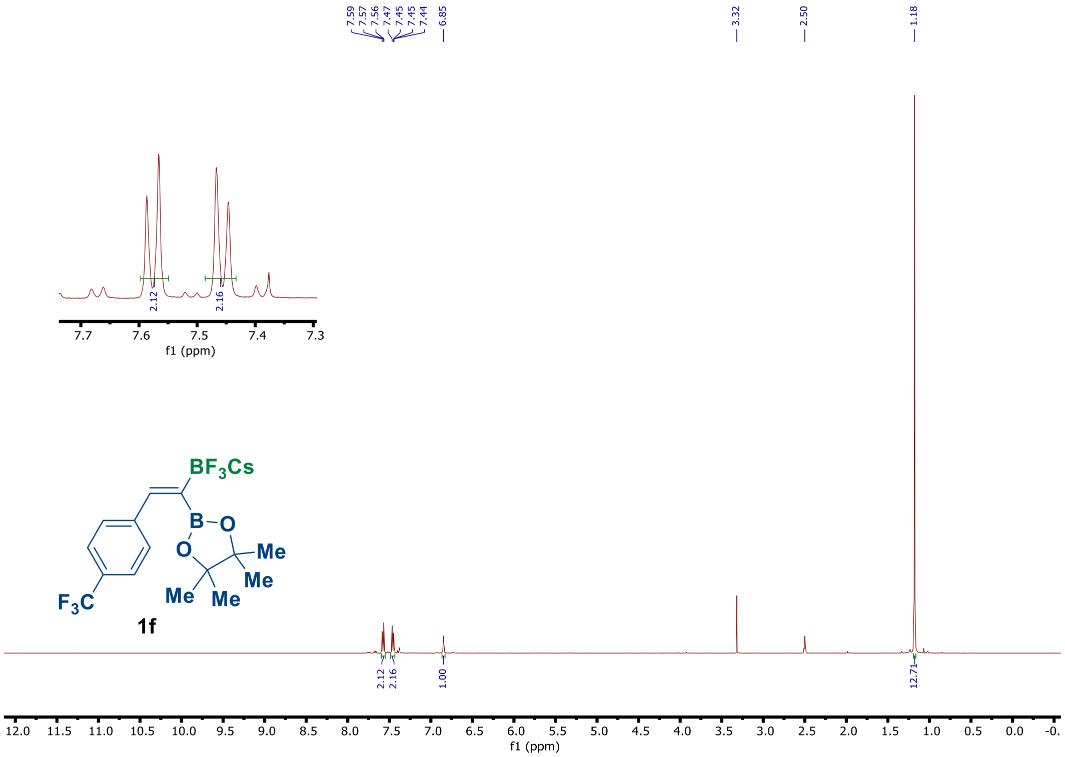

Supplementary Figure 59. ^{1}H NMR (400 MHz, DMSO-d_6) of compound (**1f**).

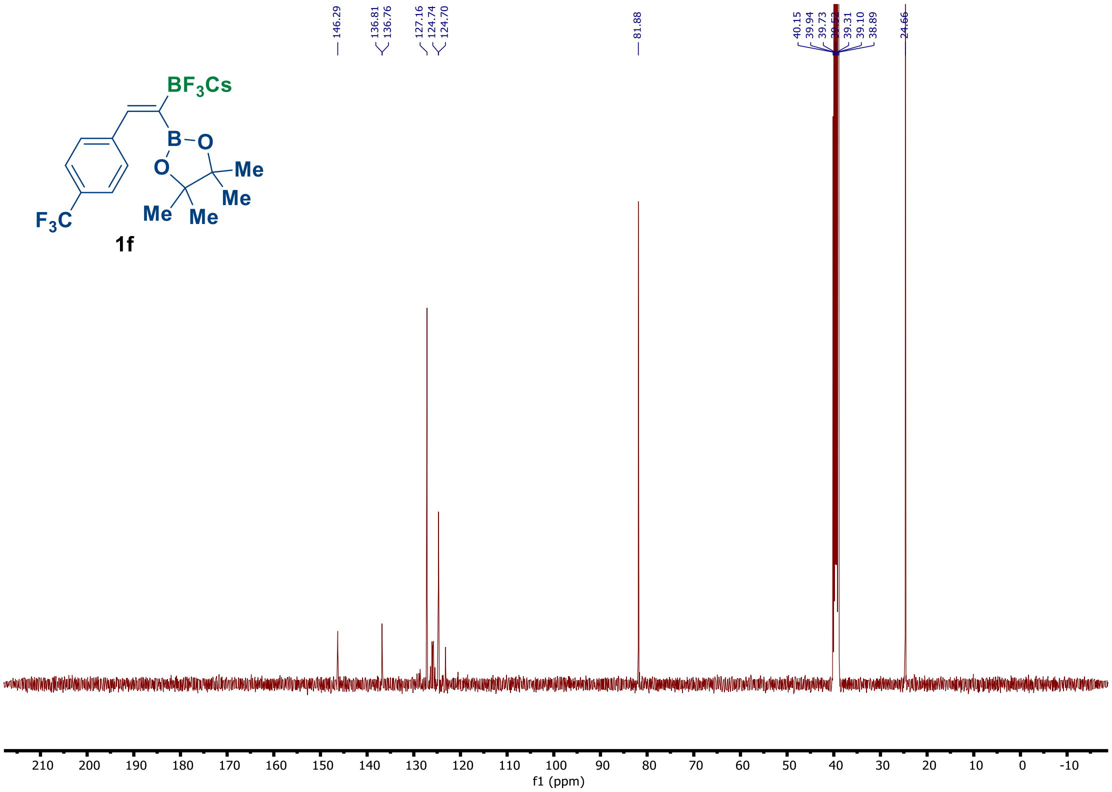

Supplementary Figure 60. ^{13}C NMR (101 MHz, DMSO-d_6) of compound (**1f**).

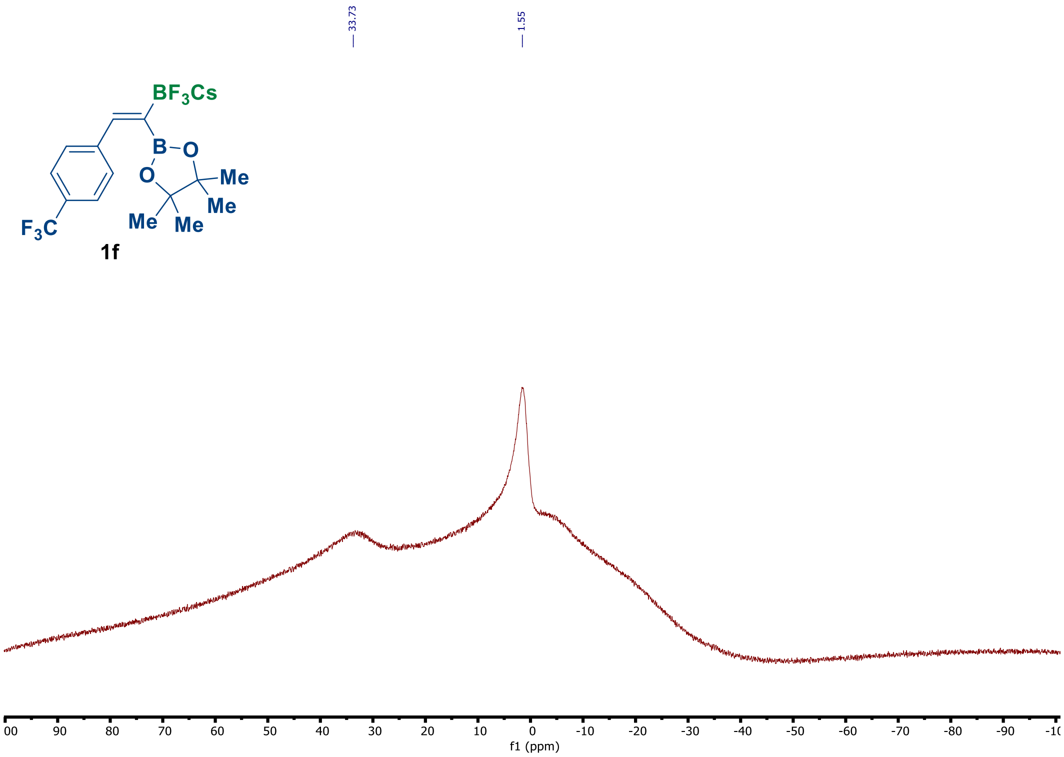

Supplementary Figure 61. ^{11}B NMR (128 MHz, DMSO-d_6) of compound (**1f**).

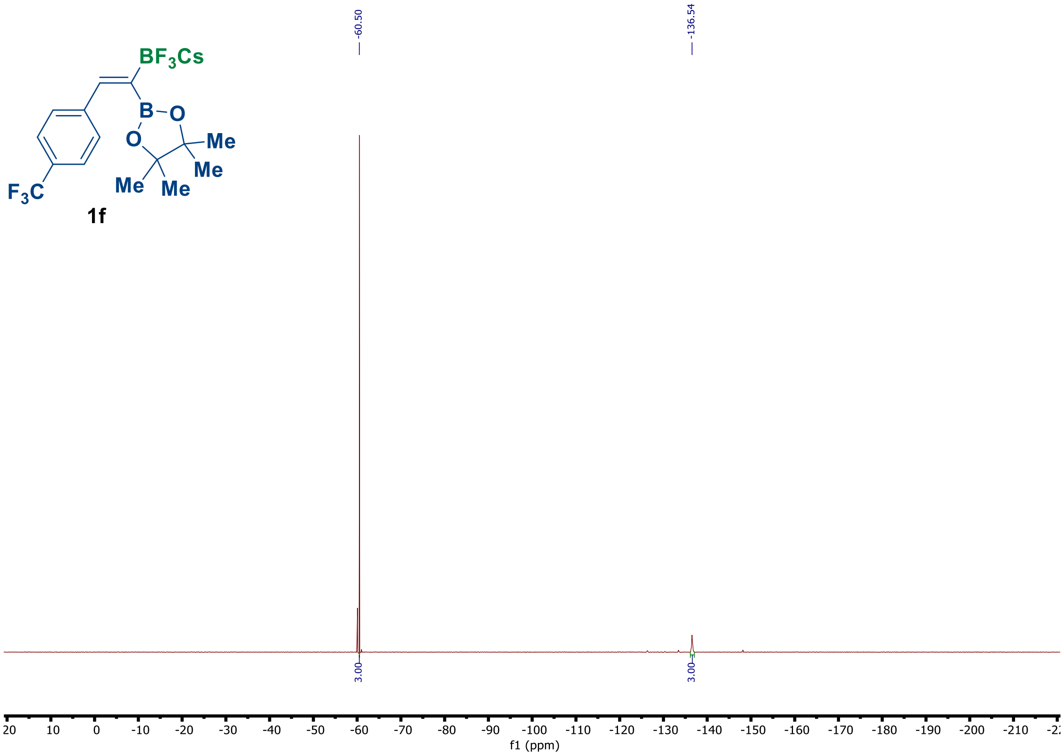

Supplementary Figure 62. ^{19}F NMR (376 MHz, DMSO-d_6) of compound (**1f**).

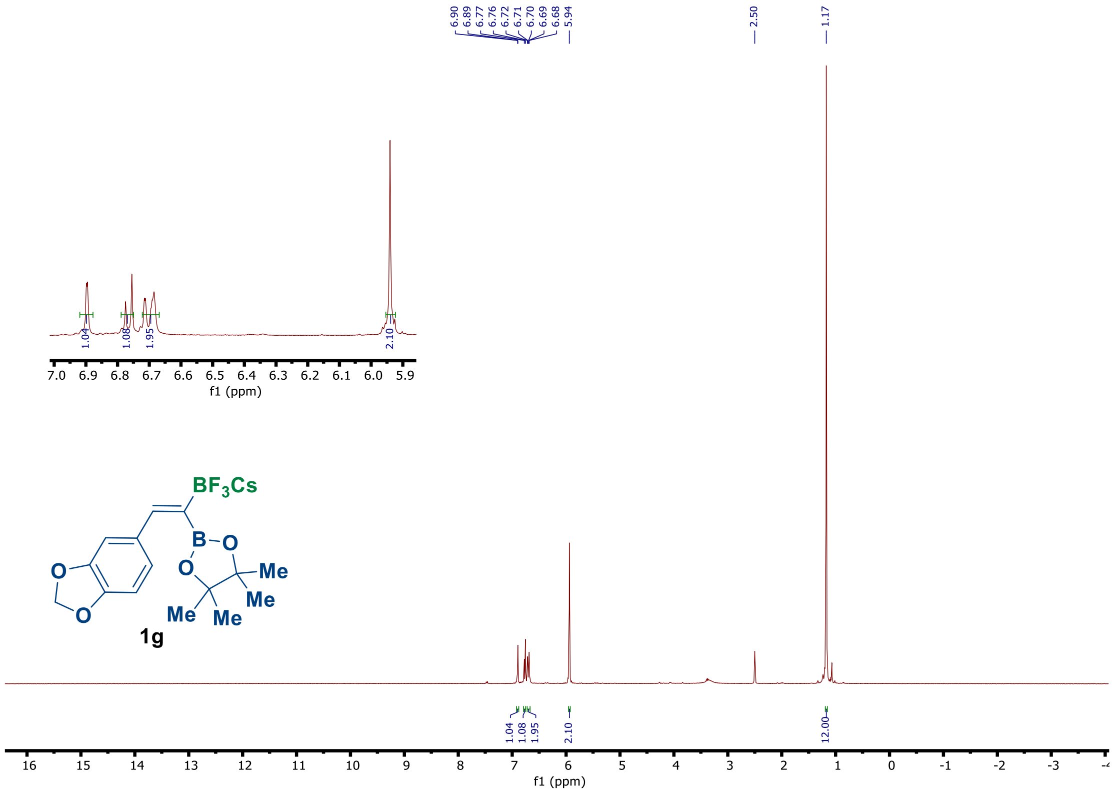

Supplementary Figure 63. ^{1}H NMR (400 MHz, DMSO-d_6) of compound (**1g**).

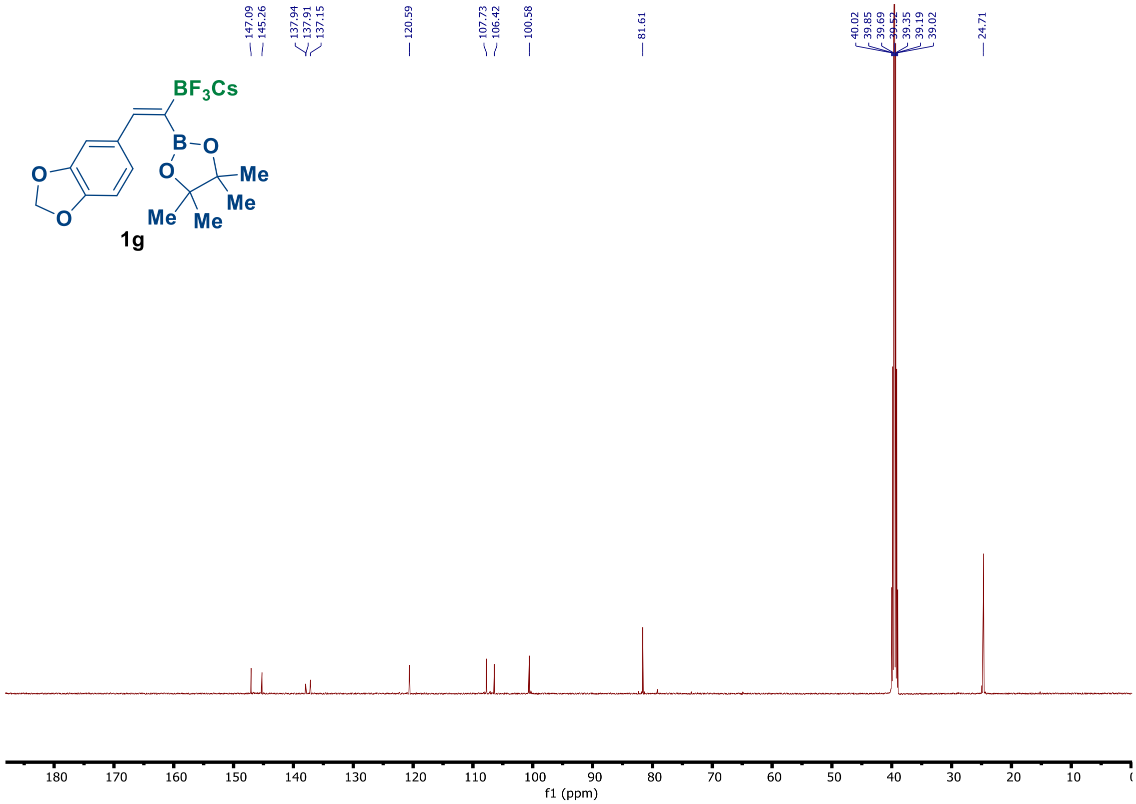

Supplementary Figure 64. ^{13}C NMR (101 MHz, DMSO-d_6) of compound (**1g**).

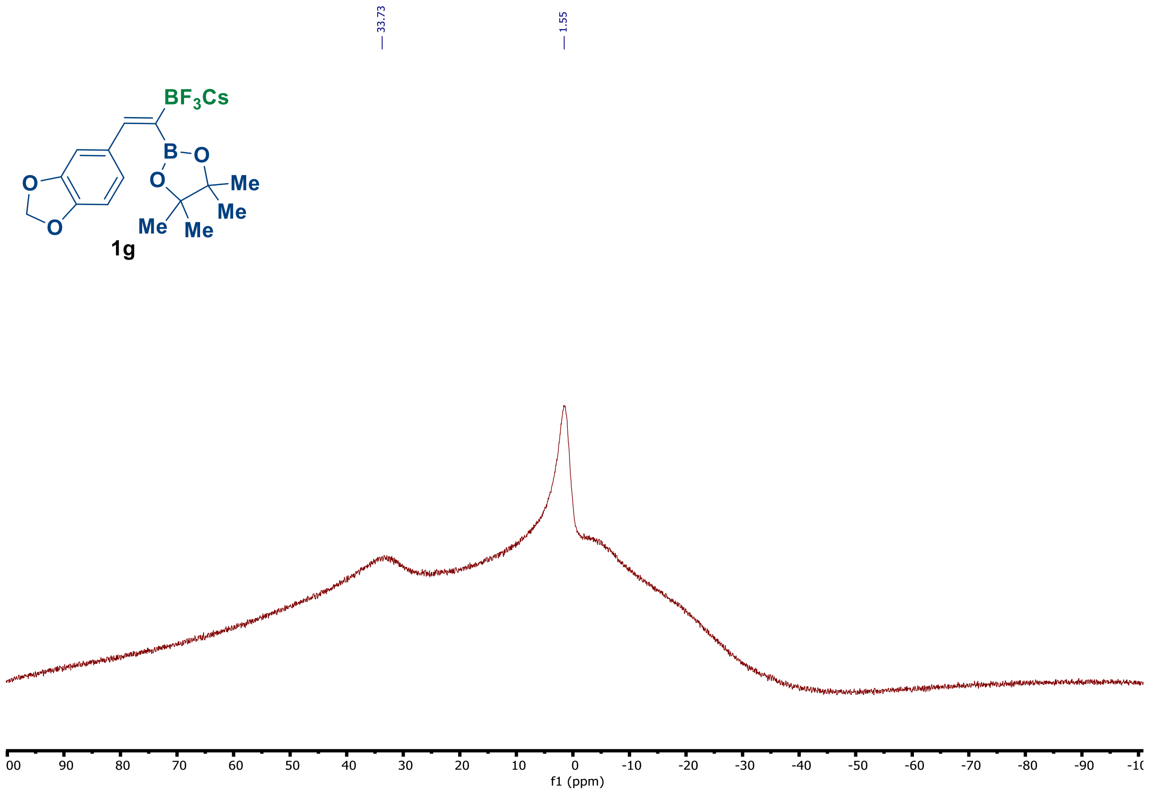

Supplementary Figure 65. ^{11}B NMR (128 MHz, DMSO-d_6) of compound (**1g**).

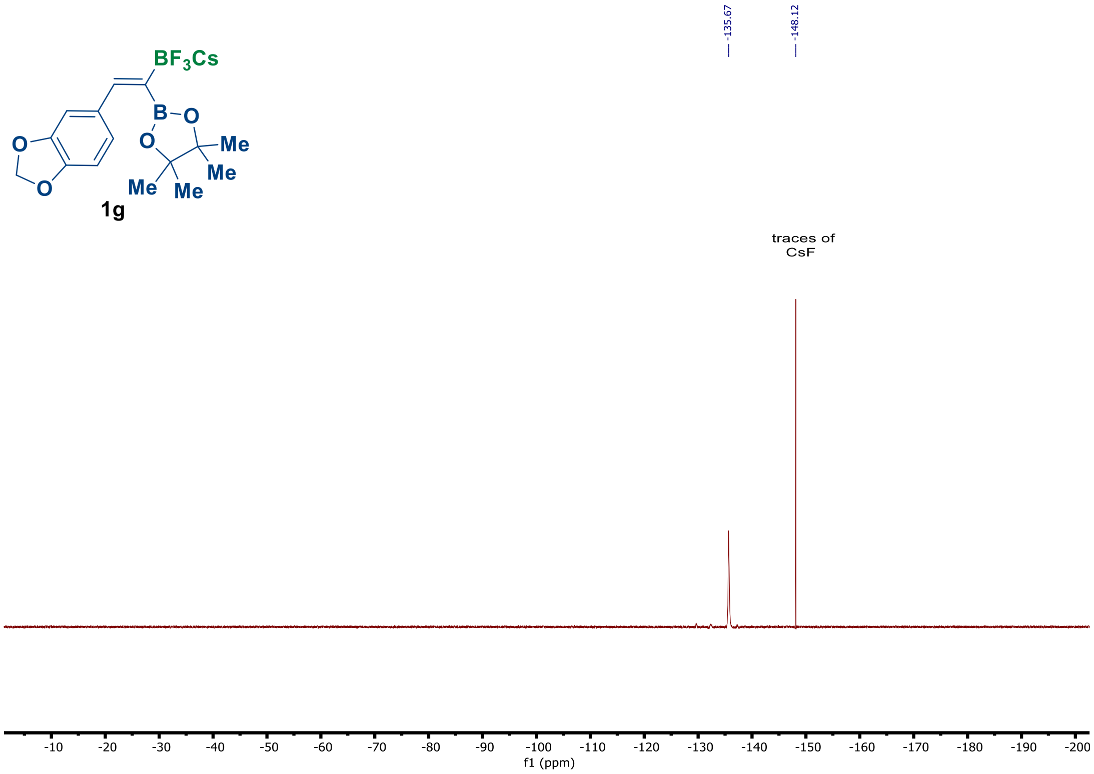

Supplementary Figure 66. ^{19}F NMR (376 MHz, DMSO-d_6) of compound (**1g**).

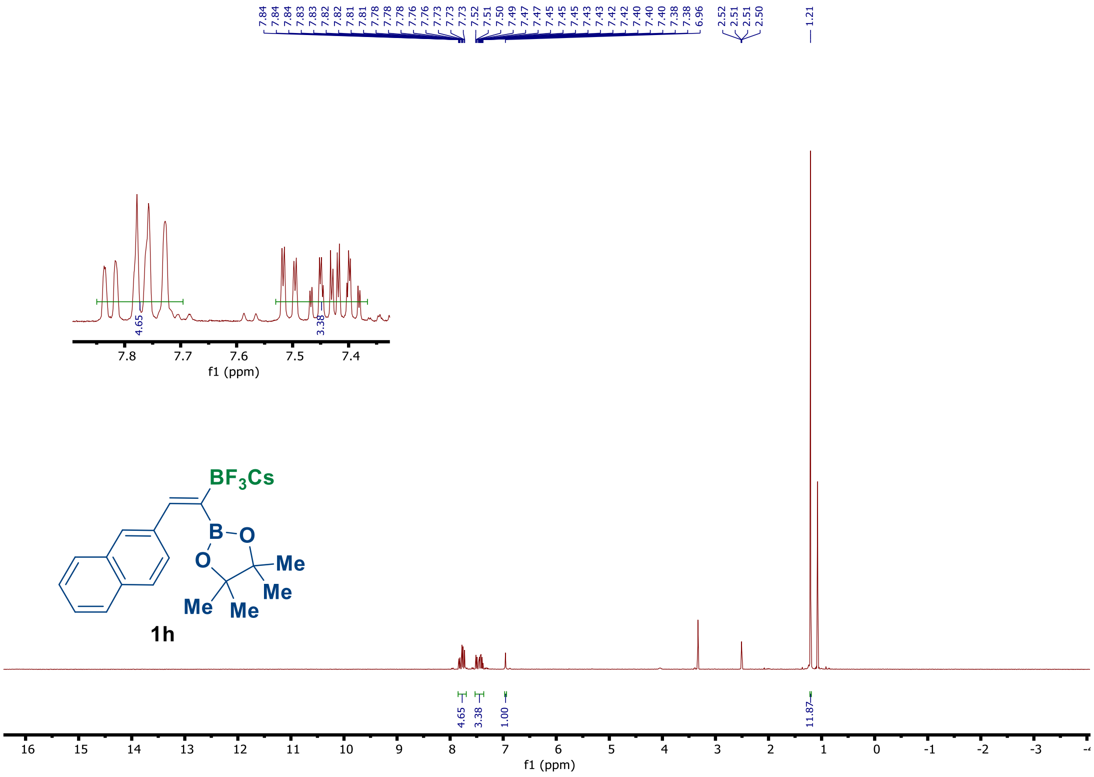

Supplementary Figure 67. ^{1}H NMR (400 MHz, DMSO-d_6) of compound (**1h**).

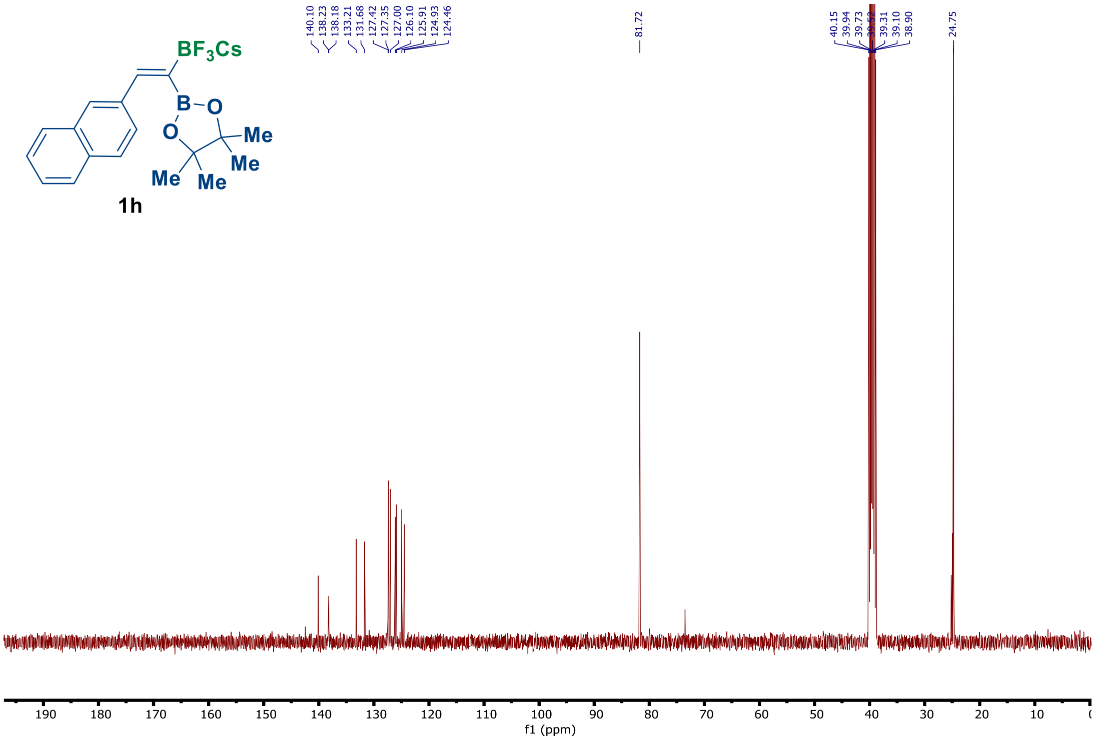

Supplementary Figure 68. ^{13}C NMR (101 MHz, DMSO-d_6) of compound (**1h**).

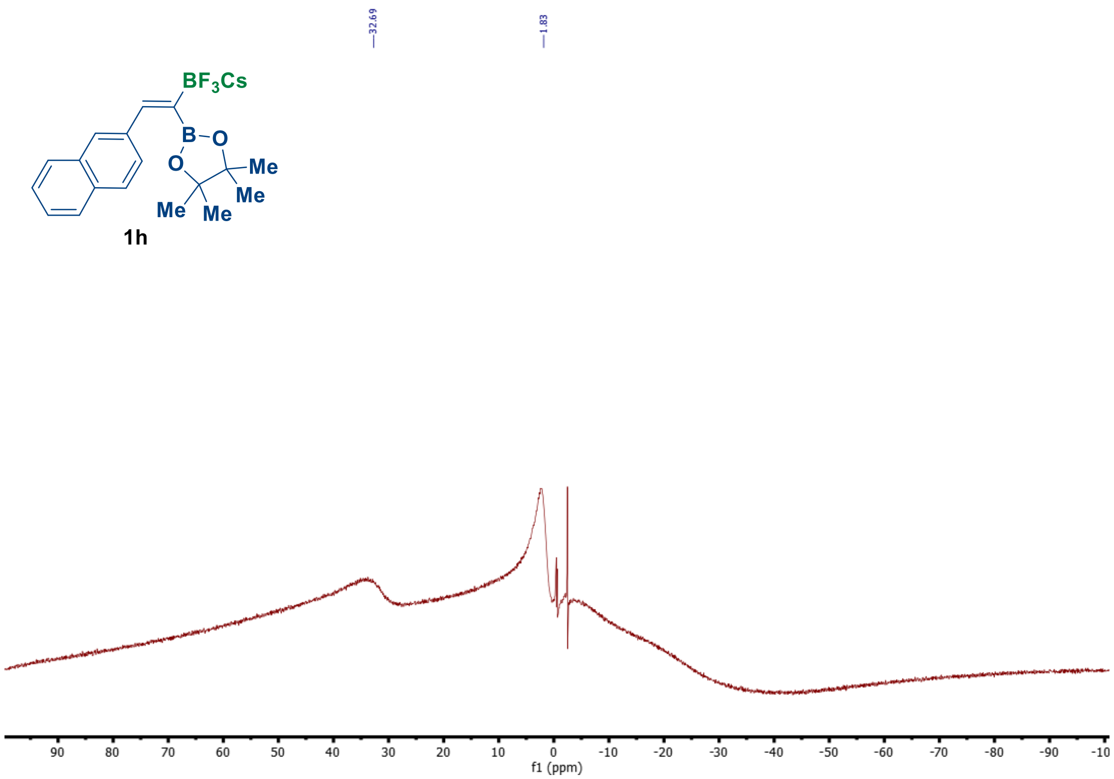

Supplementary Figure 69. ^{11}B NMR (128 MHz, DMSO-d_6) of compound (**1h**).

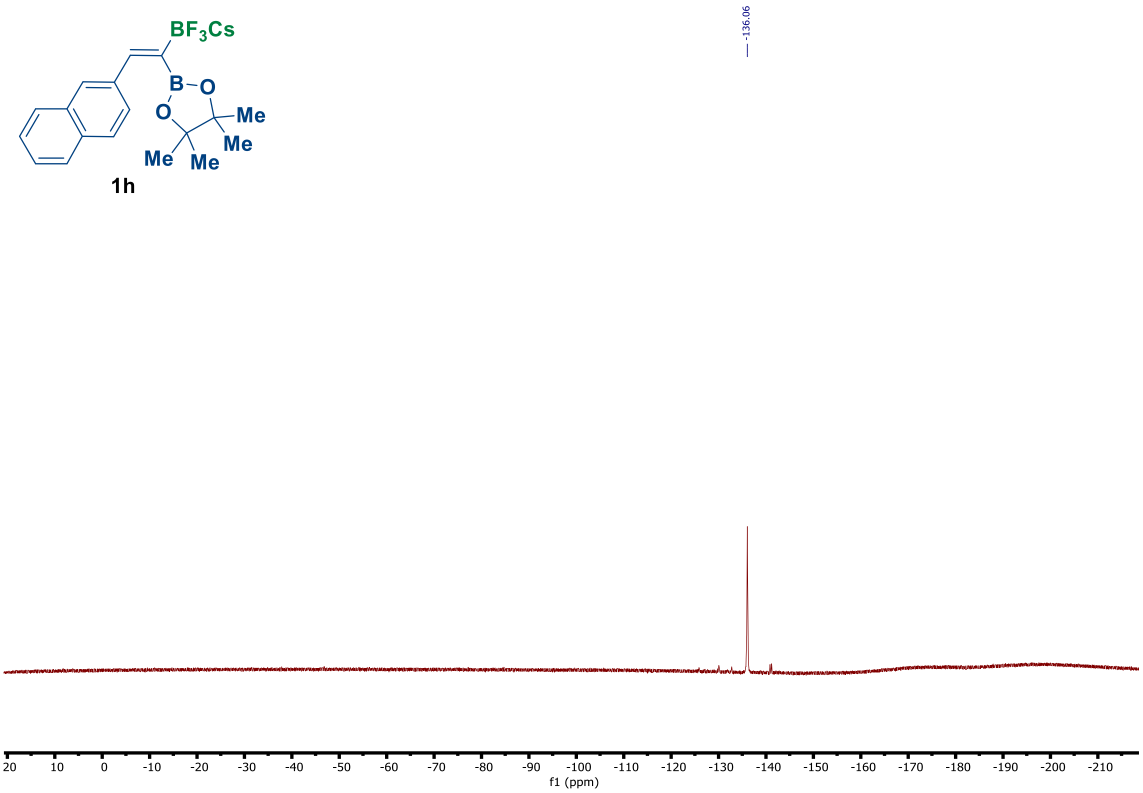

Supplementary Figure 70. ^{19}F NMR (376 MHz, DMSO-d_6) of compound (**1h**).

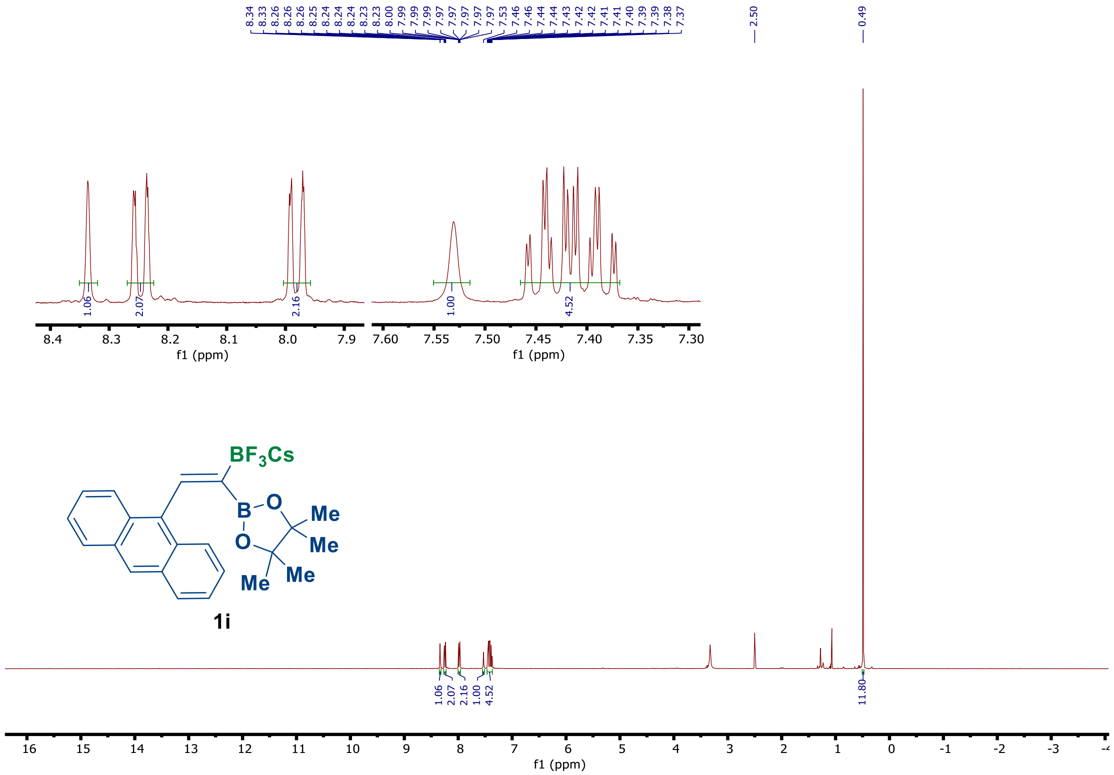

Supplementary Figure 71. ^{1}H NMR (400 MHz, DMSO-d_6) of compound (**1i**).

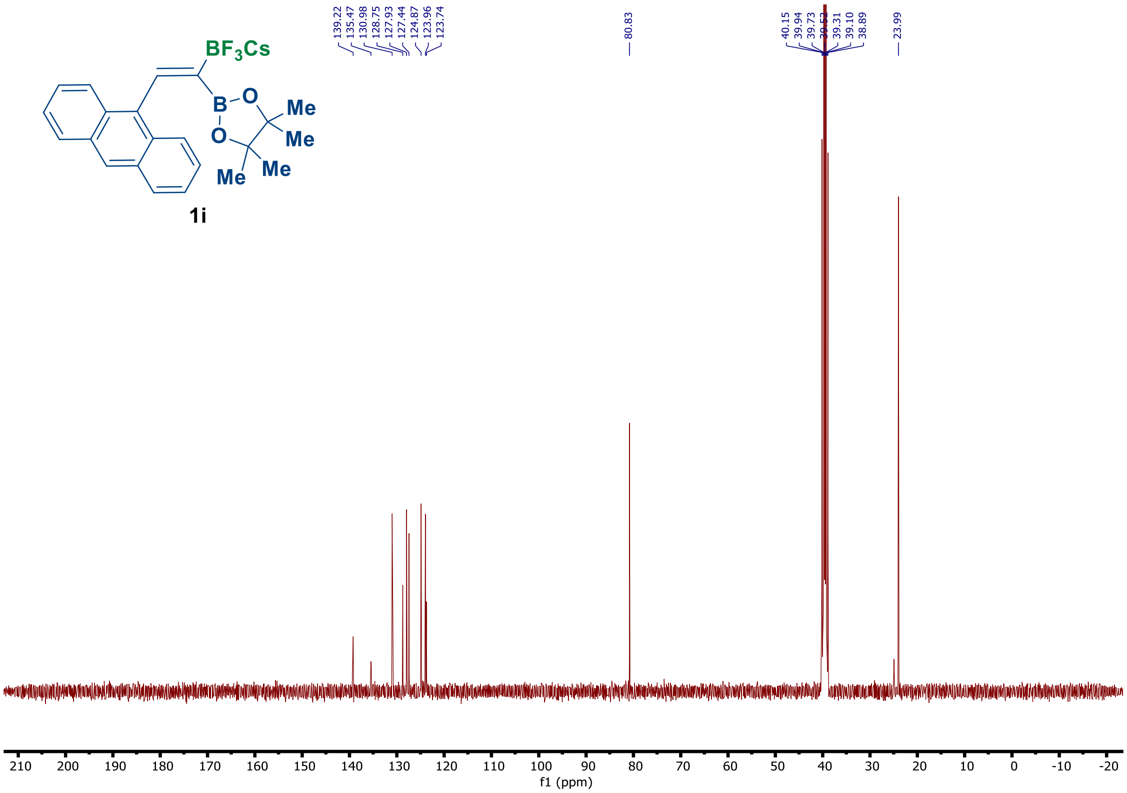

Supplementary Figure 72. ^{13}C NMR (101 MHz, DMSO-d_6) of compound (**1i**).

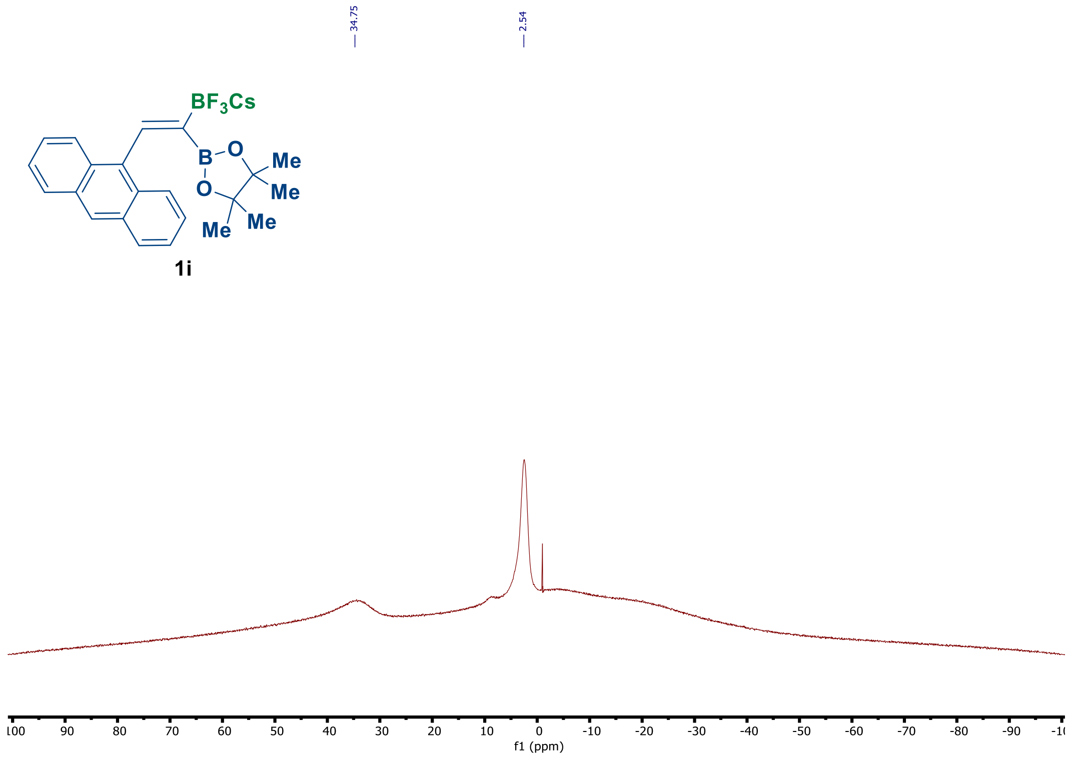

Supplementary Figure 73. ^{11}B NMR (128 MHz, DMSO-d_6) of compound (**1i**).

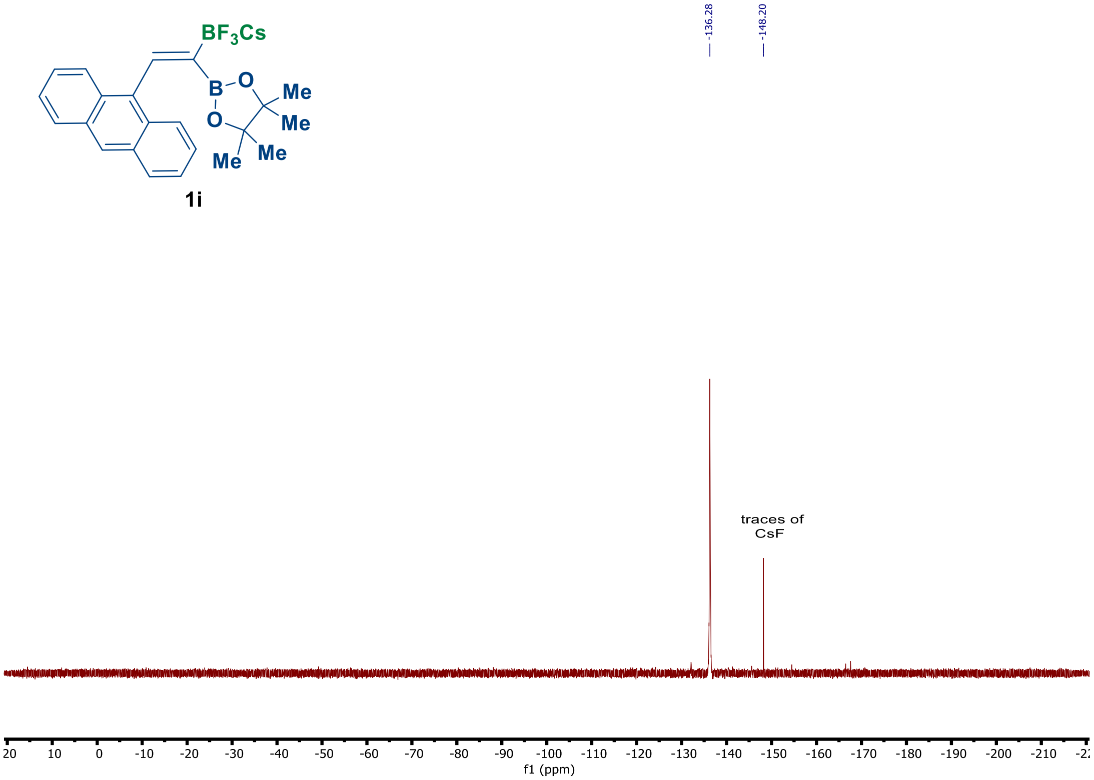

Supplementary Figure 74. ^{19}F NMR (376 MHz, DMSO-d_6) of compound (**1i**).

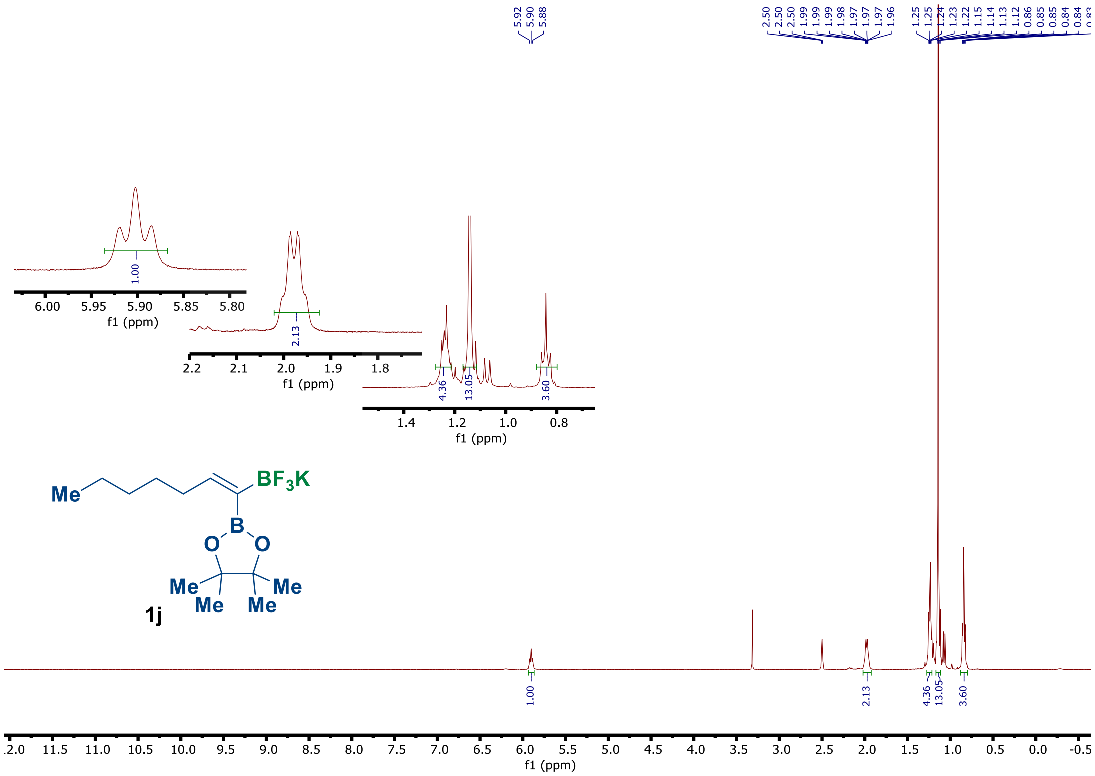

Supplementary Figure 75. ^{1}H NMR (400 MHz, DMSO-d_6) of compound (**1j**).

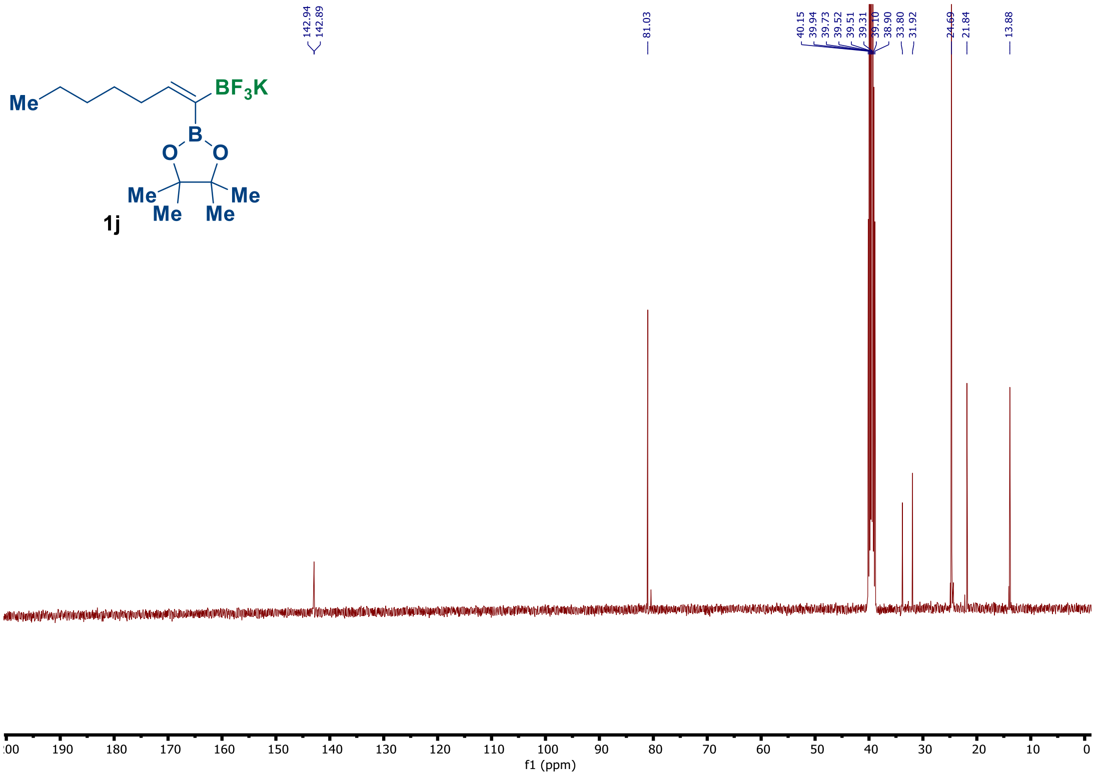

Supplementary Figure 76. ^{13}C NMR (101 MHz, DMSO-d_6) of compound (**1j**).

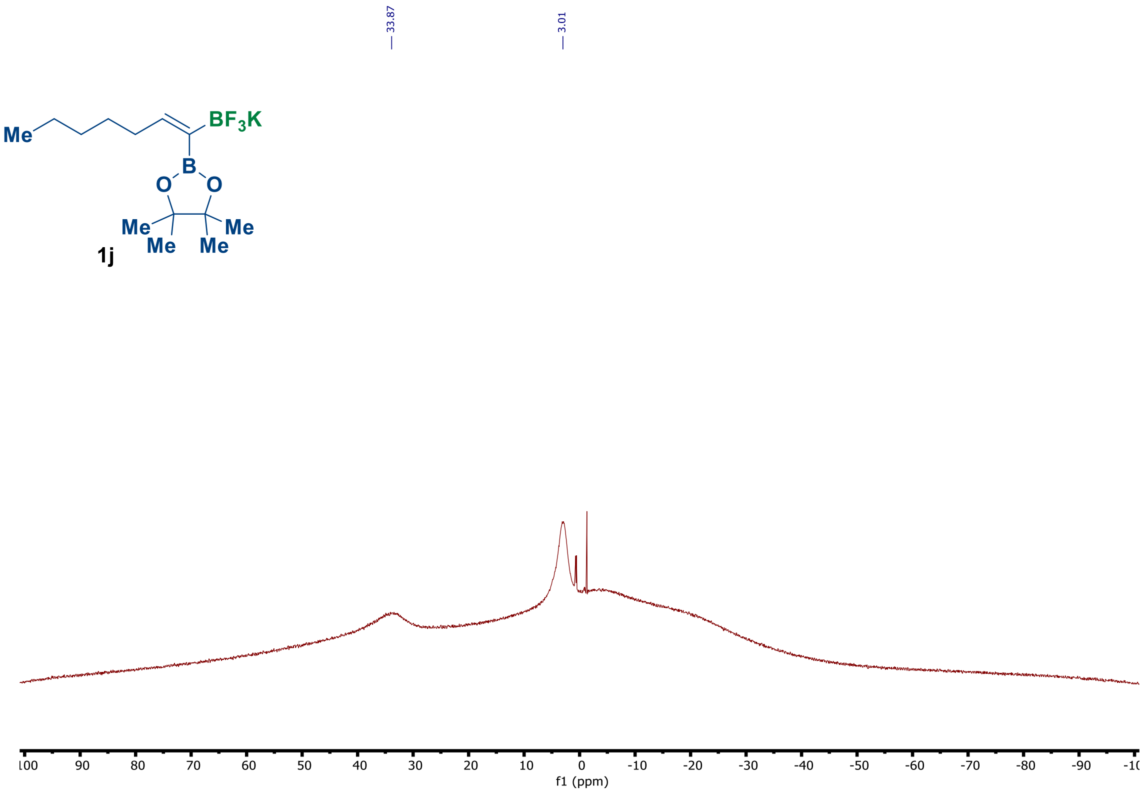

Supplementary Figure 77. ^{11}B NMR (128 MHz, DMSO-d_6) of compound (**1j**).

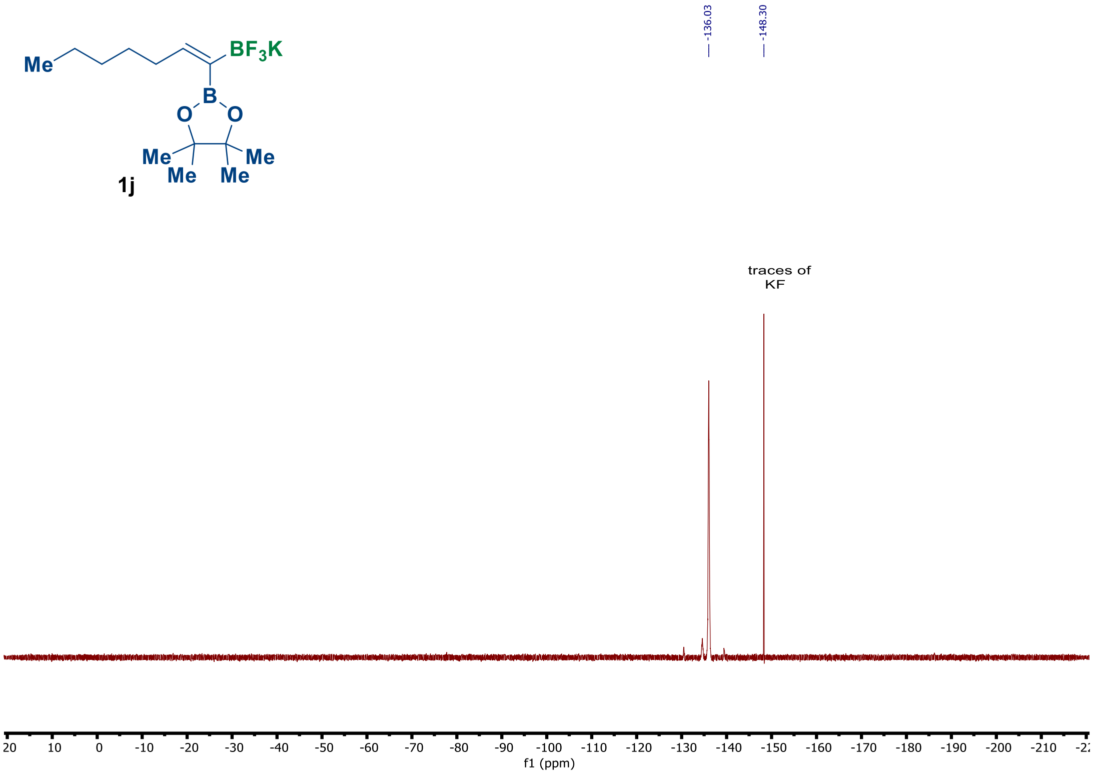

Supplementary Figure 78. ^{19}F NMR (376 MHz, DMSO-d_6) of compound (**1j**).

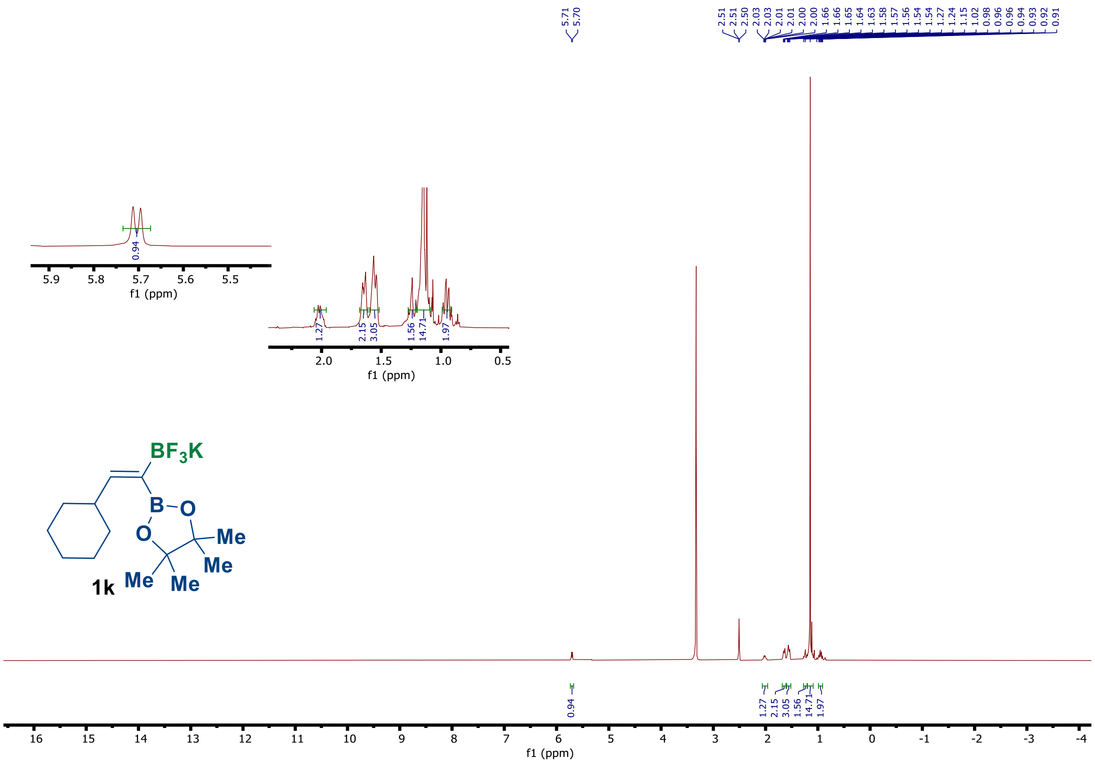

Supplementary Figure 79. ^{1}H NMR (400 MHz, DMSO-d_6) of compound (**1k**).

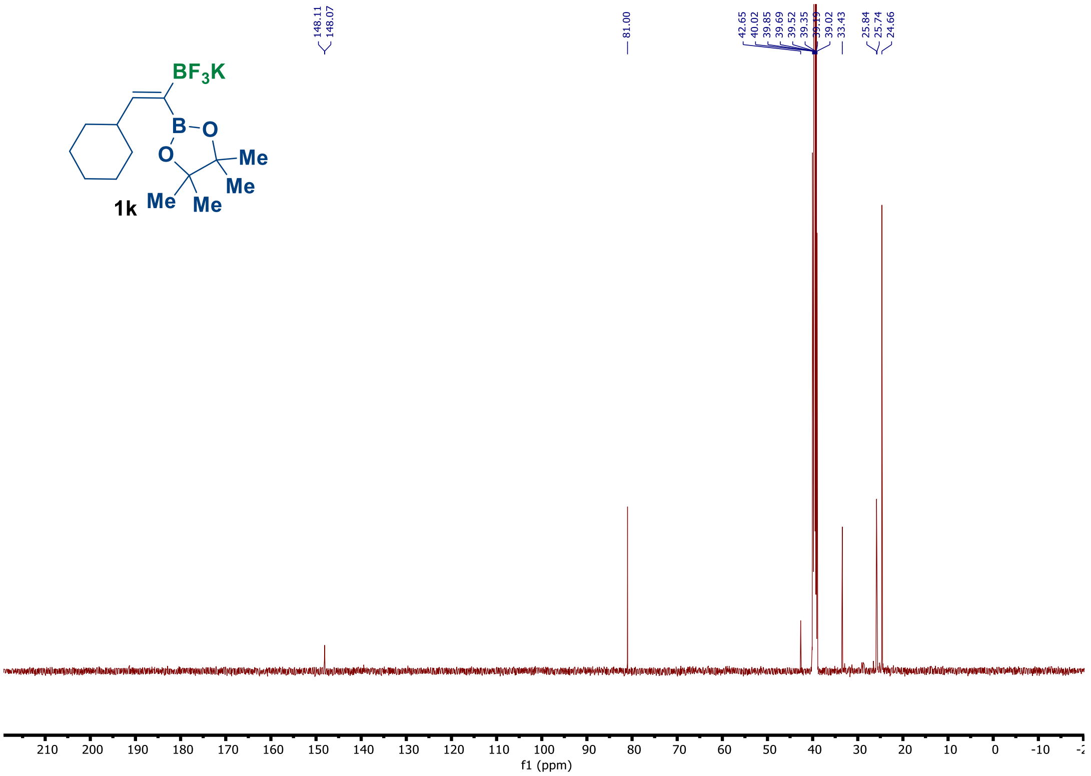

Supplementary Figure 80. ^{13}C NMR (101 MHz, DMSO-d_6) of compound (**1k**).

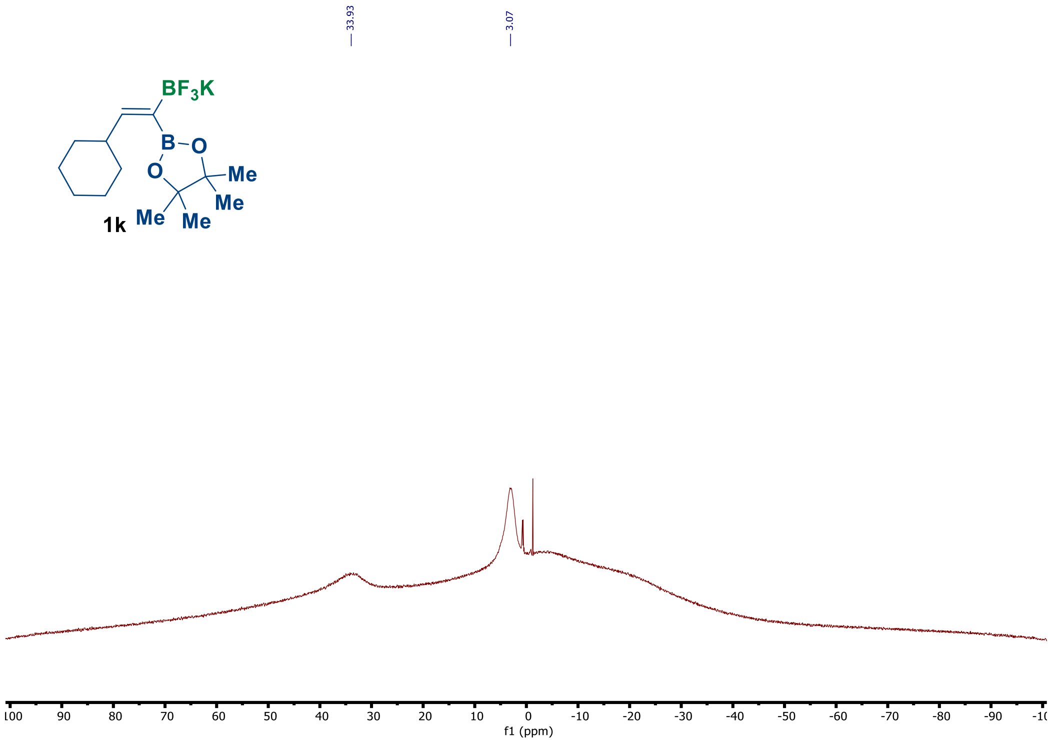

Supplementary Figure 81. ^{11}B NMR (128 MHz, DMSO-d_6) of compound (**1k**).

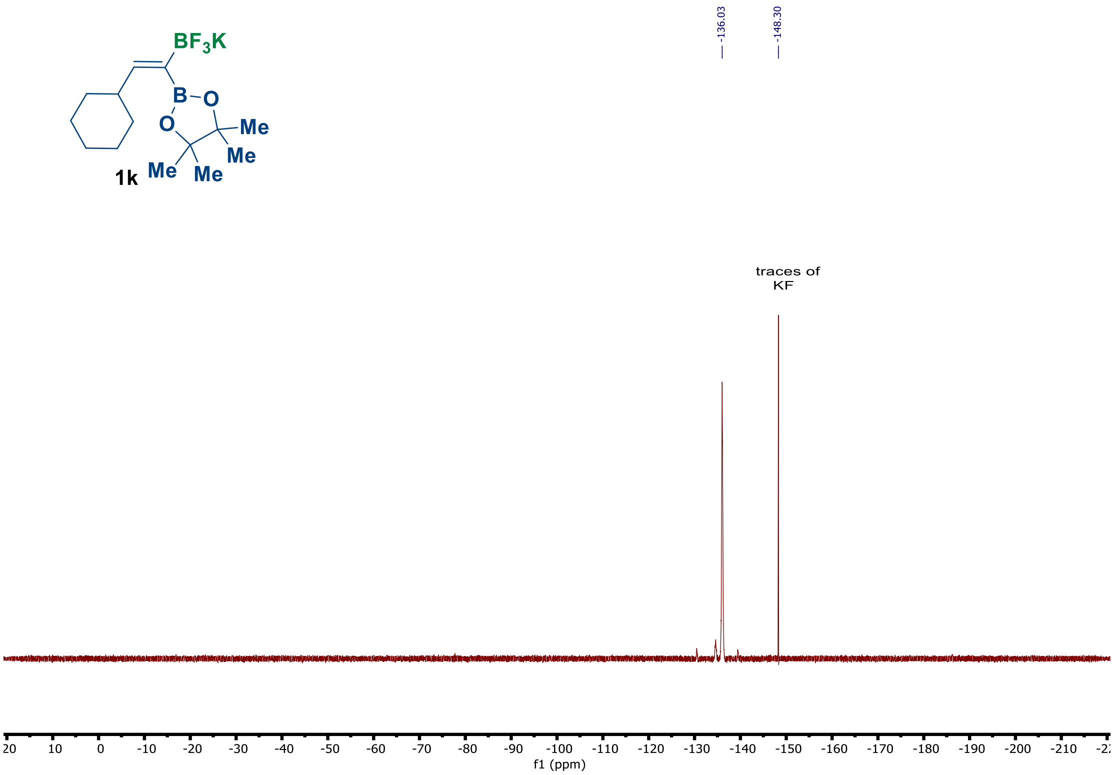

Supplementary Figure 82. ^{19}F NMR (376 MHz, DMSO-d_6) of compound (**1k**).

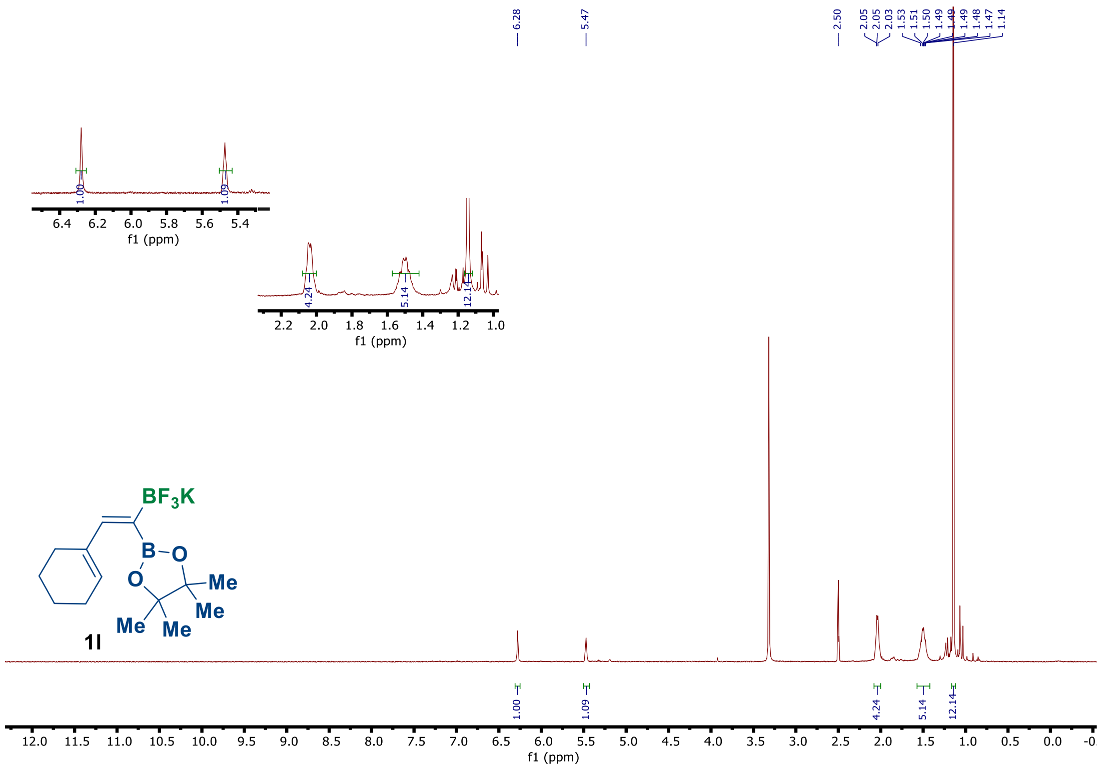

Supplementary Figure 83. ^{1}H NMR (400 MHz, DMSO-d_6) of compound (**1l**).

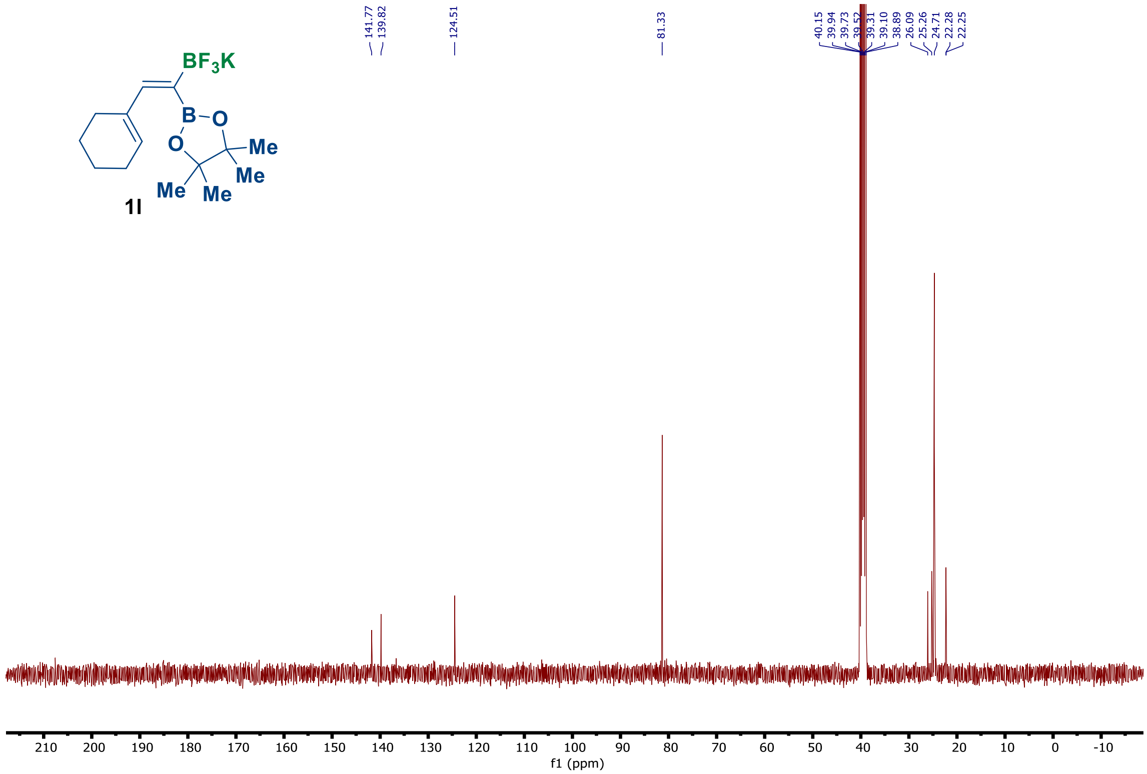

Supplementary Figure 84. ^{13}C NMR (101 MHz, DMSO-d_6) of compound (**1l**).

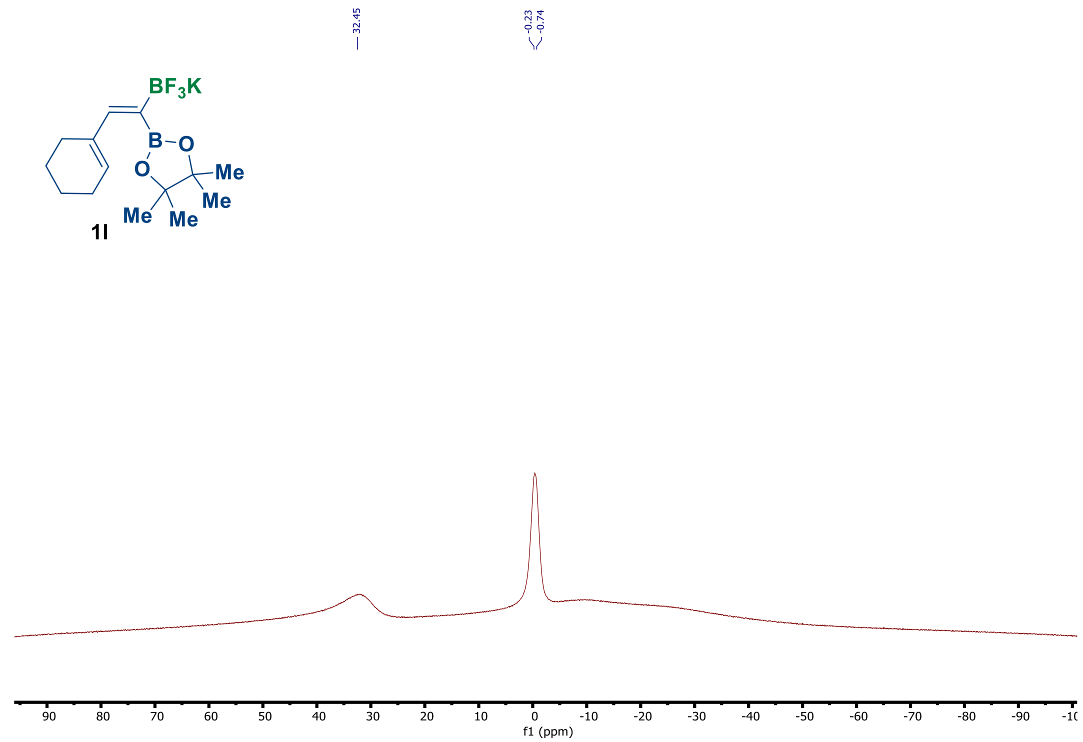

Supplementary Figure 85. ^{11}B NMR (128 MHz, DMSO-d_6) of compound (**1l**).

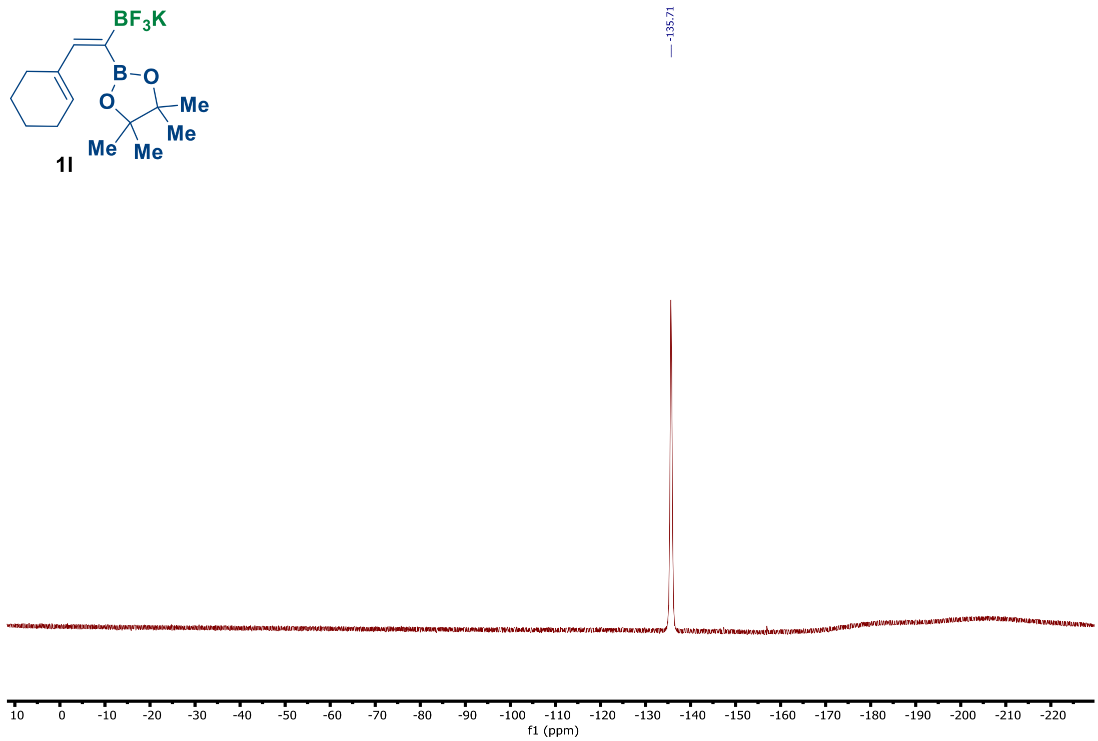

Supplementary Figure 86. ^{19}F NMR (376 MHz, DMSO-d_6) of compound (**1l**).

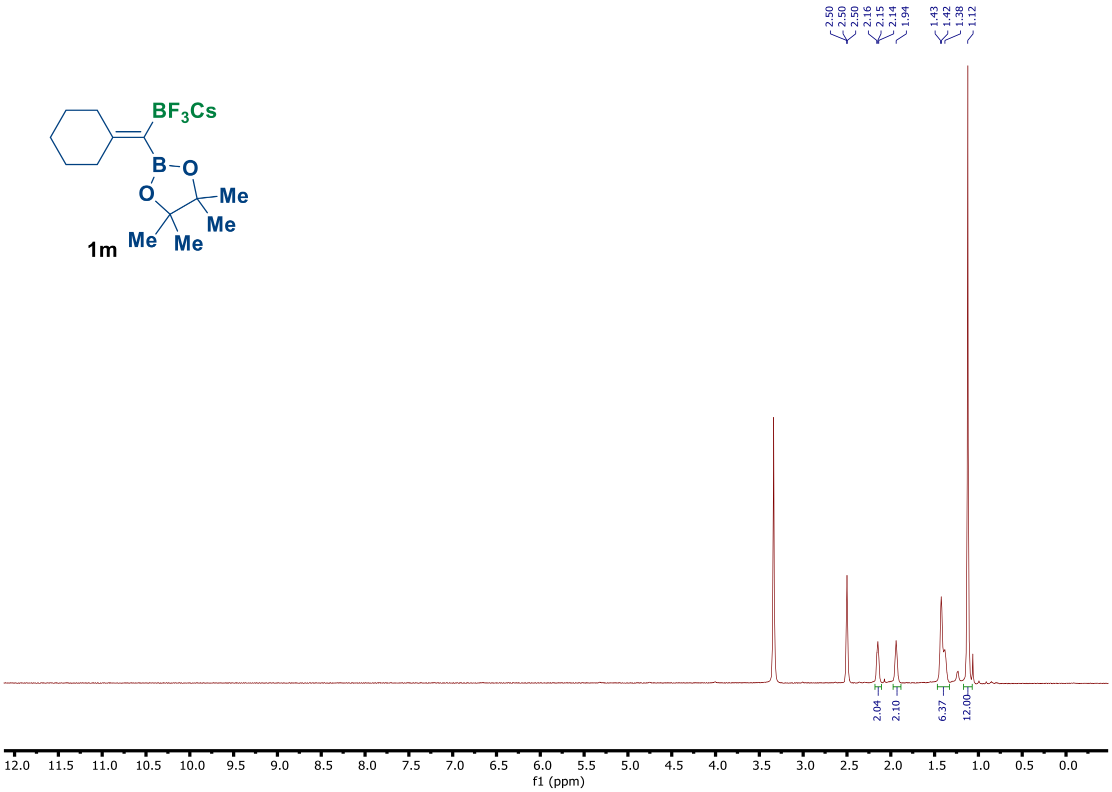

Supplementary Figure 87. ^{1}H NMR (400 MHz, DMSO-d_6) of compound (**1m**).

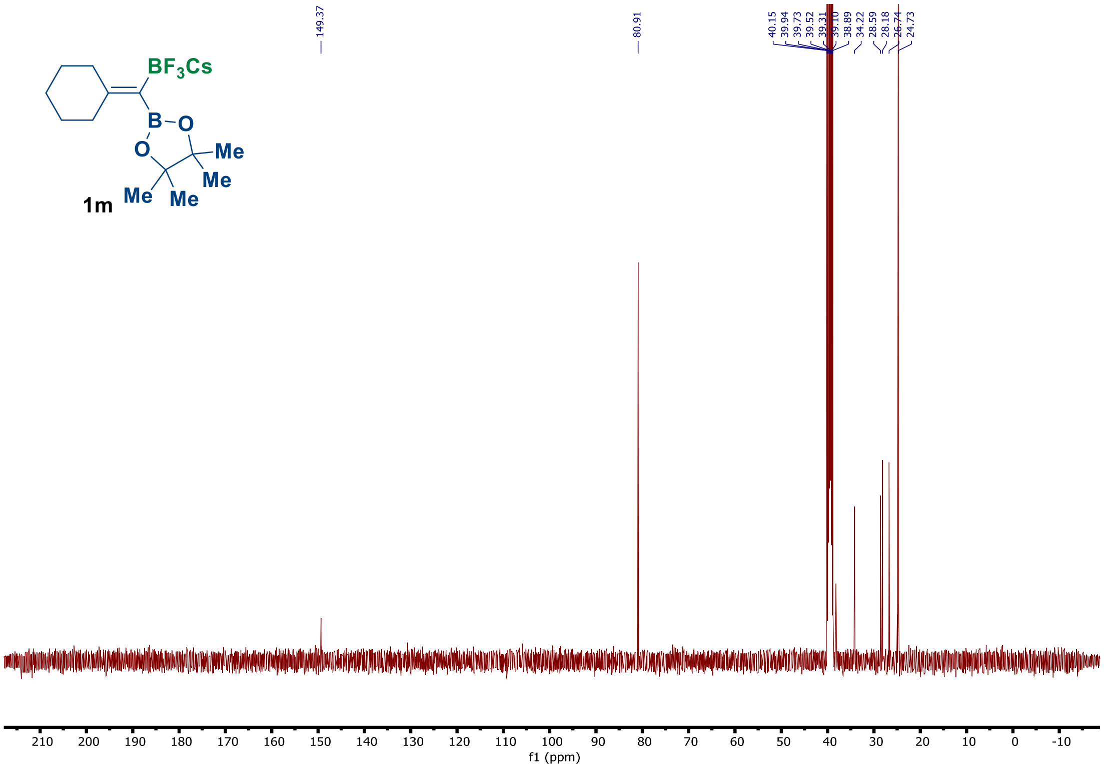

Supplementary Figure 88. ^{13}C NMR (101 MHz, DMSO-d_6) of compound (**1m**).

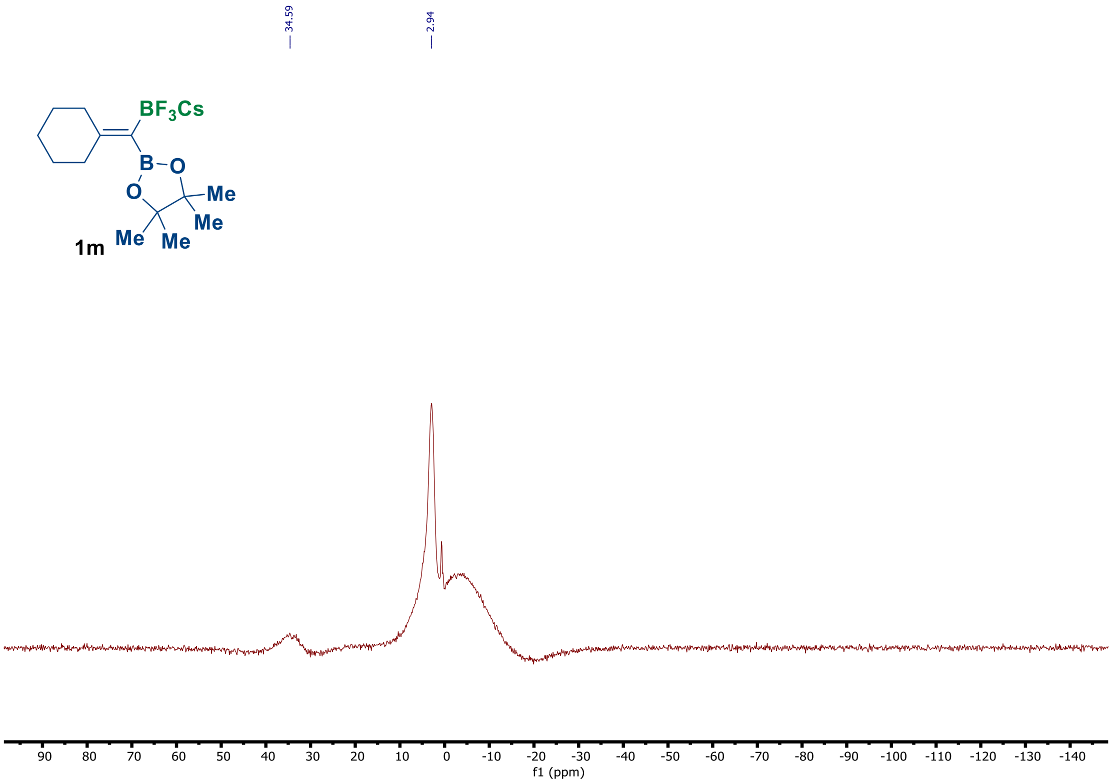

Supplementary Figure 89. ^{11}B NMR (128 MHz, DMSO-d_6) of compound (**1m**).

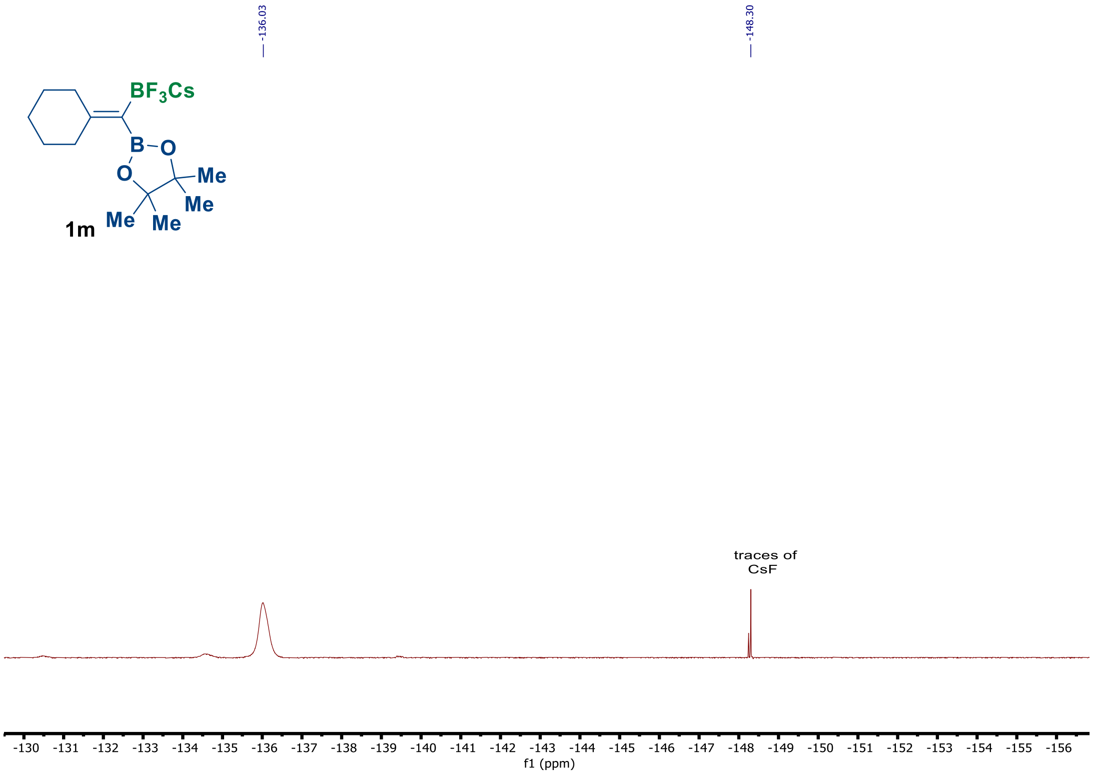

Supplementary Figure 90. ^{19}F NMR (376 MHz, DMSO-d_6) of compound (**1m**).

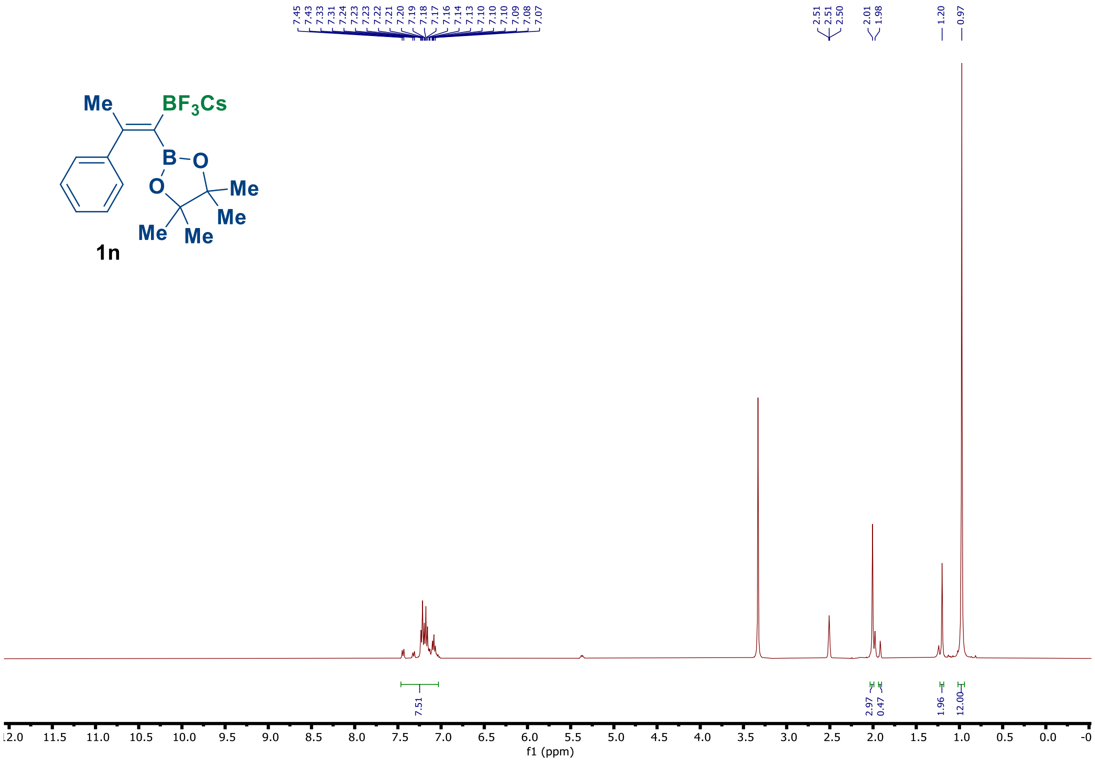

Supplementary Figure 91. ^{1}H NMR (400 MHz, DMSO-d_6) of compound (**1n**)..

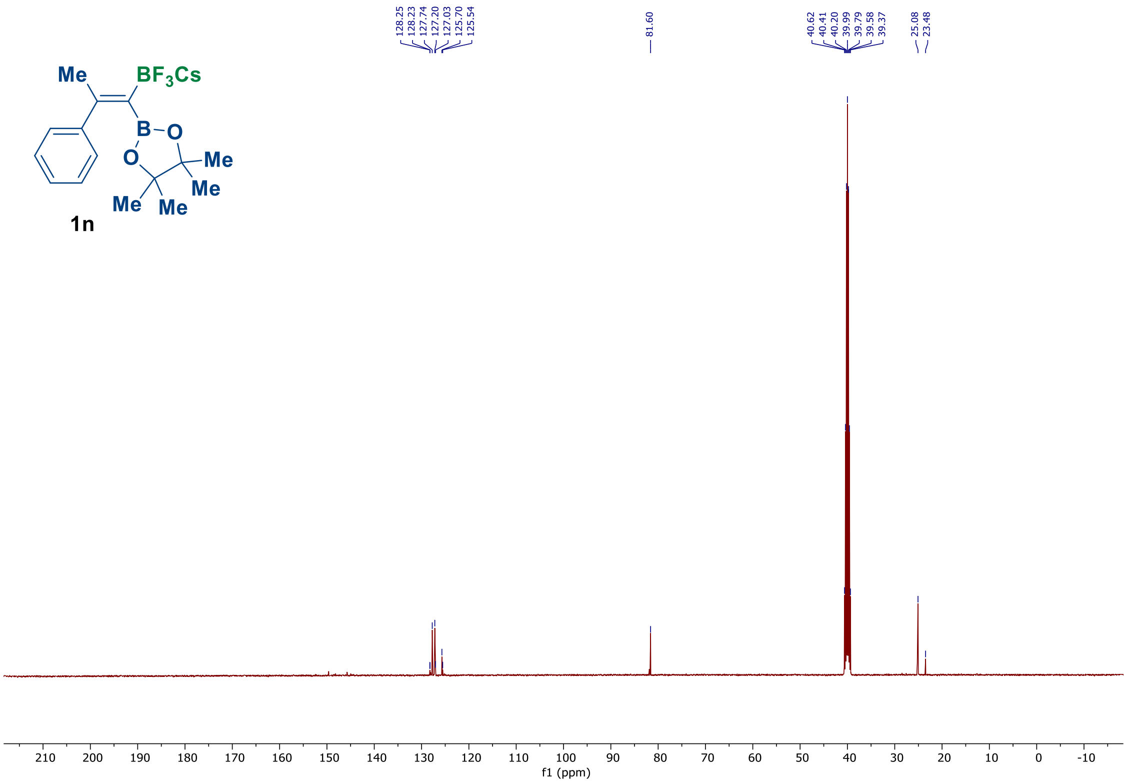

Supplementary Figure 92. ^{13}C NMR (101 MHz, DMSO-d_6) of compound (**1n**).

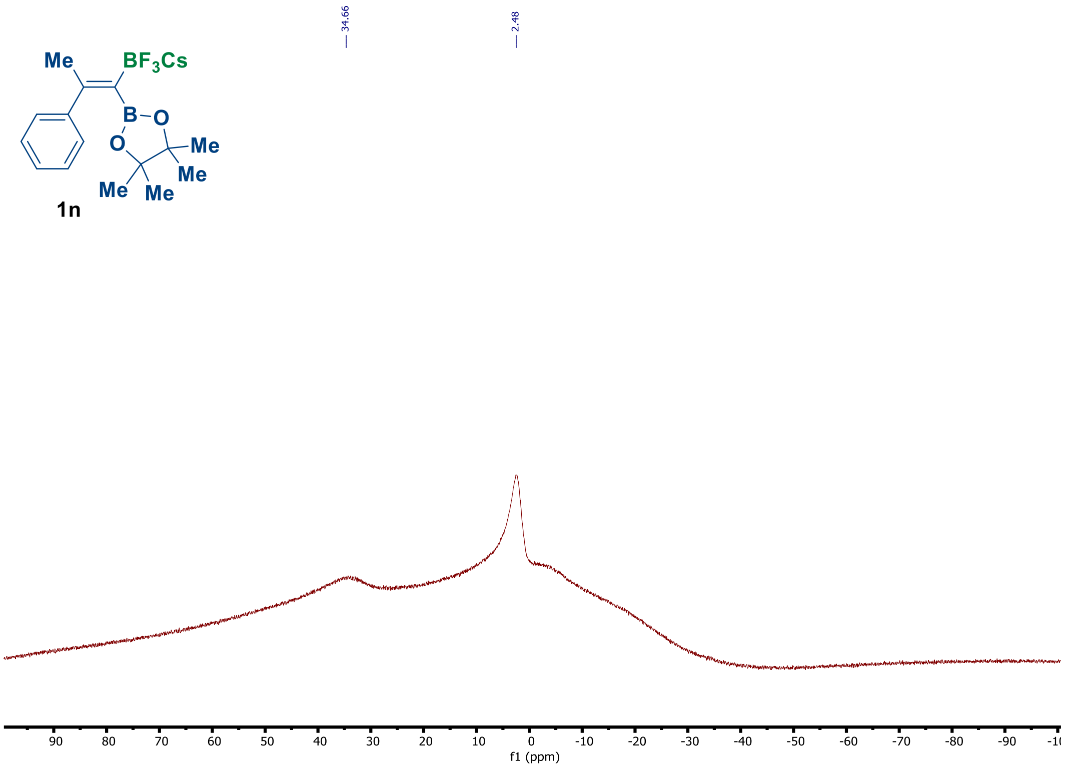

Supplementary Figure 93. ^{11}B NMR (128 MHz, DMSO-d_6) of compound (**1n**).

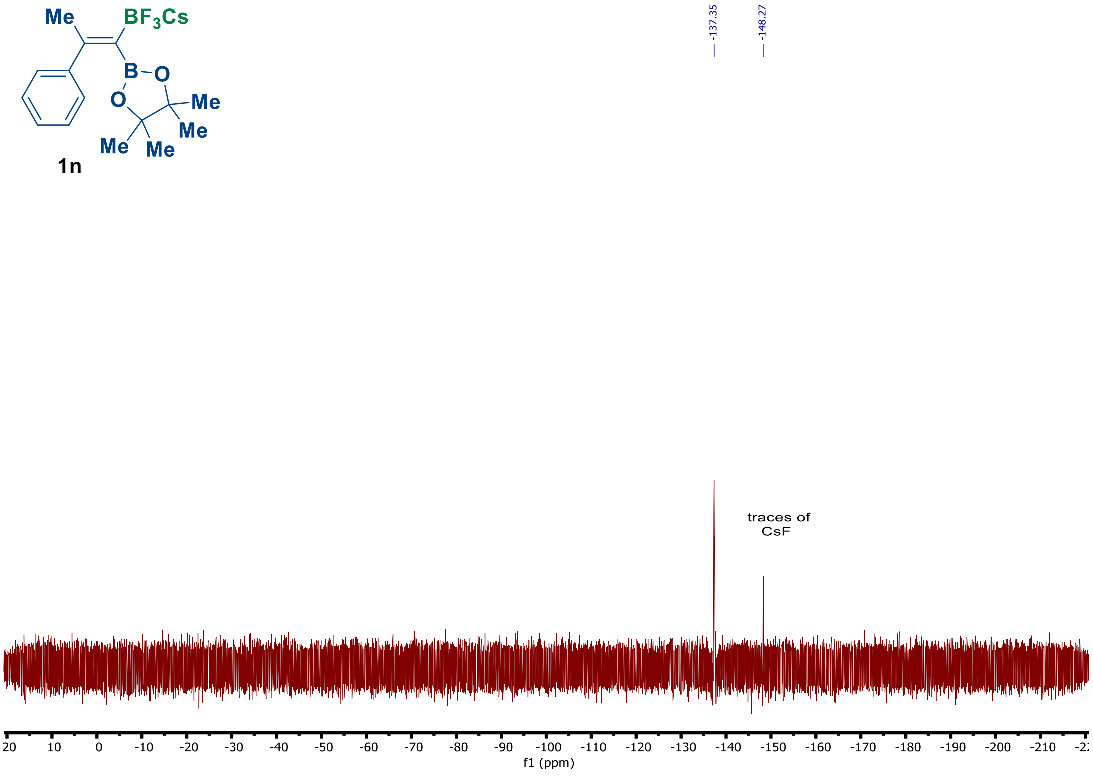

Supplementary Figure 94. ^{19}F NMR (376 MHz, DMSO-d_6) of compound (**1n**).

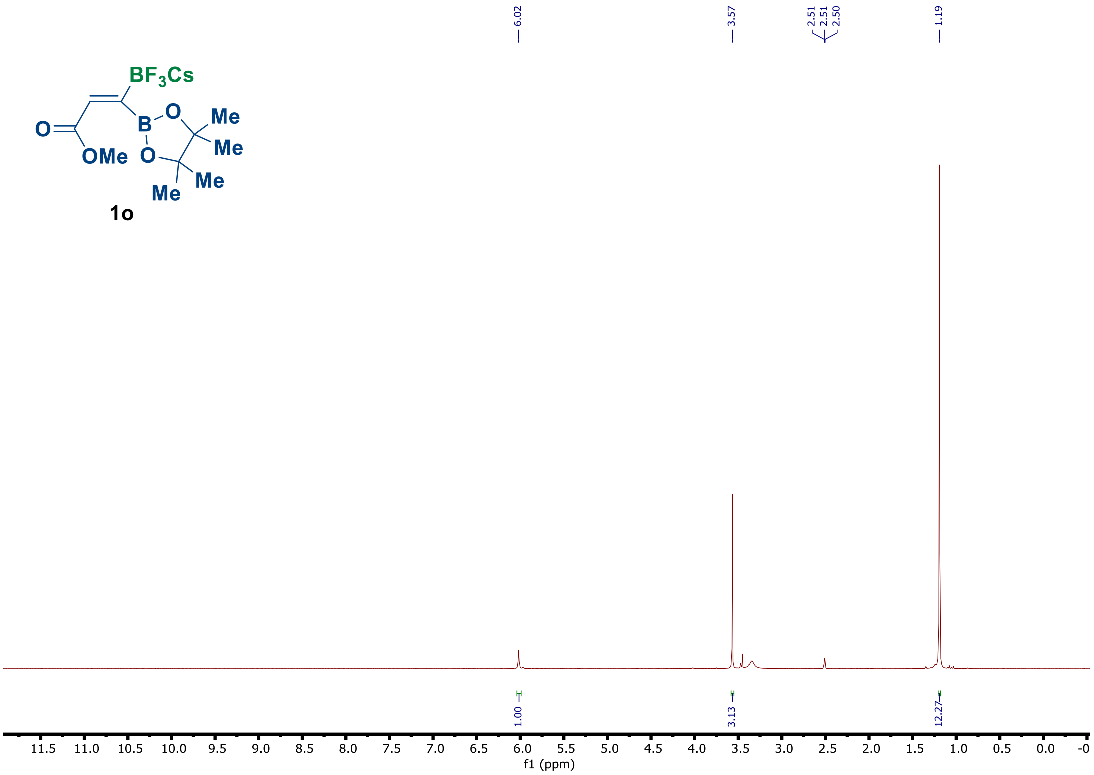

Supplementary Figure 95. ^{1}H NMR (400 MHz, DMSO-d_6) of compound (**1o**).

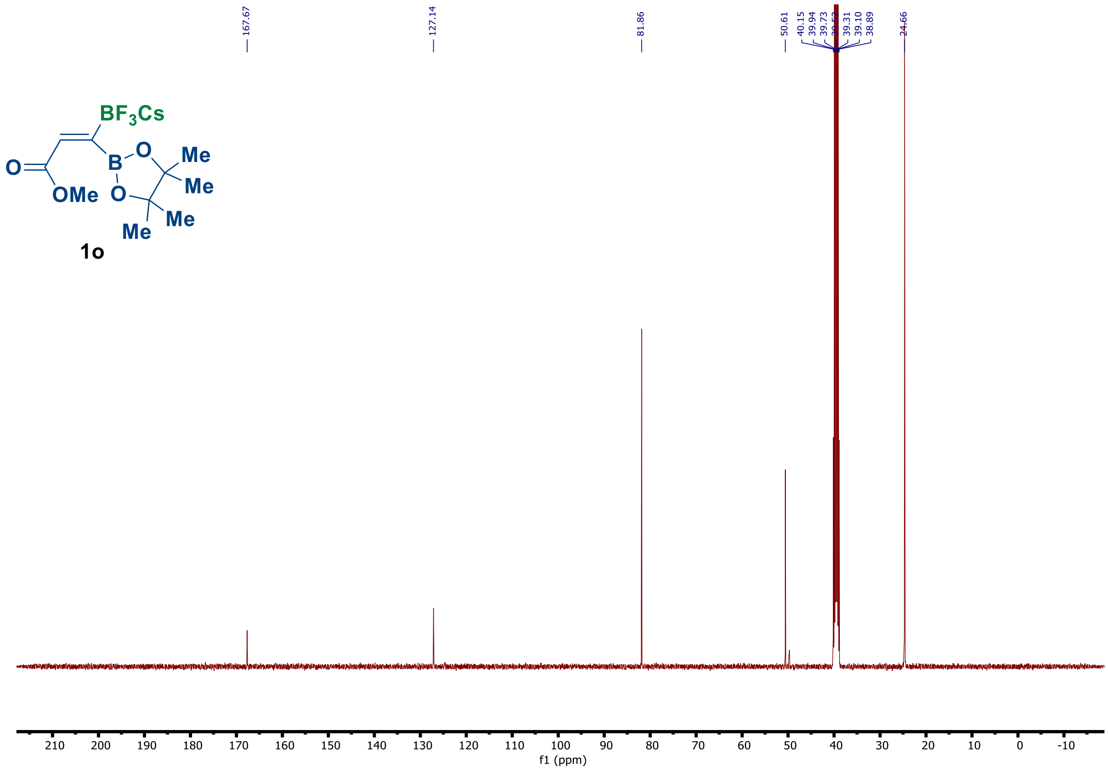

Supplementary Figure 96. ^{13}C NMR (101 MHz, DMSO-d_6) of compound (**1o**).

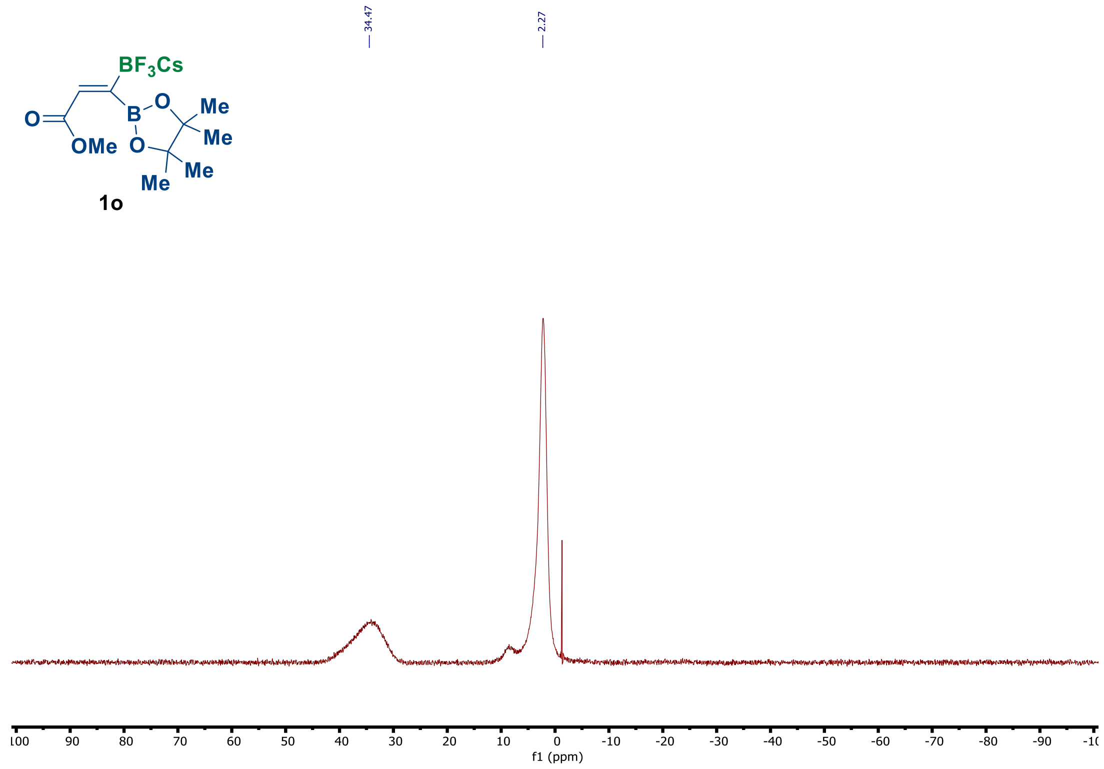

Supplementary Figure 97. ^{11}B NMR (128 MHz, DMSO-d_6) of compound (**1o**).

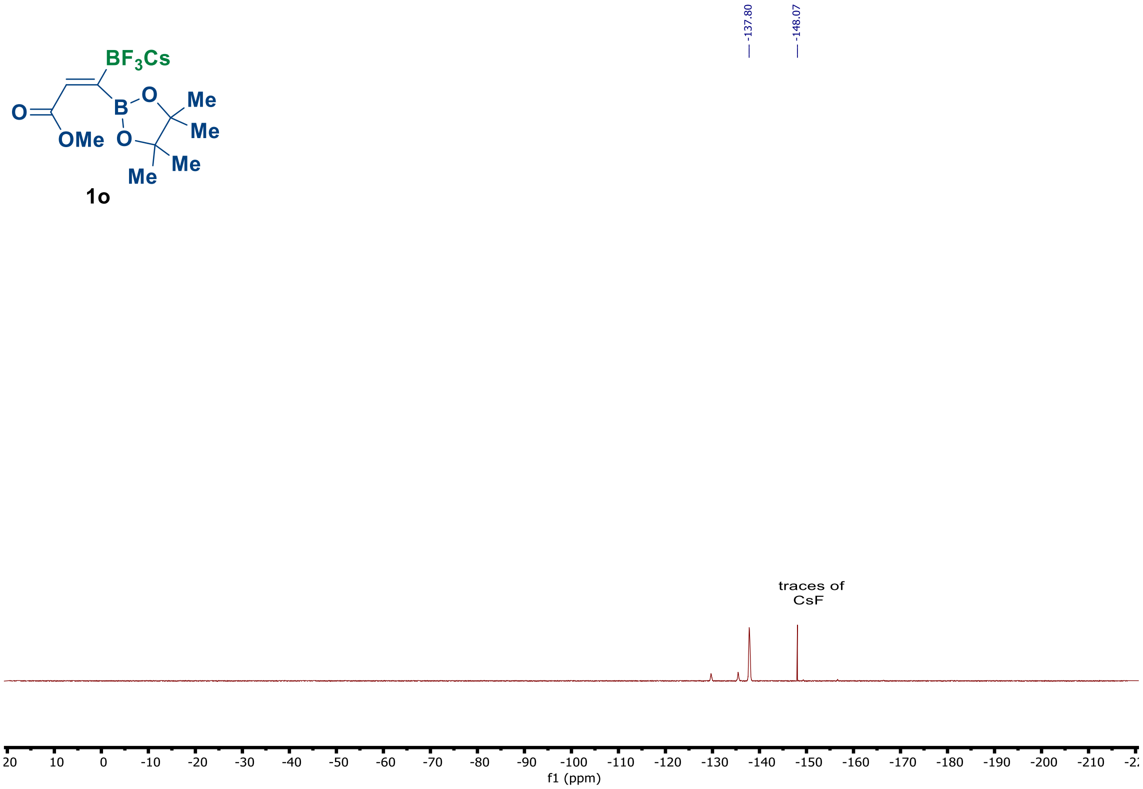

Supplementary Figure 98. ^{19}F NMR (376 MHz, DMSO-d_6) of compound (**1o**).

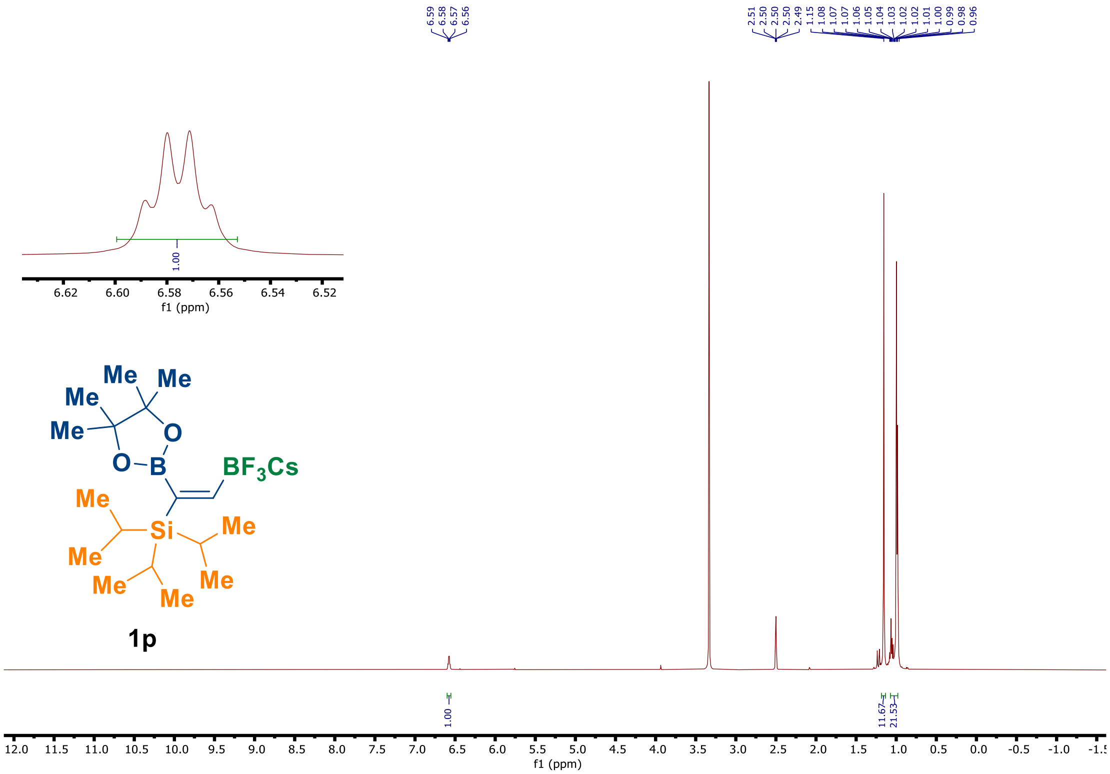

Supplementary Figure 99. ^{1}H NMR (400 MHz, DMSO-d_6) of compound (**1p**).

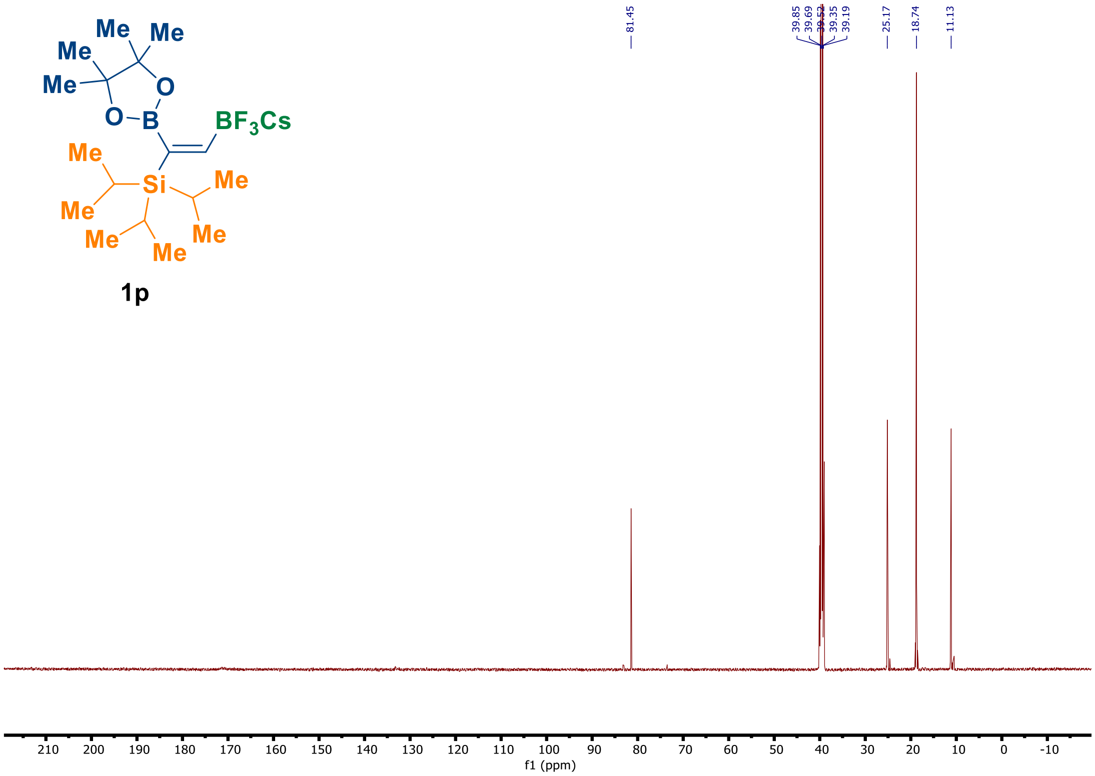

Supplementary Figure 100. ^{13}C NMR (101 MHz, DMSO-d_6) of compound (**1p**).

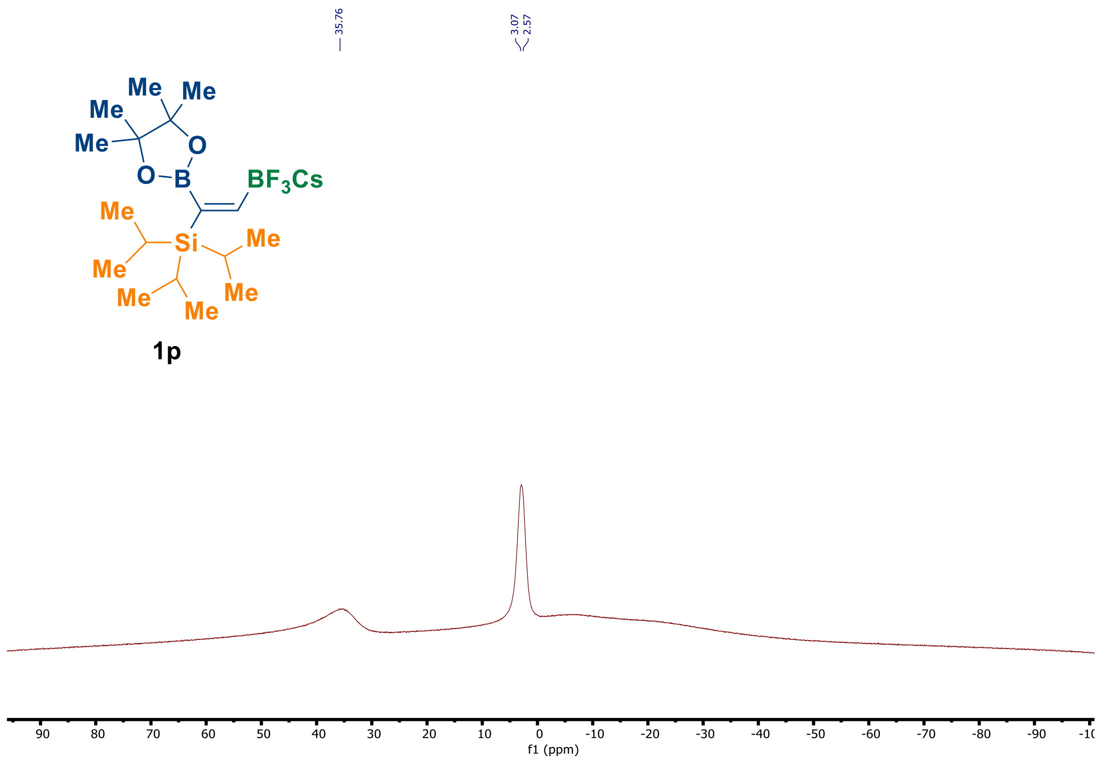

Supplementary Figure 101. ^{11}B NMR (128 MHz, DMSO-d_6) of compound (**1p**).

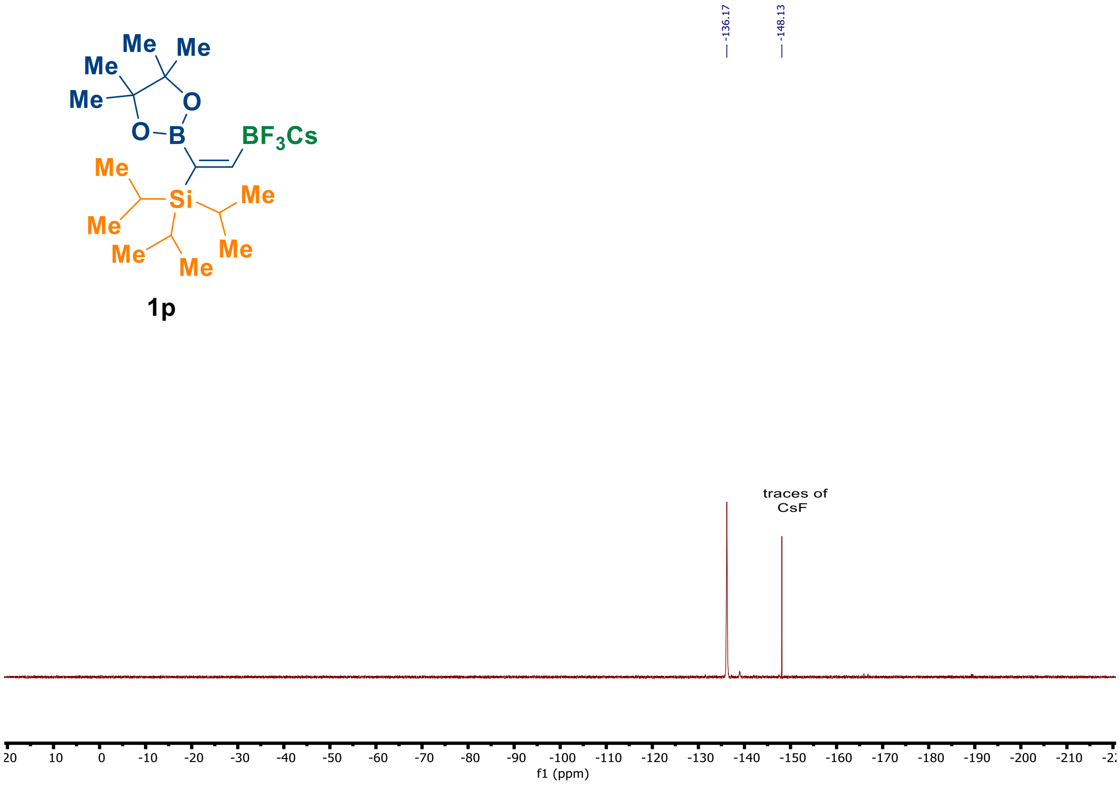

Supplementary Figure 102. ^{19}F NMR (376 MHz, DMSO-d_6) of compound (**1p**).

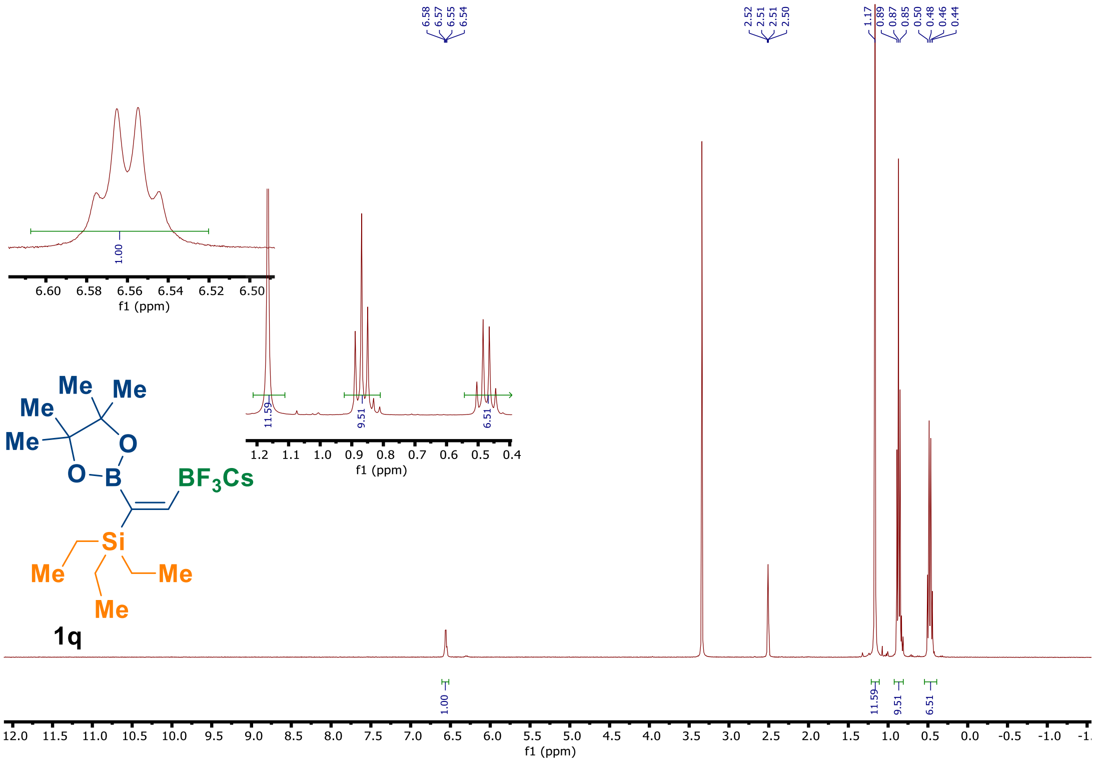

Supplementary Figure 103. ^{1}H NMR (400 MHz, DMSO-d_6) of compound (**1q**).

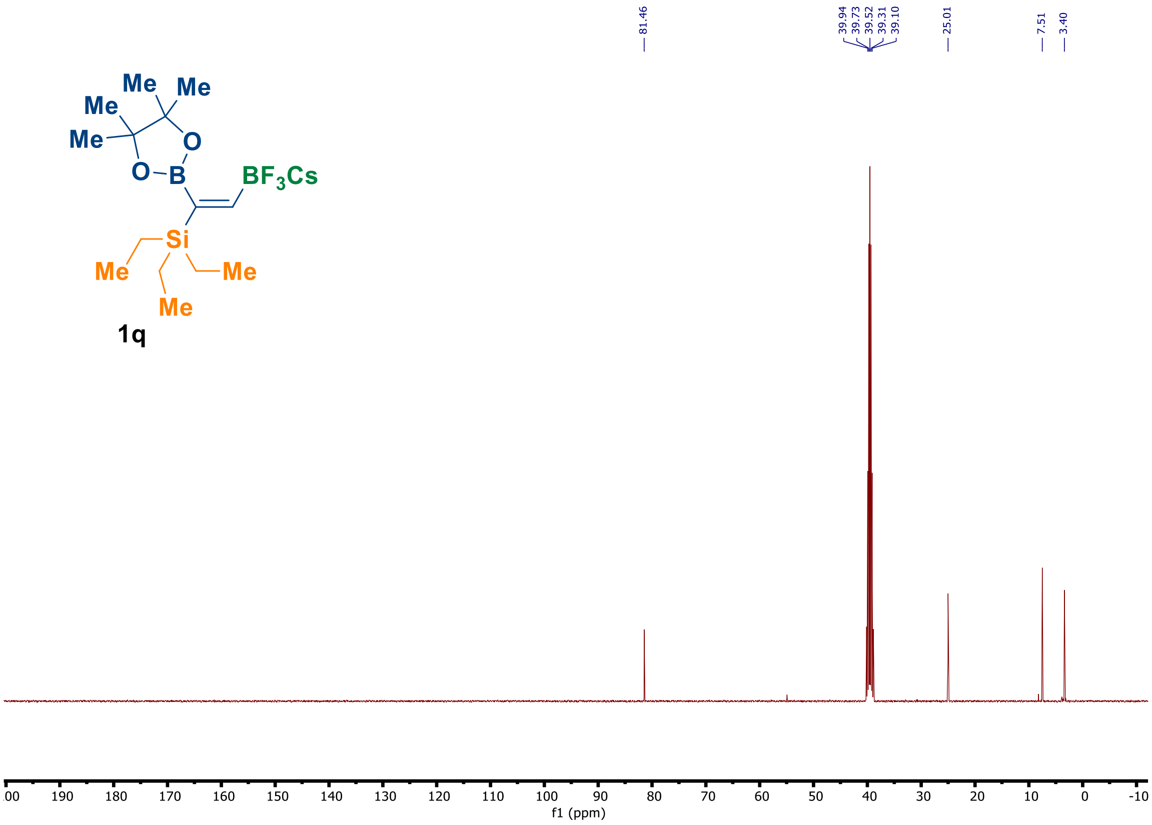

Supplementary Figure 104. ^{13}C NMR (101 MHz, DMSO-d_6) of compound (**1q**).

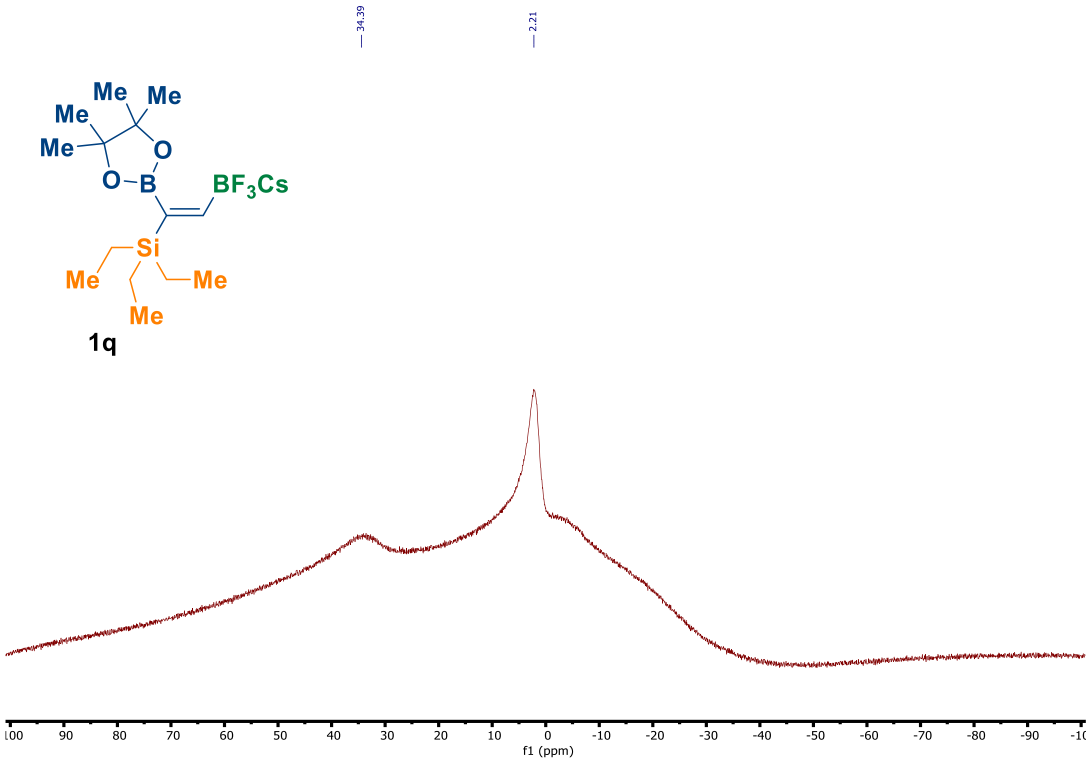

Supplementary Figure 105. ^{11}B NMR (128 MHz, DMSO-d_6) of compound (**1q**).

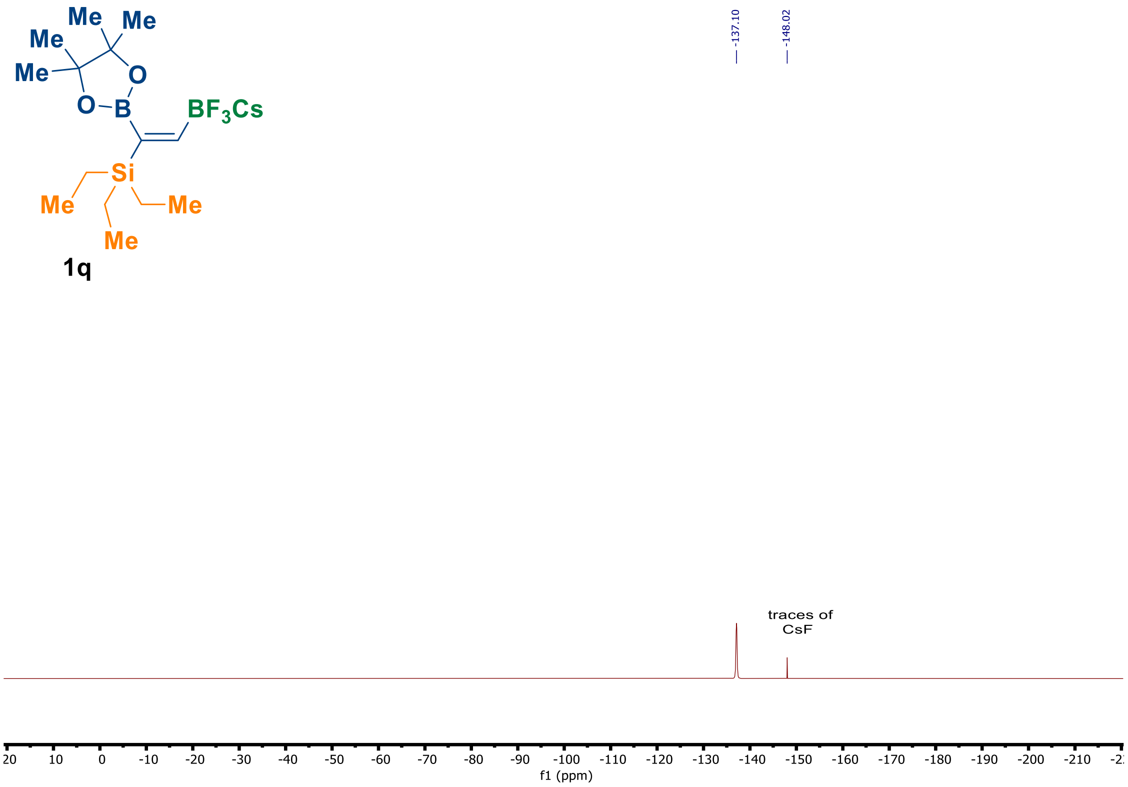

Supplementary Figure 106. ^{19}F NMR (376 MHz, DMSO-d_6) of compound (**1q**).

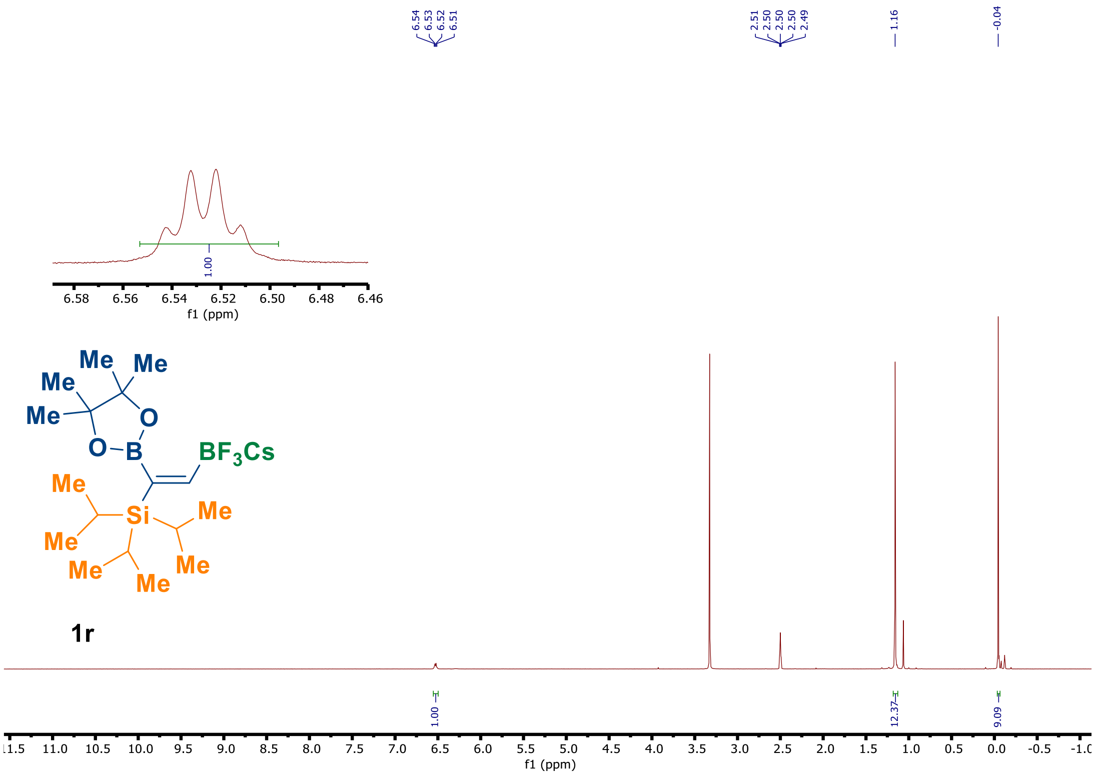

Supplementary Figure 107. ^{1}H NMR (400 MHz, DMSO-d_6) of compound (**1r**).

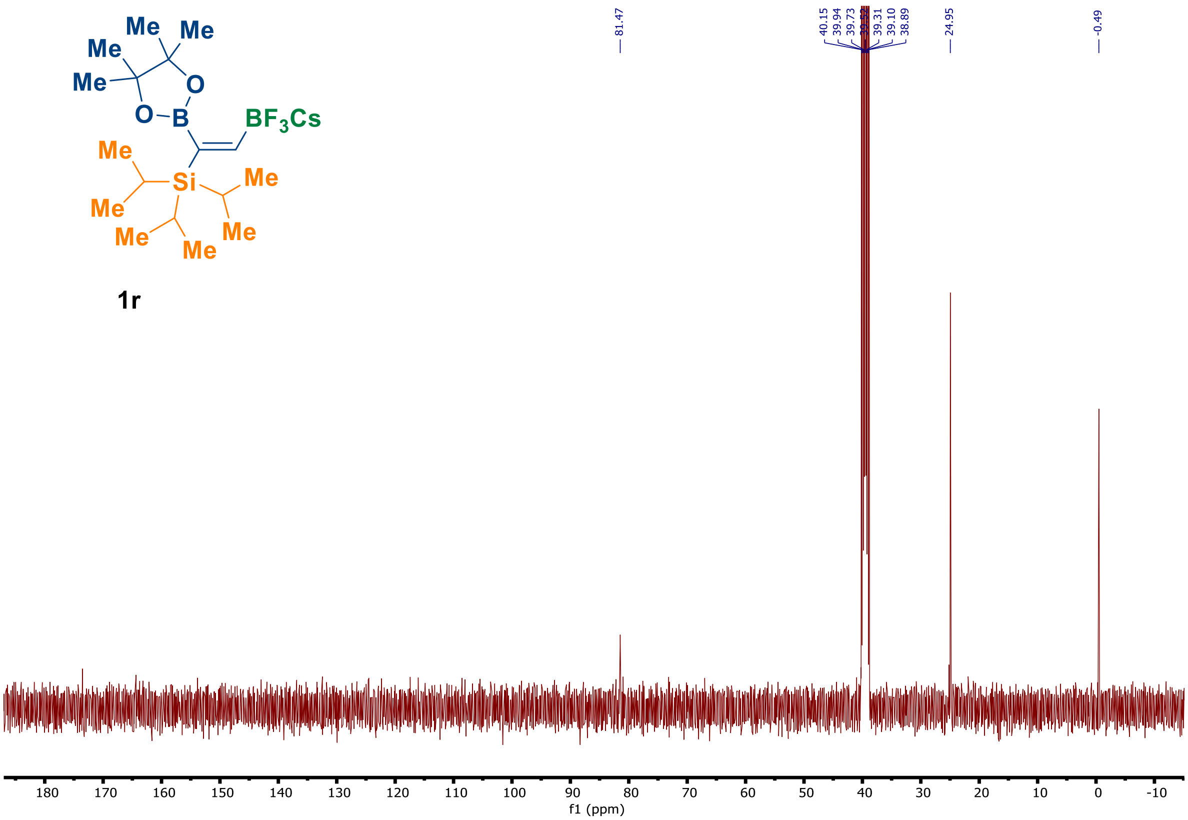

Supplementary Figure 108. ^{13}C NMR (101 MHz, DMSO-d_6) of compound (**1r**).

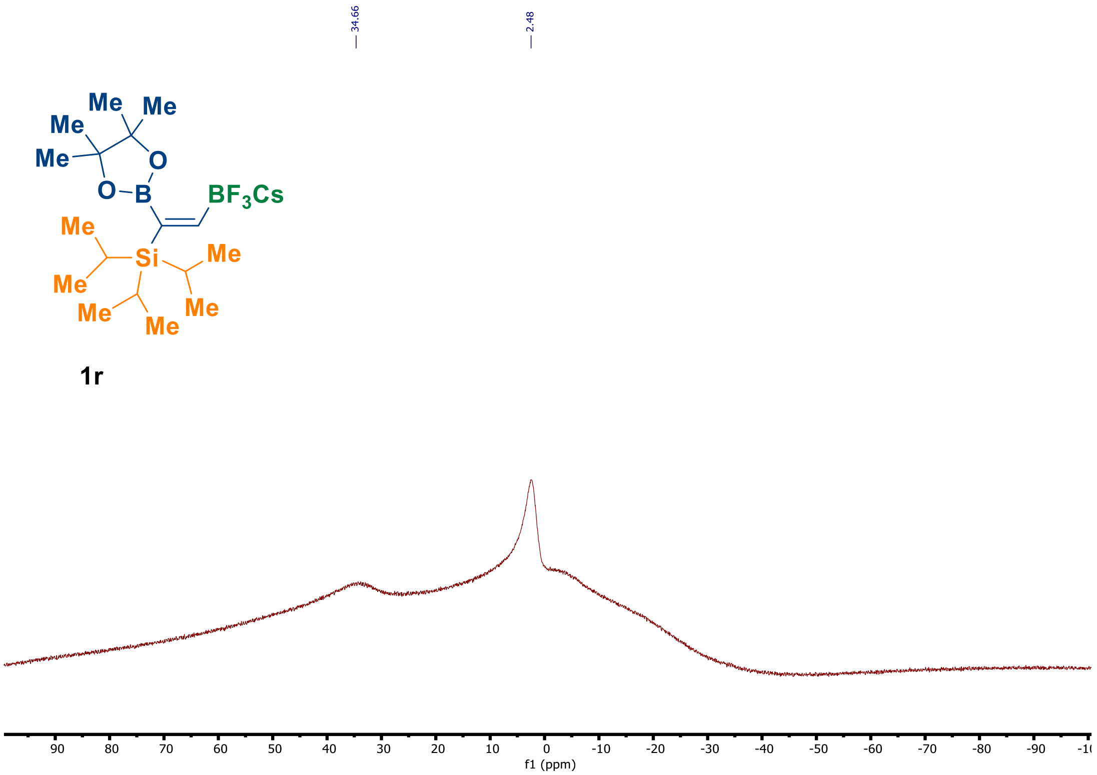

Supplementary Figure 109. ^{11}B NMR (128 MHz, DMSO-d_6) of compound (**1r**).

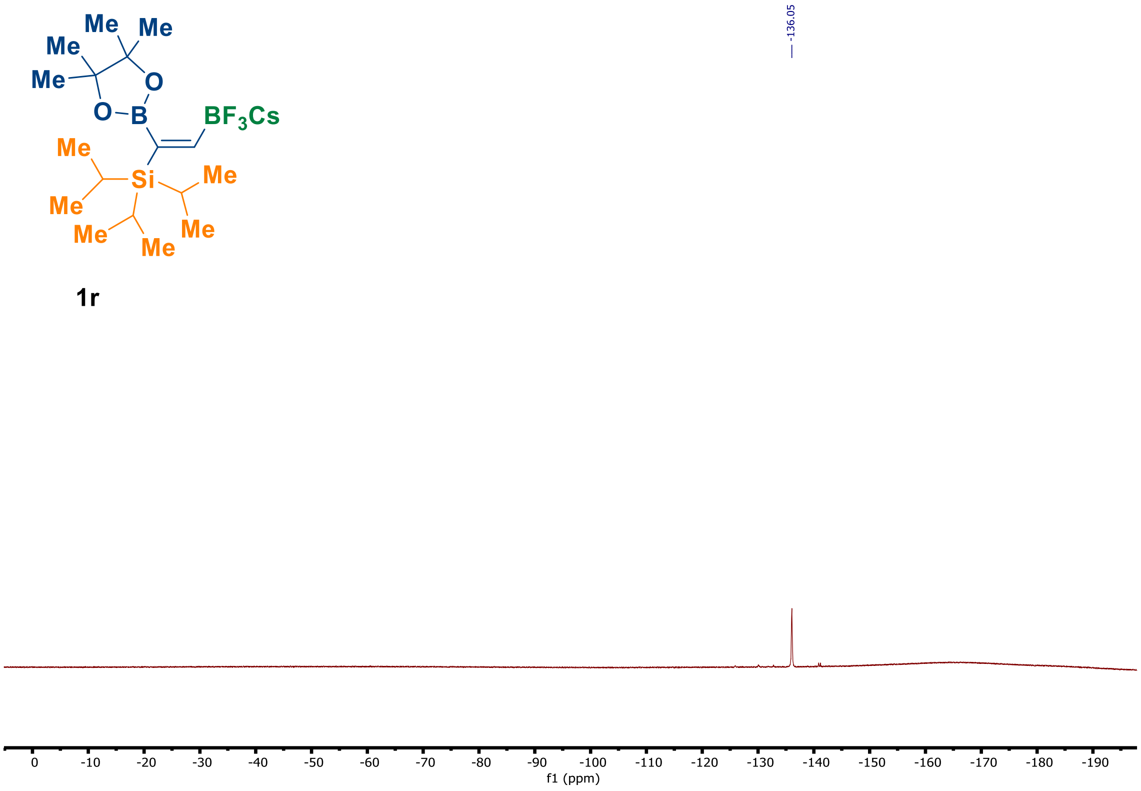

Supplementary Figure 110. ^{19}F NMR (376 MHz, DMSO-d_6) of compound (**1r**).

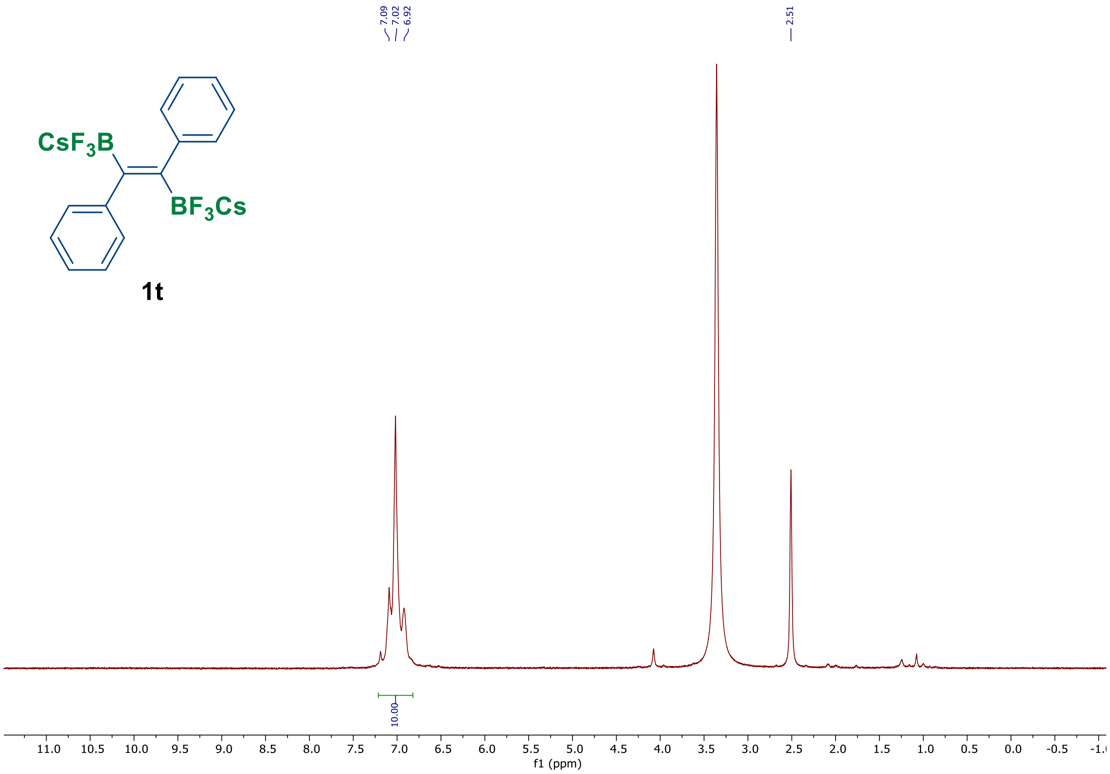

Supplementary Figure 111. ^{1}H NMR (400 MHz, DMSO-d_6) of compound (**1t**).

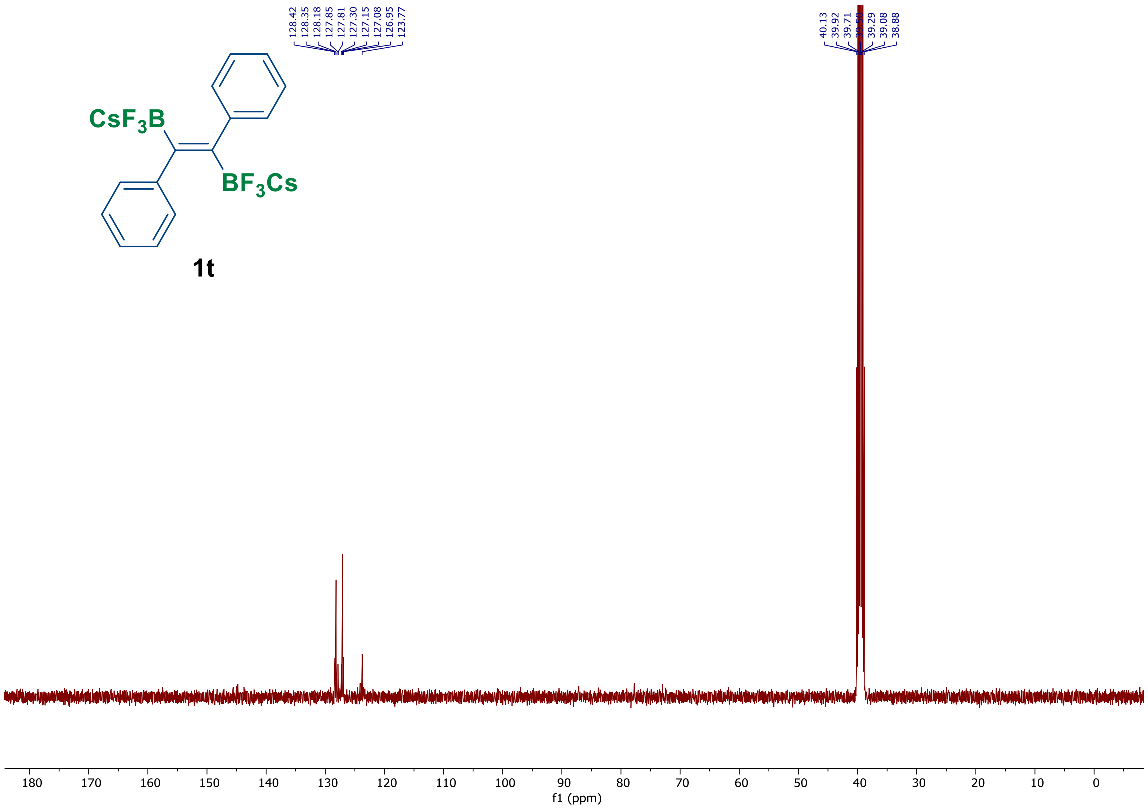

Supplementary Figure 112. ^{13}C NMR (101 MHz, DMSO-d_6) of compound (**1t**).

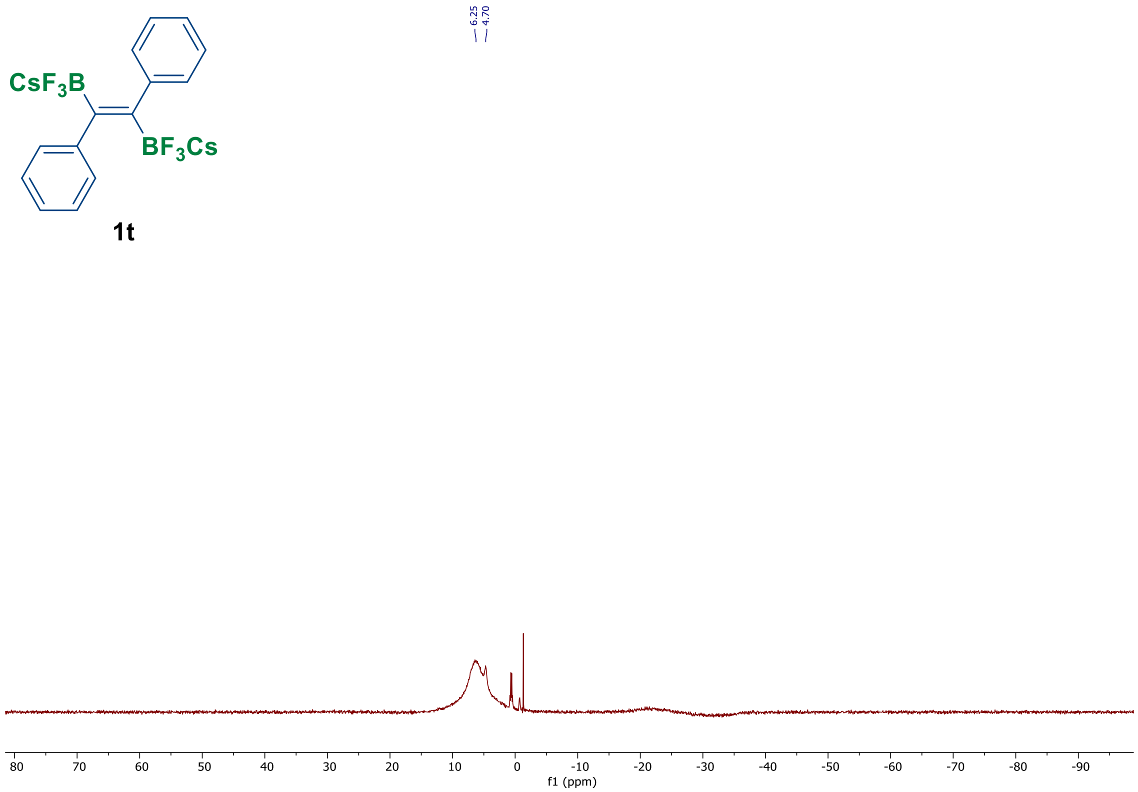

Supplementary Figure 113. ^{11}B NMR (128 MHz, DMSO-d_6) of compound (**1t**).

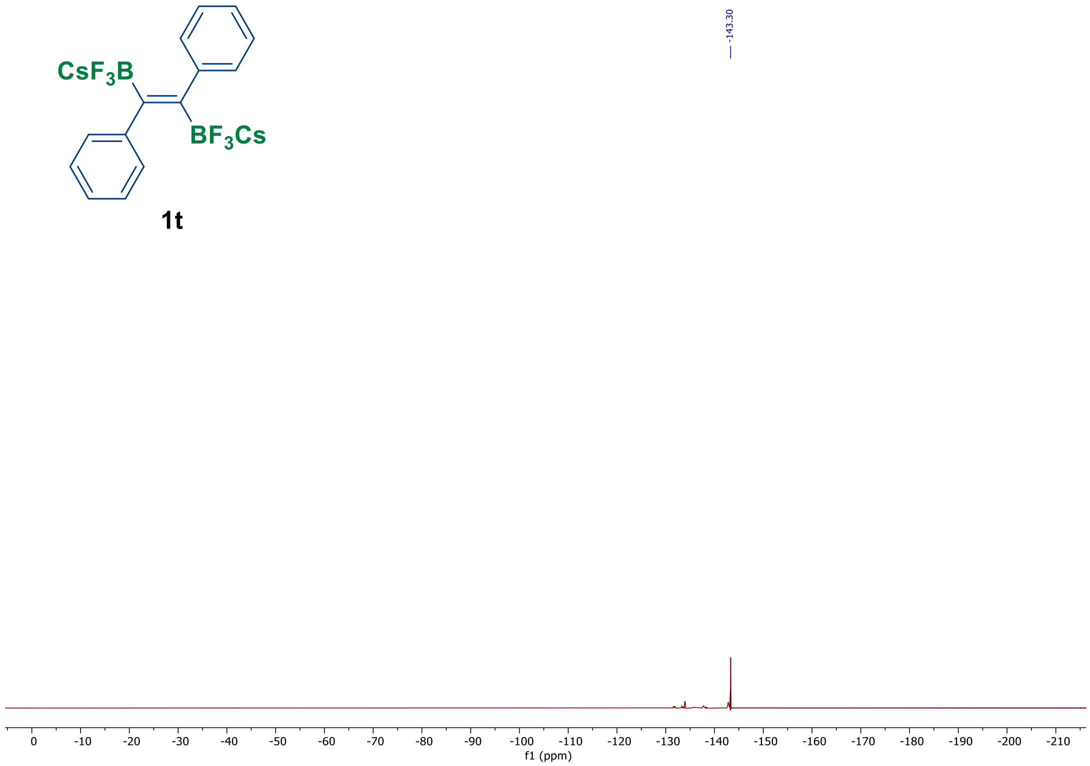

Supplementary Figure 114. ^{19}F NMR (376 MHz, DMSO-d_6) of compound (**1t**).

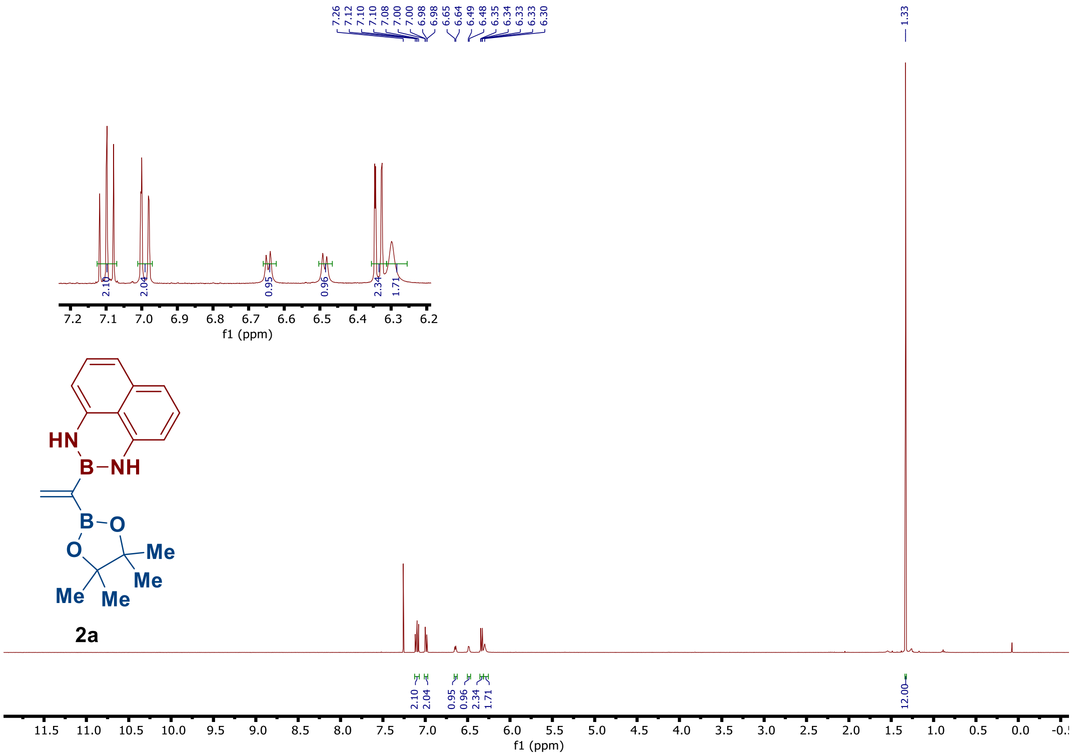

Supplementary Figure 115. ^{1}H NMR (400 MHz, $CDCl_3$) of compound (**2a**).

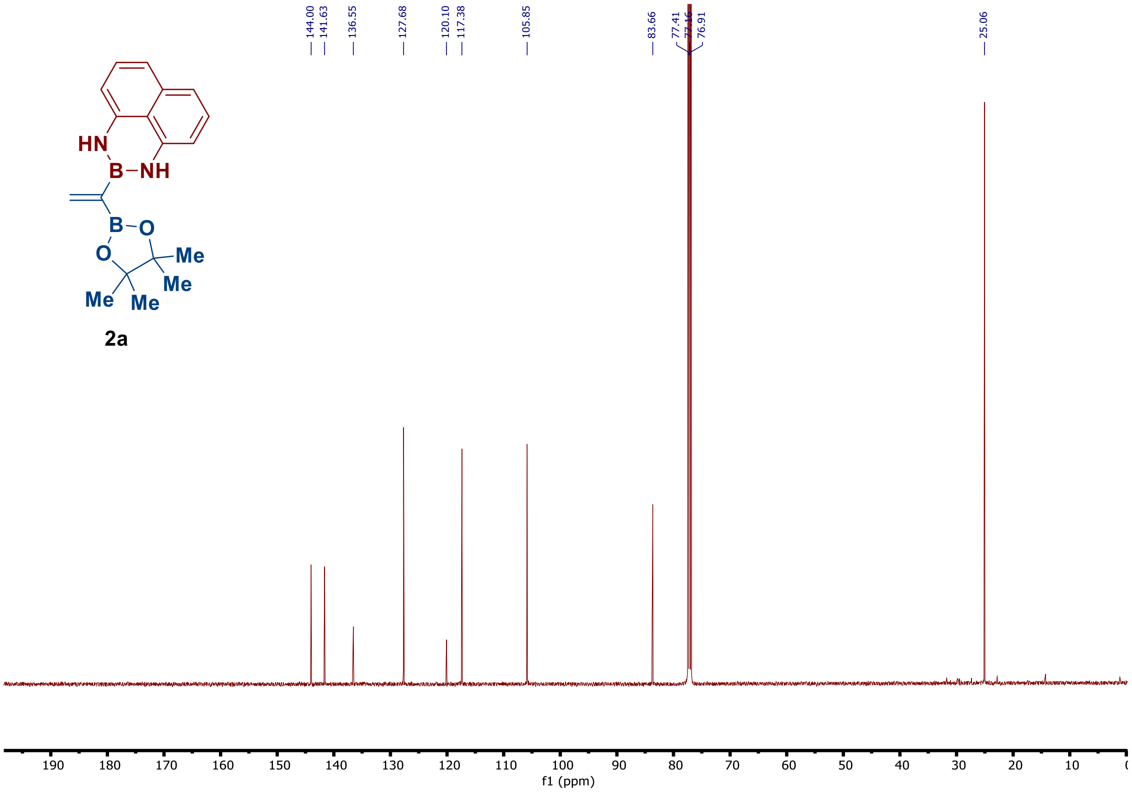

Supplementary Figure 116. ^{13}C NMR (101 MHz, $CDCl_3$) of compound (**2a**).

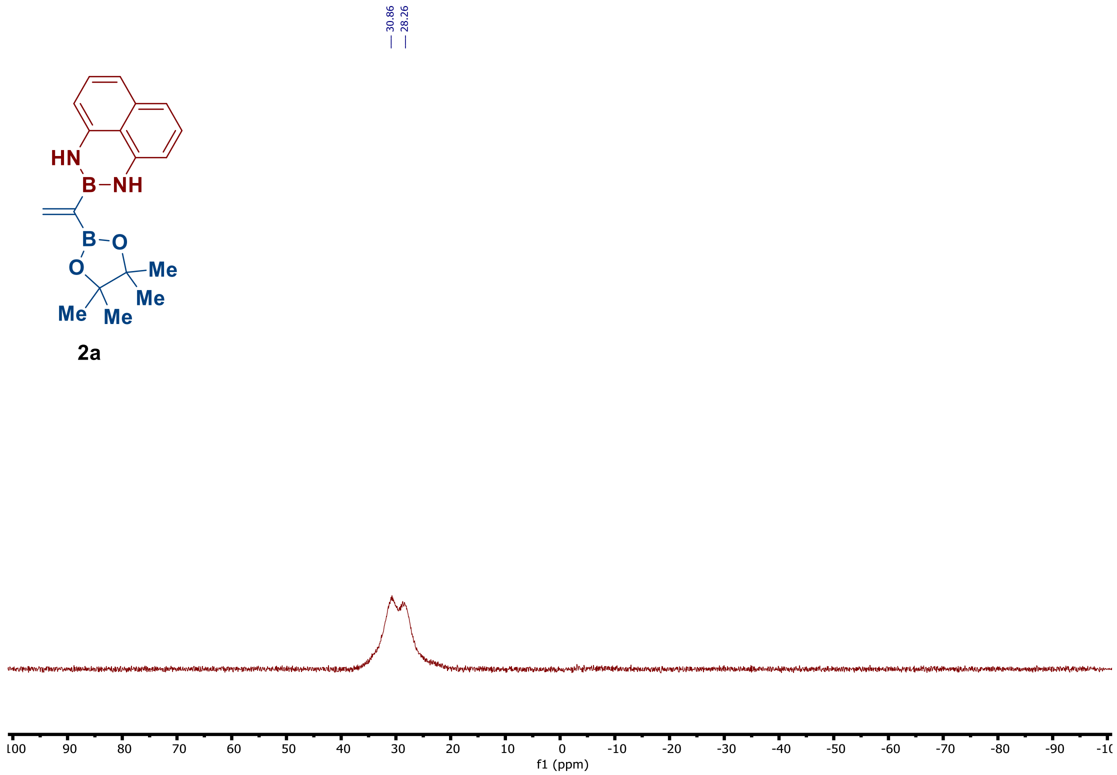

Supplementary Figure 117. ^{11}B NMR (128 MHz, $CDCl_3$) of compound (**2a**).

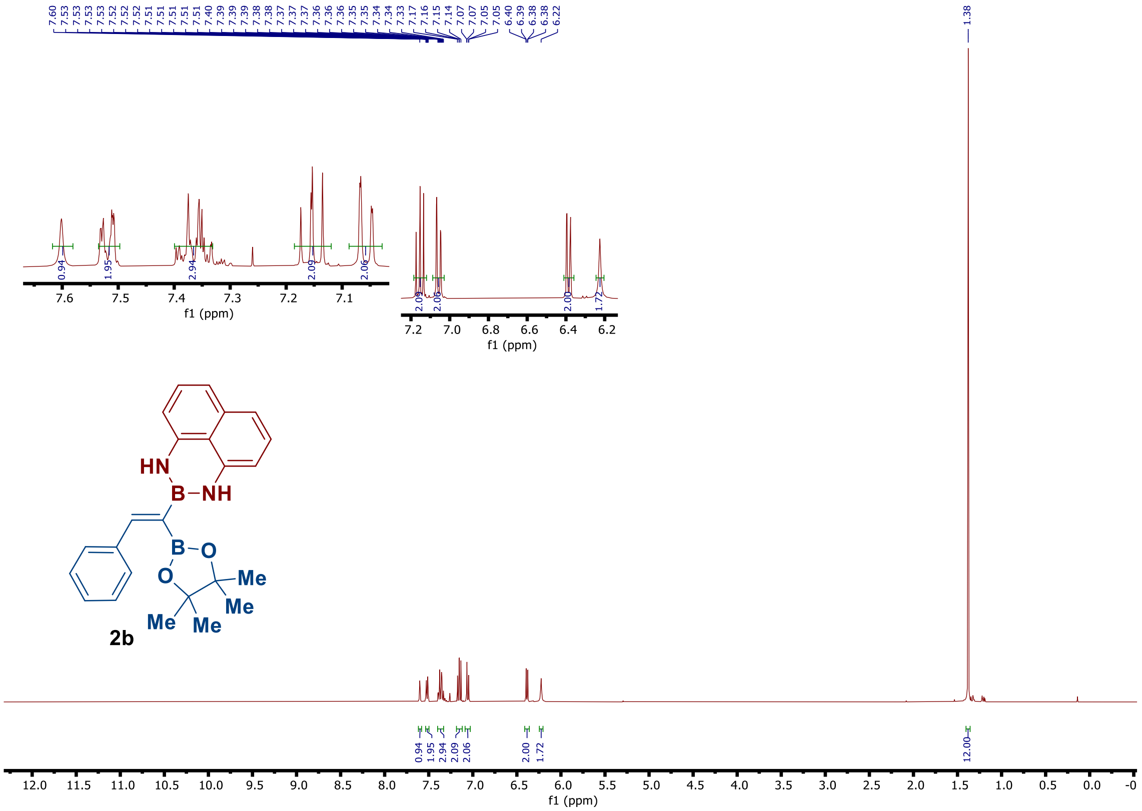

Supplementary Figure 118. ^{1}H NMR (400 MHz, $CDCl_3$) of compound (**2b**).

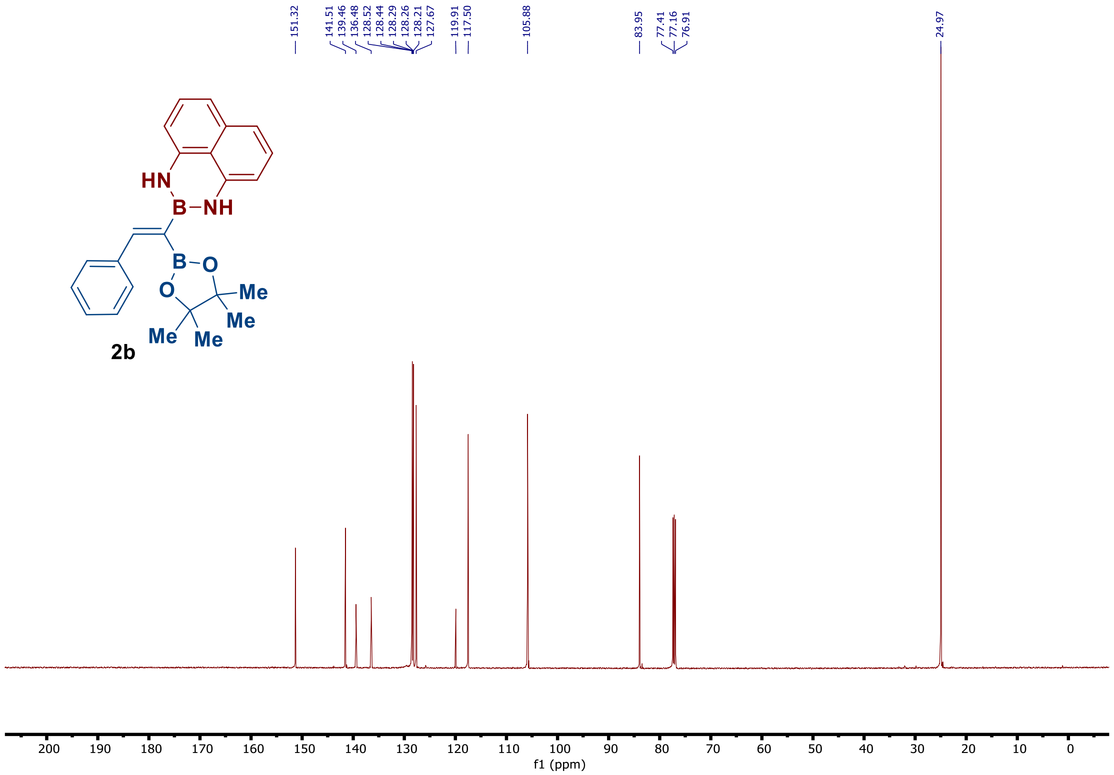

Supplementary Figure 119. ^{13}C NMR (101 MHz, $CDCl_3$) of compound (**2b**).

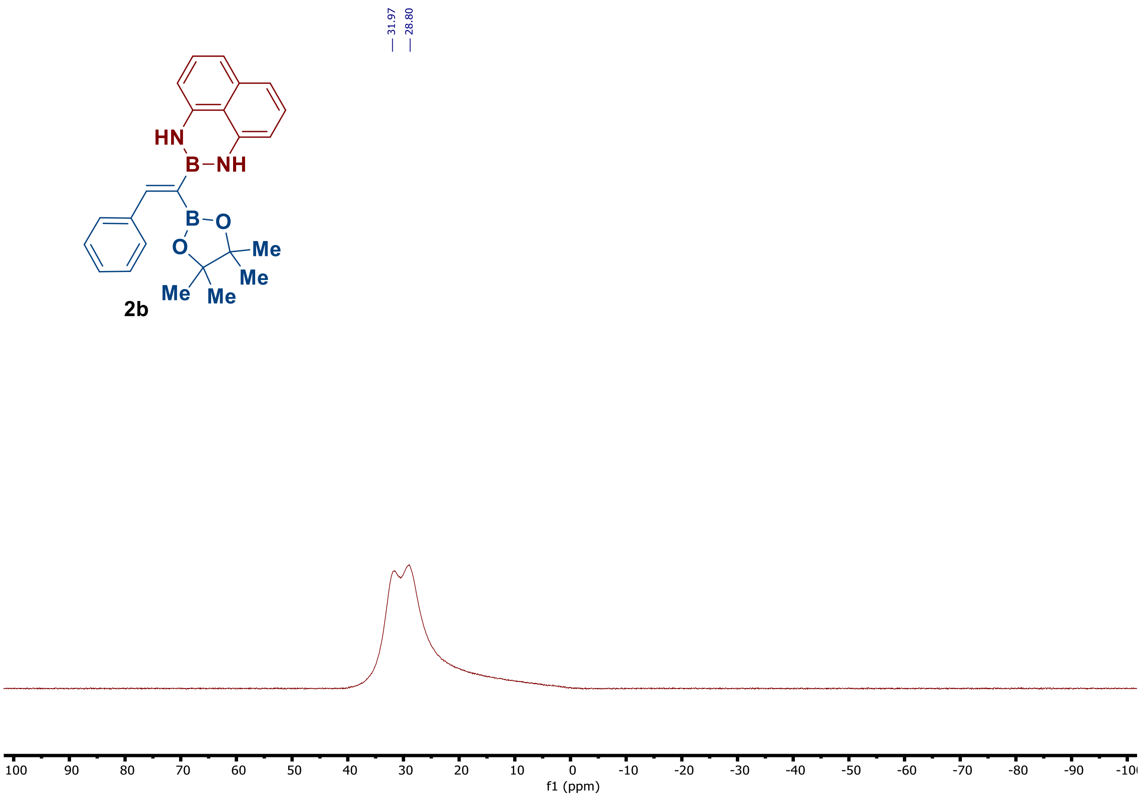

Supplementary Figure 120. ^{11}B NMR (128 MHz, $CDCl_3$) of compound (**2b**).

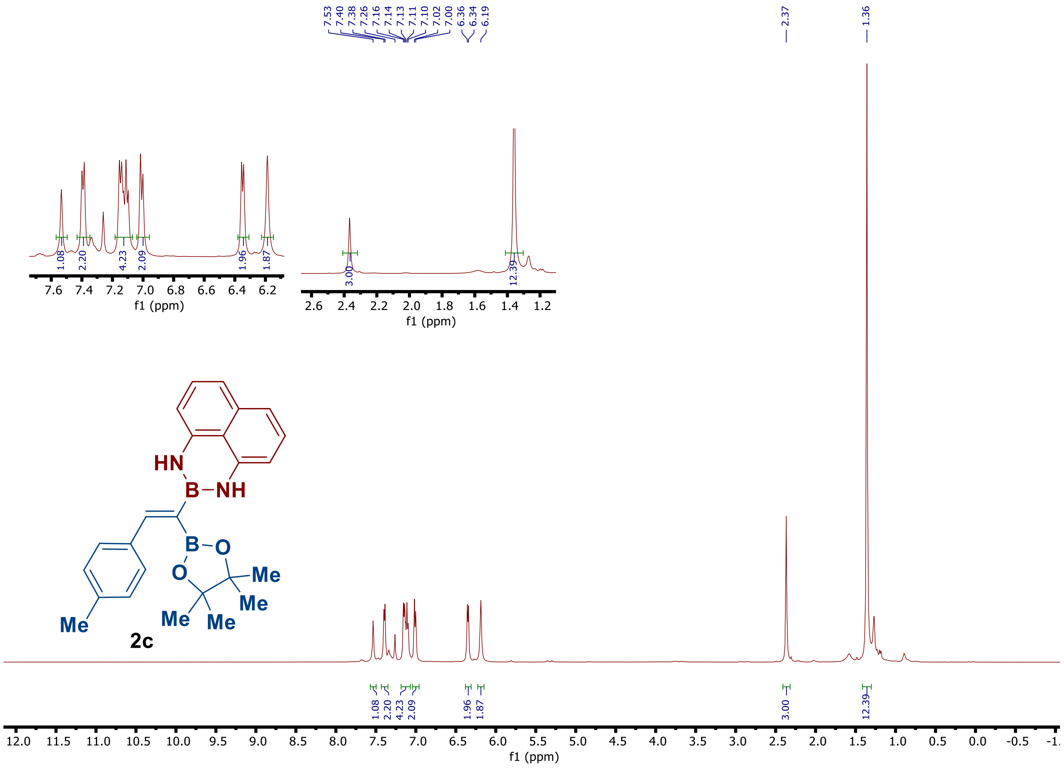

Supplementary Figure 121. ^{1}H NMR (400 MHz, $CDCl_3$) of compound (**2c**).

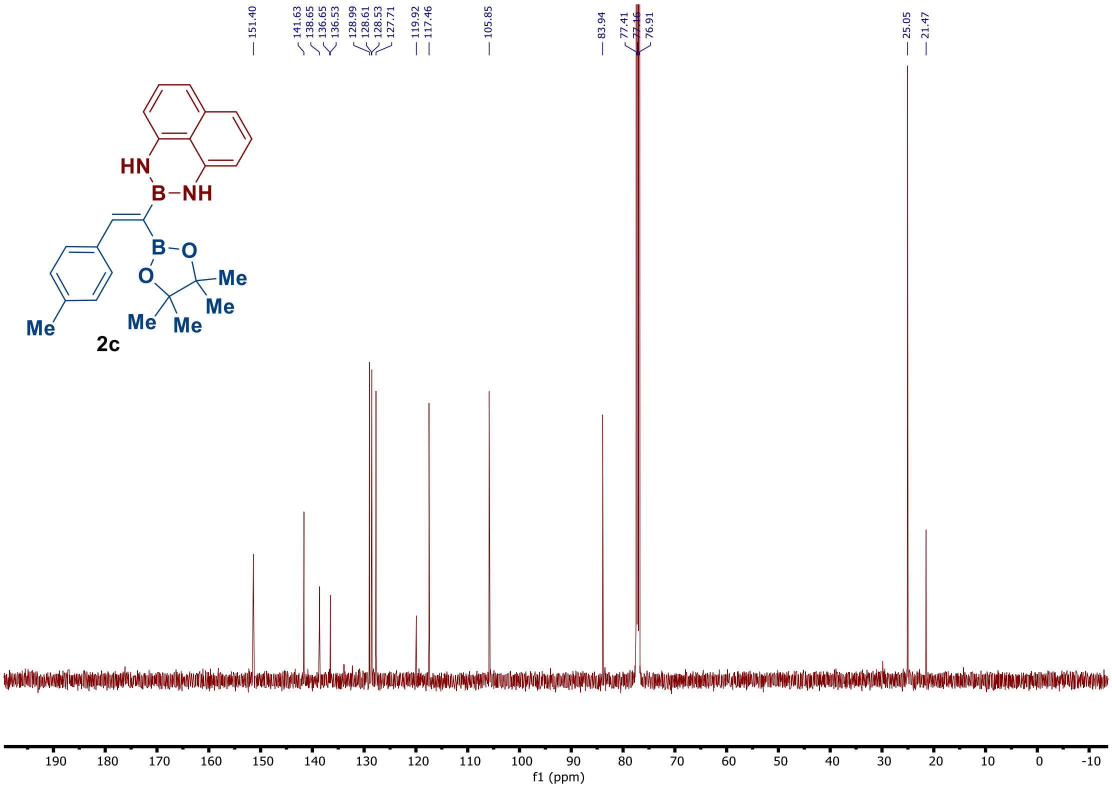

Supplementary Figure 122. ^{13}C NMR (101 MHz, $CDCl_3$) of compound (**2c**).

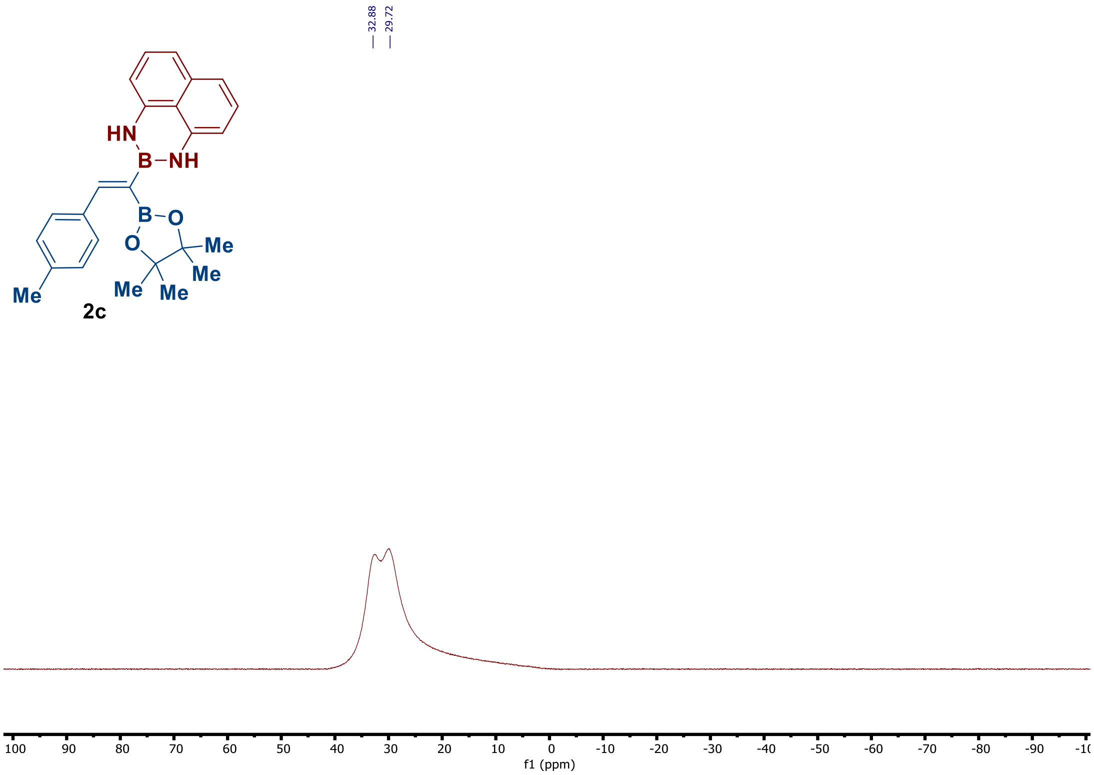

Supplementary Figure 123. ^{11}B NMR (128 MHz, $CDCl_3$) of compound (**2c**).

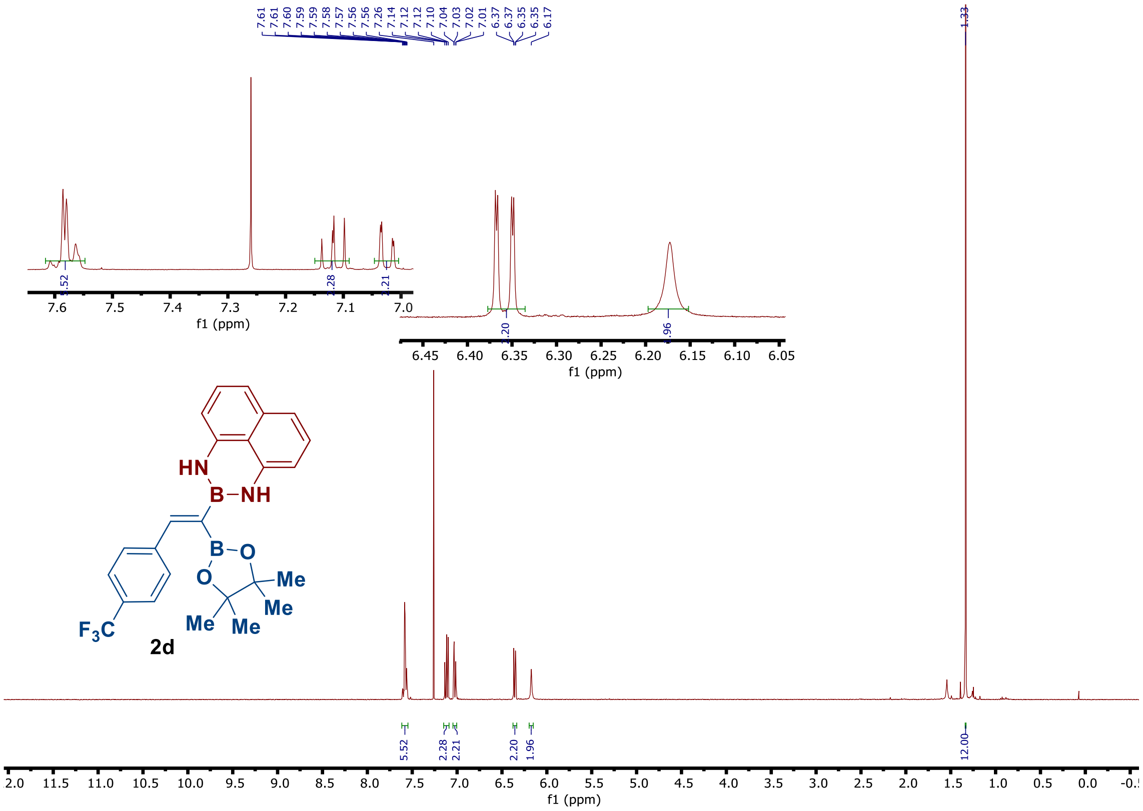

Supplementary Figure 124. ^{1}H NMR (400 MHz, $CDCl_3$) of compound (**2d**).

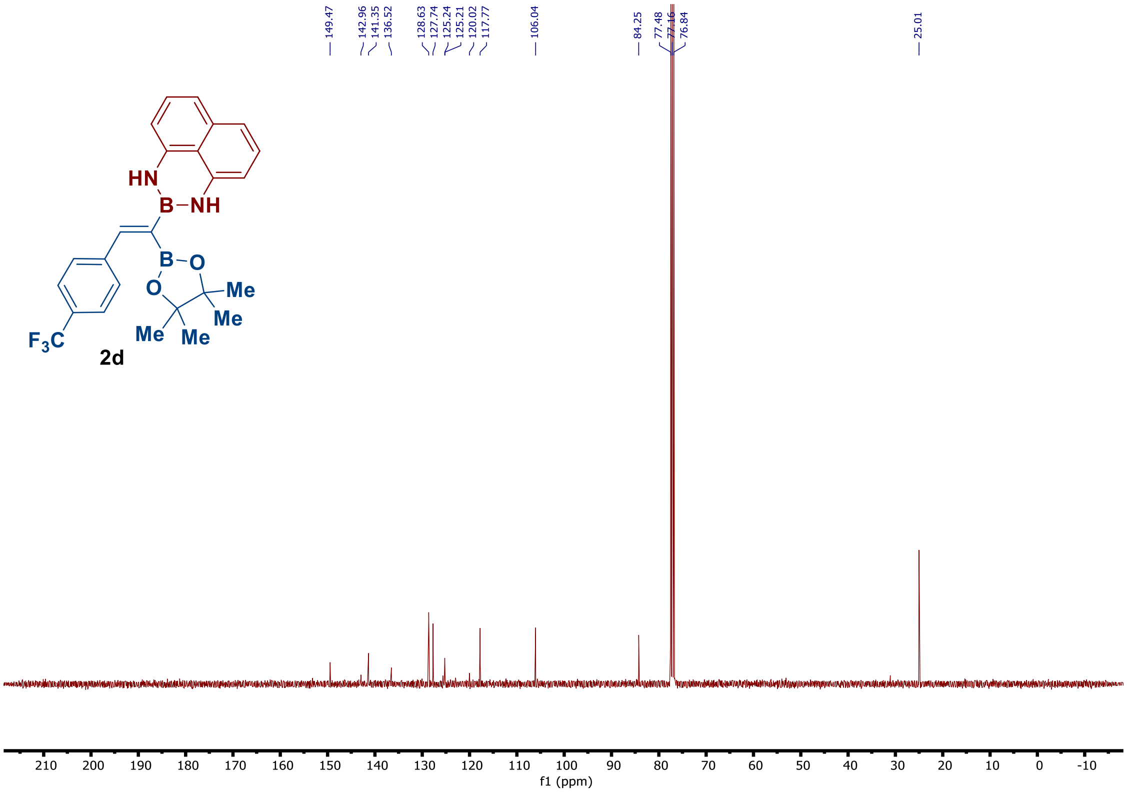

Supplementary Figure 125. ^{13}C NMR (101 MHz, $CDCl_3$) of compound (**2d**).

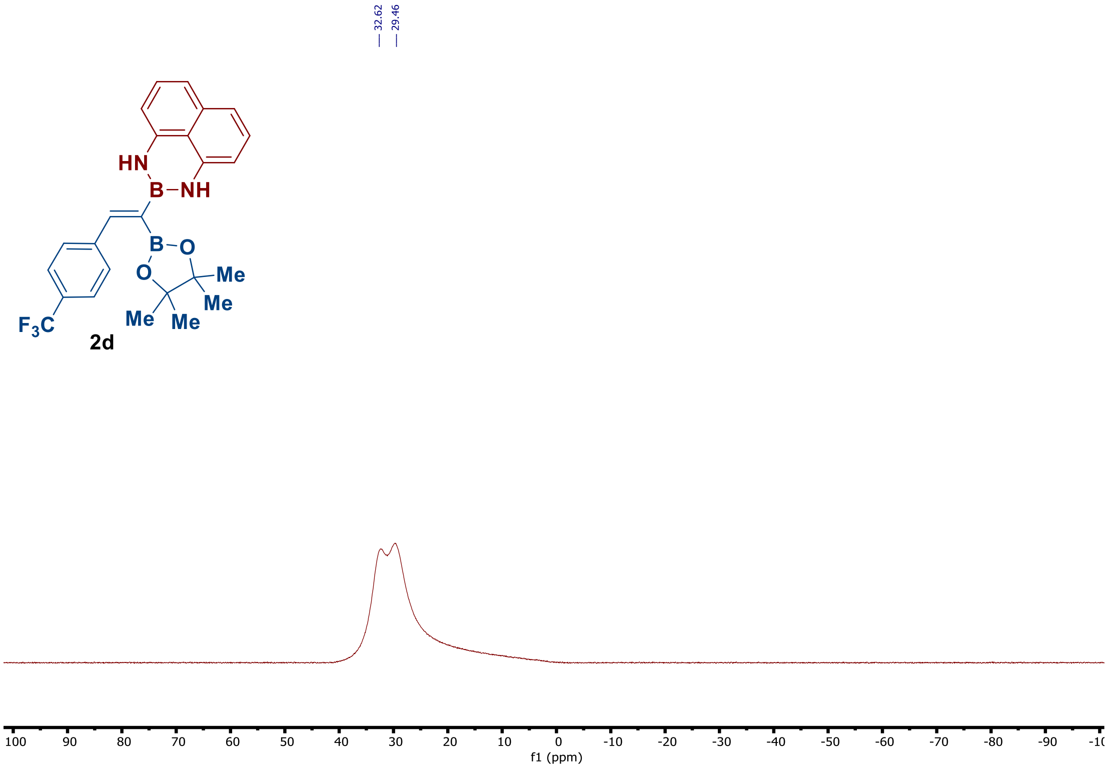

Supplementary Figure 126. ^{11}B NMR (128 MHz, $CDCl_3$) of compound (**2d**).

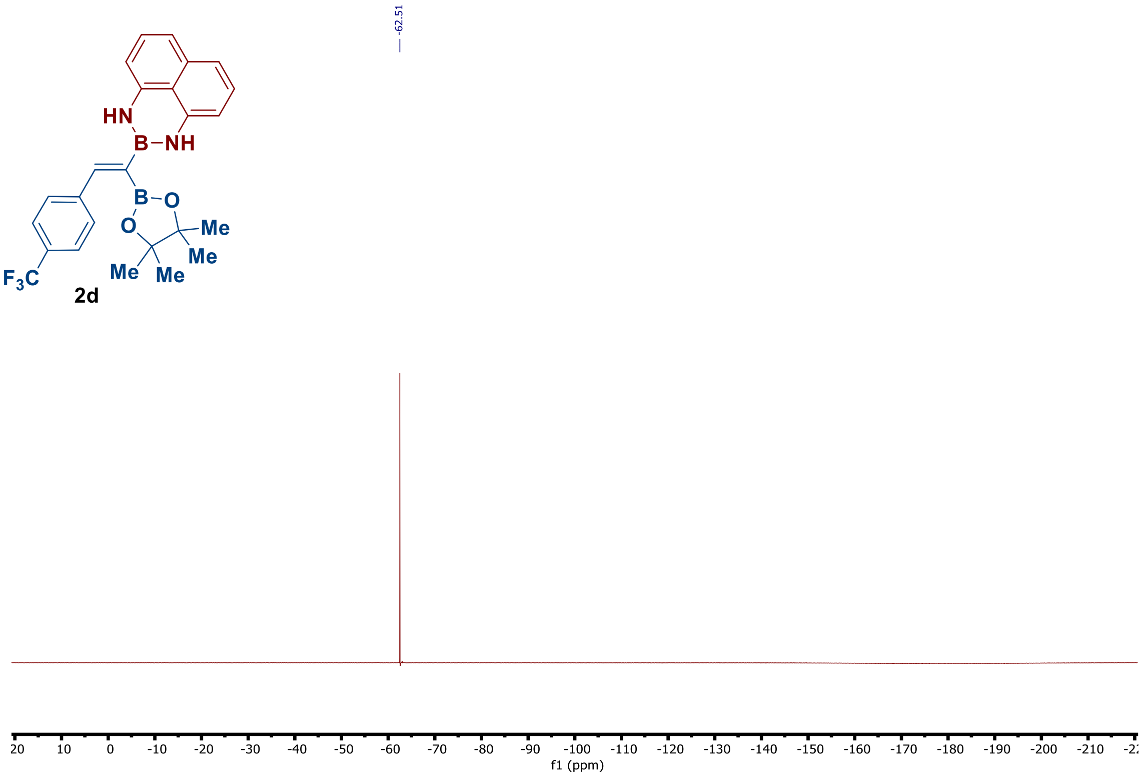

Supplementary Figure 127. ^{19}F NMR (376 MHz, $CDCl_3$) of compound (**2d**).

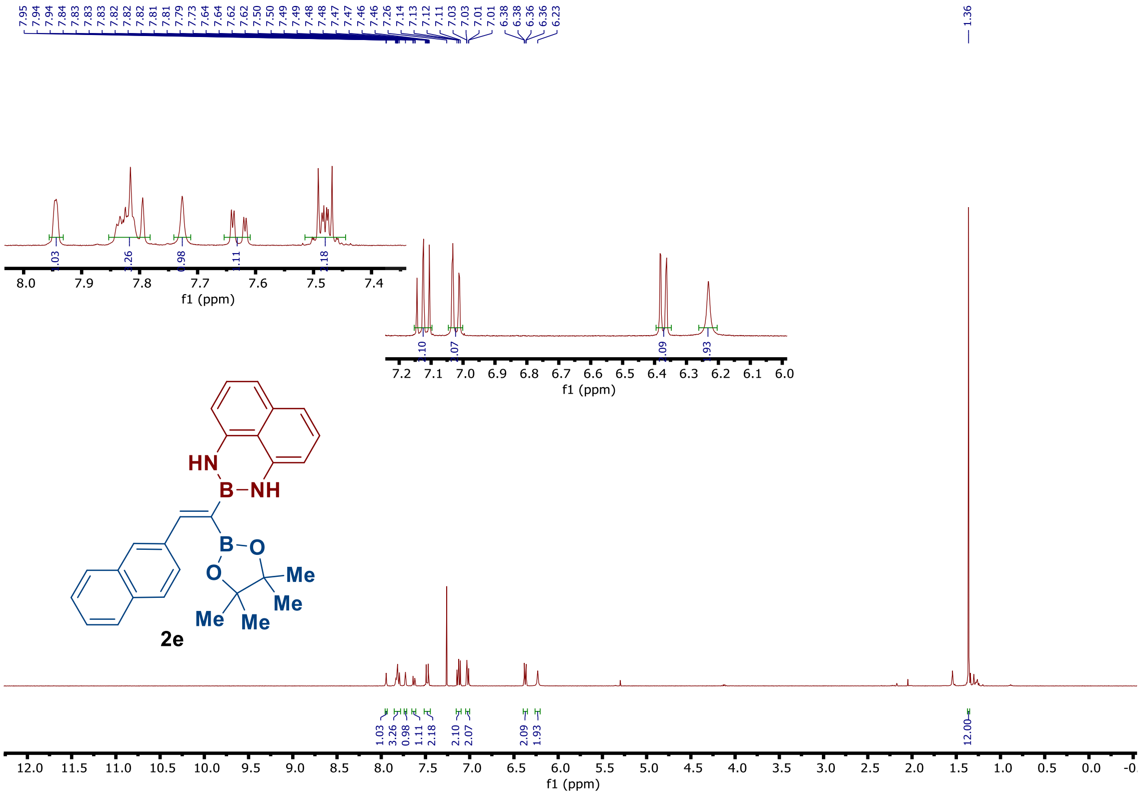

Supplementary Figure 128. ^{1}H NMR (400 MHz, $CDCl_3$) of compound (**2e**).

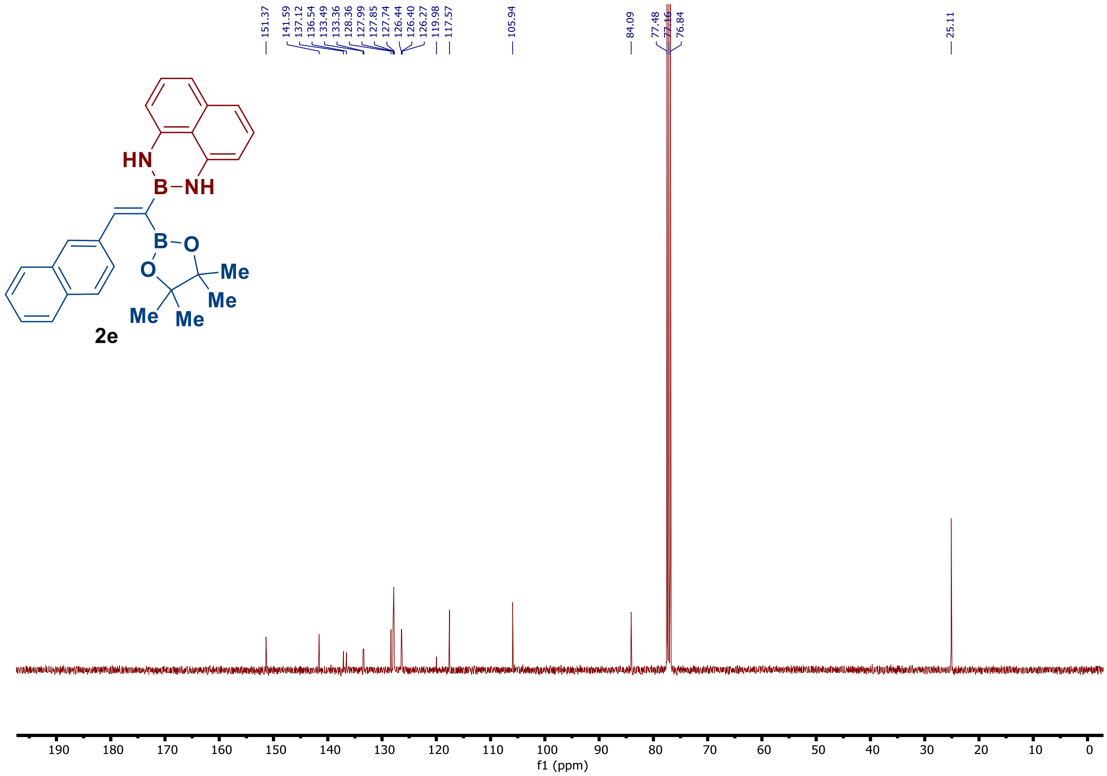

Supplementary Figure 129. ^{13}C NMR (101 MHz, $CDCl_3$) of compound (**2e**).

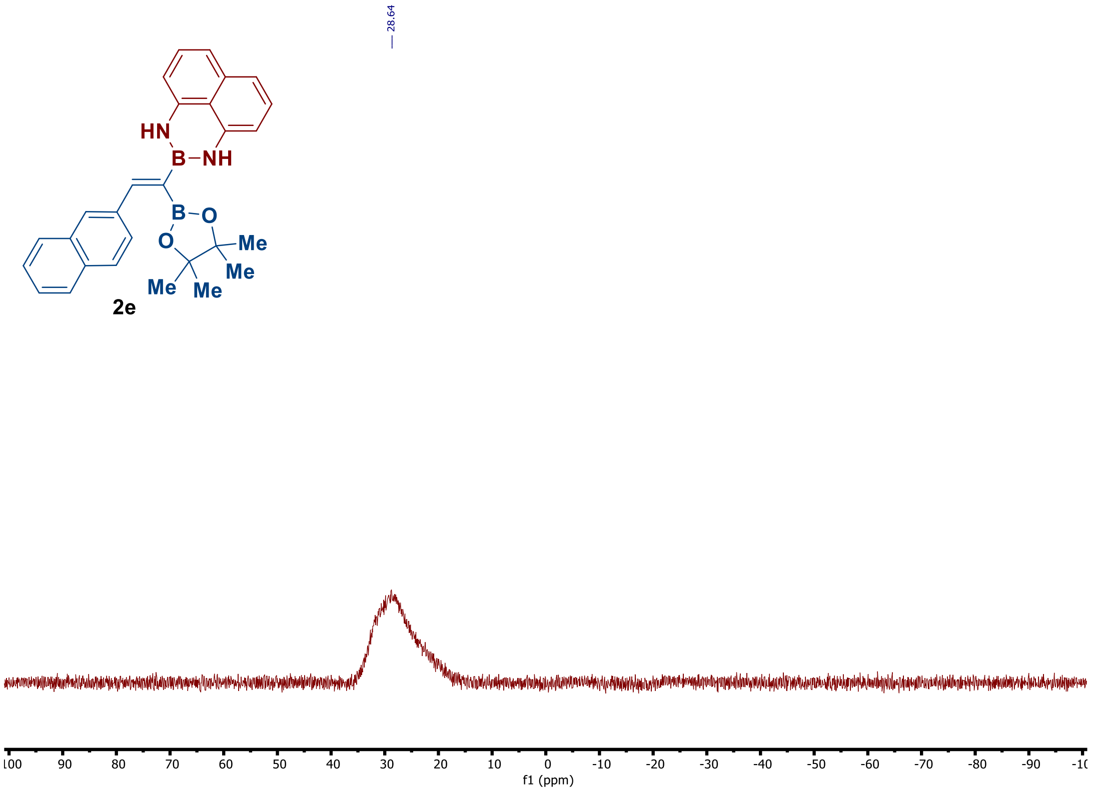

Supplementary Figure 130. ^{11}B NMR (128 MHz, $CDCl_3$) of compound (**2e**).

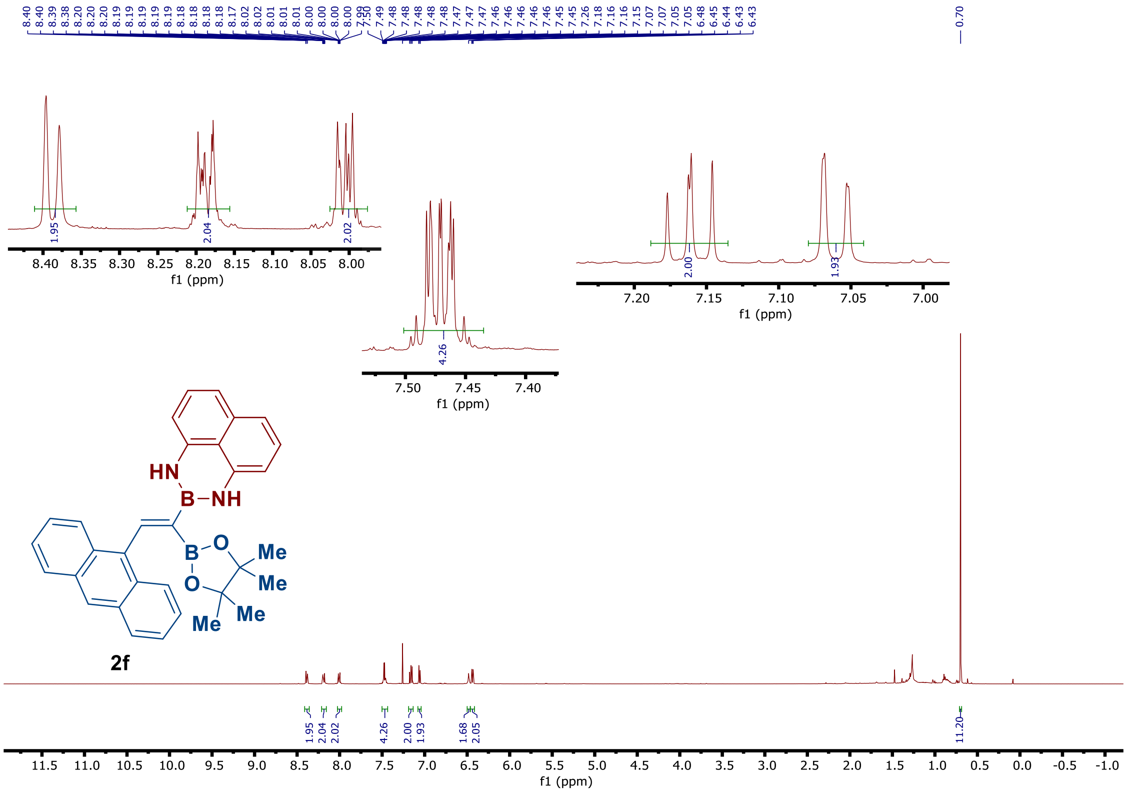

Supplementary Figure 131. ^{1}H NMR (400 MHz, $CDCl_3$) of compound (**2f**).

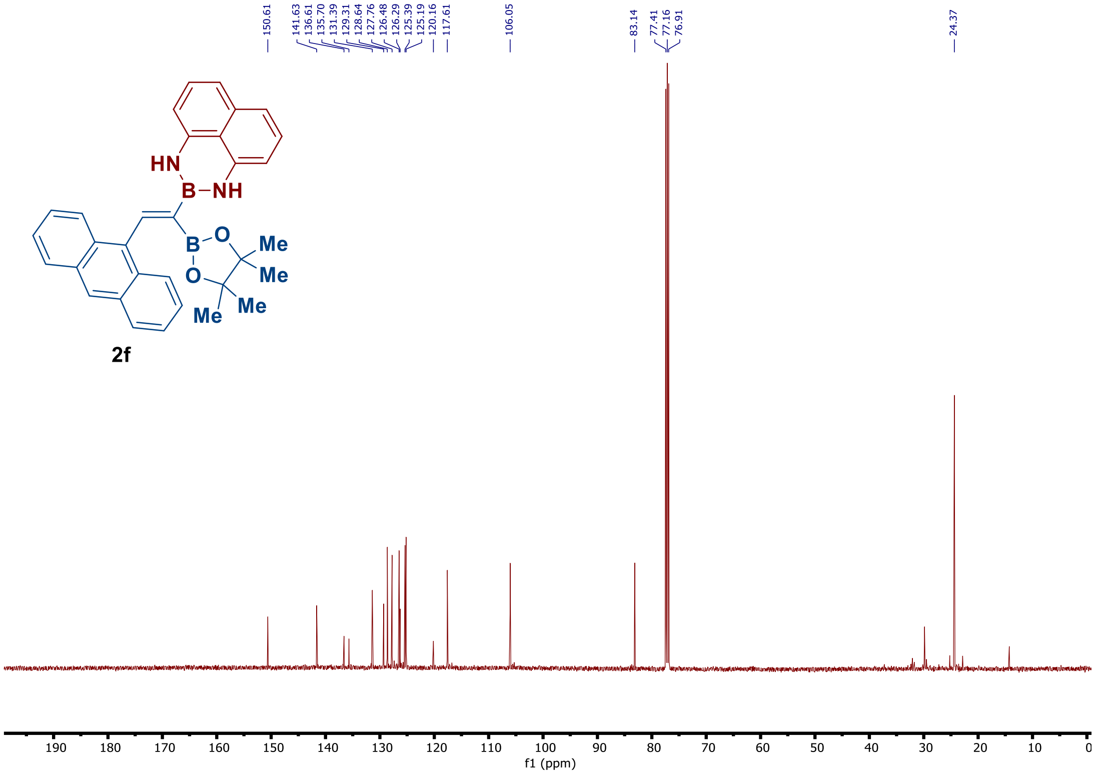

Supplementary Figure 132. ^{13}C NMR (101 MHz, $CDCl_3$) of compound (**2f**).

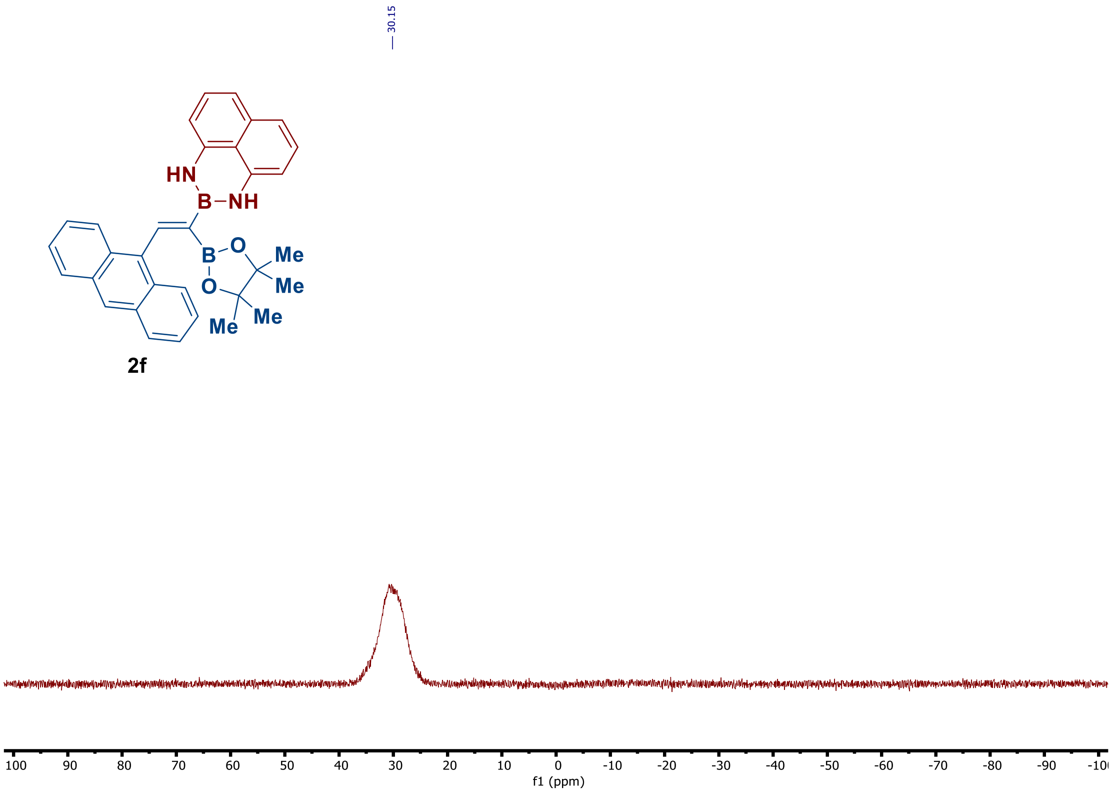

Supplementary Figure 133. ^{11}B NMR (128 MHz, $CDCl_3$) of compound (**2f**).

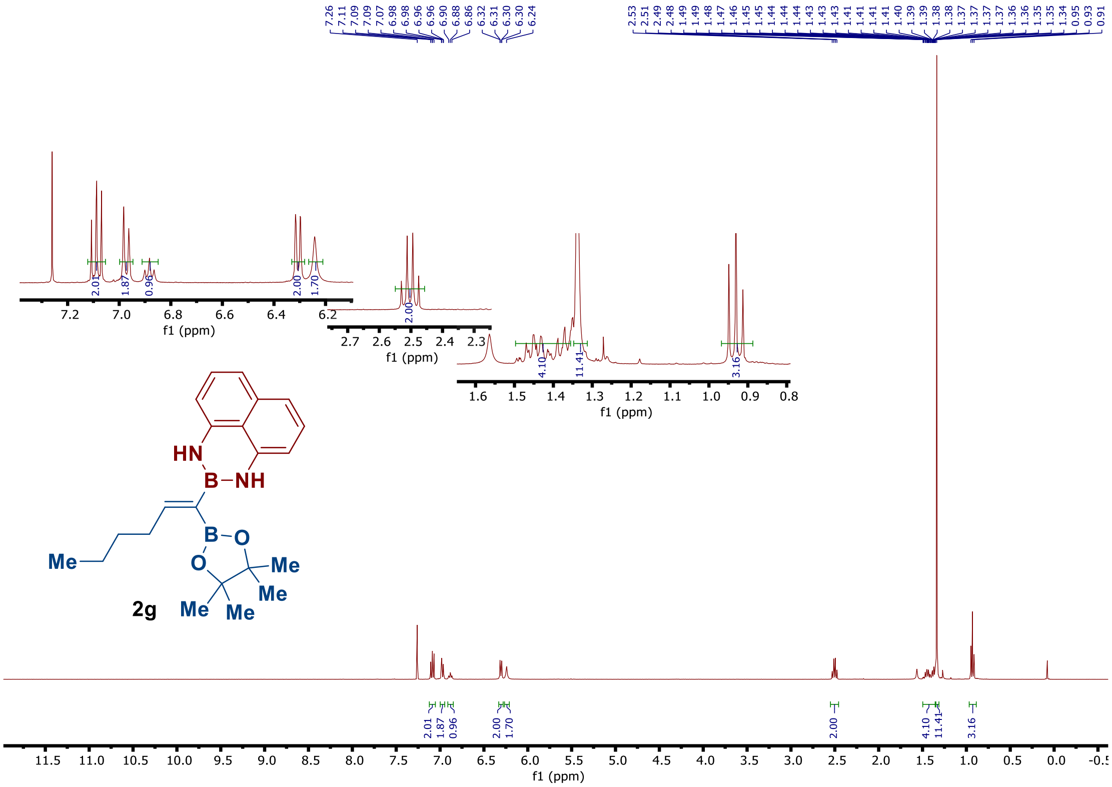

Supplementary Figure 134. ^{1}H NMR (400 MHz, $CDCl_3$) of compound (**2g**).

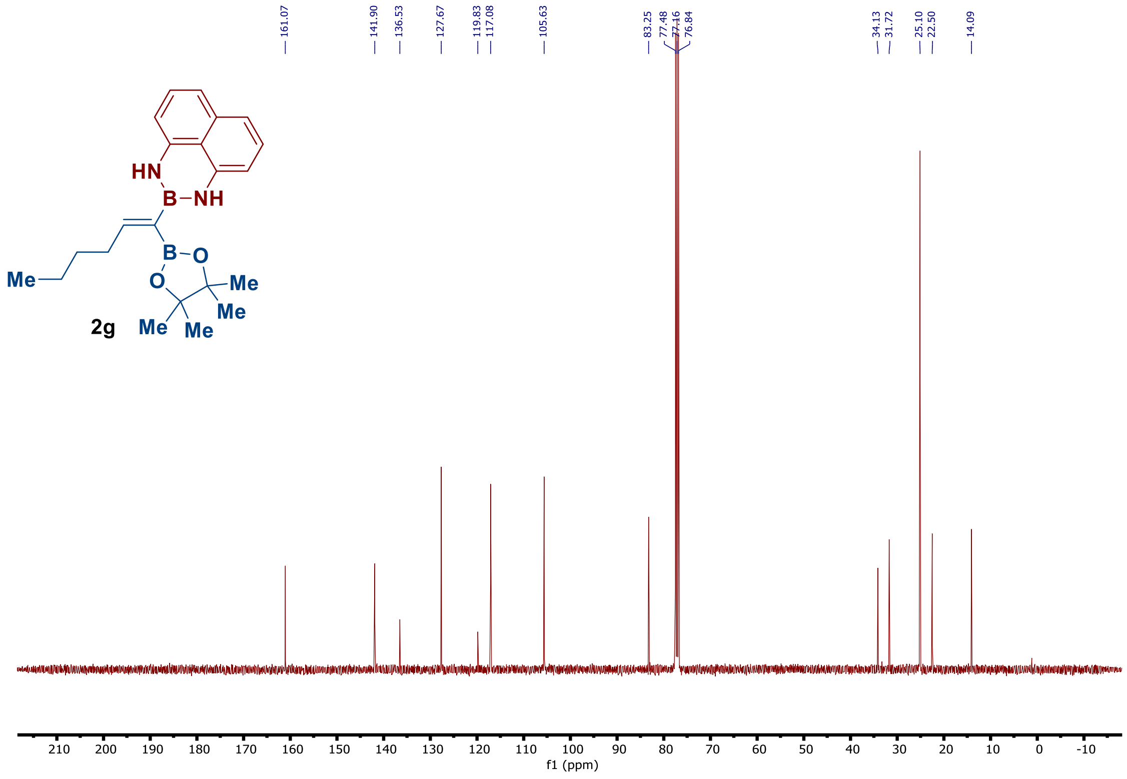

Supplementary Figure 135. ^{13}C NMR (101 MHz, $CDCl_3$) of compound (**2g**).

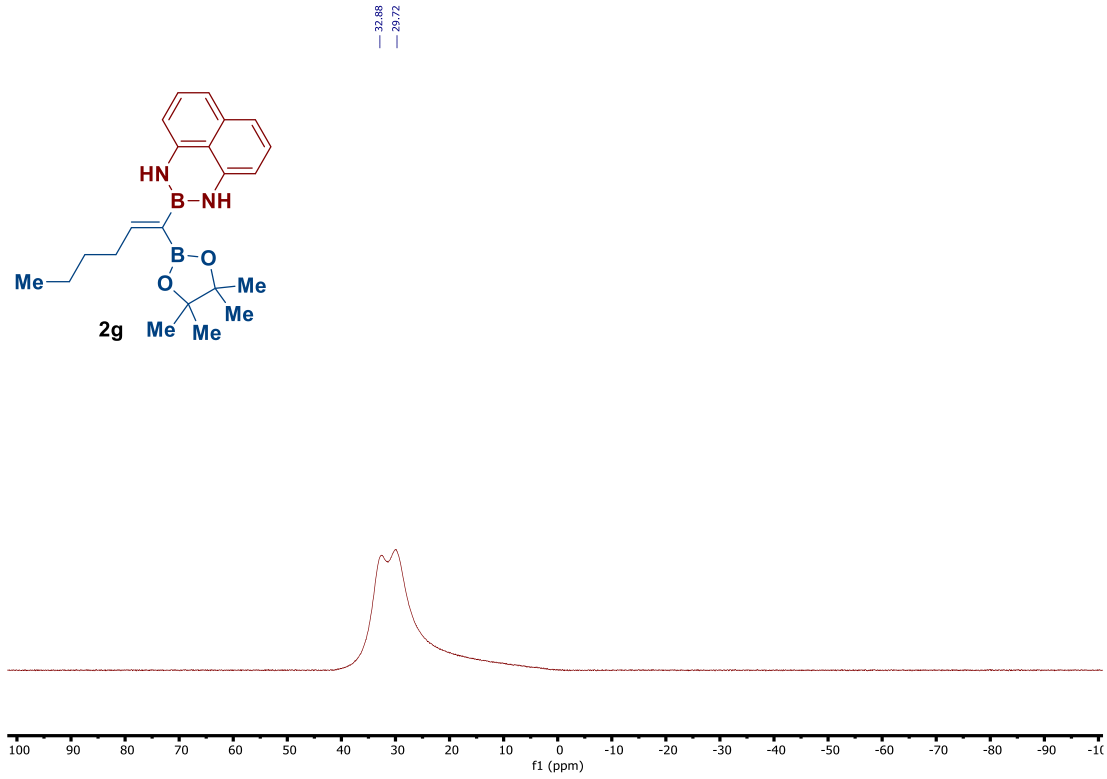

Supplementary Figure 136. ^{11}B NMR (128 MHz, $CDCl_3$) of compound (**2g**).

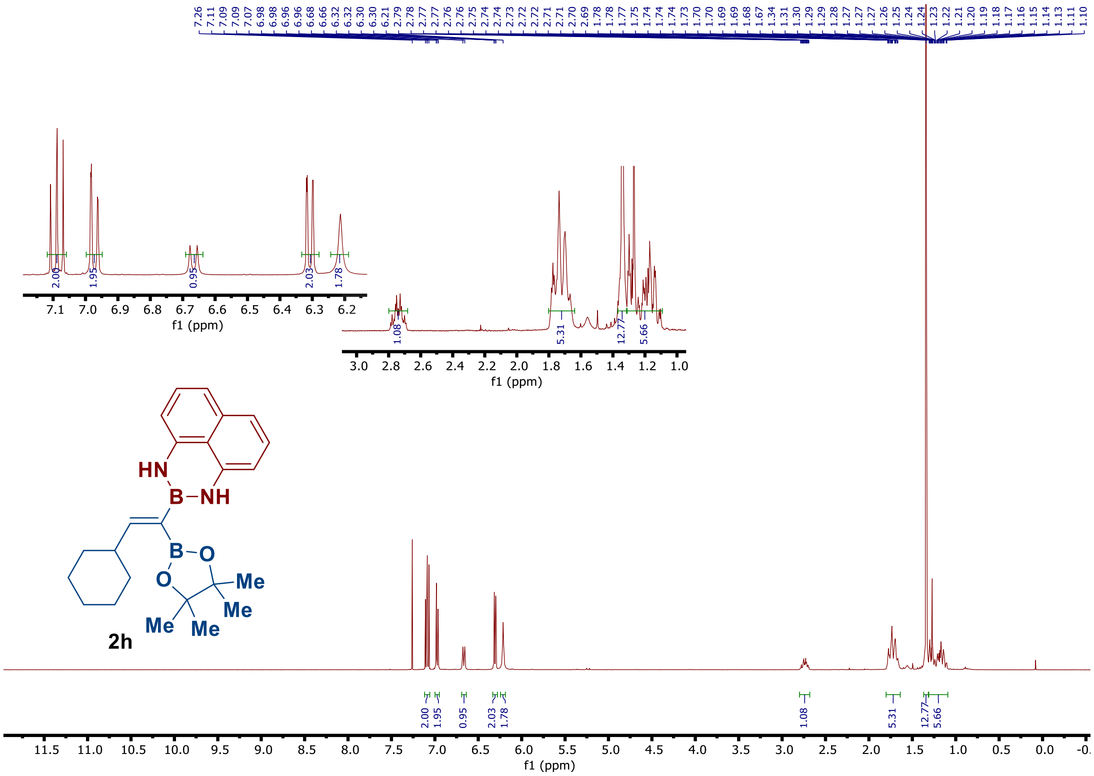

Supplementary Figure 137. ^{1}H NMR (400 MHz, $CDCl_3$) of compound (**2h**).

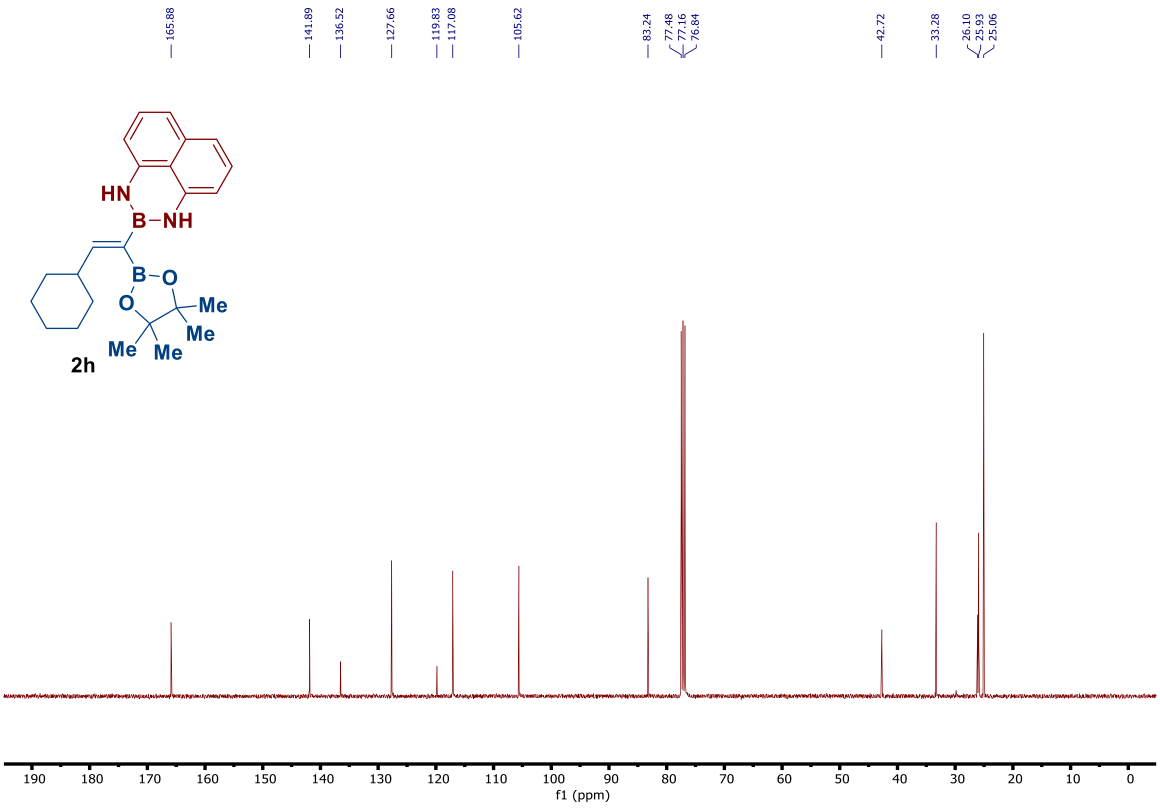

Supplementary Figure 138. ^{13}C NMR (101 MHz, $CDCl_3$) of compound (**2h**).

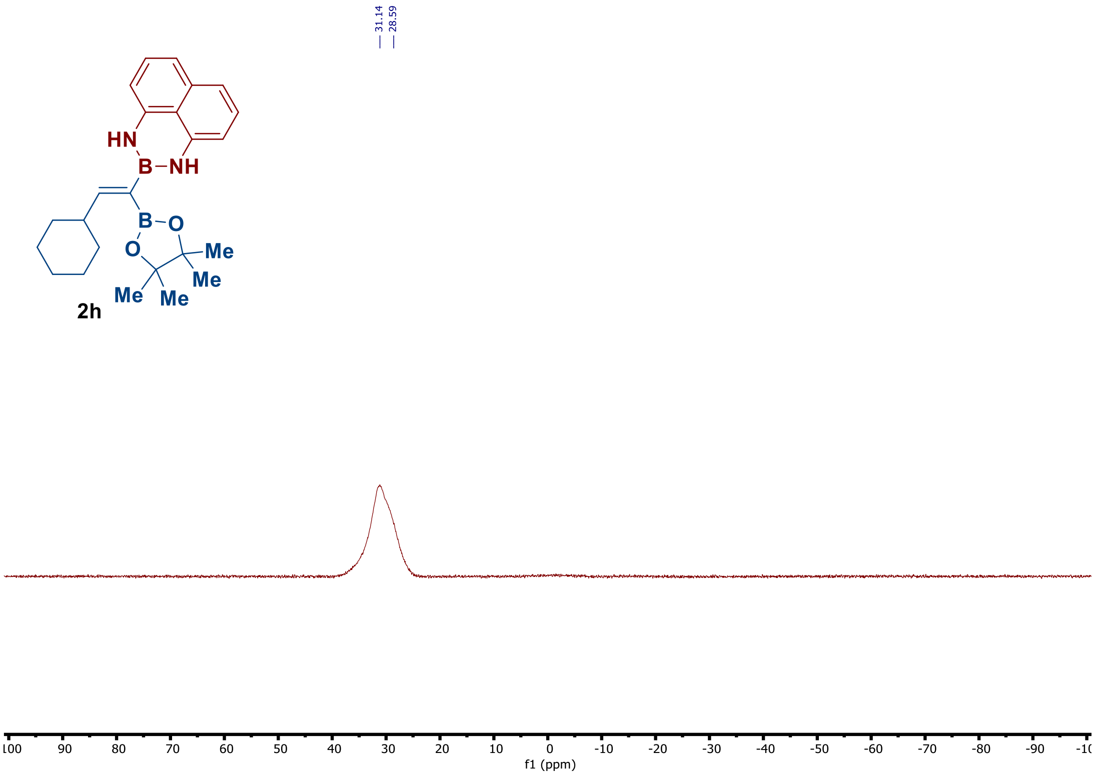

Supplementary Figure 139. ^{11}B NMR (128 MHz, $CDCl_3$) of compound (**2h**).

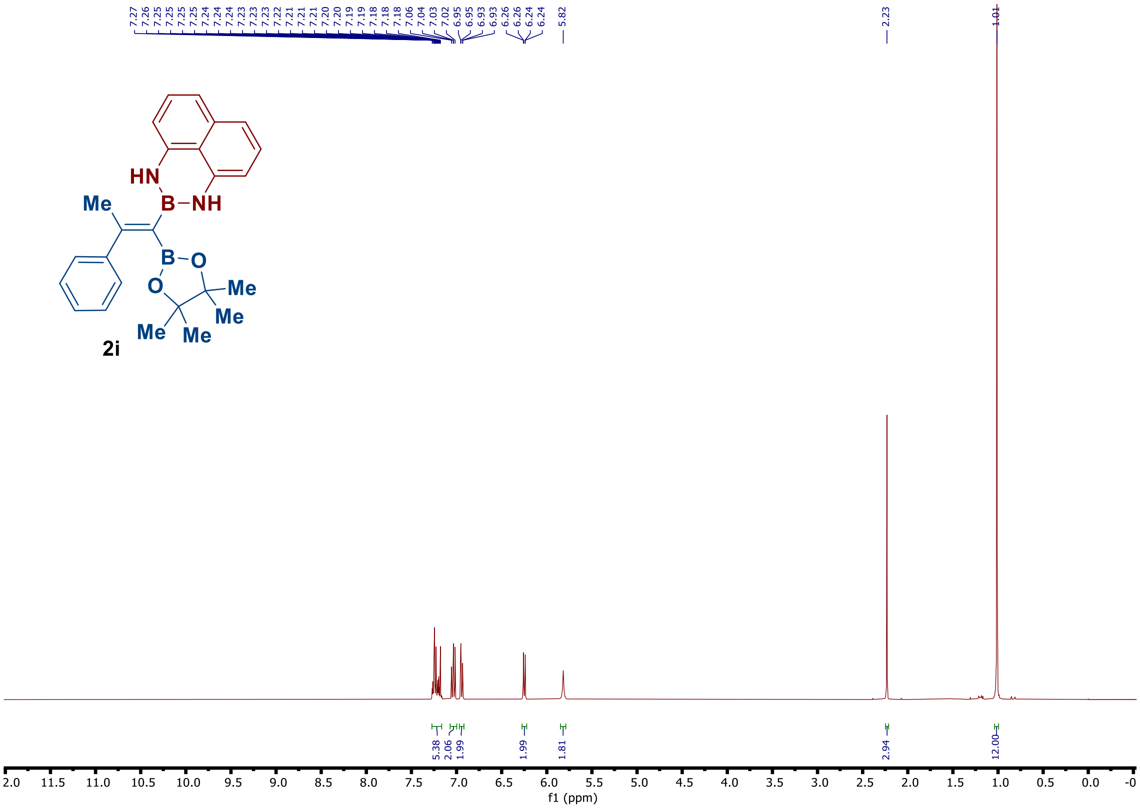

Supplementary Figure 140. ^{1}H NMR (400 MHz, $CDCl_3$) of compound (**2i**).

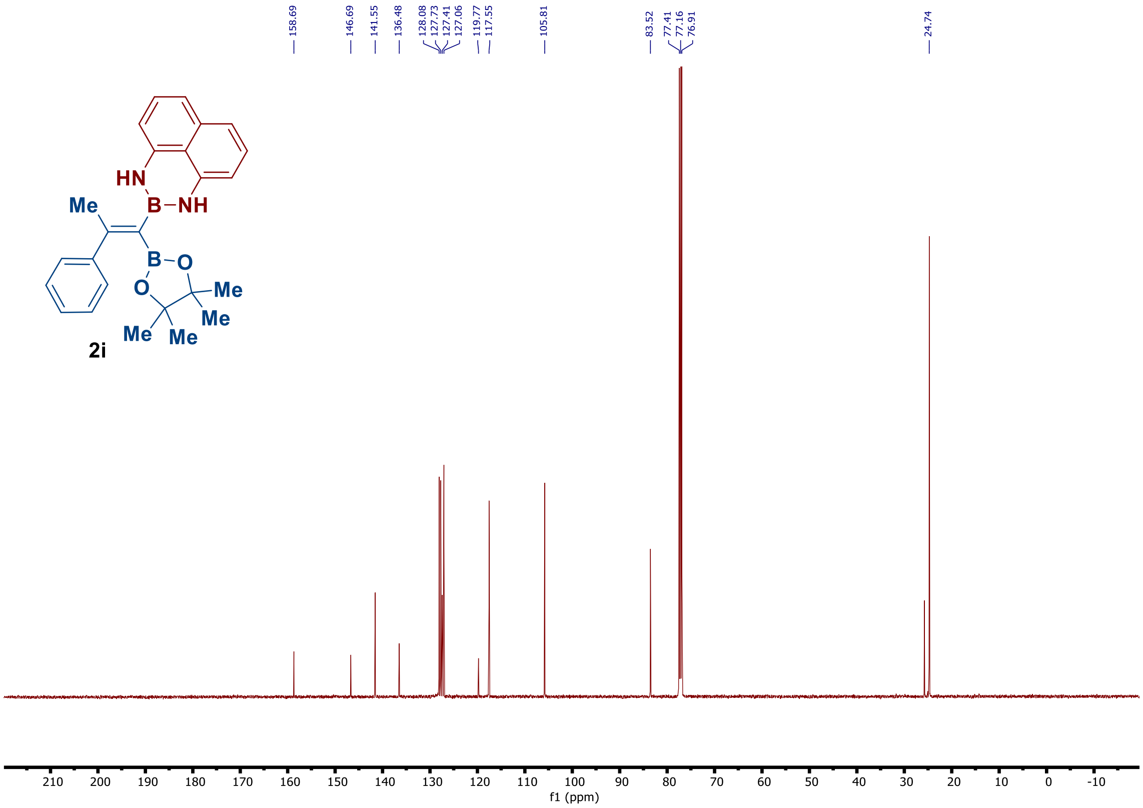

Supplementary Figure 141. ^{13}C NMR (101 MHz, $CDCl_3$) of compound (**2i**).

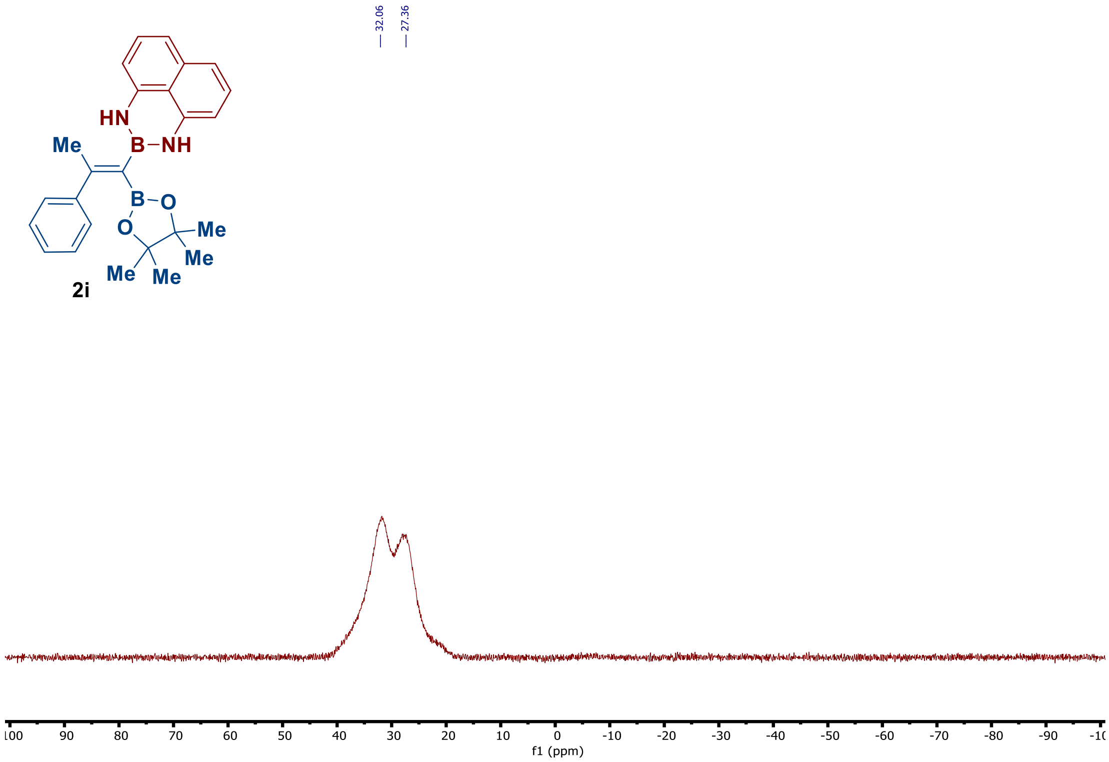

Supplementary Figure 142. ^{11}B NMR (128 MHz, $CDCl_3$) of compound (**2i**).

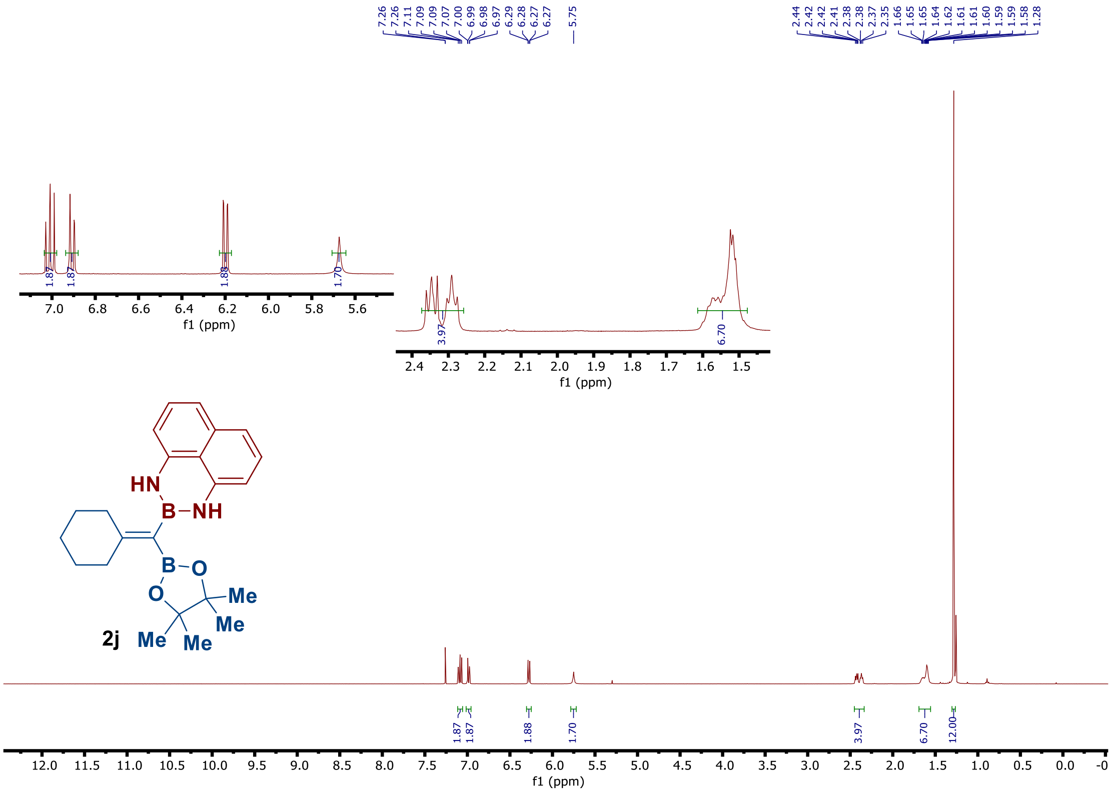

Supplementary Figure 143. ^{1}H NMR (400 MHz, $CDCl_3$) of compound (**2j**).

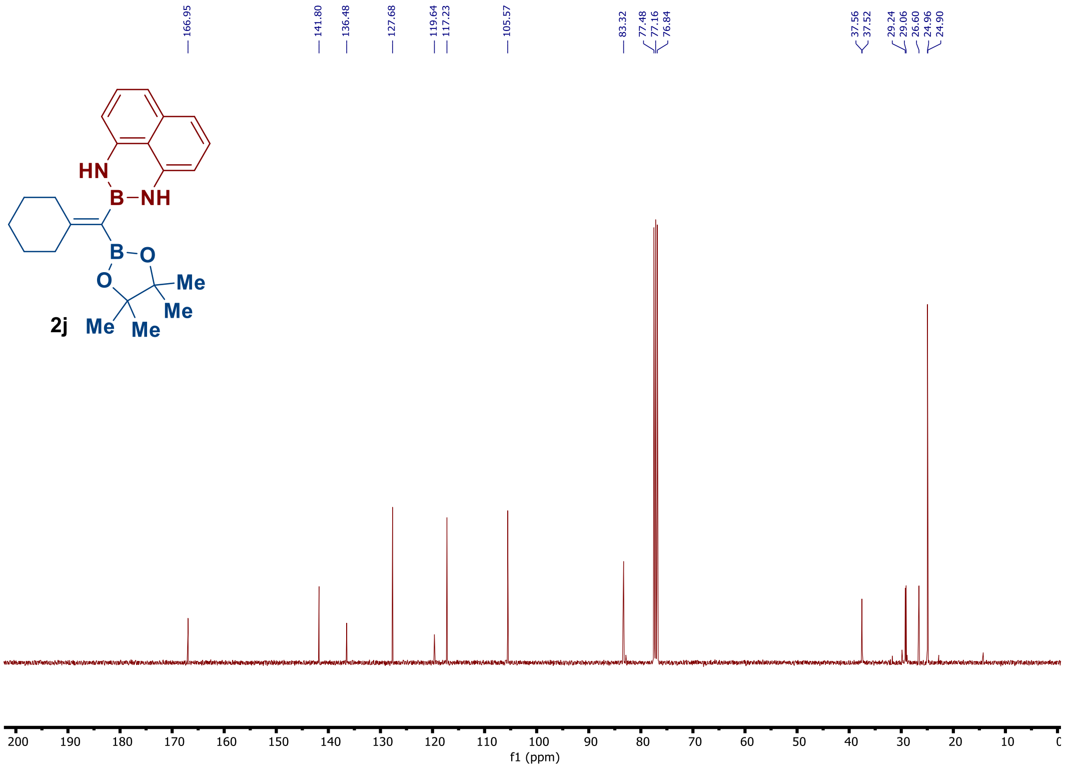

Supplementary Figure 144. ^{13}C NMR (101 MHz, $CDCl_3$) of compound (**2j**).

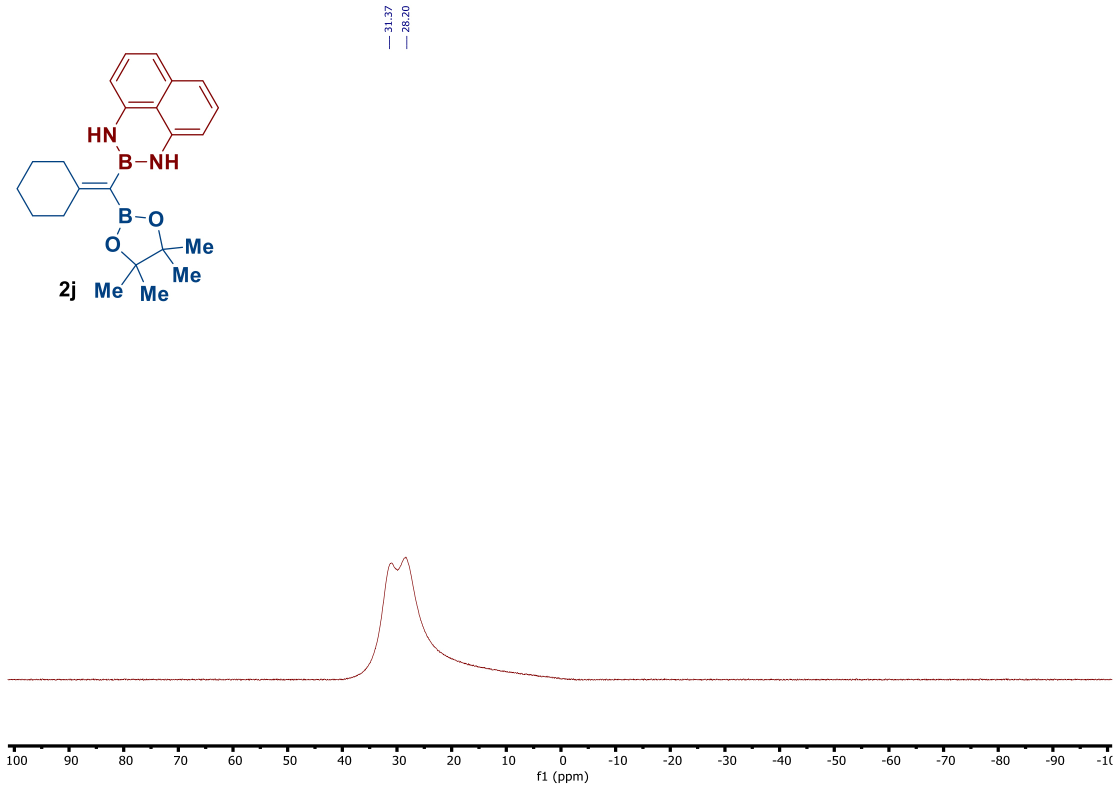

Supplementary Figure 145. ^{11}B NMR (128 MHz, $CDCl_3$) of compound (**2j**).

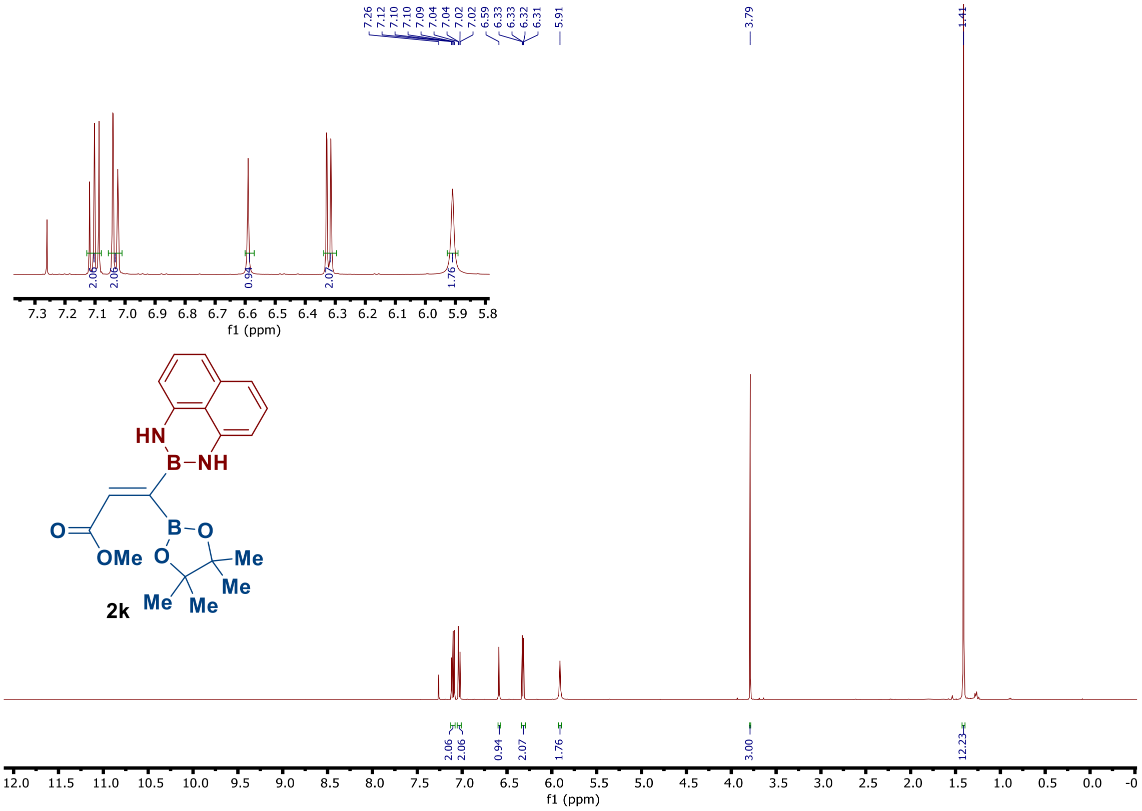

Supplementary Figure 146. ^{1}H NMR (400 MHz, $CDCl_3$) of compound (**2k**).

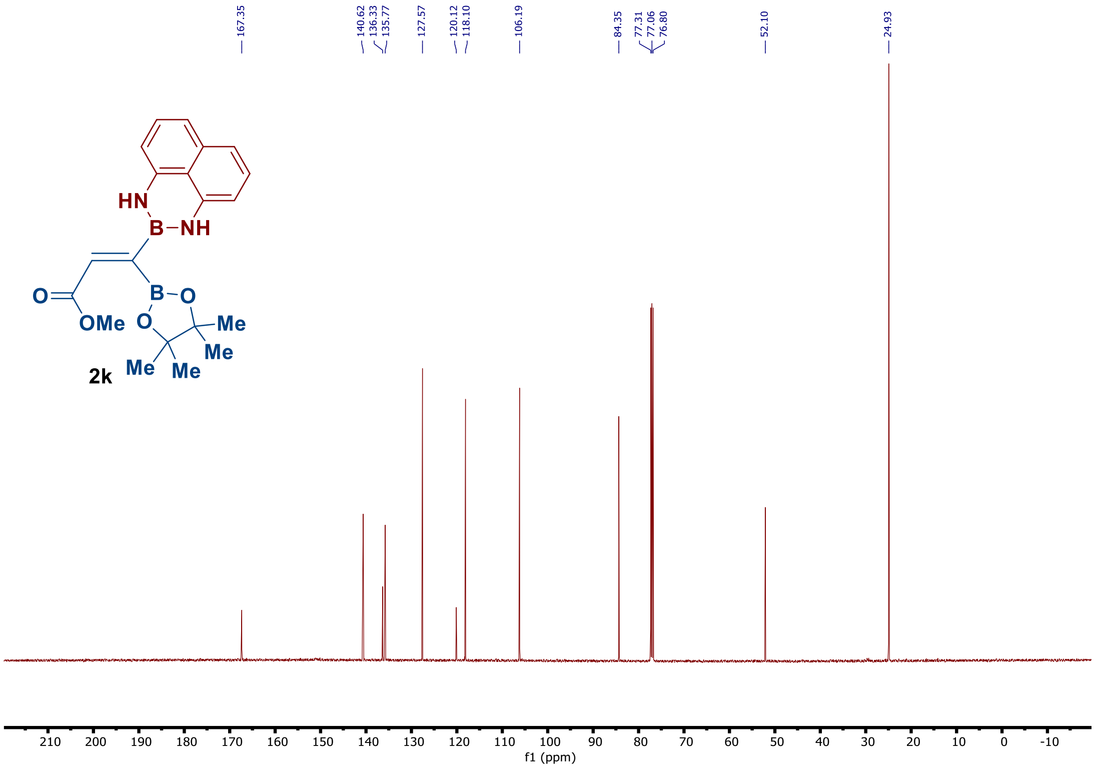

Supplementary Figure 147. ^{13}C NMR (101 MHz, $CDCl_3$) of compound (**2k**).

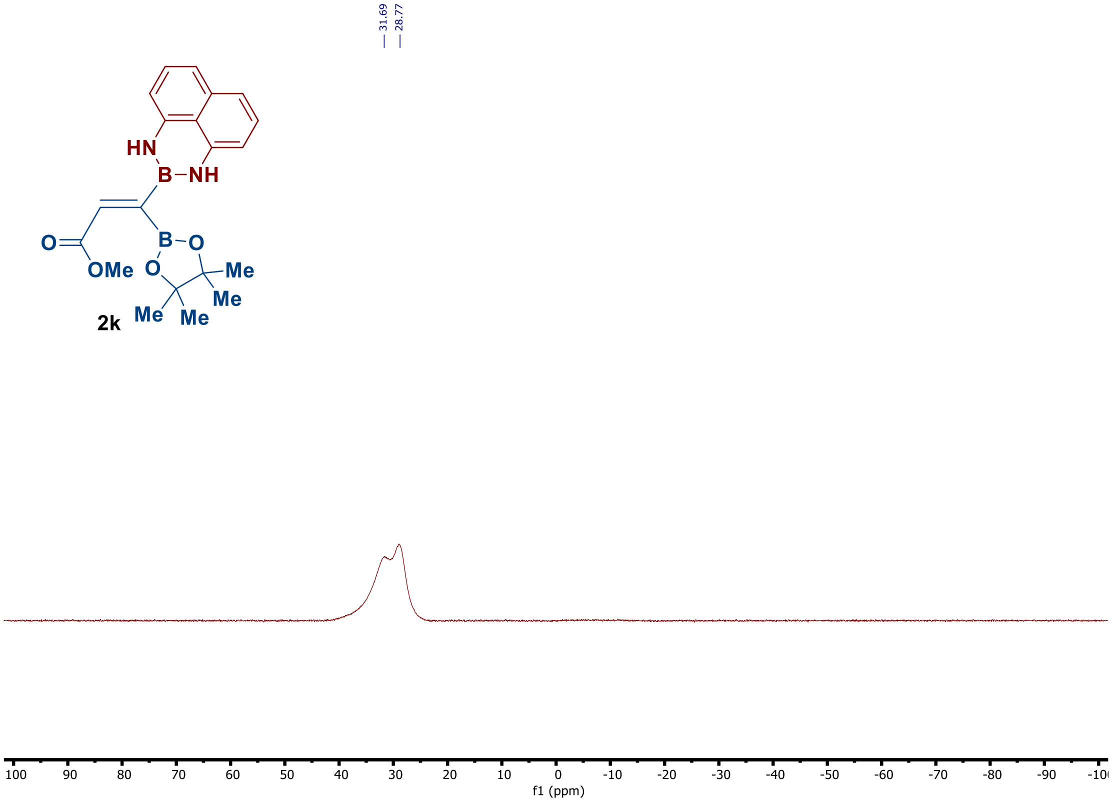

Supplementary Figure 148. ^{11}B NMR (128 MHz, $CDCl_3$) of compound (**2k**).

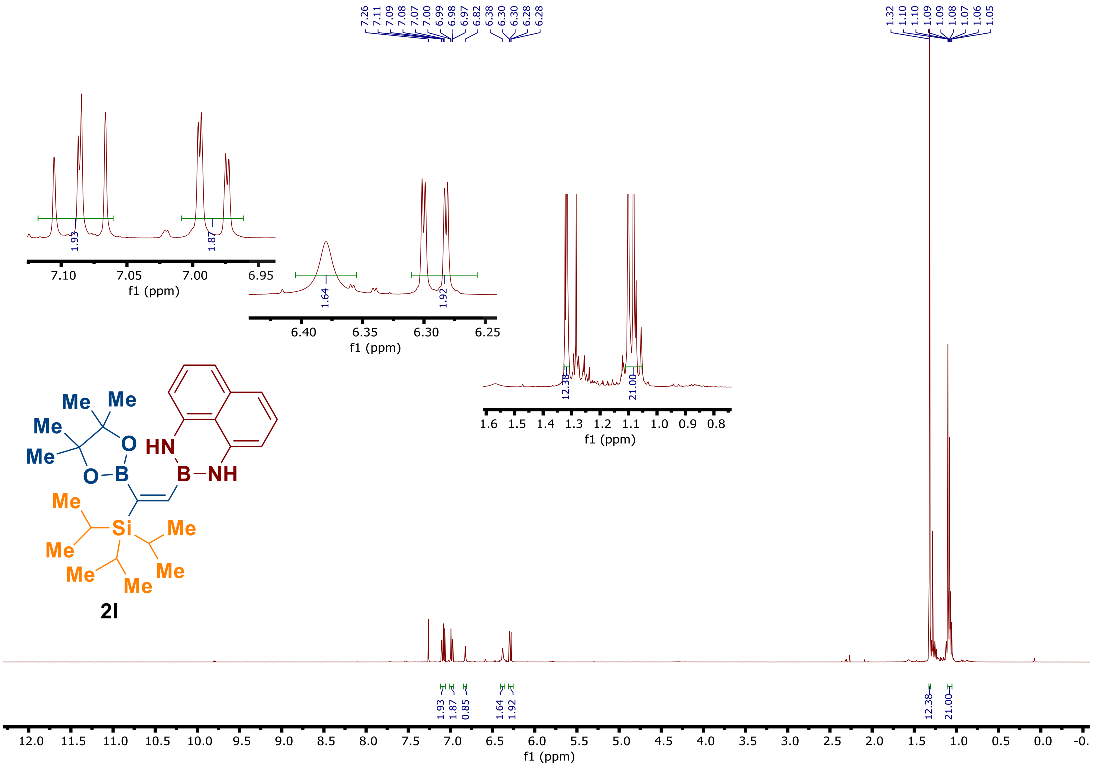

Supplementary Figure 149. ^{1}H NMR (400 MHz, $CDCl_3$) of compound (**2l**).

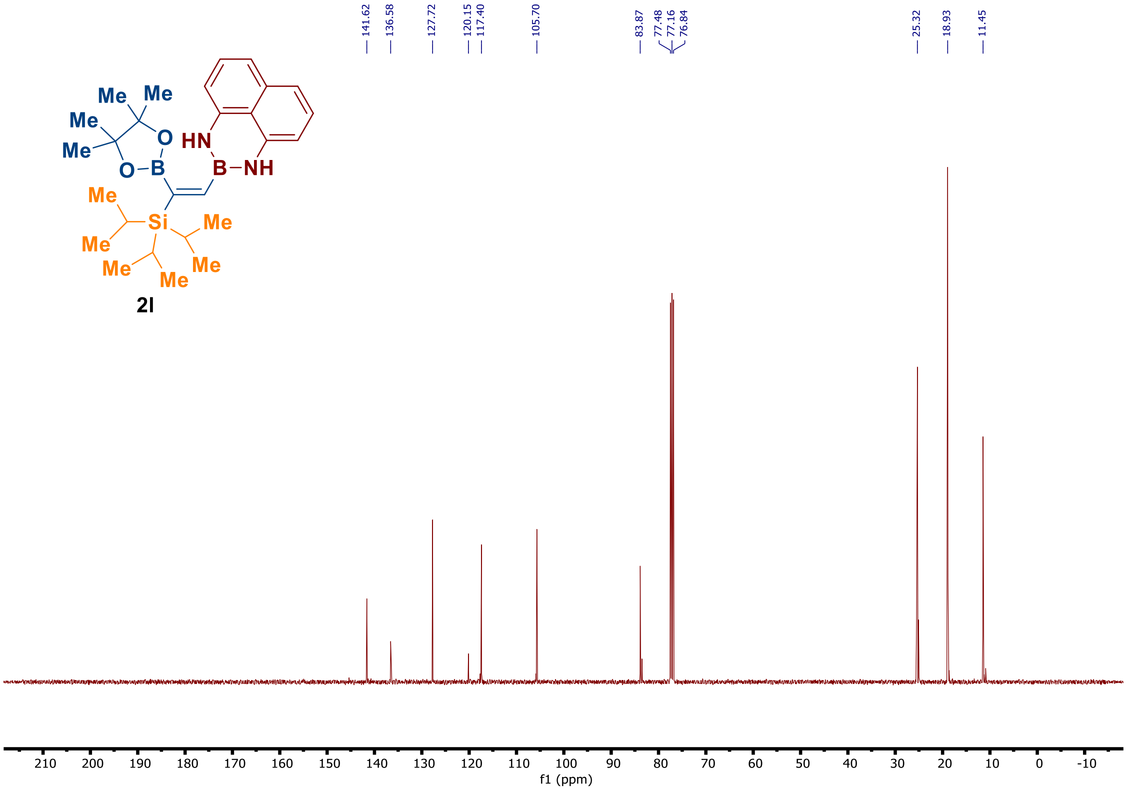

Supplementary Figure 150. ^{13}C NMR (101 MHz, $CDCl_3$) of compound (**2l**).

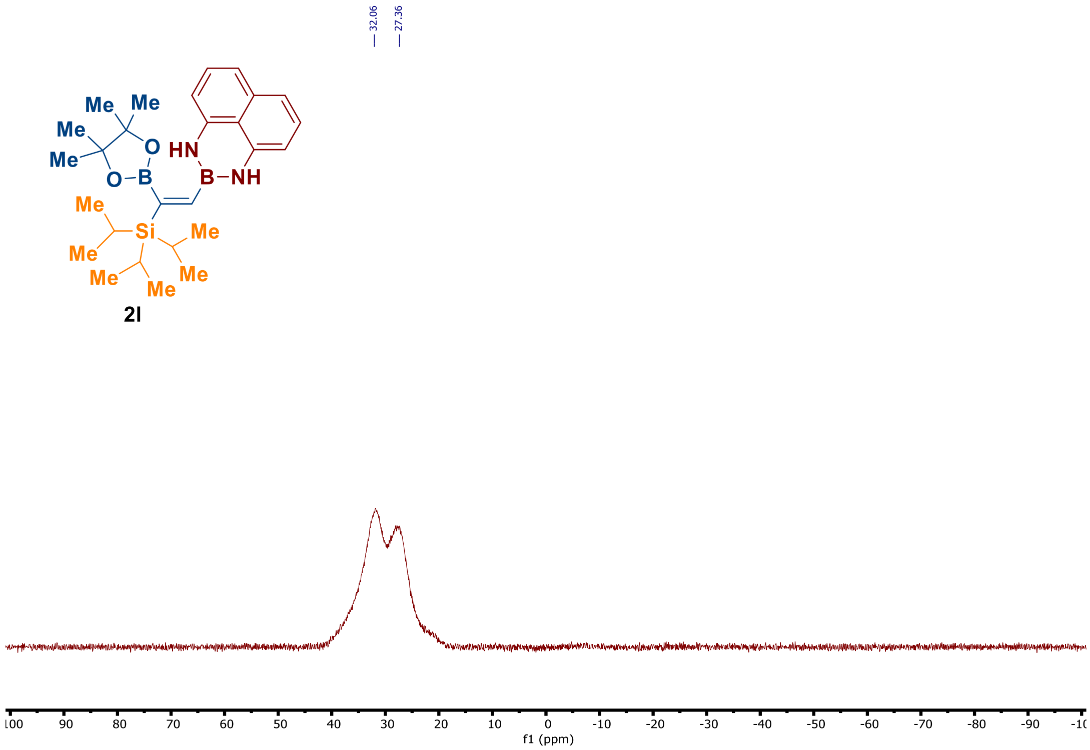

Supplementary Figure 151. ^{11}B NMR (128 MHz, $CDCl_3$) of compound (**2l**).

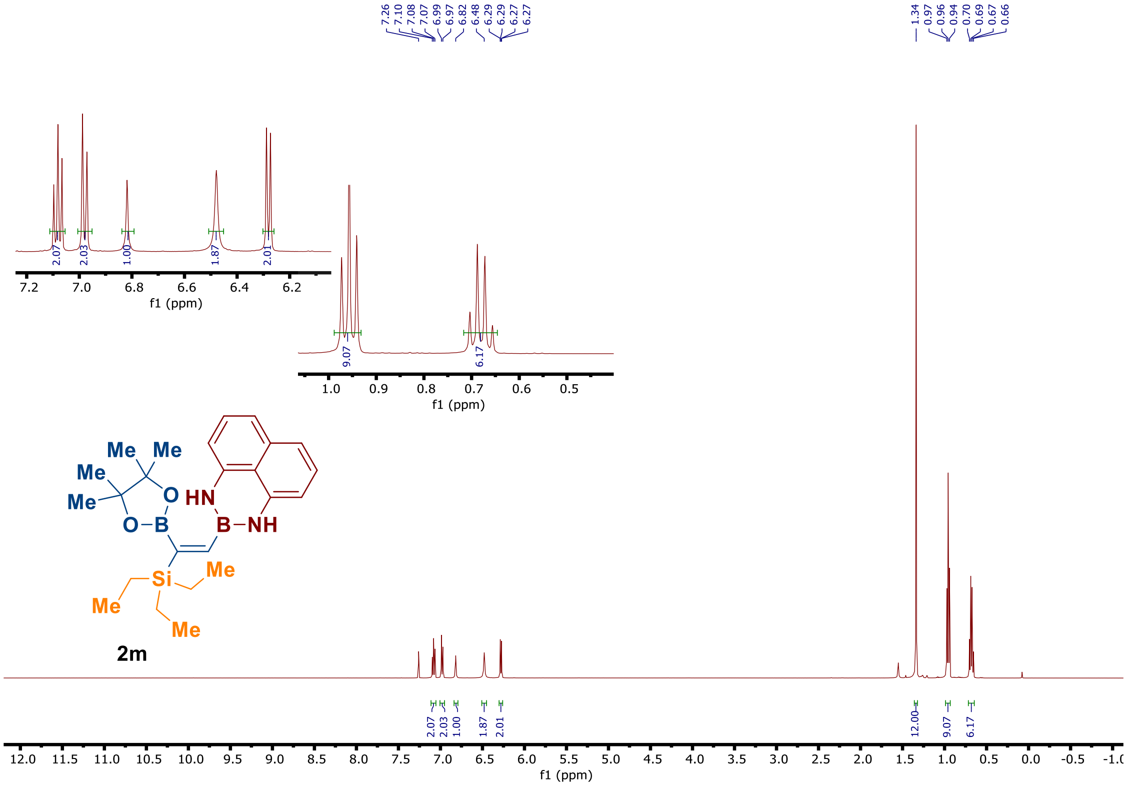

Supplementary Figure 152. ^{1}H NMR (400 MHz, $CDCl_3$) of compound (**2m**).

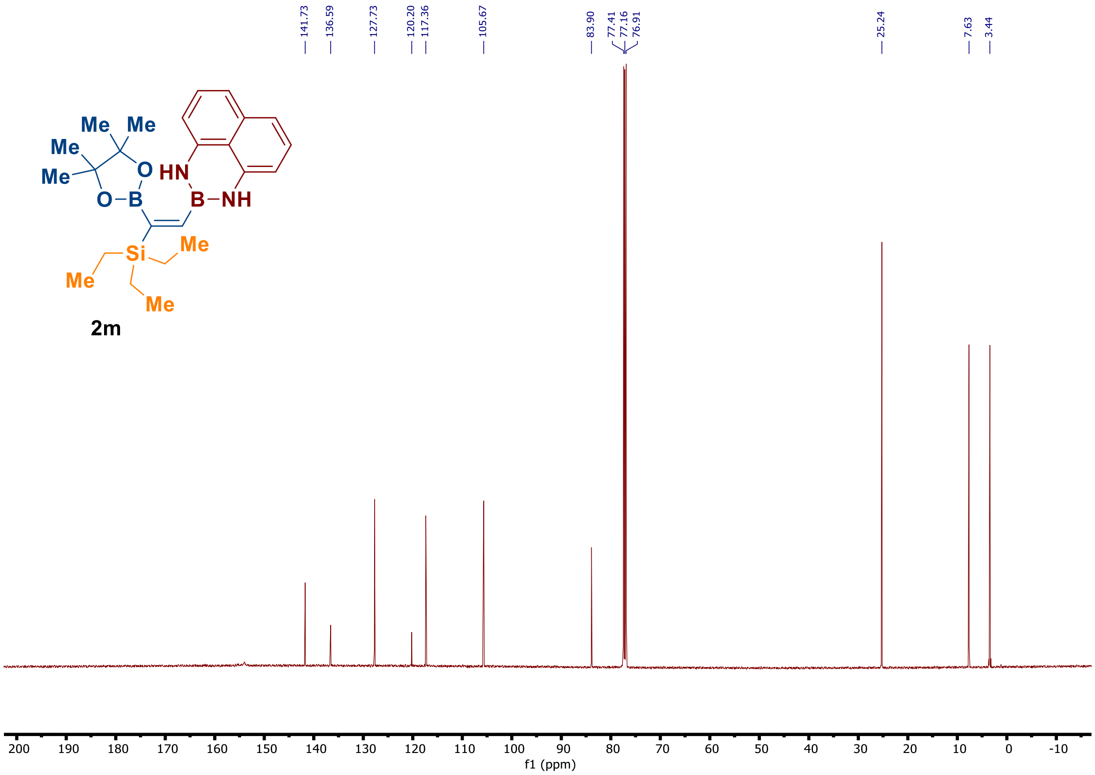

Supplementary Figure 153. ^{13}C NMR (101 MHz, $CDCl_3$) of compound (**2m**).

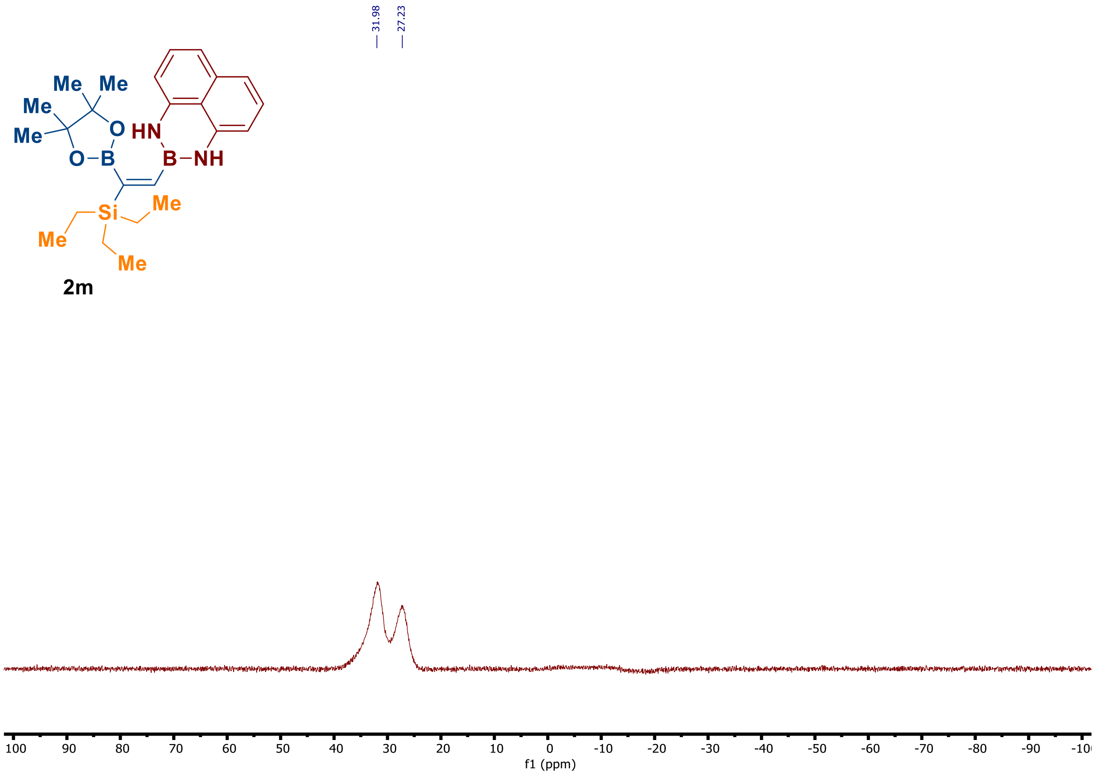

Supplementary Figure 154. ^{11}B NMR (128 MHz, $CDCl_3$) of compound (**2m**).

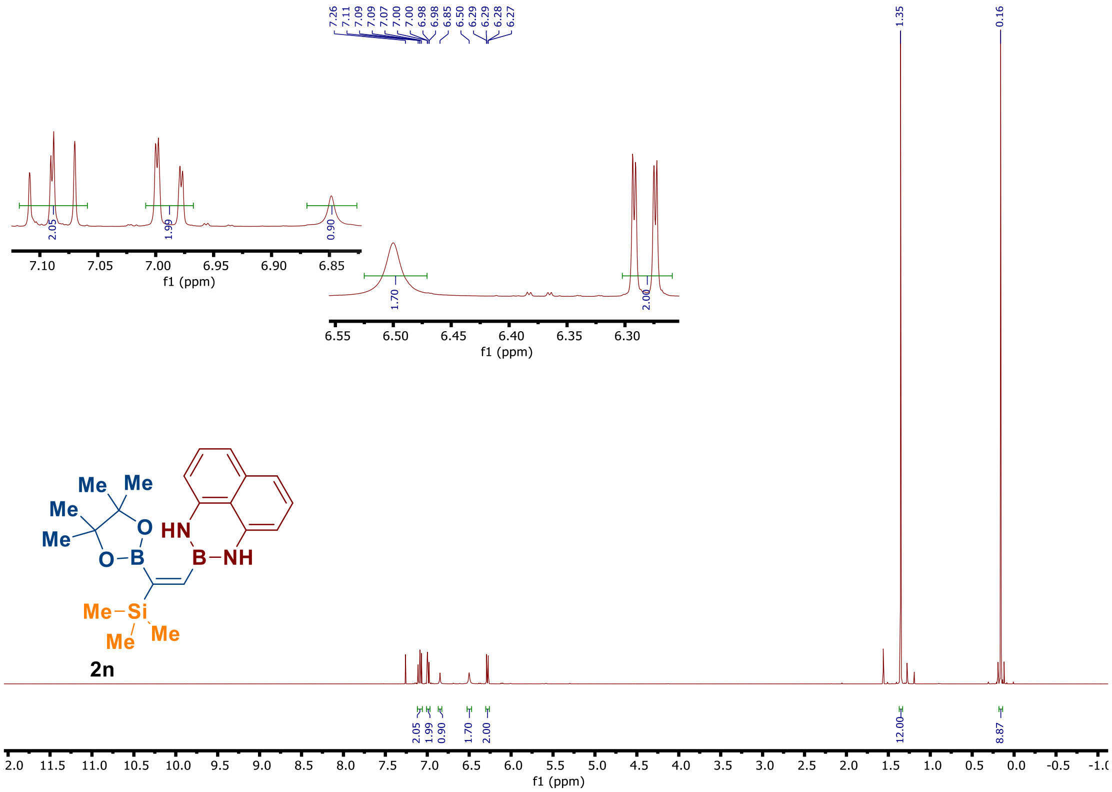

Supplementary Figure 155. ^{1}H NMR (400 MHz, $CDCl_3$) of compound (**2n**).

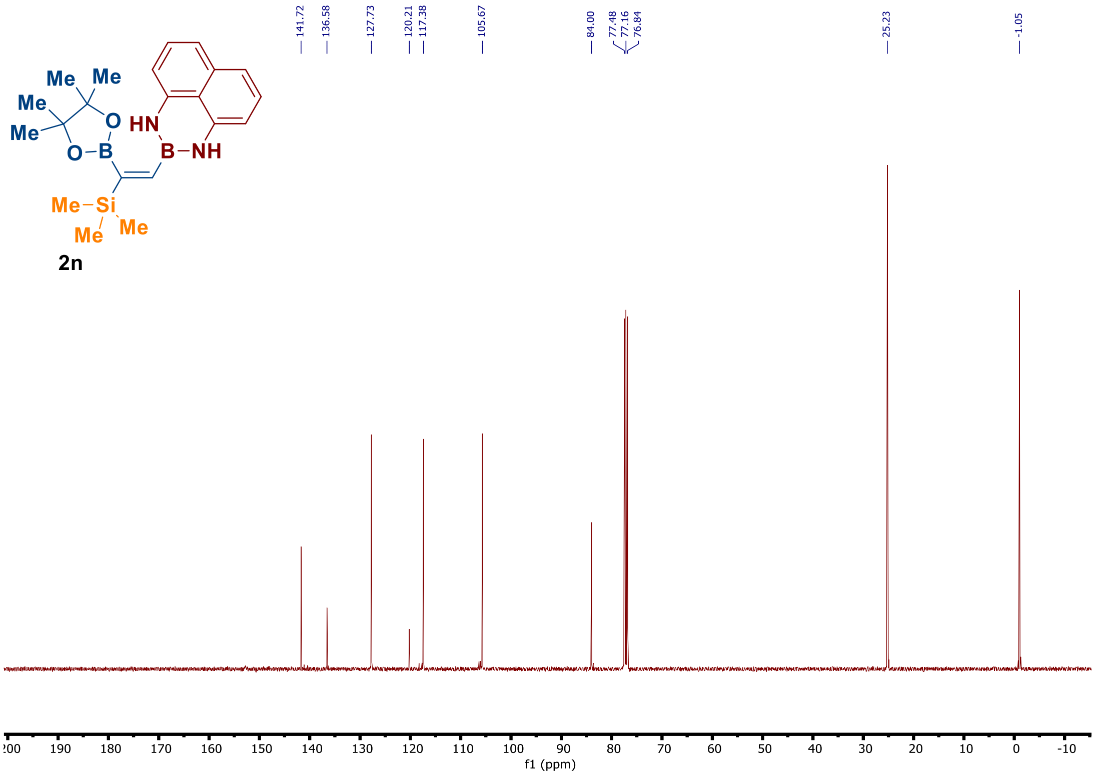

Supplementary Figure 156. ^{13}C NMR (101 MHz, $CDCl_3$) of compound (**2n**).

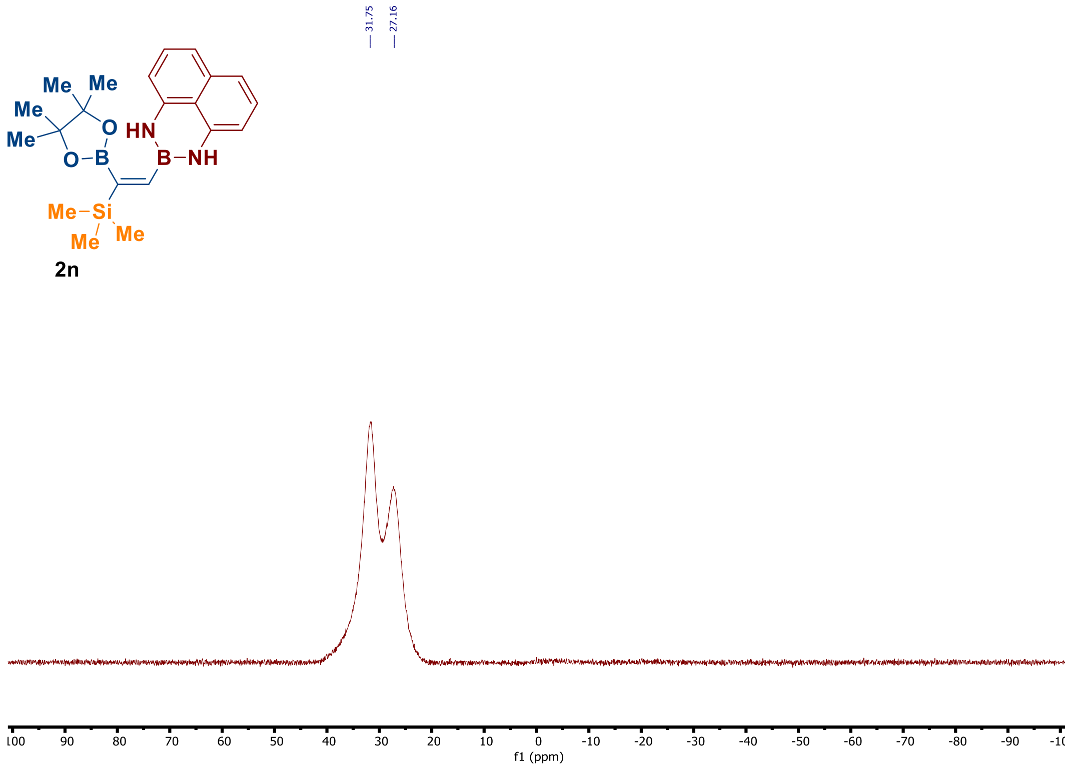

Supplementary Figure 157. ^{11}B NMR (128 MHz, $CDCl_3$) of compound (**2n**).

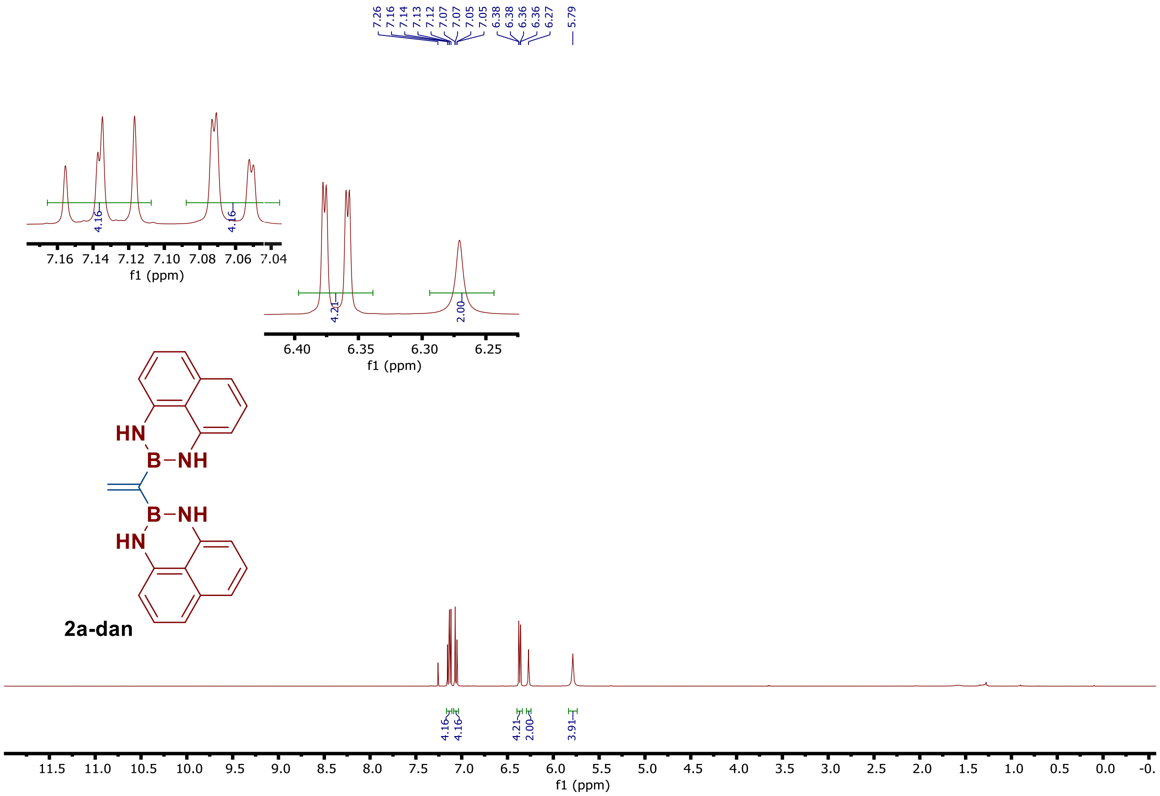

Supplementary Figure 158. ^{1}H NMR (400 MHz, $CDCl_3$) of compound (**2a-dan**).

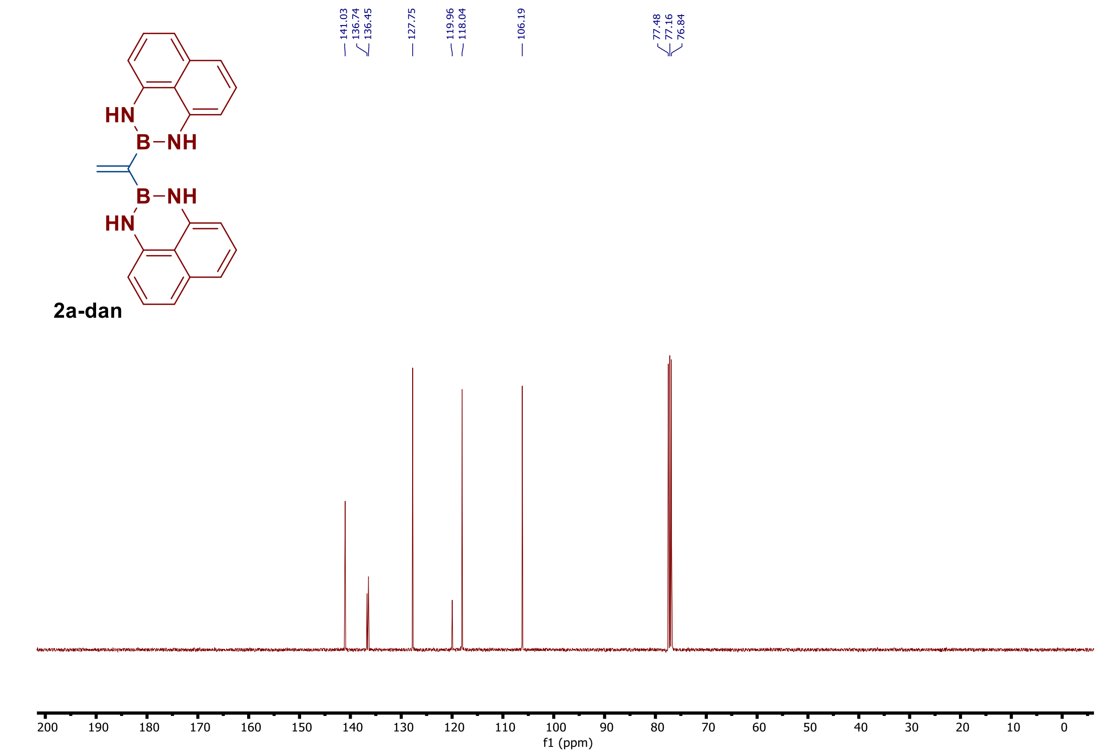

Supplementary Figure 159. ^{13}C NMR (101 MHz, $CDCl_3$) of compound (**2a-dan**).

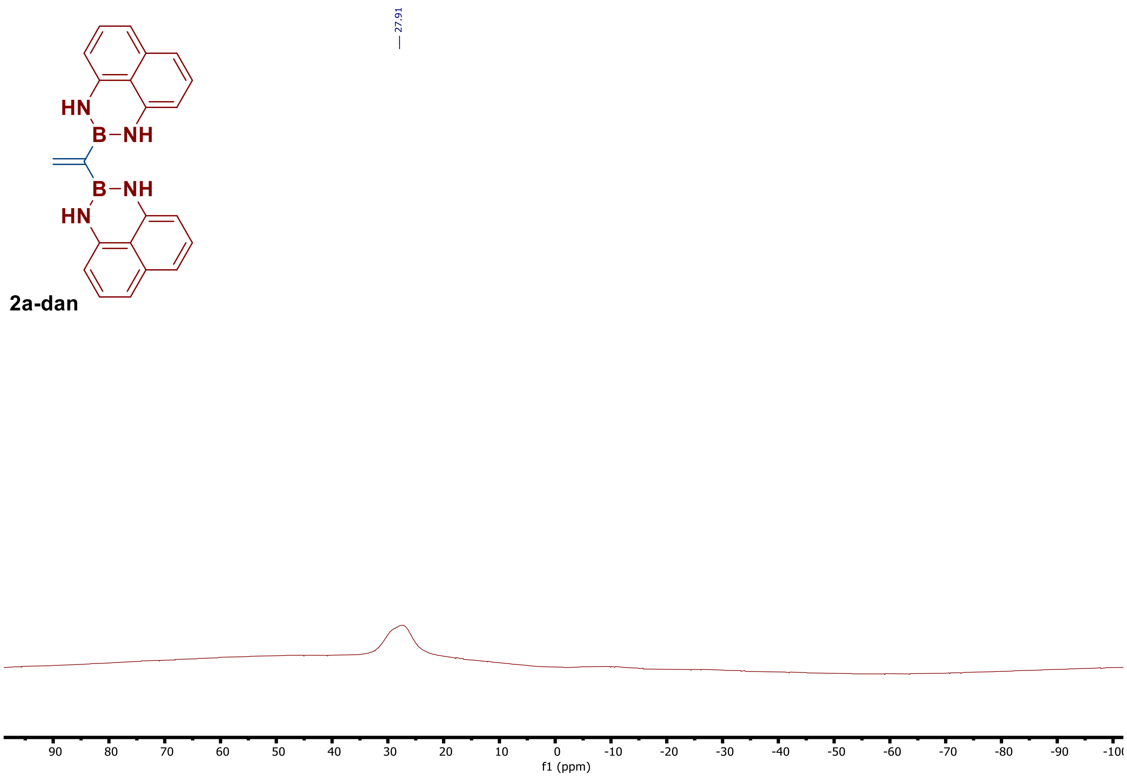

Supplementary Figure 160. ^{11}B NMR (128 MHz, $CDCl_3$) of compound (**2a-dan**).

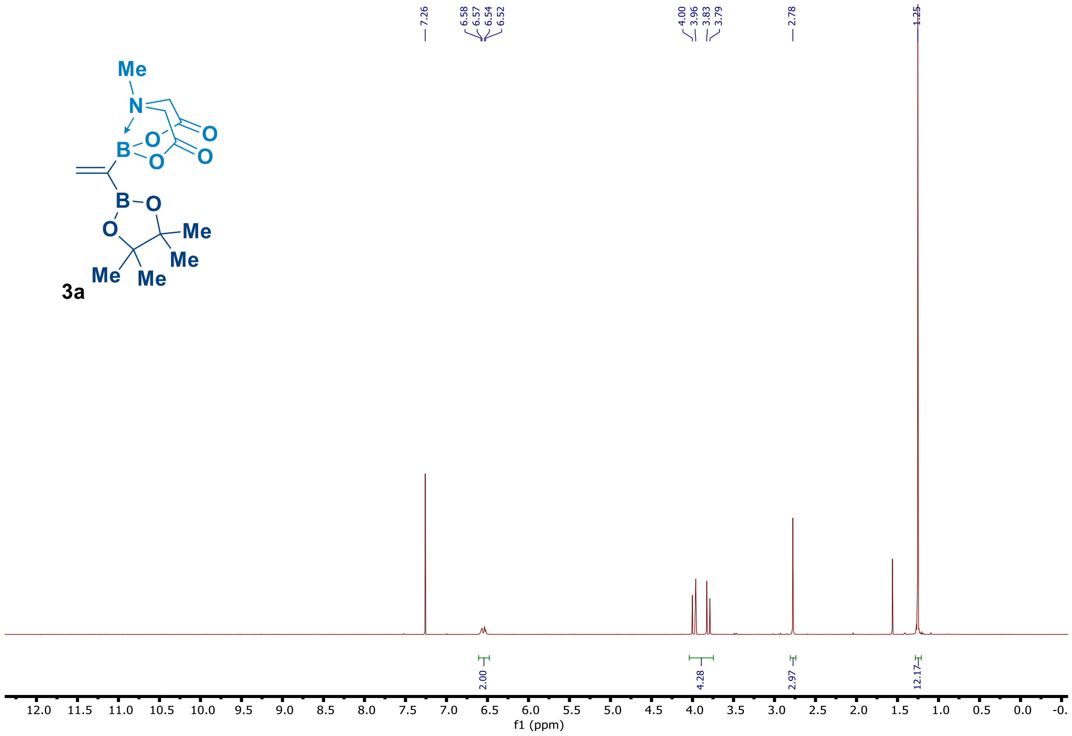

Supplementary Figure 161. ^{1}H NMR (400 MHz, $CDCl_3$) of compound (**3a**).

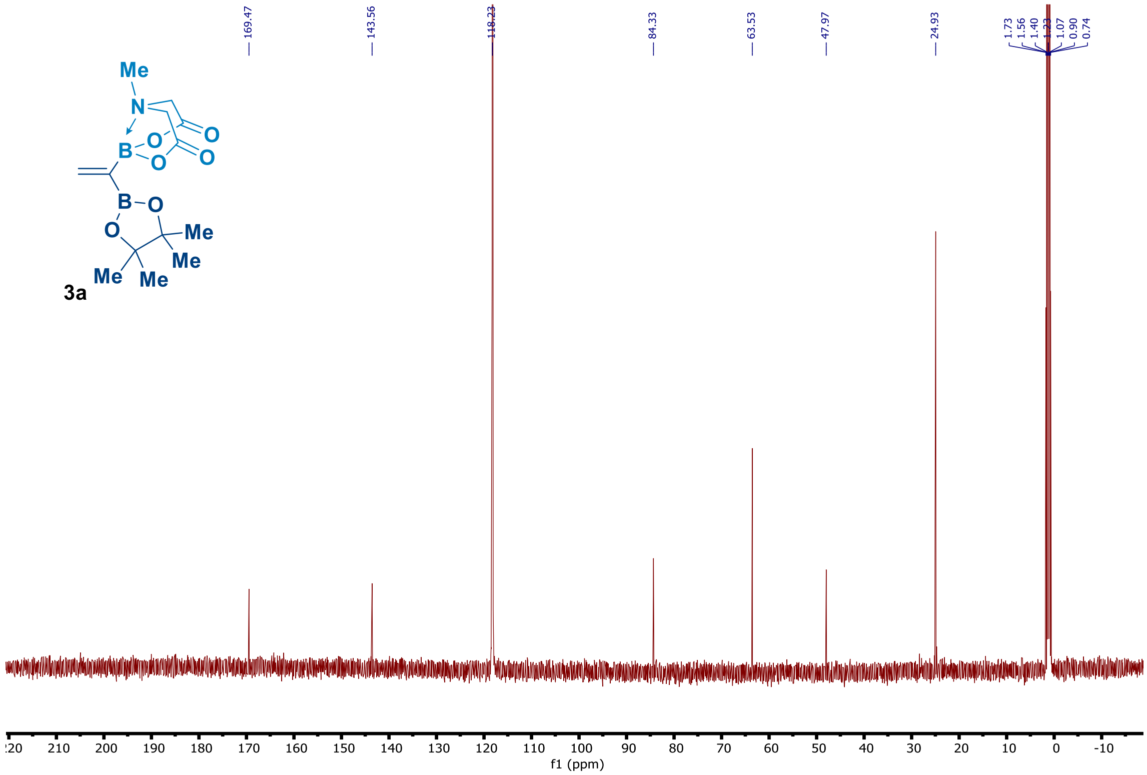

Supplementary Figure 162. ^{13}C NMR (101 MHz, CD_3CN) of compound (**3a**).

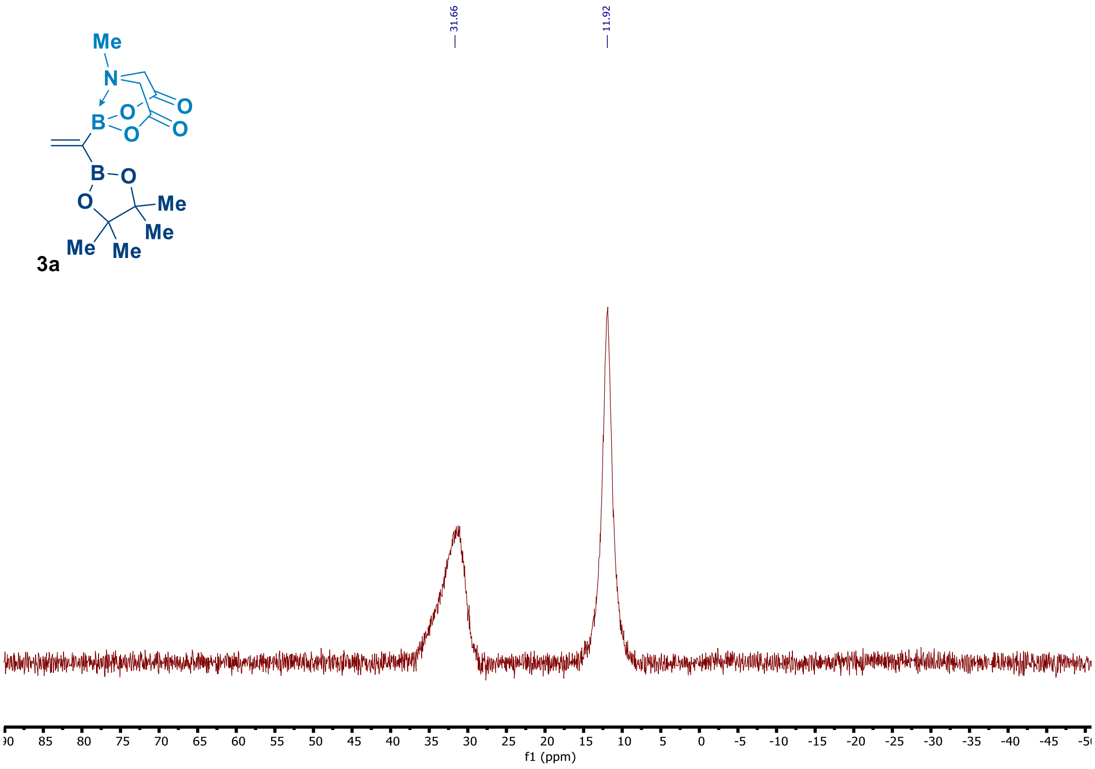

Supplementary Figure 163. ^{11}B NMR (128 MHz, $CDCl_3$) of compound (**3a**).

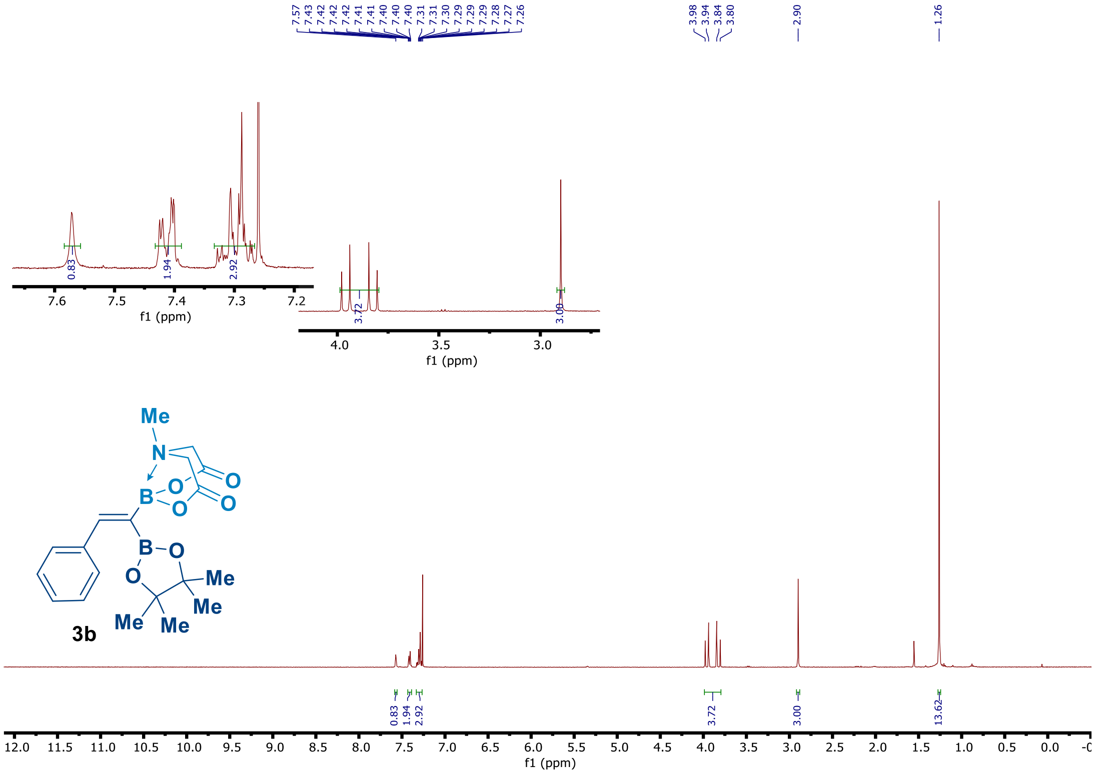

Supplementary Figure 164. ^{1}H NMR (400 MHz, $CDCl_3$) of compound (**3b**).

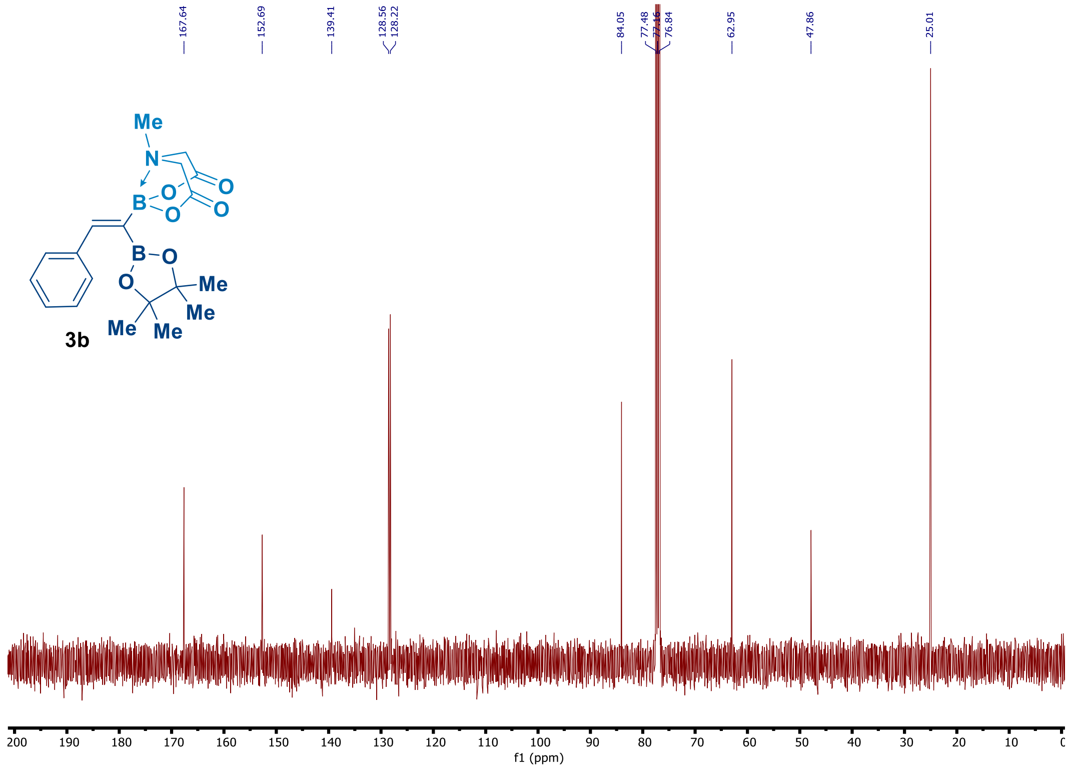

Supplementary Figure 165. ^{13}C NMR (101 MHz, $CDCl_3$) of compound (**3b**).

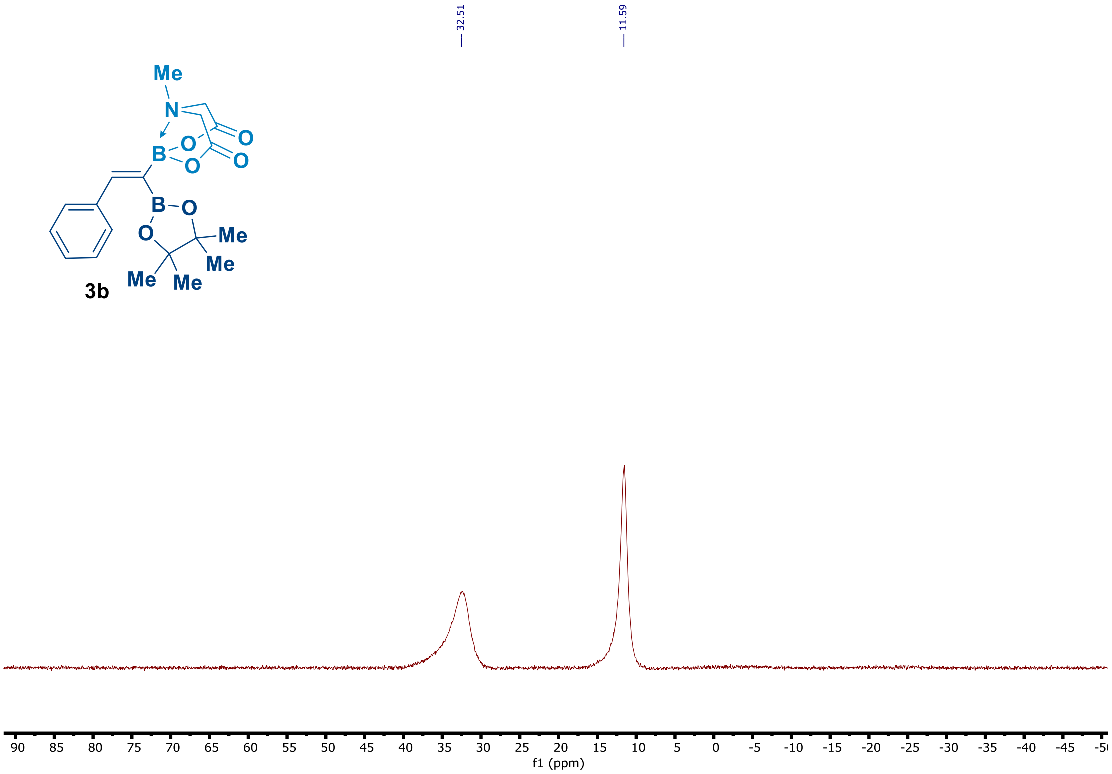

Supplementary Figure 166. ^{11}B NMR (128 MHz, $CDCl_3$) of compound (**3b**).

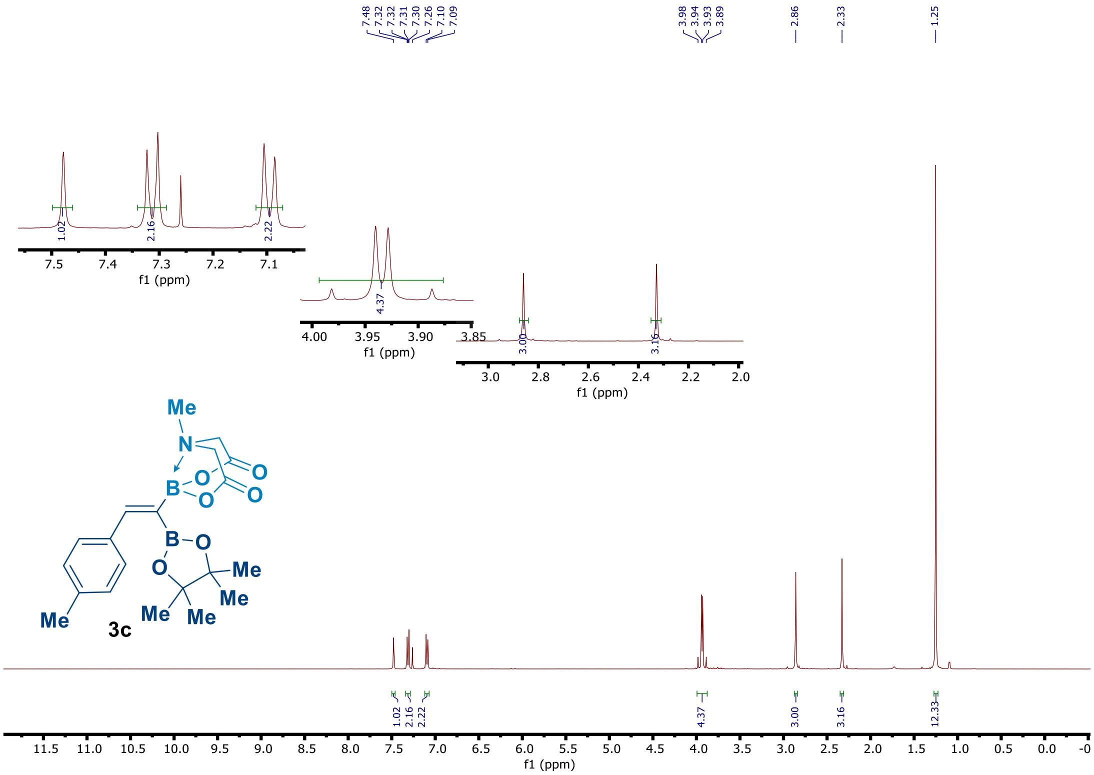

Supplementary Figure 167. ^{1}H NMR (400 MHz, $CDCl_3$) of compound (**3c**).

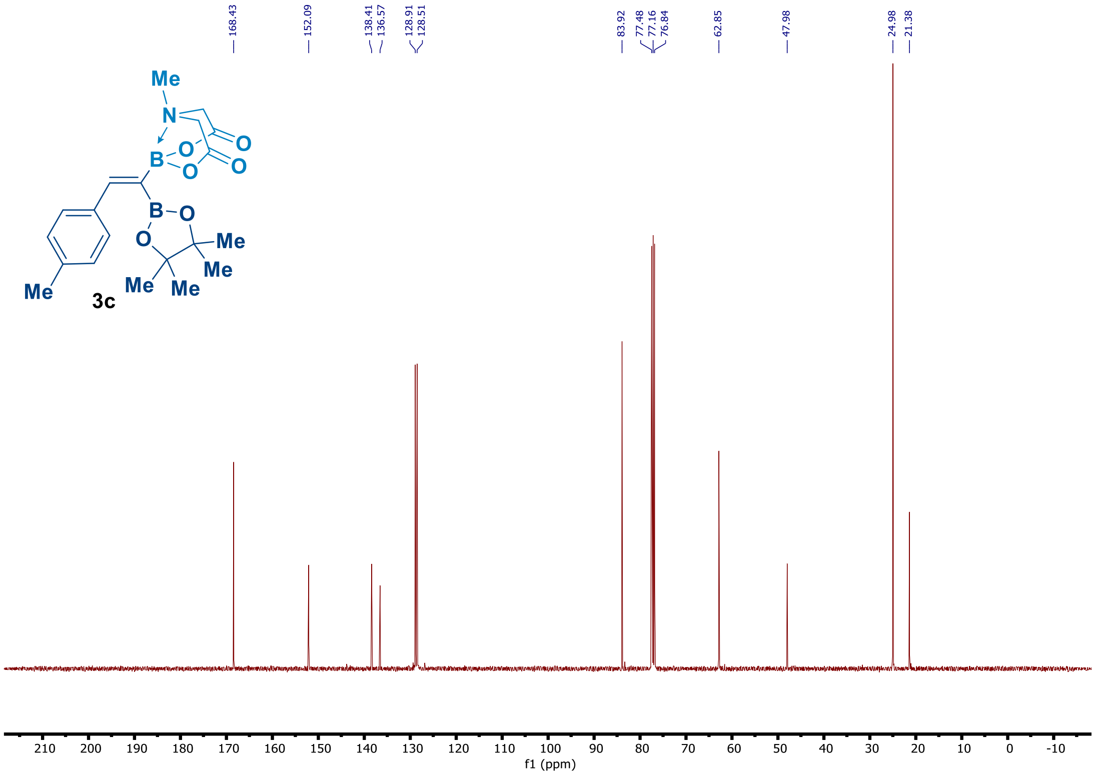

Supplementary Figure 168. ^{13}C NMR (101 MHz, $CDCl_3$) of compound (**3c**).

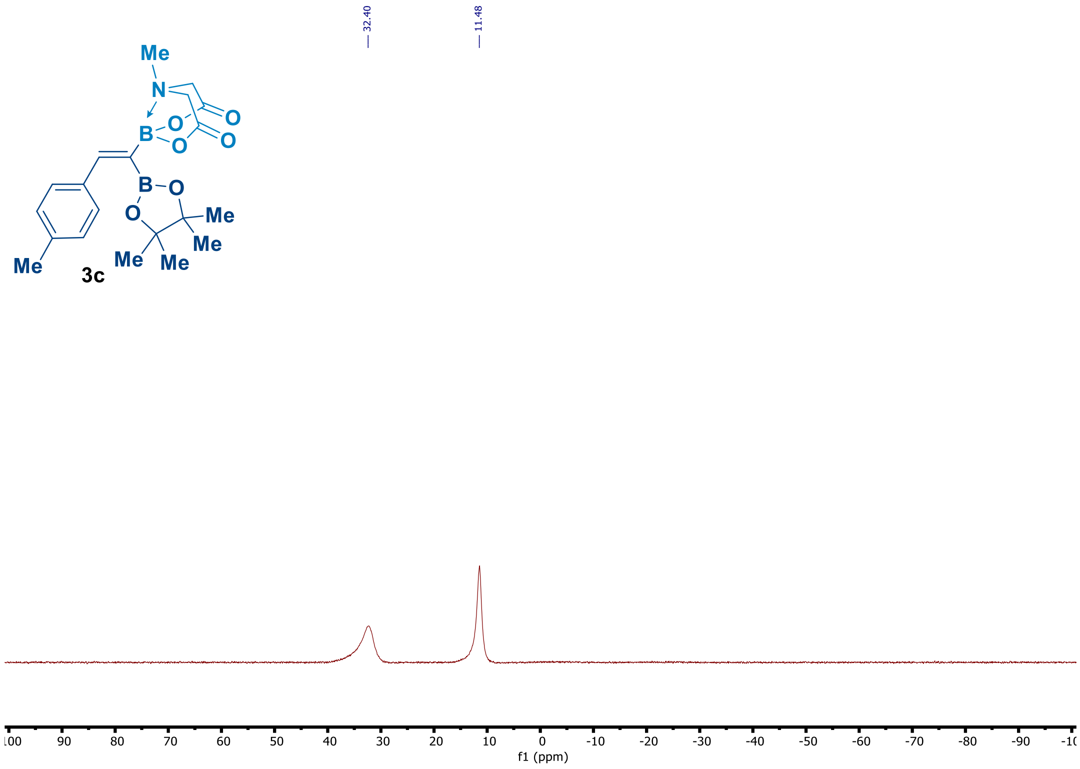

Supplementary Figure 169. ^{11}B NMR (128 MHz, $CDCl_3$) of compound (**3c**).

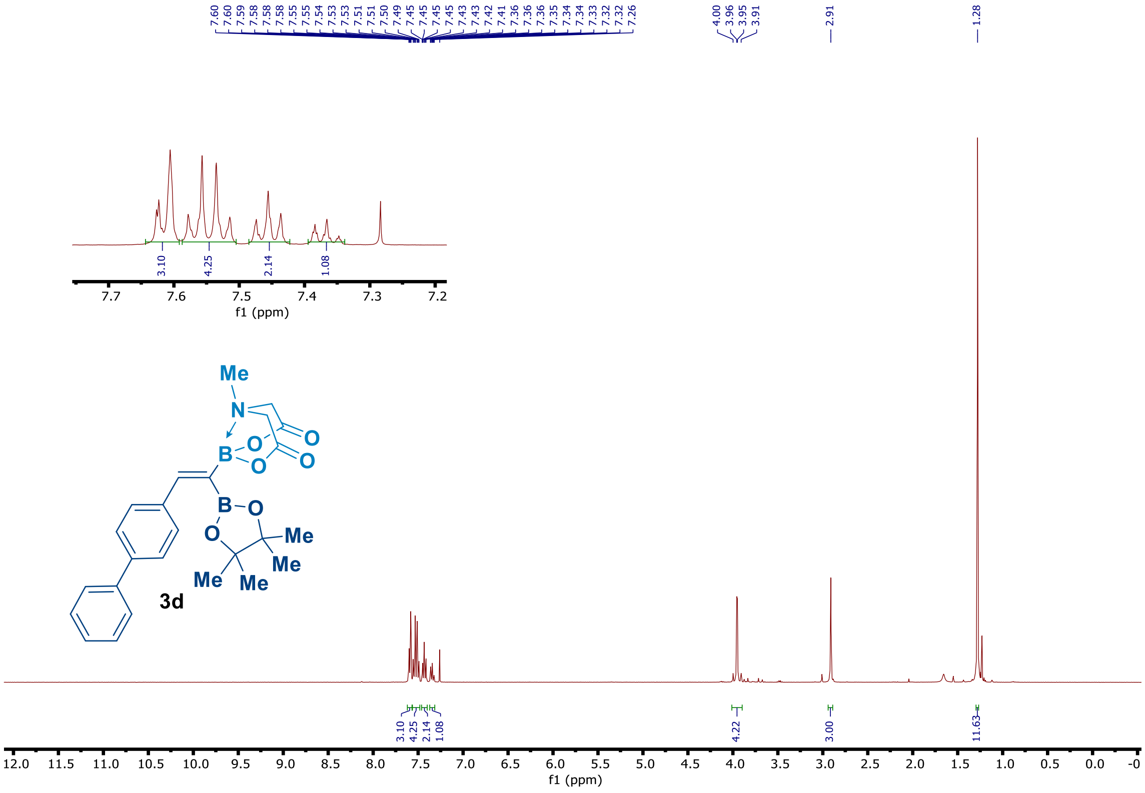

Supplementary Figure 170. ^{1}H NMR (400 MHz, $CDCl_3$) of compound (**3d**).

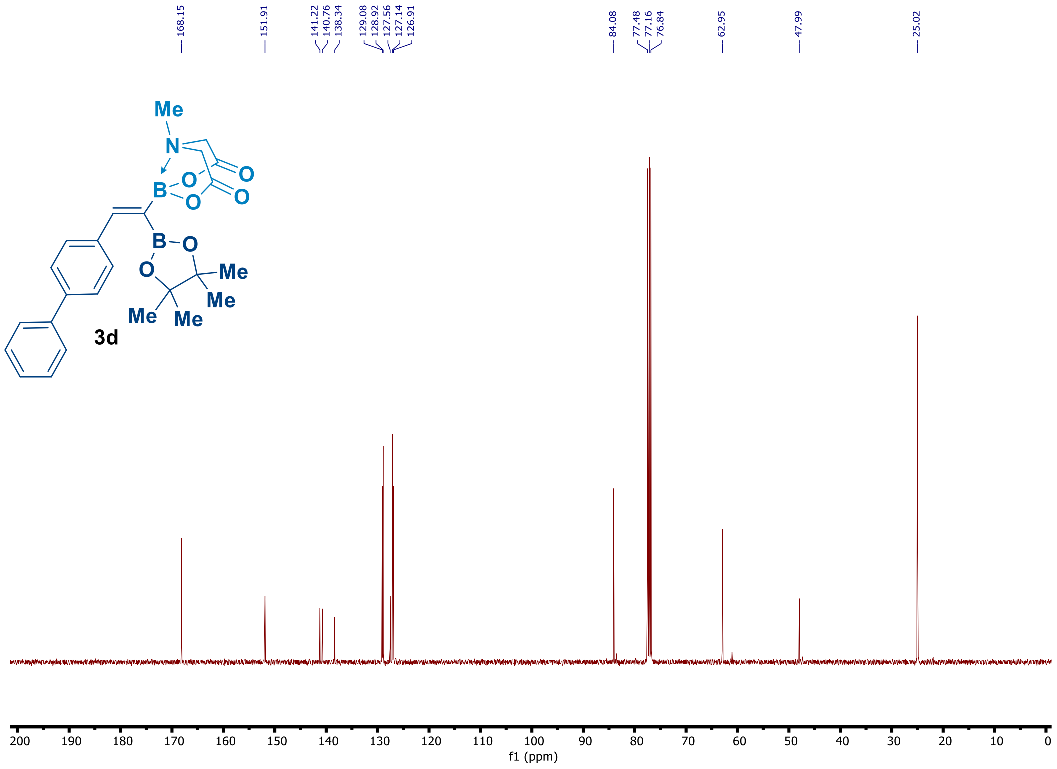

Supplementary Figure 171. ^{13}C NMR (101 MHz, $CDCl_3$) of compound (**3d**).

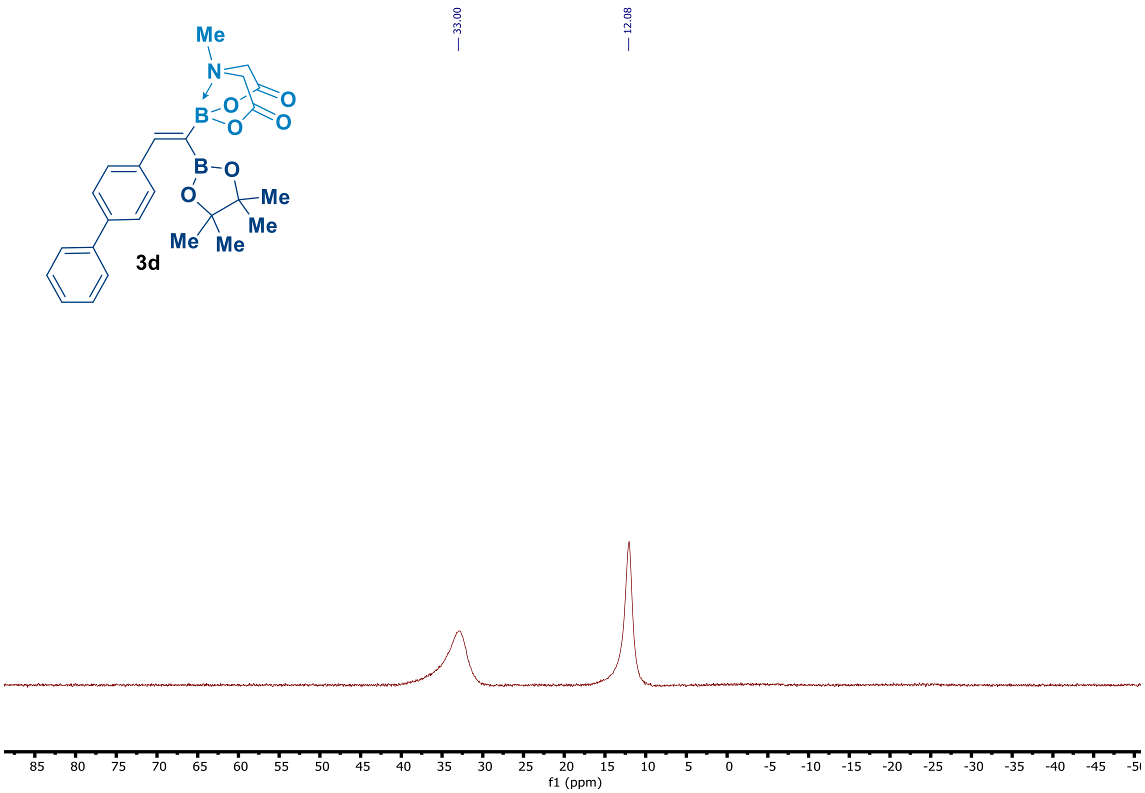

Supplementary Figure 172. ^{11}B NMR (128 MHz, $CDCl_3$) of compound (**3d**).

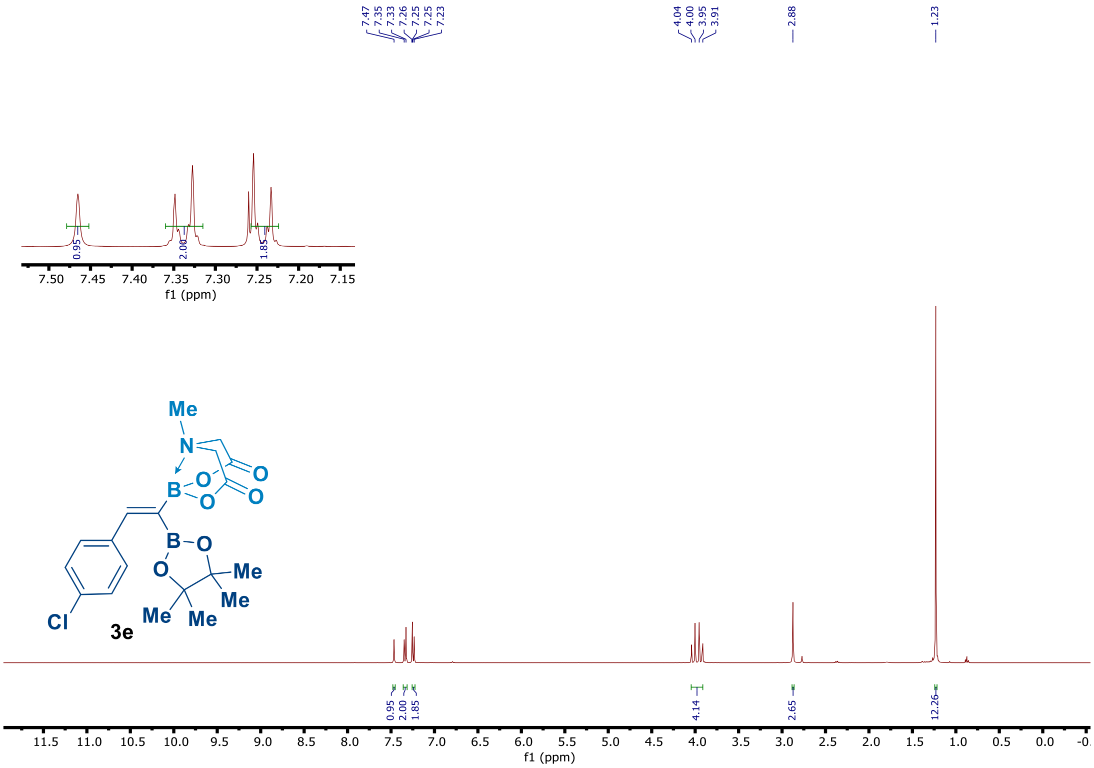

Supplementary Figure 173. ^{1}H NMR (400 MHz, $CDCl_3$) of compound (**3e**).

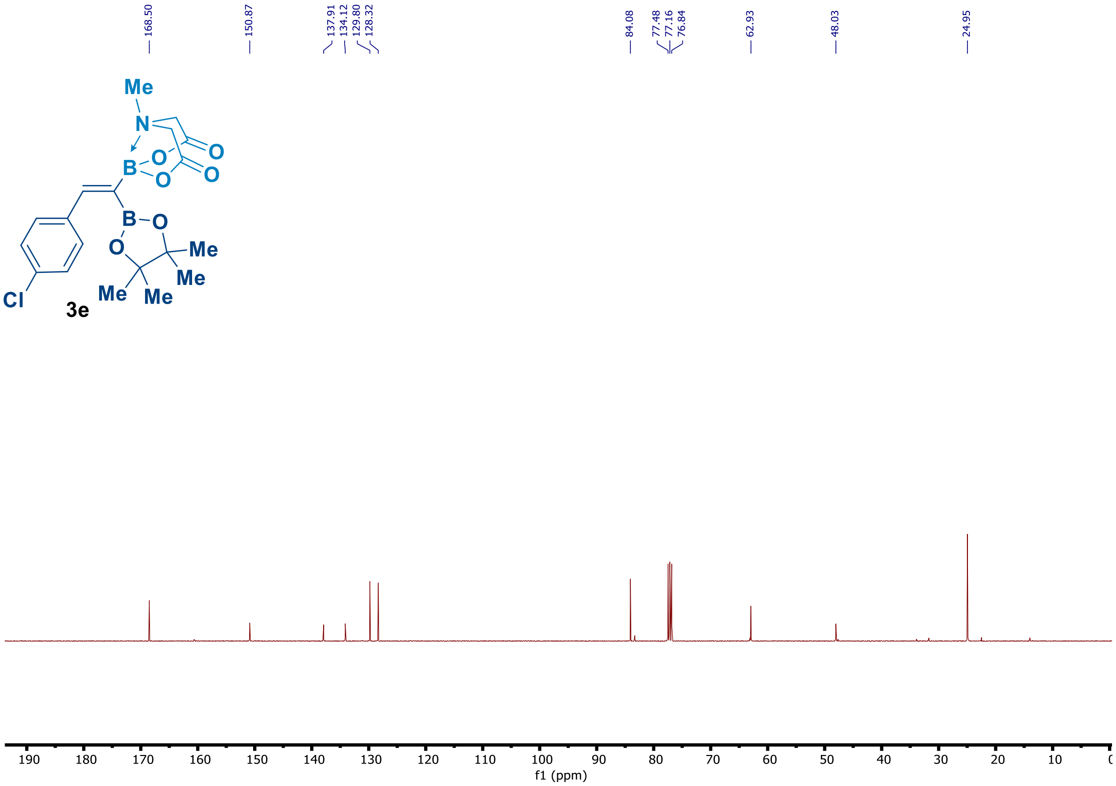

Supplementary Figure 174. ^{13}C NMR (101 MHz, $CDCl_3$) of compound (**3e**).

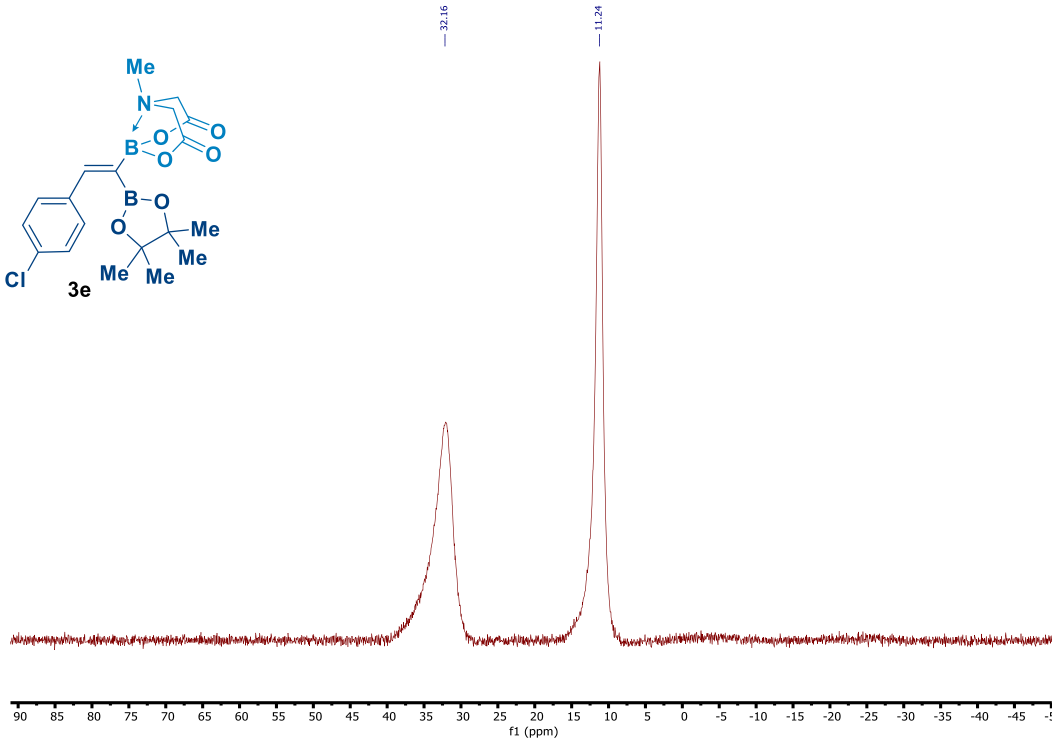

Supplementary Figure 175. ^{11}B NMR (128 MHz, $CDCl_3$) of compound (**3e**).

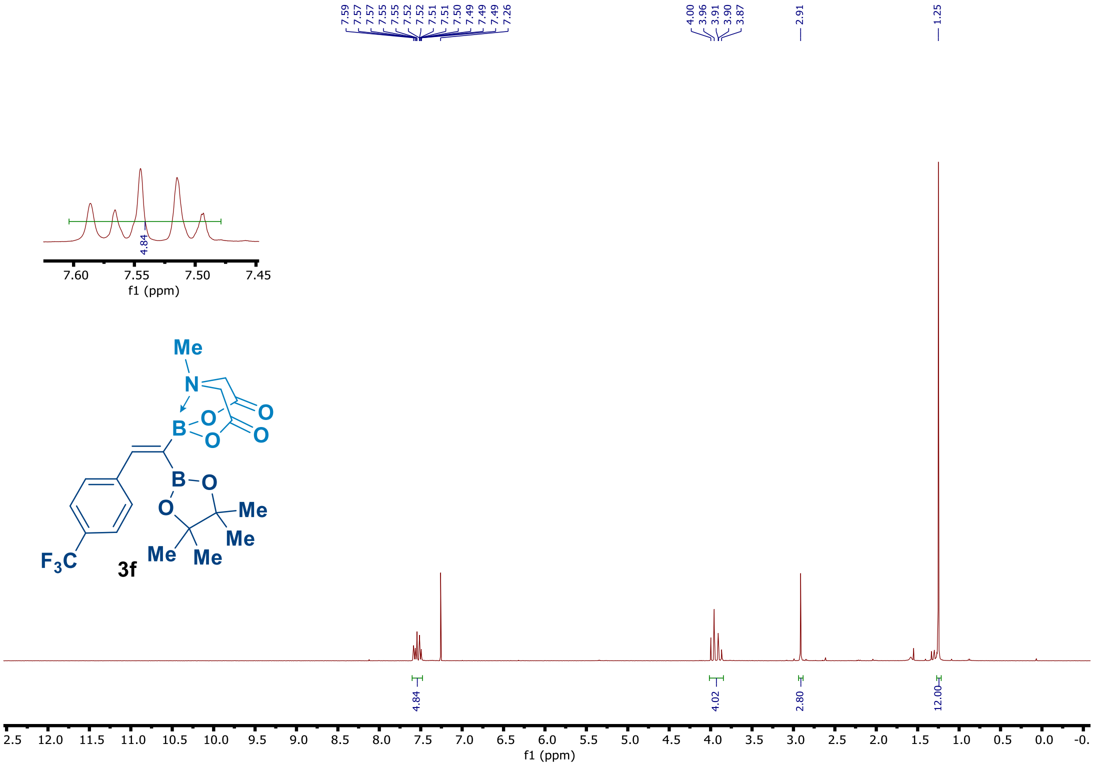

Supplementary Figure 176. ^{1}H NMR (400 MHz, $CDCl_3$) of compound (**3f**).

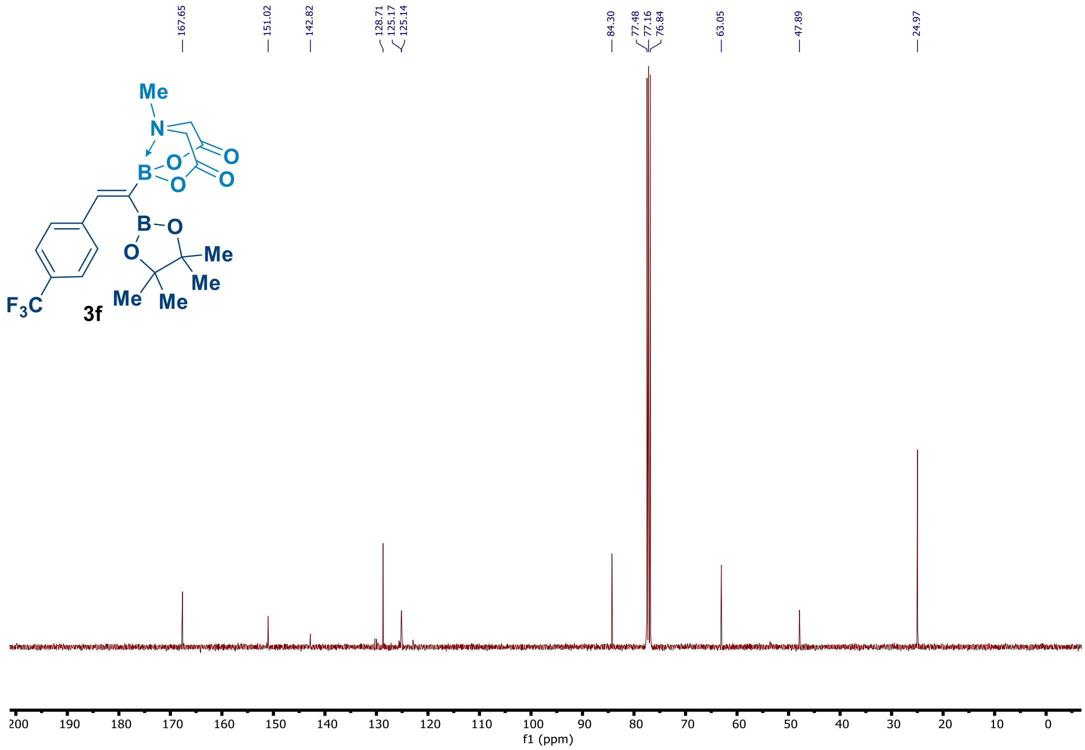

Supplementary Figure 177. ^{13}C NMR (101 MHz, $CDCl_3$) of compound (**3f**).

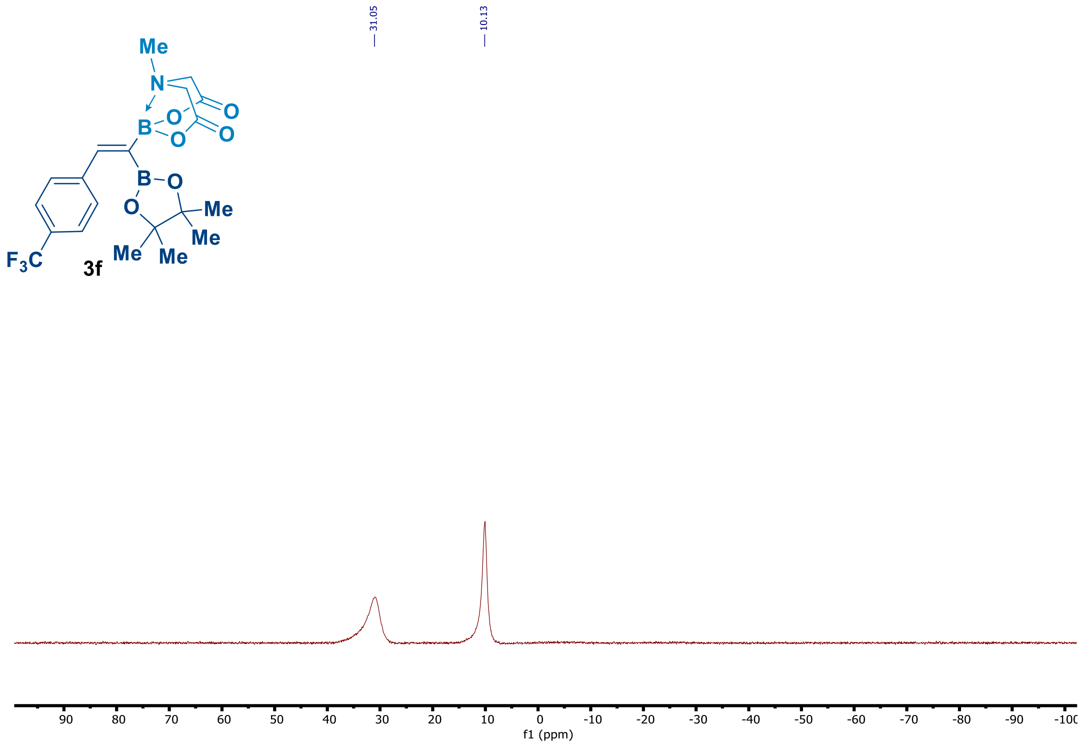

Supplementary Figure 178. ^{11}B NMR (128 MHz, $CDCl_3$) of compound (**3f**).

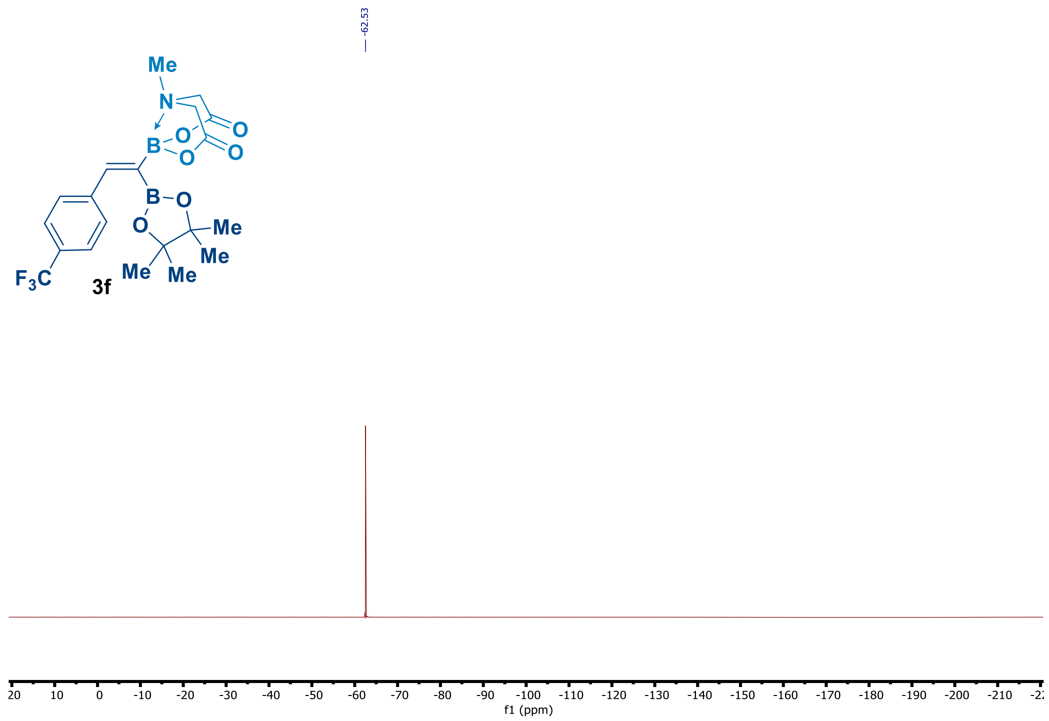

Supplementary Figure 179. ^{19}F NMR (376 MHz, $CDCl_3$) of compound (**3f**).

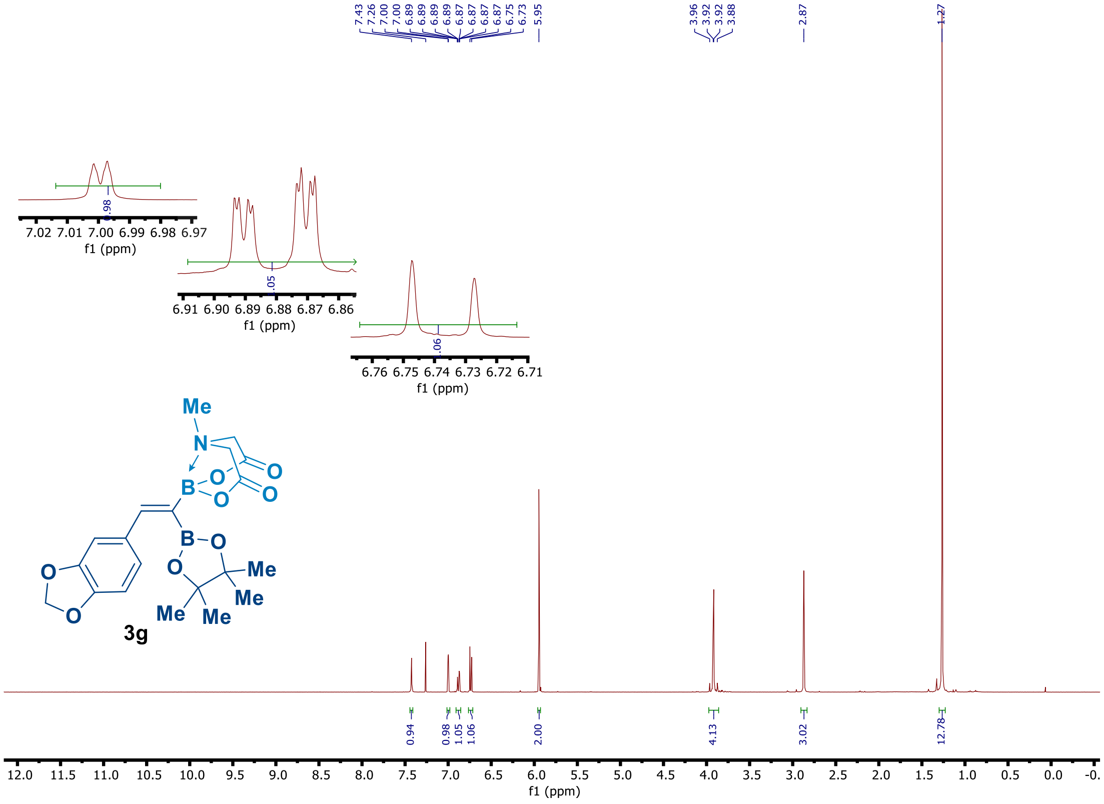

Supplementary Figure 180. ^{1}H NMR (400 MHz, $CDCl_3$) of compound (**3g**).

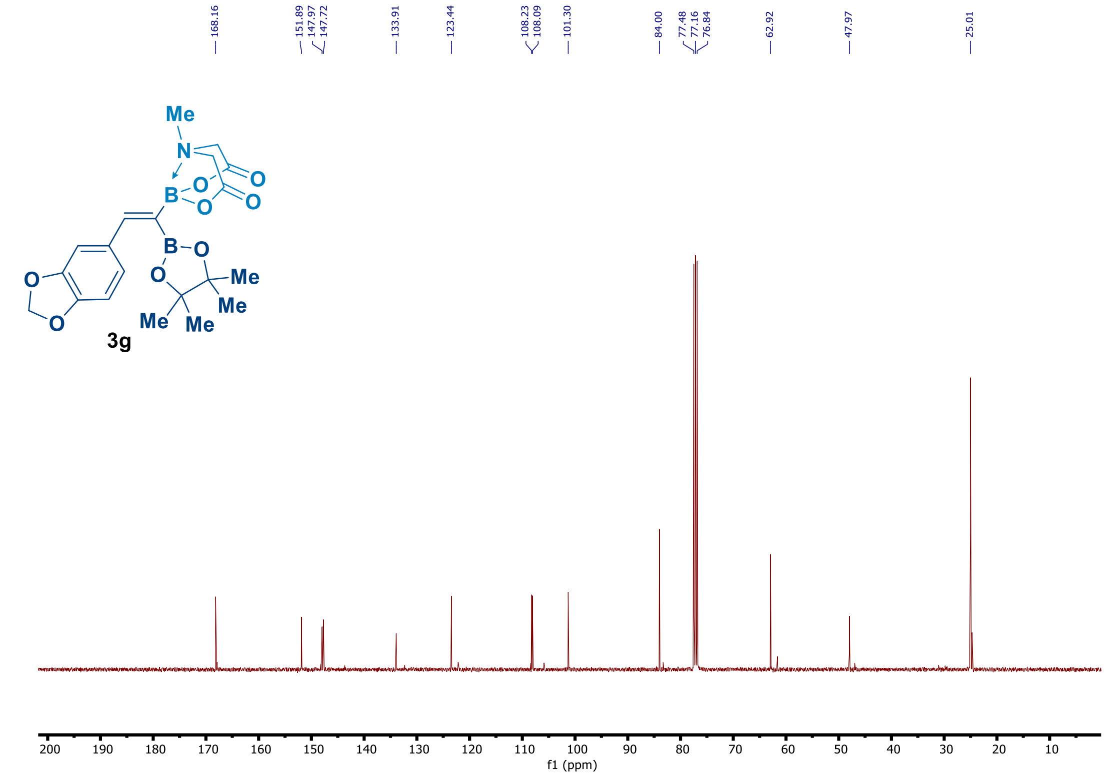

Supplementary Figure 181. ^{13}C NMR (101 MHz, $CDCl_3$) of compound (**3g**).

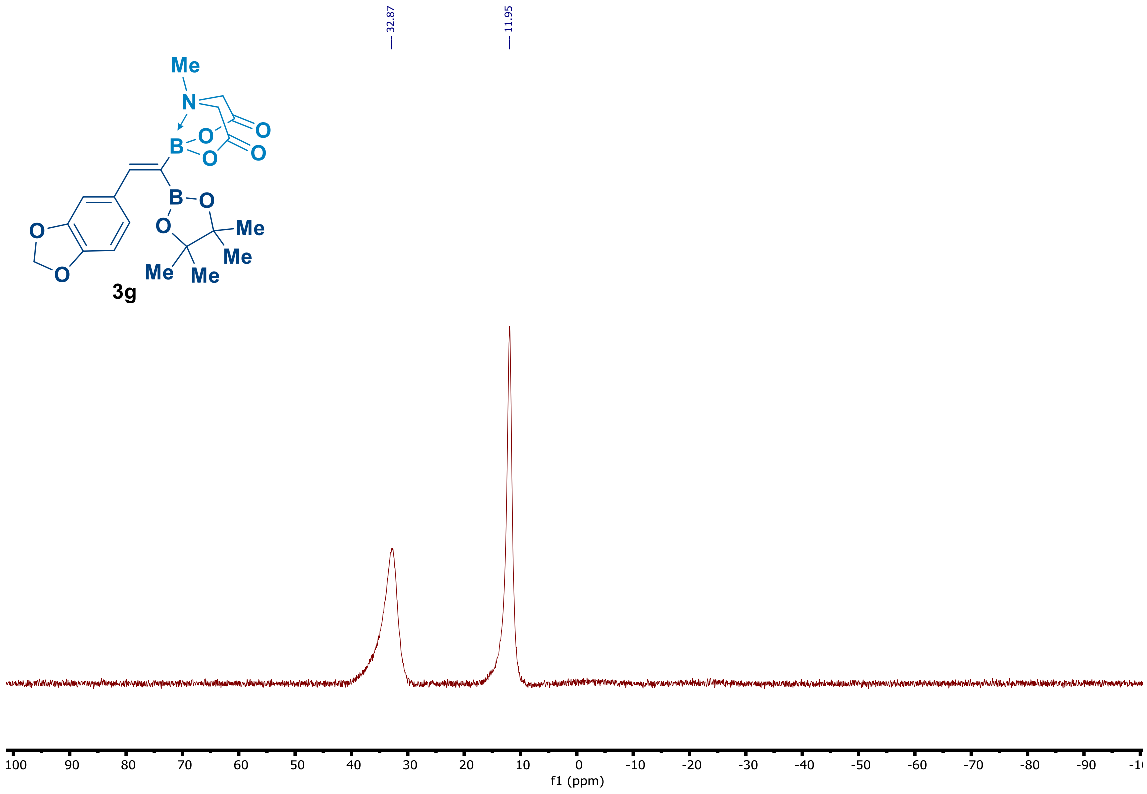

Supplementary Figure 182. ^{11}B NMR (128 MHz, $CDCl_3$) of compound (**3g**).

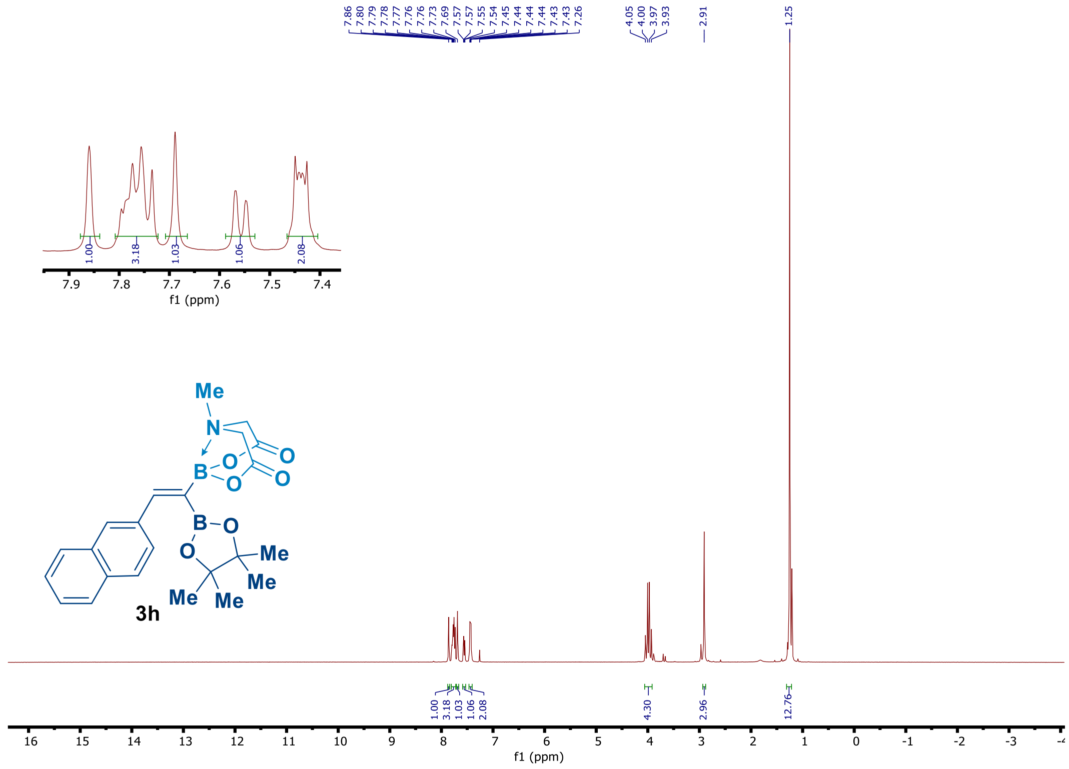

Supplementary Figure 183. ^{1}H NMR (400 MHz, $CDCl_3$) of compound (**3h**).

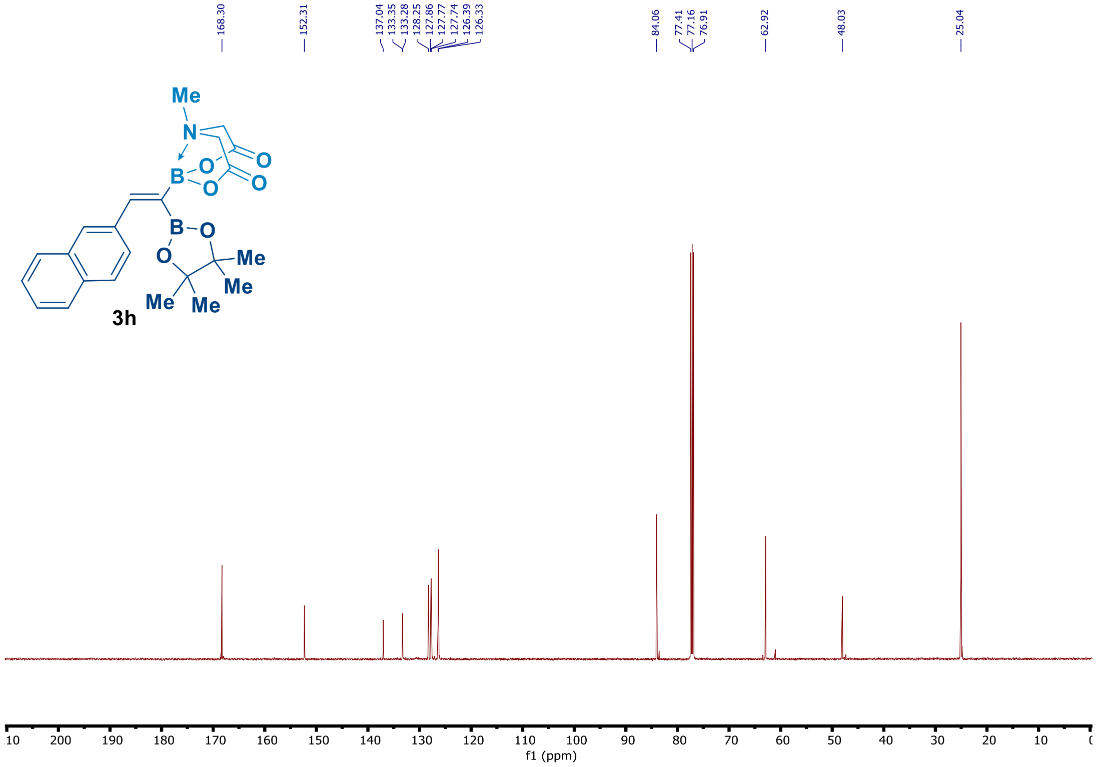

Supplementary Figure 184. ^{13}C NMR (101 MHz, $CDCl_3$) of compound (**3h**).

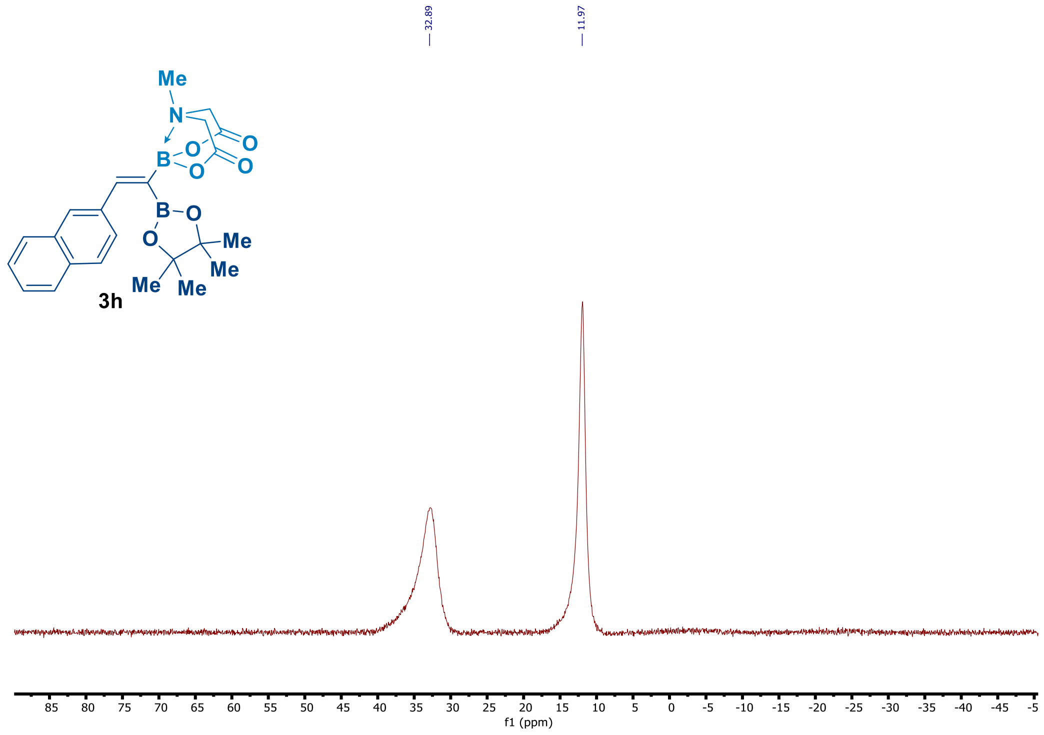

Supplementary Figure 185. ^{11}B NMR (128 MHz, $CDCl_3$) of compound (**3h**).

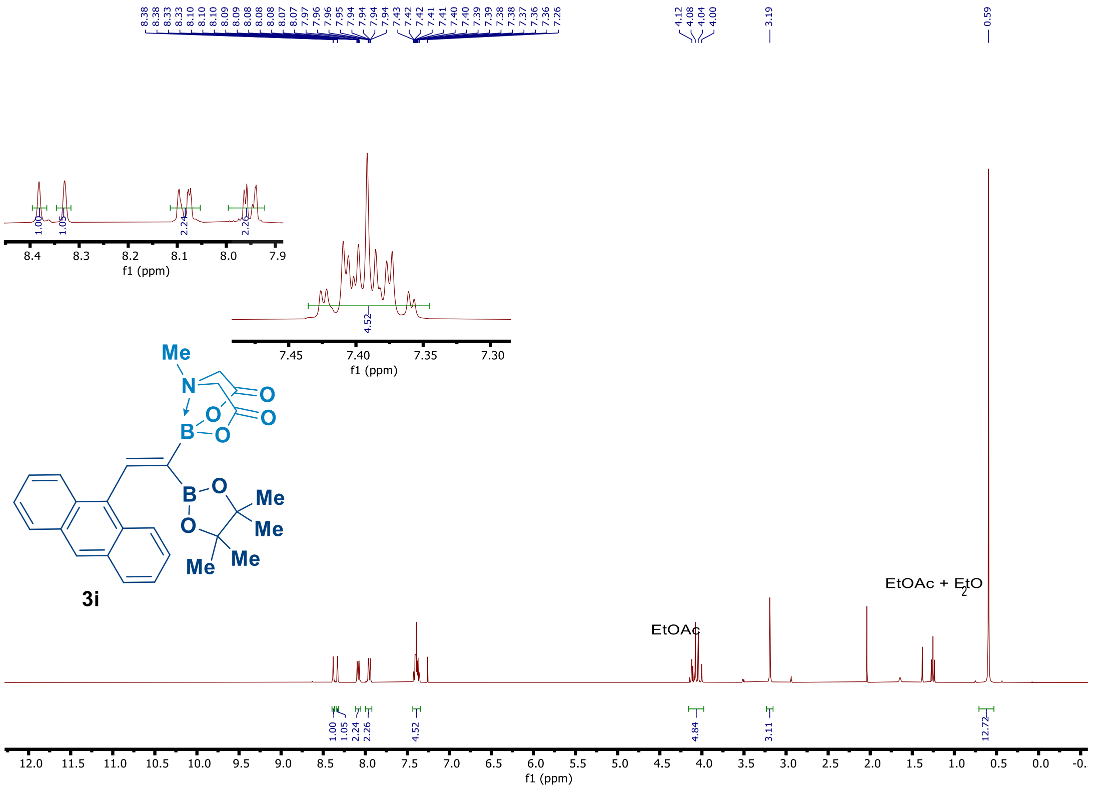

Supplementary Figure 186. ^{1}H NMR (400 MHz, $CDCl_3$) of compound (**3i**).

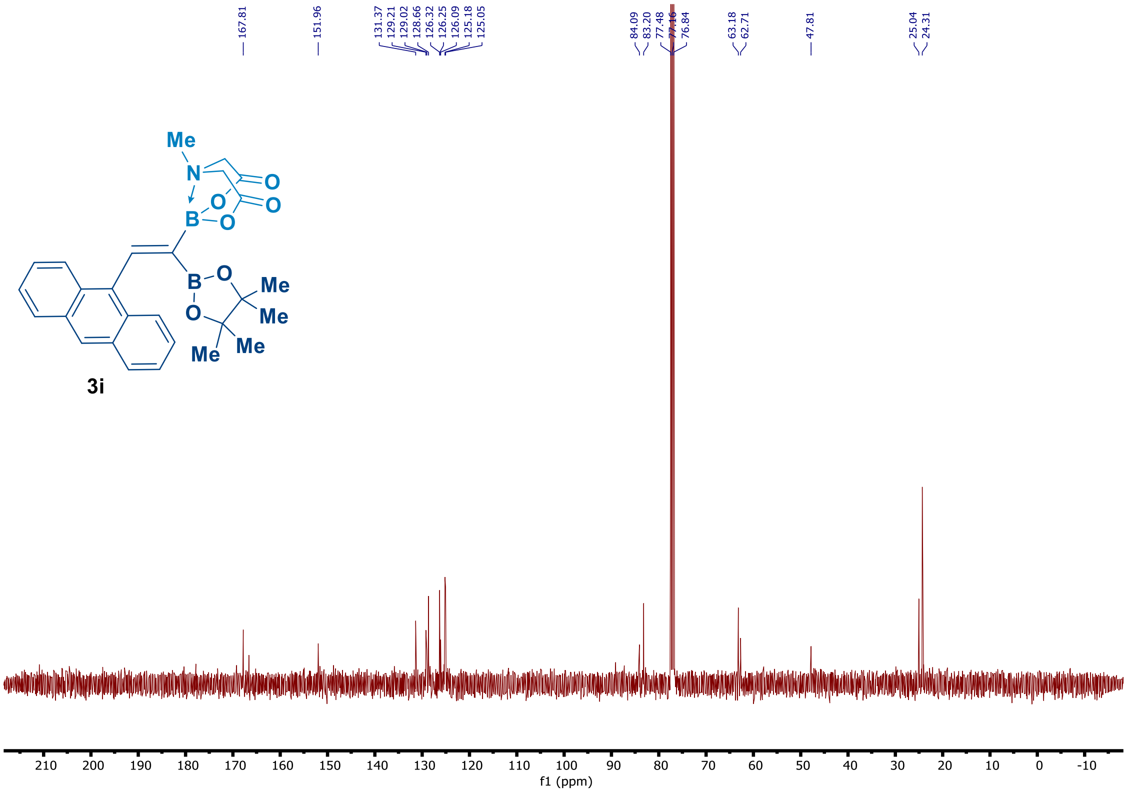

Supplementary Figure 187. ^{13}C NMR (101 MHz, $CDCl_3$) of compound (**3i**).

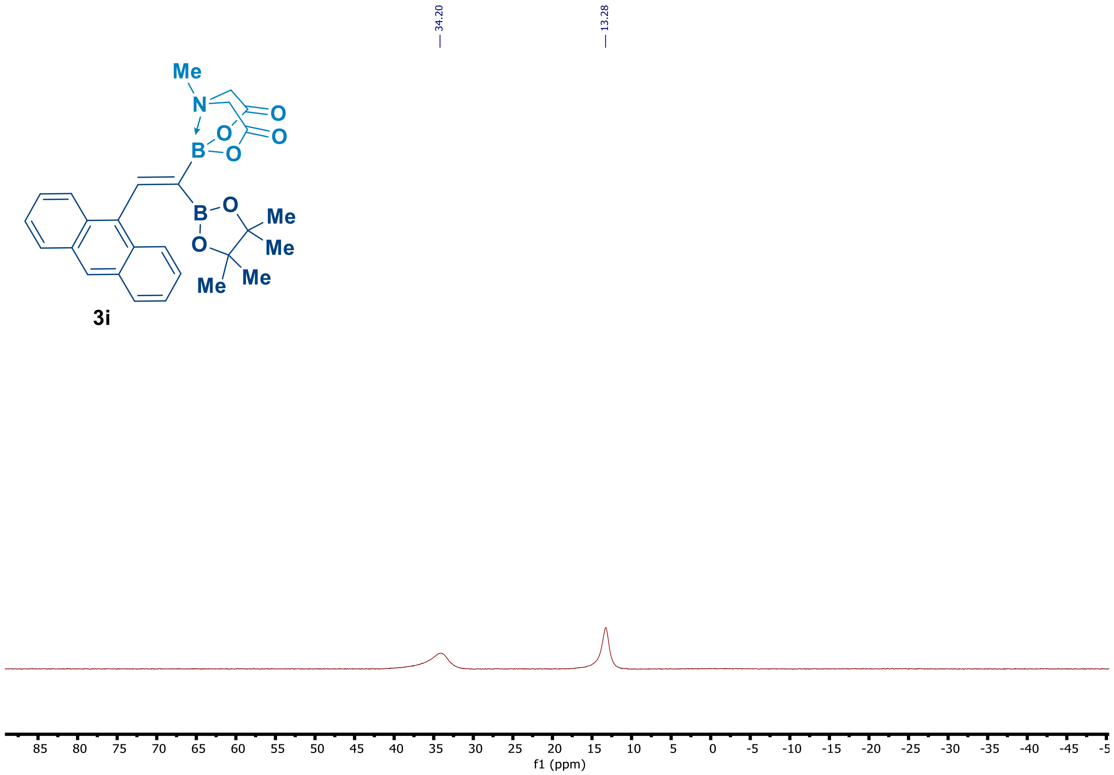

Supplementary Figure 188. ^{11}B NMR (128 MHz, $CDCl_3$) of compound (**3i**).

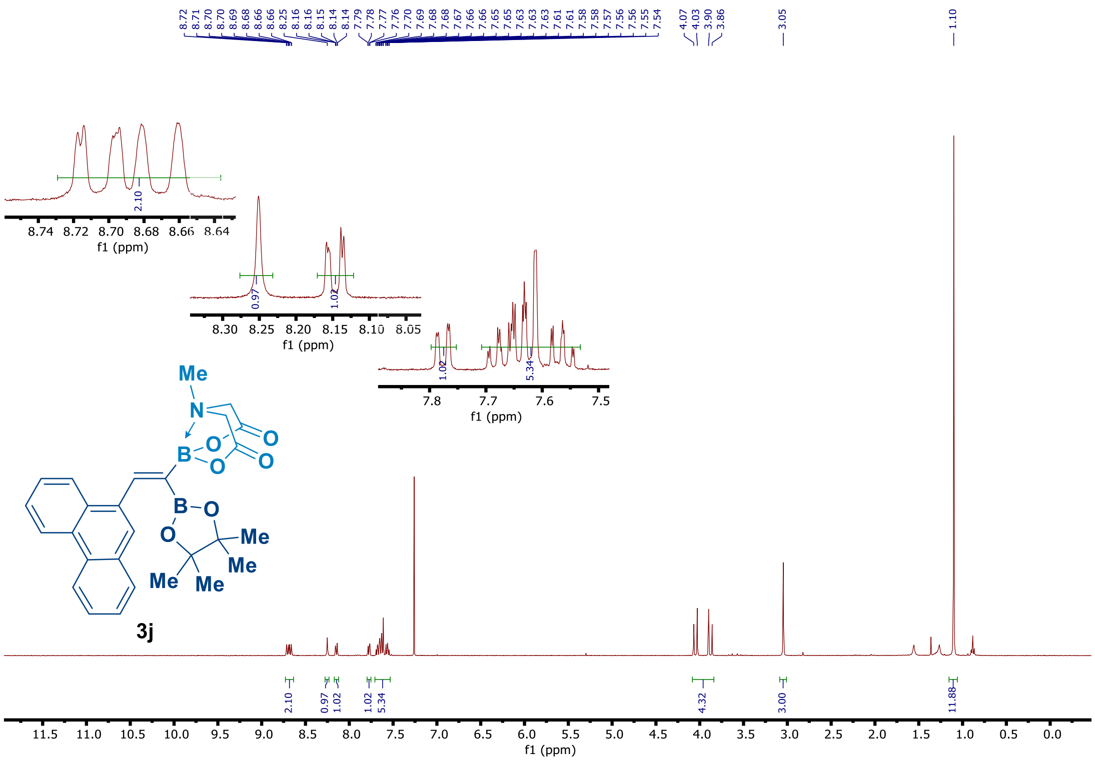

Supplementary Figure 189. ^{1}H NMR (400 MHz, $CDCl_3$) of compound (**3j**).

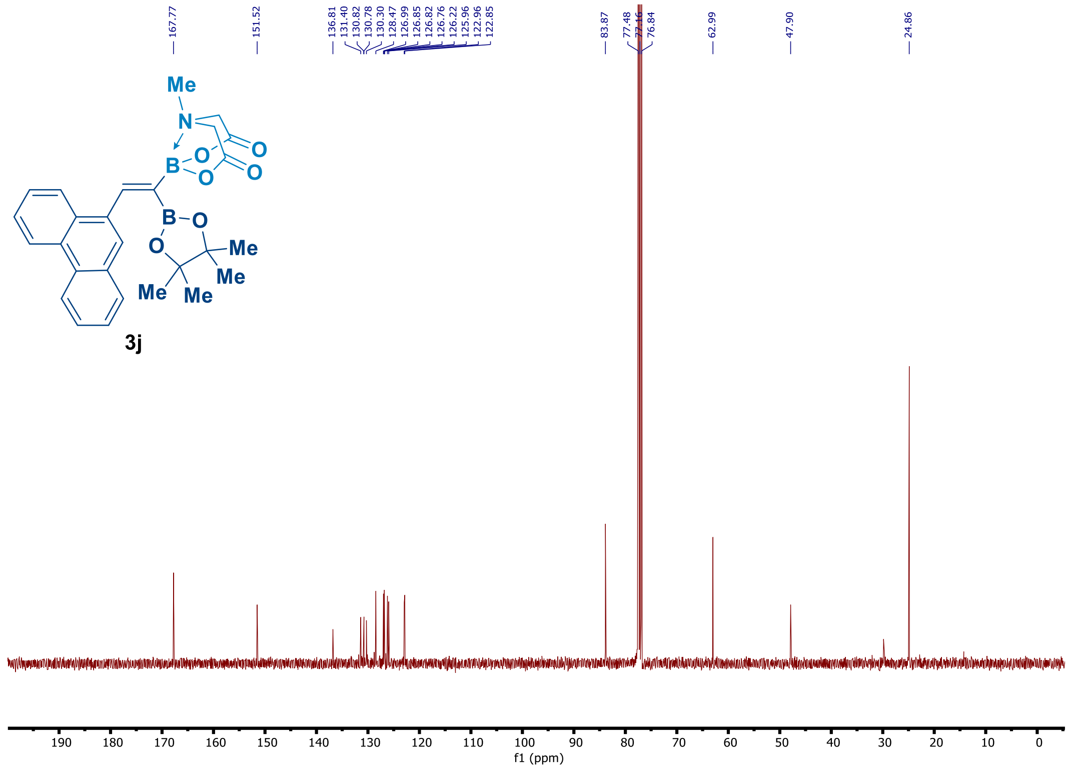

Supplementary Figure 190. ^{13}C NMR (101 MHz, $CDCl_3$) of compound (**3j**).

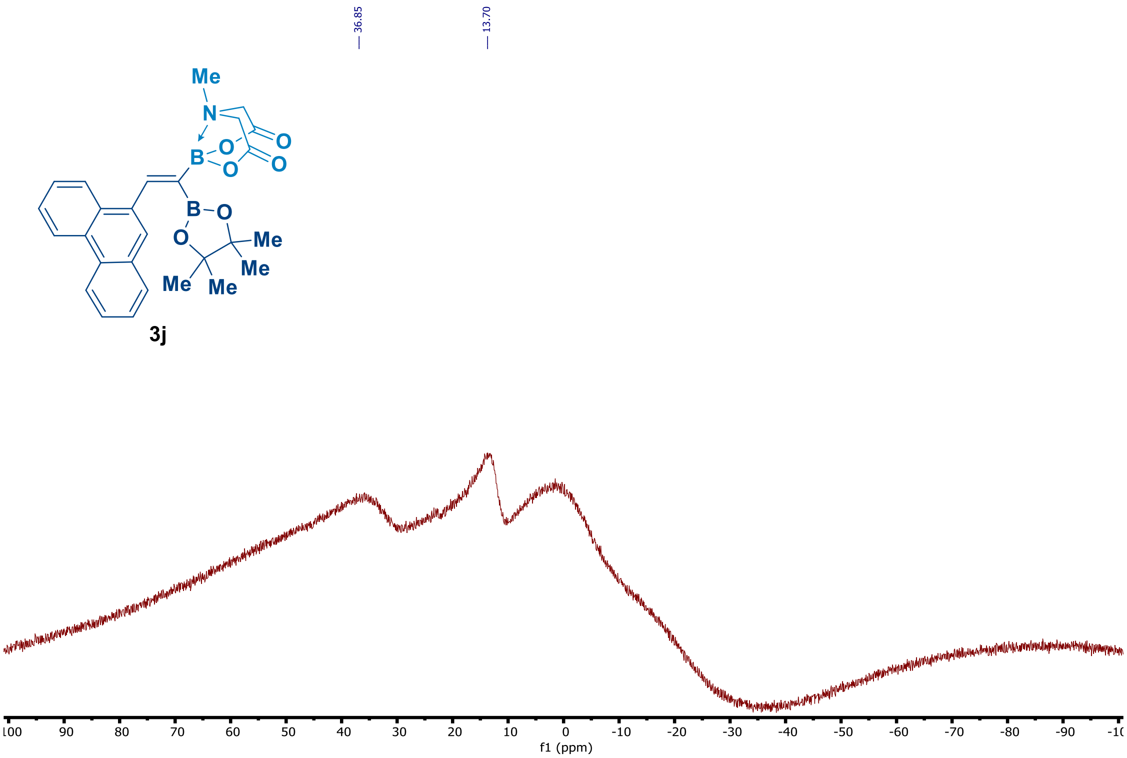

Supplementary Figure 191. ^{11}B NMR (128 MHz, $CDCl_3$) of compound (**3j**).

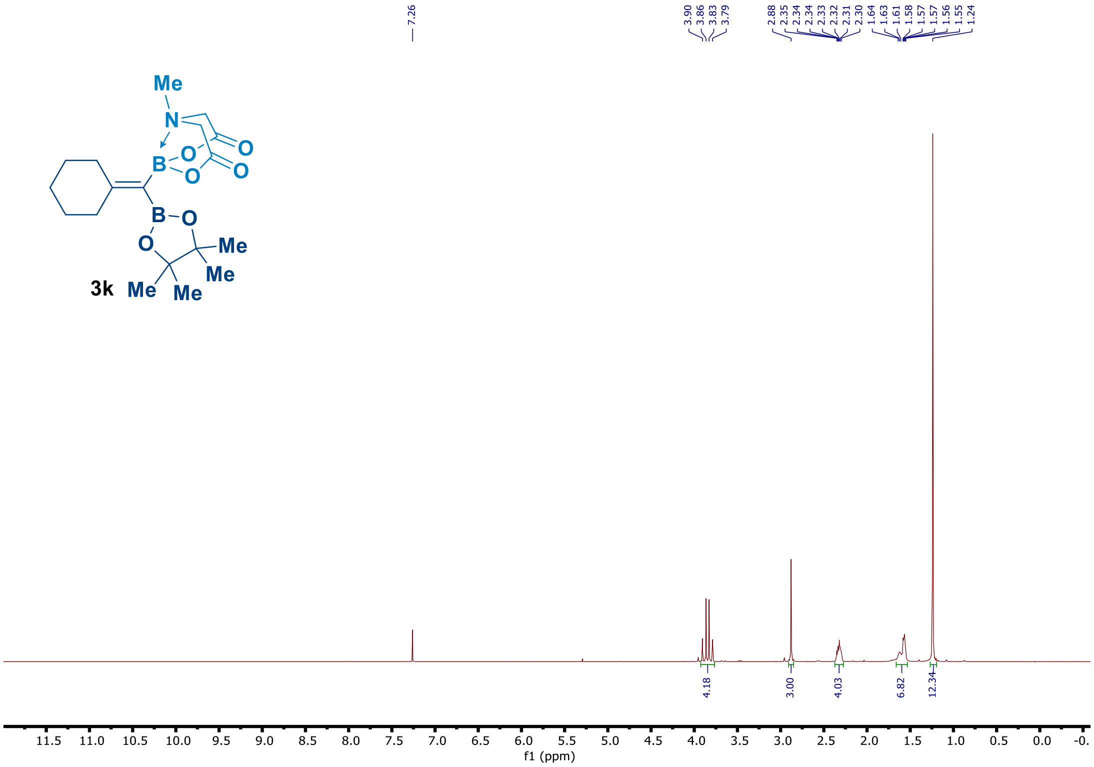

Supplementary Figure 192. ^{1}H NMR (400 MHz, $CDCl_3$) of compound (**3k**).

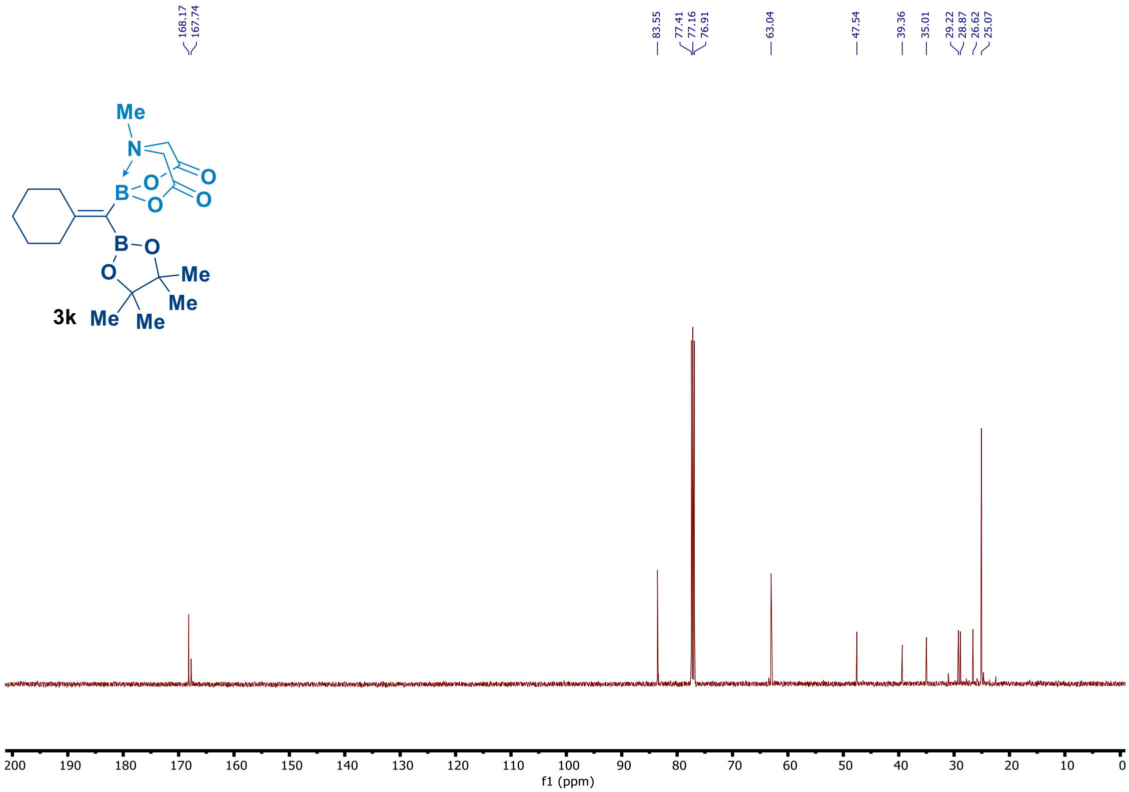

Supplementary Figure 193. ^{13}C NMR (101 MHz, $CDCl_3$) of compound (**3k**).

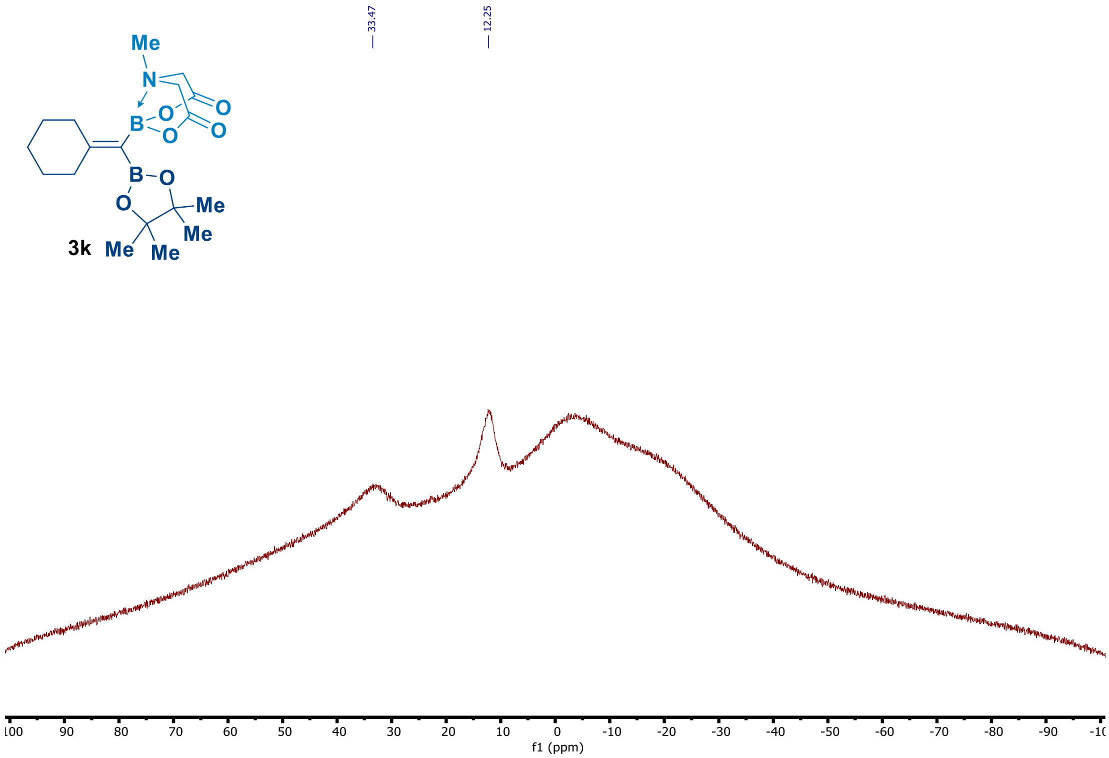

Supplementary Figure 194. ^{11}B NMR (128 MHz, $CDCl_3$) of compound (**3k**).

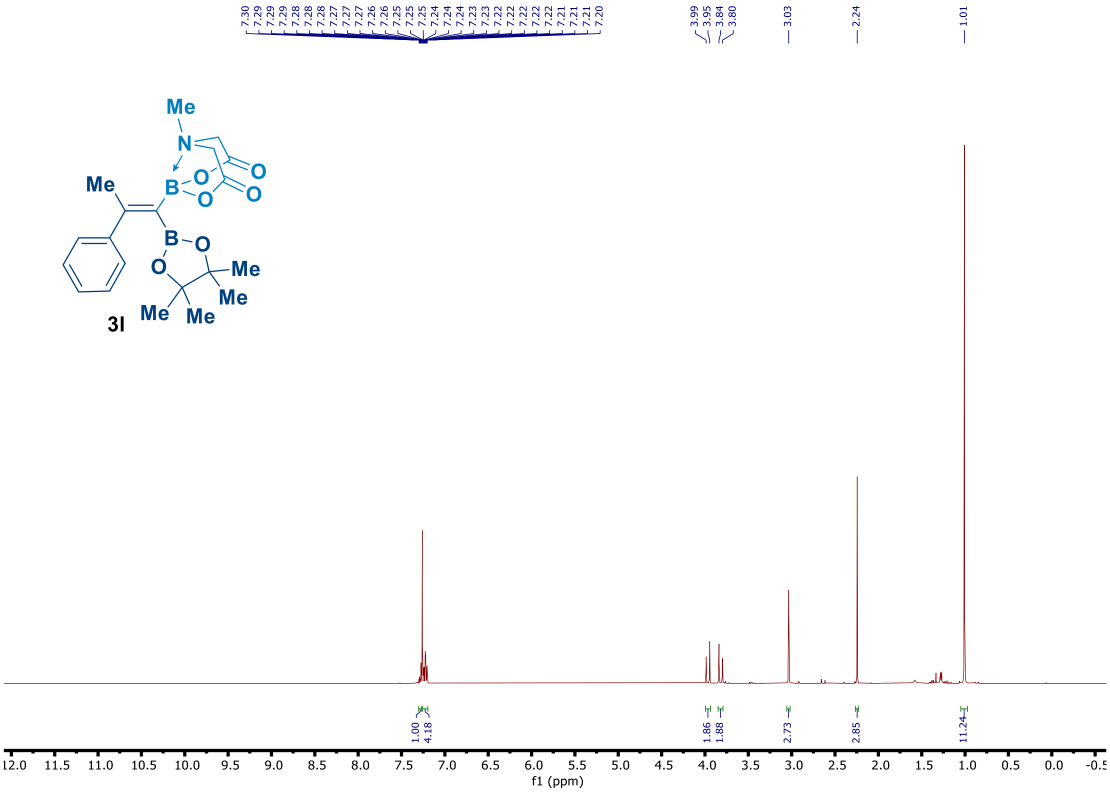

Supplementary Figure 195. ^{1}H NMR (400 MHz, $CDCl_3$) of compound (**3l**).

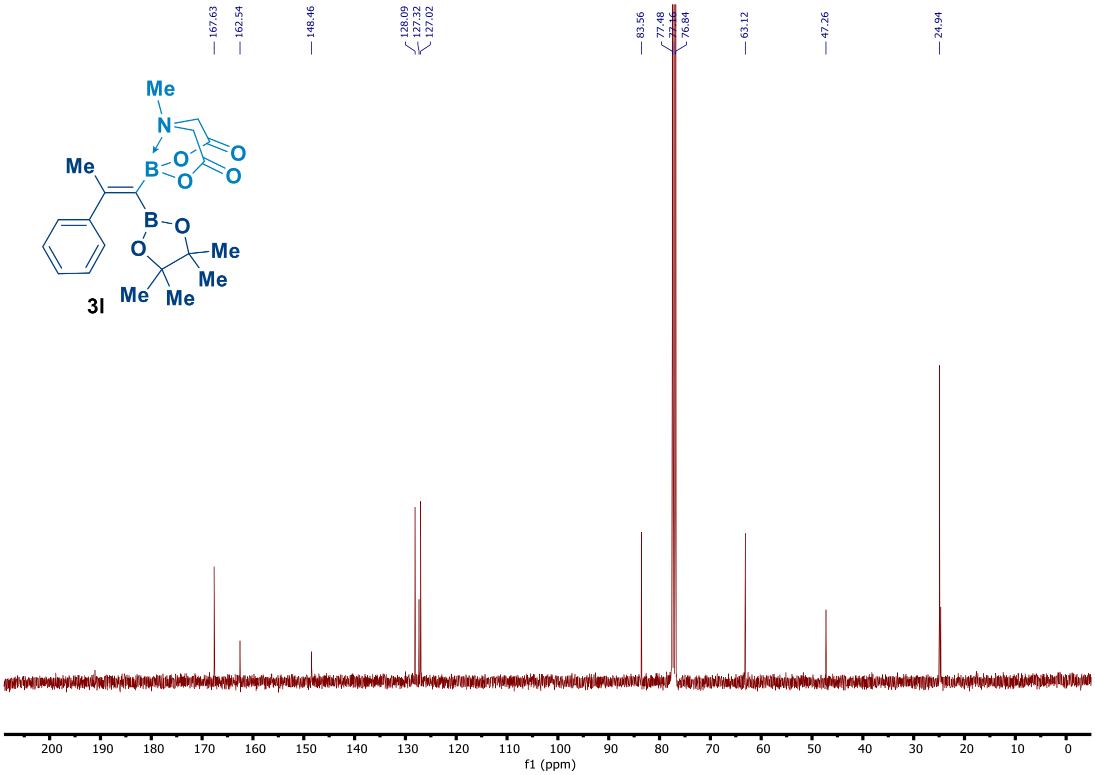

Supplementary Figure 196. ^{13}C NMR (101 MHz, $CDCl_3$) of compound (**3l**).

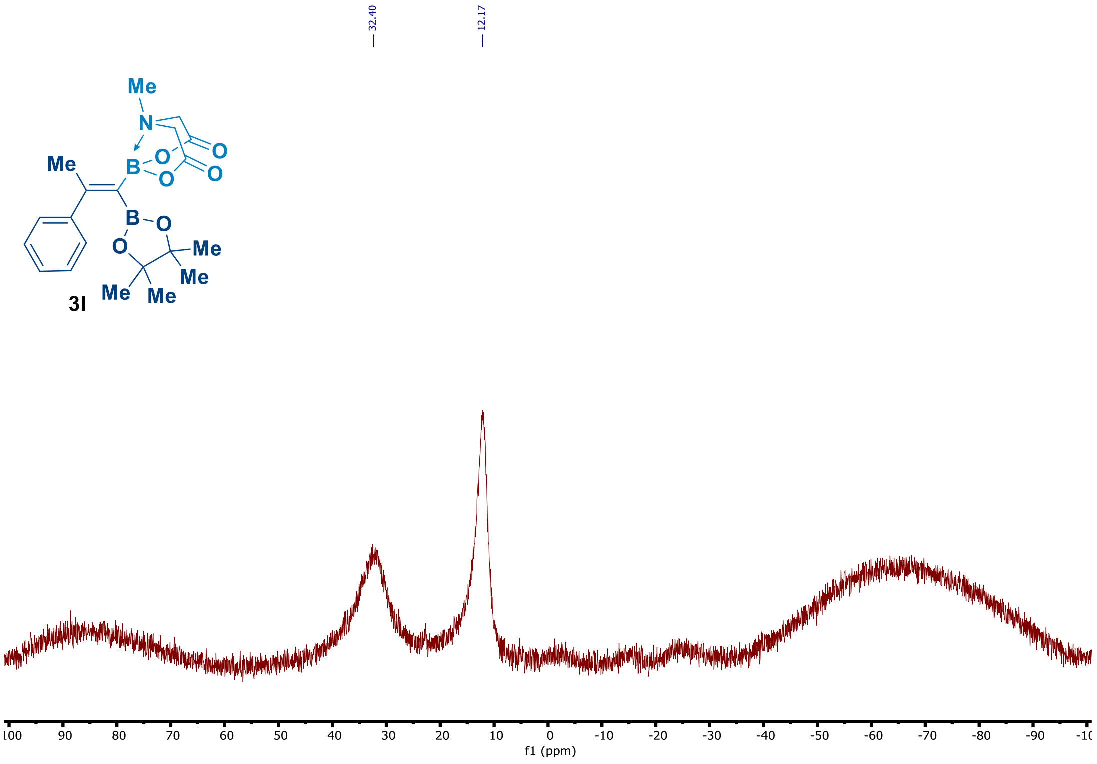

Supplementary Figure 197. ^{11}B NMR (128 MHz, $CDCl_3$) of compound (**3l**).

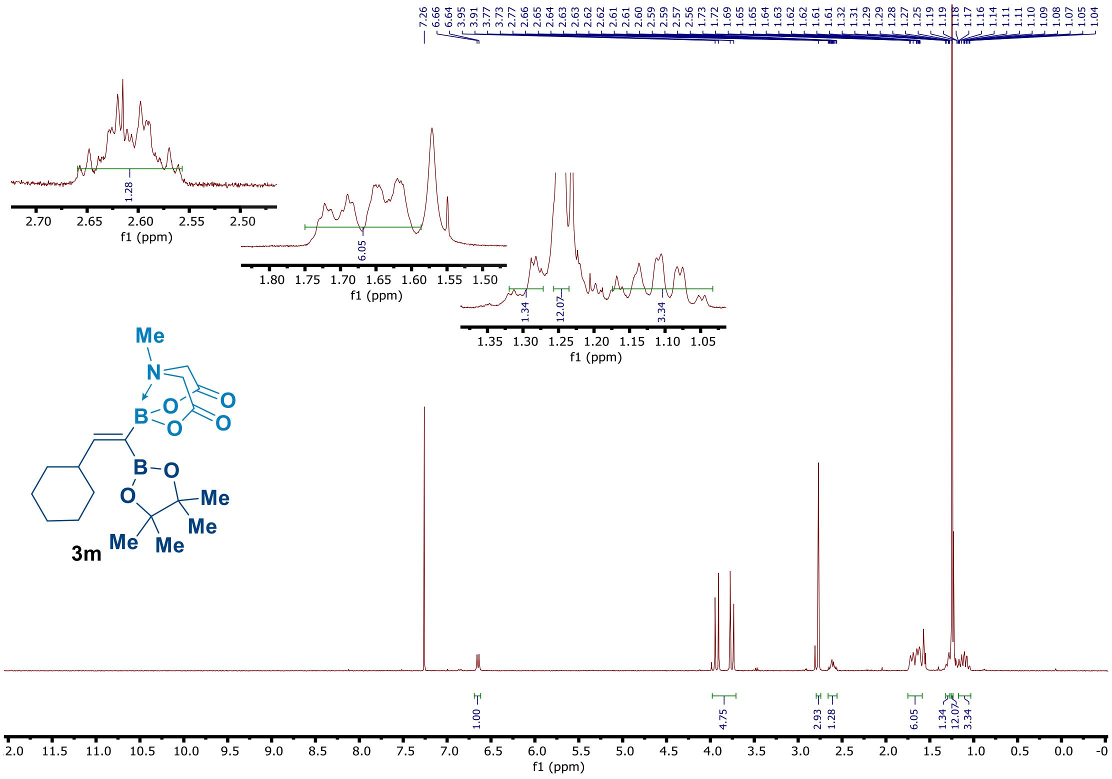

Supplementary Figure 198. ^{1}H NMR (400 MHz, $CDCl_3$) of compound (**3m**).

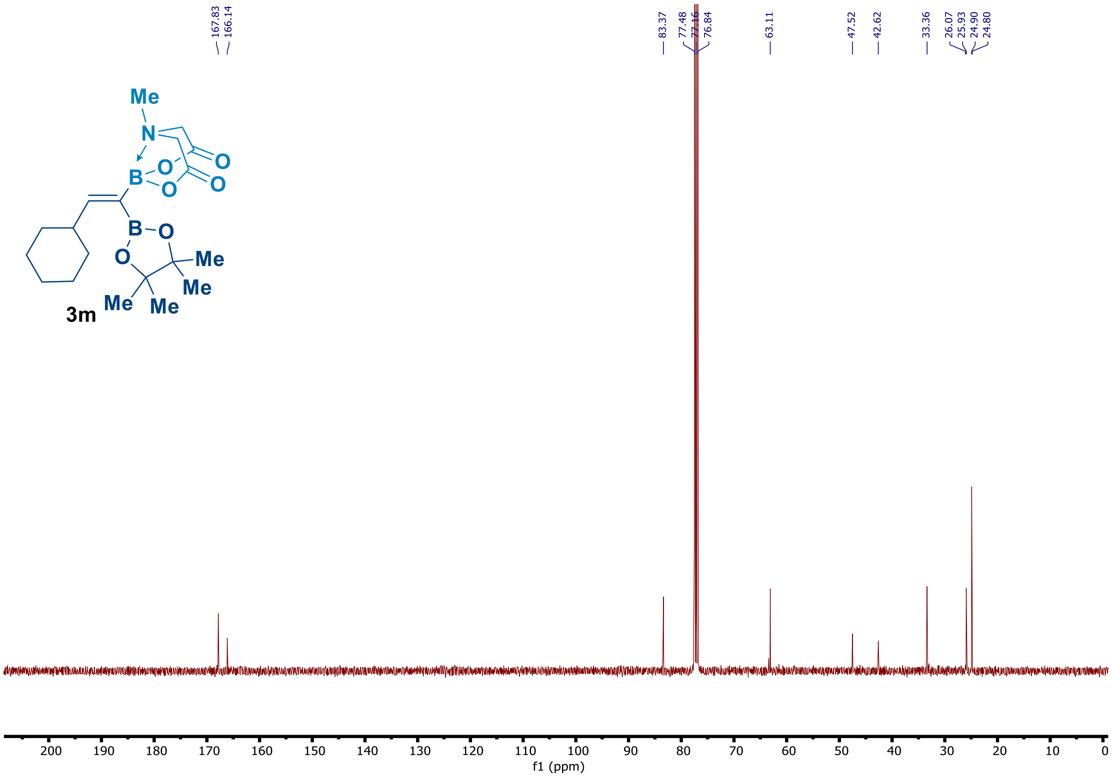

Supplementary Figure 199. ^{13}C NMR (101 MHz, $CDCl_3$) of compound (**3m**).

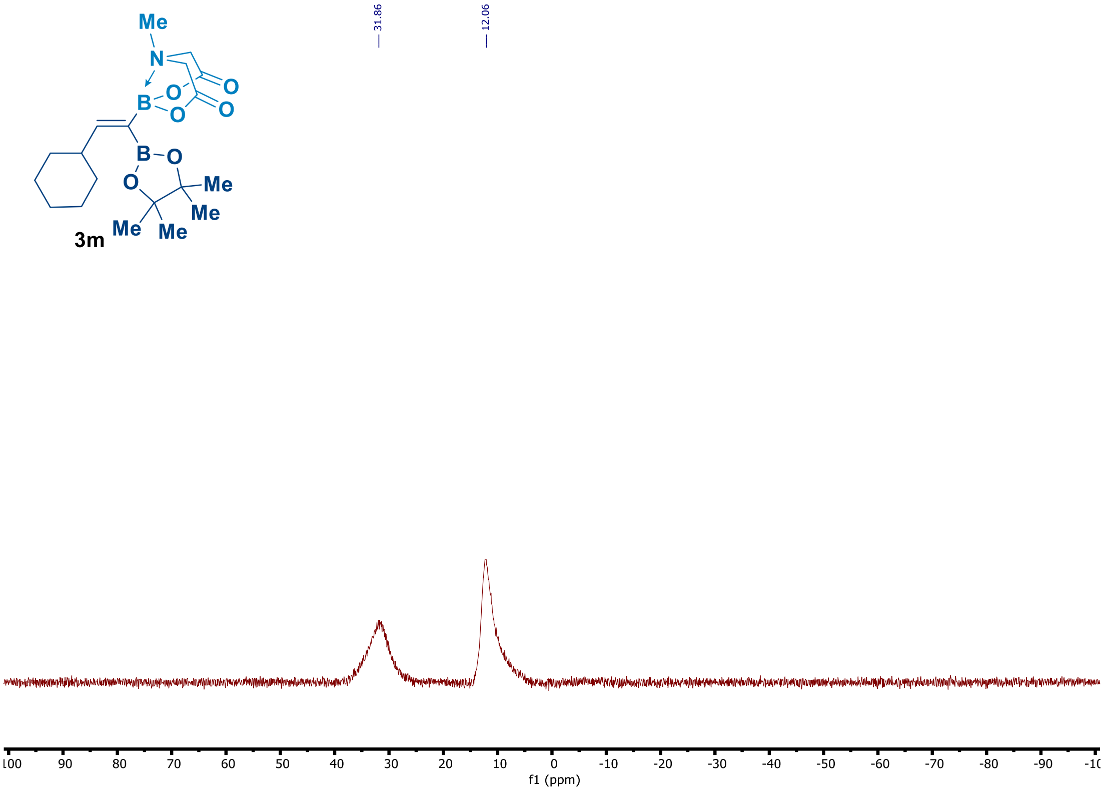

Supplementary Figure 200. ^{11}B NMR (128 MHz, $CDCl_3$) of compound (**3m**).

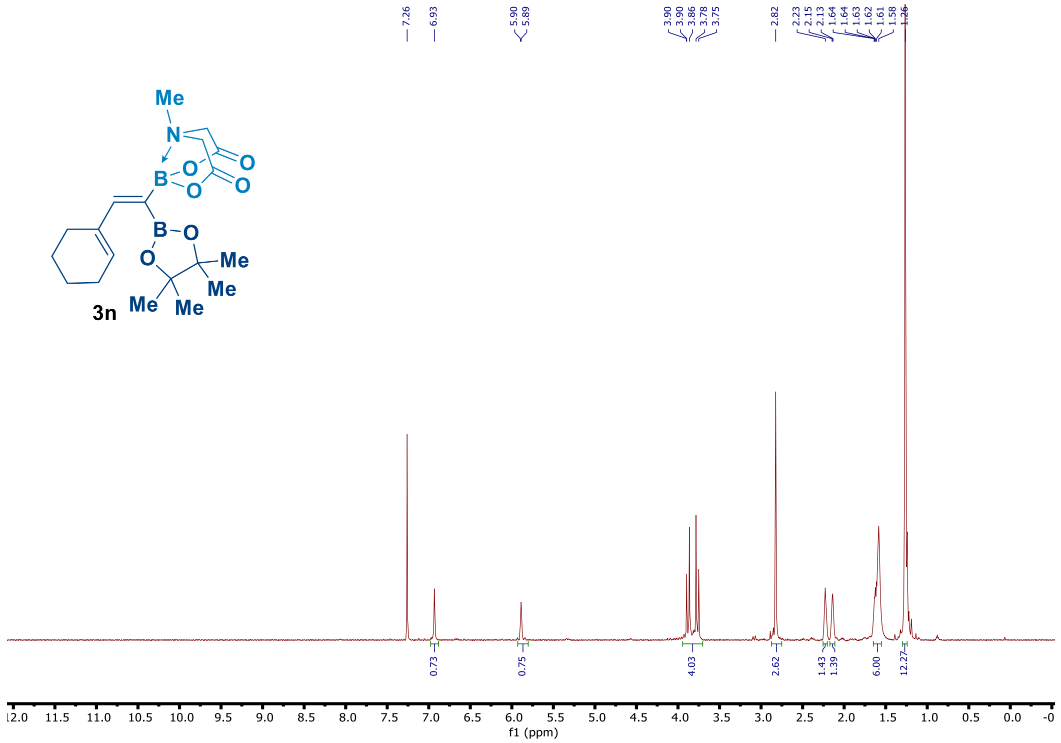

Supplementary Figure 201. ^{1}H NMR (400 MHz, $CDCl_3$) of compound (**3n**).

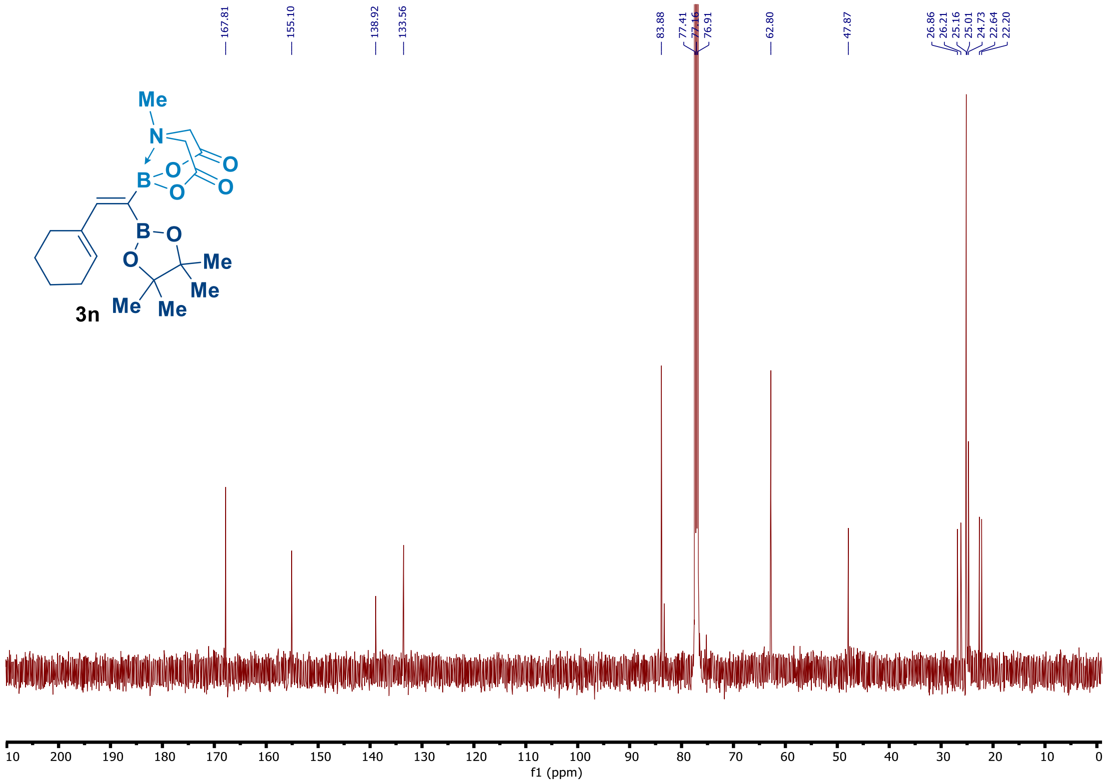

Supplementary Figure 202. ^{13}C NMR (101 MHz, $CDCl_3$) of compound (**3n**).

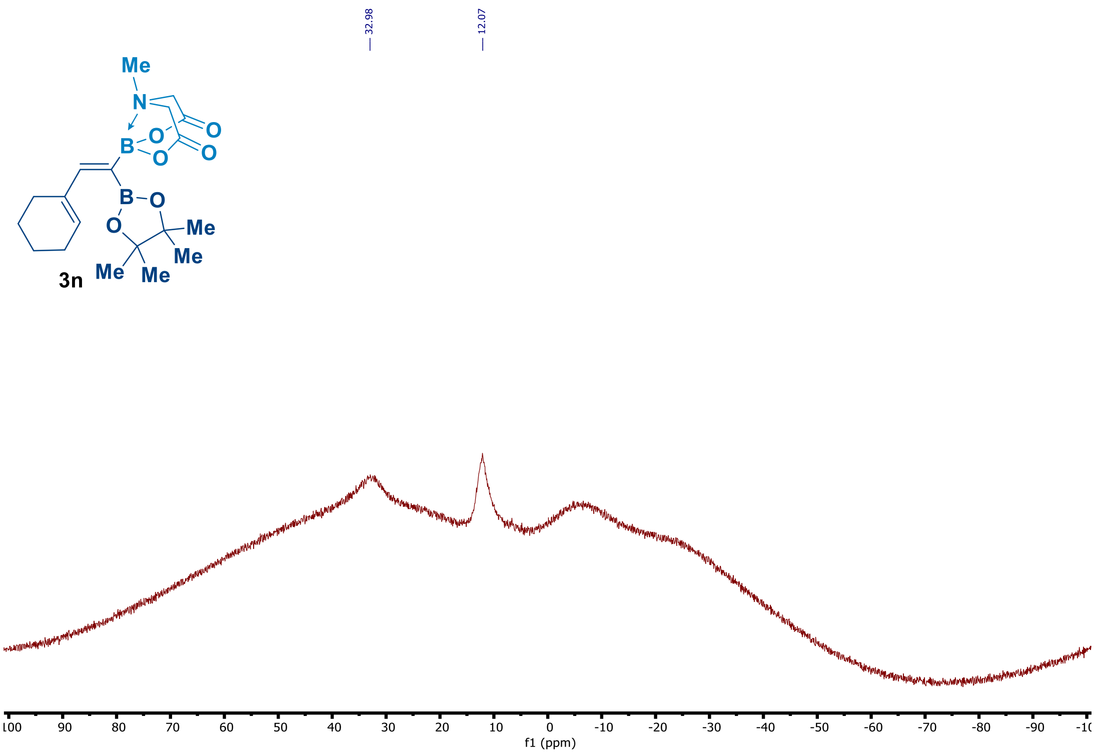

Supplementary Figure 203. ^{11}B NMR (128 MHz, $CDCl_3$) of compound (**3n**).

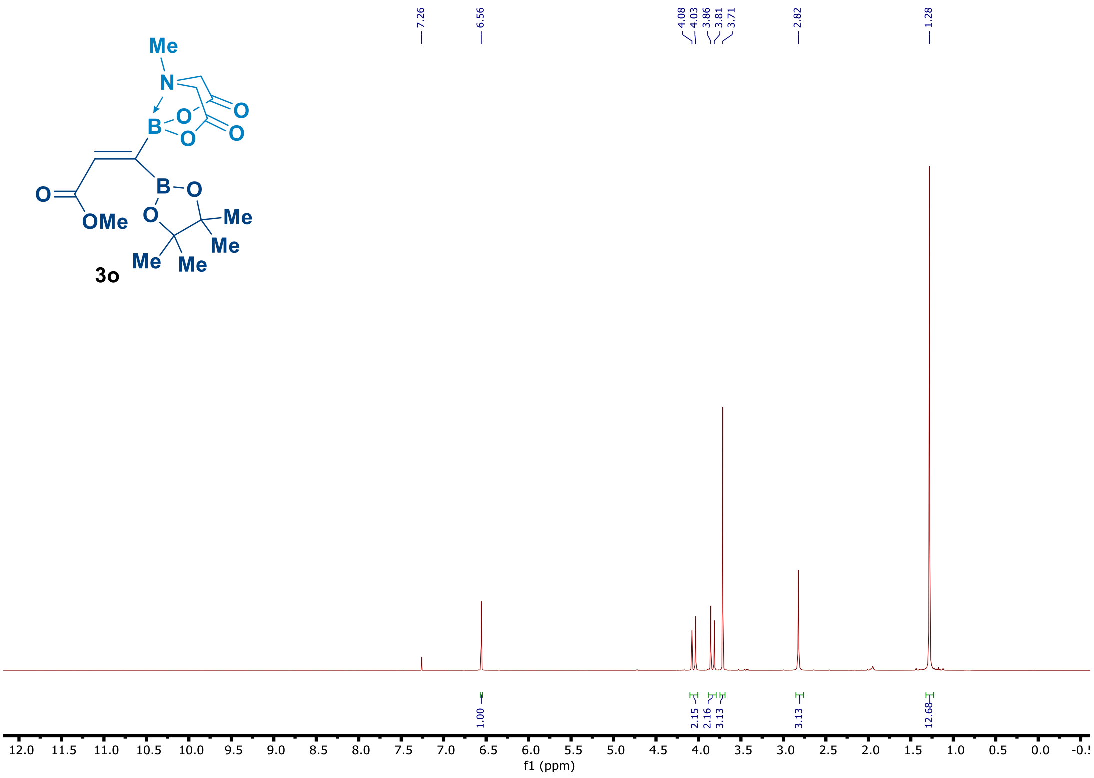

Supplementary Figure 204. ^{1}H NMR (400 MHz, $CDCl_3$) of compound (**3o**).

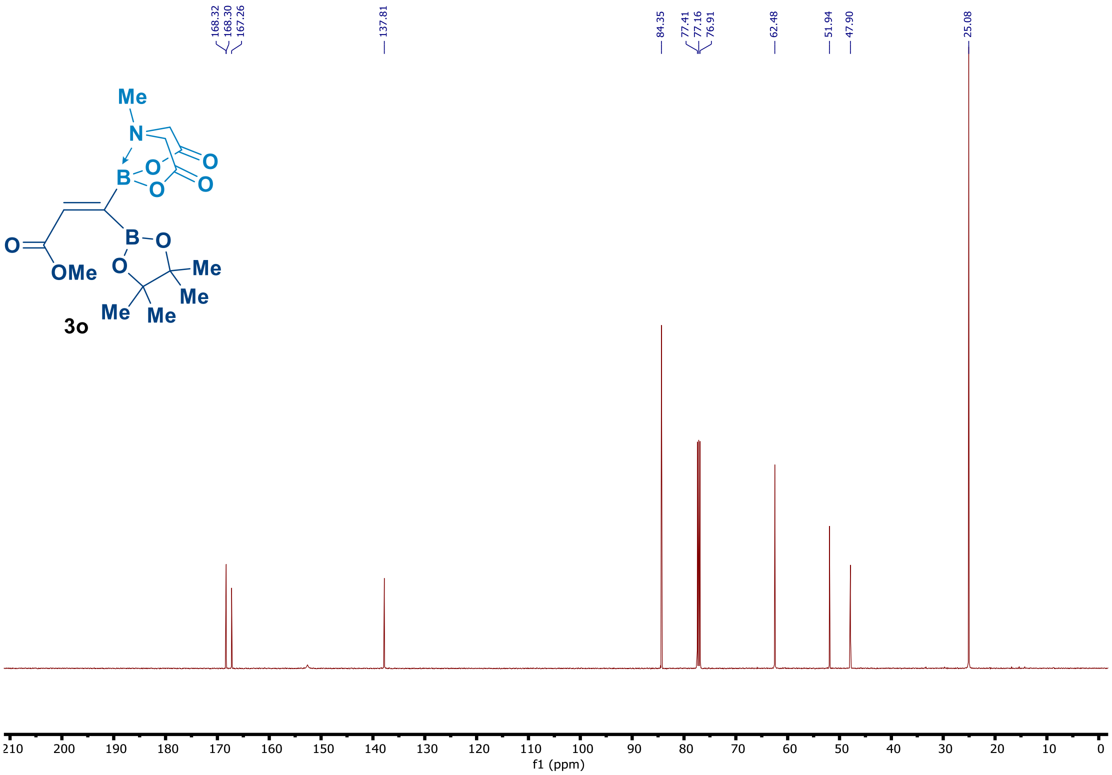

Supplementary Figure 205. ^{13}C NMR (101 MHz, $CDCl_3$) of compound (**3o**).

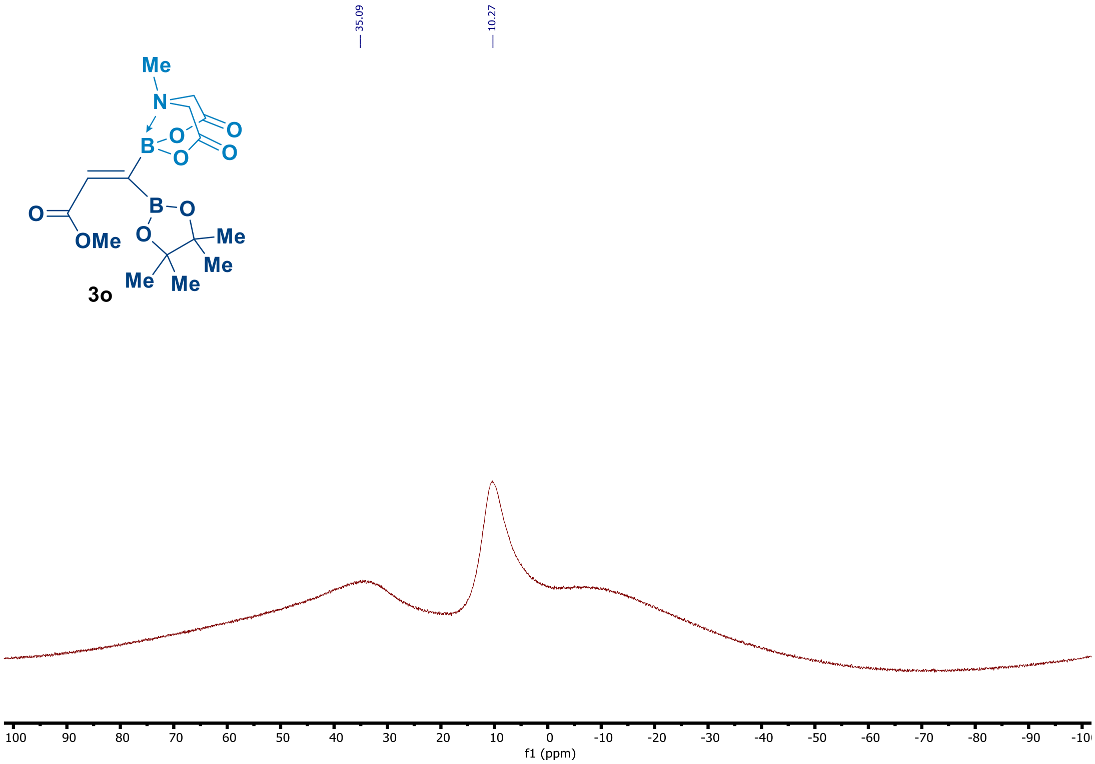

Supplementary Figure 206. ^{11}B NMR (128 MHz, $CDCl_3$) of compound (**3o**).

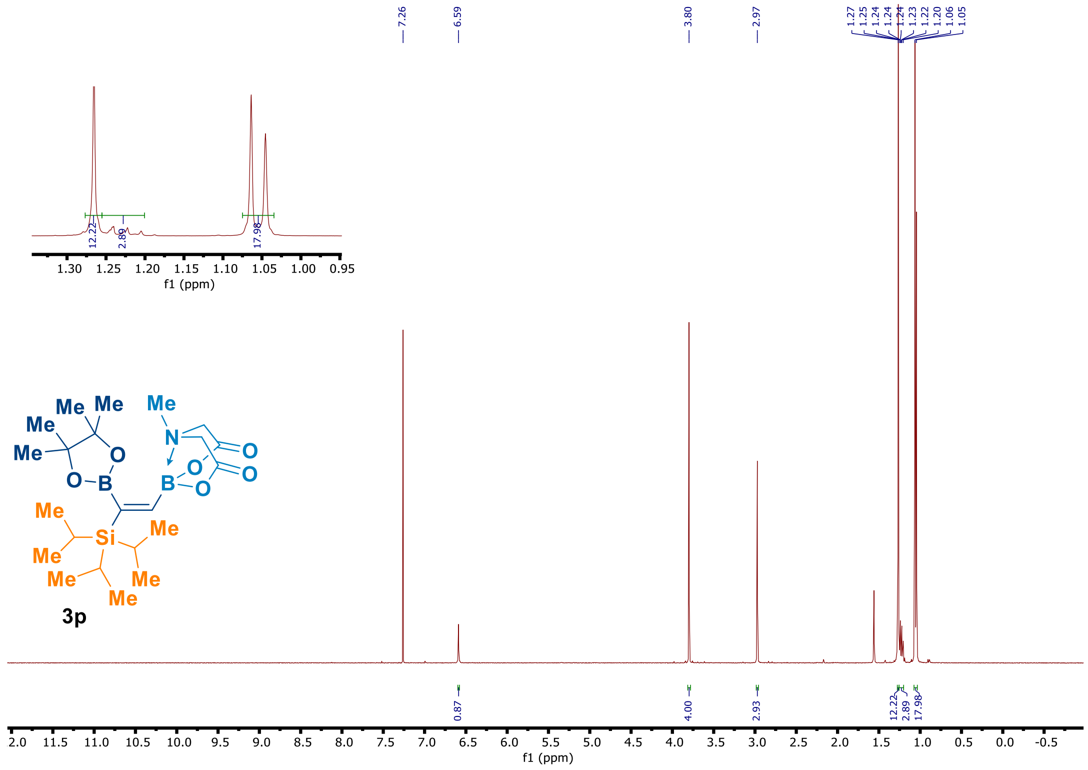

Supplementary Figure 207. ^{1}H NMR (400 MHz, $CDCl_3$) of compound (**3p**).

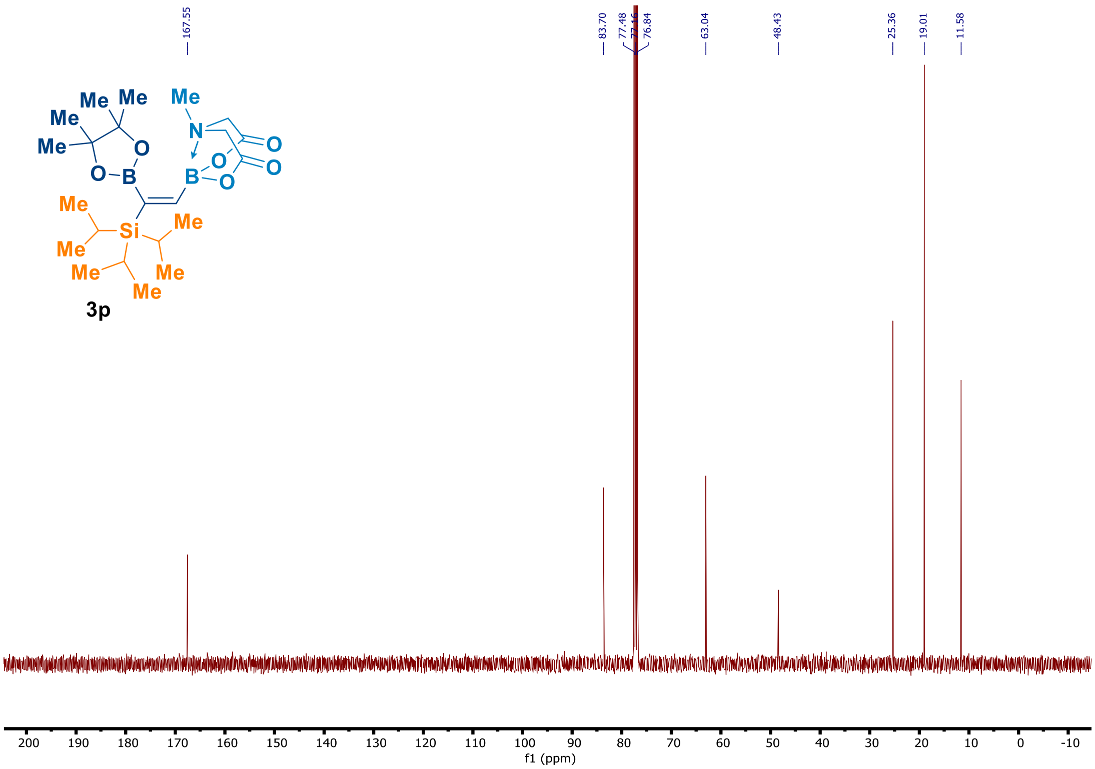

Supplementary Figure 208. ^{13}C NMR (101 MHz, $CDCl_3$) of compound (**3p**).

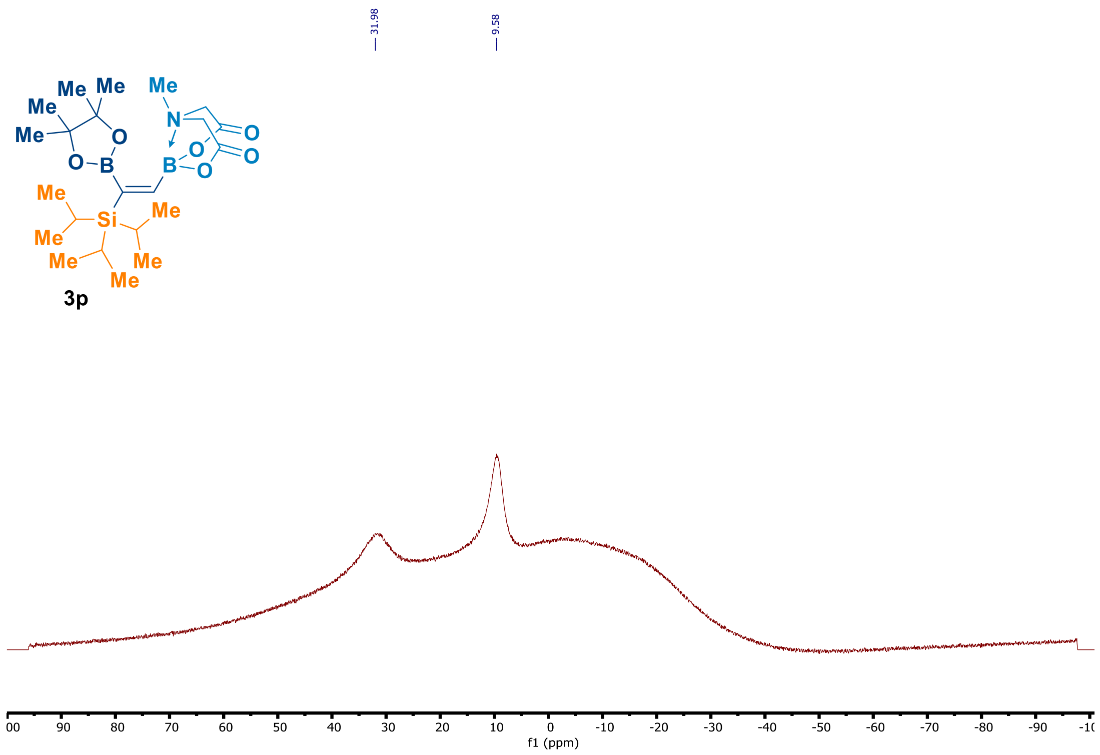

Supplementary Figure 209. ^{11}B NMR (128 MHz, $CDCl_3$) of compound (**3p**).

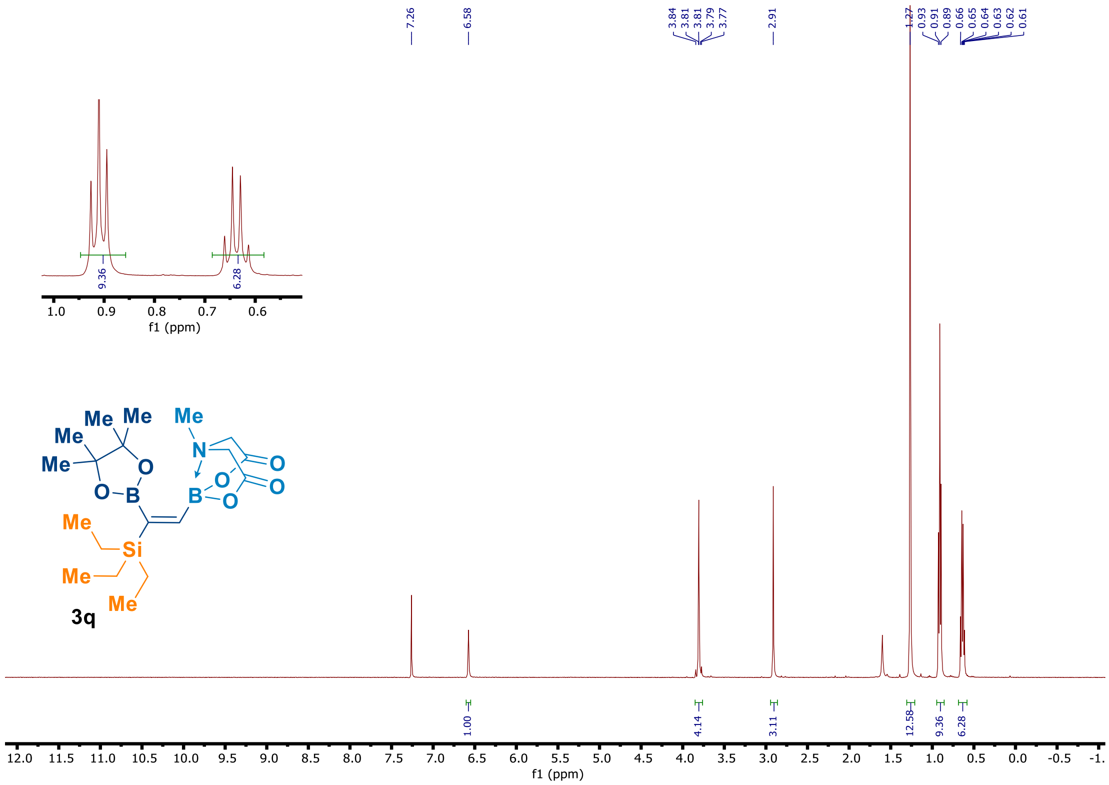

Supplementary Figure 210. ^{1}H NMR (400 MHz, $CDCl_3$) of compound (**3q**).

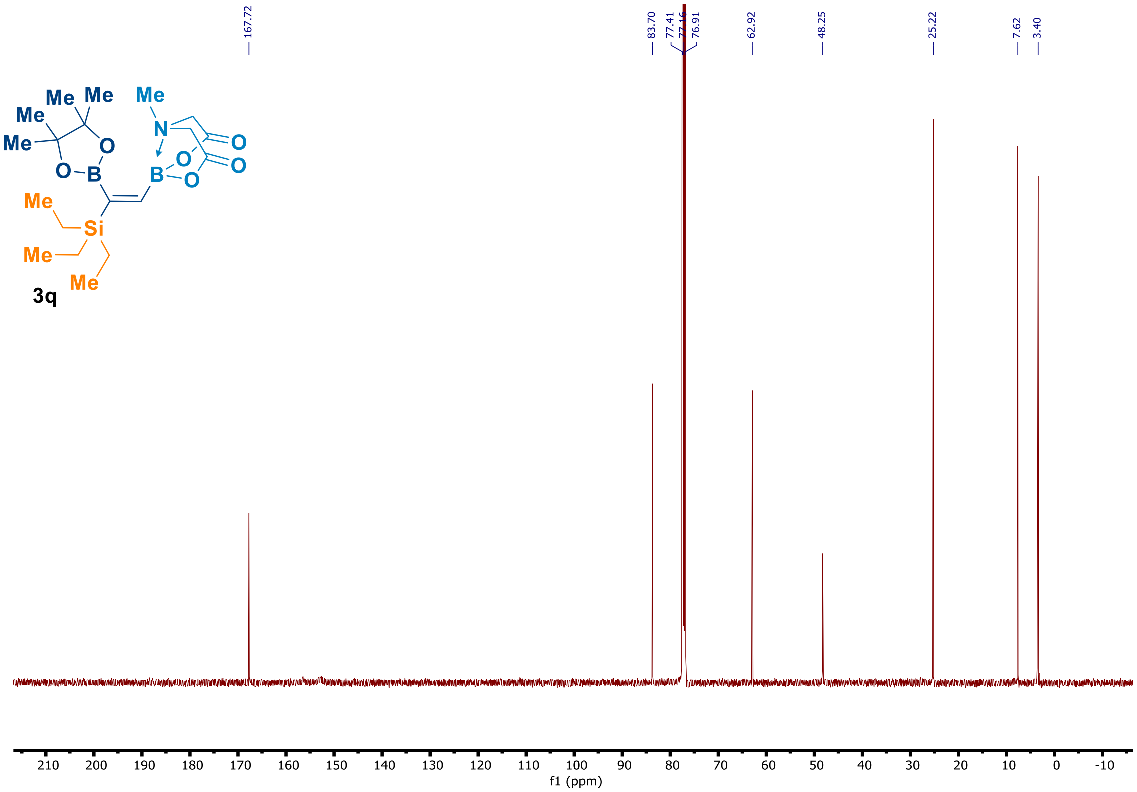

Supplementary Figure 211. ^{13}C NMR (101 MHz, $CDCl_3$) of compound (**3q**).

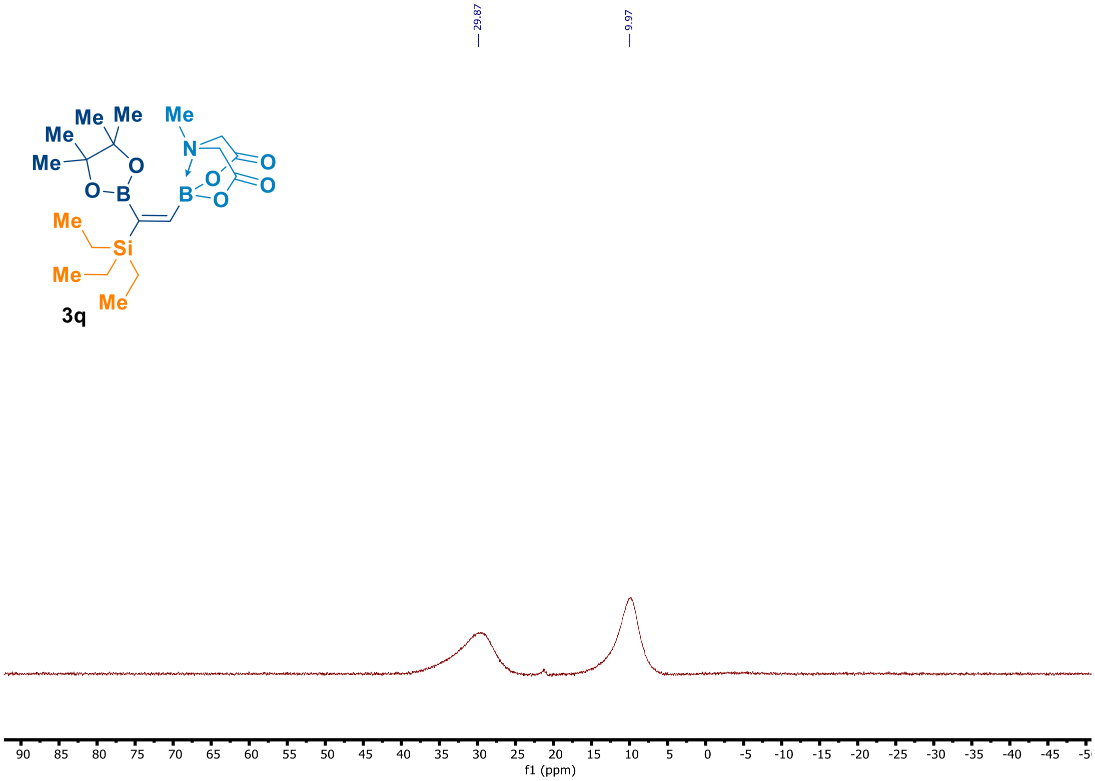

Supplementary Figure 212. ^{11}B NMR (128 MHz, $CDCl_3$) of compound (**3q**).

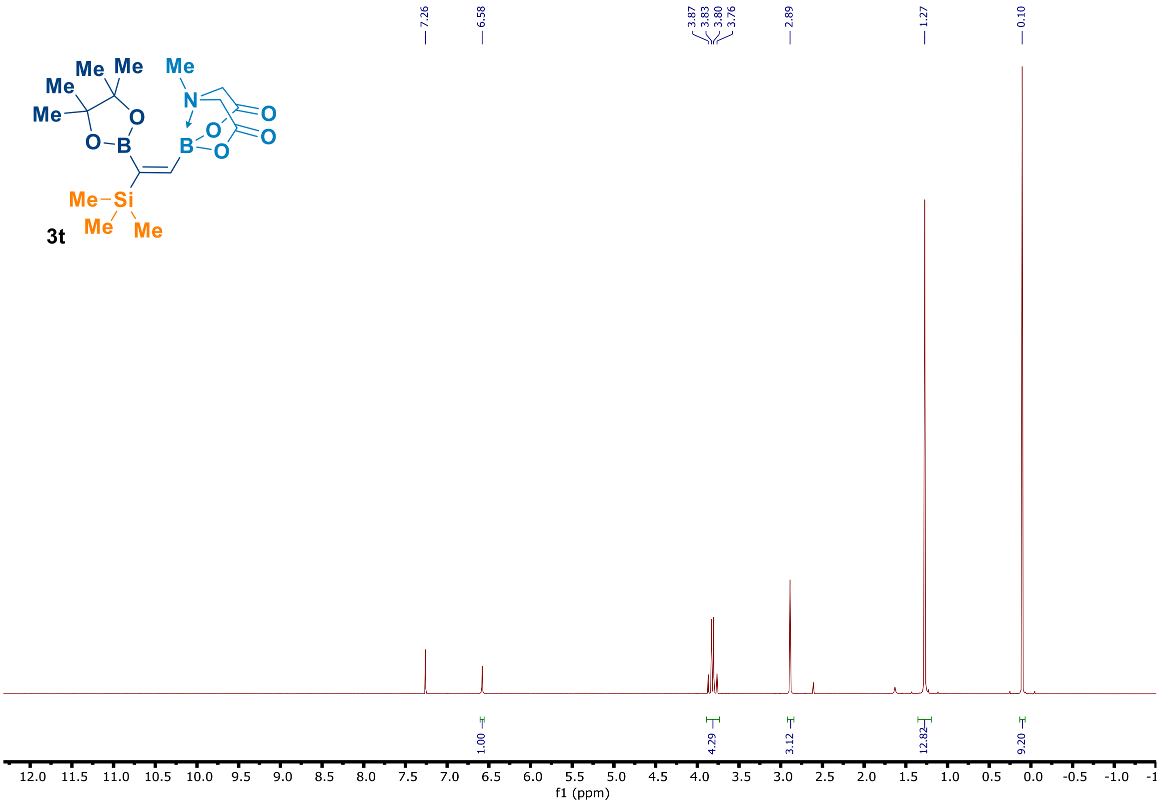

Supplementary Figure 213. ^{1}H NMR (400 MHz, $CDCl_3$) of compound (**3r**).

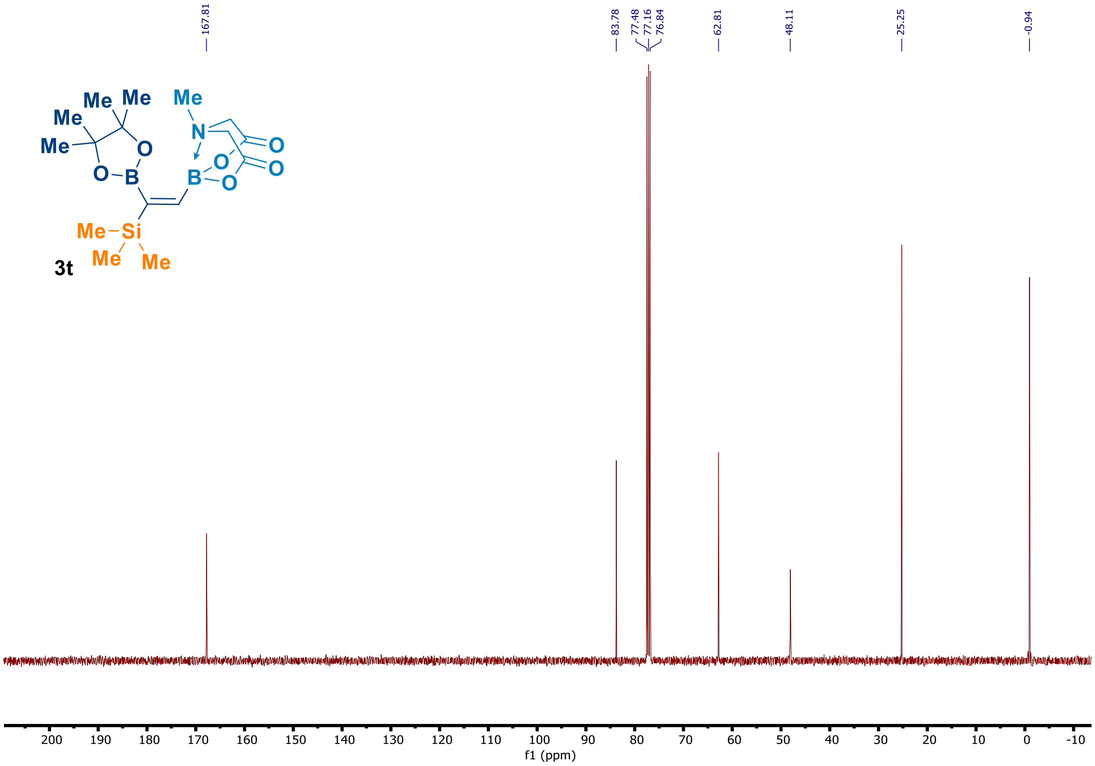

Supplementary Figure 214. ^{13}C NMR (101 MHz, $CDCl_3$) of compound (**3r**).

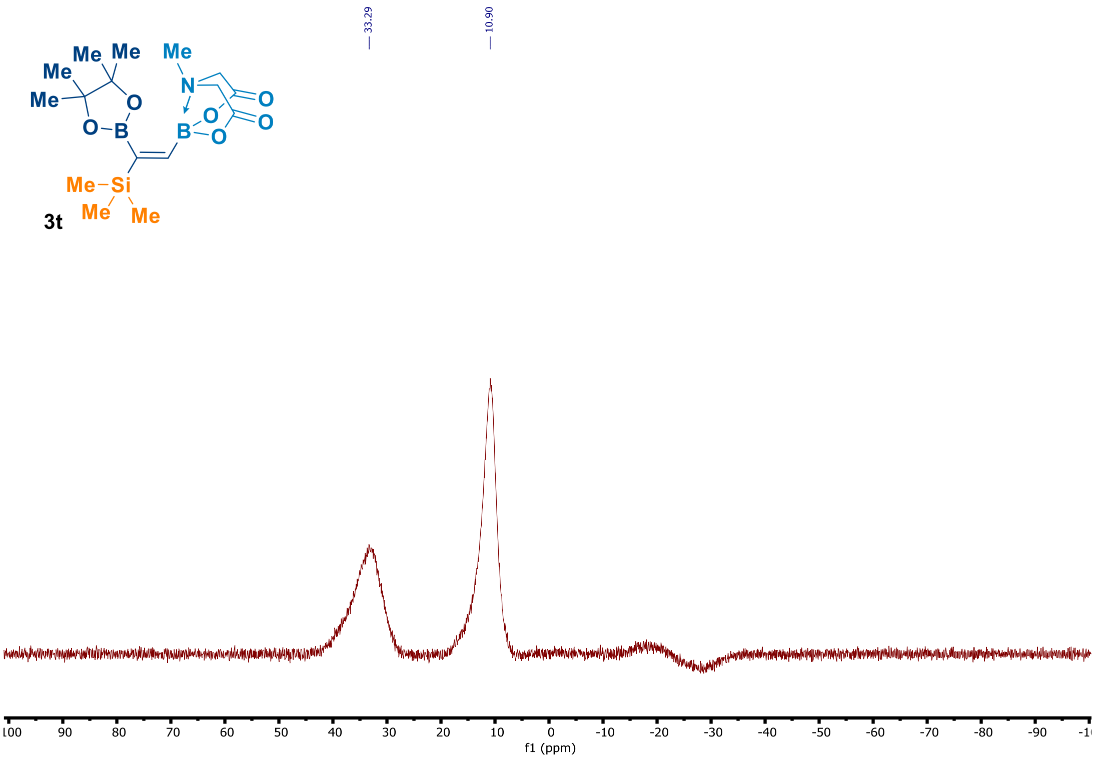

Supplementary Figure 215. ^{11}B NMR (128 MHz, $CDCl_3$) of compound (**3r**).

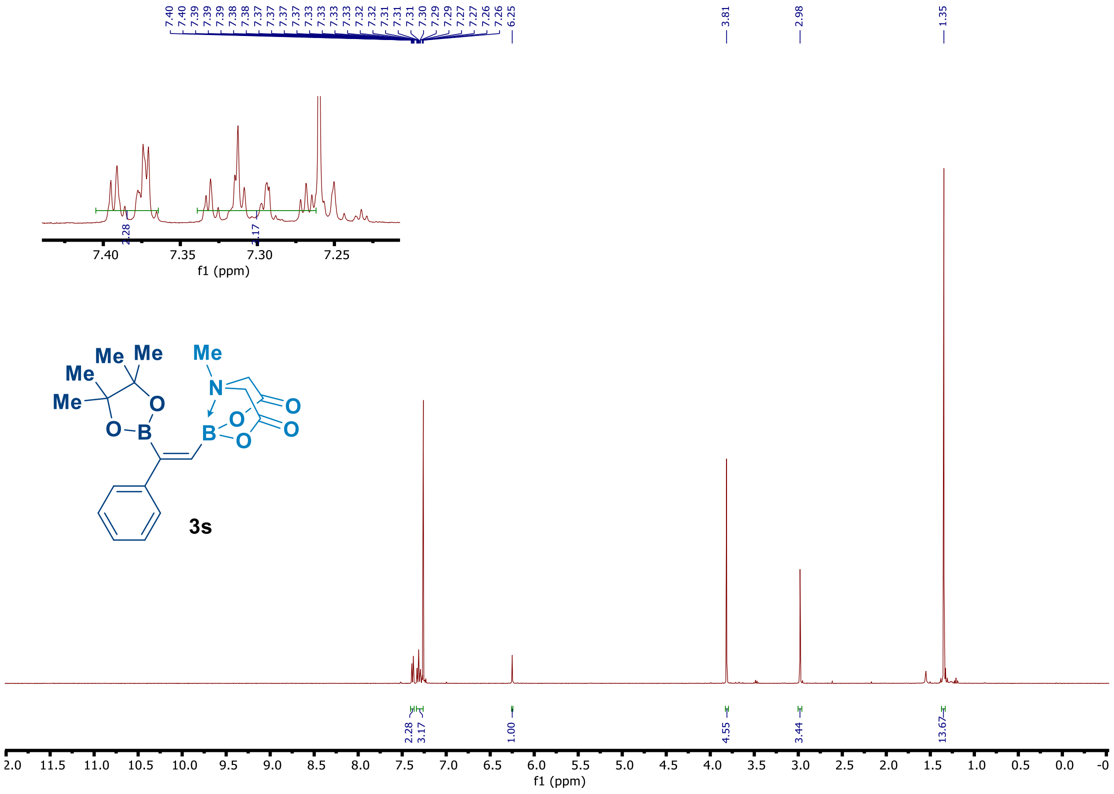

Supplementary Figure 216. ^{1}H NMR (400 MHz, $CDCl_3$) of compound (**3s**).

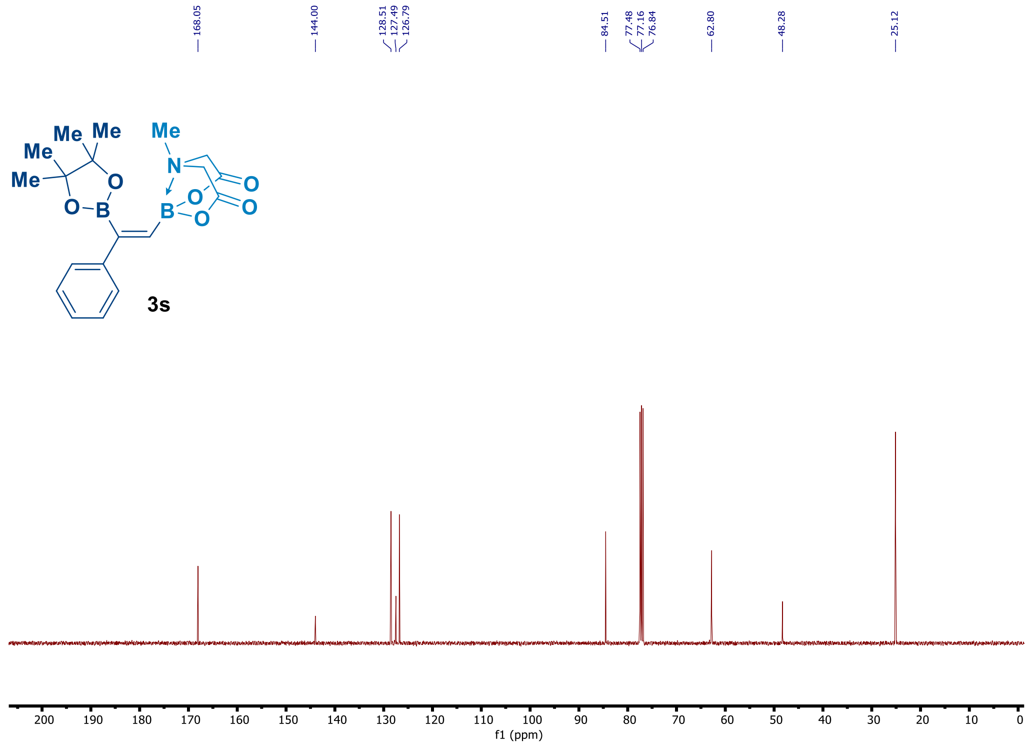

Supplementary Figure 217. ^{13}C NMR (101 MHz, $CDCl_3$) of compound (**3s**).

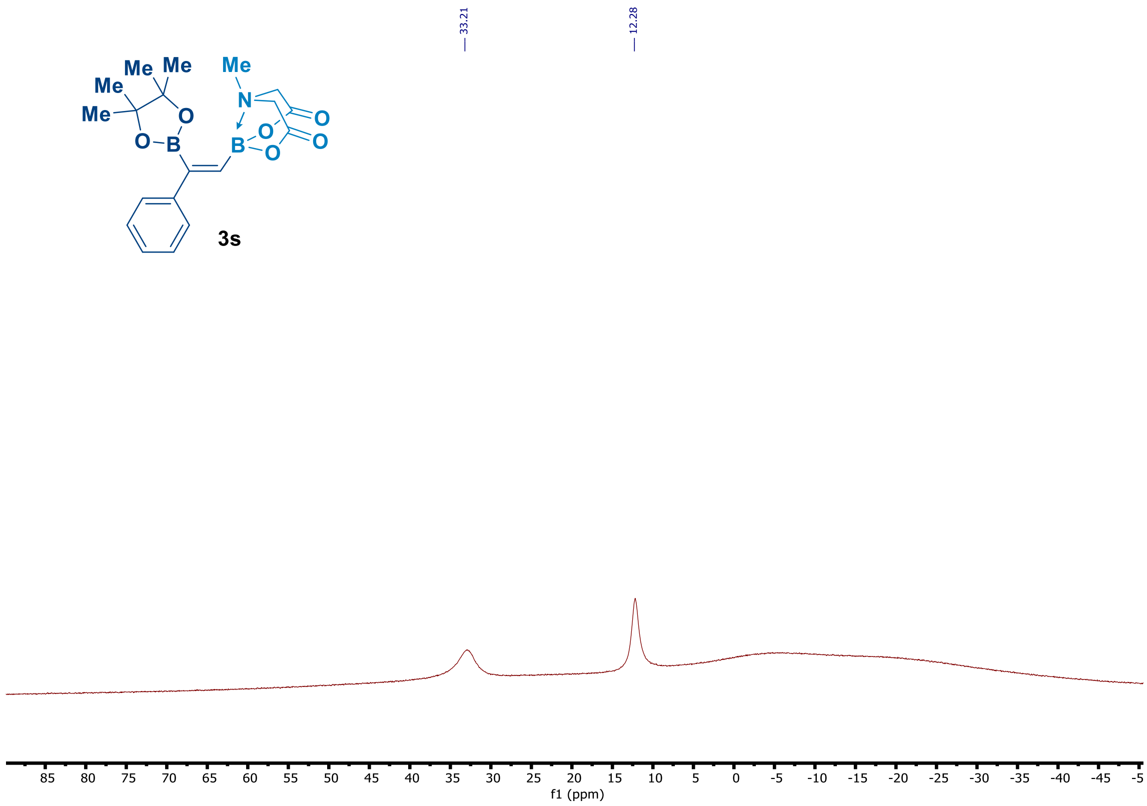

Supplementary Figure 218. ^{11}B NMR (128 MHz, $CDCl_3$) of compound (**3s**).

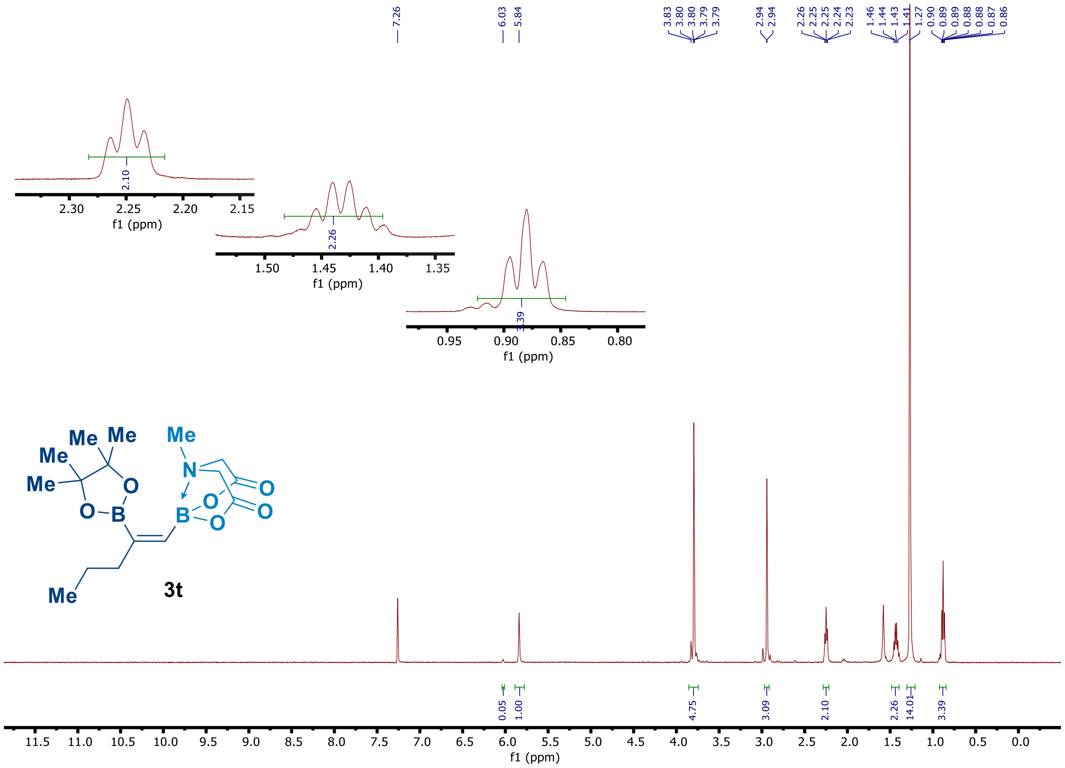

Supplementary Figure 219. ^{1}H NMR (400 MHz, $CDCl_3$) of compound (**3t**).

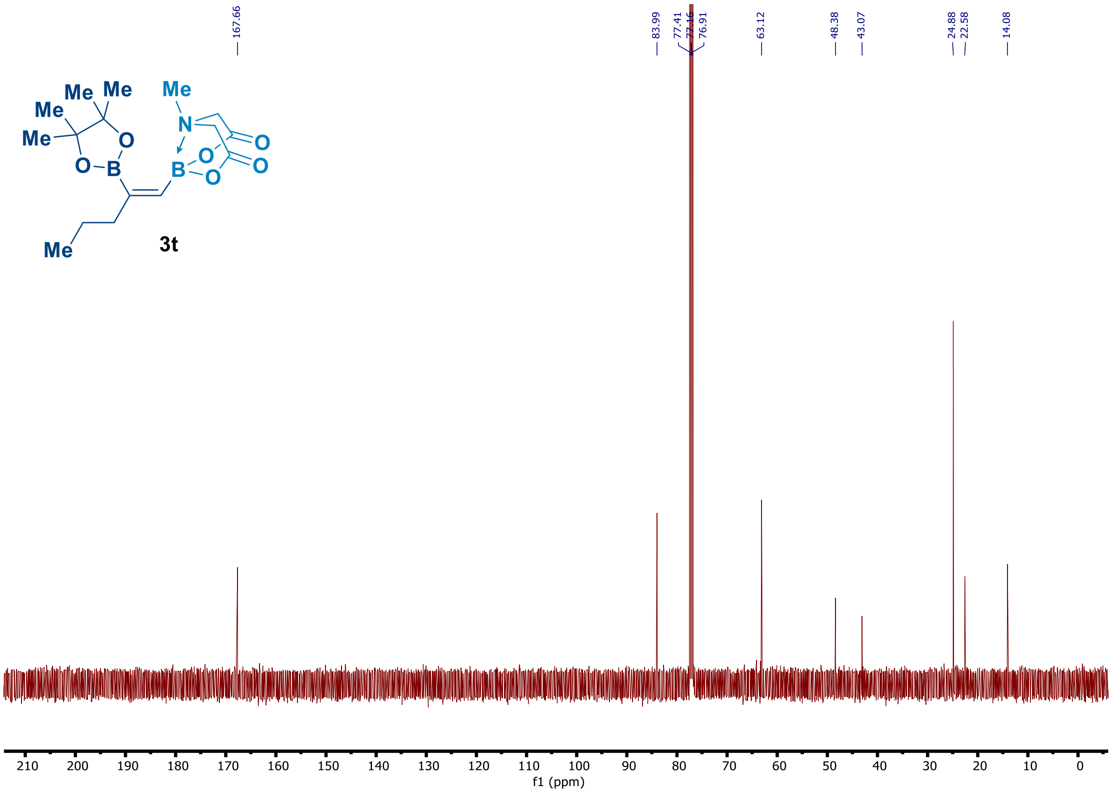

Supplementary Figure 220. ^{13}C NMR (101 MHz, $CDCl_3$) of compound (**3t**).

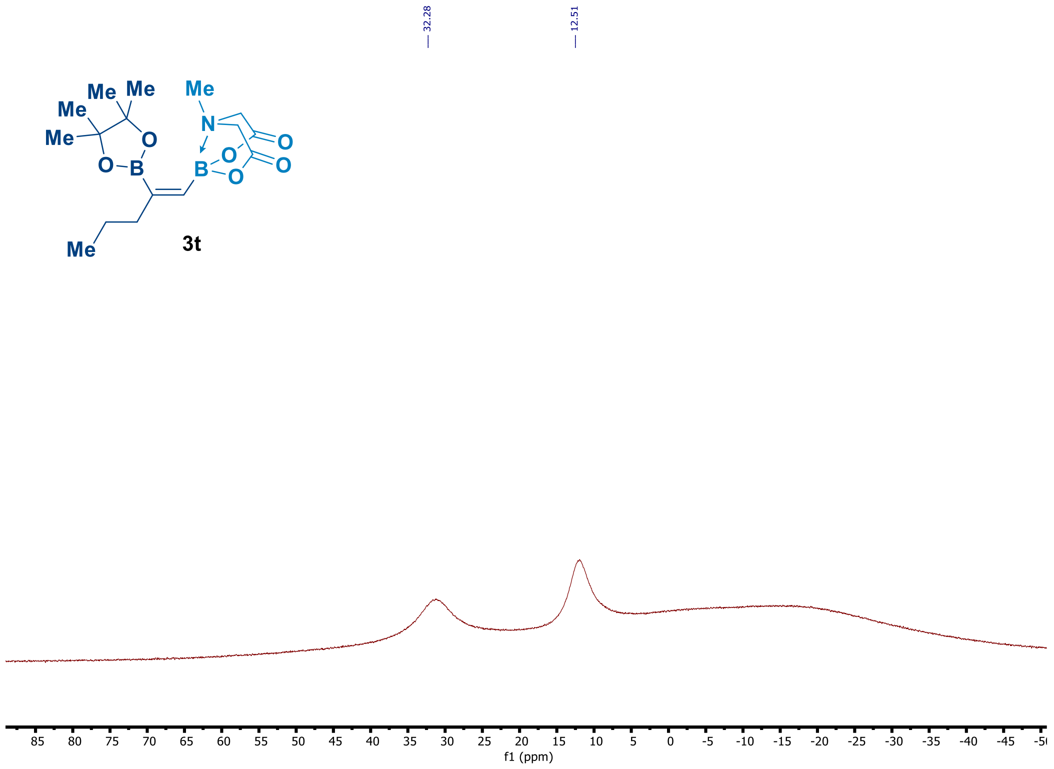

Supplementary Figure 221. ^{11}B NMR (128 MHz, $CDCl_3$) of compound (**3t**).

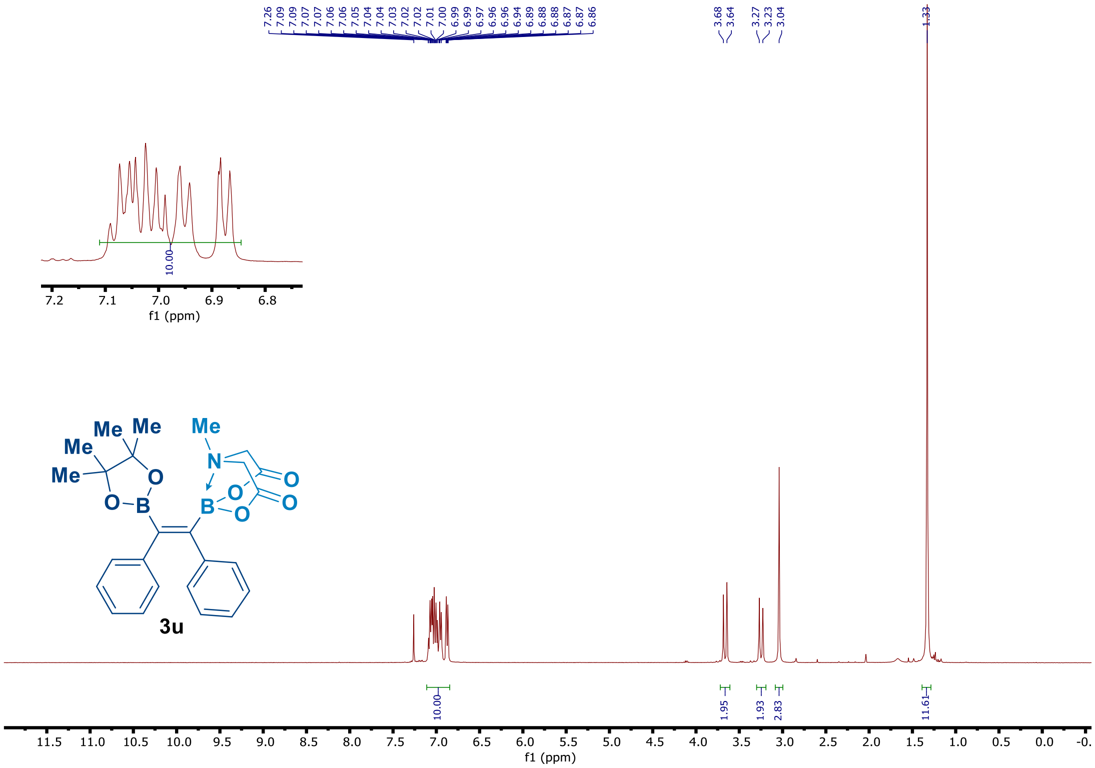

Supplementary Figure 222. ^{1}H NMR (400 MHz, $CDCl_3$) of compound (**3u**).

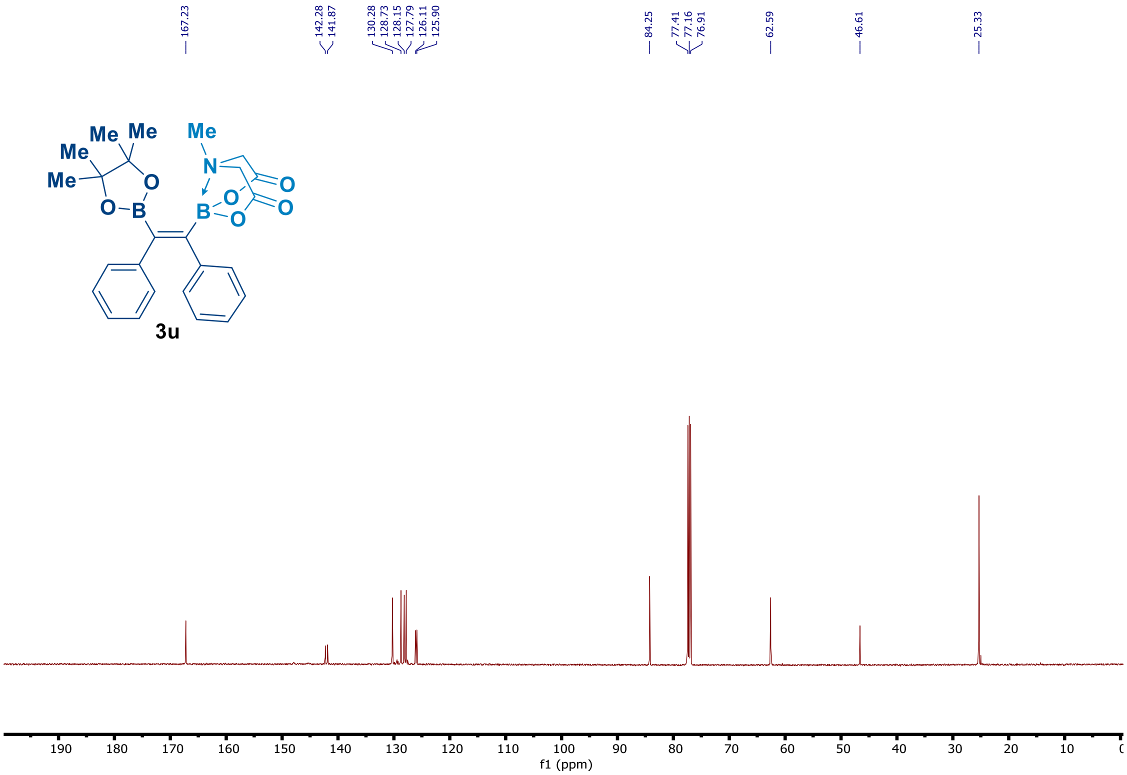

Supplementary Figure 223. ^{13}C NMR (101 MHz, $CDCl_3$) of compound (**3u**).

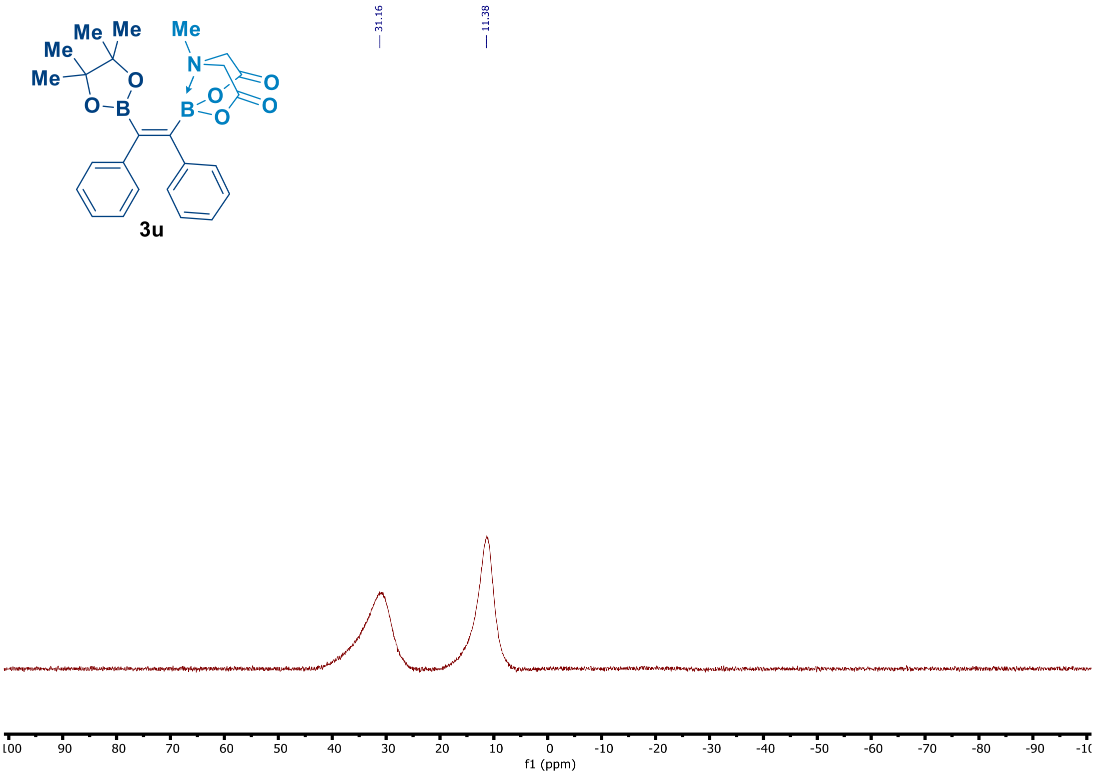

Supplementary Figure 224. ^{11}B NMR (128 MHz, $CDCl_3$) of compound (**3u**).

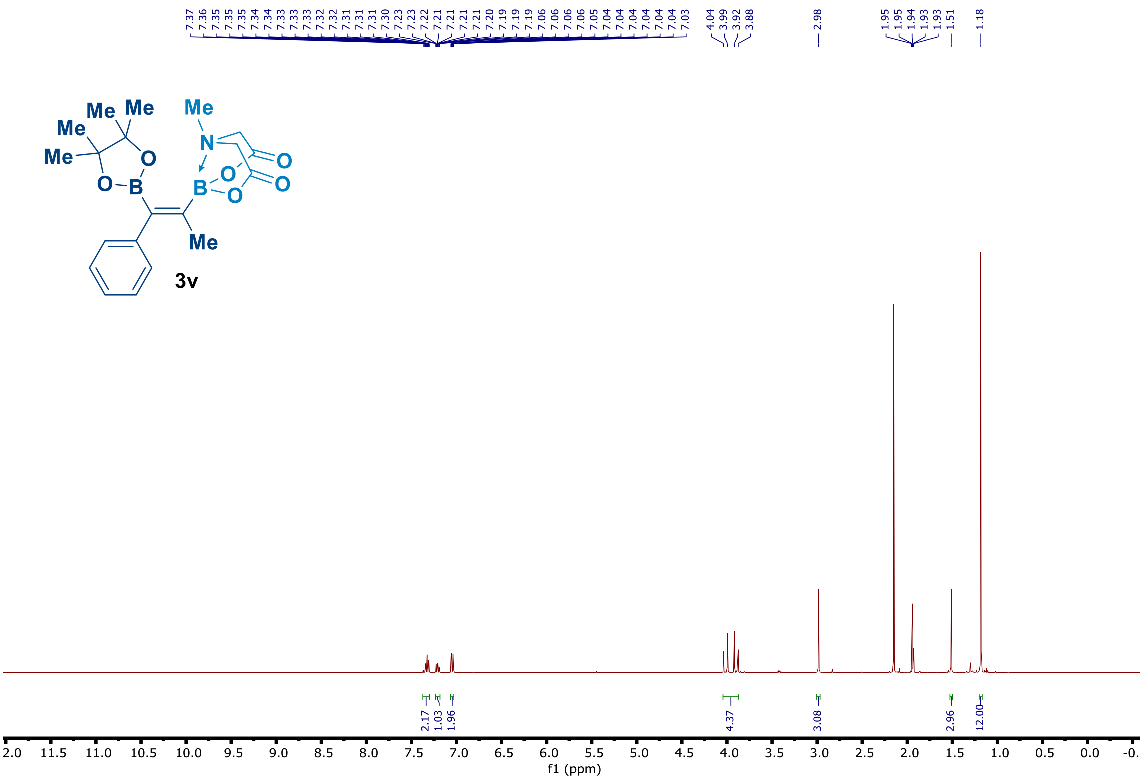

Supplementary Figure 225. ^{1}H NMR (400 MHz, CD_3CN) of compound (**3v**).

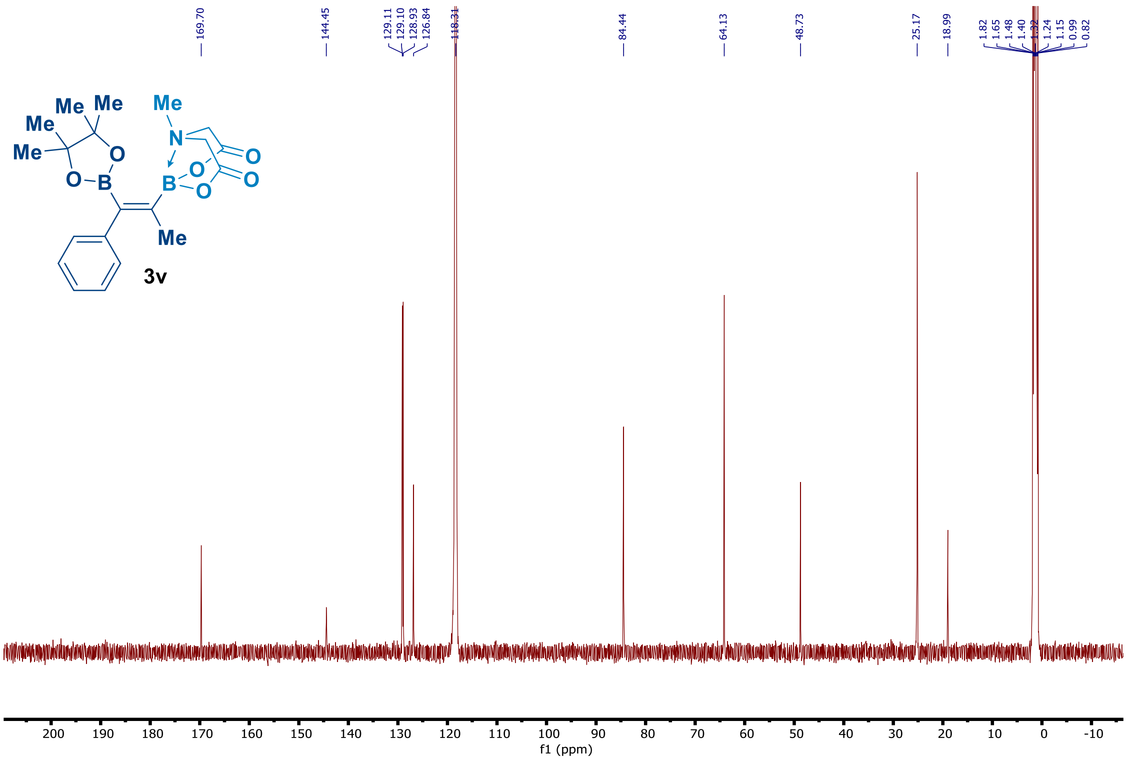

Supplementary Figure 226. ^{13}C NMR (101 MHz, CD_3CN) of compound (**3v**).

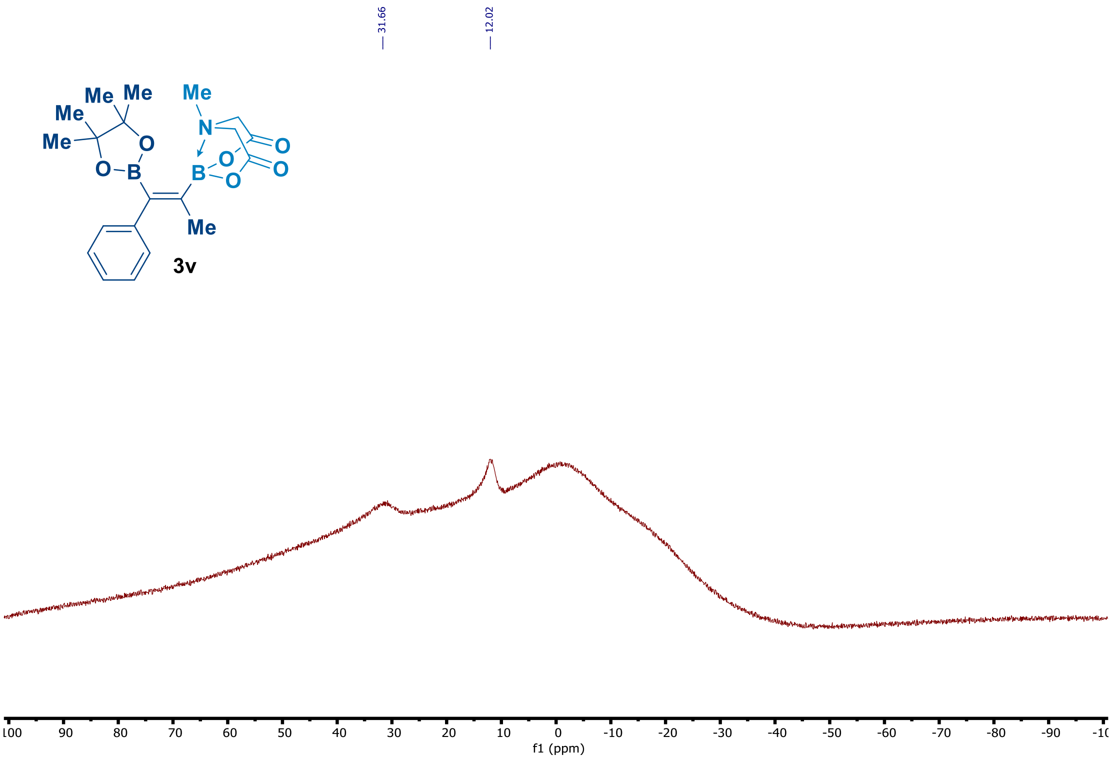

Supplementary Figure 227. ^{11}B NMR (128 MHz, CD_3CN) of compound (**3v**).

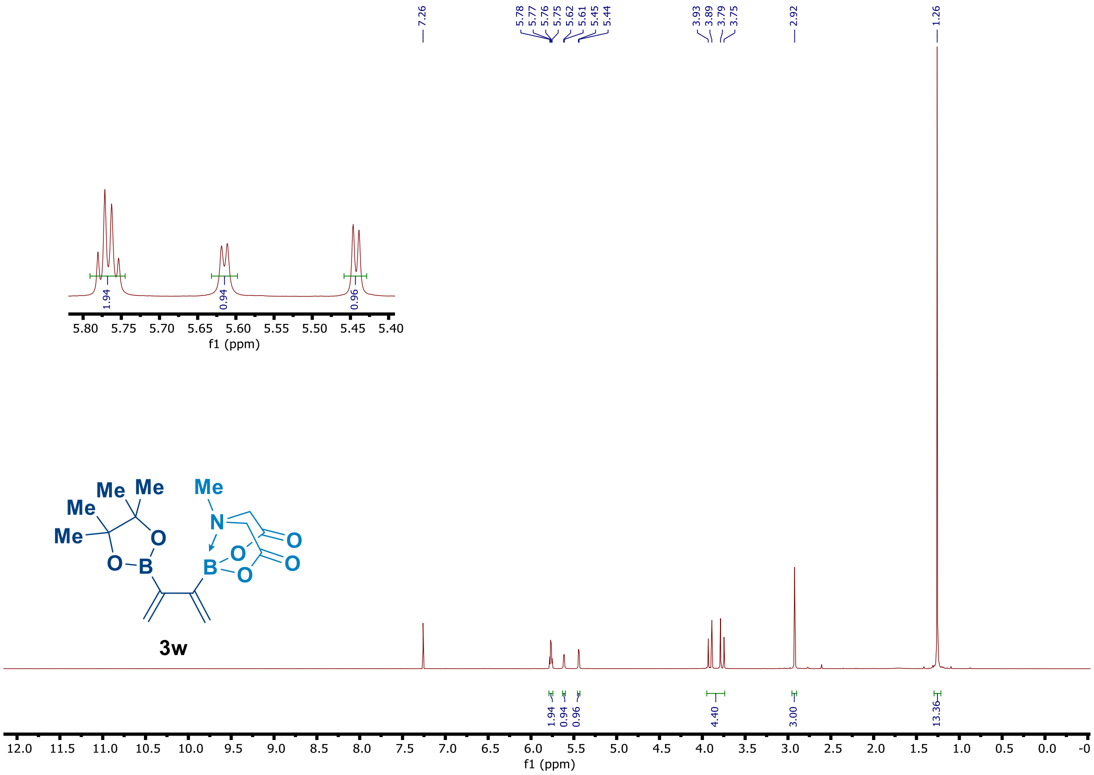

Supplementary Figure 228. ^{1}H NMR (400 MHz, $CDCl_3$) of compound (**3w**).

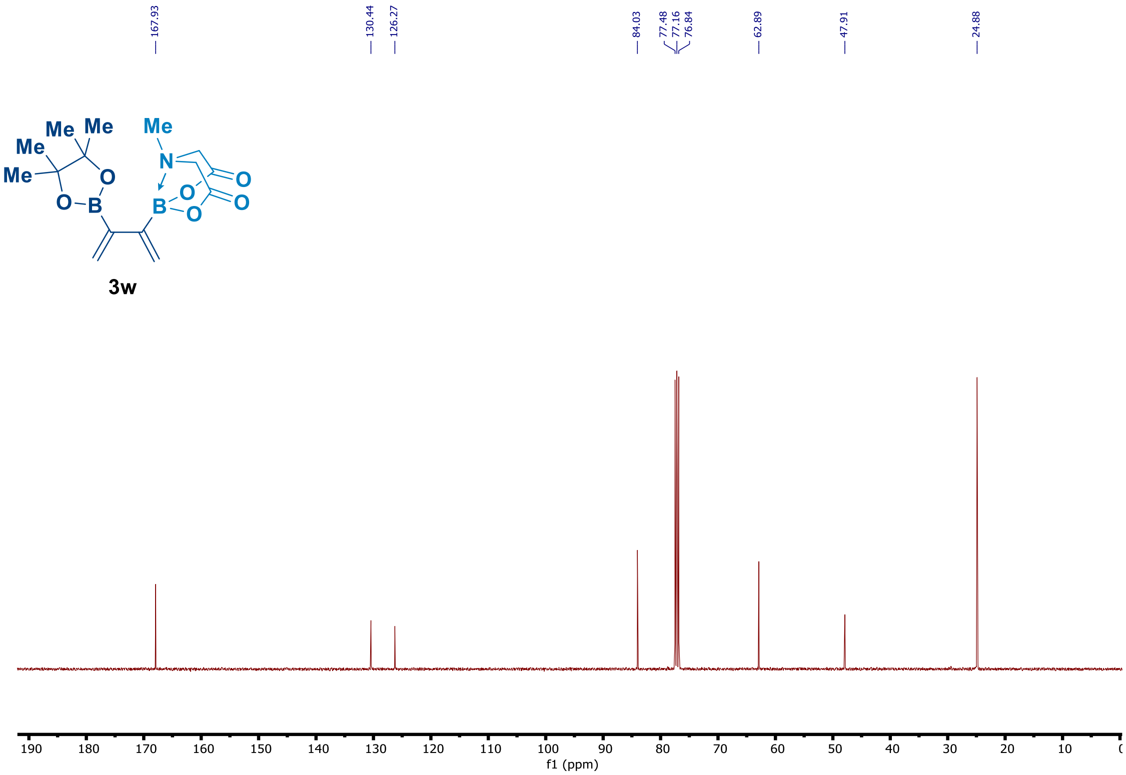

Supplementary Figure 229. ^{13}C NMR (101 MHz, $CDCl_3$) of compound (**3w**).

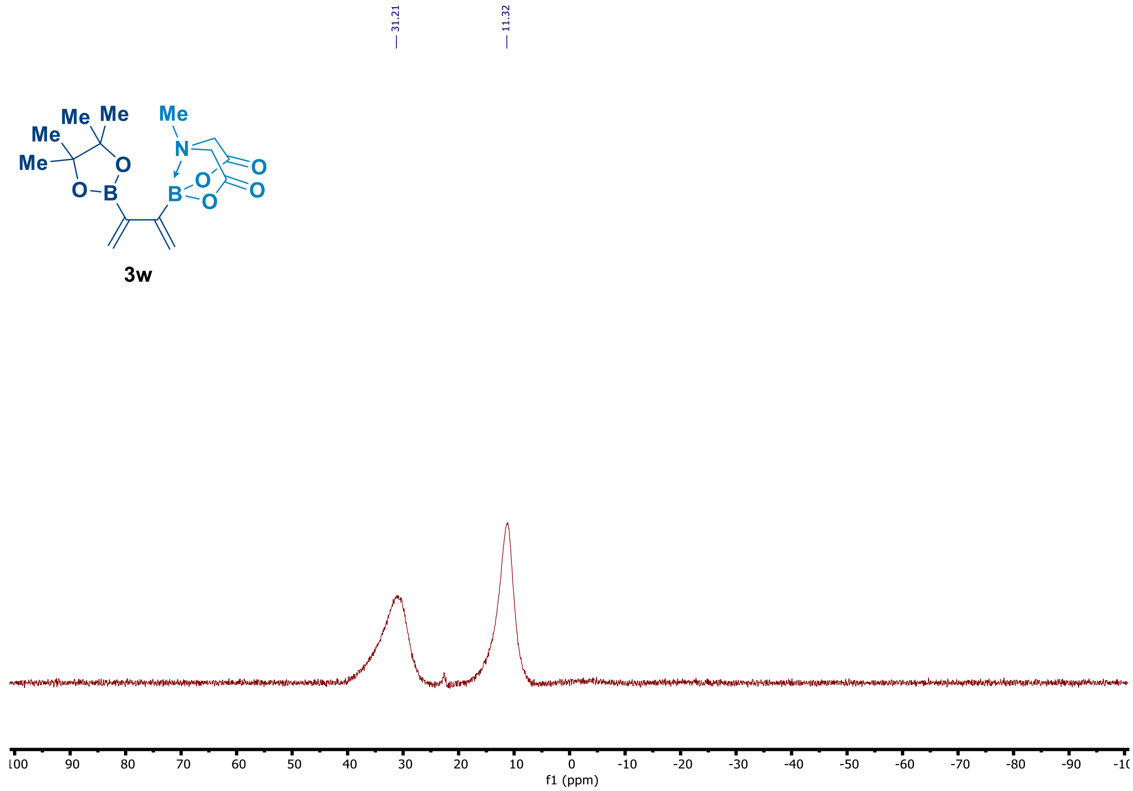

Supplementary Figure 230. ^{11}B NMR (128 MHz, $CDCl_3$) of compound (**3w**).

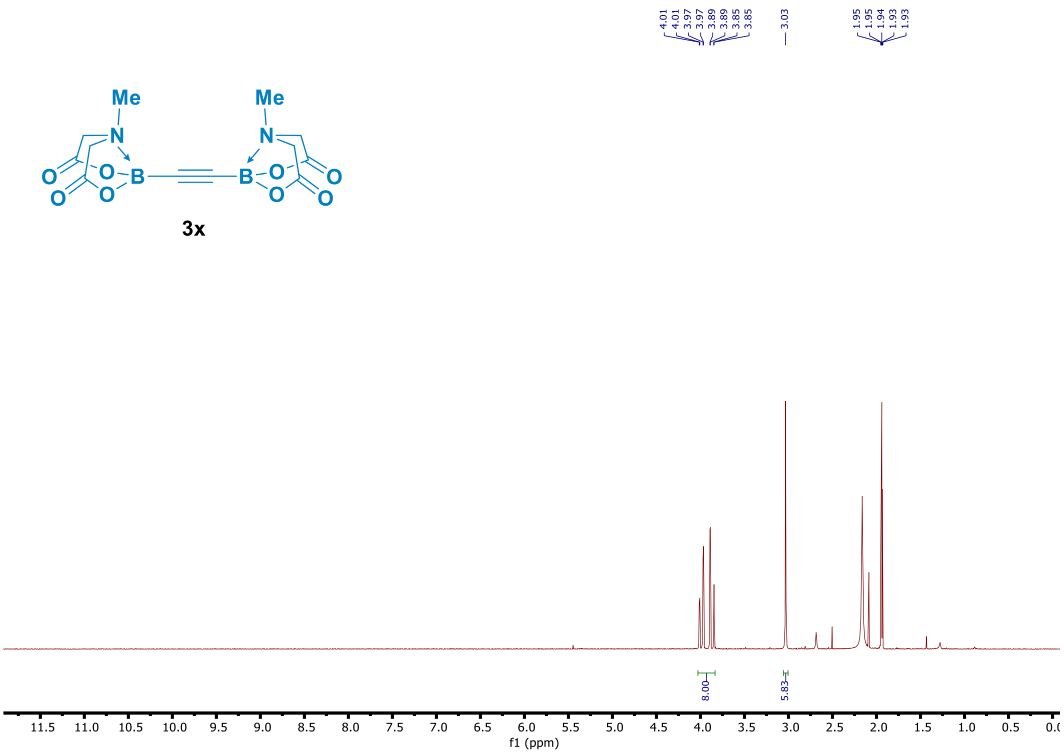

Supplementary Figure 231. ^{1}H NMR (400 MHz, CD_3CN) of compound (**3x**).

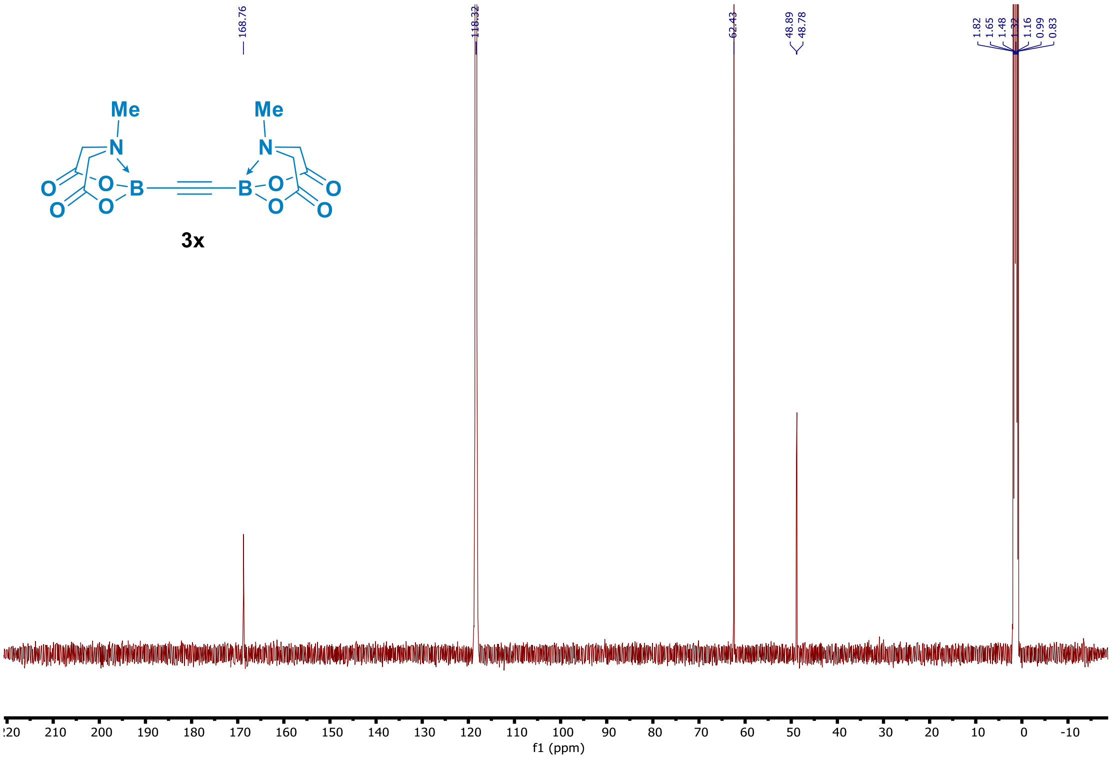

Supplementary Figure 232. ^{13}C NMR (101 MHz, CD_3CN) of compound (**3x**).

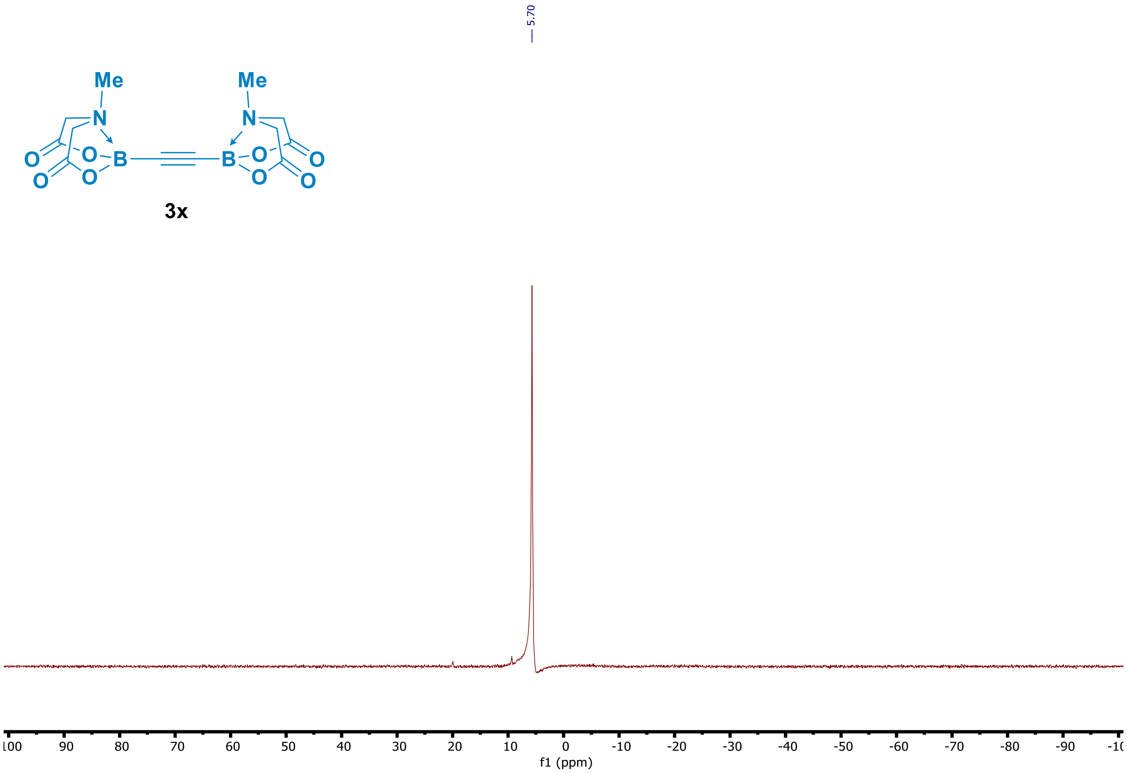

Supplementary Figure 233. ^{11}B NMR (128 MHz, CD_3CN) of compound (**3x**).

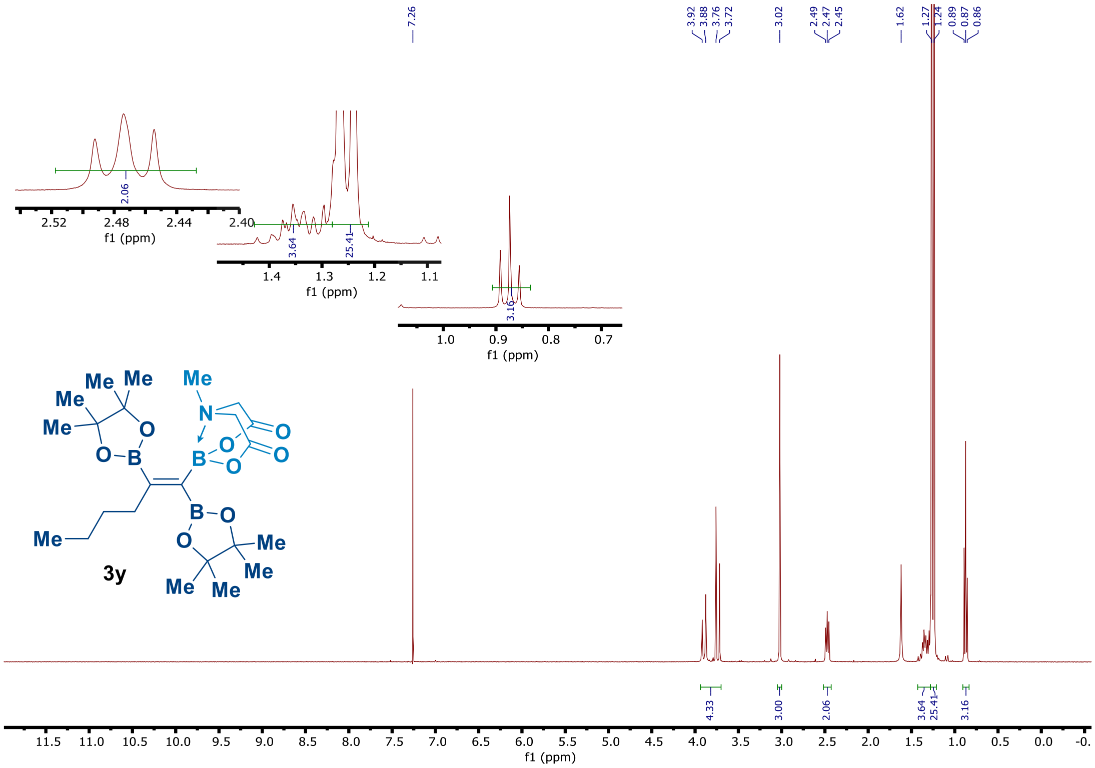

Supplementary Figure 234. ^{1}H NMR (400 MHz, $CDCl_3$) of compound (**3y**).

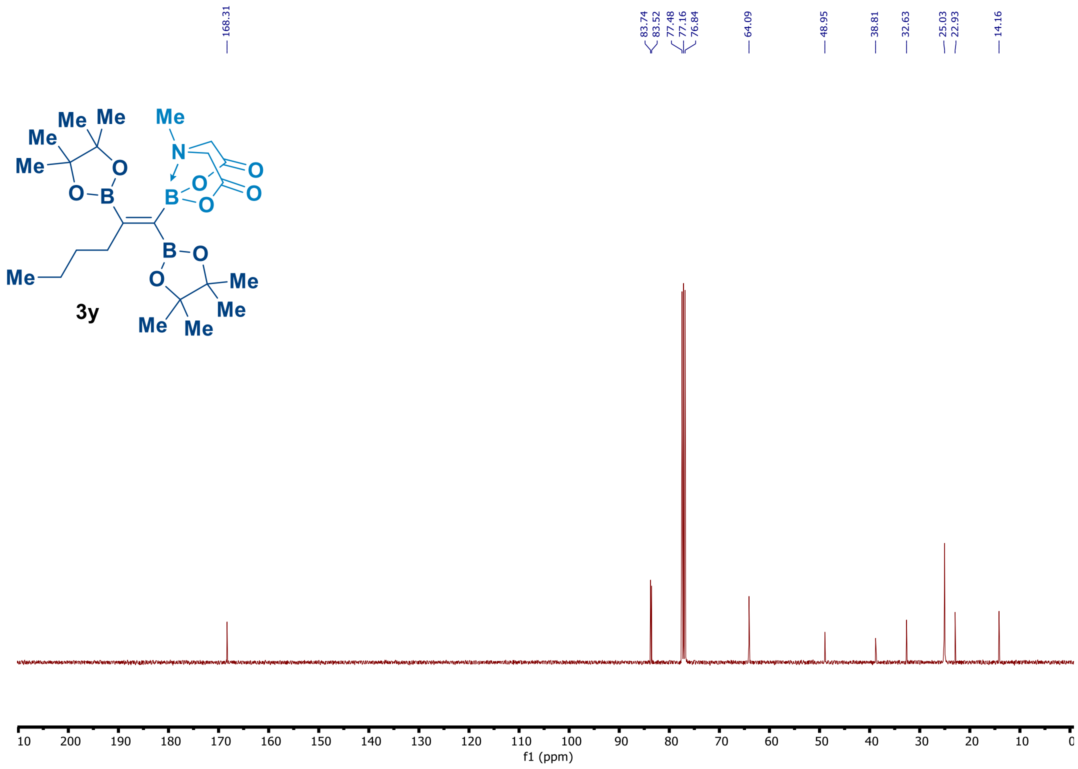

Supplementary Figure 235. ^{13}C NMR (101 MHz, $CDCl_3$) of compound (**3y**).

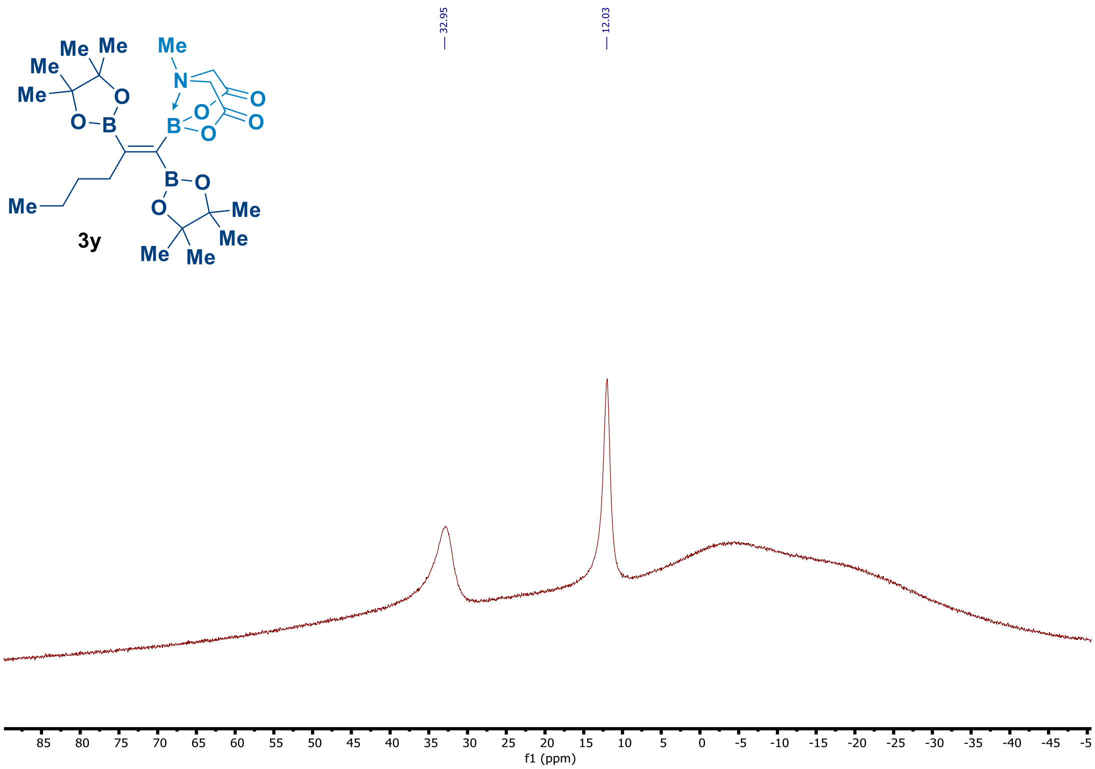

Supplementary Figure 236. ^{11}B NMR (128 MHz, $CDCl_3$) of compound (**3y**).

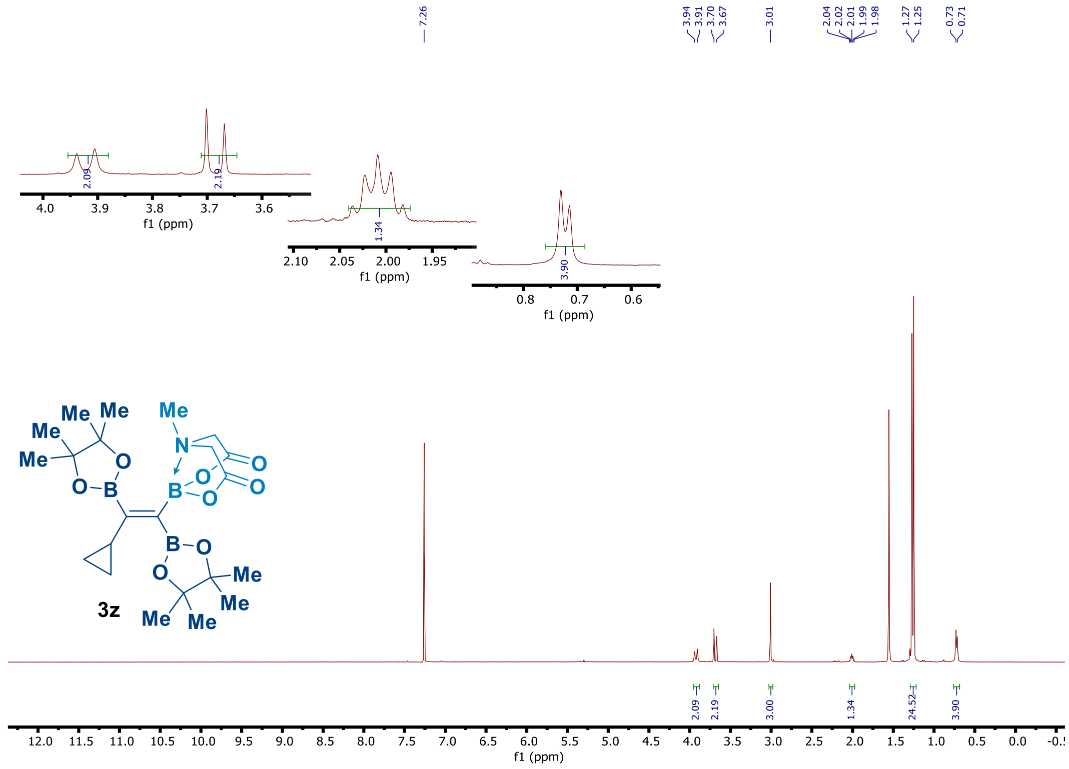

Supplementary Figure 237. ^{1}H NMR (400 MHz, $CDCl_3$) of compound (**3z**).

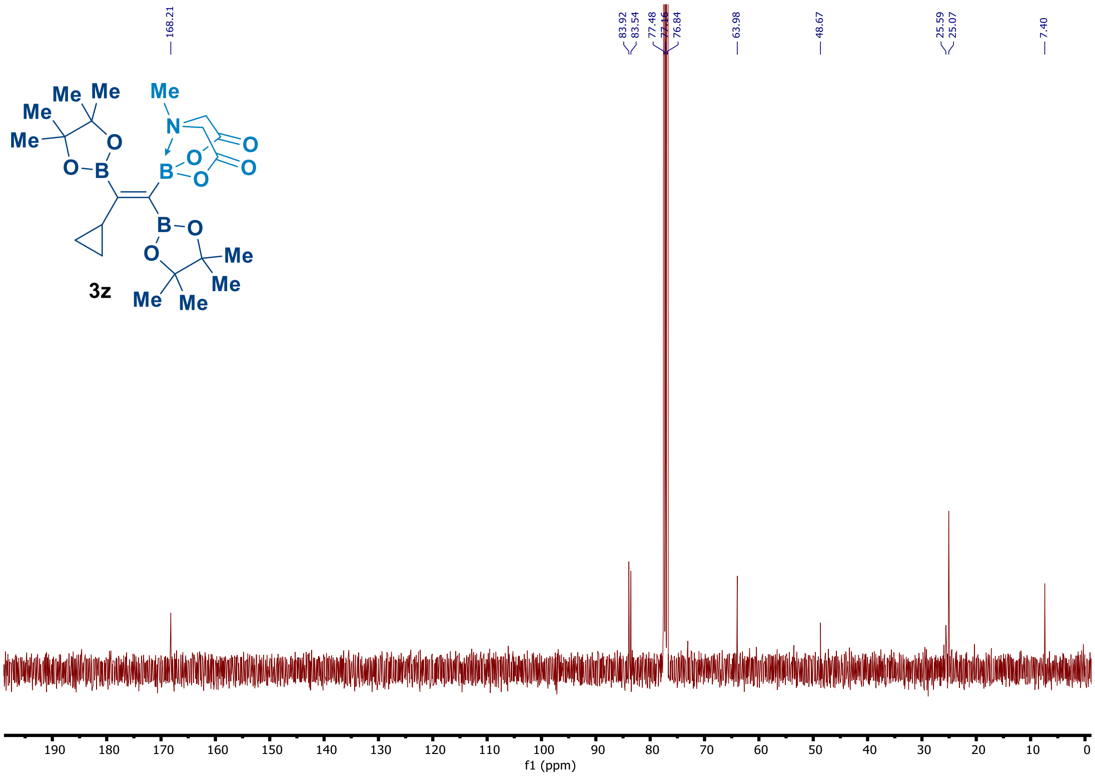

Supplementary Figure 238. ^{13}C NMR (101 MHz, $CDCl_3$) of compound (**3z**).

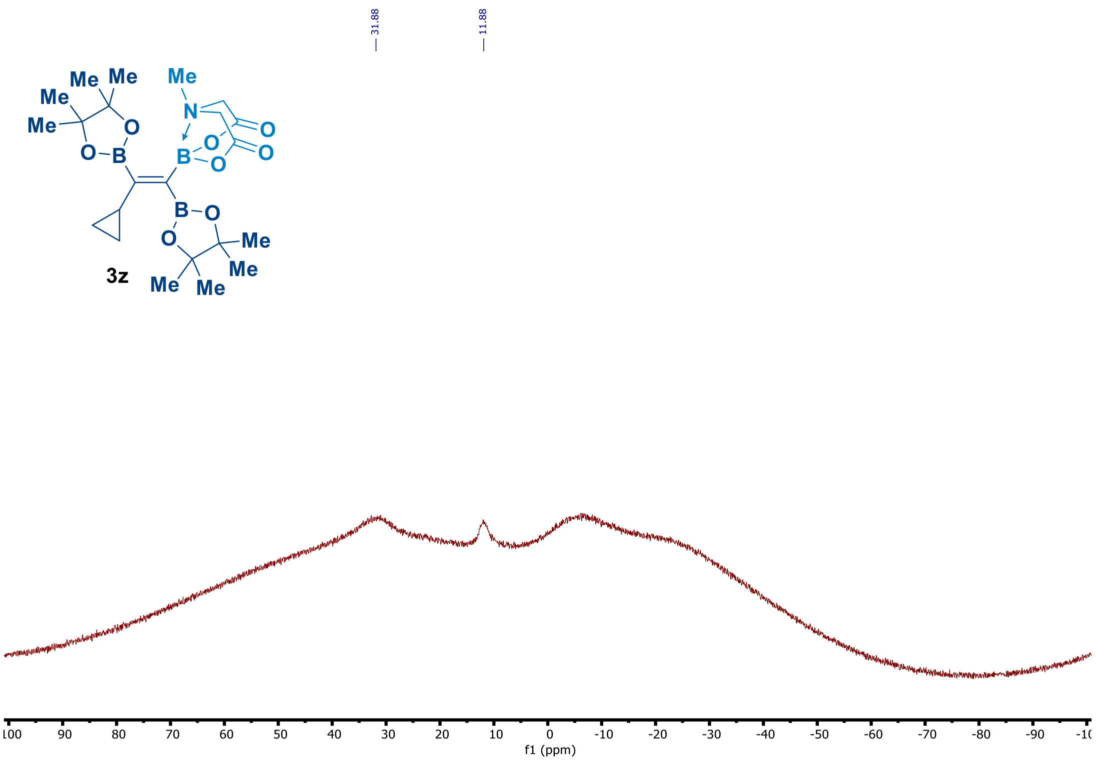

Supplementary Figure 239. ^{11}B NMR (128 MHz, $CDCl_3$) of compound (**3z**).

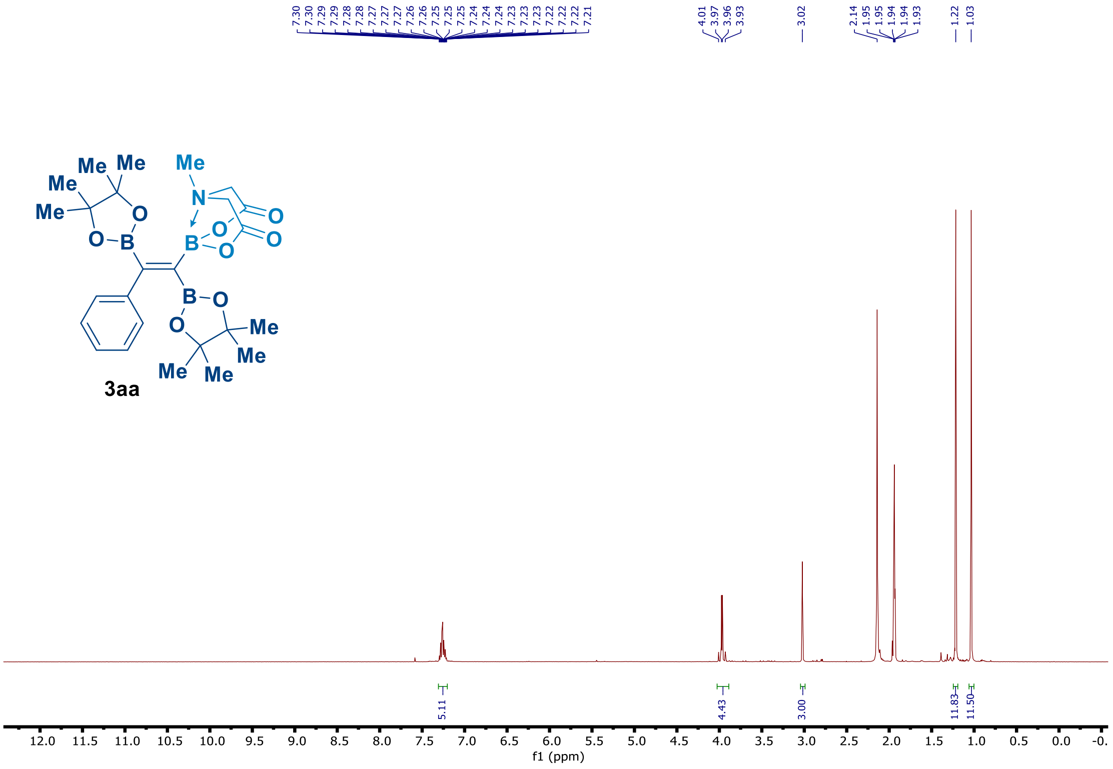

Supplementary Figure 240. ^{1}H NMR (400 MHz, CD_3CN) of compound (**3aa**).

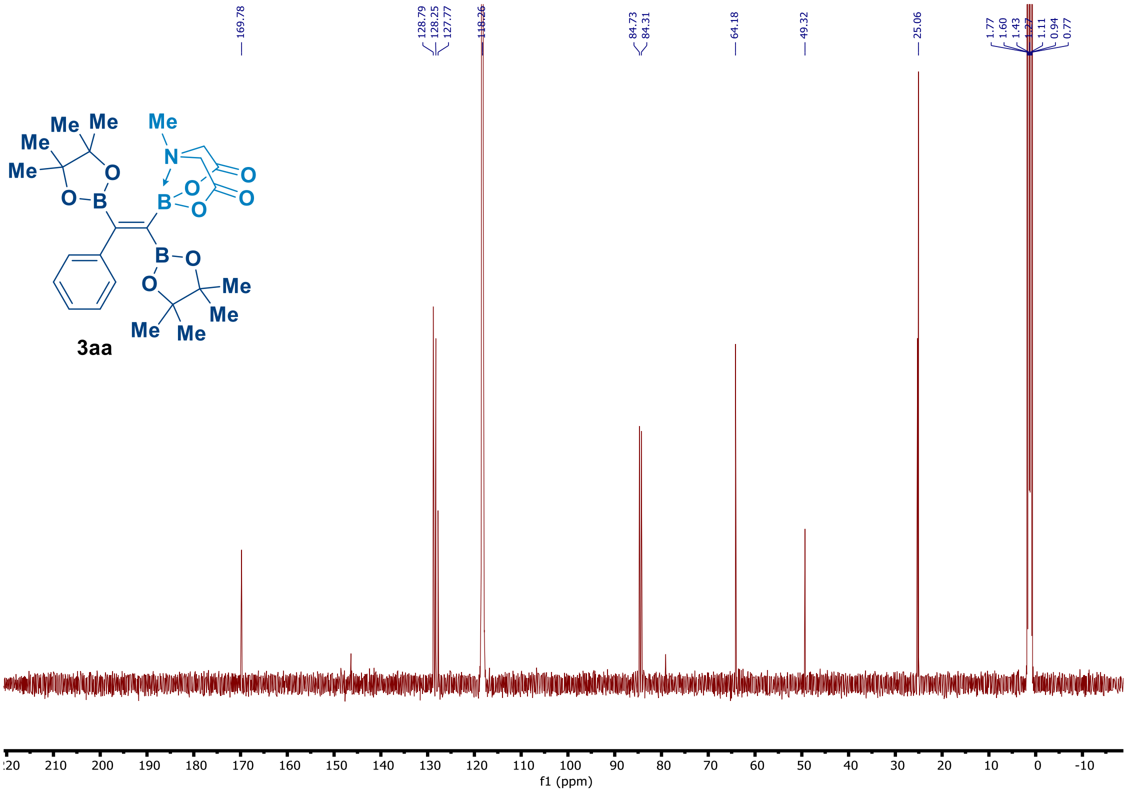

Supplementary Figure 241. ^{13}C NMR (101 MHz, $CDCl_3$) of compound (**3aa**).

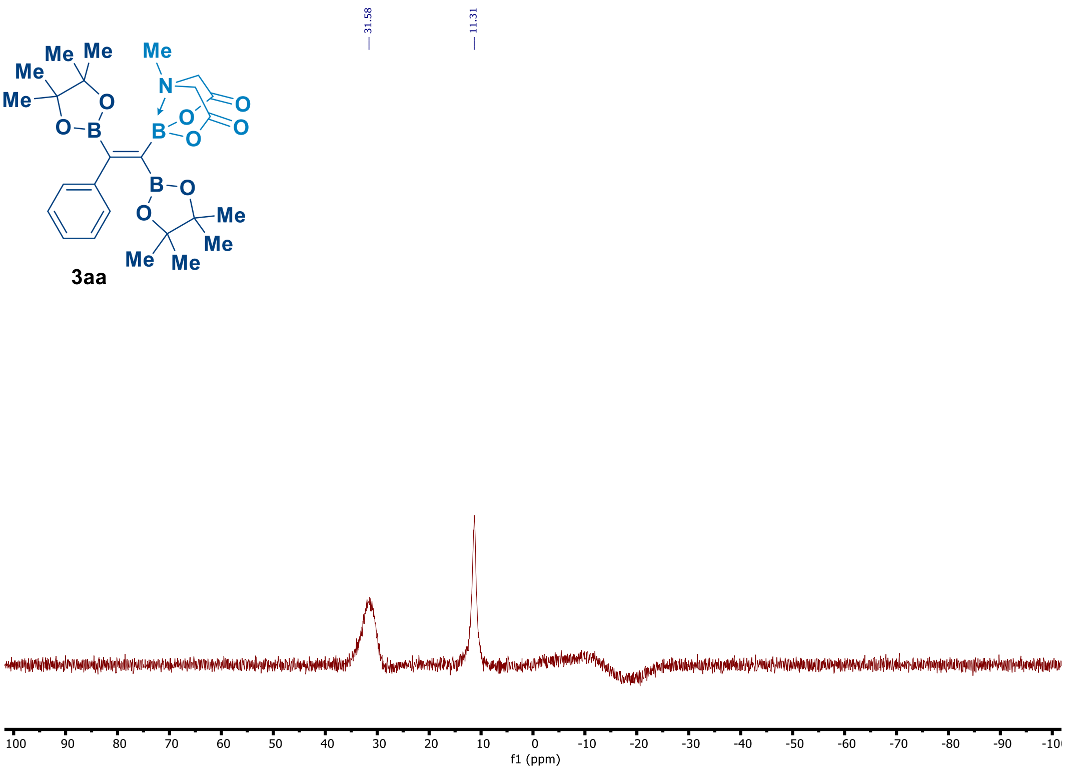

Supplementary Figure 242. ^{11}B NMR (128 MHz, $CDCl_3$) of compound (**3aa**).

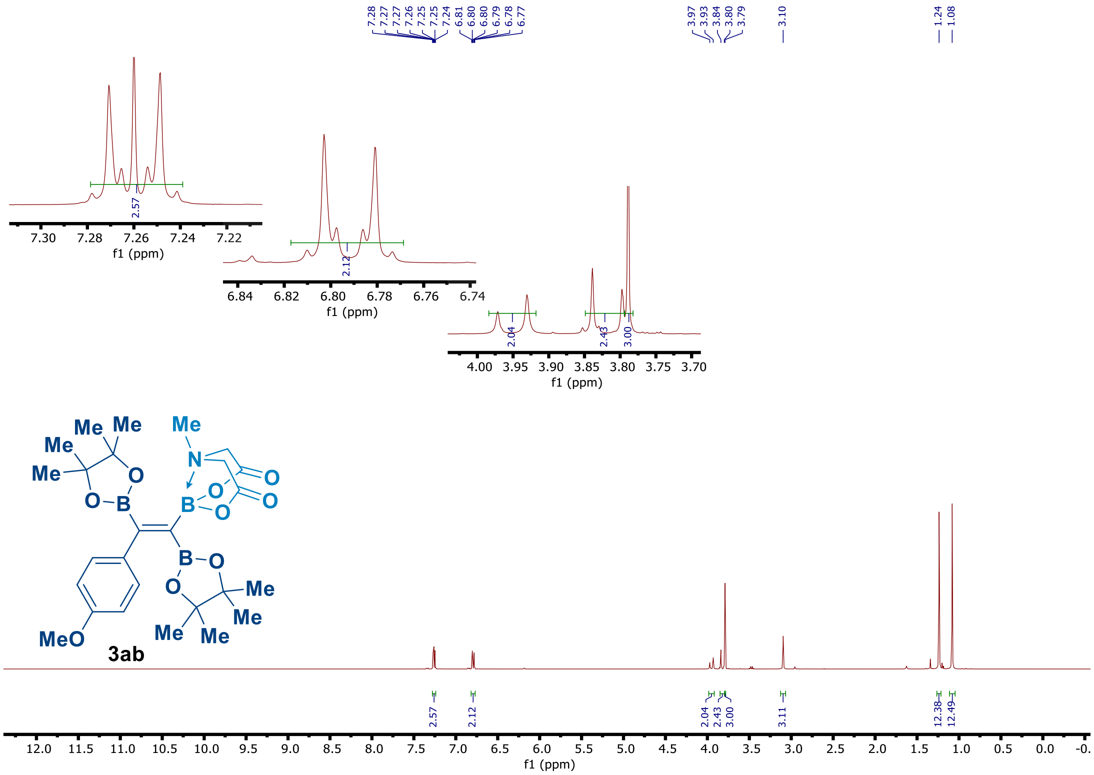

Supplementary Figure 243. ^{1}H NMR (400 MHz, $CDCl_3$) of compound (**3ab**).

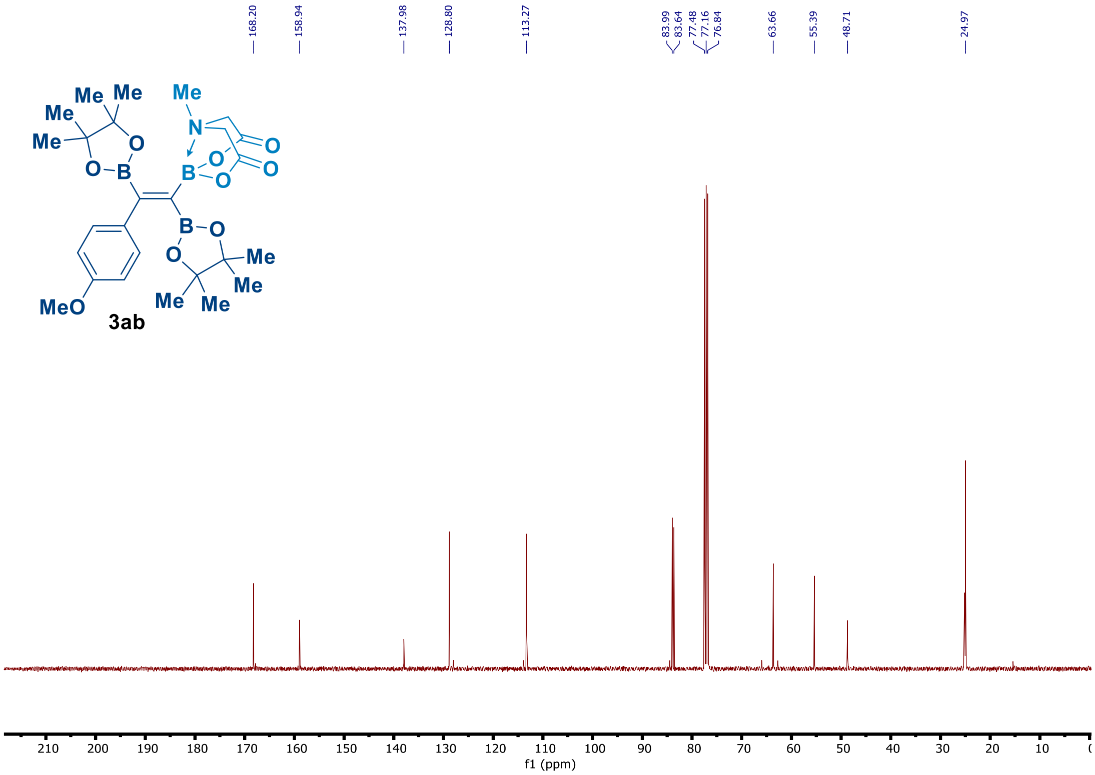

Supplementary Figure 244. ^{13}C NMR (101 MHz, $CDCl_3$) of compound (**3ab**).

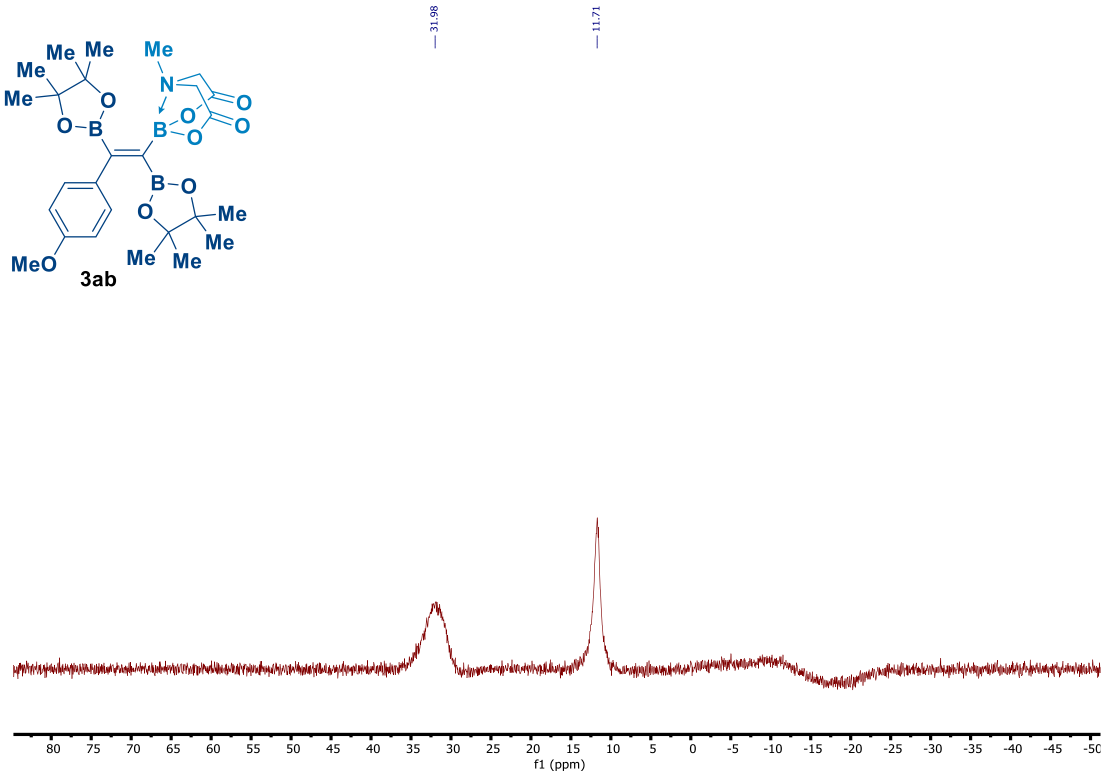

Supplementary Figure 245. ^{11}B NMR (128 MHz, $CDCl_3$) of compound (**3ab**).

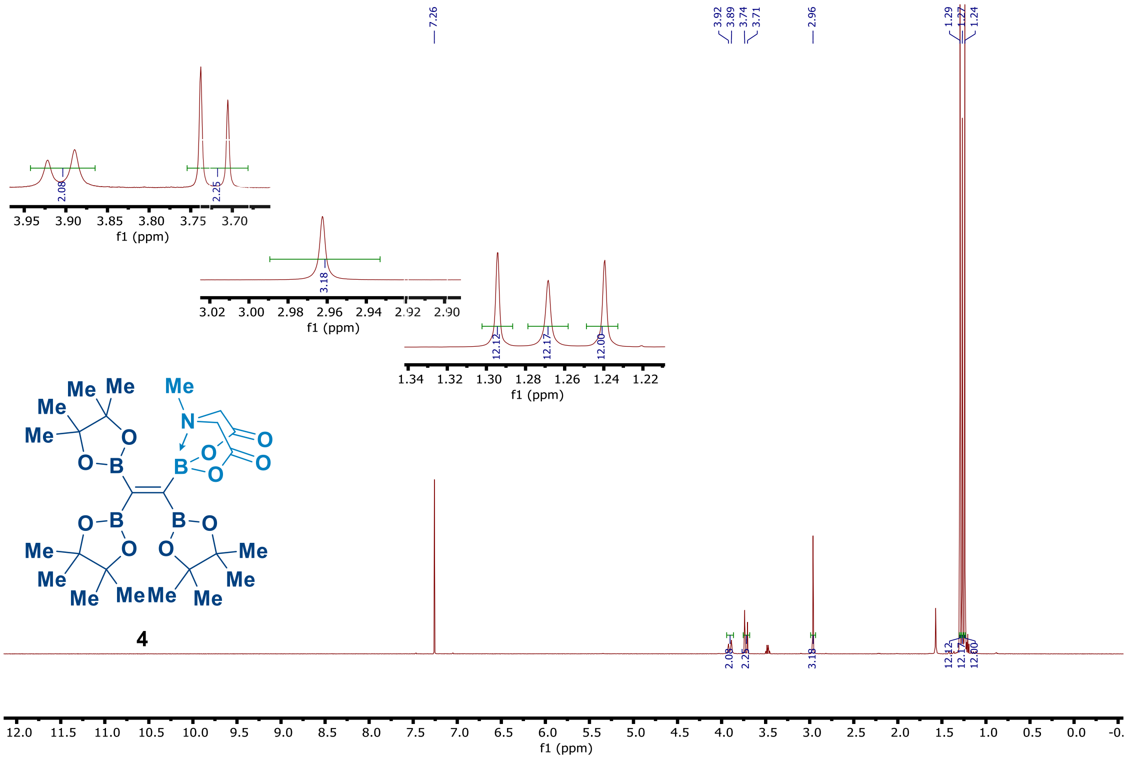

Supplementary Figure 246. ^{1}H NMR (400 MHz, $CDCl_3$) of compound (**4**).

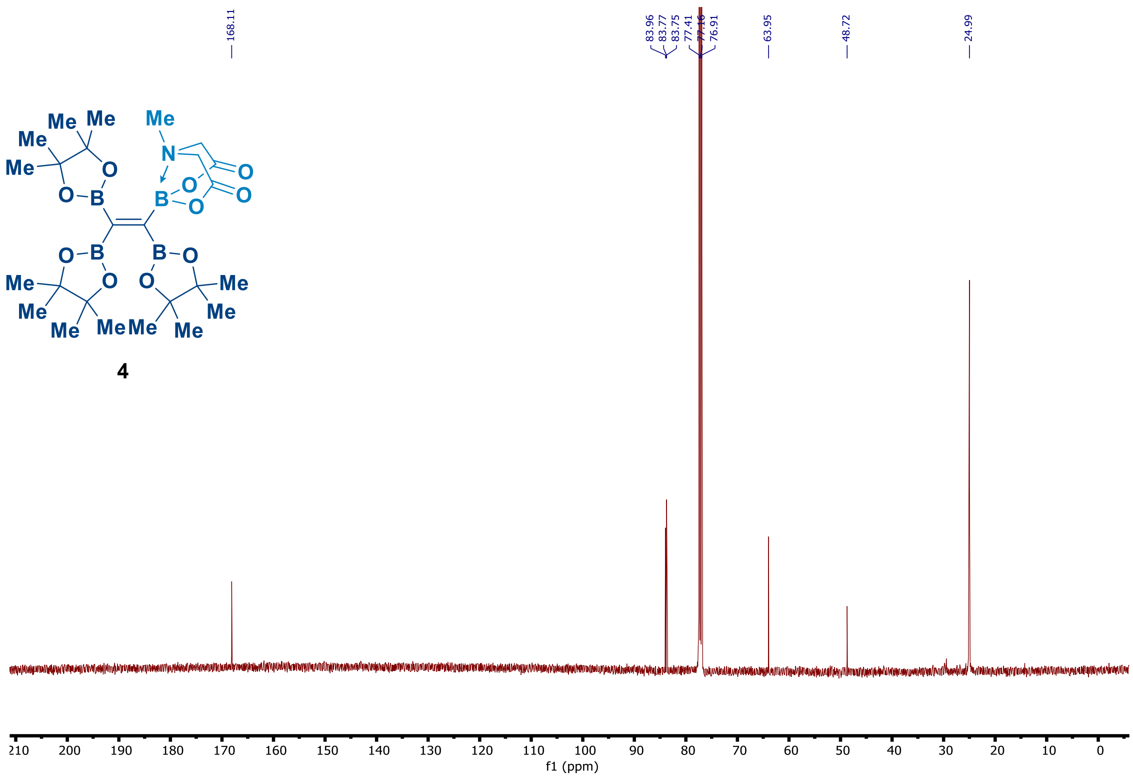

Supplementary Figure 247. ^{13}C NMR (101 MHz, $CDCl_3$) of compound (**4**).

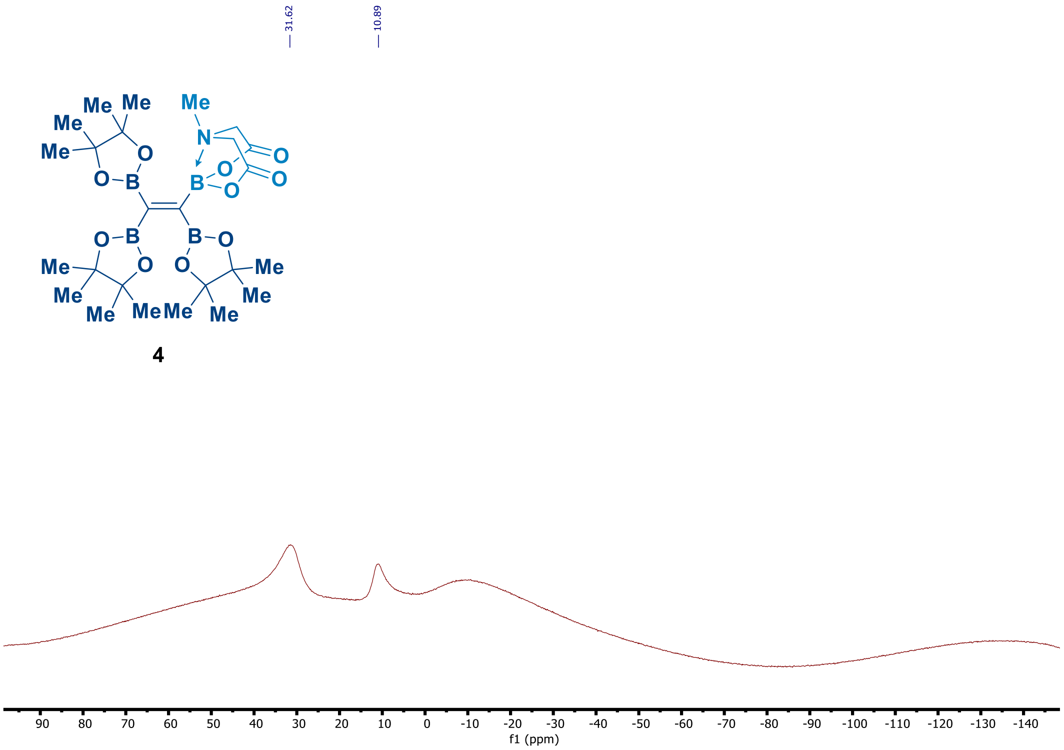

Supplementary Figure 248. ^{11}B NMR (128 MHz, $CDCl_3$) of compound (**4**).

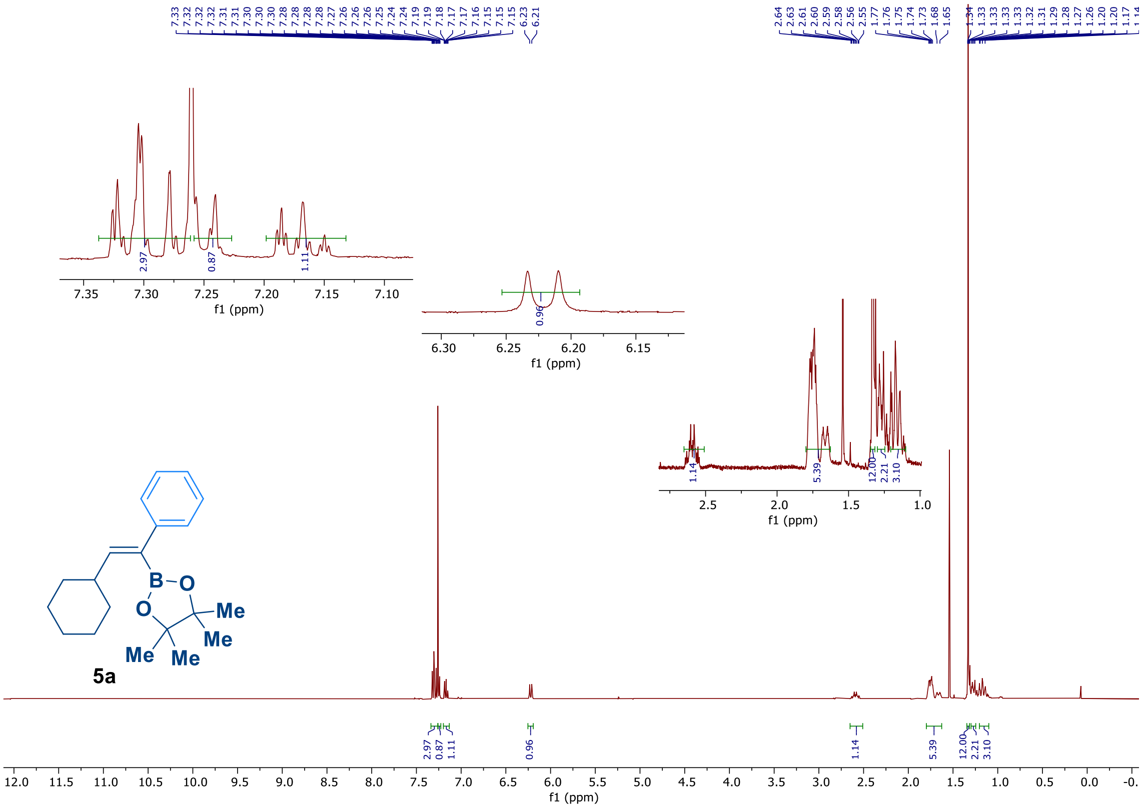

Supplementary Figure 249. ^{1}H NMR (400 MHz, $CDCl_3$) of compound (**5a**).

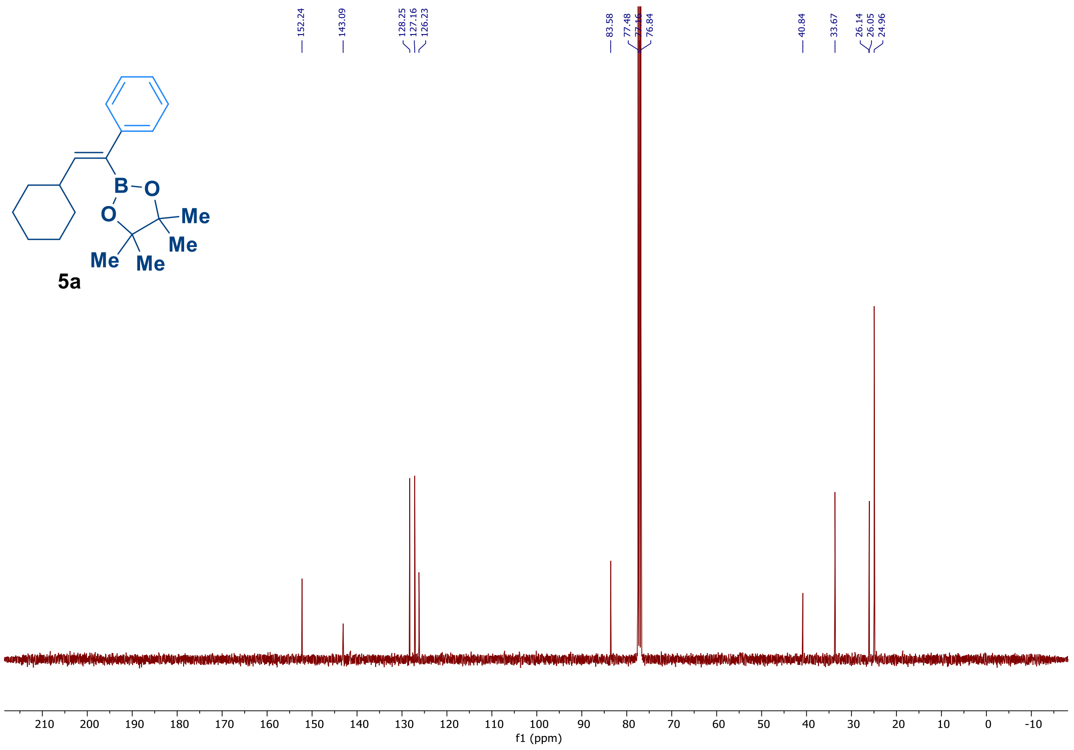

Supplementary Figure 250. ^{13}C NMR (101 MHz, $CDCl_3$) of compound (**5a**).

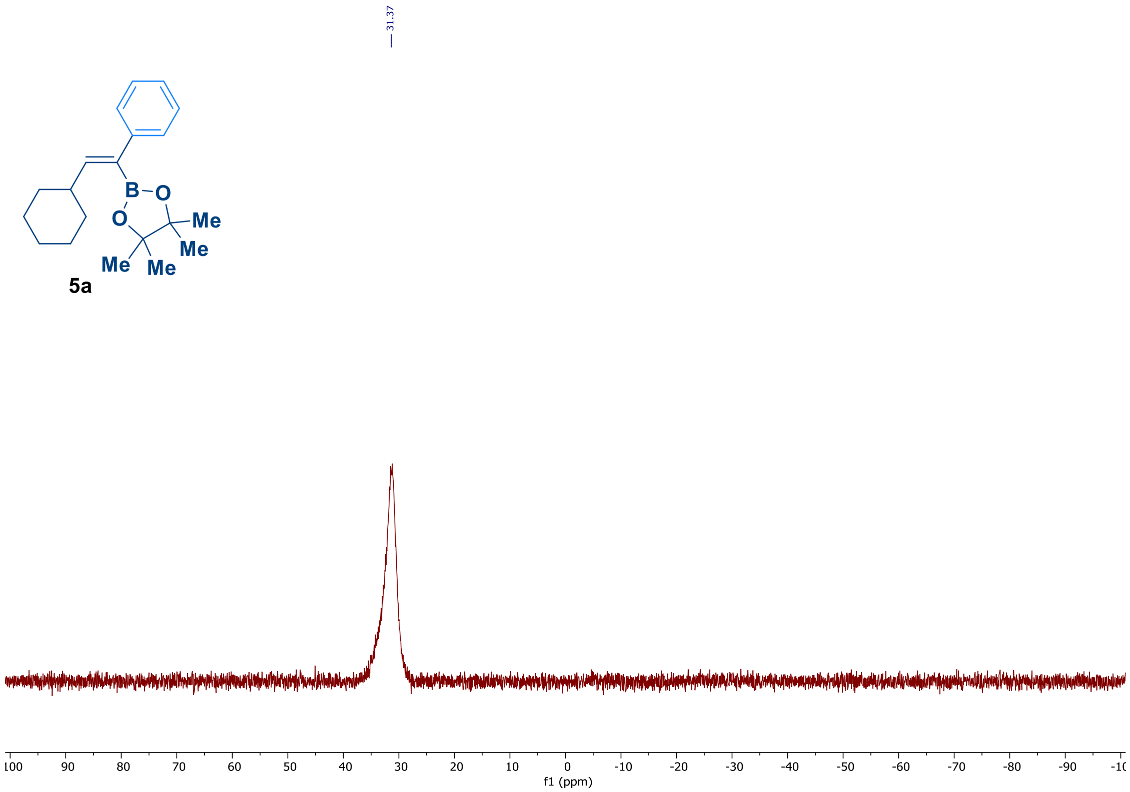

Supplementary Figure 251. ^{11}B NMR (128 MHz, $CDCl_3$) of compound (**5a**).

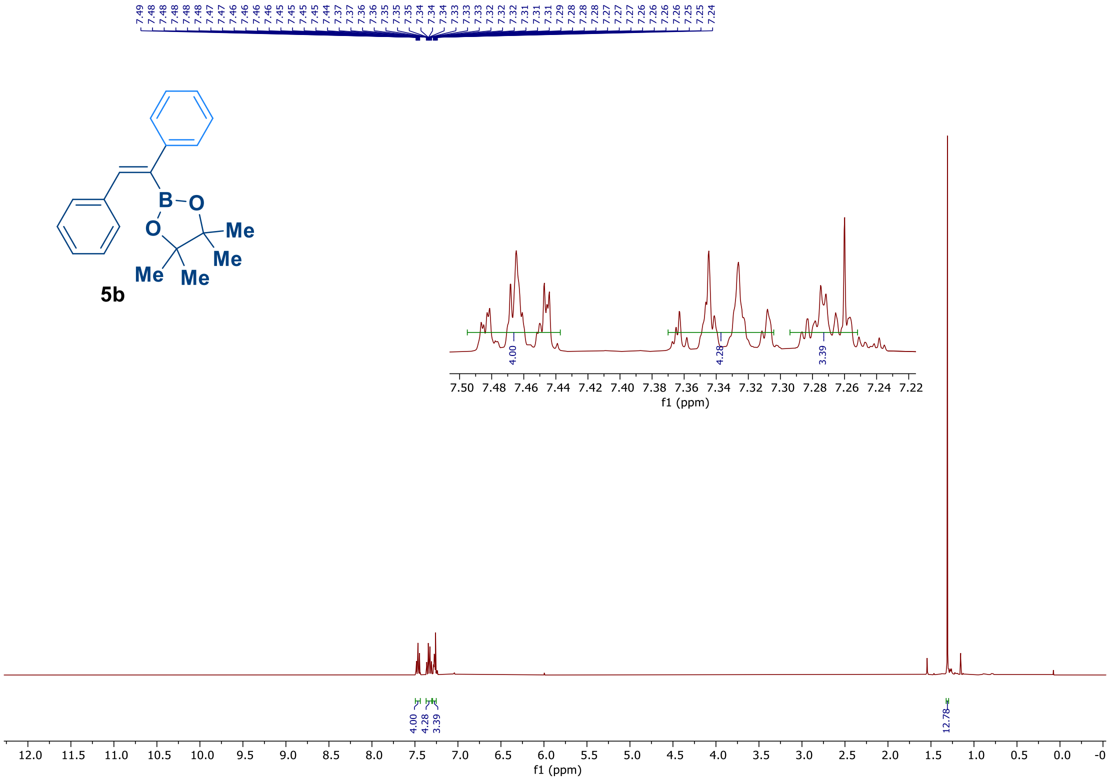

Supplementary Figure 252. ^{1}H NMR (400 MHz, $CDCl_3$) of compound (**5b**).

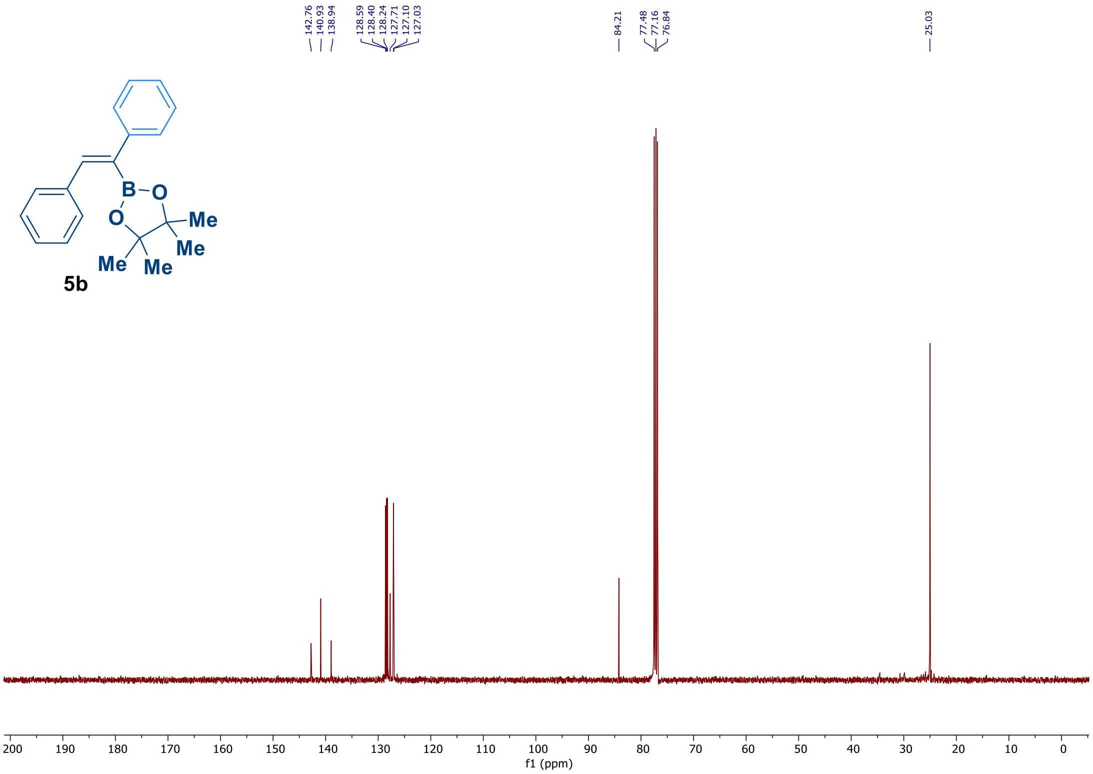

Supplementary Figure 253. ^{13}C NMR (101 MHz, $CDCl_3$) of compound (**5b**).

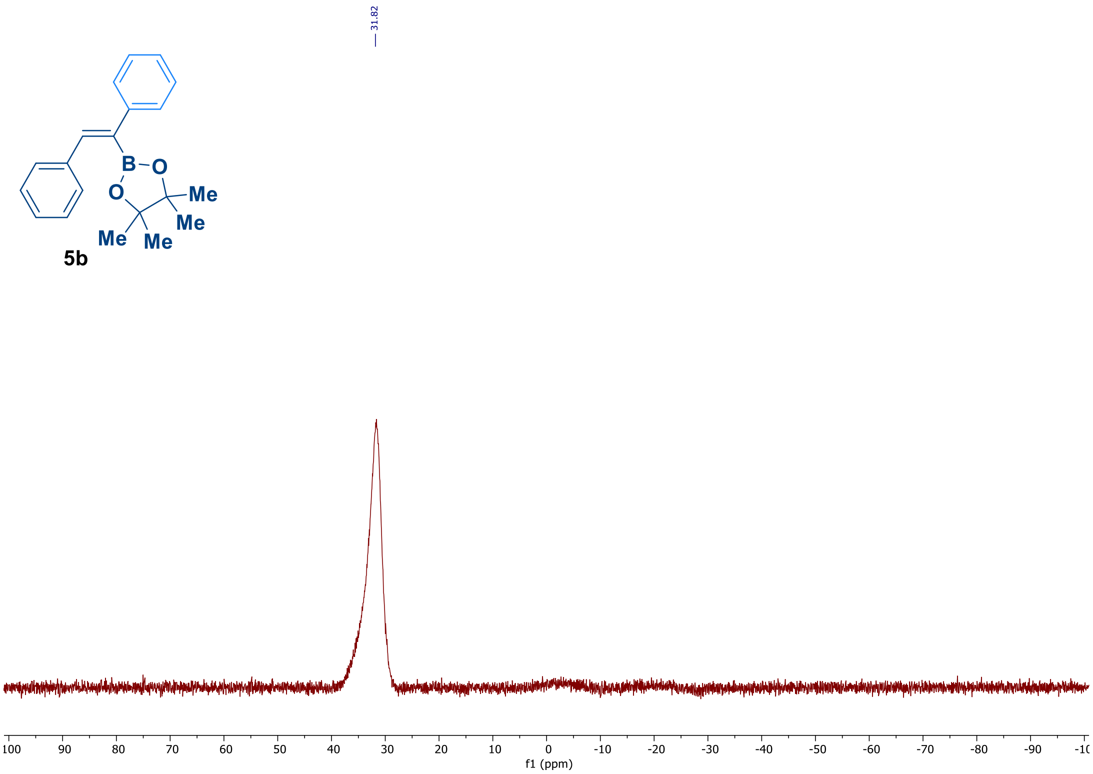

Supplementary Figure 254. ^{11}B NMR (128 MHz, $CDCl_3$) of compound (**5b**).

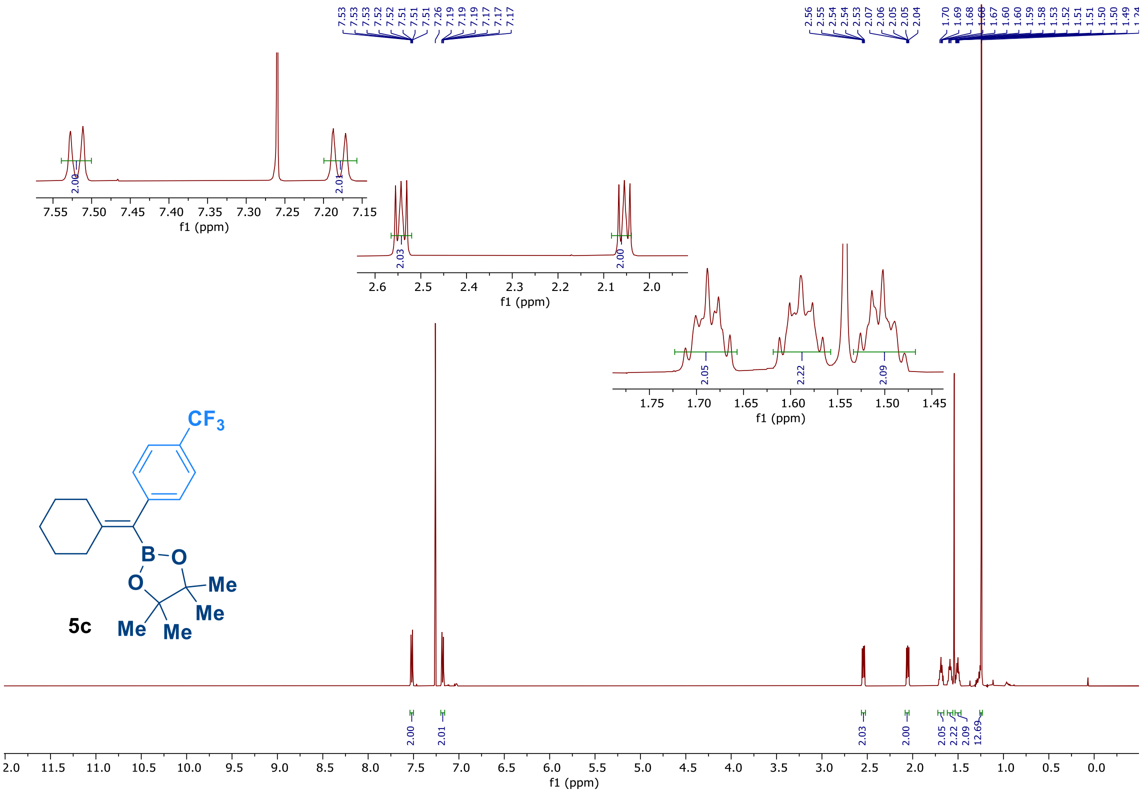

Supplementary Figure 255. ^{1}H NMR (400 MHz, $CDCl_3$) of compound (**5c**).

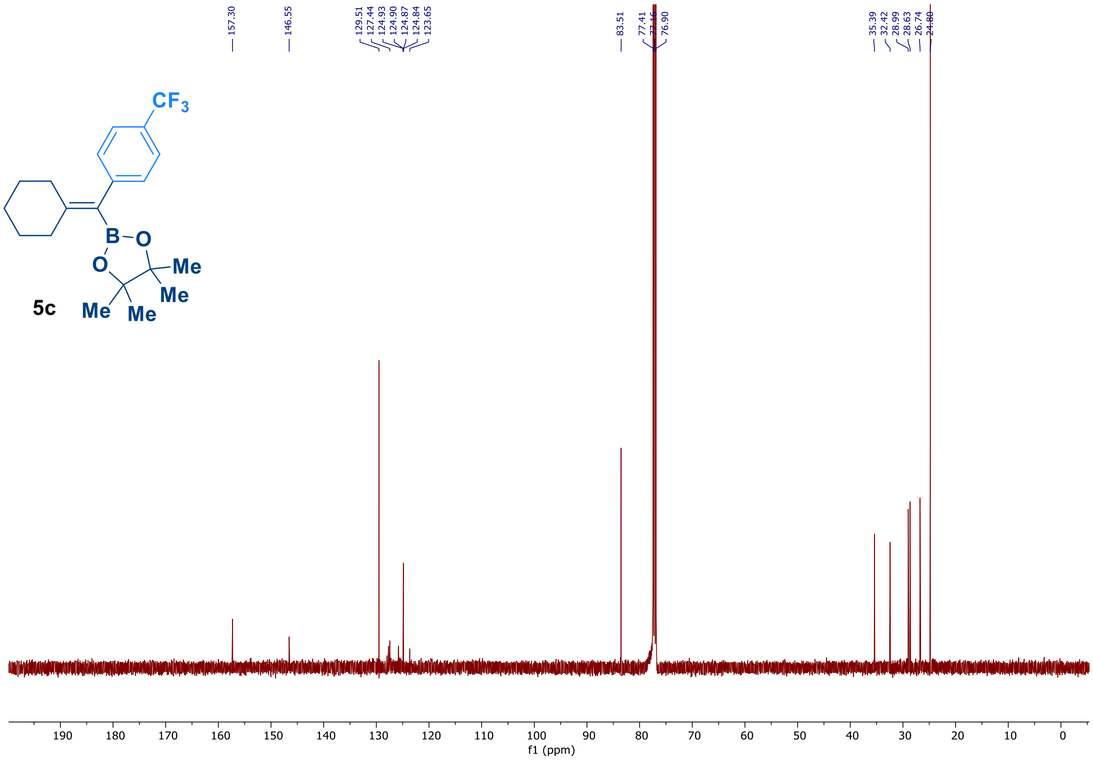

Supplementary Figure 256. ^{13}C NMR (101 MHz, $CDCl_3$) of compound (**5c**).

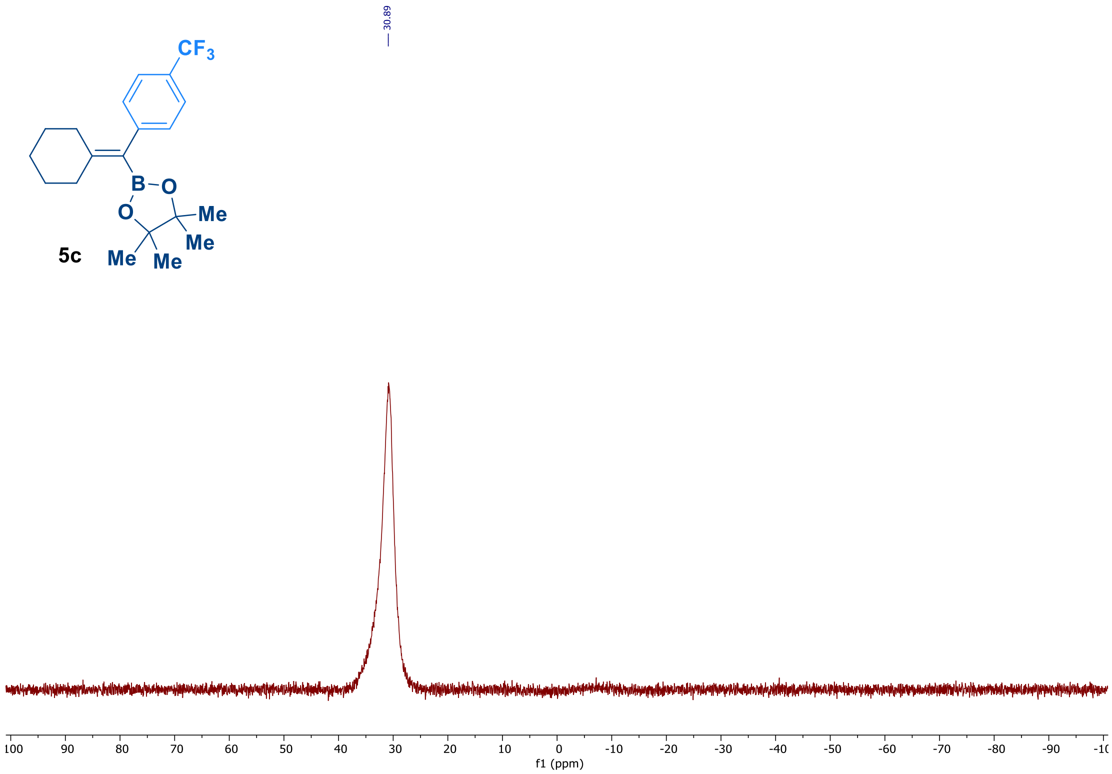

Supplementary Figure 257. ^{11}B NMR (128 MHz, $CDCl_3$) of compound (**5c**).

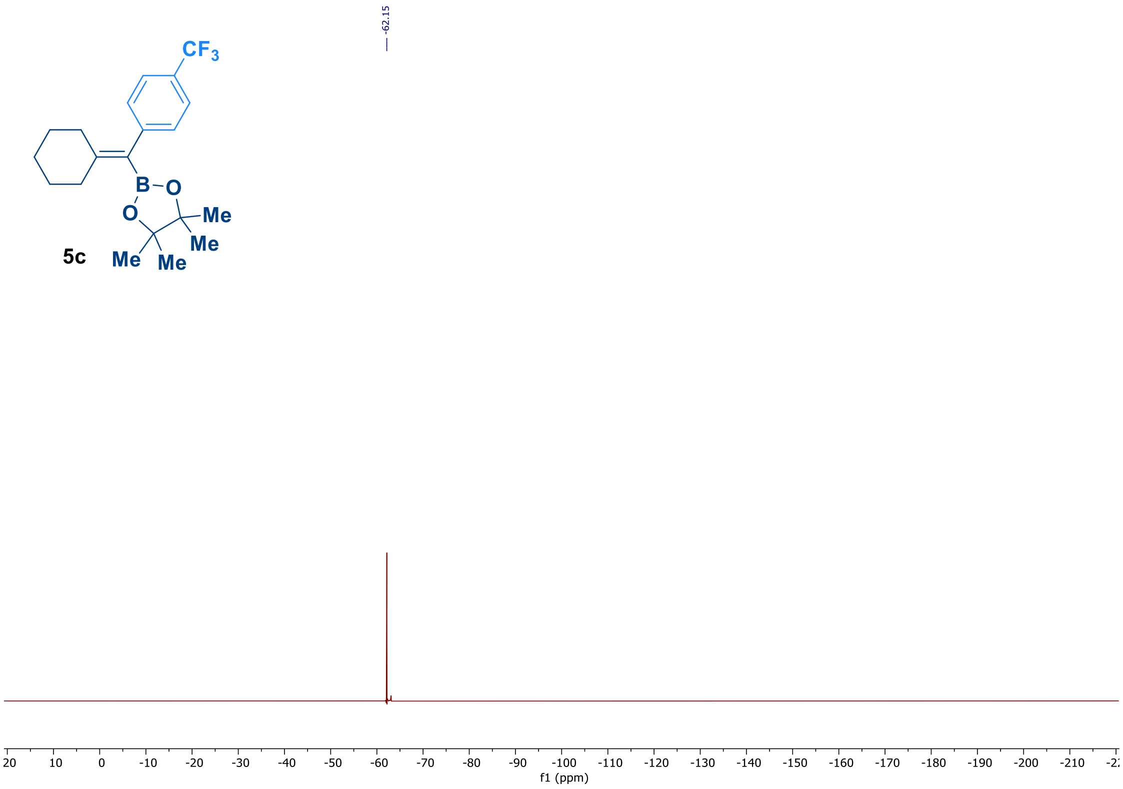

Supplementary Figure 258. ^{19}F NMR (376 MHz, $CDCl_3$) of compound (**5c**).

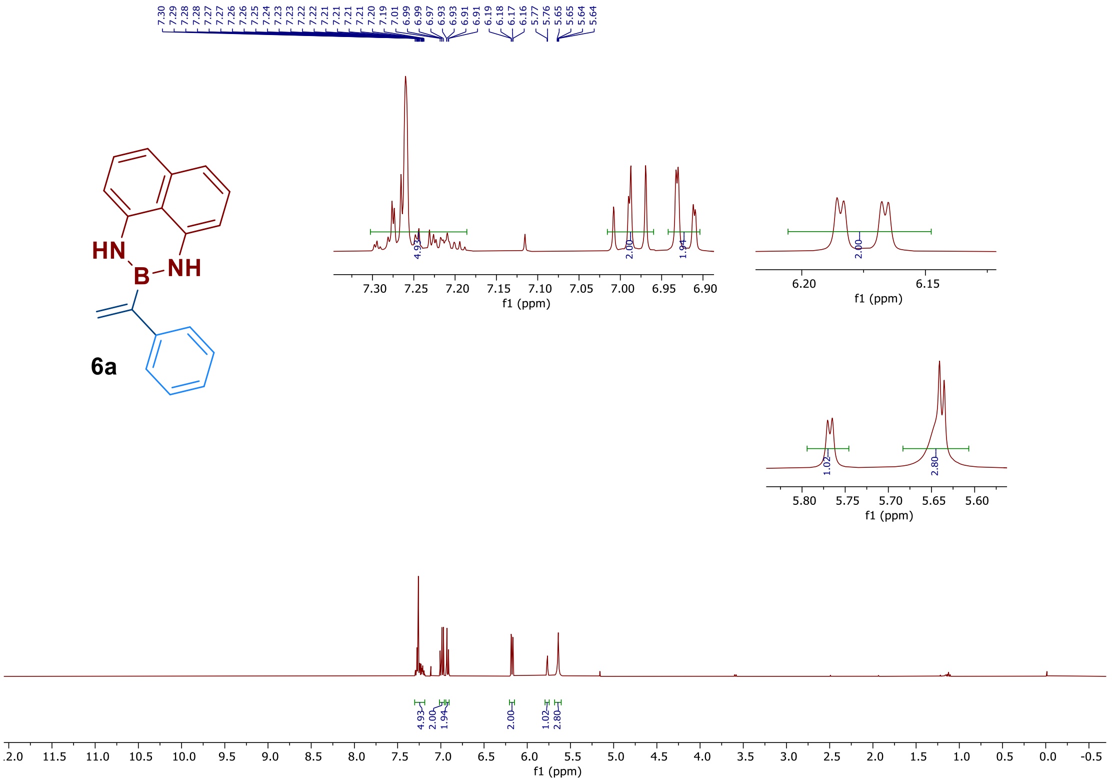

Supplementary Figure 259. ^{1}H NMR (400 MHz, $CDCl_3$) of compound (**6a**).

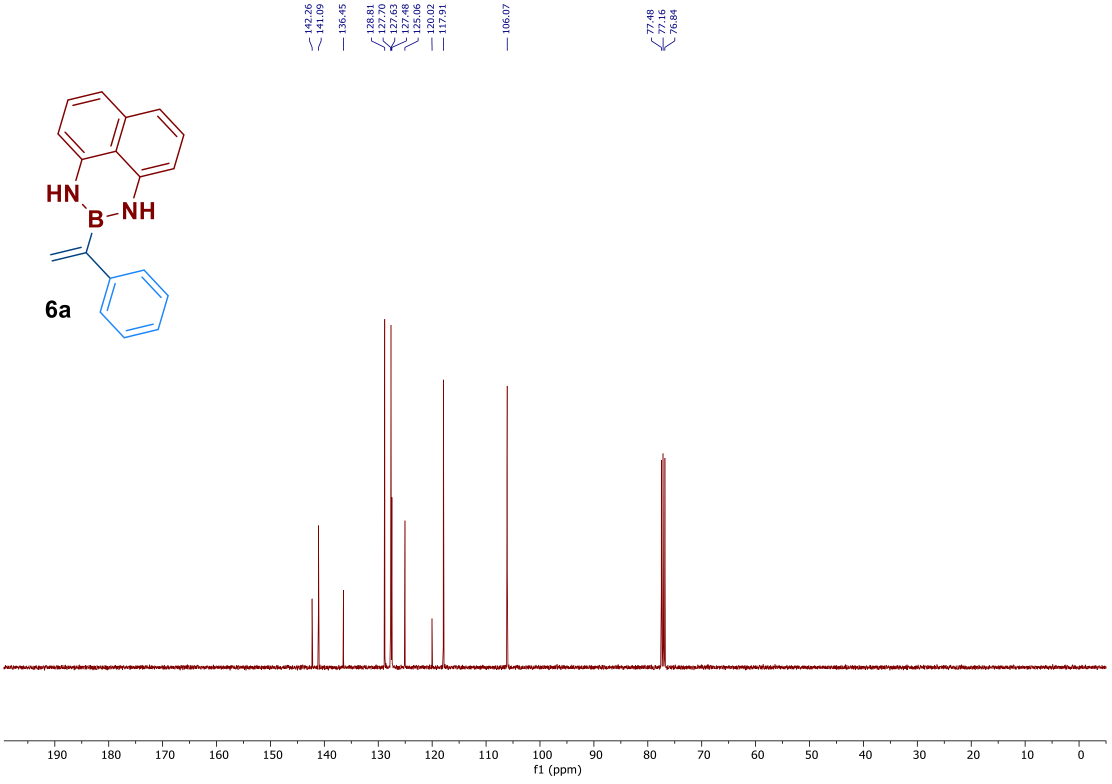

Supplementary Figure 260. ^{13}C NMR (101 MHz, $CDCl_3$) of compound (**6a**).

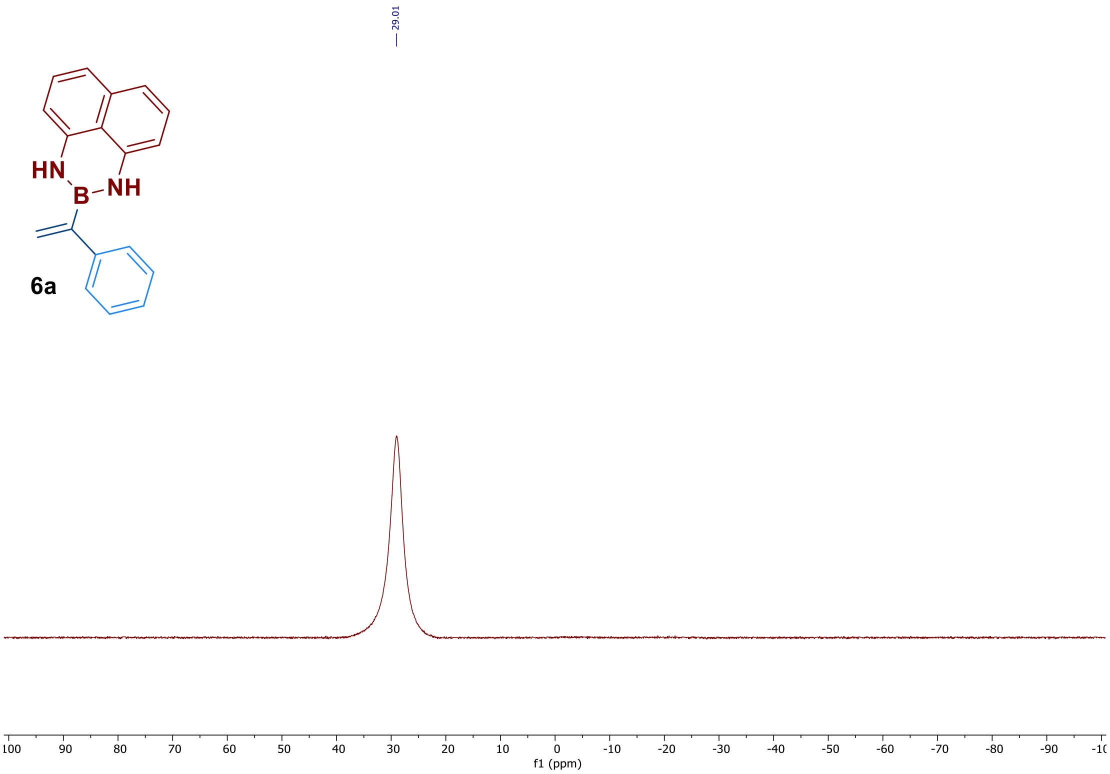

Supplementary Figure 261. ^{11}B NMR (128 MHz, $CDCl_3$) of compound (**6a**).

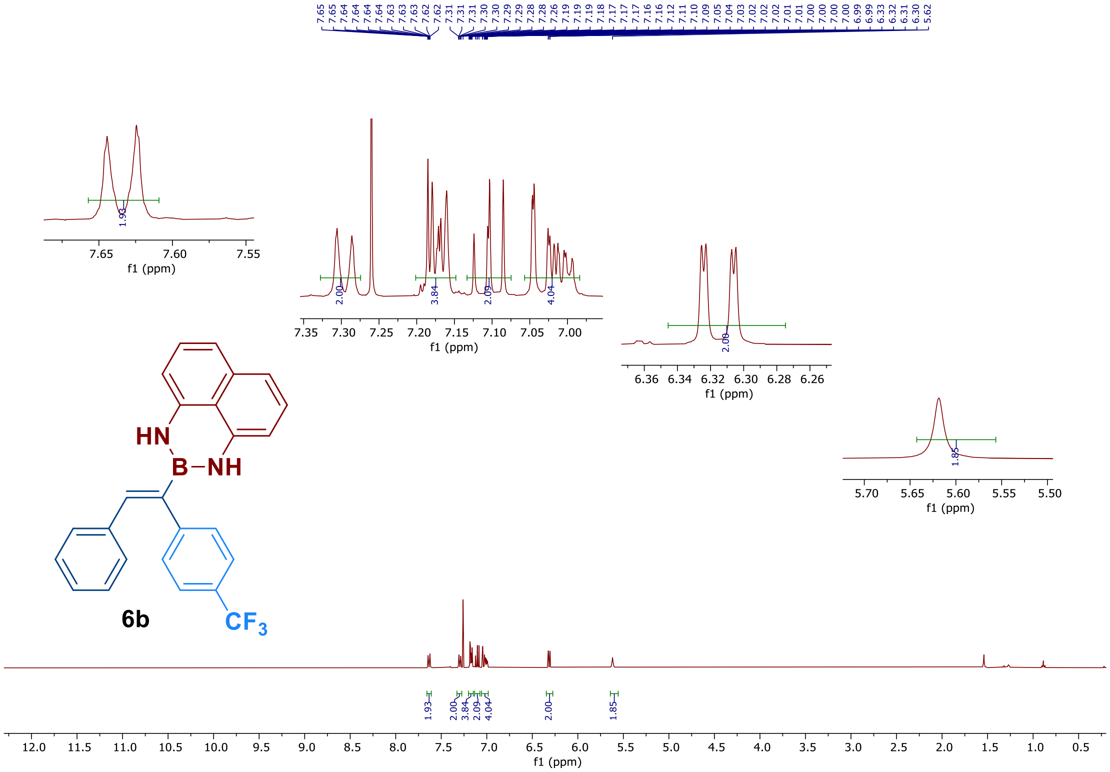

Supplementary Figure 262. ^{1}H NMR (400 MHz, $CDCl_3$) of compound (**6b**).

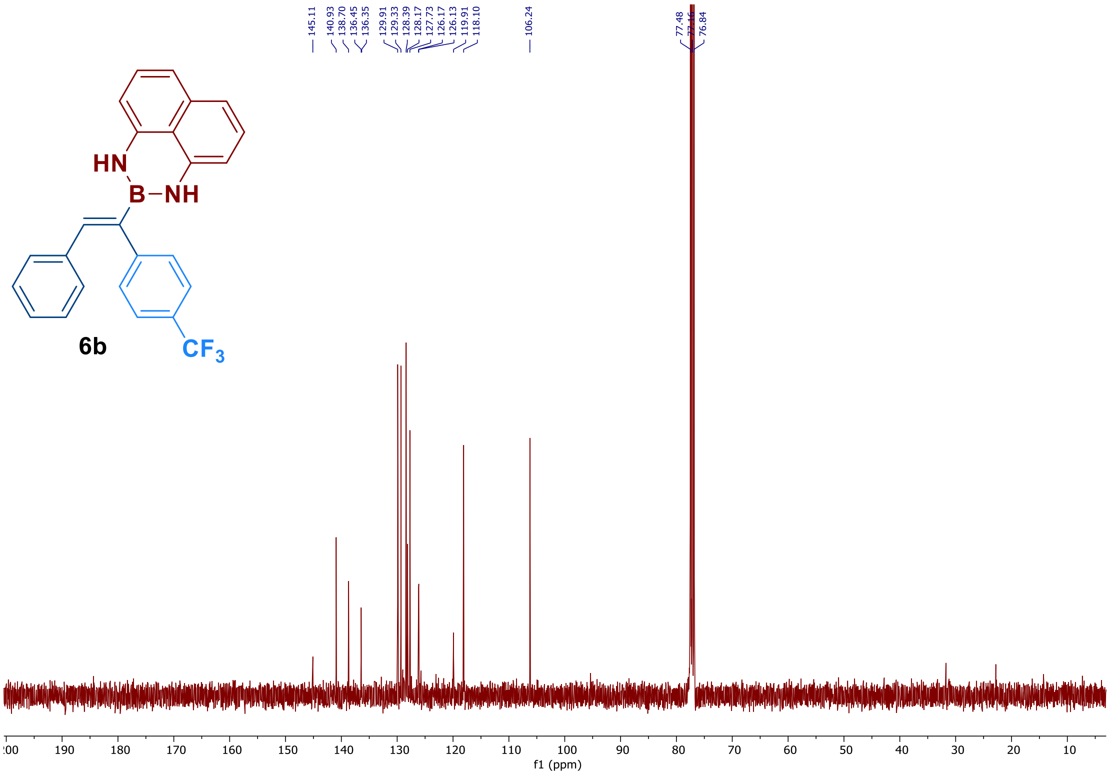

Supplementary Figure 263. ^{13}C NMR (101 MHz, $CDCl_3$) of compound (**6b**).

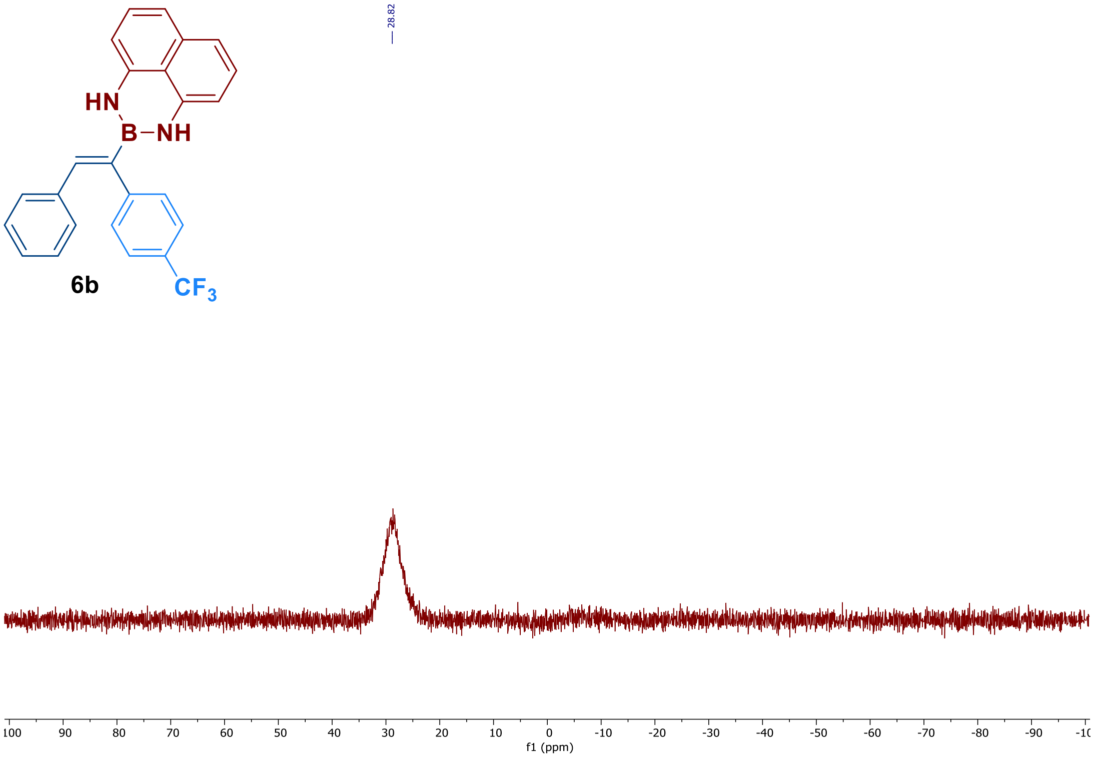

Supplementary Figure 264. ^{11}B NMR (128 MHz, $CDCl_3$) of compound (**6b**).

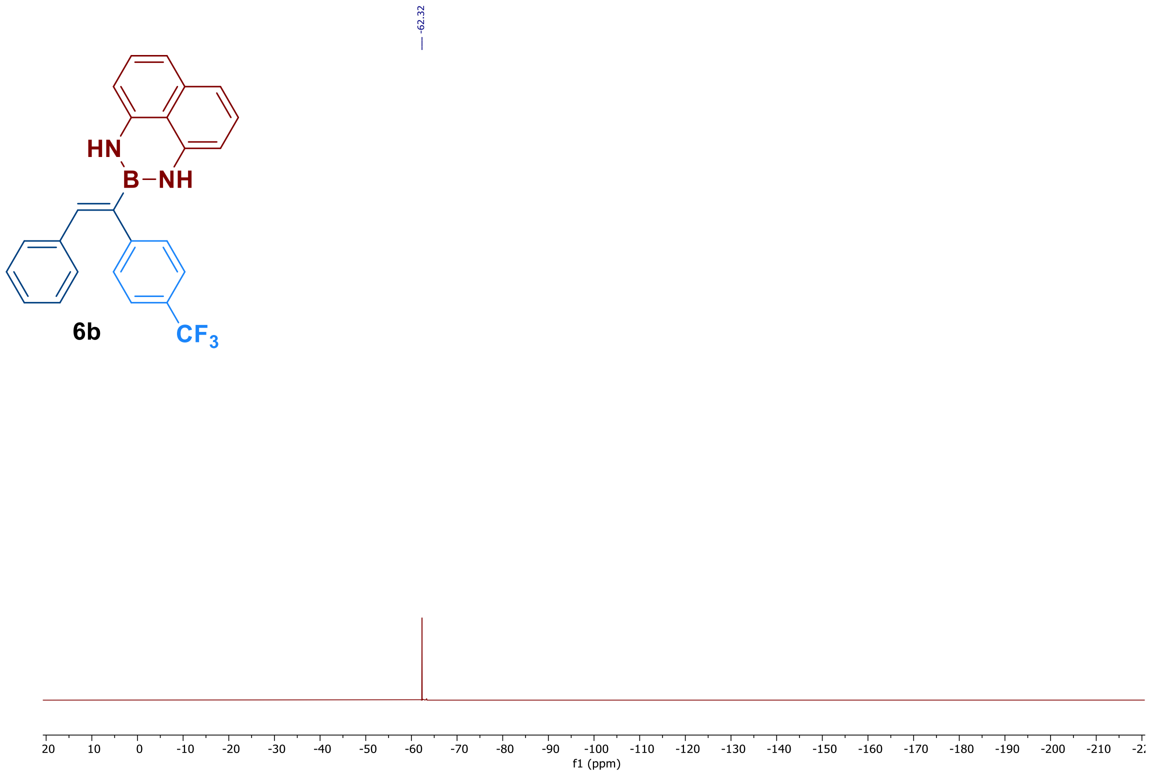

Supplementary Figure 265. ^{19}F NMR (376 MHz, $CDCl_3$) of compound (**6b**).

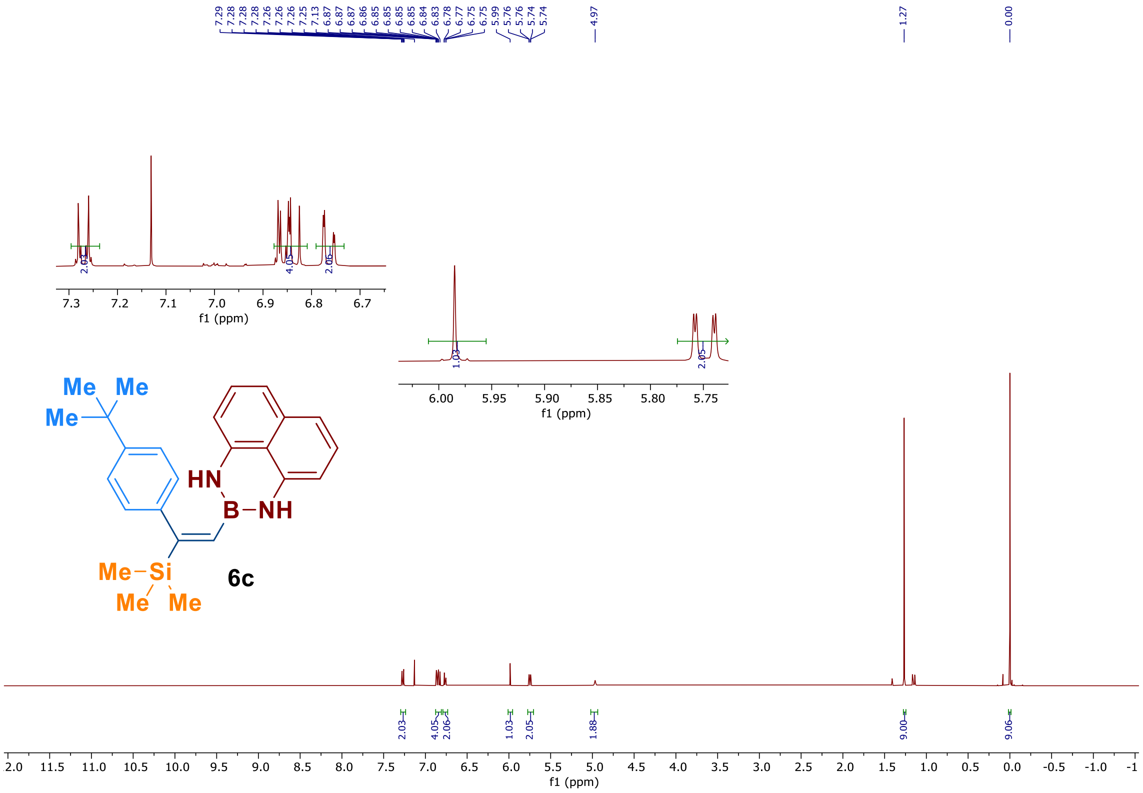

Supplementary Figure 266. ^{1}H NMR (400 MHz, $CDCl_3$) of compound (**6c**).

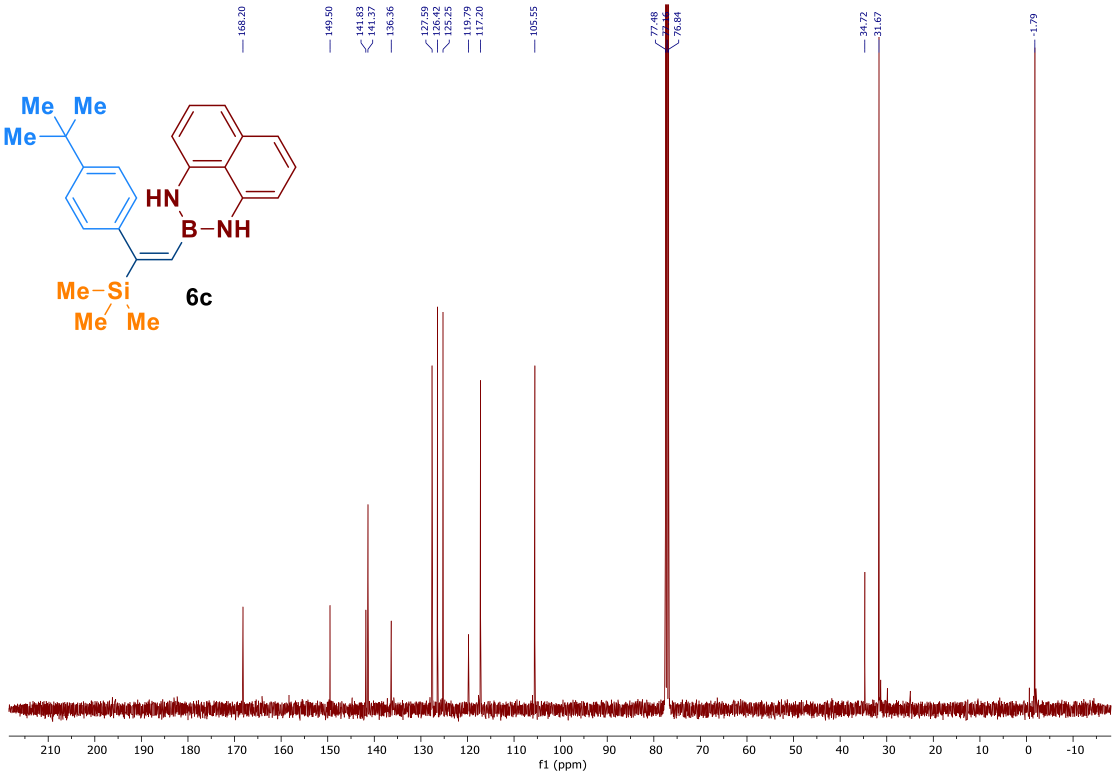

Supplementary Figure 267. ^{13}C NMR (101 MHz, $CDCl_3$) of compound (**6c**).

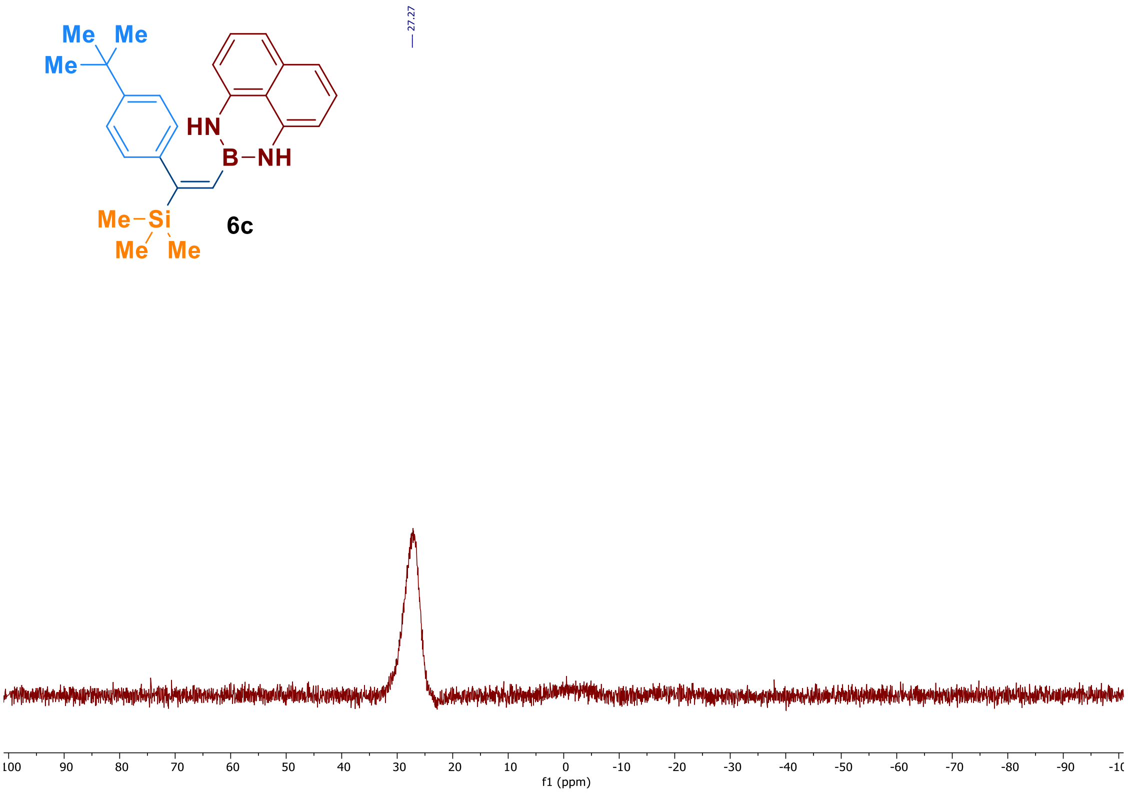

Supplementary Figure 268. ^{11}B NMR (128 MHz, $CDCl_3$) of compound (**6c**).

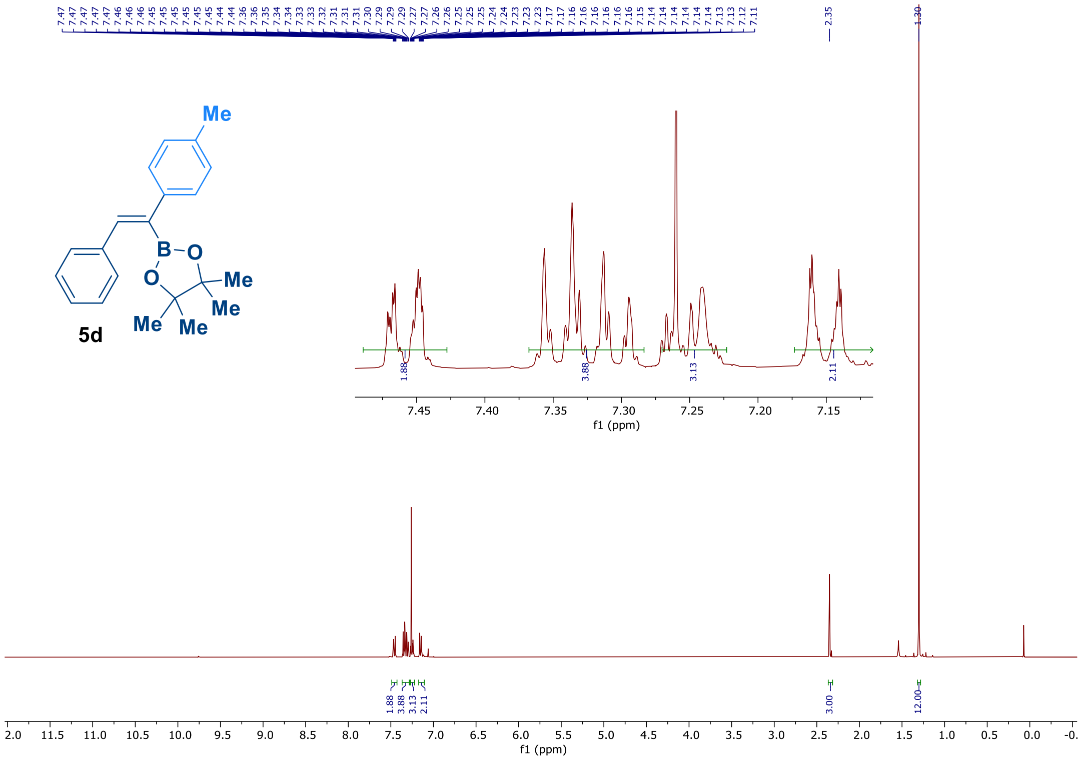

Supplementary Figure 269. ^{1}H NMR (400 MHz, $CDCl_3$) of compound (**5d**).

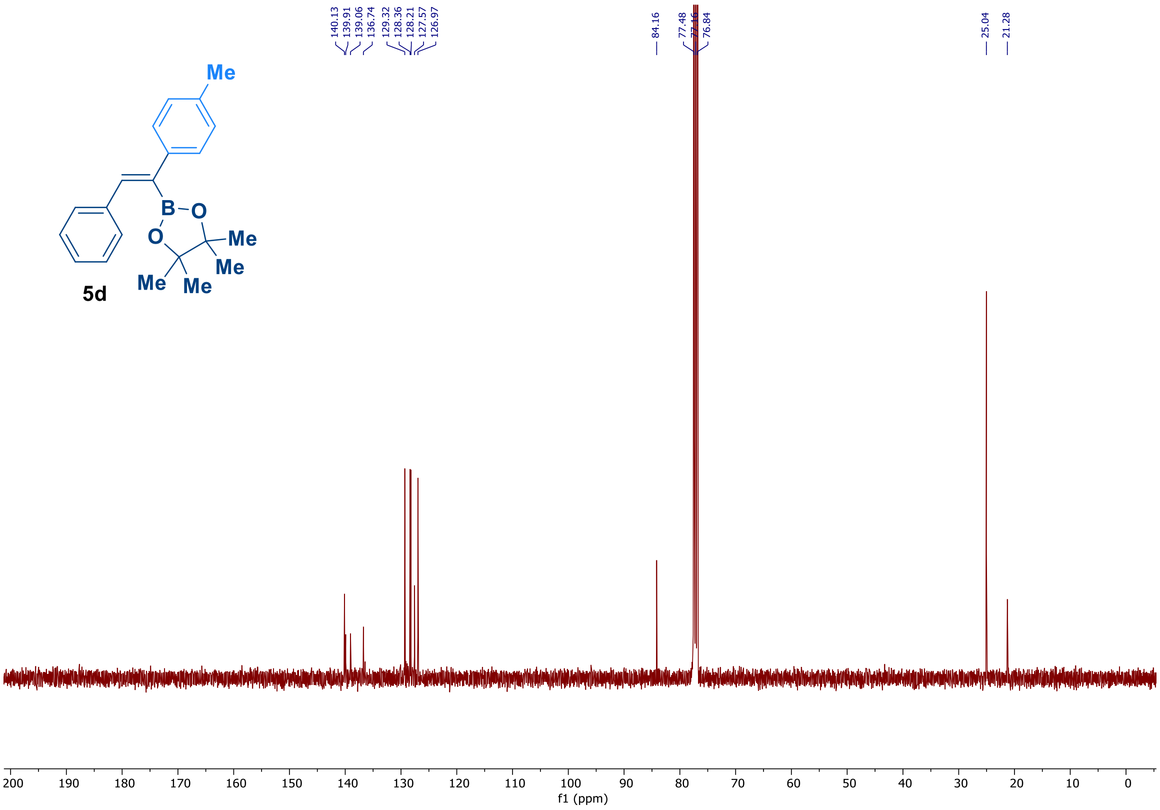

Supplementary Figure 270. ^{13}C NMR (101 MHz, $CDCl_3$) of compound (**5d**).

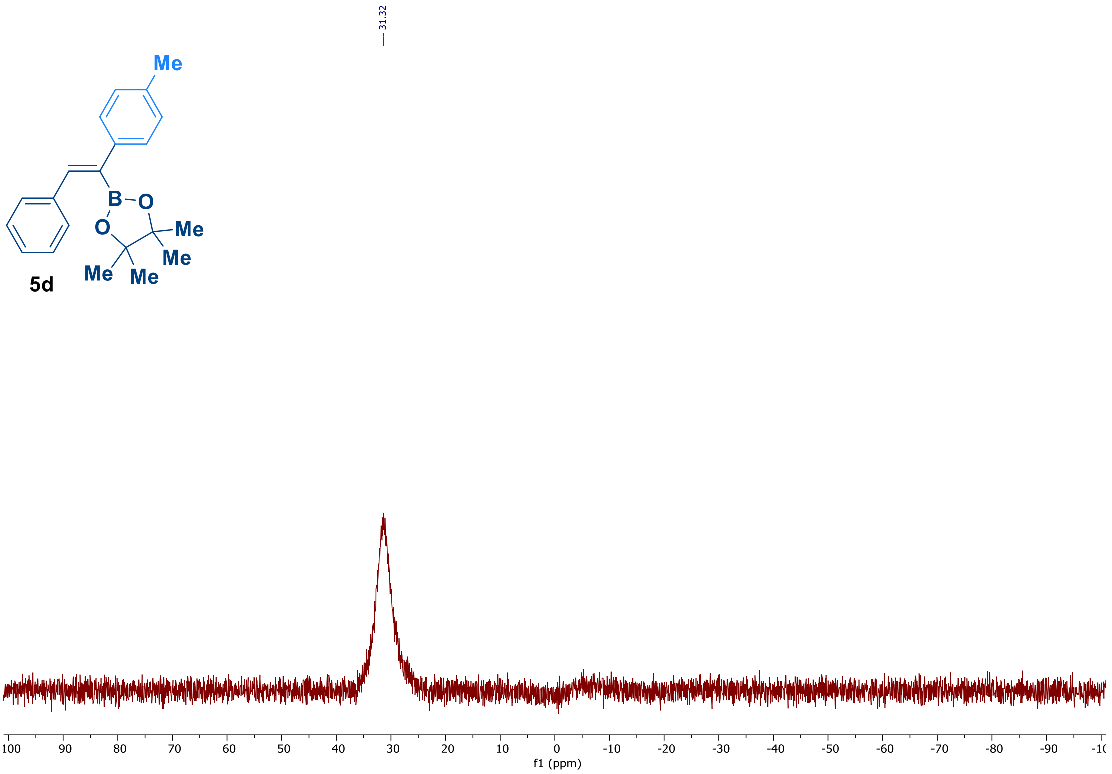

Supplementary Figure 271. ^{11}B NMR (128 MHz, $CDCl_3$) of compound (**5d**).

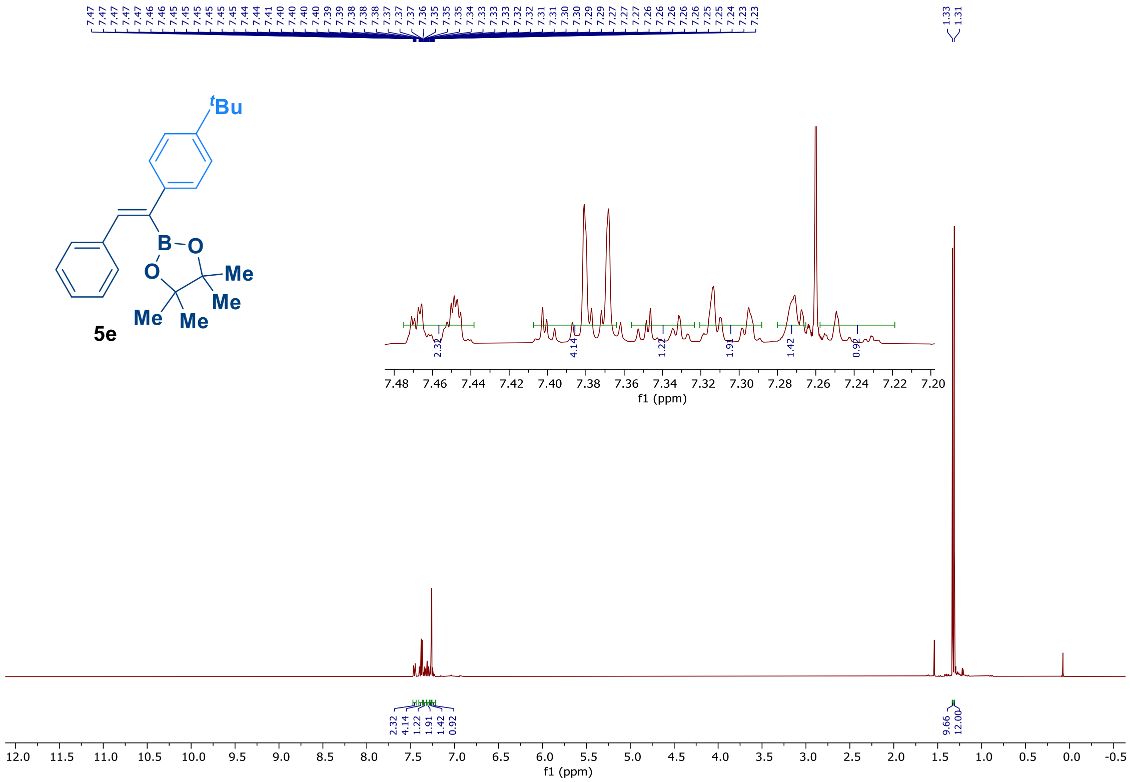

Supplementary Figure 272. ^{1}H NMR (400 MHz, $CDCl_3$) of compound (**5e**).

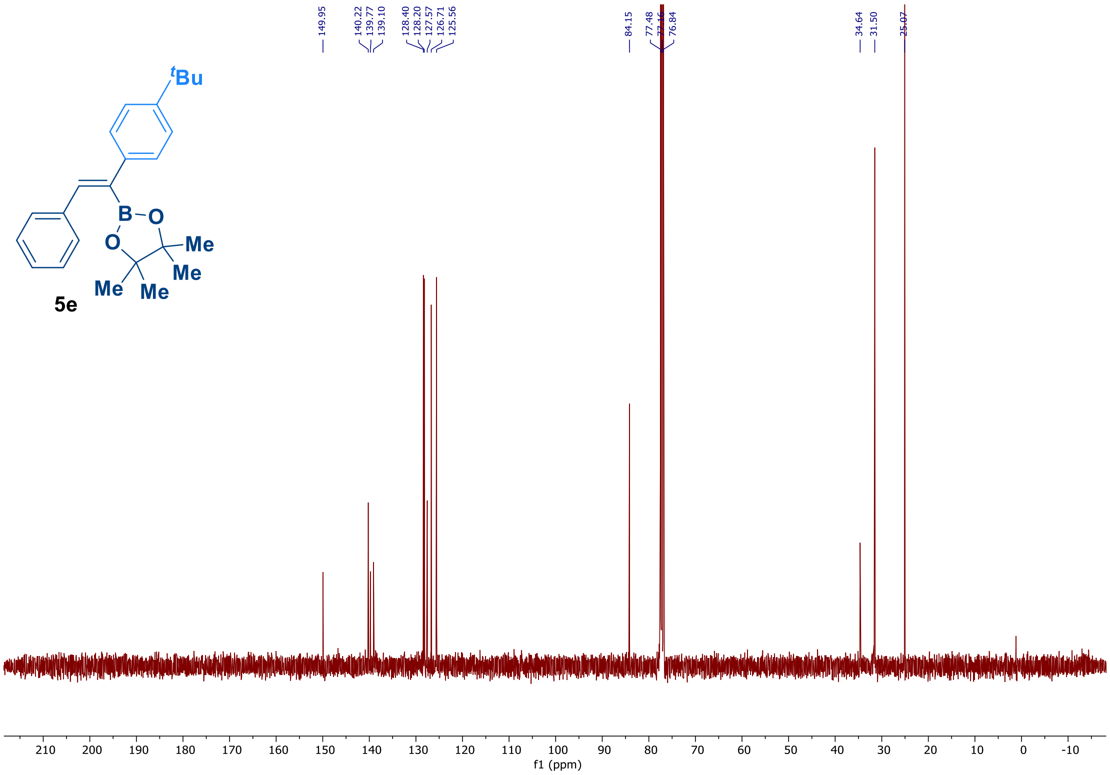

Supplementary Figure 273. ^{13}C NMR (101 MHz, $CDCl_3$) of compound (**5e**).

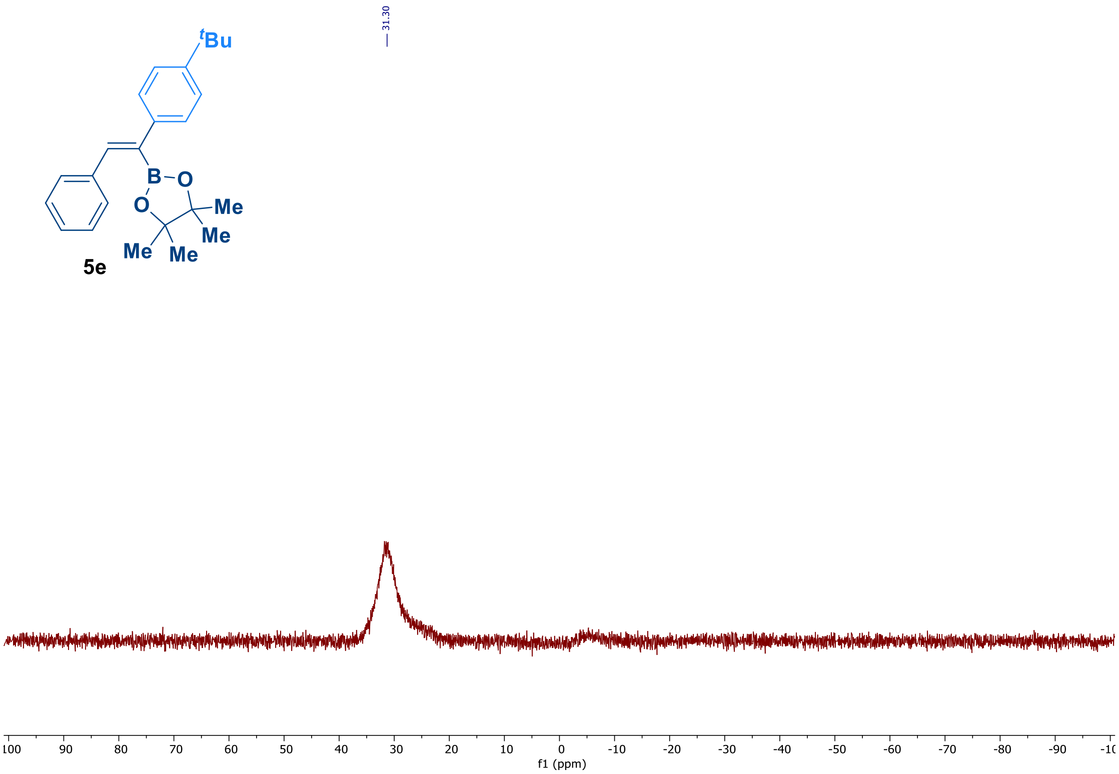

Supplementary Figure 274. ^{11}B NMR (128 MHz, $CDCl_3$) of compound (**5e**).

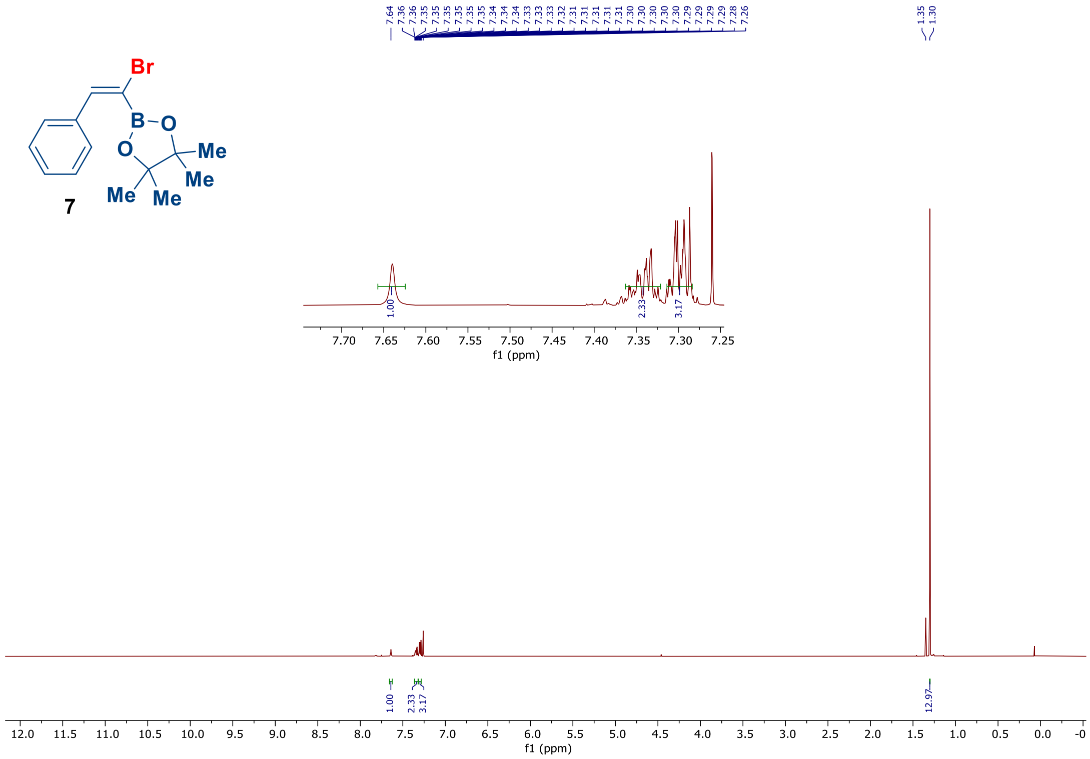

Supplementary Figure 275. ^{1}H NMR (400 MHz, $CDCl_3$) of compound (**7**).

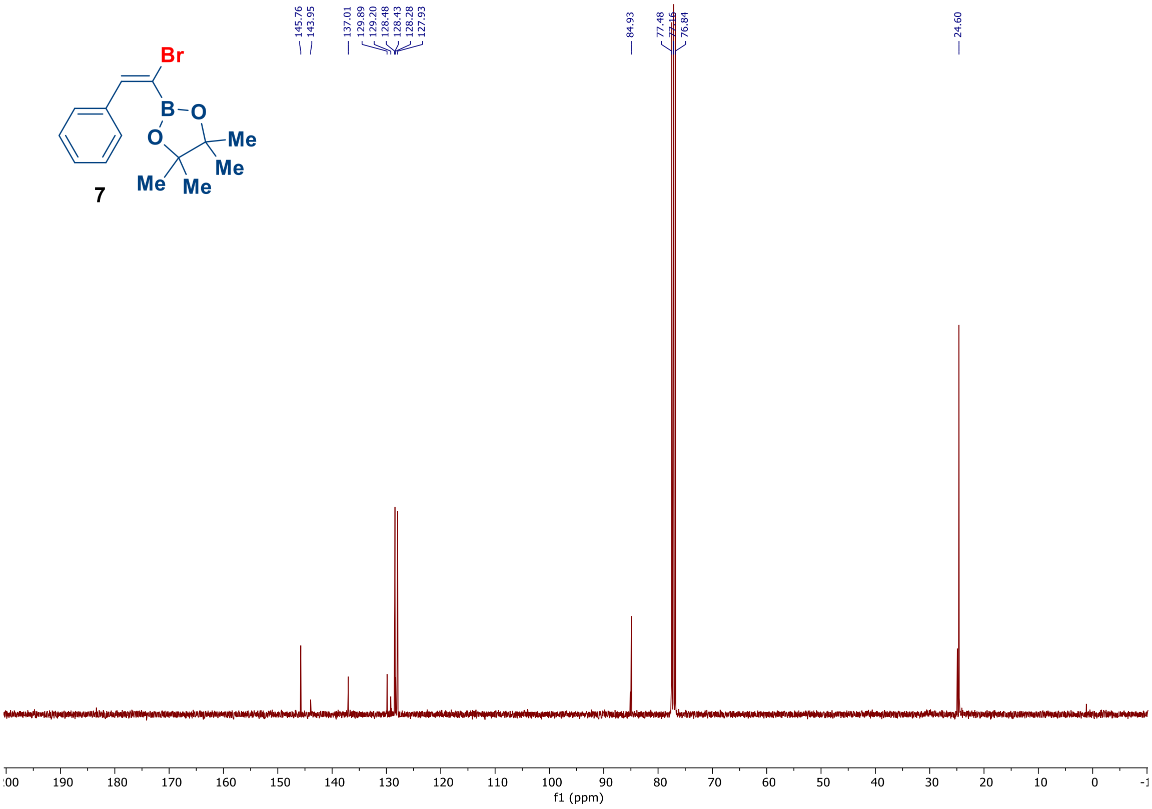

Supplementary Figure 276. ^{13}C NMR (101 MHz, $CDCl_3$) of compound (**5d**).

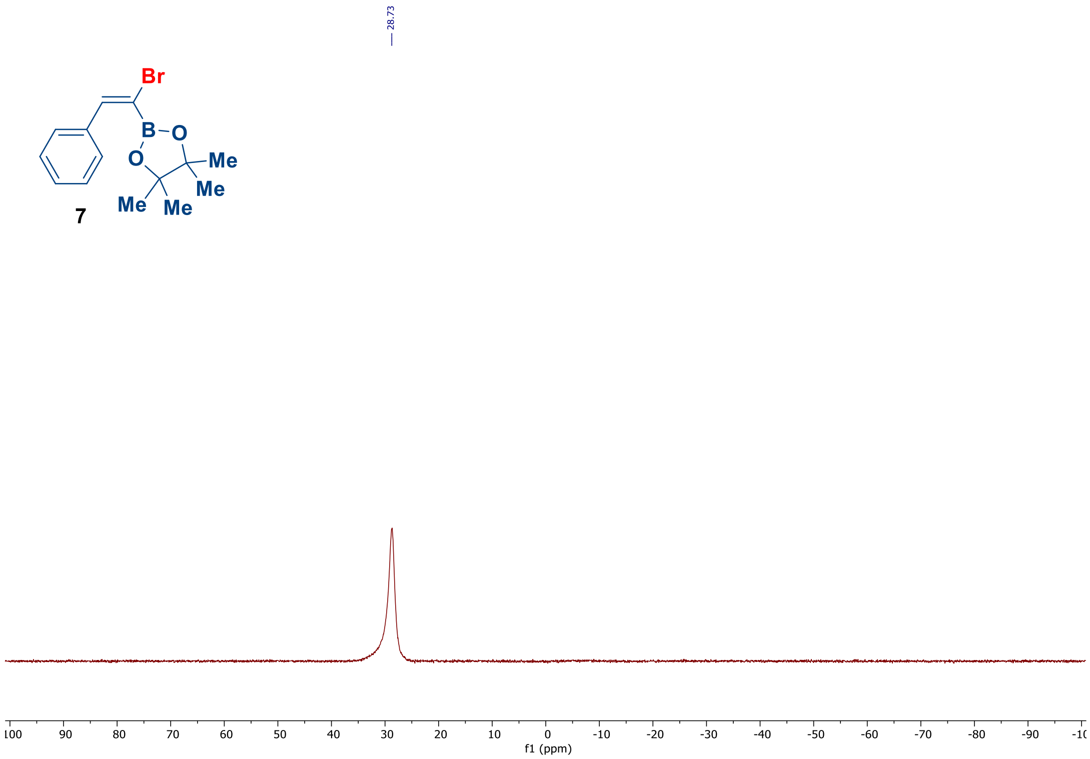

Supplementary Figure 277. ^{11}B NMR (128 MHz, $CDCl_3$) of compound (**7**).

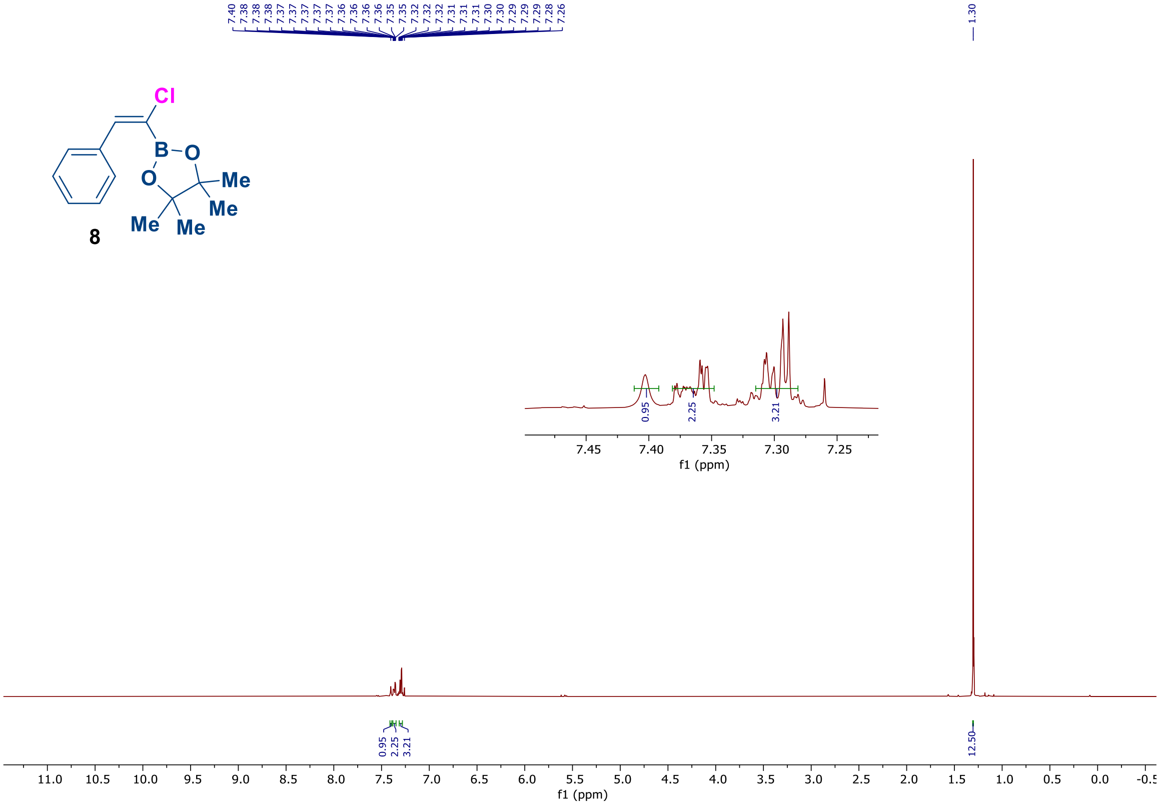

Supplementary Figure 278. ^{1}H NMR (400 MHz, $CDCl_3$) of compound (**8**).

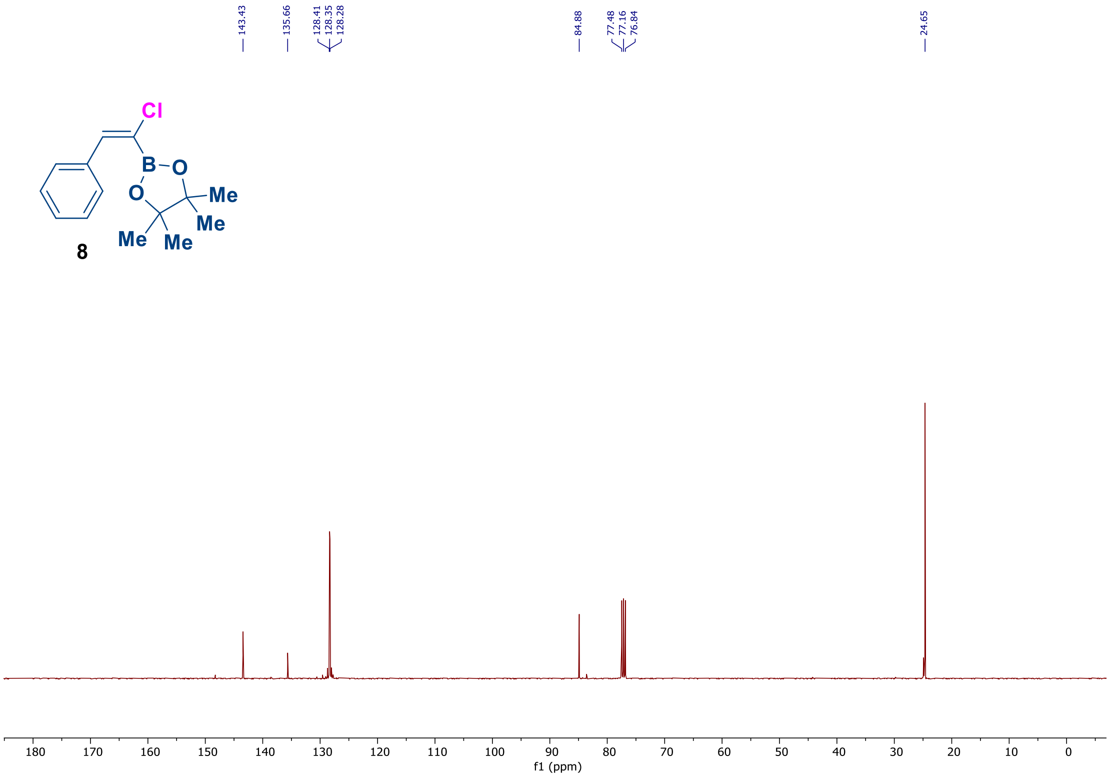

Supplementary Figure 279. ^{13}C NMR (101 MHz, $CDCl_3$) of compound (**8**).

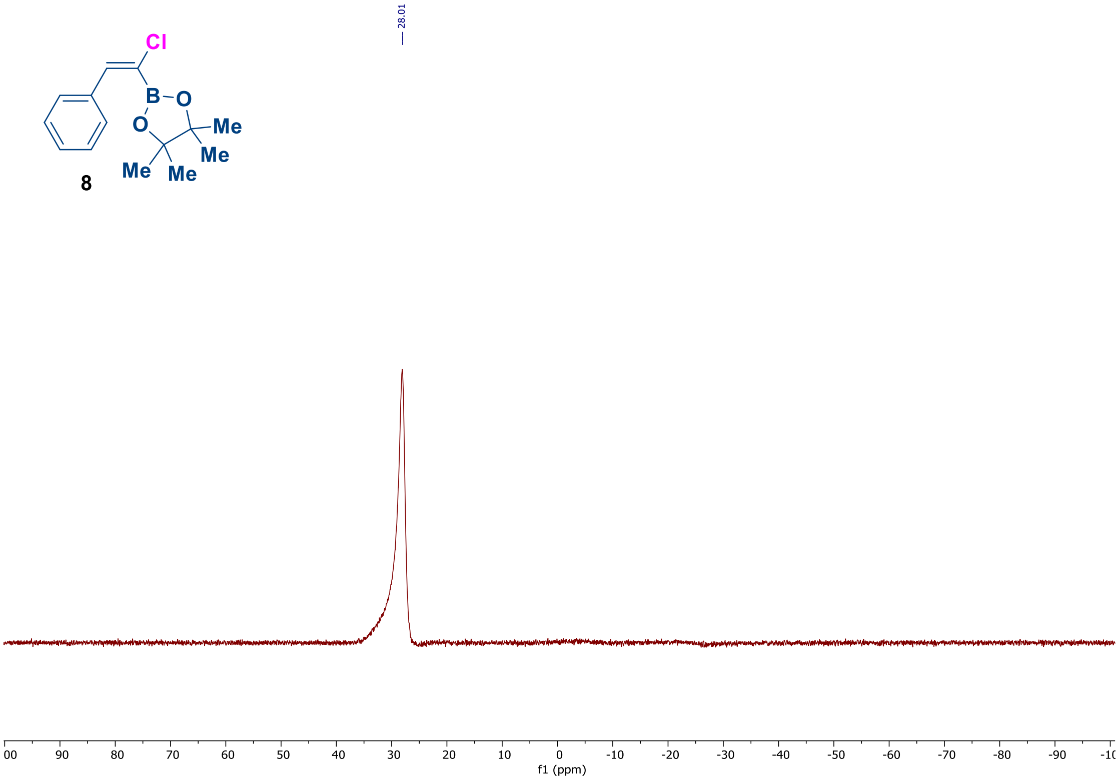

Supplementary Figure 280. ^{11}B NMR (128 MHz, $CDCl_3$) of compound (**8**).

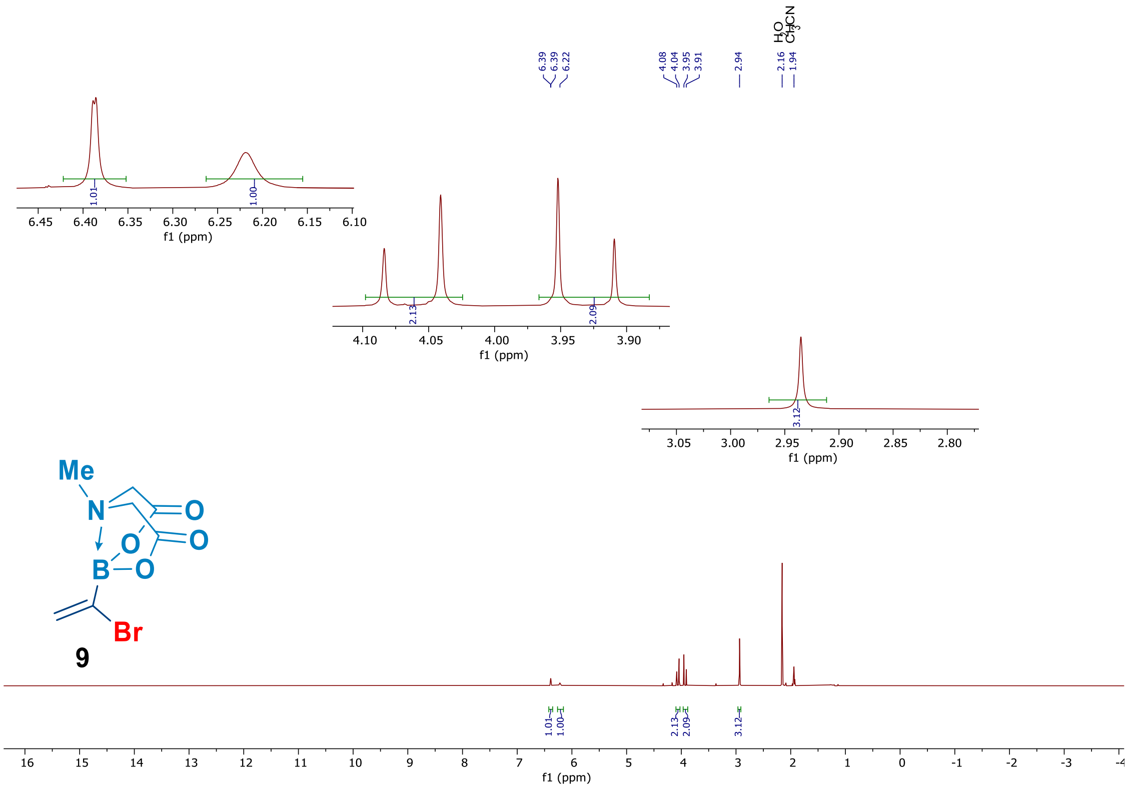

Supplementary Figure 281. ^{1}H NMR (400 MHz, $CDCl_3$) of compound (**9**).

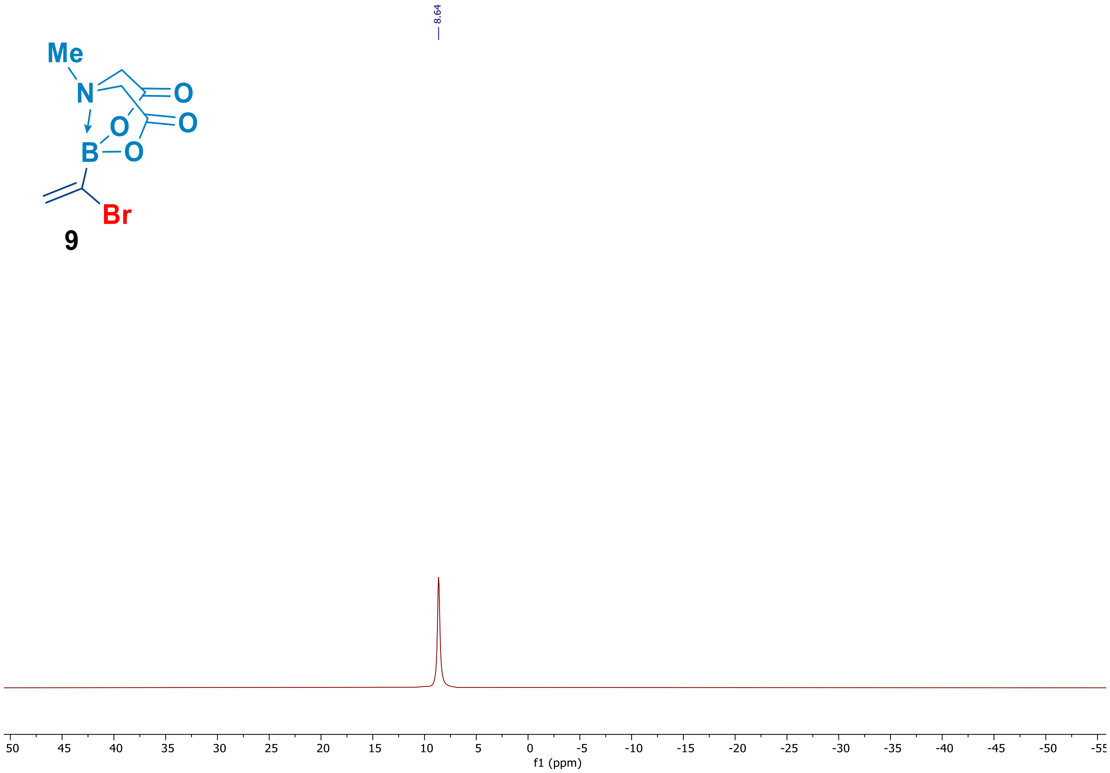

Supplementary Figure 282. ^{11}B NMR (128 MHz, $CDCl_3$) of compound (**9**).

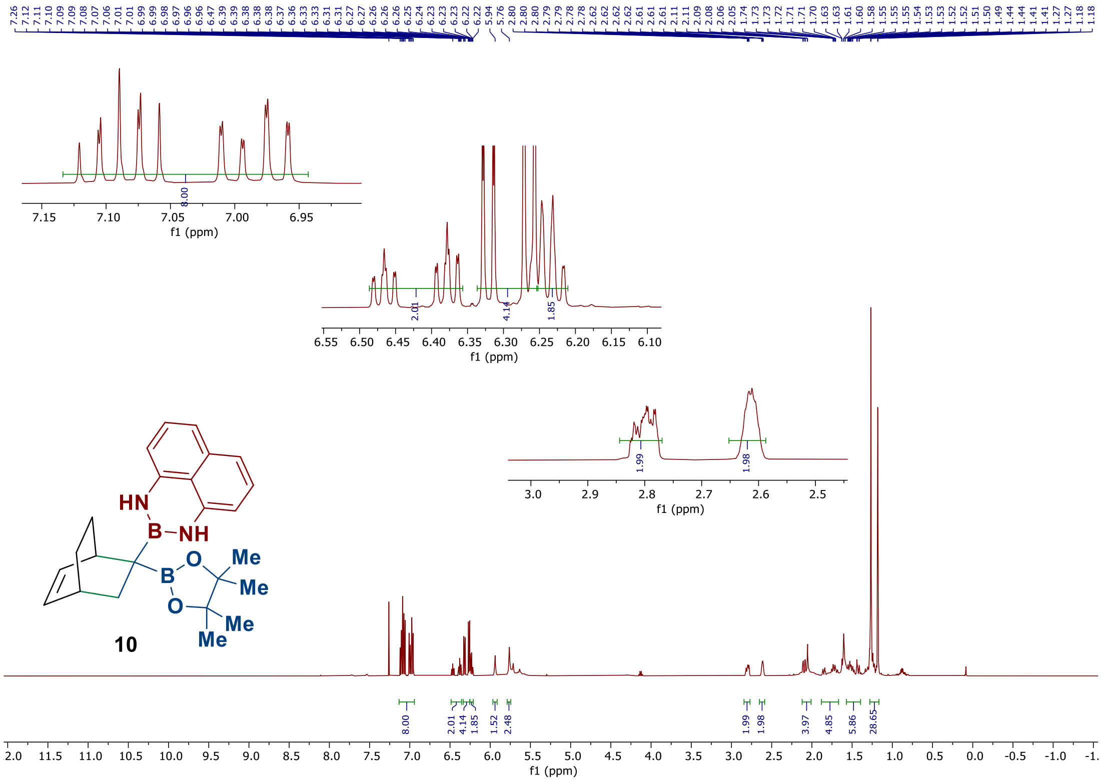

Supplementary Figure 283. ^{1}H NMR (400 MHz, $CDCl_3$) of compound (**10**).

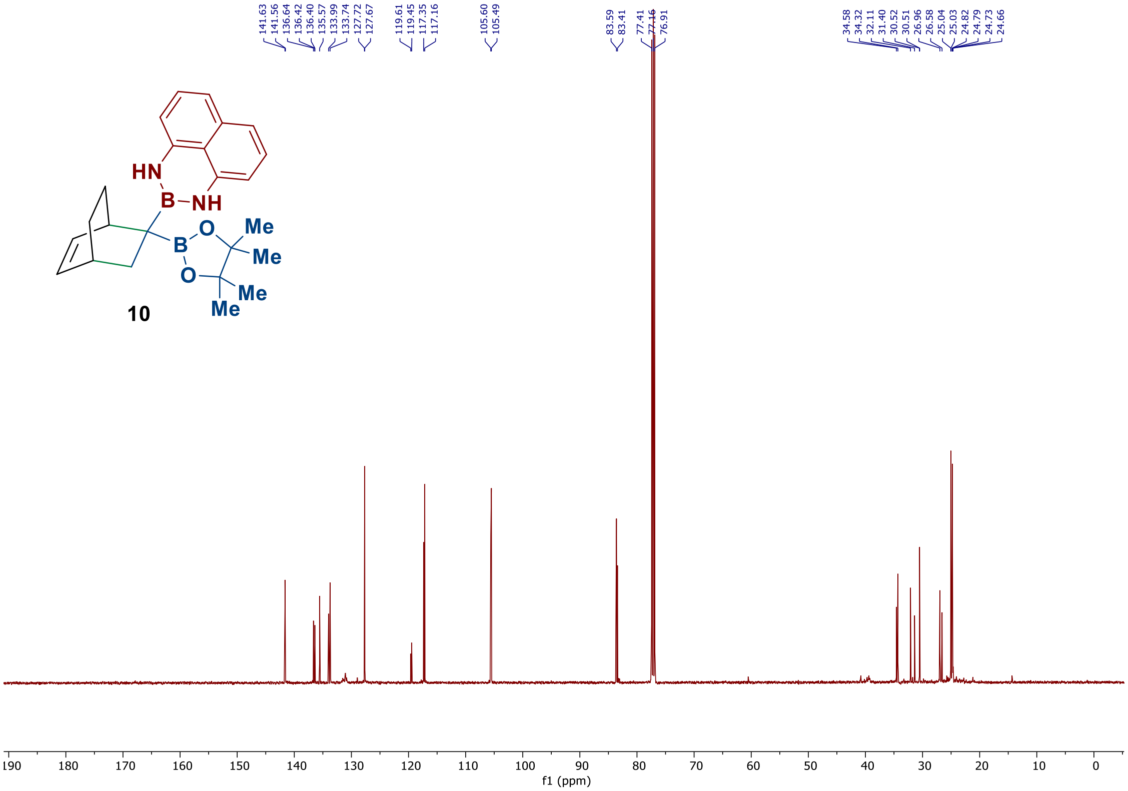

Supplementary Figure 284. ^{13}C NMR (101 MHz, $CDCl_3$) of compound (**10**).

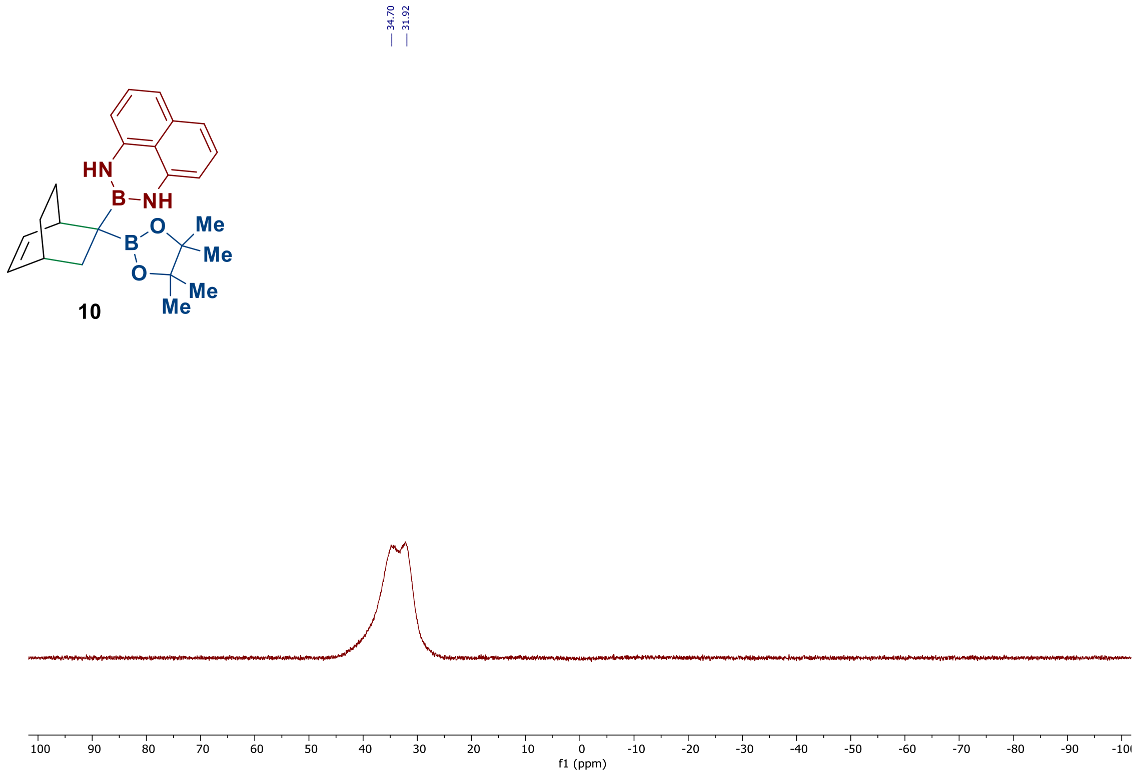

Supplementary Figure 285. ^{11}B NMR (128 MHz, $CDCl_3$) of compound (**10**).

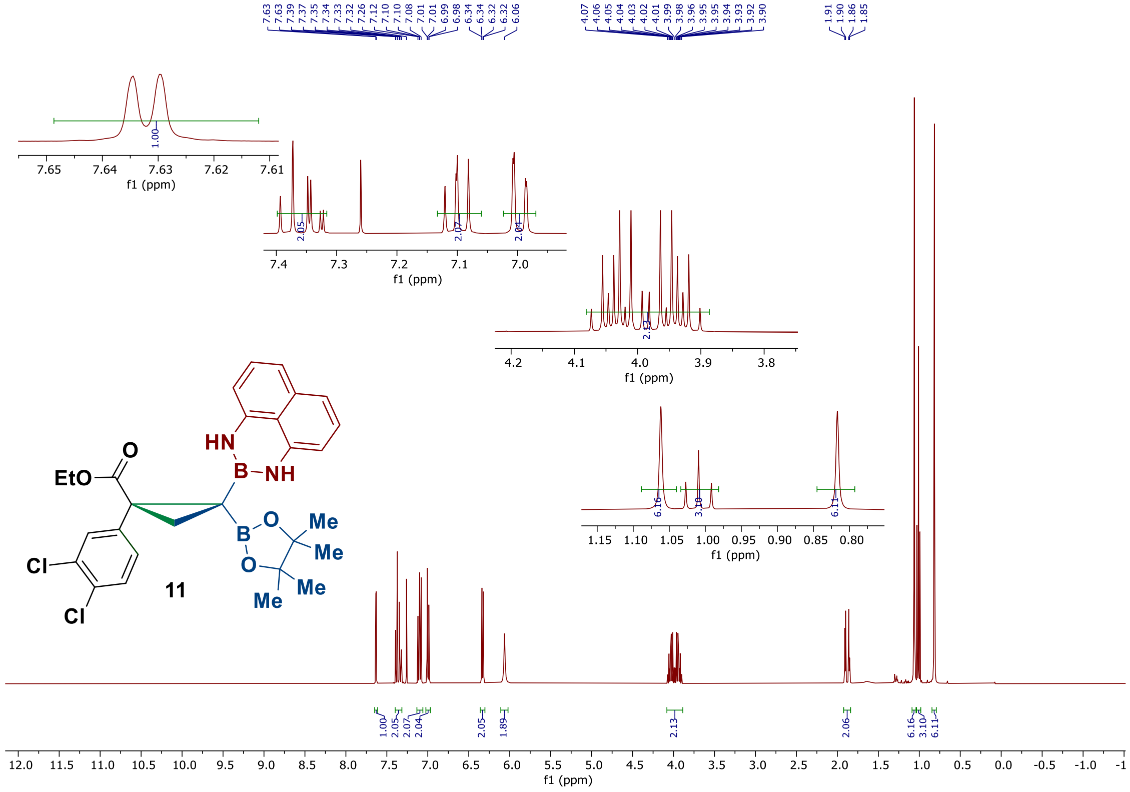

Supplementary Figure 286. ^{1}H NMR (400 MHz, $CDCl_3$) of compound (**11**).

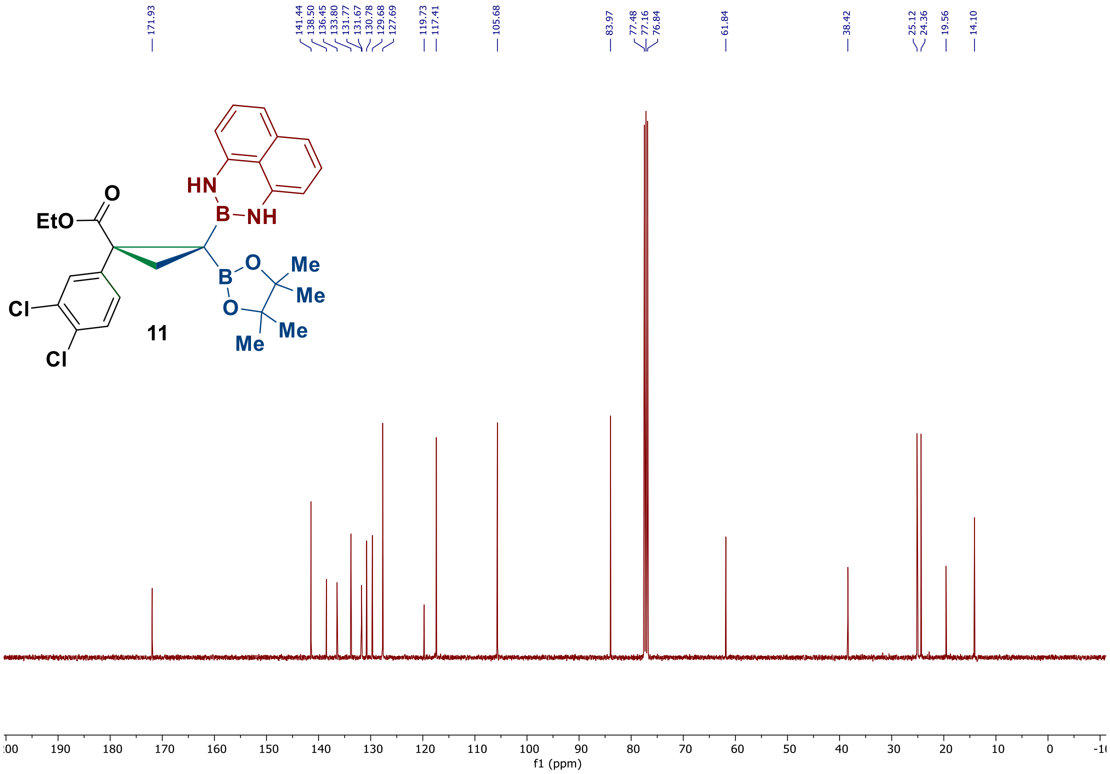

Supplementary Figure 287. ^{13}C NMR (101 MHz, $CDCl_3$) of compound (**11**).

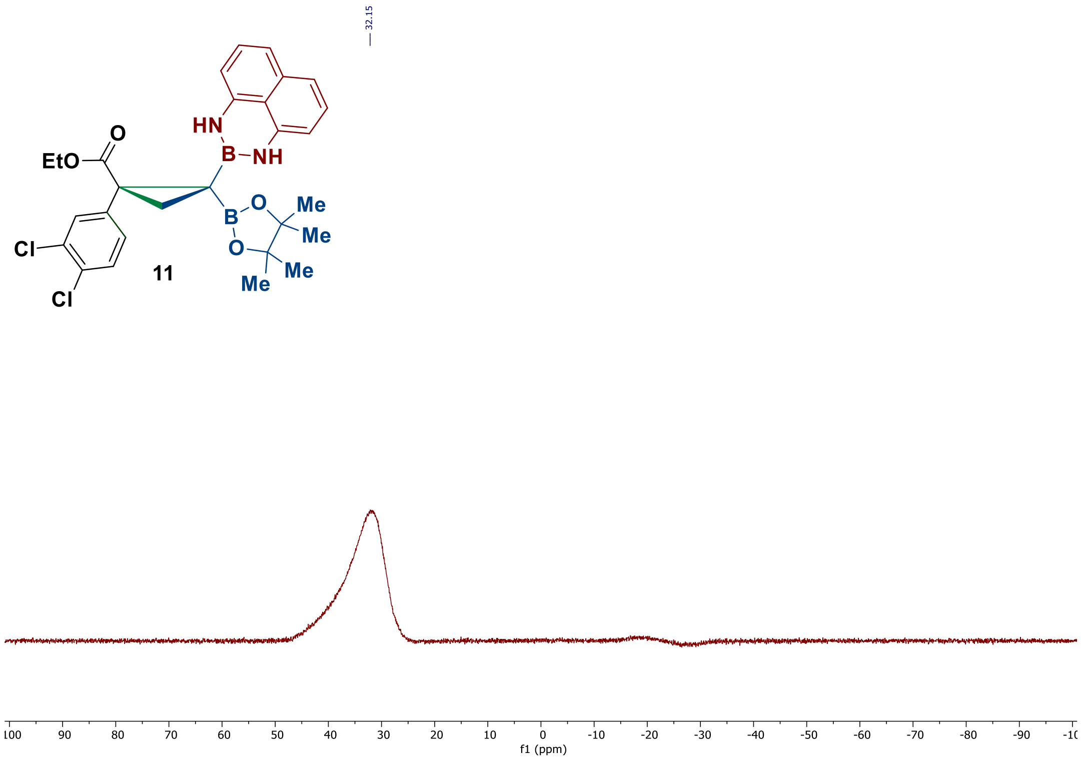

Supplementary Figure 288. ^{11}B NMR (128 MHz, $CDCl_3$) of compound (**11**).

3.3 2D-NMR NOESY Analysis

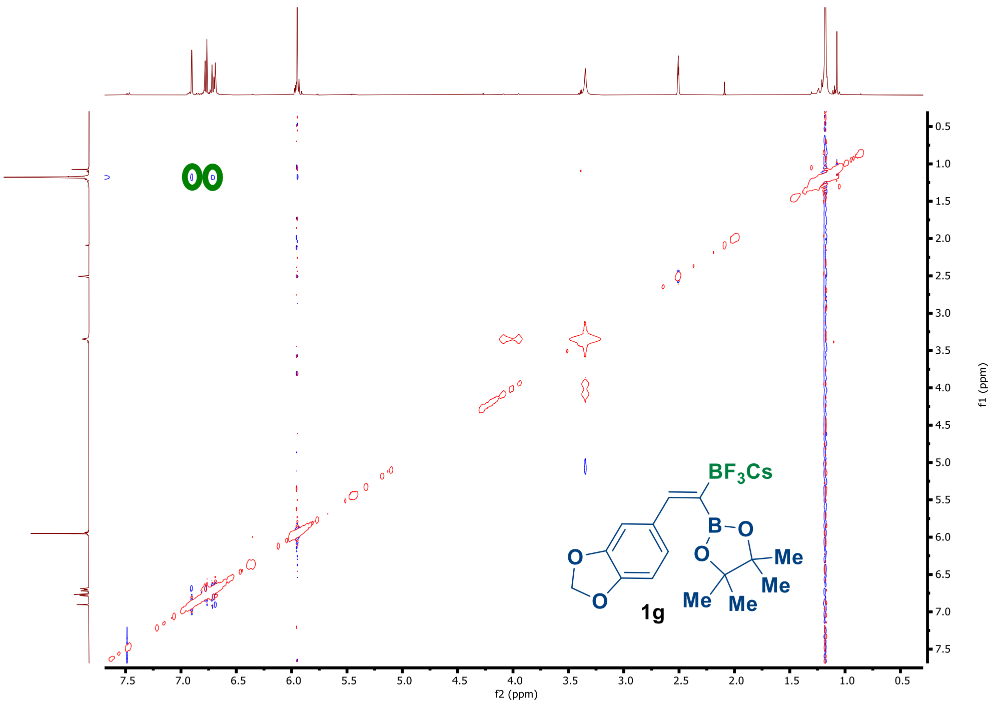

Supplementary Figure 289. 2D-NMR NOESY Analysis for Compound **1g** in (DMSO-d_6).

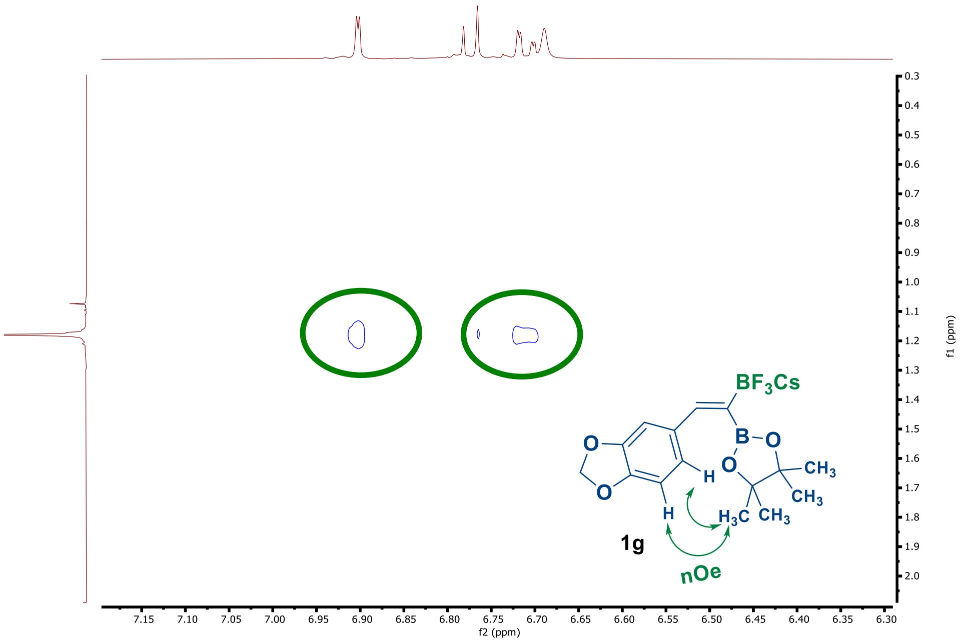

Supplementary Figure 290. Expansion of 2D-NMR NOESY Analysis for Compound **1g** in (DMSO-d_6).

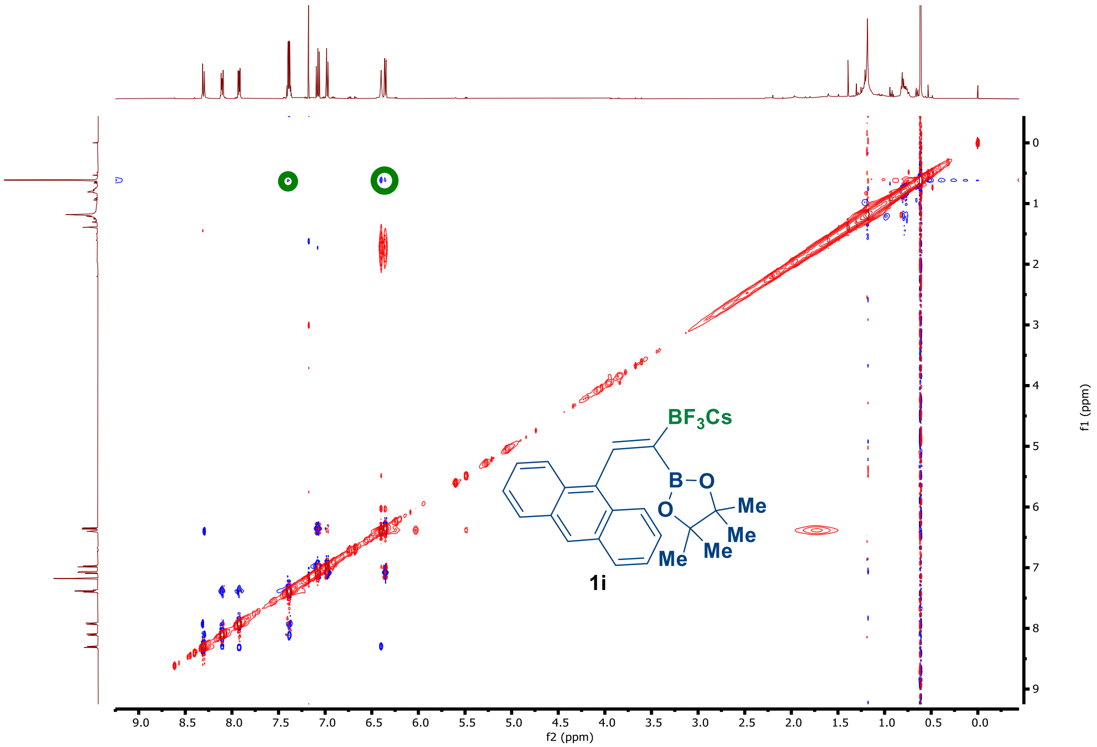

Supplementary Figure 291. 2D-NMR NOESY Analysis for Compound **1i** in (DMSO-d_6).

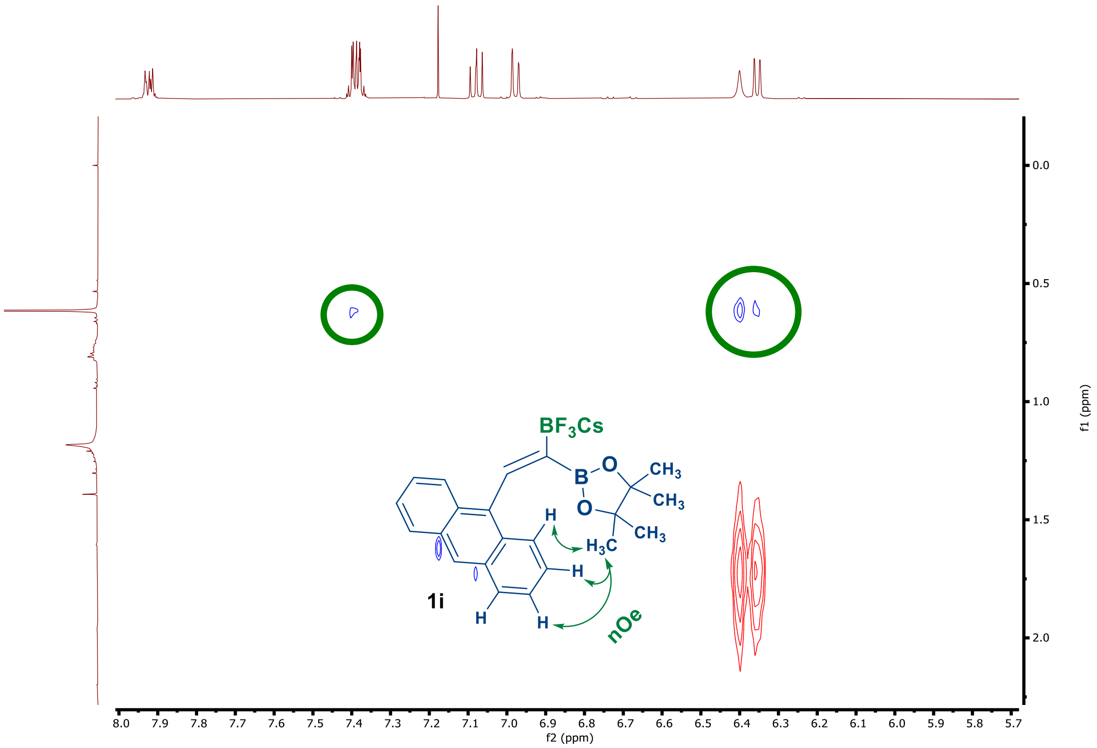

Supplementary Figure 292. Expansion of 2D-NMR NOESY Analysis for Compound **1i** in (DMSO-d_6).

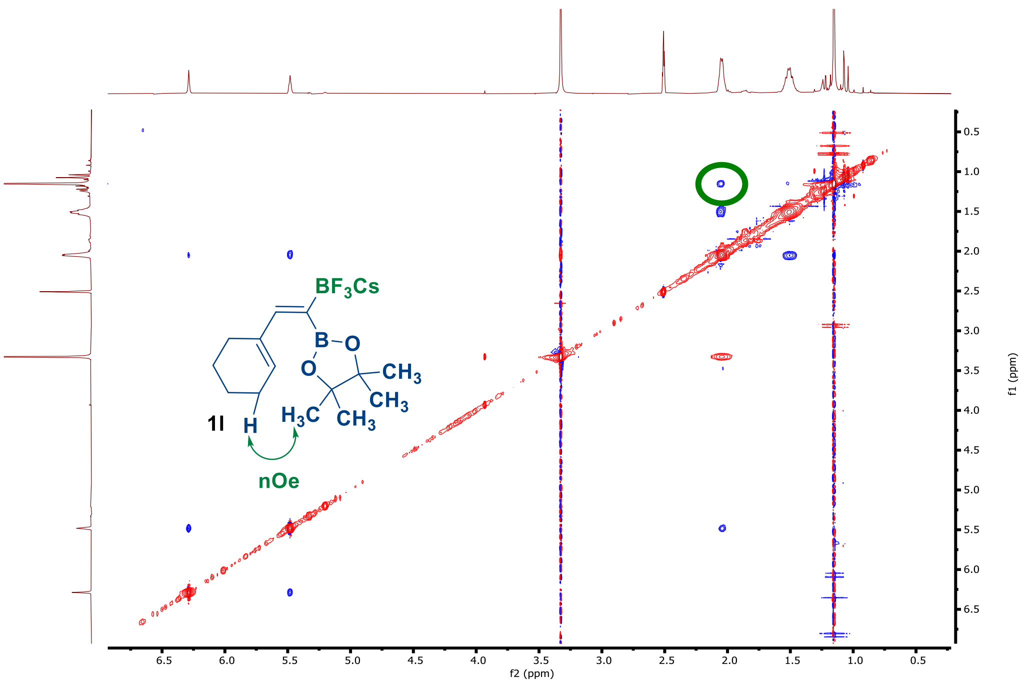

Supplementary Figure 293. 2D-NMR NOESY Analysis for Compound **1l** in (DMSO-d_6).

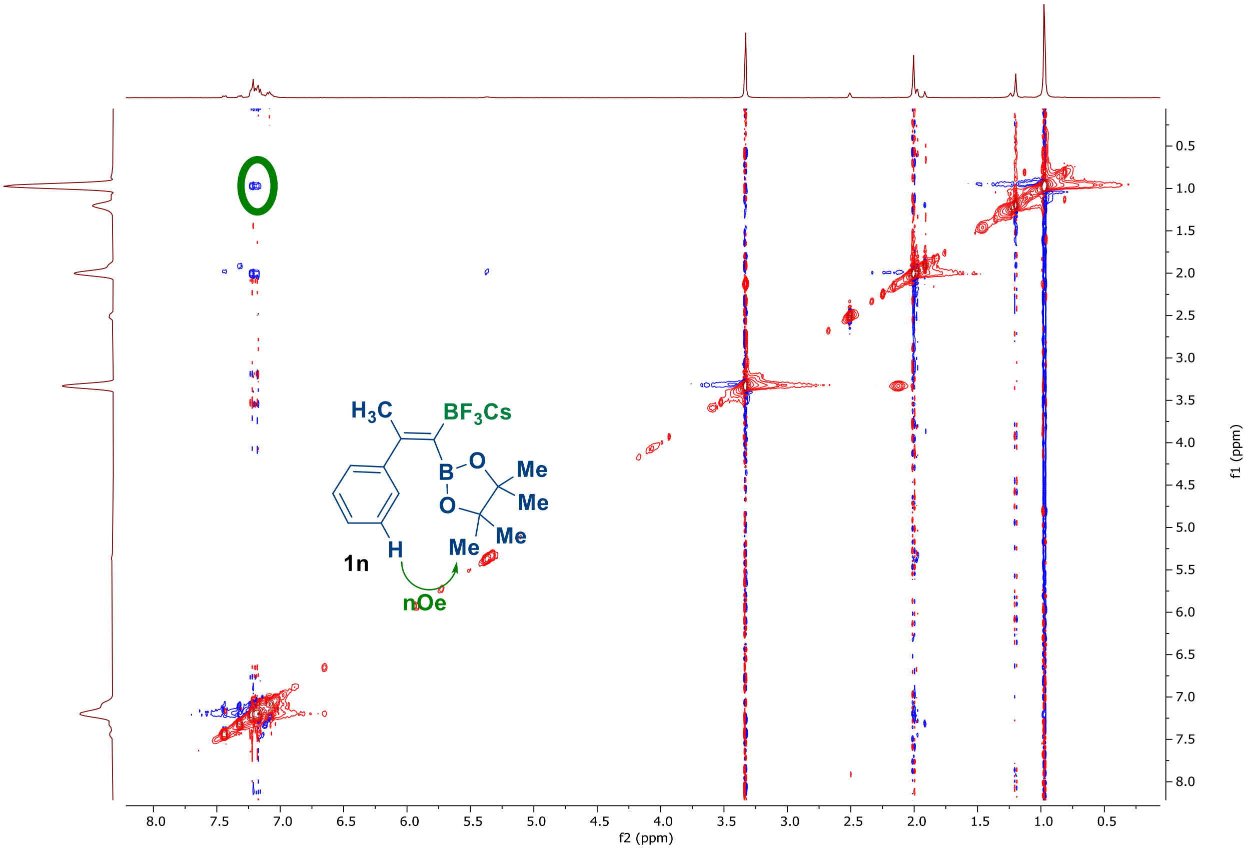

Supplementary Figure 294. 2D-NMR NOESY Analysis for Compound **1n** in (DMSO-d_6).

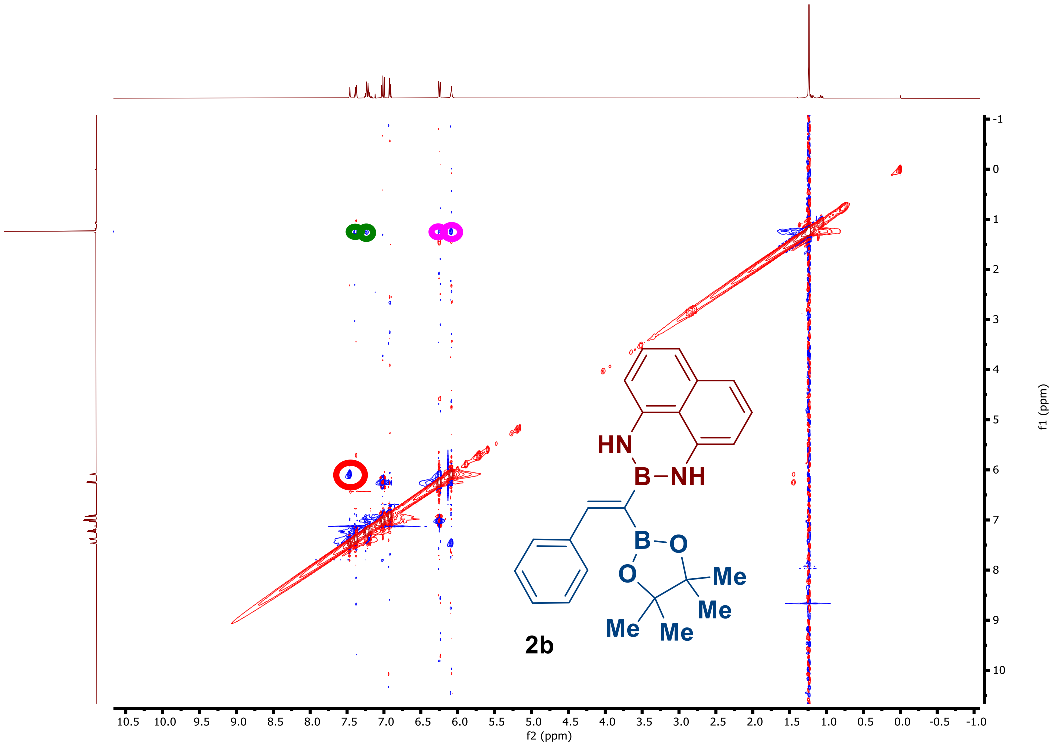

Supplementary Figure 295. 2D-NMR NOESY Analysis for Compound **2b** in ($CDCl_3$).

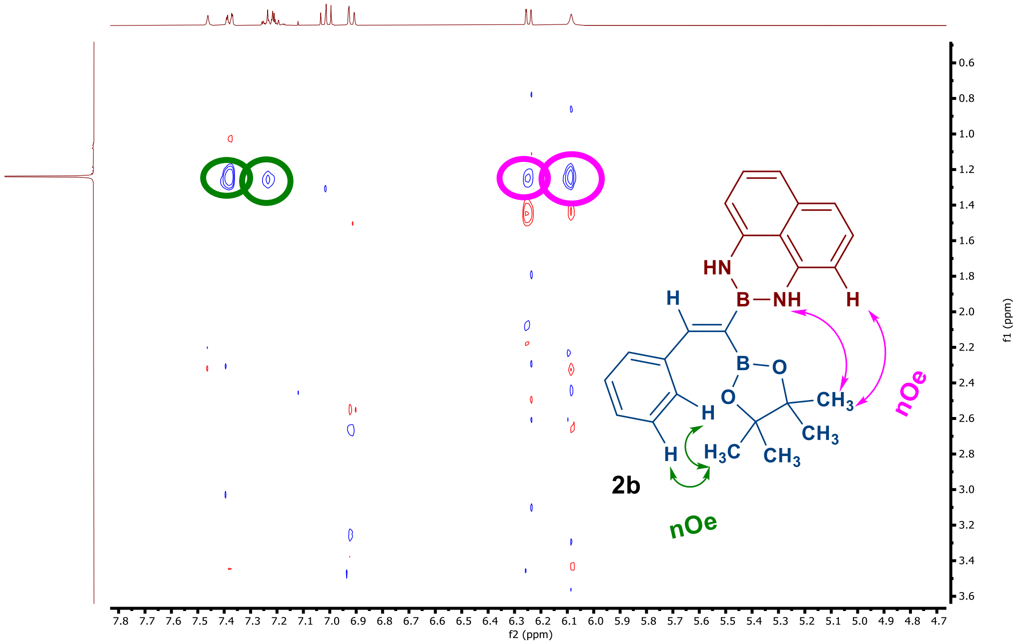

Supplementary Figure 296. Expansion of 2D-NMR NOESY Analysis for Compound **2b** in ($CDCl_3$).

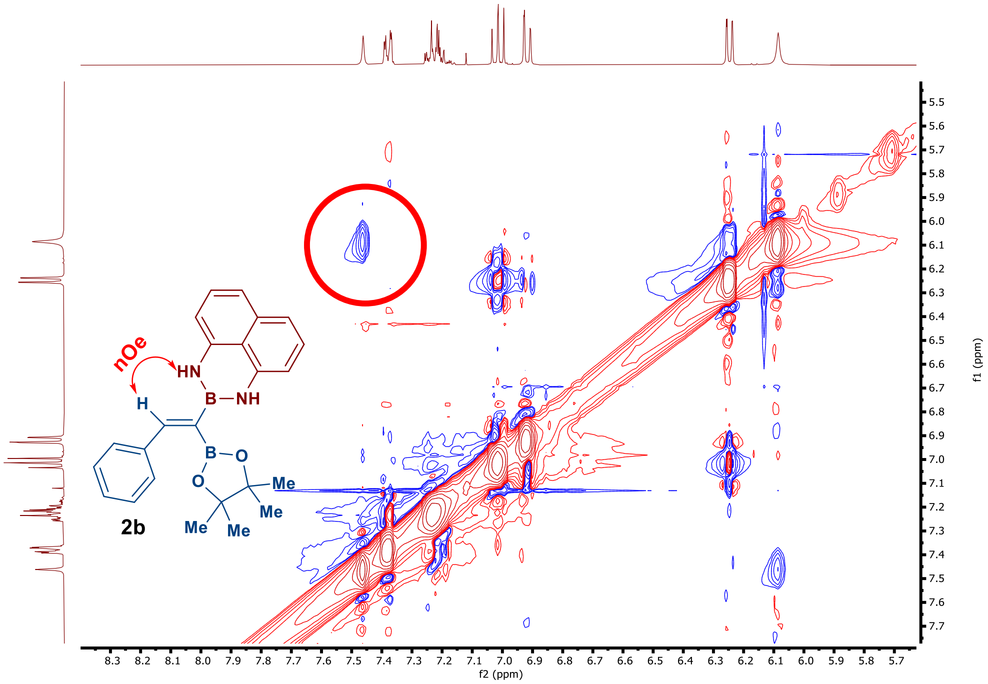

Supplementary Figure 297. Expansion of 2D-NMR NOESY Analysis for Compound **2b** in ($CDCl_3$).

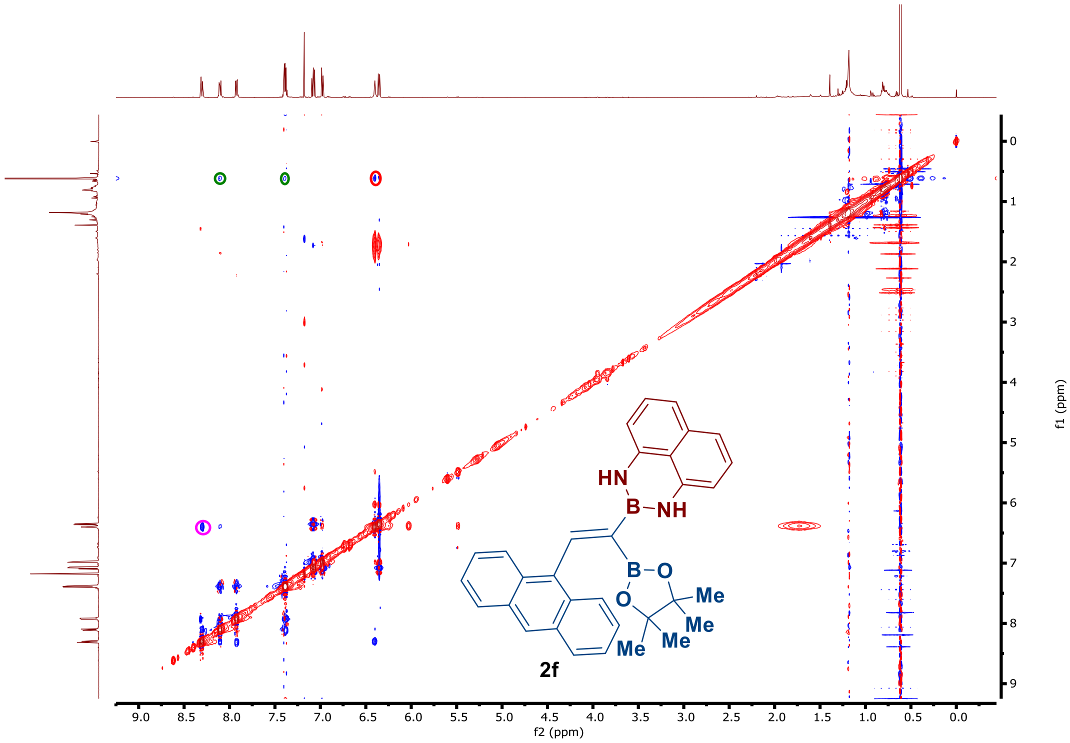

Supplementary Figure 298. 2D-NMR NOESY Analysis for Compound **2f** in ($CDCl_3$).

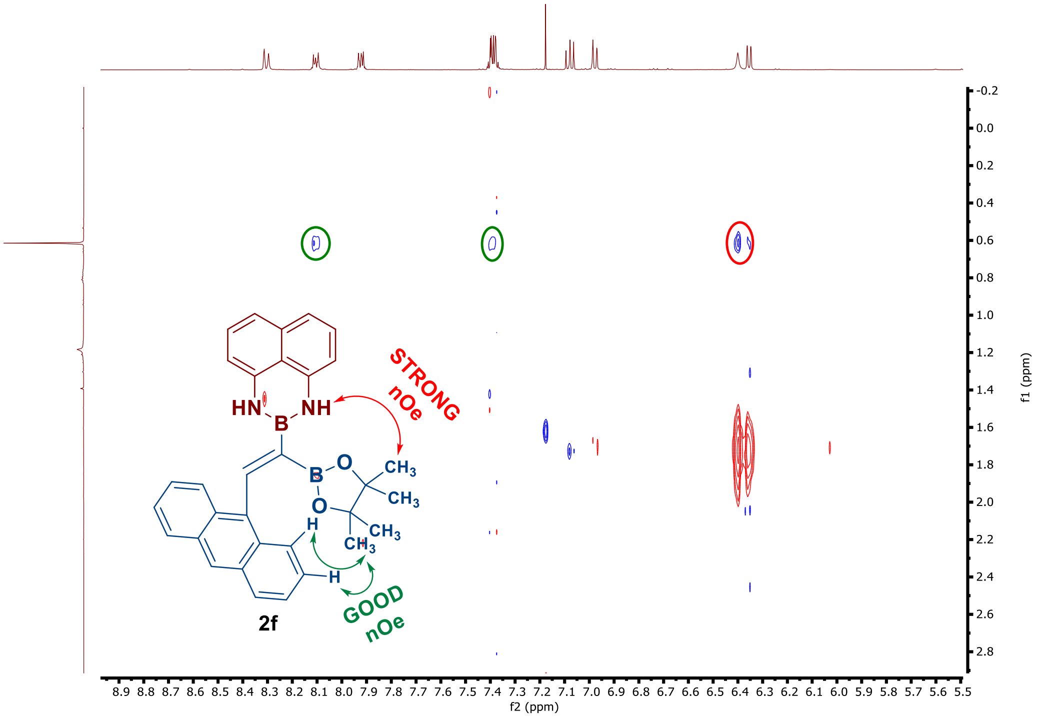

Supplementary Figure 299. Expansion of 2D-NMR NOESY Analysis for Compound **2f** in ($CDCl_3$).

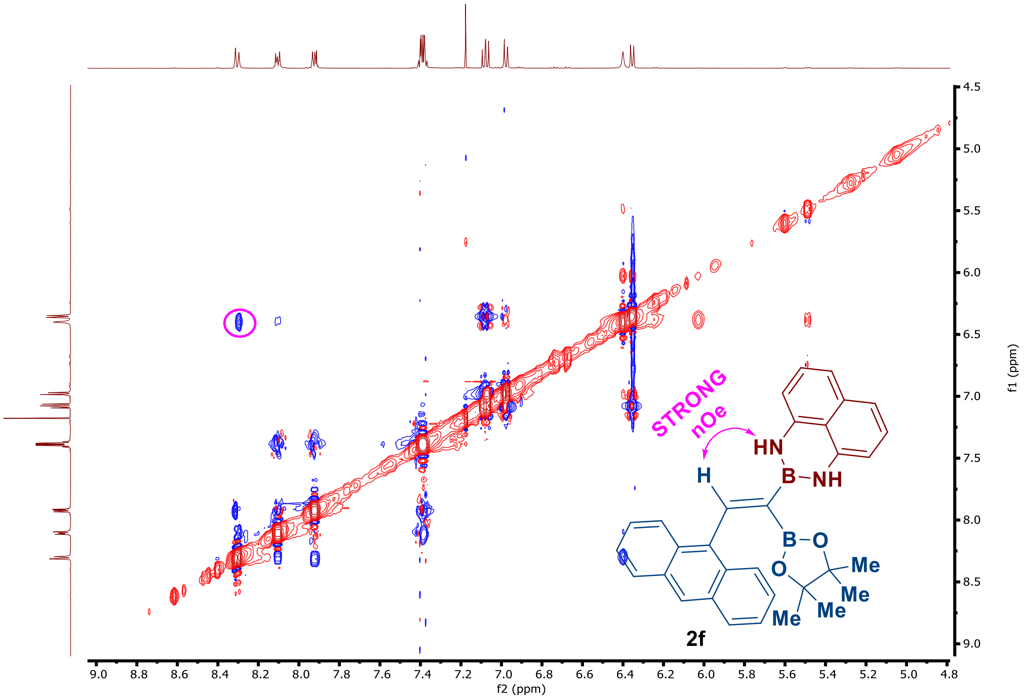

Supplementary Figure 300. Expansion of 2D-NMR NOESY Analysis for Compound **2f** in ($CDCl_3$).

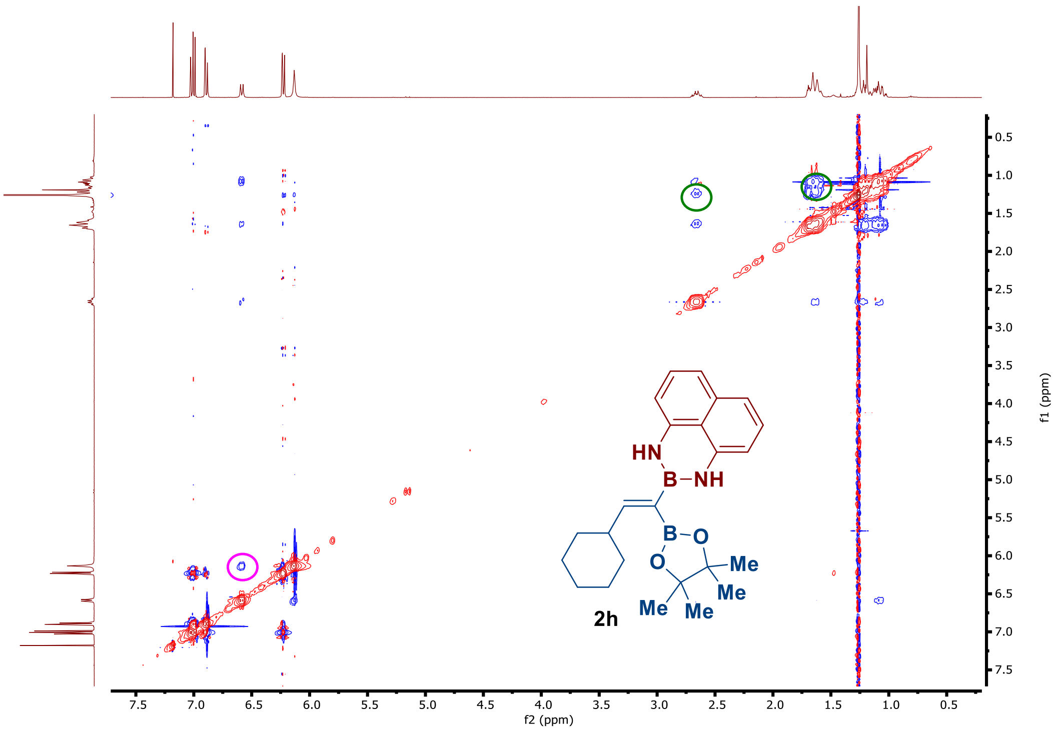

Supplementary Figure 301. 2D-NMR NOESY Analysis for Compound **2h** in ($CDCl_3$).

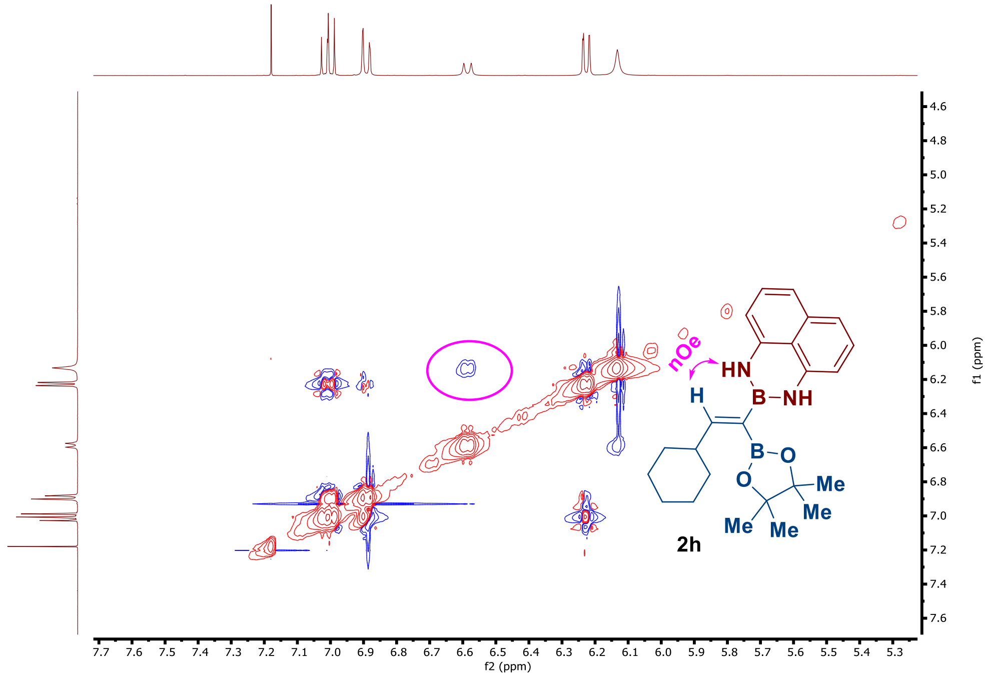

Supplementary Figure 302. Expansion of 2D-NMR NOESY Analysis for Compound **2h** in ($CDCl_3$).

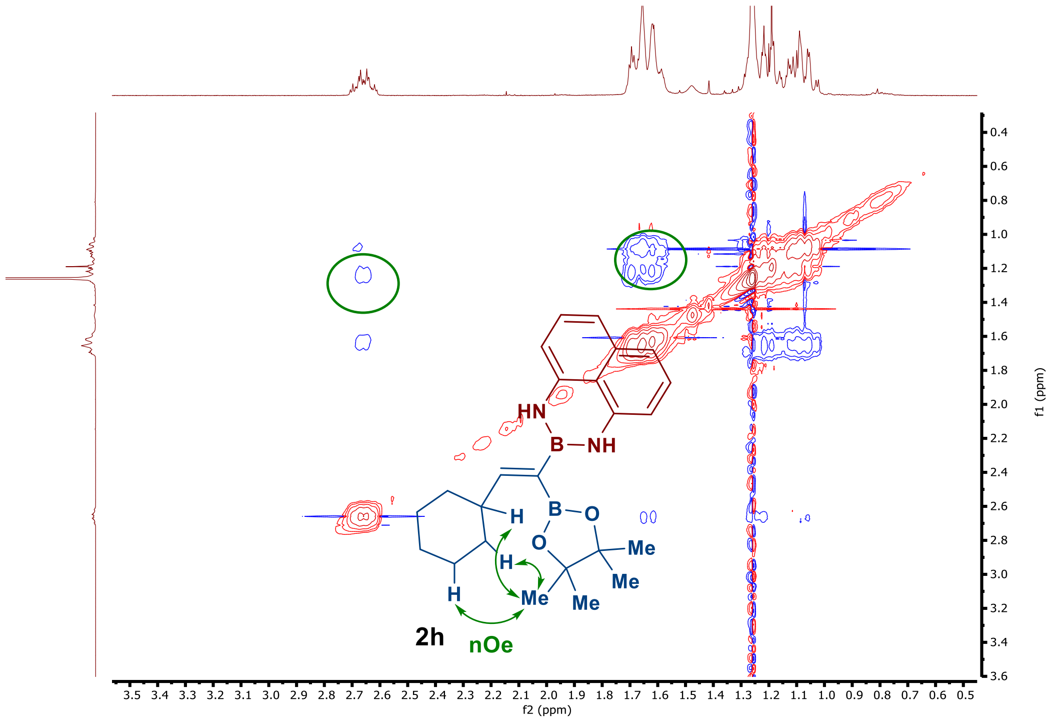

Supplementary Figure 303. Expansion of 2D-NMR NOESY Analysis for Compound **2h** in ($CDCl_3$).

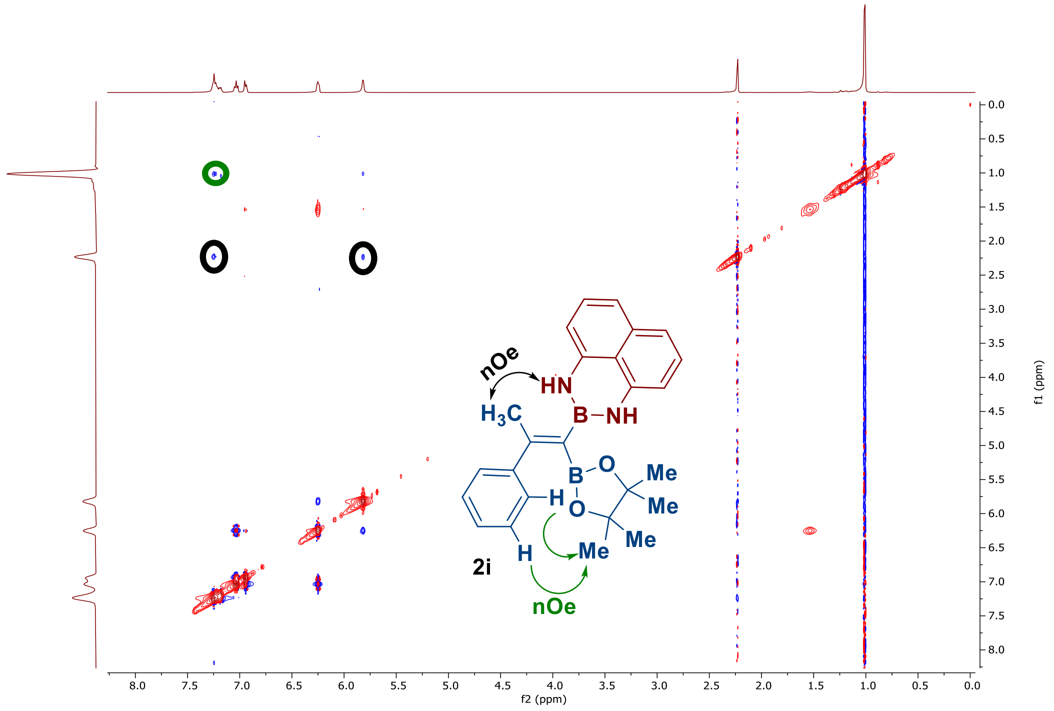

Supplementary Figure 304. 2D-NMR NOESY Analysis for Compound **2i** in ($CDCl_3$).

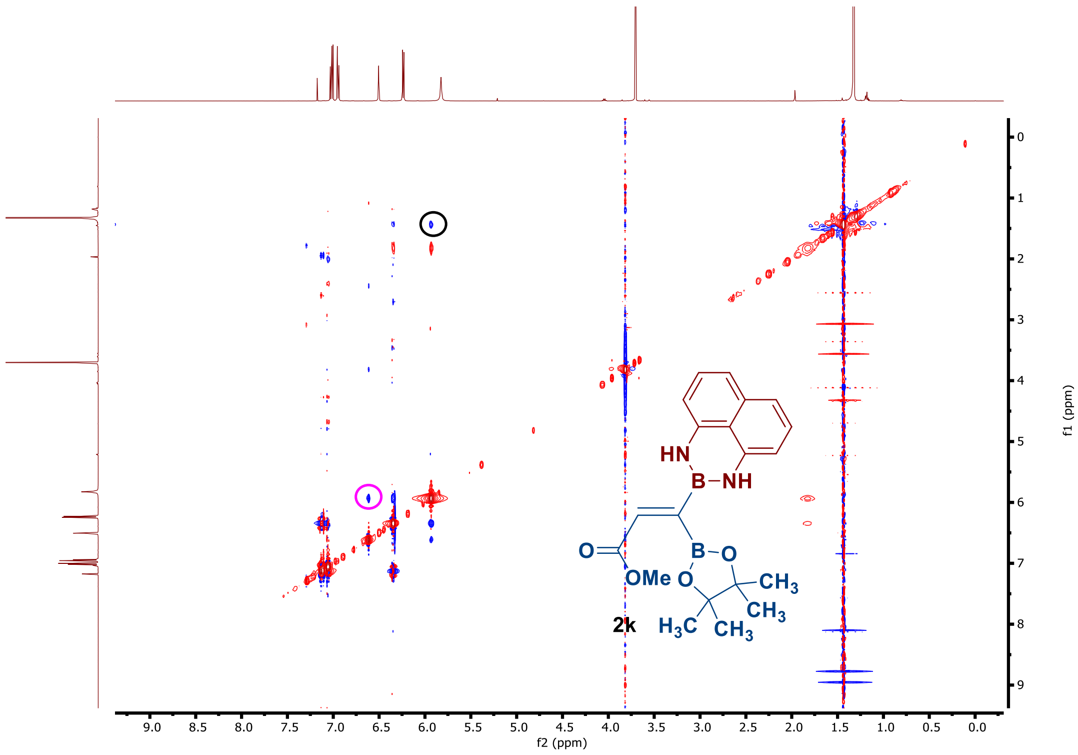

Supplementary Figure 305. 2D-NMR NOESY Analysis for Compound **2k** in ($CDCl_3$).

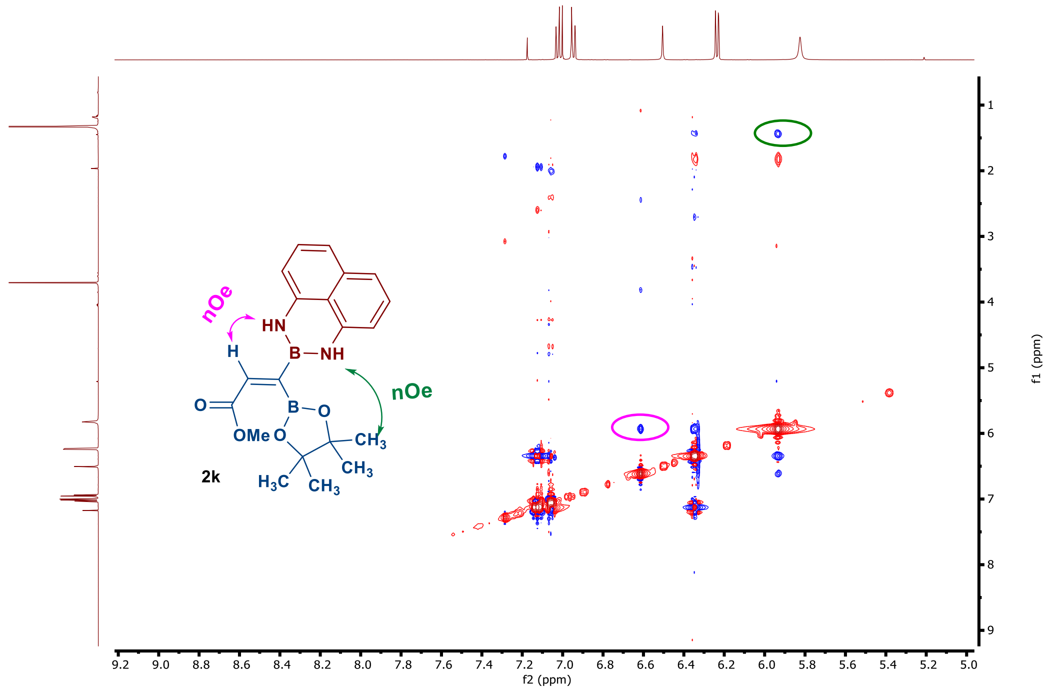

Supplementary Figure 306. Expansion of 2D-NMR NOESY Analysis for Compound **2k** in ($CDCl_3$).

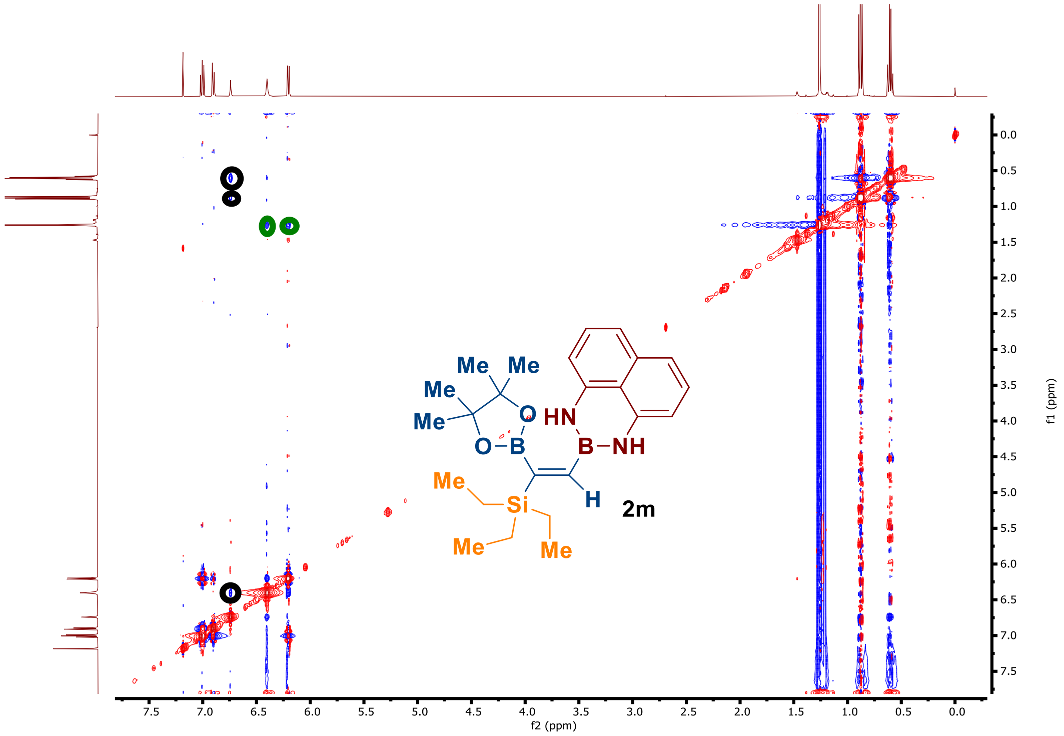

Supplementary Figure 307. 2D-NMR NOESY Analysis for Compound **2m** in ($CDCl_3$).

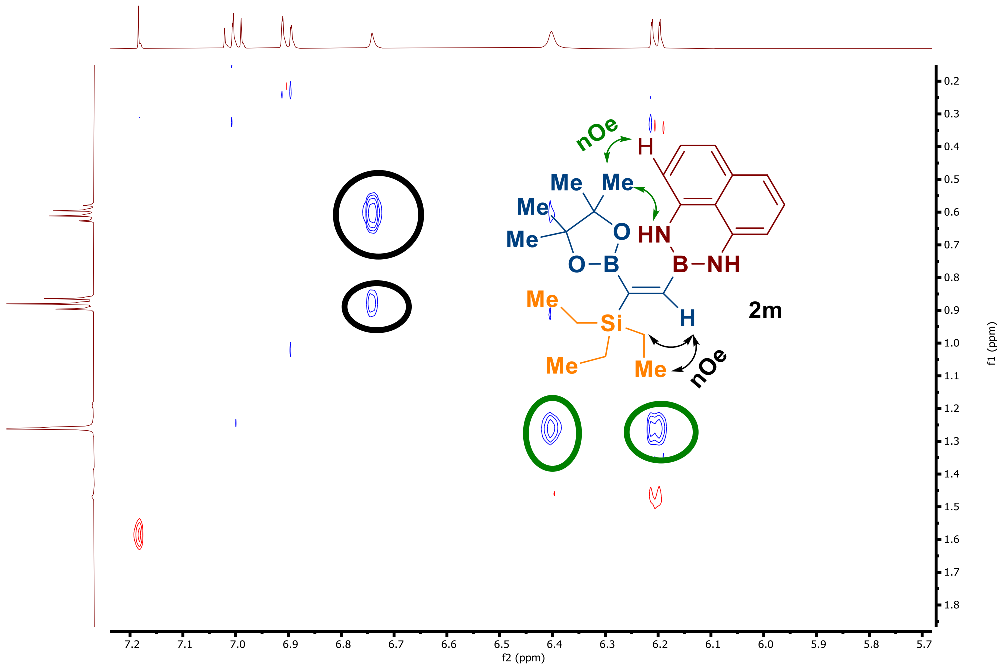

Supplementary Figure 308. Expansion of 2D-NMR NOESY Analysis for Compound **2m** in ($CDCl_3$).

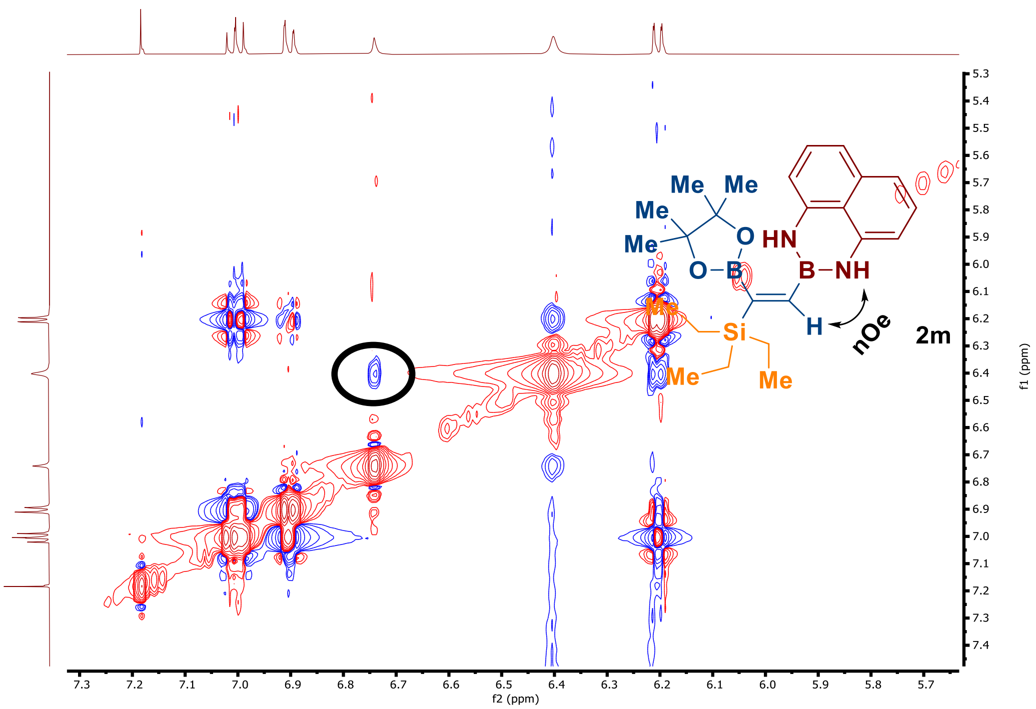

Supplementary Figure 309. Expansion of 2D-NMR NOESY Analysis for Compound **2m** in ($CDCl_3$).

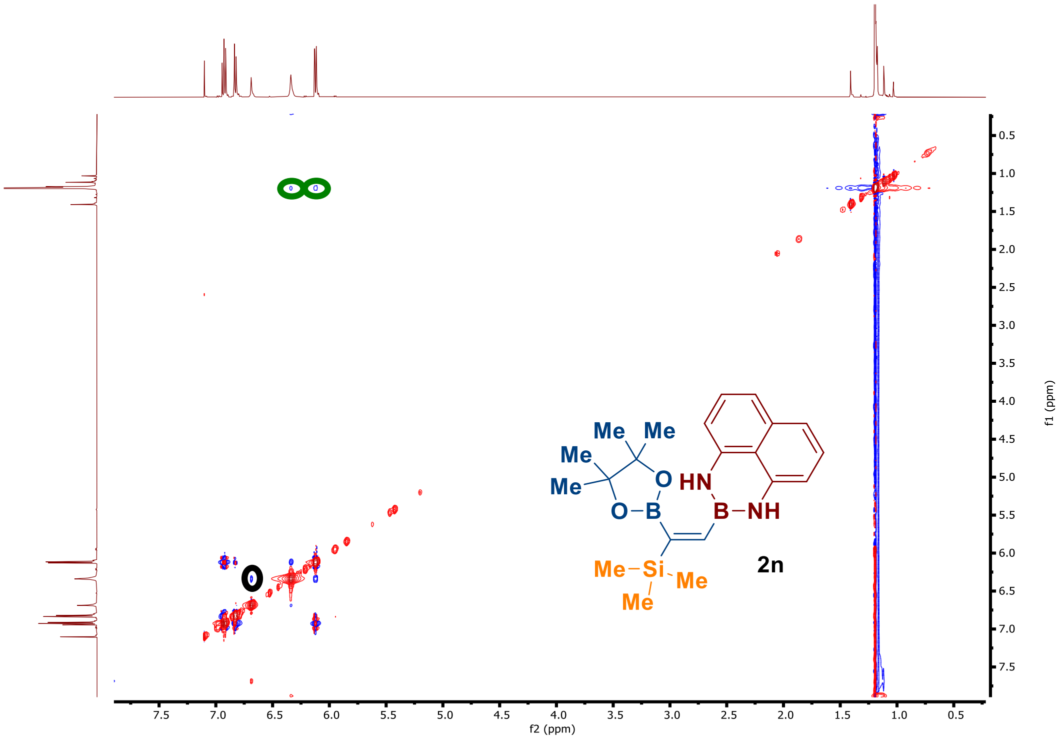

Supplementary Figure 310. Expansion of key protons of 2D-NMR NOESY Analysis for Compound **2n** in ($CDCl_3$).

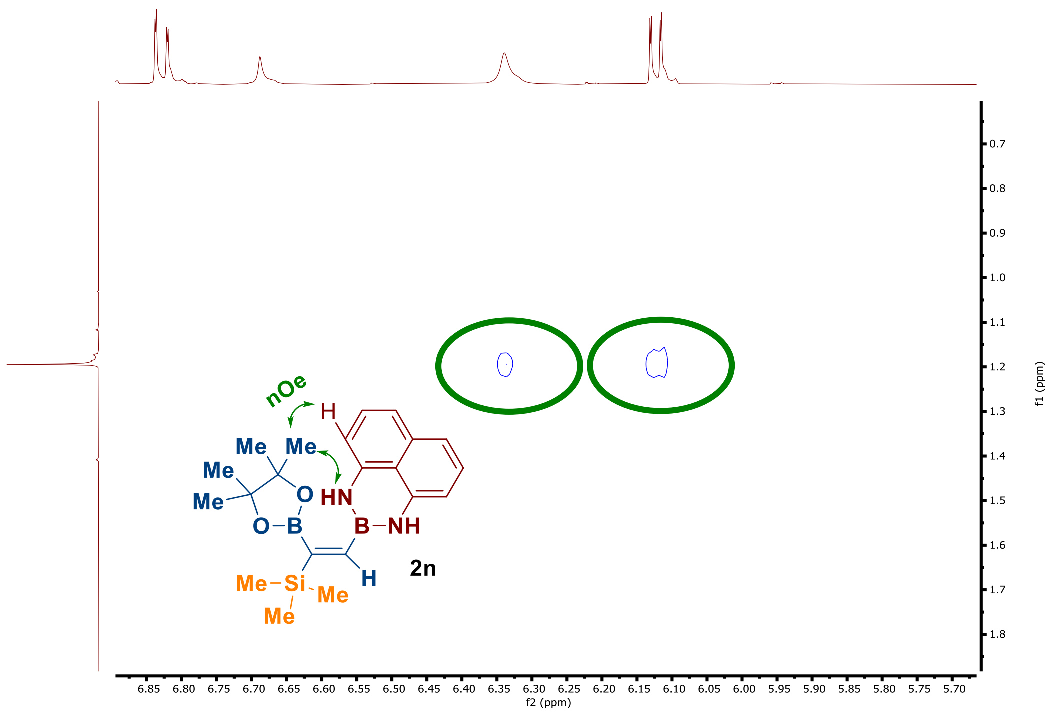

Supplementary Figure 311. 2D-NMR NOESY Analysis for Compound **2n** in ($CDCl_3$).

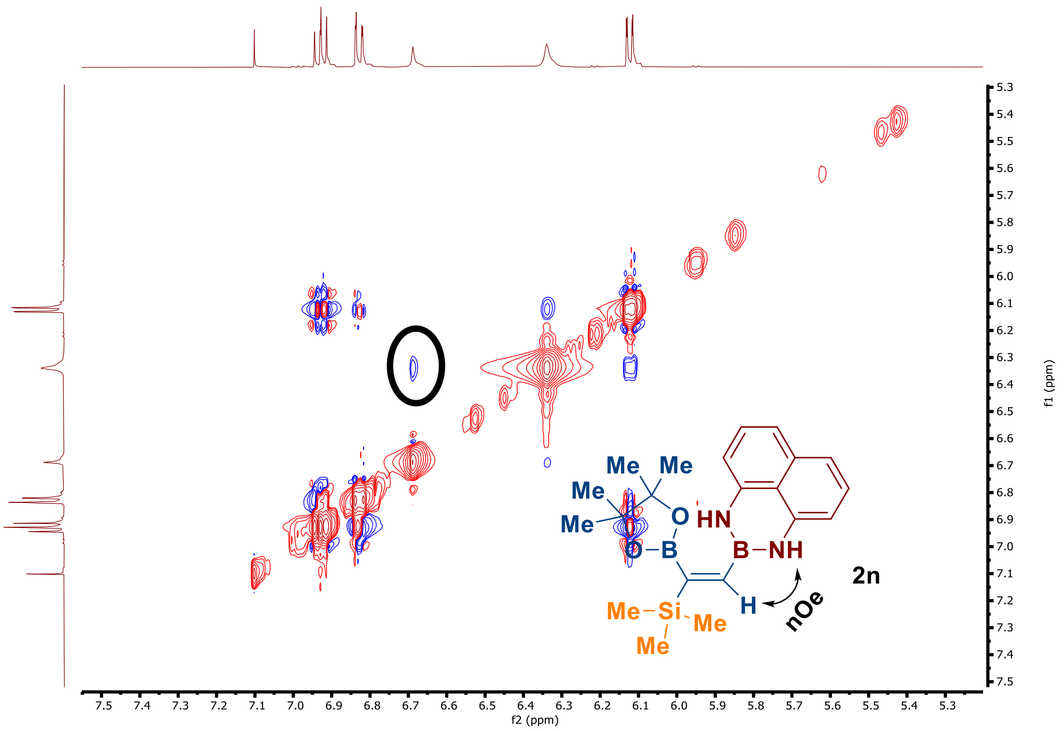

Supplementary Figure 312. Expansion of 2D-NMR NOESY Analysis for Compound **2n** in ($CDCl_3$).

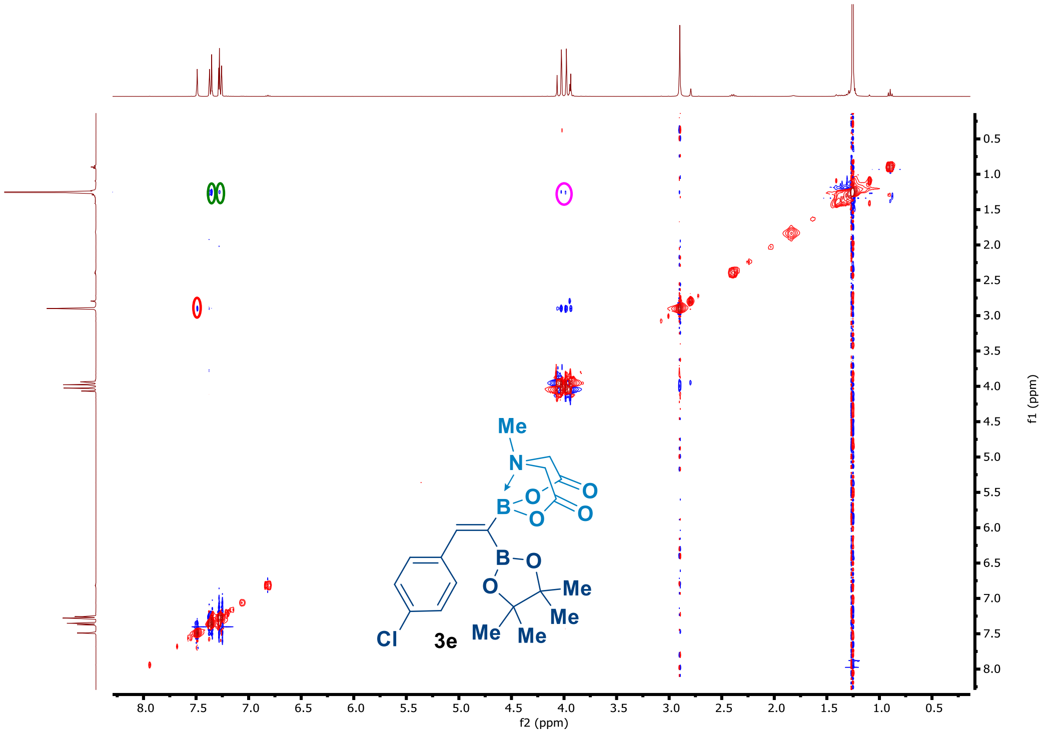

Supplementary Figure 313. 2D-NMR NOESY Analysis for Compound **3e** in ($CDCl_3$).

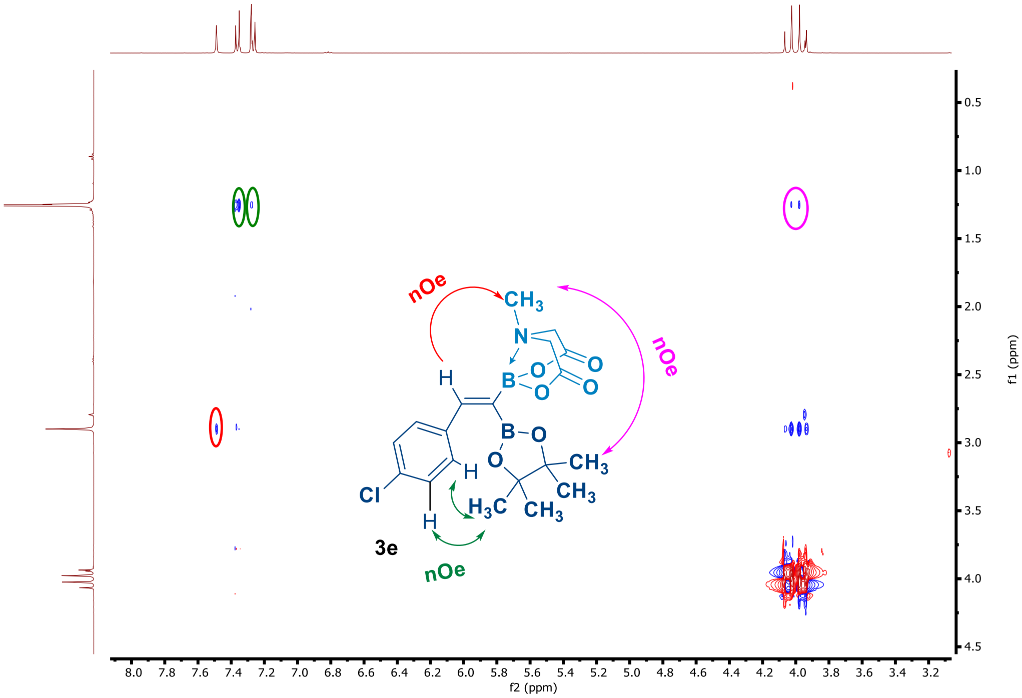

Supplementary Figure 314. Expansion of 2D-NMR NOESY Analysis for Compound **3e** in ($CDCl_3$).

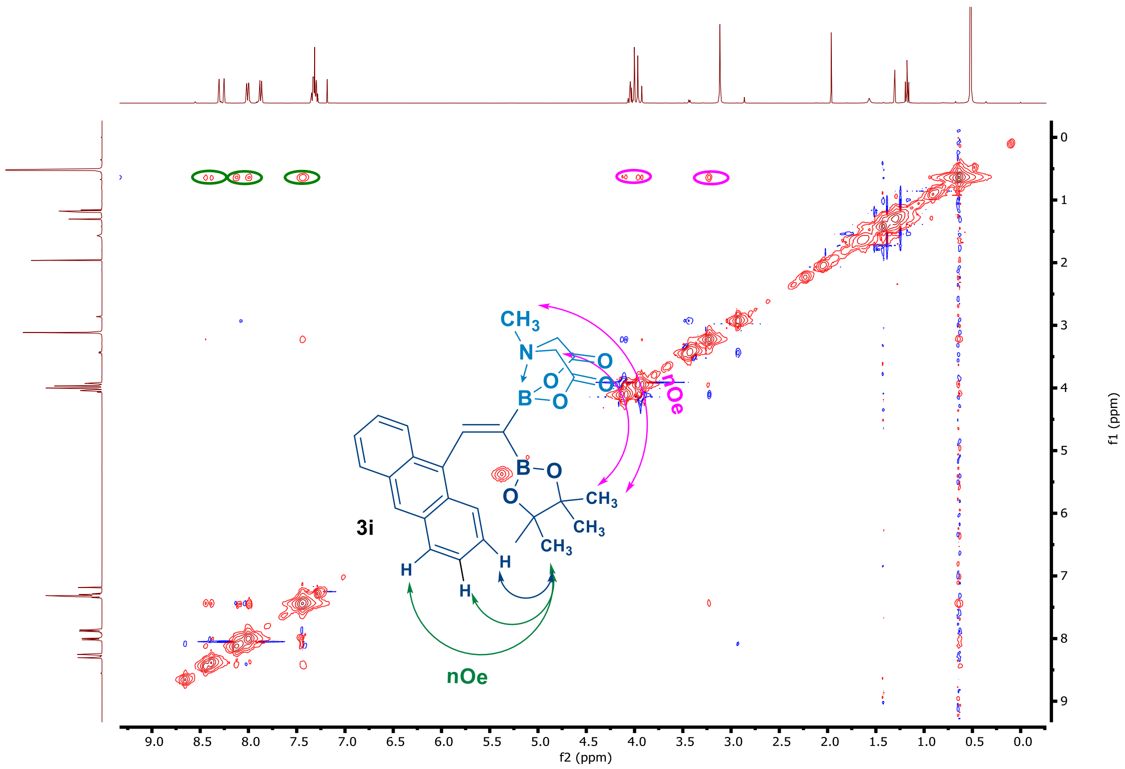

Supplementary Figure 315. 2D-NMR NOESY Analysis for Compound **3i** in ($CDCl_3$).

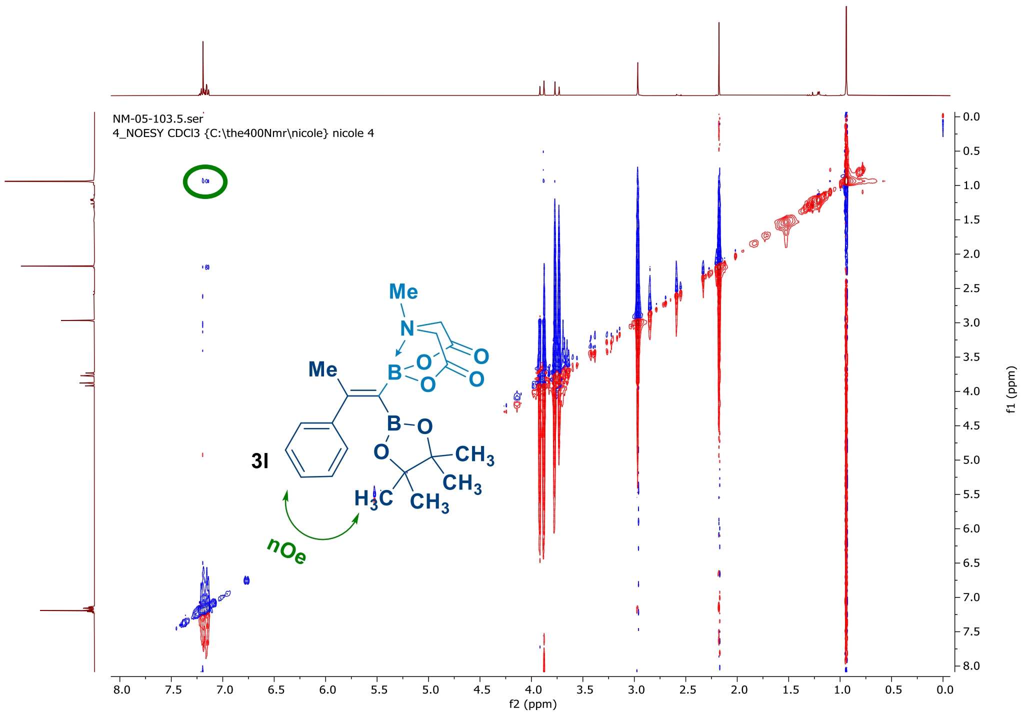

Supplementary Figure 316. 2D-NMR NOESY Analysis for Compound **3l** in ($CDCl_3$).

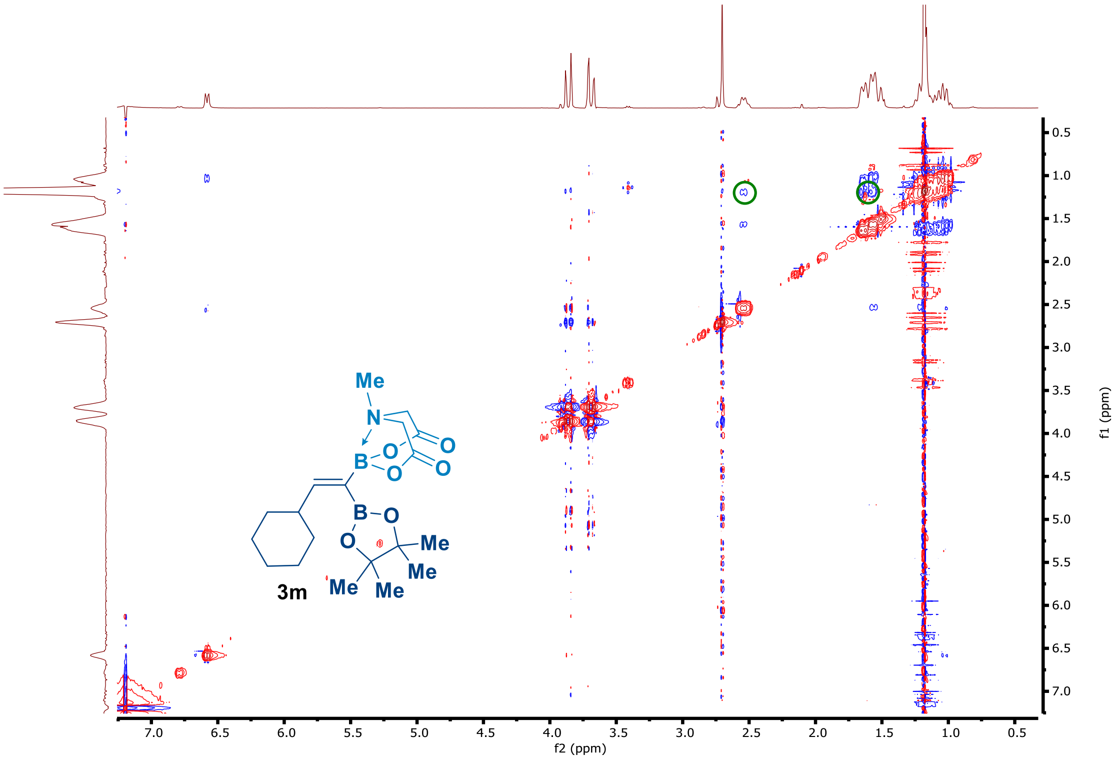

Supplementary Figure 317. 2D-NMR NOESY Analysis for Compound **3m** in ($CDCl_3$).

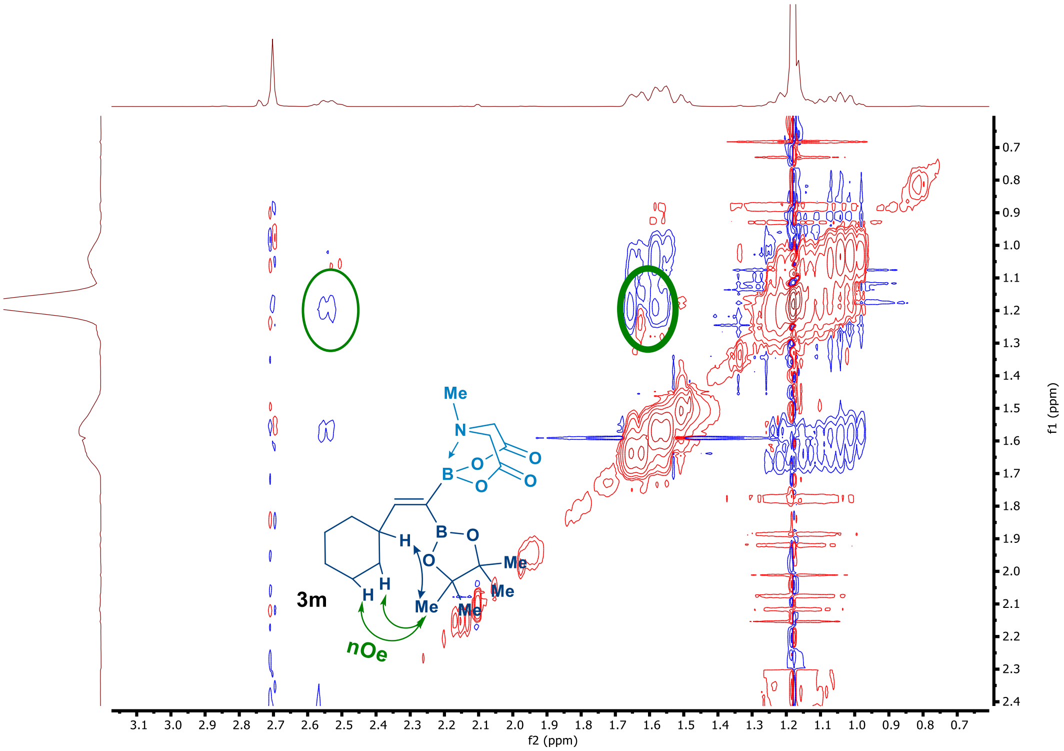

Supplementary Figure 318. Expansion of 2D-NMR NOESY Analysis for Compound **3m** in ($CDCl_3$).

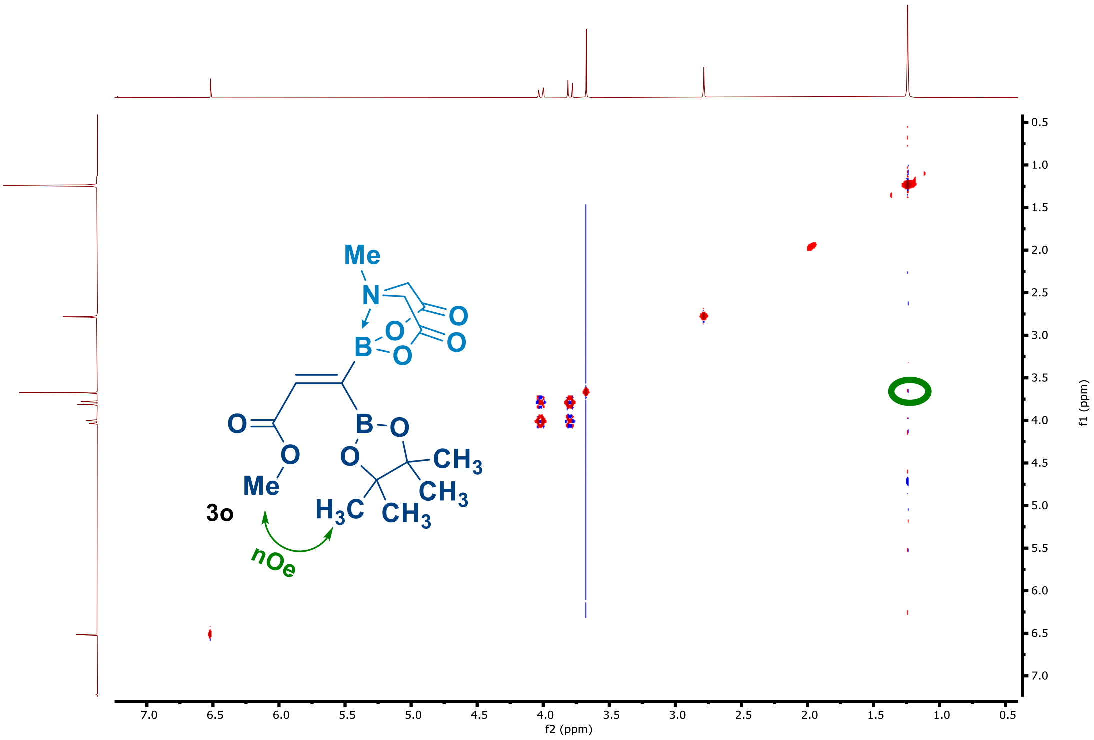

Supplementary Figure 319. 2D-NMR NOESY Analysis for Compound **3o** in ($CDCl_3$).

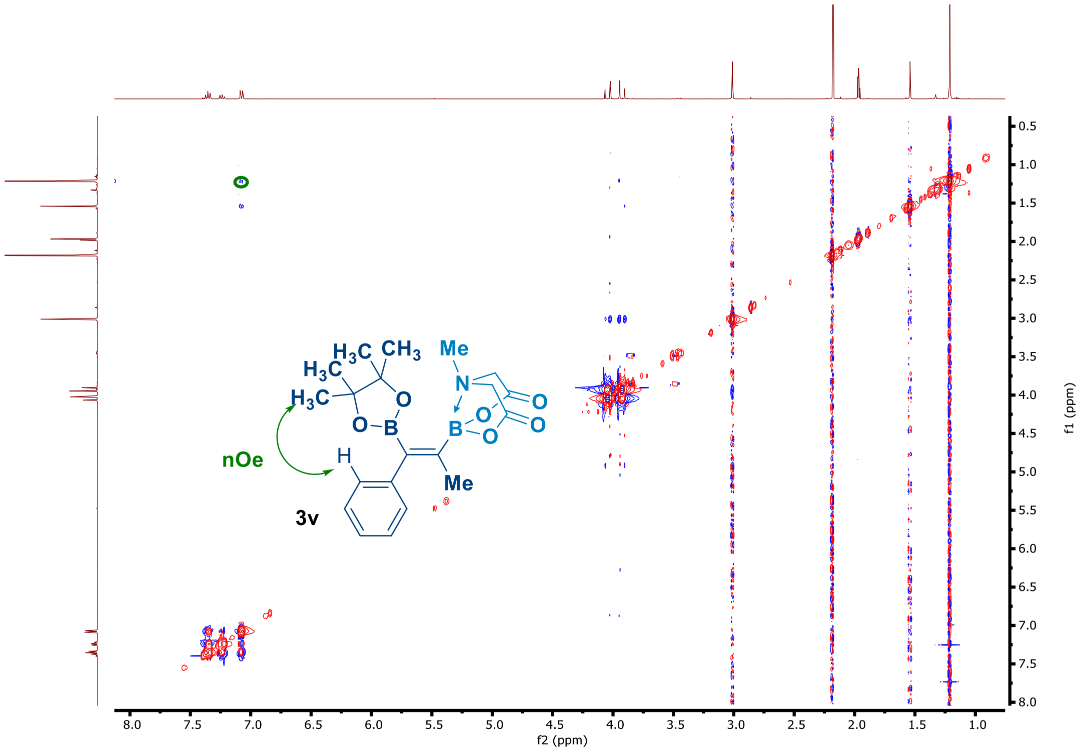

Supplementary Figure 320. 2D-NMR NOESY Analysis for Compound **3v** in ($CDCl_3$).

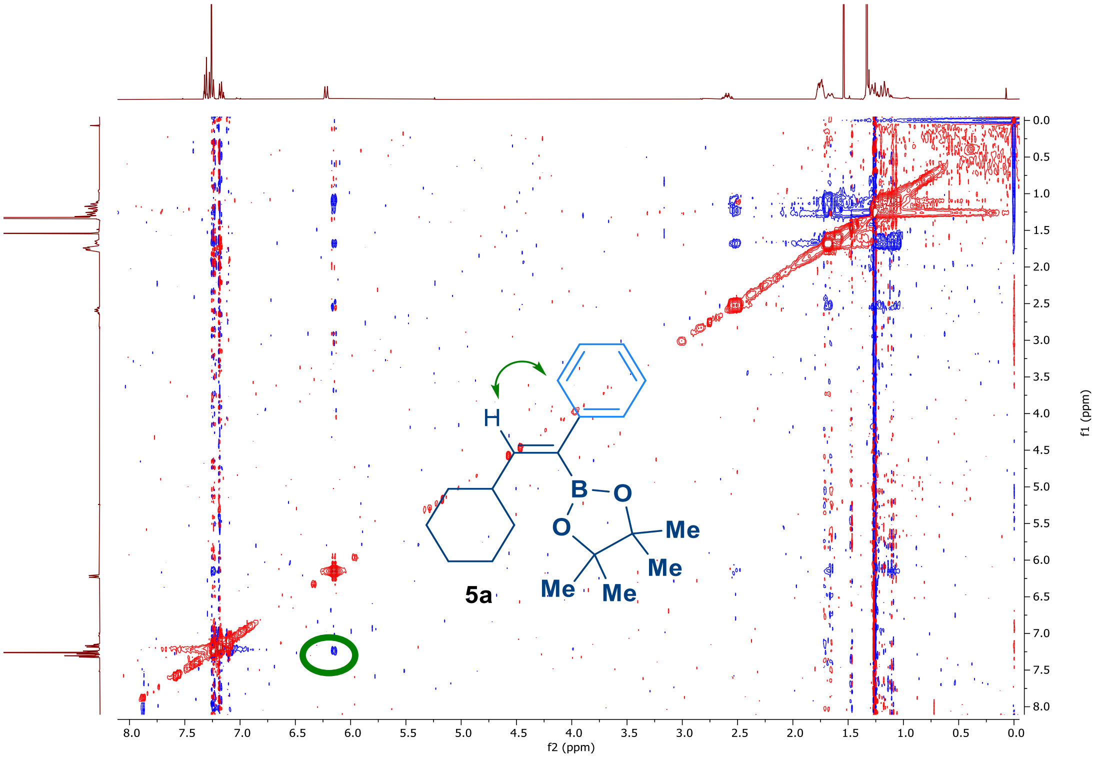

Supplementary Figure 321. 2D-NMR NOESY Analysis for Compound **5a** in ($CDCl_3$).

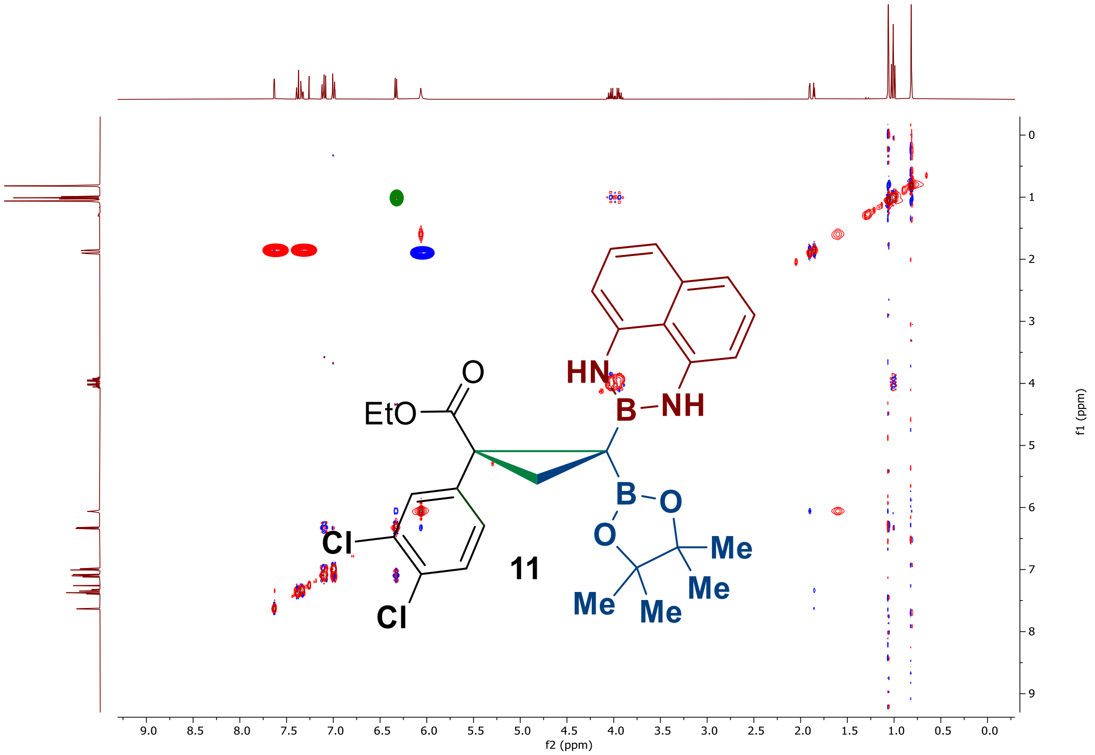

Supplementary Figure 322. 2D-NMR NOESY Analysis for Compound **11** in ($CDCl_3$).

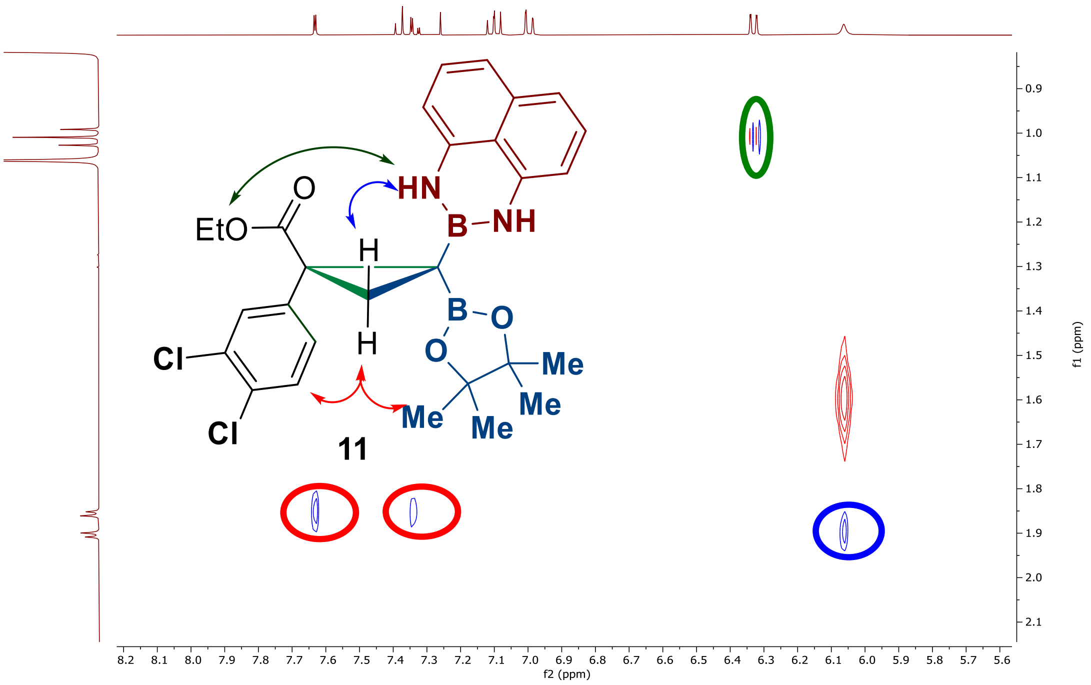

Supplementary Figure 323. Expansion of 2D-NMR NOESY Analysis for Compound **11** in ($CDCl_3$).

4. Supplementary References

1. Platon, A. Multipurpose Crystallographic Tool: AL Spek. *J. Appl. Cryst* **36**, 7 (2003).

2. Eghbarieh, N. *et al.* Stereoselective Diels–Alder reactions of gem-diborylalkenes: toward the synthesis of *gem*-diboron-based polymers. *J. Am. Chem. Soc.* **143**, 6211-6220 (2021).

3. Liu, X., Ming, W., Friedrich, A., Kerner, F. & Marder, T. B. Copper-Catalyzed Triboration of Terminal Alkynes Using B_2pin_2: Efficient Synthesis of 1, 1, 2-Triborylalkenes. *Angew. Chem. Int. Ed.* **59**, 304-309 (2020).

4. Kumar, N., Eghbarieh, N., Stein, T., Shames, A. I. & Masarwa, A. Photoredox-Mediated Reaction of *gem*-Diborylalkenes: Reactivity Toward Diverse 1, 1-Bisborylalkanes. *Chem. Eur. J.* **26**, 5360-5364 (2020).

5. Ho, H. E., Asao, N., Yamamoto, Y. & Jin, T. Carboxylic acid-catalyzed highly efficient and selective hydroboration of alkynes with pinacolborane. *Org. Lett.* **16**, 4670-4673 (2014).

6. Tao, L. *et al.* Rhodium-catalyzed deoxygenation and borylation of ketones: a combined experimental and theoretical investigation. *J. Am. Chem. Soc.* **142**, 18118-18127 (2020).

7. Kurahashi, T. *et al.* Geminal dimetalation of alkylidene-type carbenoids with silylboranes and diborons. *Tetrahedron* **58**, 6381-6395 (2002).

8. Shimizu, M. *et al.* Stereoselective cross-coupling reaction of 1, 1-diboryl-1-alkenes with electrophiles: A highly stereocontrolled approach to 1, 1, 2-triaryl-1-alkenes. *J. Am. Chem. Soc.* **127**, 12506-12507 (2005).

9. Morinaga, A., Nagao, K., Ohmiya, H. & Sawamura, M. Synthesis of 1, 1-Diborylalkenes through a Brønsted Base Catalyzed Reaction between Terminal Alkynes and Bis (pinacolato) diboron. *Angew. Chem. Int. Ed.* **127**, 16085-16088 (2015).

10. Chen, Q. *et al.* Remarkable catalytic property of nanoporous gold on activation of diborons for direct diboration of alkynes. *Org. Lett.* **15**, 5766-5769 (2013).

11. Zhang, M., Yao, Y., Stang, P. J. & Zhao, W. Divergent and stereoselective synthesis of tetraarylethylenes from vinylboronates. *Angew. Chem. Int. Ed.* **59**, 20090-20098 (2020).

12. Kumar, N., Reddy, R. R. & Masarwa, A. Stereoselective Desymmetrization of gem-Diborylalkanes by “Trifluorination”. *Chem. Eur. J.* **25**, 8008-8012 (2019).

13. Wen, H., Zhang, L., Zhu, S., Liu, G. & Huang, Z. Stereoselective Synthesis of Trisubstituted Alkenes via Cobalt-Catalyzed Double Dehydrogenative Borylations of 1-Alkenes. *ACS Catal.* **7**, 6419-6425 (2017).

14. Yoshida, H., Takemoto, Y. & Takaki, K. A masked diboron in Cu-catalysed borylation reaction: highly regioselective formal hydroboration of alkynes for synthesis of branched alkenylborons. *Chem. Commun.* **50**, 8299-8302 (2014).

15. Roseblade, S. J., Smilović, I. G. & Časar, Z. Study of chemoselective asymmetric hydrogenation of (1-bromo-1-alkenyl) boronic esters with iridium–PˆN complexes. *Tetrahedron* **70**, 2654-2660 (2014).

16. Gazić Smilović, I. *et al.* Iridium-Catalyzed Chemoselective and Enantioselective Hydrogenation of (1-Chloro-1-Alkenyl) Boronic Esters. *Angew. Chem. Int. Ed.* **124**, 1038-1042 (2012).

17. Woerly, E. M., Miller, J. E. & Burke, M. D. (1-Bromovinyl)-MIDA boronate: a readily accessible and highly versatile building block for small molecule synthesis. *Tetrahedron* **69**, 7732-7740 (2013).

18. Hanania, N., Nassir, M., Eghbarieh, N. & Masarwa, A. A Stereodivergent Approach to the Synthesis of *gem*-Diborylcyclopropanes. *Chem. Eur. J.* **28**, e202202748 (2022).
